# Supplementary material for: Migratory functionalization of unactivated alkyl bromides for construction of all-carbon quaternary centers via transposed tert-C-radicals
Source: Nat Commun. 2020 Sep 25;11:4860. doi: 10.1038/s41467-020-18658-4 (PMC7519689; doi:10.1038/s41467-020-18658-4)
Supplement: Supplementary file 1 — Supplementary Information [file 41467_2020_18658_MOESM1_ESM.pdf]

Supplementary Information for

Migratory Functionalization of Unactivated Alkylbromides for Construction of All-Carbon Quaternary Centers *via* Transposed *tert*-C-Radicals

Chuan Zhu et al.

Supplementary Methods

General information

All operations were performed under a nitrogen atmosphere unless otherwise specified. ^{1}H, ^{13}C and ^{19}F-NMR spectra were recorded on a Bruker 400 (400 MHz for ^{1}H, 100 MHz for ^{13}C and 376 MHz for ^{19}F) or a JEOL ECX-400 (400 MHz for ^{1}H, 100 MHz for ^{13}C and 376 MHz for ^{19}F) spectrometer using residue solvent as internal reference. Silica gel (200~300 mesh) was used for flash column chromatography. High resolution mass analyses (ESI+) were performed on a Waters mass spectrometer.

Reagents: Unless otherwise noted, commercial reagents were used as received. Dehydrated DMA was purchased from Energy®. Toluene, acetonitrile and dichloromethane were purified by Vigor® solvent purification system. Tetrahydrofuran (THF) was freshly distilled from Na/benzophenone under nitrogen atmosphere. Unless otherwise noted, all reactions are performed under nitrogen.

Preparation of Substrates

Structure of alkylbromide

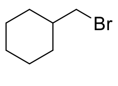
1a

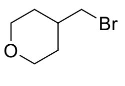
1b

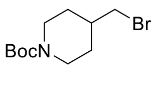
1c

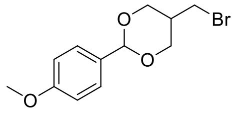
1d

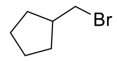
1e

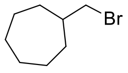
1f

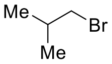
1g

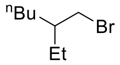
1h

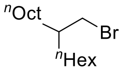
1i

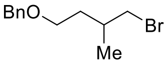
1j

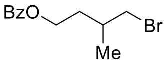
1k

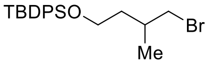
1l

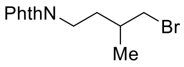
1m

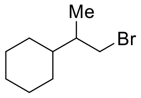
1n

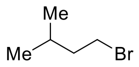
1o

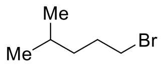
1p

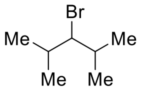
1q

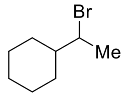
1r

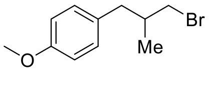
1s

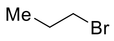
1t

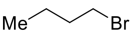
1u

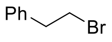
1v

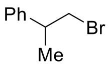
1w

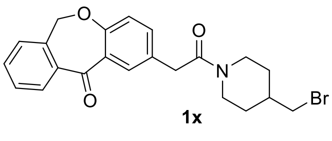
1x

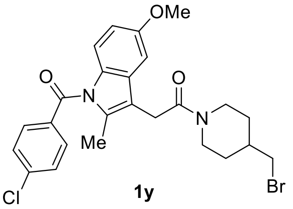
1y

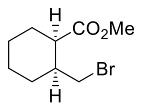
1z

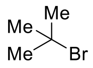
1aa

1a, 1b, 1c, 1e, 1g, 1h, 1i, 1o, 1p, 1t, 1u, 1v, 1w, 1aa were purchased from commercial suppliers and used

as received.

Preparation of compound 1d

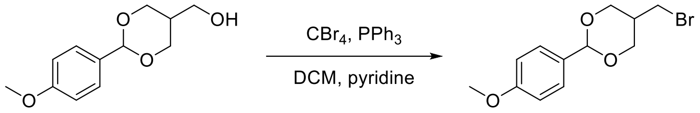

An oven-dried 50 mL flask equiped with magnetic stirring bar was evacuated and refilled with nitrogen (3 times). To the flask was charged S1[1] (0.45 g, 2.0 mmol, 1.0 equiv), anhydrous DCM (2 mL), pyridine (0.15 mL), CBr_4 (0.99 g, 3.0 mmol, 1.5 equiv), the resulting clear solution was cooled to 0 ℃ and stirred for 5 min. PPh_3 (0.5 g, 2 mmol, 1.0 equiv) in anhydrous DCM (2 mL) was then dropwise at 0 ℃ and then the reaction mixture was allowed to warm to room temperature and stirred for 3 hours.

The reaction mixture was quenched by adding H_2O (10 mL) followed by extraction with DCM (3 × 10 mL). The combined organic layers were dried with anhydrous Na_2SO_4, filtered, and evaporated to dryness under reduced pressure. The crude residue was purified by column chromatography on silica gel (petroleum ether/ethyl acetate = 4 : 1) to afford compound **1d** (0.38 g, 1.34 mmol) in 67% yield as white solid. **^{1}H NMR (400 MHz, $CDCl_3$):** δ = 7.45 – 7.36 (m, 2H), 6.94 – 6.85 (m, 2H), 5.37 (s, 1H), 4.37 – 4.31 (m, 2H), 3.80 (s, 3H), 3.68 (t, J = 11.3 Hz, 2H), 3.19 (d, J = 6.5 Hz, 2H), 2.56 – 2.45 (m, 1H). **HRMS (ESI, m/z):** calcd for $C_{12}H_{16}BrO_3$ $[M+H]^+$: 287.0283, found: 287.0282.

Preparation of compound 1f

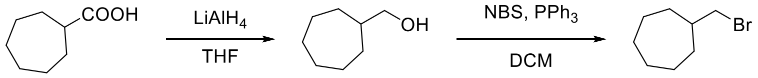

1) To an oven-dried 150 mL round-bottom flask equipped with magnetic stirring bar was charged cycloheptanecarboxylic acid (1.4 g, 10 mmol, 1.0 equiv). The flask was sealed, evacuated and refilled with nitro (3 times). Anhydrous THF (25 mL) was added and the resulting clear solution was cooled to 0 ℃ and stirred for 5 min at the same temperature. $LiAlH_4$ (0.65 g, 17 mmol, 1.7 equiv) in anhydrous THF (17 mL) was then added dropwise at 0 ℃ over 10 minutes. After stirring at 0 ℃ for 1 hour, the mixture was allowed to stir at room temperature for additional 16 hours. The reaction was quenched by adding H_2O (1.4 mL) dropwise over 10 min. Then 15% w/w NaOH (1.4 mL) was added and the reaction was allowed to stirred at room temperature for 20 minutes. Then the resulting suspension was diluted with H_2O (15 mL) followed by extraction with ethyl acetate (3 × 20 mL). The combined organic layers

were dried over anhydrous Na_2SO_4, filtered and evaporated to dryness under reduced pressure. The crude residue was purified by column chromatography on silica gel (petroleum ether/ethyl acetate = 10 : 1) to afford compound **S2** (1.2 g, 9.4 mmol) in 94% yield as colorless liquid. **^{1}H NMR (400 MHz, CDCl$_3$):** δ = 3.38 (d, *J* = 6.6 Hz, 2H), 1.94 (s, 1H), 1.76 – 1.67 (m, 2H), 1.71 – 1.49 (m, 5H), 1.51 – 1.37 (m, 4H), 1.19 – 1.10 (m, 2H). **^{13}C NMR (100 MHz, CDCl$_3$):** δ = 68.7, 42.2, 30.8, 28.7, 26.6. **HRMS (ESI, m/z):** calcd for $C_8H_{17}O$ $[M+H]^+$: 129.1279, found: 129.1285.

2) An oven-dried 50 mL round-bottom flask equiped with magnetic stirring bar was evacuated and refilled with nitrogen (3 times). To the flask was charged **S2** (0.64 g, 5.0 mmol, 1.0 equiv), PPh_3 (1.57 g, 6.0 mmol, 1.2 equiv) and DCM (10 mL) under nitrogen atmosphere. The resulting clear solution was cooled to 0 ℃ and stirred for 5 min. NBS (1.07 g, 6 mmol, 1.2 equiv) was then added by portions at 0 ℃ over 5 min against a nitrogen flow. The reaction was allowed to warm to room temperature and stirred for 3 hours. Then the reaction mixture was quenched by adding H_2O (10 mL) followed by extraction with DCM (3 × 10 mL). The combined organic layers were dried with anhydrous Na_2SO_4, filtered, and evaporated to dryness under reduced pressure. The crude residue was purified by column chromatography on silica gel (petroleum ether) to afford compound **1f** (0.86 g, 4.5 mmol) in 90% yield as colorless liquid. **^{1}H NMR (400 MHz, CDCl$_3$):** δ = 3.32 (d, *J* = 5.9 Hz, 2H), 1.90 – 1.80 (m,3H), 1.71 – 1.63 (m, 2H), 1.61 – 1.41 (m, 6H), 1.36 – 1.26 (m, 2H). **^{13}C NMR (100 MHz, CDCl$_3$):** δ = 42.1, 41.8, 33.1, 28.4, 26.4. **HRMS (ESI, m/z):** calcd for $C_8H_{16}Br$ $[M+H]^+$: 191.0435, found: 191.0428.

Preparation of compound 1j

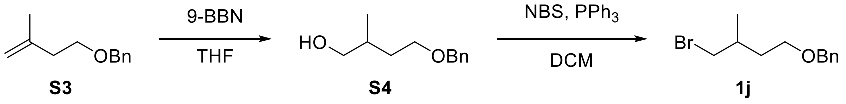

1) An oven-dried 150 mL round-bottom flask equiped with magnetic stirring bar was evacuated and refilled with nitrogen (3 times). To the flask a solution of **S3**[2] (1.76 g, 10 mmol, 1.0 equiv) in anhydrous THF (10 mL) was added via syringe. After cooling to 0 ℃ and stirring for 5 min, to this solution 9-BBN solution in THF (0.5 mol/L, 30 mL, 15 mmol, 1.5 equiv) was added dropwise. The reaction mixture was then stirred at 0 ℃ additional 30 min, followed by stirring at 35 ℃ for 8 h. After cooling to 0 ℃, the reaction was quenched by adding a NaOH solution (3 mol/L, 16 mL, 48 mmol) followed by the slow addition of H_2O_2 (30 wt%, 16 mL) solution. After stirring at room temperature for 90 min, the aqueous

layer was separated and extracted with ethyl acetate (2 × 30 mL). The combined organic layers were washed with brine (60 mL), dried over anhydrous Na_2SO_4, evaporated to dryness under reduced pressure. The crude residue was purified by flash chromatography (petroleum ether/ethyl acetate = 9 : 1) to give **S4** (1.55 g, 8.0 mmol) in 80% yield as a colorless liquid. **^{1}H NMR (400 MHz, CDCl$_3$):** δ = 7.40 – 7.23 (m, 5H), 4.52 (s, 2H), 3.62 – 3.57 (m, 1H), 3.55 – 3.48 (m, 2H), 3.45 – 3.41 (m, 1H), 2.35 (s, 1H), 1.87 – 1.76 (m, 1H), 1.74 – 1.66 (m, 1H), 1.61 – 1.54 (m, 1H), 0.92 (d, J = 6.8 Hz, 3H). **^{13}C NMR (100 MHz, CDCl$_3$):** δ = 138.1, 128. 6, 127.9, 127.9, 73.3, 68.8, 68.2, 34.3, 34.2, 17.3. **HRMS (ESI, m/z):** calcd for $C_{12}H_{18}NaO_2$ $[M+Na]^+$: 217.1204, found: 217.1204.

2) An oven-dried 50 mL round-bottom flask equiped with magnetic stirring bar was evacuated and refilled with nitrogen (3 times). To the flask was charged **S4** (0.39 g, 2.0 mmol, 1.0 equiv), PPh_3 (0.63 g, 2.4 mmol, 1.2 equiv) and DCM (5 mL) under nitrogen atmosphere. The resulting clear solution was cooled to 0 ℃ and stirred for 5 min. NBS (0.43 g, 2.4 mmol, 1.2 equiv) was then added by portions at 0 ℃ over 5 min. The reaction was allowed to warm to room temperature and stirred for 3 hours. Then the reaction mixture was quenched by adding H_2O (10 mL) followed by extraction with DCM (3 × 10 mL). The combined organic layers were dried with anhydrous Na_2SO_4, filtered, and evaporated to dryness under reduced pressure. The crude residue was purified by column chromatography on silica gel (petroleum ether/ethyl acetate = 50 : 1) to afford compound **1j** (0.44 g, 1.6 mmol) in 80% yield as colorless liquid. **^{1}H NMR (400 MHz, CDCl$_3$):** δ = 7.39 – 7.28 (m, 5H), 4.51 (s, 2H), 3.58 – 3.49 (m, 2H), 3.47 – 3.43 (m, 1H), 3.40 – 3.36 (m, 1H), 2.09 – 2.02 (m, 1H), 1.85 – 1.76 (m, 1H), 1.61 – 1.52 (m, 1H), 1.04 (d, J = 6.7 Hz, 3H). **^{13}C NMR (100 MHz, CDCl$_3$):** δ = 138.5, 128.5, 127.7, 73.1, 67.9, 41.7, 34.7, 32.2, 18.9. **HRMS (ESI, m/z):** calcd for $C_{12}H_{17}BrNaO$ $[M+H]^+$: 279.0360, found: 279.0358.

Preparation of compound 1k

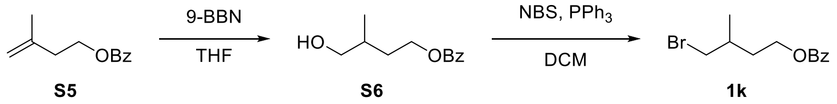

1) An oven-dried 150 mL round-bottom flask equipped with magnetic stirring bar was evacuated and refilled with nitrogen (3 times). To the flask were charged **S5**[3] (1.90 g, 10 mmol, 1.0 equiv) and anhydrous THF (10 mL) via syringe. After cooling to 0 ℃ and stirring for 5 min, to this solution 9-BBN solution in THF (0.5 mol/L, 30 mL, 15 mmol, 1.5 equiv) was added dropwise. The reaction mixture was

then stirred at 0 ℃ additional 30 min, followed by stirring at 35 ℃ for 8 h. After cooling to 0 ℃, the reaction was quenched by adding a NaOH solution (3 mol/L, 16 mL, 48 mmol) followed by the slow addition of H_2O_2 (30 wt%, 16 mL) solution. After stirring at room temperature for 90 min, the aqueous layer was separated and extracted with ethyl acetate (2 × 30 mL). The combined organic layers were washed with brine (60 mL), dried over anhydrous Na_2SO_4, evaporated to dryness under reduced pressure. The crude residue was purified by flash chromatography (petroleum ether/ethyl acetate = 9 : 1) to give **S6** (1.79 g, 8.6 mmol) in 86% yield as a colorless liquid. **^{1}H NMR (400 MHz, CDCl$_3$):** δ = 8.05 – 7.96 (m, 2H), 7.54 – 7.50 (m, 1H), 7.42 - 7.38 (m, 2H), 4.42 – 4.32 (m, 2H), 3.55 – 3.47 (m, 2H), 2.49 (s, 1H), 1.96 – 1.88 (m, 1H), 1.86 – 1.80 (m, 1H), 1.62 – 1.52 (m, 1H), 0.98 (d, *J* = 6.7 Hz, 3H). **^{13}C NMR (100 MHz, CDCl$_3$):** δ = 166.9, 133.1, 130.3, 129.6, 128.5, 67.8, 63.5, 33.0, 32.2, 16.7. **HRMS (ESI, m/z):** calcd for $C_{12}H_{17}O_3$ $[M+H]^+$: 209.1178, found: 209.1172.

2) An oven-dried 50 mL round-bottom flask equipped with magnetic stirring bar was evacuated and refilled with nitrogen (3 times). To the flask was charged **S6** (1.04 g, 5.0 mmol, 1.0 equiv), PPh_3 (1.70 g, 6.5 mmol, 1.3 equiv) and DCM (12 mL) under nitrogen atmosphere. The resulting clear solution was cooled to 0 ℃ and stirred for 5 min. NBS (1.16 g, 6.5 mmol, 1.3 equiv) was then added by portions at 0 ℃ over 5 min. The reaction was allowed to warm to room temperature and stirred for 3 hours. Then the reaction mixture was quenched by adding H_2O (10 mL) followed by extraction with DCM (3 × 10 mL). The combined organic layers were dried with anhydrous Na_2SO_4, filtered, and evaporated to dryness under reduced pressure. The crude residue was purified by column chromatography on silica gel (petroleum ether/ethyl acetate = 50 : 1) to afford compound **1k** (1.16 g, 4.3 mmol) in 86% yield as colorless liquid. The ^{1}H NMR data is in accordance with the literature. **^{1}H NMR (400 MHz, CDCl$_3$):** δ = 8.08 – 8.00 (m, 2H), 7.61 – 7.51 (m, 1H), 7.49 – 7.39 (m, 2H), 4.46 – 4.29 (m, 2H), 3.45 (d, *J* = 5.1 Hz, 2H), 2.11 – 2.02 (m, 1H), 2.05 – 1.92 (m, 1H), 1.79 – 1.64 (m, 1H), 1.11 (d, *J* = 6.6 Hz, 3H). **^{13}C NMR (100 MHz, CDCl$_3$):** δ = 166.7, 133.1, 130.3, 129.7, 128.5, 62.9, 41.0, 33.6, 32.3, 18.8. **HRMS (ESI, m/z):** calcd for $C_{12}H_{16}BrO_2$ $[M+H]^+$: 271.0334, found: 271.0329.

Preparation of compound 1l

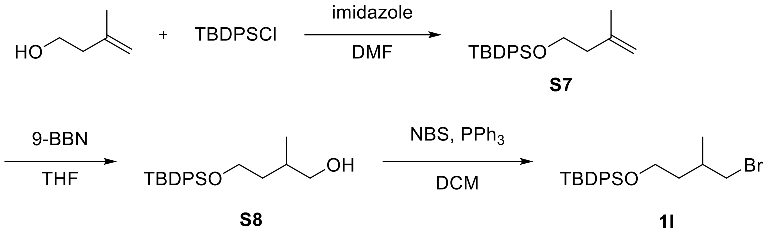

1) To an oven-dried 150 mL round-bottom flask equipped with magnetic stirring bar charged TBDPSCl (5.50 g, 20 mmol, 2.0 equiv), imidazole (1.36 g, 20 mmol, 2.0 equiv). The flask was sealed, evacuated and refilled with nitrogen (3 times). Anhydrous DMF (10 mL) and 3-methyl-3-buten-1-ol (0.9 mL, 10 mmol, 1.0 equiv) were added. The reaction mixture was stirred at room temperature for 6 hours. The mixture was extracted with petroleum ether (3 ×15 mL). The combined organic layers were dried with Na_2SO_4, filtered, evaporated to dryness under reduced pressure. The crude residue was purified by column chromatography on silica gel (petroleum ether) to afford compound **S7** (5.80 g, 18 mmol) in 90% yield as colorless liquid. **^{1}H NMR (400 MHz, $CDCl_3$):** δ = 7.72 – 7.63 (m, 4H), 7.48 – 7.33 (m, 6H), 4.75 – 4.73 (m, 1H), 4.69 – 4.67 (m, 1H), 3.76 (t, J = 6.9 Hz, 2H), 2.27 (t, J = 6.9 Hz, 2H), 1.68 (s, 3H), 1.04 (s, 9H). **^{13}C NMR (100 MHz, $CDCl_3$):** δ = 143.1, 135.7, 134.1, 129. 7, 127.7, 111.8, 62.8, 41.0, 26.9, 22.9, 19.3. **HRMS (ESI, m/z):** calcd for $C_{21}H_{29}OSi$ $[M+H]^+$: 325.1988, found: 325.1979.

2) An oven-dried 150 mL round-bottom flask equiped with magnetic stirring bar was evacuated and refilled with nitrogen (3 times). To the flask a solution of **S7** (3.24 g, 10 mmol, 1.0 equiv) in anhydrous THF (10 mL) was added via syringe. After cooling to 0 ℃ and stirring for 5 min, to this solution 9-BBN solution in THF (0.5 mol/L, 30 mL, 15 mmol, 1.5 equiv) was added dropwise. The reaction mixture was then stirred at 0 ℃ additional 30 min, followed by stirring at 35 ℃ for 8 h. After cooled to 0 ℃, the reaction was quenched by adding a NaOH solution (3 mol/L, 16 mL, 48 mmol) followed by the slow addition of H_2O_2 (30 wt%, 16 mL) solution. After stirring at room temperature for 90 min, the aqueous layer was separated and extracted with ethyl acetate (2 × 30 mL). The combined organic layers were washed with brine (60 mL), dried over anhydrous Na_2SO_4, evaporated to dryness under reduced pressure. The crude residue was purified by flash chromatography (petroleum ether/ethyl acetate = 10 : 1) to give **S8** (3.10 g, 9.0 mmol) in 90% yield as a colorless liquid. **^{1}H NMR (400 MHz, $CDCl_3$):** δ = 7.73 – 7.64 (m, 4H), 7.47 – 7.37 (m, 6H), 3.82 – 3.65 (m, 2H), 3.58 – 3.43 (m, 2H), 2.49 (s, 1H), 1.90 – 1.82 (m, 1H), 1.68 – 1.59 (m, 1H), 1.54 – 1.46 (m, 1H), 1.06 (s, 9H), 0.91 (d, J = 6.8 Hz, 3H).**^{13}C NMR (100 MHz, $CDCl_3$):** δ = 135.7, 133.5, 129.9, 127.8, 68.4, 62.6, 36.9, 34.1, 26.9, 19.2, 17.3. **HRMS (ESI,**

m/z): calcd for $C_{21}H_{31}O_2Si$ $[M+H]^+$: 343.2093, found: 343.2091.

3) An oven-dried 50 mL round-bottom flask equiped with magnetic stirring bar was evacuated and refilled with nitrogen (3 times). To the flask was charged **S8** (1.70 g, 5.0 mmol, 1.0 equiv), PPh_3 (1.57, 6.0 mmol, 1.2 equiv) and DCM (12 mL) against a nitrogen flow. The resulting clear solution was cooled to 0 °C and stirred for 5 min. NBS (1.07 g, 6 mmol, 1.2 equiv) was then added by portions at 0 °C under a nitrogen flow over 5 min. The reaction was allowed to warm to room temperature and stirred for 3 hours. Then the reaction mixture was quenched by adding H_2O (10 mL), followed by extraction with DCM (3 × 10 mL). The combined organic layers were dried with anhydrous Na_2SO_4, filtered, and evaporated to dryness under reduced pressure. The crude residue was purified by column chromatography on silica gel (petroleum ether/ethyl acetate = 50 : 1) to afford compound **1l** (1.70 g, 4.2 mmol) in 85% yield as colorless liquid. **^{1}H NMR (400 MHz, $CDCl_3$):** δ = 7.70 – 7.65 (m, 4H), 7.47 – 7.36 (m, 6H), 3.71 (t, *J* = 5.3 Hz, 2H), 3.45 – 3.42 (M, 1H), 3.37 - 3.33 (m, 1H), 2.12 – 2.04 (m, 1H), 1.77 – 1.69 (m, 1H), 1.55 – 1.40 (m, 1H), 1.06 (s, 9H), 1.00 (d, *J* = 6.7 Hz, 3H). **^{13}C NMR (100 MHz, $CDCl_3$):** δ = 135.7, 133.9, 129.8, 127.8, 61.6, 41.9, 37.5, 32.0, 27.0, 19.3, 18.9. **HRMS (ESI, m/z):** calcd for $C_{21}H_{30}BrOSi$ $[M+H]^+$: 405.1249, found: 405.1241.

Preparation of compound 1m

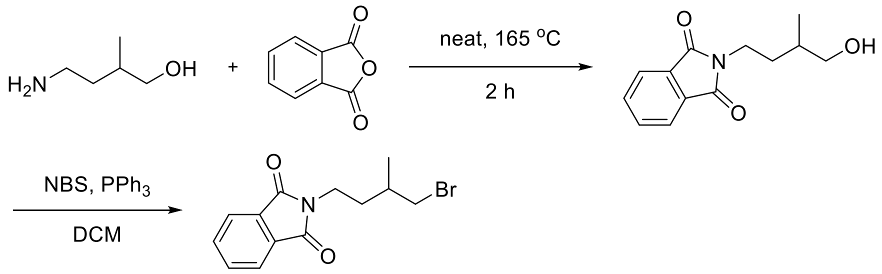

1m

An oven-dried 25 mL Schlenk tube equipped with magnetic stirring bar was charged 4-amino-2-methyl-1-butanol (1.13 g, 11 mmol, 1.1 equiv) and succinic anhydride (1.48 g, 10 mmol, 1.0 equiv). The Schlenk tube was sealed, evacuated and refilled with nitrogen (3 times). The reaction mixture was stirred at 165 ^{o}C for 2 hours. The reaction was cooled to room temperature. Without any treatment, the crude alcohol (1.17 g, 5 mmol, 1.0 equiv) and PPh_3 (1.57 g, 6.0 mmol, 1.2 equiv) was added to an oven-dried 50 mL round-bottom flask equipped with magnetic stirring bar, the flask was sealed, evacuated and refilled with nitrogen (3 times). Anhydrous DCM (12 mL) was added via syringe. The resulting clear

solution was cooled to 0 ℃ and stirred for 5 min. NBS (1.07 g, 6.0 mmol, 1.2 equiv) was then added by portions at 0 ℃ over 5 min under a nitrogen flow. The reaction was allowed to warm to room temperature and stirred for 3 hours. Then the reaction mixture was quenched by adding H_2O (10 mL) followed by extraction with DCM (3 × 10 mL). The combined organic layers were dried with anhydrous Na_2SO_4, filtered, and evaporated to dryness under reduced pressure. The crude residue was purified by column chromatography on silica gel (petroleum ether/ethyl acetate = 20 : 1) to afford compound **1m** (1.89 g, 6.4 mmol) in 64% yield as colorless liquid. **^{1}H NMR (400 MHz, $CDCl_3$):** δ = 7.77 - 7.74 (m, 2H), 7.66 – 7.64 (m, 2H), 3.66 (t, *J* = 7.0 Hz, 2H), 3.44 – 3.30 (m, 2H), 1.85 – 1.73 (m, 2H), 1.59 – 1.50 (m, 1H), 1.04 (d, *J* = 6.5 Hz, 3H). **^{13}C NMR (100 MHz, $CDCl_3$):** δ = 168.4, 134.1, 132.1, 123.3, 40.8, 35.8, 33.6, 32.6, 18.6. **HRMS (ESI, m/z):** calcd for $C_{13}H_{15}NBrO_2$ $[M+H]^+$: 296.0286, found: 296.0291.

Preparation of compound 1n

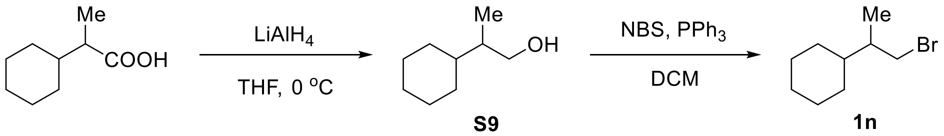

1) To an oven-dried 150 mL round-bottom flask equipped with magnetic stirring bar was charged cycloheptanecarboxylic acid (0.47 g, 3 mmol, 1.0 equiv). The flask was sealed, evacuated and refilled with nitrogen (3 times). Anhydrous THF (10 mL) was added via syringe and the resulting clear solution was cooled to 0 ℃ and stirred for 5 min at the same temperature. $LiAlH_4$ (0.19 g, 5.1 mmol, 1.7 equiv) in anhydrous THF (10 mL) was then added dropwise at 0 ℃ over 10 minutes. After stirring at 0 ℃ for 1 hour, the mixture was allowed to stir at room temperature for additional 16 hours. The reaction was quenched by adding H_2O (0.7 mL) dropwise over 10 min. Then 15% w/w NaOH (0.7 mL) was added and the reaction was allowed to stirred at room temperature for 20 minutes. Then the resulting suspension was diluted with H_2O (10 mL) followed by extraction with ethyl acetate (3 × 10 mL). The combined organic layers were dried over anhydrous Na_2SO_4, filtered and evaporated to dryness under reduced pressure. The crude residue was purified by column chromatography on silica gel (petroleum ether/ethyl acetate = 10 : 1) to afford compound **S9** (0.40 g, 2.9 mmol) in 95% yield as colorless liquid. **^{1}H NMR (400 MHz, $CDCl_3$):** δ = 3.62 – 3.58 (m, 1H), 3.47 – 3.43 (m, 1H), 1.76 – 1.69 (m, 2H), 1.66 – 1.61 (m, 3H), 1.53 – 1.44 (m, 1H), 1.39 (s, 1H), 1.36 – 1.28 (m, 1H), 1.26 – 1.03 (m, 4H), 1.02 – 0.92 (m, 1H), 0.88 (d, *J* = 6.9 Hz, 3H). **^{13}C NMR (100 MHz, $CDCl_3$):** δ = 66.4, 41.0, 39.4, 31.1, 28.9, 26.9, 26.8, 26.7,

13.5. **HRMS (ESI, m/z):** calcd for $C_9H_{18}NaO$ $[M+Na]^+$: 165.1255, found: 165.1263.

2) An oven-dried 50 mL round-bottom flask equiped with magnetic stirring bar was evacuated and refilled with nitrogen (3 times). To the flask was charged **S9** (0.43 g, 3.0 mmol, 1.0 equiv), PPh_3 (0.94 g, 3.6 mmol, 1.2 equiv) and DCM (10 mL) under a nitrogen flow. The resulting clear solution was cooled to 0 ℃ and stirred for 5 min. NBS (0.64 g, 3.6 mmol, 1.2 equiv) was then added by portions at 0 ℃ over 5 min under a nitrogen flow. The reaction was allowed to warm to room temperature and stirred for 3 hours. Then the reaction mixture was quenched by adding H_2O (10 mL) followed by extraction with DCM (3 × 10 mL). The combined organic layers were dried with anhydrous Na_2SO_4, filtered, and evaporated to dryness under reduced pressure. The crude residue was purified by column chromatography on silica gel (petroleum ether) to afford compound **1n** (0.51 g, 2.5 mmol) in 84% yield as colorless liquid. **^{1}H NMR (400 MHz, CDCl$_3$):** δ = 3.48 – 3.44 (m, 1H), 3.39 – 3.34 (m, 1H), 1.76 – 1.59 (m, 6H), 1.43 – 1.34 (m, 1H), 1.31 – 1.04 (m, 4H), 0.99 (d, J = 6.8 Hz, 3H), 0.96 – 0.86 (m, 1H).. **^{13}C NMR (100 MHz, CDCl$_3$):** δ = 40.9, 40.6, 40.4, 30.8, 28.9, 26.6, 26.5, 15.9. **HRMS (ESI, m/z):** calcd for $C_9H_{18}Br$ $[M+H]^+$: 205.0592, found: 205.0591.

Preparation of compound 1q

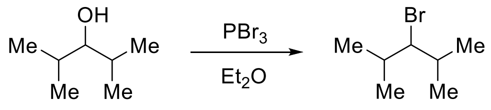

1q

An oven-dried 150 mL round-bottom flask equipped with magnetic stirring bar was evacuated and refilled with nitrogen (3 times). To the flask was charged 2,4-dimethyl-3-pentanol (2.8 mL, 20 mmol, 1.0 equiv) and Et_2O (40 mL). The resulting clear solution was cooled to 0 ℃ and stirred for 5 min. After PBr_3 (0.75 mL, 8 mmol, 0.4 equiv) was added dropwise at 0 ℃, the reaction mixture was allowed to warmed to room temperature and stirred for additional 3 h. The mixture was then diluted with diethyl ether (30 mL), washed with saturated aqueous $NaHCO_3$ solution (30 mL), dried over anhydrous Na_2SO_4 and evaporated to dryness under reduced pressure. The crude residue was purified by column chromatography on silica gel (petroleum ether) to afford compound **1q** (0.71 g, 4.0 mmol) in 20% yield as colorless liquid. **^{1}H NMR (400 MHz, CDCl$_3$):** δ = 3.76 (t, J = 6.1 Hz, 1H), 2.05 – 1.88 (m, 2H), 1.02 (d, J = 12.4 Hz, 6H), 1.00 (d, J = 12.5 Hz, 6H). **^{13}C NMR (100 MHz, CDCl$_3$):** δ = 32.5, 21.8, 19.8. **HRMS (ESI, m/z):** calcd for $C_7H_{15}BrNa$ $[M+Na]^+$: 201.0255, found: 201.0260.

Preparation of compound 1r

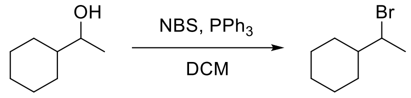

S10 1r

An oven-dried 50 mL round-bottom flask equipped with magnetic stirring bar was evacuated and refilled with nitrogen (3 times). To the flask was charged **S10**[4] (0.64 g, 5.0 mmol, 1.0 equiv), PPh_3 (1.57 g, 6.0 mmol, 1.2 equiv) and DCM (10 mL) under nitrogen atmosphere. The resulting clear solution was cooled to 0 ℃ and stirred for 5 min. NBS (1.07 g, 6.0 mmol, 1.2 equiv) was added by portions at 0 ℃ over 5 min. The reaction was allowed to warm to room temperature and stirred for 3 hours. Then the reaction mixture was quenched by adding H_2O (10 mL) followed by extraction with DCM (3 × 10 mL). The combined organic layers were dried with anhydrous Na_2SO_4, filtered, and evaporated to dryness under reduced pressure. The crude residue was purified by column chromatography on silica gel (petroleum ether) to afford compound **1r** (0.38 g, 2.0 mmol) in 40% yield as colorless liquid. **^{1}H NMR (400 MHz, CDCl$_3$):** δ = 4.13 – 4.03 (m, 1H), 1.88 – 1.69 (m, 4H), 1.65 (d, *J* = 6.8 Hz, 3H), 1.49 – 1.41 (m, 1H), 1.29 – 1.01 (m, 6H). **^{13}C NMR (100 MHz, CDCl$_3$):** δ = 58.7, 46.0, 30.4, 29.8, 26.4, 26.2, 26.1, 23.4. **MS (EI, m/z):** calcd for $C_8H_{15}Br$ $[M]^+$: 190.04, found: 191.0.

Preparation of compound 1s

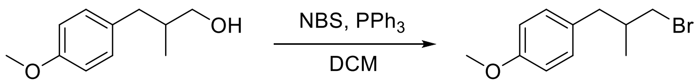

S11 1s

An oven-dried 50 mL round-bottom flask equipped with magnetic stirring bar was evacuated and refilled with nitrogen (3 times). To the flask was charged **S11**[5] (0.36 g, 2.0 mmol, 1.0 equiv), PPh_3 (0.63 g, 2.4 mmol, 1.2 equiv) and DCM (5 mL) under nitrogen atmosphere. The resulting clear solution was cooled to 0 ℃ and stirred for 5 min. NBS (0.43 g, 2.4 mmol, 1.2 equiv) was added by portions at 0 ℃ over 5 min. The reaction was allowed to warm to room temperature and stirred for 3 hours. Then the reaction mixture was quenched by adding H_2O (10 mL) followed by extraction with DCM (3 × 10 mL). The combined organic layers were dried with anhydrous Na_2SO_4, filtered, and evaporated to dryness under reduced pressure. The crude residue was purified by column chromatography on silica gel

(petroleum ether/ethyl acetate = 100 : 1) to afford compound **1s** (0.36 g, 1.5 mmol) in 75% yield as colorless liquid. **^{1}H NMR (400 MHz, CDCl$_3$):** δ = 7.15 – 7.05 (m, 2H), 6.89 – 6.79 (m, 2H), 3.80 (s, 3H), 3.39 – 3.36 (m, 1H), 3.32 – 3.28 (m, 1H), 2.72 – 2.66 (m, 1H), 2.53 – 2.48 (m, 1H), 2.10 – 1.99 (m, 1H), 1.03 (d, J = 6.6 Hz, 3H). **^{13}C NMR (100 MHz, CDCl$_3$):** δ = 158.1, 131.9, 130.2, 113.9, 55.4 (t, J = 3.9 Hz), 40.9, 40.1, 37.3, 18.8. **HRMS (ESI, m/z):** calcd for $C_{11}H_{16}BrO$ [M+H]$^+$: 243.0385, found: 243.0384.

Preparation of compound 1x

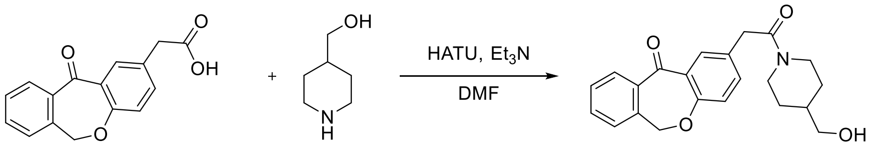

S12

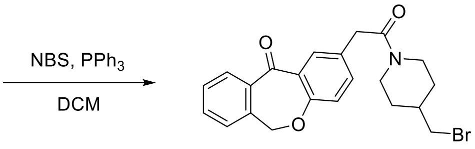

1x

1) An oven-dried 150 mL round-bottom flask equipped with magnetic stirring bar was charged isoxepac (1.34 g, 5 mmol, 1.0 equiv), 4-piperidinemethanol (0.86 g, 7.5 mmol, 1.5 equiv) and HATU (3.8 g, 10 mmol, 2.0 equiv). The flask was sealed, evacuated and refilled with nitrogen (3 times). DMF (50 mL) and Et_3N (2.1 mL, 15 mmol, 3.0 equiv) was added via syringe and the resulting suspension was stirred at room temperature for 6 hours. Then the reaction was quenched with H_2O (20 mL) and the aqueous layer was extracted with ethyl acetate (3 × 20 mL). The combined organic layers were washed with water (2 × 20 mL), dried over Na_2SO_4, filtered and evaporated to dryness under reduced pressure. The crude residue was purified by flash column chromatography (ethyl acetate) to afford compound **S12** (1.10 g, 3.0 mmol) in 60% yield as white solid. **^{1}H NMR (400 MHz, CDCl$_3$):** δ = 8.00 (d, J = 2.4 Hz, 1H), 7.84 – 7.82 (m, 1H), 7.54 – 7.50 (m, 1H), 7.44 – 7.40 (m, 1H), 7.38 – 7.35 (m, 1H), 7.33 – 7.31 (m, 1H), 6.98 (d, J = 8.4 Hz, 1H), 5.13 (s, 2H), 4.61 – 4.56 (m, 1H), 3.90 – 3.85 (m, 1H), 3.68 (s, 2H), 3.46 – 3.31 (m, 2H), 3.02 – 2.88 (m, 1H), 2.68 (s, 1H), 2.56 – 2.49 (m, 1H), 1.73 – 1.58 (m, 3H), 1.14 – 0.94 (m, 2H). **^{13}C NMR (100 MHz, CDCl$_3$):** δ = 191.1, 169.3, 160.3, 140.4, 136.2, 135.7, 133.0, 131.8, 129.5, 129.3, 129.1, 128.0, 125.1, 121.2, 73.6, 66.9, 46.2, 42.1, 39.7, 38.7, 29.2, 28.4. **HRMS (ESI, m/z):** calcd

for $C_{22}H_{24}NO_4$ $[M+H]^+$: 366.1705, found: 366.1709.

2) An oven-dried 50 mL round-bottom flask equipped with magnetic stirring bar was charged **S12** (0.73 g, 2 mmol, 1.0 equiv) and PPh_3 (0.63 g, 2.4 mmol, 1.2 equiv). The flask was sealed, evacuated and refilled with nitrogen (3 times). DCM (5 mL) was added under a nitrogen flow. The resulting clear solution was cooled to 0 ℃ and stirred for 5 min. NBS (0.43 g, 2.4 mmol, 1.2 equiv) was added by portions at 0 ℃ under a nitrogen flow over 5 min. The reaction was allowed to warm to room temperature and stirred for 3 hours. Then the reaction mixture was quenched by adding H_2O (10 mL) followed by extraction with DCM (3 × 10 mL). The combined organic layers were dried with anhydrous Na_2SO_4, filtered, and evaporated to dryness under reduced pressure. The crude residue was purified by column chromatography on silica gel (petroleum ether/ethyl acetate = 1 : 1) to afford compound **1x** (0.51 g, 1.2 mmol) in 60% yield as white solid. **^{1}H NMR (400 MHz, CDCl$_3$):** δ = 8.04 (d, *J* = 2.4 Hz, 1H), 7.89 – 7.87 (m, 1H), 7.58 – 7.54 (m, 1H), 7.49 – 7.45 (m, 1H), 7.43 -7.40 (m, 1H), 7.37 – 7.35 (m, 1H), 7.02 (d, *J* = 8.4 Hz, 1H), 5.18 (s, 2H), 4.72 – 4.66 (m, 1H), 3.97 – 3.91 (m, 1H), 3.73 (s, 2H), 3.31 – 3.22 (m, 2H), 3.04 – 2.97 (m, 1H), 2.60 – 2.53 (m, 1H), 1.89 – 1.81 (m,3H), 1.25 – 1.14 (m, 1H), 1.11 – 1.01 (m, 1H). **^{13}C NMR (100 MHz, CDCl$_3$):** δ = 191.0, 169.1, 160.4, 140.5, 136.1, 135.7, 132.9, 131.8, 129.6, 129.4, 129.1, 127.9, 125.2, 121.3, 73.8, 46.0, 41.8, 39.9, 38.6, 38.5, 31.3, 30.6. **HRMS (ESI, m/z):** calcd for $C_{22}H_{23}BrNO_3$ $[M+H]^+$: 428.0861, found: 428.0859.

Preparation of compound 1y

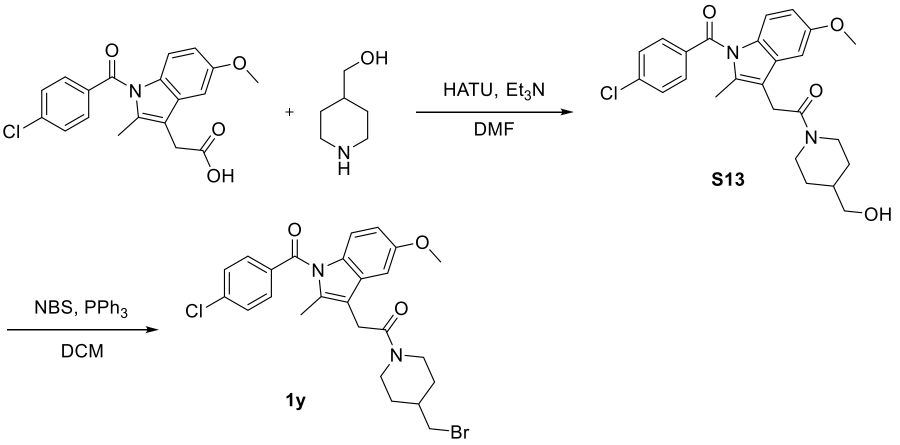

1) An oven-dried 150 mL round-bottom flask equipped with magnetic stirring bar was charged indometacin (1.78 g, 5 mmol, 1.0 equiv), 4-piperidinemethanol (0.86 g, 7.5 mmol, 1.5 equiv) and HATU (3.8 g, 10 mmol, 2.0 equiv). The flask was sealed, evacuated and refilled with nitrogen (3 times). DMF

(50 mL) and Et_3N (2.1 mL, 15 mmol, 3.0 equiv) was added via syringe and the resulting suspension was stirred at room temperature for 6 hours. Then the reaction was quenched with H_2O (20 mL) and the aqueous layer was extracted with ethyl acetate (3 × 20 mL). The combined organic layers were washed with water (2 × 20 mL), dried over Na_2SO_4, filtered and evaporated to dryness under reduced pressure. The crude residue was purified by flash column chromatography (ethyl acetate) to afford compound **S13** (1.73 g, 3.8 mmol) in 76% yield as yellow solid. **^{1}H NMR (400 MHz, $CDCl_3$):** δ = 7.71 – 7.61 (m, 2H), 7.48 – 7.44 (m, 2H), 7.01 – 6.99 (m, 1H), 6.84 – 6.81 (m, 1H), 6.66 – 6.63 (m, 1H), 4.69 – 4.65 (m, 1H), 3.99 – 3.93 (m, 1H), 3.81 (s, 3H), 3.76 – 3.67 (m, 2H), 3.46 – 3.42 (m, 2H), 3.06 – 2.99 (m, 1H), 2.65 – 2.52 (m, 1H), 2.37 (s, 3H), 1.75 – 1.68 (m, 3H), 1.53 (s, 1H), 1.16 – 1.06 (m, 1H), 1.02 – 0.94 (m, 1H). **^{13}C NMR (100 MHz, $CDCl_3$):** δ = 168.6, 168.4, 156.1, 139.4, 135.2, 134.0, 131.3, 130.9, 130.9, 129.2, 115.0, 113.7, 111.6, 101.6, 67.3, 55.8, 46.1, 42.2, 38.8, 30.6, 29.2, 28.5, 13.6. **HRMS (ESI, m/z):** calcd for $C_{25}H_{28}ClN_2O_4$ $[M+H]^+$: 455.1738, found: 455.1735.

2) An oven-dried 50 mL round-bottom flask equipped with magnetic stirring bar was charged **S13** (0.91 g, 2 mmol, 1.0 equiv) and PPh_3 (0.63 g, 2.4 mmol, 1.2 equiv). The flask was sealed, evacuated and refilled with nitrogen (3 times). DCM (5 mL) was added under a nitrogen flow. The resulting clear solution was cooled to 0 ℃ and stirred for 5 min. NBS (0.43 g, 2.4 mmol, 1.2 equiv) was added by portions at 0 ℃ under a nitrogen flow over 5 min. The reaction was allowed to warm to room temperature and stirred for 3 hours. Then the reaction mixture was quenched by adding H_2O (10 mL) followed by extraction with DCM (3 × 10 mL). The combined organic layers were dried with anhydrous Na_2SO_4, filtered, and evaporated to dryness under reduced pressure. The crude residue was purified by column chromatography on silica gel (petroleum ether/ethyl acetate = 1 : 1) to afford compound **1y** (0.93 g, 1.8 mmol) in 90% yield as yellow solid. **^{1}H NMR (400 MHz, $CDCl_3$):** δ = 7.67 – 7.59 (m, 2H), 7.48 – 7.39 (m, 2H), 6.98 (d, J = 2.6 Hz, 1H), 6.80 - 6.77 (m, 1H), 6.66 – 6.57 (m, 1H), 4.69 – 4.64 (m, 1H), 3.99 – 3.87 (m, 1H), 3.79 (s, 3H), 3.69 – 3.68 (m, 2H), 3.26 – 3.22 (m, 1H), 3.19 – 3.15 (m, 1H), 2.99 (t, J = 12.3 Hz, 1H), 2.54 (t, J = 12.2 Hz, 1H), 2.35 (s, 3H), 1.88 – 1.73 (m, 3H), 1.18 – 1.08 (m, 1H), 1.01 – 0.91 (m, 1H). **^{13}C NMR (100 MHz, $CDCl_3$):** δ = 168.6, 168.3, 156.1, 139.3, 135.1, 134.0, 131.3, 130.9, 130.8, 129.2, 115.0, 113.5, 111.6, 101.6, 55.8, 45.9, 42.0, 38.6, 38.4, 31.2, 30.7, 30.6, 13.5. **HRMS (ESI, m/z):** calcd for $C_{25}H_{27}BrClN_2O_3$ $[M+H]^+$: 517.0894, found: 517.0889.

Preparation of compound 1z

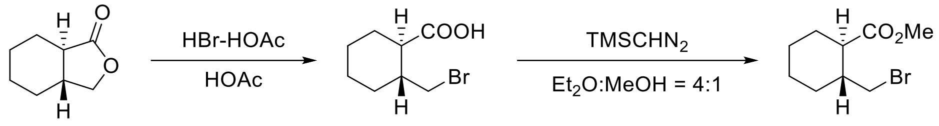

An oven-dried 50 mL round-bottom flask equipped with magnetic stirring bar was evacuated and refilled with nitrogen (3 times). To the flask was charged **S14**[6] (0.56 g, 4.0 mmol, 1.0 equiv), 33% w/w HBr in HOAc (12 mL) and HOAc (5 mL) under a nitrogen flow. The resulting clear solution was stirred at room temperature for 2 hours, then heated to 70 ℃ and stirred for 1.5 hours, after that stirred at room temperature for 8 hours. After diluted with H_2O (10 mL), extraction with ethyl acetate (3 × 10 mL) and H_2O (3 × 10 mL), the combined organic layers were dried with anhydrous Na_2SO_4, filtered, and evaporated to dryness under reduced pressure. The crude residue was dissolved in Et_2O (12 mL) and MeOH (3 mL), $TMSCHN_2$ (4 mL, 8 mmol, 2 mol/L in hexane, 2.0 equiv) was added at 0 °C. The mixture was stirred at 0 °C for 30 min. The volatile was removed under reduced pressure and the crude residue was purified by column chromatography or preparative TLC on silica gel (petroleum ether/ethyl acetate = 20 : 1) to afford compound **1z** (0.84 g, 3.6 mmol) in 90% yield as colorless liquid. **^{1}H NMR (400 MHz, $CDCl_3$):** δ = 3.70 (s, 3H), 3.42 – 3.33 (m, 2H), 2.35 – 2.29 (m, 1H), 2.01 – 1.83 (m, 3H), 1.84 – 1.71 (m, 2H), 1.52 – 1.41 (m, 1H), 1.36 – 1.21 (m, 3H). **^{13}C NMR (100 MHz, $CDCl_3$):** δ = 175.7, 51.8, 47.2, 40.0, 39.1, 30.0, 29.9, 25.3, 25.2. **HRMS (ESI, m/z):** calcd for $C_9H_{16}BrO_2$ $[M+H]^+$: 235.0334, found: 235.0338.

Structure of trifluoromethyl alkenes

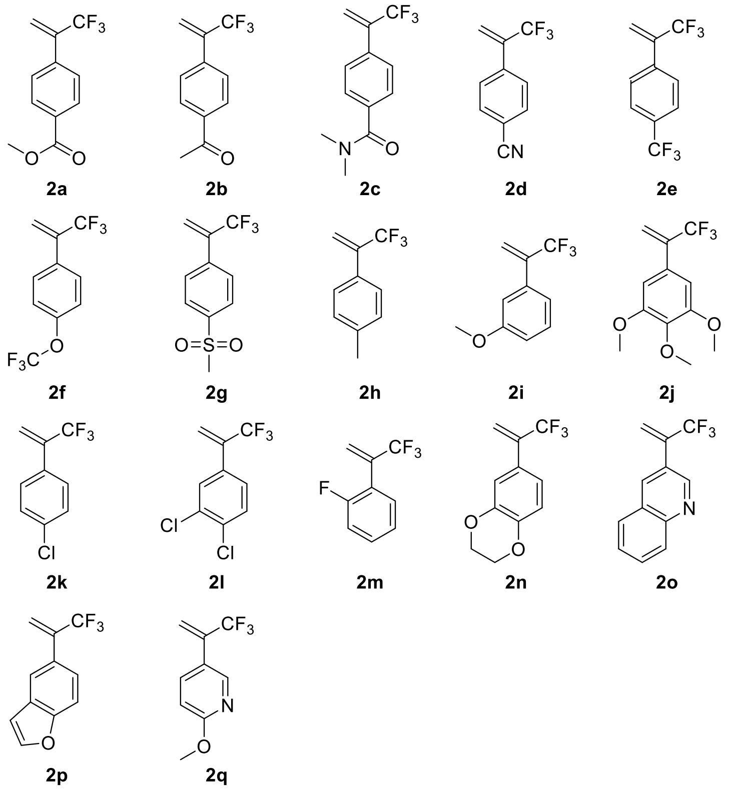

2a, **2b**,[7] **2c**,[8] **2d**,[7] **2e**,[9] **2f**, **2g**,[8] **2h**,[10] **2i**,[11] **2j**,[12] **2k**,[11] **2l**,[7] **2n**,[12] **2o**, **2p**,[7] and **2q**[8] were prepared according to the literature methods.

Preparation of compound 2m

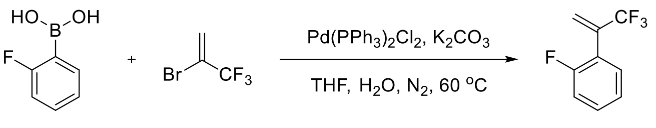

2m

To an oven-dried 250 mL three-necked round-bottom flask equipped with a condenser and magnetic stirring bar was charged 2-fluorophenylboronic acid (1.40 g, 10 mmol, 1.0 equiv), $Pd(PPh_3)_2Cl_2$ (0.07g, 0.1 mmol, 0.01 equiv) and K_2CO_3 (5.52g, 40 mmol, 4.0 equiv), the flask was evacuated and refilled with nitrogen (3 times). THF (30 mL), H_2O (20 mL) and 2-bromo-3,3,3-trifluoropropene (3.50 g, 20 mmol, 2.0 equiv) was added via syringe and the resulting solution was stirred at 60 °C for 24 hours. After cooling to room temperature, the reaction mixture was quenched with saturated aqueous NH_4Cl (10 mL), and extracted with ethyl acetate (3 × 20 mL). The combined organic layers were dried over anhydrous Na_2SO_4, filtered and evaporated to dryness under reduced pressure. The crude residue was purified by column chromatography on silica gel (petroleum ether) to afford **2m** (1.14 g, 6.0 mmol) in 60% yield as colorless liquid. **^{1}H NMR (400 MHz, $CDCl_3$):** δ = 7.41 – 7.34 (m, 2H), 7.20 – 7.11 (m, 2H), 6.19 (s, 1H), 5.79 (s,

1H). **^{19}F NMR (376 MHz, $CDCl_3$):** δ = -65.86 (d, *J* = 5.7 Hz), -114.22 – -114.33 (m). **^{13}C NMR (100 MHz, $CDCl_3$):** δ = 160.2 (d, *J* = 249.6 Hz), 133.2 (q, *J* = 31.9 Hz), 130.8 (d, *J* = 8.3 Hz), 130.7, 124.66 – 124.38 (m), 124.1 (d, *J* = 3.7 Hz), 121.7 (d, *J* = 14.8 Hz), 121.5, 116.2 (d, *J* = 22.4 Hz). **HRMS (ESI, m/z):** calcd for $C_9H_6F_4Na$ $[M+Na]^+$: 213.0303, found: 213.0295.

Structure of ligands

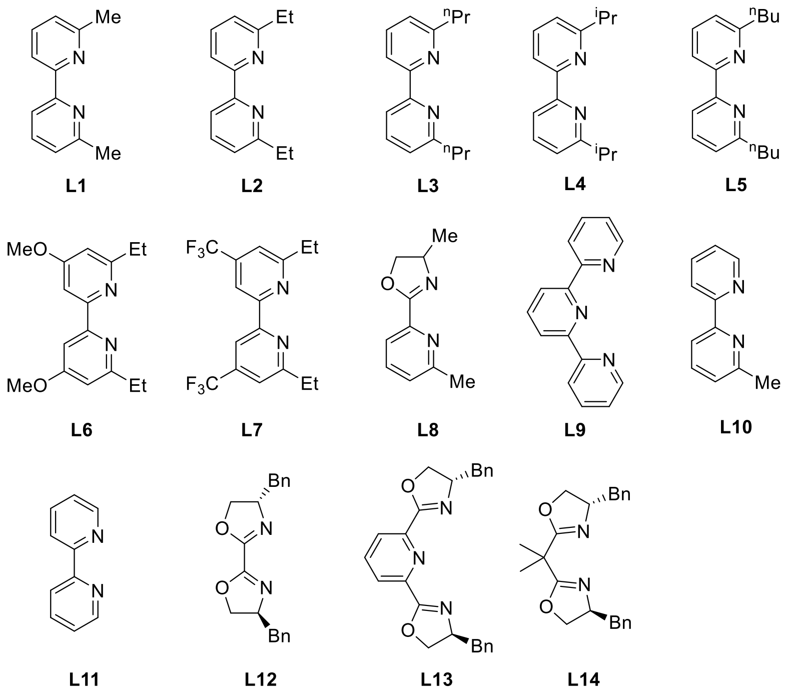

L1, **L9**-**L14** were purchased from the commercial supplier and used as received. **L8** was prepared according to the literature method.[13]

General procedure for the preparation of L2-L7

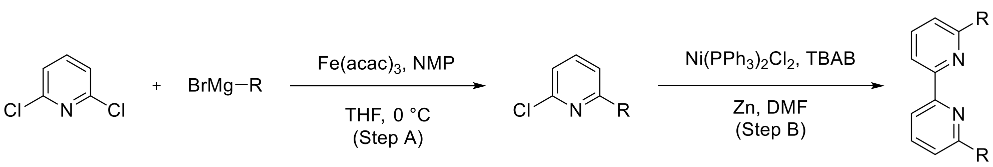

Step A. To an oven-dried 150 mL round-bottom flask equipped with magnetic stirring bar was charged 2,6-dichloropyridine (0.7400 g, 5 mmol, 1.0 equiv), ferric acetylacetonate (0.0883g, 0.25 mmol, 0.05 equiv). The flask was sealed, evacuated and refilled with nitrogen (3 times). Anhydrous THF (20 mL) and NMP (4.3 mL, 45 mmol, 9 equiv) was added via syringe and the resulting solution was cooled to 0 °C and added corresponding Grignard reagent (7.5 mL, 1M in THF, 1.5 equiv) slowly. After stirring at 0 °C for 1 hour, the mixture was allowed to stir at room temperature for additional 2 hours. The reaction was quenched by adding saturated NH_4Cl solution (5 mL). Then the resulting suspension was diluted

with H_2O (15 mL) followed by extraction with CH_2Cl_2 (3 × 10 mL). The combined organic layers were dried over anhydrous Na_2SO_4, filtered and evaporated to dryness under reduced pressure. The crude residue was purified by column chromatography on silica gel to afford corresponding compound.

Step B. To an oven-dried 50 mL round-bottom flask equipped with magnetic stirring bar was charged $Ni(PPh_3)_2Cl_2$ (0.2 mmol, 0.2 equiv), TBAB (0.3 mmol, 0.3 equiv), Zn (1.0 mmol, 1.0 equiv), the flask was sealed, evacuated and refilled with nitrogen (3 times). Anhydrous DMF (5 mL) and the product of Step A (1 mmol, 1.0 equiv) was added via syringe, the flask was stirred at 55 ^oC for 24 hours. The resulting suspension was diluted with H_2O (10 mL) followed by extraction with ethyl acetate (3 × 10 mL) and H_2O (3 × 10 mL). The combined organic layers were dried over anhydrous Na_2SO_4, filtered and evaporated to dryness under reduced pressure. The crude residue was purified by column chromatography on silica gel to afford corresponding compound.

2-chloro-6-ethylpyridine S15

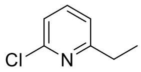

S15

Following the procedure of step A, **S15** (0.56g, 4.0 mmol) was obtained as colorless liquid in 79% yield by column chromatography on silica gel (petroleum ether/ethyl acetate = 100:1).

^{1}H NMR (400 MHz, $CDCl_3$): δ = 7.55 (t, *J* = 7.7 Hz, 1H), 7.14 (d, *J* = 7.9 Hz, 1H), 7.08 (d, *J* = 7.6 Hz, 1H), 2.79 (q, *J* = 7.6 Hz, 2H), 1.29 (t, *J* = 7.6 Hz, 3H). **^{13}C NMR (100 MHz, $CDCl_3$):** δ = 164.8, 150.9, 139.1, 121.4, 120.5, 31.1, 13.9. **HRMS (ESI, m/z):** calcd for C_7H_9ClN $[M+H]^+$: 142.0424, found: 142.0420.

6,6'-diethyl-2,2'-bipyridine L2

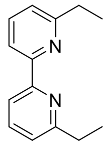

L2

Following the procedure of step B, **L2** (89.0 mg, 0.42 mmol) was obtained as colorless liquid in 42% yield by column chromatography on silica gel (petroleum ether/ethyl acetate = 50:1). **^{1}H NMR (400 MHz, $CDCl_3$):** δ = 8.24 (d, *J* = 7.8 Hz, 2H), 7.69 (t, *J* = 7.7 Hz, 2H), 7.14 (d, *J* = 7.6 Hz, 2H), 2.89 (q, *J* = 7.6 Hz, 4H), 1.36 (t, *J* = 7.6 Hz, 6H). **^{13}C NMR (100 MHz, $CDCl_3$):** δ = 162.9, 156.0, 137.2, 121.9, 118.4, 31.6, 14.0. **HRMS (ESI, m/z):** calcd for $C_{14}H_{17}N_2$ $[M+H]^+$: 213.1392, found: 213.1393.

2-chloro-6-propylpyridine S16

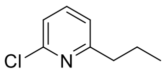

S16

Following the procedure of step A, **S16** (0.42 g, 2.7 mmol) was obtained as colorless liquid in 54% yield by column chromatography on silica gel (petroleum ether/ethyl acetate = 100:1).

^{1}H NMR (400 MHz, CDCl$_3$): δ = 7.53 (t, *J* = 7.7 Hz, 1H), 7.13 (d, *J* = 7.8 Hz, 1H), 7.05 (d, *J* = 7.5 Hz, 1H), 2.72 (t, *J* = 7.7 Hz, 2H), 1.78 – 1.68 (m, 2H), 0.95 (t, *J* = 7.4 Hz, 3H). **^{13}C NMR (100 MHz, CDCl$_3$):** δ = 163.6, 150.8, 138.9, 121. 5, 121.3, 40.1, 23.1, 13.9. **HRMS (ESI, m/z):** calcd for $C_8H_{11}ClN$ $[M+H]^+$: 156.0580, found: 156.0575.

6,6'-dipropyl-2,2'-bipyridine L3

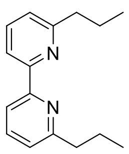

L3

Following the procedure of step B, **L3** (33.6 mg, 0.14 mmol) was obtained as colorless liquid in 14% yield by column chromatography on silica gel (petroleum ether/ethyl acetate = 50:1).

^{1}H NMR (400 MHz, CDCl$_3$): δ = 8.23 (dd, *J* = 7.8, 1.1 Hz, 2H), 7.69 (t, *J* = 7.7 Hz, 2H), 7.13 (dd, *J* = 7.6 Hz, 1.1 Hz, 2H), 2.83 (t, J = 7.7 Hz, 4H), 1.89 – 1.77 (m, 4H), 1.00 (t, *J* = 7.4 Hz, 6H). **^{13}C NMR (100 MHz, CDCl$_3$):** δ = 161.7, 156.1, 137.0, 122.6, 118.4, 40.5, 23.1, 14.0. **HRMS (ESI, m/z):** calcd for $C_{16}H_{21}N_2$ $[M+H]^+$: 241.1705, found: 241.1706.

2-chloro-6-isopropylpyridine S17

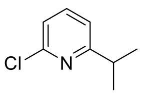

S17

Following the procedure of step A, **S17** (0.35 g, 2.3 mmol) was obtained as colorless liquid in 45% yield by column chromatography on silica gel (petroleum ether/ethyl acetate = 100:1).

^{1}H NMR (400 MHz, CDCl$_3$): δ = 7.55 (t, *J* = 7.7 Hz, 1H), 7.12 (d, *J* = 7.8 Hz, 1H), 7.08 (d, *J* = 7.6 Hz, 1H), 3.08 - 2.97 (m, 1H), 1.27 (d, *J* = 6.9 Hz, 6H). **^{13}C NMR (100 MHz, CDCl$_3$):** δ = 168.7, 150.7, 139.1, 121.6, 119.0, 36.2, 22.5. **HRMS (ESI, m/z):** calcd for $C_8H_{11}ClN$ $[M+H]^+$: 156.0580, found: 156.0576.

6,6'-diisopropyl-2,2'-bipyridine L4

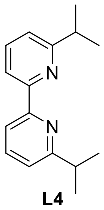

L4

Following the procedure of step B, **L4** (48.0 mg, 0.2 mmol) was obtained as colorless solid in 20% yield by column chromatography on silica gel (petroleum ether/ethyl acetate = 50:1). **^{1}H NMR (400 MHz, CDCl$_3$):** δ = 8.29 (dd, *J* = 7.8, 0.9 Hz, 2H), 7.71 (t, *J* = 7.7 Hz, 2H), 7.16 (dd, *J* = 7.7, 0.8 Hz, 2H), 3.18 – 3.07 (m, 2H), 1.36 (d, *J* = 6.9 Hz, 12H). **^{13}C NMR (100 MHz, CDCl$_3$):** δ = 166.6, 155.9, 137.1, 120.5, 118.4, 36.5, 22.8. **HRMS (ESI, m/z):** calcd for $C_{16}H_{21}N_2$ [M+H]$^+$: 241.1705, found: 241.1704.

2-butyl-6-chloropyridine S18

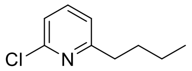

S18

Following the procedure of step A, **S18** (0.39 g, 2.3 mmol) was obtained as colorless liquid in 45% yield by column chromatography on silica gel (petroleum ether/ethyl acetate = 100:1).

^{1}H NMR (400 MHz, CDCl$_3$): δ = 7.51 (t, *J* = 7.7 Hz, 1H), 7.09 (d, *J* = 7.9 Hz, 1H), 7.02 (d, *J* = 7.6 Hz, 1H), 2.72 (t, J = 7.8 Hz, 2H), 1.72 – 1.58 (m, 2H), 1.38 – 1.29 (m, 2H), 0.89 (t, *J* = 7.4 Hz, 3H). **^{13}C NMR (100 MHz, CDCl$_3$):** δ = 163.8, 150.7, 138.9, 121.4, 121.2, 37.9, 32.0, 22.5, 14.0. **HRMS (ESI, m/z):** calcd for $C_9H_{13}ClN$ [M+H]$^+$: 170.0737, found: 170.0738.

6,6'-dibutyl-2,2'-bipyridine L5

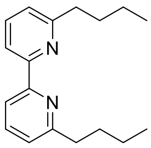

L5

Following the procedure of step B, **L5** (53.6 mg, 0.2 mmol) was obtained as colorless liquid in 20% yield by column chromatography on silica gel (petroleum ether/ethyl acetate = 50:1).

^{1}H NMR (400 MHz, CDCl$_3$): δ = 8.24 (d, *J* = 7.8 Hz, 2H), 7.69 (t, *J* = 7.7 Hz, 2H), 7.13 (d, *J* = 7.6 Hz, 2H), 2.86 (t, *J* = 7.7 Hz, 4H), 1.83 – 1.75 (m, 4H), 1.48 – 1.38 (m, 4H), 0.97 (t, *J* = 7.4 Hz, 6H). **^{13}C NMR (100 MHz, CDCl$_3$):** δ = 161.9, 156.1, 137.0, 122.5, 118.4, 38.3, 32.0, 22.6, 14.1. **HRMS (ESI, m/z):** calcd for $C_{18}H_{25}N_2$ [M+H]$^+$: 269.2018, found: 269.2020.

Preparation of compound S19

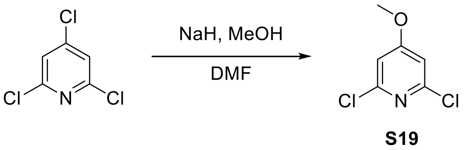

S19

To an oven-dried 50 mL round-bottom flask equipped with magnetic stirring bar was charged 2,4,6-trichloropyridine (1.82 g, 10 mmol, 1.0 equiv) and the flask was sealed, evacuated and refilled with nitro (3 times). Anhydrous DMF (10 mL) was added via syringe and the resulting solution was cooled to 0 °C. To the suspension, a solution of NaH (0.42g, 2.1 mmol, 1.05 equiv) in MeOH (0.34g, 10.5 mmol, 1.05 equiv) was added dropwise under nitrogen. The resulting mixture was stirred at room temperature for 16 hours, then the mixture was diluted with H_2O (15 mL), the organic phase was extracted with ethyl acetate (3 × 10 mL), the combined organic layers were washed with water, dried over Na_2SO_4, filtered and evaporated to dryness under reduced pressure. The crude residue was purified by column chromatography on silica gel (petroleum ether/ethyl acetate = 50:1) to afford **S19** (1.07 g, 6.1 mmol) as white solid in 61% yield. **^{1}H NMR (400 MHz, $CDCl_3$):** δ = 6.79 (s, 2H), 3.87 (s, 3H). **^{13}C NMR (100 MHz, $CDCl_3$):** δ = 168.6, 151.4, 109.3, 56.2. **HRMS (ESI, m/z):** calcd for $C_6H_6Cl_2NO$ $[M+H]^+$: 177.9826, found: 177.9825.

2-chloro-6-ethyl-4-methoxypyridine S20

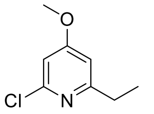

S20

Following the procedure of step A, **S20** (0.75 g, 4.4 mmol) was obtained as colorless liquid in 87% yield by column chromatography on silica gel (petroleum ether/ethyl acetate = 20:1).

^{1}H NMR (400 MHz, $CDCl_3$): δ = 6.63 (d, J = 2.1 Hz, 1H), 6.57 (d, J = 2.1 Hz, 1H), 3.80 (s, 3H), 2.69 (q, J = 7.6 Hz, 2H), 1.23 (t, J = 7.6 Hz, 2H). **^{13}C NMR (100 MHz, $CDCl_3$):** δ = 167.7, 165.5, 151.7, 107.6, 106.7, 55.6, 31.3, 13.8. **HRMS (ESI, m/z):** calcd for $C_8H_{11}ClNO$ $[M+H]^+$: 172.0529, found: 172.0529.

6,6'-diethyl-4,4'-dimethoxy-2,2'-bipyridine L6

Following the procedure of step B, **L6** (24.5 mg, 0.09 mmol) was obtained as yellow solid in 9% yield by column chromatography on silica gel (petroleum ether/ethyl acetate = 10:1).

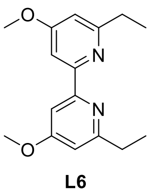

L6

^{1}H NMR (400 MHz, $CDCl_3$): δ = 7.81 (d, J = 2.3 Hz, 2H), 6.69 (d, J = 2.4 Hz, 2H), 3.93 (s, 6H), 2.84 (q, J = 7.6 Hz, 4H), 1.34 (t, J = 7.6 Hz, 6H). **^{13}C NMR (100 MHz, $CDCl_3$):** δ = 167.1, 164.5, 157.7, 108.5, 104.2, 55.3, 31.5, 13.9. **HRMS (ESI, m/z):** calcd for $C_{16}H_{21}N_2O_2$ $[M+H]^+$: 273.1603, found: 273.1606.

2-chloro-6-ethyl-4-(trifluoromethyl)pyridine S21

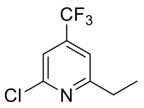
S21

Following the procedure of step A, **S21** (0.69 g, 3.3 mmol) was prepared from 2,6-dichloro-4-(trifluoromethyl)pyridine, obtained as yellow liquid in 65% yield by column chromatography on silica gel (petroleum ether/ethyl acetate = 100:1).

^{1}H NMR (400 MHz, $CDCl_3$): δ = 7.38 (s, 1H), 7.30 (s, 1H), 2.89 (q, *J* = 7.6 Hz, 2H), 1.33 (t, *J* = 7.6 Hz, 3H). **^{19}F NMR (376 MHz, $CDCl_3$):** δ = -64.59. **^{13}C NMR (100 MHz, $CDCl_3$):** δ = 166.4, 151.7, 141.3 (q, *J* = 34.1 Hz), 122.2 (q, *J* = 273.6 Hz), 117.7 (q, *J* = 3.8 Hz), 116.5 (q, *J* = 3.4 Hz), 31.3, 13.5. **HRMS (ESI, m/z):** calcd for $C_8H_8ClF_3N$ $[M+H]^+$: 210.0297, found: 210.0298.

6,6'-diethyl-4,4'-bis(trifluoromethyl)-2,2'-bipyridine L7

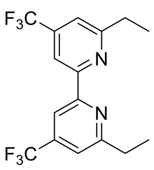
L7

Following the procedure of step B, **L7** (187.9 mg, 0.54 mmol) was obtained as yellow solid in 54% yield by column chromatography on silica gel (petroleum ether/ethyl acetate = 100:1).

^{1}H NMR (400 MHz, $CDCl_3$): δ = 8.54 (s, 2H), 7.41 (s, 2H), 2.99 (q, *J* = 7.6 Hz, 4H), 1.41 (t, *J* = 7.6 Hz, 6H). **^{19}F NMR (376 MHz, $CDCl_3$):** δ = -64.55. **^{13}C NMR (100 MHz, $CDCl_3$):** δ = 164.7, 155.9, 139.7 (q, *J* = 33.6 Hz), 123.2 (q, *J* = 273.4 Hz), 118.2 (q, *J* = 3.2 Hz), 114.4 (q, *J* = 3.4 Hz), 31.5, 13.7. **HRMS (ESI, m/z):** calcd for $C_{16}H_{15}F_6N_2$ $[M+H]^+$: 349.1139, found: 349.1141.

Optimization of reaction conditions

To an oven-dried Schlenk tube equipped with a magnetic stir bar was added Ni salt (0.01 mmol, 5.0 mol%), ligand (0.012 mmol, 6.0 mol%), Mn powder (16.5 mg, 0.3 mmol, 1.5 equiv). After the tube was evacuated and refilled with nitrogen (3 times), solvent (1.0 mL), compound **1a** (106.2 mg, 0.6 mmol, 3.0 equiv), compound **2a** (46.0 mg, 0.2 mmol, 1.0 equiv) was added under nitrogen atmosphere. The tube was capped and stirred at 25 ℃ for 12 h. The reaction mixture was diluted with H_2O (5 mL) and extracted with ethyl acetate (3 × 5 mL). The combined organic layers were washed with water (10 mL), brine (10 mL), dried over anhydrous Na_2SO_4, filtered and evaporated under reduced pressure. The crude residue was analyzed by ^{19}F NMR with 1-iodo-4-(trifluoromethyl)benzene as internal standard. The pure product was then isolated by column chromatography or preparative TLC on silica gel to afford the desired product.

Representative example of assay yield determination by ^{19}F NMR

To the crude products was added 1-iodo-4-(trifluoromethyl)benzene (36.3mg, 0.133 mmol) as internal standard. The resulting mixture was analyzed by ^{19}F NMR and the assay yield was calculated by comparing the integration of signals of the standard (s, 63.0 ppm) and the desired products (d, 87.5 ppm, d, 90.3 ppm). For example, the assay yield of the reaction shown in entry 1 of Table S1 was calculated by the equation “assay yield = integration (3a)/integration (standard)×100%” as 64%.

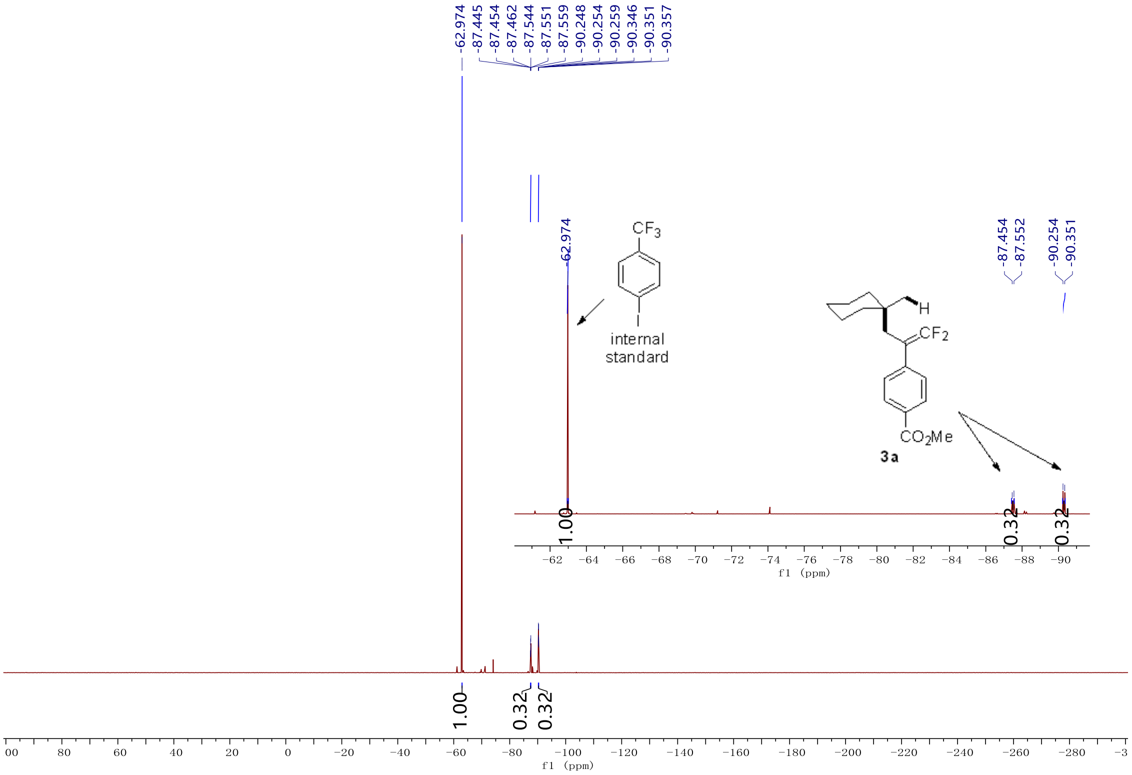

Supplementary Figure 1. Crude ^{19}F NMR spectrum of the reaction shown in entry 1 of Table S1

Supplementary Table 1. Screening of Screening of Ligands.[a]

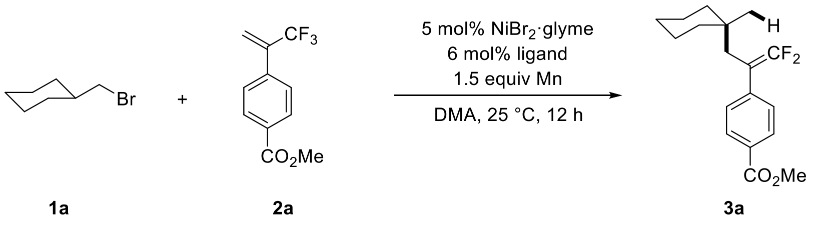

Entry	Ligand	AY/%[b]	rr[c]
1	L1	64	45:1
2	L2	72(67)[d]	41:1
3	L3	70(62)[d]	38:1
4	L4	54	45:1

5	L5	62	25:1
6	L6	62	6:1
7	L7	n.r.	-
8	L8	n.r.	-
9	L9	n.r.	-
10	L10	n.r.	-
11	L11	n.r.	-
12	L12	n.r.	-
13	L13	n.r.	-
14	L14	n.r.	-

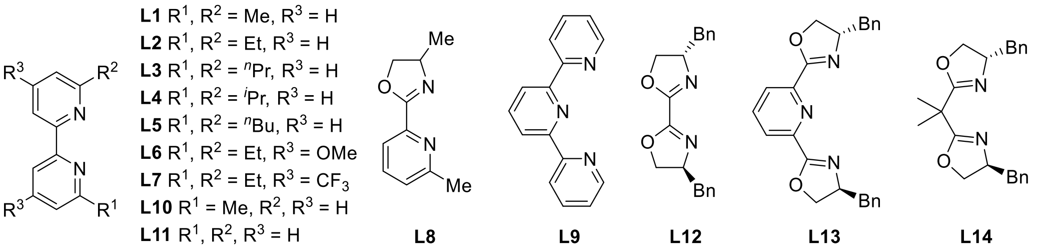

[a] The reaction was carried out with **1a** (0.6 mmol), **2a** (0.2 mmol), $NiBr_2$ glyme (0.01 mmol), ligand (0.012 mmol), Mn powder (0.3 mmol) in DMA (1.0 mL) under nitrogen atmosphere at 25 ℃ for 12 h. [b] Assay yields were determined by the ^{19}F NMR of the crude products with 1-iodo-4-(trifluoromethyl)benzene as internal standard (0.133 mmol). [c] Rr refers to the ratio of desired product to the sum of all the other regioisomers, which was determined by the ^{19}F NMR analysis of the crude products. [d] Isolated yield was indicated in the parentheses.

Supplementary Table 2. Screening of Solvents.[a]

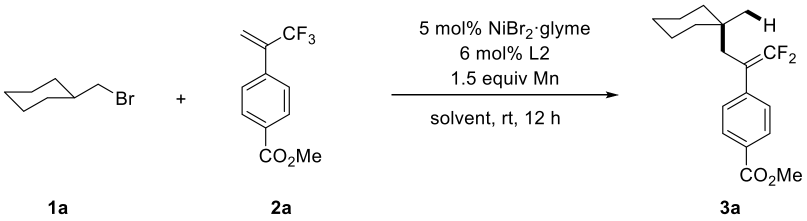

Entry	Solvent	Additive	AY/%[b]	rr[c]
1	DMA	-	72(67)[d]	41:1
2	NMP	-	68	15:1
3	DMSO	-	44	1:1
4	THF	-	80(84)[d]	51:1
5	1,4-Dioxane	-	n.r.	-

[a] The reaction was carried out with **1a** (0.6 mmol), **2a** (0.2 mmol), $NiBr_2$ glyme (0.01 mmol), L2 (0.012 mmol), Mn powder (0.3 mmol) in solvent (1.0 mL) under nitrogen atmosphere at 25 ℃ for 12 h. [b] Assay yields were determined by the ^{19}F NMR of the crude products with 1-iodo-4-(trifluoromethyl)benzene as internal standard (0.133 mmol). [c] Rr refers to the ratio of desired product to the sum of all the other regioisomers, which was determined by the ^{19}F NMR analysis of the crude products. [d] Isolated yield was indicated in the parentheses.

Supplementary Table 3. Screening of Ni salts.[a]

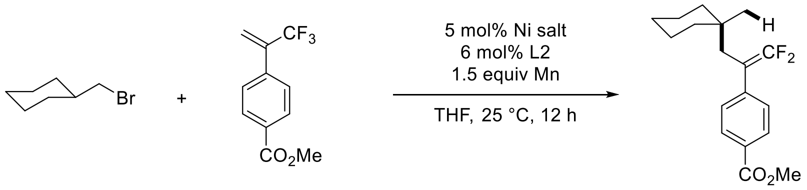

1a **2a** **3a**

Entry	Ni salt	AY/%[b]	rr[c]
1	$Ni(ClO_4)_2$ $6H_2O$	n.r.	/
2	$NiCl_2$ glyme	58	60:1
3	$NiBr_2$ glyme	80(84)[d]	51:1
4	$Ni(OTf)_2$	n.r.	/
5	$Ni(OAc)_2$ $4H_2O$	n.r.	/
6	$NiBr_2$	n.r.	/
7	NiI_2 $6H_2O$	n.r.	/

[a] The reaction was carried out with **1a** (0.6 mmol), **2a** (0.2 mmol), Ni salt (0.01 mmol), L2 (0.012 mmol), Mn powder (0.3 mmol) in THF (1.0 mL) under nitrogen atmosphere at 25 ℃ for 12 h. [b] Assay yields were determined by the ^{19}F NMR of the crude products with 1-iodo-4-(trifluoromethyl)benzene as internal standard (0.133 mmol). [c] Rr refers to the ratio of desired product to the sum of all the other regioisomers, which was determined by the ^{19}F NMR analysis of the crude products. [d] Isolated yield was indicated in the parentheses.

Supplementary Table 4. Screening of reductants.

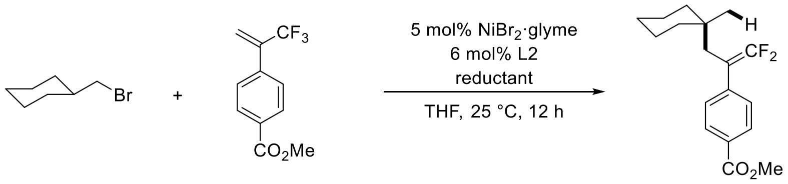

1a **2a** **3a**

Entry	Reductant	AY/%[b]	rr[c]
1	Mn powder (1.5 equiv)	80(84)[d]	51:1
2	Zn powder (1.5 equiv)	n.d.p.	/

3	Zn powder (1.5 equiv)<br>$MgCl_2$ (1.0 equiv)	n.d.p.	/
4	HCOONa (1.5 equiv)	n.r.	/
5	B_2pin_2 (1.5 equiv)<br>K_3PO_4 (1.5 equiv)	8%	3:1
6	DEMS (1.5 equiv)<br>Na_2CO_3(1.5 equiv)	6%	2:1

[a] The reaction was carried out with **1a** (0.6 mmol), **2a** (0.2 mmol), $NiBr_2$ glyme (0.01 mmol), L2 (0.012 mmol), Mn powder (0.3 mmol) in THF (1.0 mL) under nitrogen atmosphere at 25 ℃ for 12 h. [b] Assay yields were determined by the ^{19}F NMR of the crude products with 1-iodo-4-(trifluoromethyl)benzene as internal standard (0.133 mmol). [c] Rr refers to the ratio of desired product to the sum of all the other regioisomers, which was determined by the ^{19}F NMR analysis of the crude products. [d] Isolated yield was indicated in the parentheses. DEMS, diethxoylmethylsilane.

General procedure for the difluoroallylation reaction

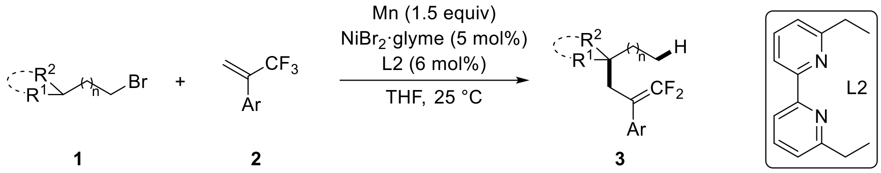

To an oven-dried 10 mL Schlenk tube (see below) equipped with a magnetic stirring bar was added $NiBr_2$·glyme (3.1 mg, 0.01 mmol, 5.0 mol%), L2 (2.5 mg, 0.012 mmol, 6.0 mol%), Mn powder (16.5 mg, 0.3 mmol, 1.5 equiv). After tube was evacuated and refilled with nitrogen (3 times), THF (1.0 mL), compound **1** (0.6 mmol, 3.0 equiv), and compound **2** (0.2 mmol, 1.0 equiv) were added under nitrogen atmosphere. The tube was capped and stirred at 25 ℃ for 12 h to 24 h. The reaction mixture was diluted with H_2O (5 mL) and extracted with ethyl acetate (3 × 5 mL). The combined organic layers were washed with water (10 mL), brine (10 mL), dried over anhydrous Na_2SO_4, filtered and evaporated under reduced pressure. The crude residue was analyzed by ^{19}F NMR with 1-iodo-4-(trifluoromethyl)benzene as internal standard. The pure product was isolated by column chromatography or preparative TLC on silica gel. In the cases of **3v**, **3w** and **3x,** DMA was used as solvent instead of THF.

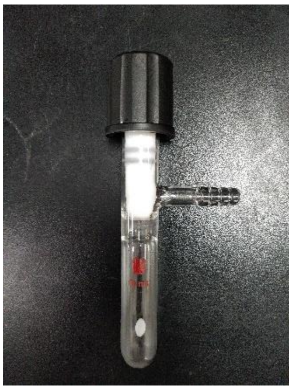

methyl 4-(1,1-difluoro-3-(1-methylcyclohexyl)prop-1-en-2-yl)benzoate 3a

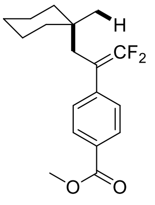

3a

Following general procedure, **3a** (51.7 mg, 0.168 mmol) was obtained as colorless liquid in 84% yield by preparative TLC on silica gel (petroleum ether/ethyl acetate = 50 : 1). **^{1}H NMR (400 MHz, $CDCl_3$):** δ = 8.01 – 7.97 (m, 2H), 7.41 – 7.37 (m, 2H), 3.91 (s, 3H), 2.37 (t, *J* = 2.40 Hz, 2H), 1.43 – 1.09 (m, 10H), 0.74 (s, 3H). **^{19}F NMR (376 MHz, $CDCl_3$):** δ = -87.39 (d, *J* = 36.8 Hz), -90.19 (d, *J* = 36.7 Hz). **^{13}C NMR (100 MHz, $CDCl_3$):** δ = 166.9, 154.5 (dd, *J* = 291.5, 289.0 Hz), 141.0 (dd, *J* = 4.6, 2.9 Hz), 129.6, 128.7, 128.6 (t, *J* = 2.5 Hz), 90.4 (dd, *J* = 22.4, 12.5 Hz), 52.2, 40.2, 38.0, 35.3, 26.3, 24.7, 22.0. **HRMS (ESI, m/z):** calcd for $C_{18}H_{23}F_2O_2$ $[M+H]^+$: 309.1666, found: 309.1671.

methyl 4-(1,1-difluoro-3-(4-methyltetrahydro-2H-pyran-4-yl)prop-1-en-2-yl)benzoate 3b

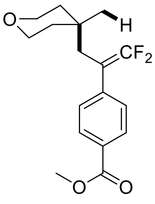

3b

Following general procedure, **3b** (52.8 mg, 0.170 mmol) was obtained as colorless liquid in 85% yield by preparative TLC on silica gel (petroleum ether/ethyl acetate = 20 : 1).

^{1}H NMR (400 MHz, $CDCl_3$): δ = 8.02 – 8.00 (m, 2H), 7.41 – 7.38 (m, 2H), 3.91 (s, 3H), 3.64 – 3.59 (m, 2H), 3.54 – 3.47 (m, 2H), 2.45 (t, *J* = 2.4 Hz, 2H), 1.43 – 1.36 (m, 2H), 1.18 – 1.12 (m, 2H), 0.87 (s, 3H). **^{19}F NMR (376 MHz, $CDCl_3$):** δ = -86.90 (d, *J* = 35.5 Hz), -89.53 (d, *J* = 35.6 Hz). **^{13}C NMR (100 MHz, $CDCl_3$):** δ = 166.8, 154.7 (dd, *J* = 292.7, 289.2 Hz), 140.4 (dd, *J* = 4.8, 3.1 Hz), 129.8, 129.0, 128.5 (t, *J* = 2.5 Hz), 89.8 (dd, *J* = 22.1, 13.1 Hz), 63.8, 52.2, 40.3, 37.7, 33.1, 23.6. **HRMS (ESI, m/z):** calcd for $C_{17}H_{21}F_2O_3$ $[M+H]^+$: 311.1459, found: 311.1458.

tert-butyl 4-(3,3-difluoro-2-(4-(methoxycarbonyl)phenyl)allyl)-4-methylpiperidine-1-carboxylate 3c

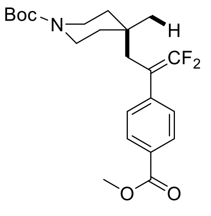

3c

Following general procedure, **3c** (53.2 mg, 0.130 mmol) was obtained as colorless liquid in 65% yield by preparative TLC on silica gel (petroleum ether/ethyl acetate = 10 : 1).

^{1}H NMR (400 MHz, $CDCl_3$): δ = 8.01 (d, *J* = 8.4 Hz, 2H), 7.38 (d, *J* = 7.6 Hz, 2H), 3.92 (s, 3H), 3.53 (s, 2H), 3.06 – 2.99 (m, 2H), 2.42 (s, 2H), 1.42 (s, 9H), 1.26 – 1.14 (m, 4H), 0.82 (s, 3H). **^{19}F NMR (376 MHz, $CDCl_3$):** δ = -86.84 (d, *J* = 35.4Hz), -89.42 (d, *J* = 35.5Hz). **^{13}C NMR (100 MHz, $CDCl_3$):** δ = 166.8, 154.9, 154.7 (dd, *J* = 291.9, 288.8 Hz), 140.4 (dd, *J* = 4.6, 3.0 Hz), 129.8, 129.0, 128.5, 89.8 (dd, *J* = 22.1, 13.2 Hz), 79.4, 52.3, 39.8, 36.9, 34.0, 28.5 (overlapped), 23.1. **HRMS (ESI, m/z):** calcd for $C_{22}H_{29}F_2NNaO_4$ $[M+Na]^+$: 432.1962, found: 432.1959.

methyl 4-(1,1-difluoro-3-((2r,5r)-2-(4-methoxyphenyl)-5-methyl-1,3-dioxan-5-yl)prop-1-en-2-yl)benzoate 3d

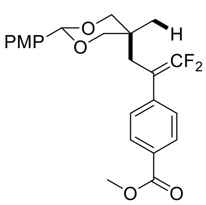

3d

Following general procedure, **3d** (27.6 mg, 0.066 mmol) was obtained as colorless liquid in 33% yield by preparative TLC on silica gel (petroleum ether/ethyl acetate = 10 : 1).

^{1}H NMR (400 MHz, $CDCl_3$): δ = 8.07 – 7.99 (m, 2H), 7.50 – 7.48 (m, 2H), 7.42 – 7.34 (m, 2H), 6.95 – 6.87 (m, 2H), 5.31 (s, 1H), 3.91 (s, 3H), 3.82 (s, 3H), 3.72 (d, *J* = 11.1 , 2H), 3.50 (d, *J* = 11.2 Hz, 2H), 2.96 (s, 2H), 0.60 (s, 3H). **^{19}F NMR (376 MHz, $CDCl_3$):** δ = -86.25 (d, *J* = 33.3 Hz), -88.47 (d, *J* = 33.3 Hz).**^{13}C NMR (100 MHz, $CDCl_3$):** δ = 166.8, 160.2, 157.9 (dd, *J* = 293.0, 289.6 Hz), 139.6 (dd, *J* = 4.2 Hz, 3.3 Hz), 130.8, 129.8, 129.1, 128.5 (t, *J* = 2.7 Hz), 127.5, 113.8, 101.9, 89.9 (dd, *J* = 22.0, 13.1 Hz), 75.8, 55.4, 52.2, 34.5, 31.9, 19.1. **HRMS (ESI, m/z):** calcd for $C_{23}H_{25}F_2O_5$ $[M+H]^+$: 419.1670, found: 419.1669.

methyl 4-(1,1-difluoro-3-(1-methylcyclopentyl)prop-1-en-2-yl)benzoate 3e

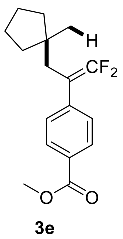

3e

Following general procedure, **3e** (38.3 mg, 0.130 mmol) was obtained as colorless liquid in 65% yield by preparative TLC on silica gel (petroleum ether/ethyl acetate = 50 : 1).

^{1}H NMR (400 MHz, $CDCl_3$): δ = 8.00 (d, *J* = 8.3 Hz, 2H), 7.39 (d, *J* = 8.1 Hz, 2H), 3.91 (s, 3H), 2.47 (t, *J* = 2.4 Hz, 2H), 1.58 – 1.49 (m, 4H), 1.29 – 1.10 (m, 4H), 0.81 (s, 3H). **^{19}F NMR (376 MHz, $CDCl_3$):** δ = -88.54 (d, *J* = 37.8 Hz), -90.68 (d, *J* = 37.9 Hz). **^{13}C NMR**

(100 MHz, $CDCl_3$): δ = 166.9, 154.6 (dd, *J* = 291.3, 288.4 Hz), 140.6 (dd, *J* = 4.7, 2.9 Hz), 129.6, 128.8, 128.6 (t, *J* = 2.5 Hz), 91.3 (dd, *J* = 22.2, 12.5 Hz), 52.2, 44.0, 39.4, 39.0, 25.8, 23.8. **HRMS (ESI, m/z):** calcd for $C_{17}H_{21}F_2O_2$ $[M+H]^+$: 295.1510, found: 295.1517.

methyl 4-(1,1-difluoro-3-(1-methylcycloheptyl)prop-1-en-2-yl)benzoate 3f

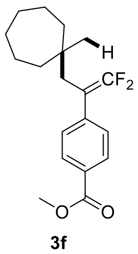

3f

Following general procedure, **3f** (32.8 mg, 0.102 mmol) was obtained as colorless liquid in 51% yield by preparative TLC on silica gel (petroleum ether/ethyl acetate = 50 : 1). **^{1}H NMR (400 MHz, $CDCl_3$):** δ = 8.01 – 7.98 (m, 2H), 7.40 – 7.37 (m, 2H), 3.91 (s, 3H), 2.36 (t, *J* = 2.4 Hz, 2H), 1.46 – 1.41 (m, 4H), 1.34 – 1.20 (m, 8H), 0.67 (s, 3H). **^{19}F NMR (376 MHz, $CDCl_3$):** δ = -87.67 (d, *J* = 36.5 Hz), -90.15 (d, *J* = 36.5 Hz). **^{13}C NMR (100 MHz, $CDCl_3$):** δ = 166.9, 154.7 (dd, *J* = 291.7, 288.7 Hz), 140.9 (dd, *J* = 4.7, 3.1 Hz), 129.6, 128.7, 128.5 (t, *J* = 2.5 Hz), 90.8 (dd, *J* = 22.2, 12.2 Hz), 52.2, 40.5, 40.0, 38.4, 30.9, 27.9, 22.8. **HRMS (ESI, m/z):** calcd for $C_{19}H_{25}F_2O_2$ $[M+H]^+$: 323.1823, found: 323.1821.

methyl 4-(1,1-difluoro-4,4-dimethylpent-1-en-2-yl)benzoate 3g

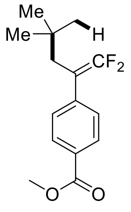

3g

Following general procedure, **3g** (34.8 mg, 0.130 mmol) was obtained as colorless liquid in 65% yield by preparative TLC on silica gel (petroleum ether/ethyl acetate = 50 : 1). **^{1}H NMR (400 MHz, $CDCl_3$):** δ = 8.01 – 7.99 (m, 2H), 7.41 – 7.38 (m, 2H), 3.91 (s, 3H), 2.36 (t, *J* = 2.4 Hz, 2H), 0.79 (s, 9H). **^{19}F NMR (376 MHz, $CDCl_3$):** δ = -87.63 (d, *J* = 36.2 Hz), -90.34 (d, *J* = 36.5 Hz). **^{13}C NMR (100 MHz, $CDCl_3$):** δ = 166.9, 154.6 (dd, *J* = 291.5, 289.4 Hz), 140.7 (dd, *J* = 4.7, 3.3 Hz), 129.7, 128.8, 128.5 (t, *J* = 2.7 Hz), 91.0 (dd, *J* = 22.2, 12.3 Hz), 52.2, 41.0, 32.9, 29.7. **HRMS (ESI, m/z):** calcd for $C_{15}H_{19}F_2O_2$ $[M+H]^+$: 269.1353, found: 269.1361.

methyl 4-(4-ethyl-1,1-difluoro-4-methyloct-1-en-2-yl)benzoate 3h

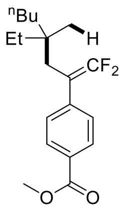

3h

Following general procedure, **3h** (41.5 mg, 0.128 mmol) was obtained as colorless liquid in 64% yield by preparative TLC on silica gel (petroleum ether/ethyl acetate = 50 : 1). **^{1}H NMR (400 MHz, $CDCl_3$):** δ = 8.01 – 7.98 (m, 2H), 7.40 – 7.36 (m, 2H), 3.91 (s, 3H), 2.35 (t, *J* = 2.4, 2H), 1.20 – 1.01 (m, 8H), 0.80 – 0.76 (m, 3H), 0.68 (t, *J* = 7.5 Hz, 3H), 0.66 (d, *J* = 0.8 Hz, 3H). **^{19}F NMR (376 MHz, $CDCl_3$):** δ = -87.83 (d, *J* = 37.2 Hz), -90.10 (d, *J* = 37.1 Hz). **^{13}C NMR (100 MHz, $CDCl_3$):** δ = 166.9, 154.4 (dd, *J* = 290.9, 289.2 Hz), 140.9 (dd, *J*

= 4.9, 2.6 Hz), 129.6, 128.7, 128.6 (t, J = 2.4 Hz), 90.7 (dd, J = 22.2, 12.8 Hz), 52.2, 38.3, 37.7, 37.1, 31.5, 25.7, 24.7, 23.5, 14.1, 8.0. **HRMS (ESI, m/z):** calcd for $C_{19}H_{27}F_2O_2$ $[M+H]^+$: 325.1979, found: 325.1977.

methyl 4-(1,1-difluoro-4-hexyl-4-methyldodec-1-en-2-yl)benzoate 3i

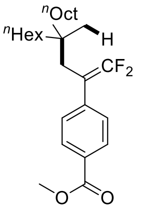

3i

Following general procedure, **3i** (55.0 mg, 0.126 mmol) was obtained as colorless liquid in 63% yield by preparative TLC on silica gel (petroleum ether/ethyl acetate = 50 : 1). **^{1}H NMR (400 MHz, CDCl$_3$):** δ = 8.01 - 7.98 (m, 2H), 7.39 – 7.36 (m, 2H), 3.91 (s, 3H), 2.34 (t, J = 2.3 Hz, 2H), 1.30 – 1.01 (m, 24H), 0.87 (t, J = 6.9 Hz, 3H), 0.84 (t, J = 7.0 Hz, 3H), 0.68 (s, 3H). **^{19}F NMR (376 MHz, CDCl$_3$):** δ = -87.91 (d, J = 37.3 Hz), -90.17 (d, J = 37.2 Hz). **^{13}C NMR (100 MHz, CDCl$_3$):** δ = 166.8, 154.4 (dd, J = 290.9, 289.7 Hz), 140.9 (dd, J = 5.0, 2.6 Hz), 129.6, 128.75, 128.69 (t, J = 2.2 Hz), 90.7 (dd, J = 22.2, 12.9 Hz), 52.2, 39.3, 37.7, 37.4, 31.96, 31.93, 30.5, 30.1, 29.7, 29.4, 25.2, 23.56, 23.55, 22.7, 14.2, 14.1. **HRMS (ESI, m/z):** calcd for $C_{27}H_{42}F_2NaO_2$ $[M+Na]^+$: 459.3051, found: 459.3045.

methyl 4-(6-(benzyloxy)-1,1-difluoro-4,4-dimethylhex-1-en-2-yl)benzoate 3j

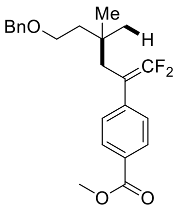

3j

Following general procedure, **3j** (52.0 mg, 0.134 mmol) was obtained as colorless liquid in 67% yield by preparative TLC on silica gel (petroleum ether/ethyl acetate = 20 : 1).

^{1}H NMR (400 MHz, CDCl$_3$): δ = 8.02 – 7.99 (m, 2H), 7.39 – 7.35 (m, 2H), 7.35 – 7.31 (m, 2H), 7.31 – 7.27 (m, 3H), 4.42 (s, 2H), 3.91 (s, 3H), 3.45 (t, J = 7.2 Hz, 2H), 2.41 (t, J = 2.4 Hz, 2H), 1.52 (t, J = 7.2 Hz, 2H), 0.77 (s, 6H). **^{19}F NMR (376 MHz, CDCl$_3$):** δ = -87.24 (d, J = 35.9 Hz), -89.77 (d, J = 35.9 Hz). **^{13}C NMR (100 MHz, CDCl$_3$):** δ = 166.8, 154.6 (dd, J = 292.8, 289.4 Hz), 140.6 (dd, J = 4.6, 3.0 Hz), 138.5, 129.7, 128.9, 128.54 (t, J = 2.5 Hz), 128.46, 127.7, 127.6, 90.5 (dd, J = 22.1, 12.7 Hz), 73.1, 67.2, 52.2, 41.5, 40.0, 34.8, 27.4. **HRMS (ESI, m/z):** calcd for $C_{23}H_{27}F_2O_3$ $[M+H]^+$: 389.1928, found: 389.1935.

methyl 4-(6-(benzoyloxy)-1,1-difluoro-4,4-dimethylhex-1-en-2-yl)benzoate 3k

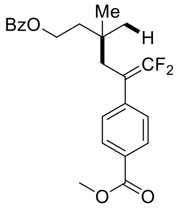

3k

Following general procedure, **3k** (59.6 mg, 0.148 mmol) was obtained as colorless liquid in 74% yield by preparative TLC on silica gel (petroleum ether/ethyl acetate = 20 : 1).

^{1}H NMR (400 MHz, $CDCl_3$): δ = 8.02 – 7.97 (m, 4H), 7.57 – 7.53 (m, 1H), 7.45 – 7.39 (m, 4H), 4.29 (t, *J* = 7.2 Hz, 2H), 3.89 (s, 3H), 2.47 (t, *J* = 2.3 Hz, 2H), 1.65 (t, *J* = 7.2 Hz, 2H), 0.86 (s, 6H). **^{19}F NMR (376 MHz, $CDCl_3$):** δ = -87.07 (d, *J* = 35.4 Hz), -89.50 (d, *J* = 35.4 Hz). **^{13}C NMR (100 MHz, $CDCl_3$):** δ = 166.74, 166.67, 154.7 (dd, *J* = 292.4, 289.2 Hz), 140.3 (dd, *J* = 4.7, 2.9 Hz), 133.0, 130.4, 129.8, 129.6, 129.0, 128.5 (t, *J* = 2.7 Hz), 128.4, 90.3 (dd, *J* = 22.1, 13.0 Hz), 62.0, 52.2, 40.2, 39.9, 34.9, 27.3. **HRMS (ESI, m/z):** calcd for $C_{23}H_{25}F_2O_4$ $[M+H]^+$: 403.1721, found: 403.1725.

methyl 4-(6-((tert-butyldiphenylsilyl)oxy)-1,1-difluoro-4,4-dimethylhex-1-en-2-yl)benzoate 3l

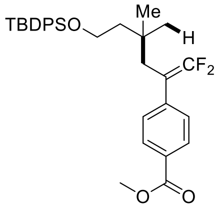

3l

Following general procedure, **3l** (77.2 mg, 0.144 mmol) was obtained as colorless liquid in 72% yield by preparative TLC on silica gel (petroleum ether/ethyl acetate = 20 : 1).

^{1}H NMR (400 MHz, $CDCl_3$): δ = 8.03 – 8.00(m, 2H), 7.68 – 7.65 (m, 4H), 7.46 – 7.34 (m, 8H), 3.93 (s, 3H), 3.67 (t, *J* = 7.1 Hz, 2H), 2.37 (s, 2H), 1.48 (t, *J* = 7.1 Hz, 2H), 1.04 (s, 9H), 0.72 (s, 6H).**^{19}F NMR (376 MHz, $CDCl_3$):** δ = -87.35 (d, *J* = 36.0 Hz), -89.90 (d, *J* = 36.0 Hz). **^{13}C NMR (100 MHz, $CDCl_3$):** δ = 166.8, 157.6 (dd, *J* = 292.0, 289.5 Hz), 140.7 (dd, *J* = 4.4, 3.0 Hz), 135.7, 134.0, 129.7, 128.8, 128.5, 127.7, 90.6 (dd, *J* = 22.1, 12.6 Hz), 60.8, 52.2, 44.7, 40.2, 34.8, 27.3, 26.9, 19.2. **HRMS (ESI, m/z):** calcd for $C_{32}H_{39}F_2O_3Si$ $[M+H]^+$: 537.2637, found: 537.2633.

methyl 4-(6-(1,3-dioxoisoindolin-2-yl)-1,1-difluoro-4,4-dimethylhex-1-en-2-yl)benzoate 3m

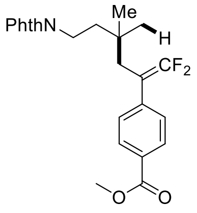

3m

Following general procedure, **3m** (44.4 mg, 0.104 mmol) was obtained as colorless liquid in 52% yield by preparative TLC on silica gel (petroleum ether/ethyl acetate = 6 : 1).

^{1}H NMR (400 MHz, $CDCl_3$): δ = 8.02 – 7.97 (m, 2H), 7.84 – 7.81 (m, 2H), 7.72 – 7.68 (m, 2H), 7.41 – 7.39 (m, 2H), 3.90 (s, 3H), 3.67 – 3.63 (m, 2H), 2.46 (t, *J* = 2.2 Hz, 2H), 1.57 – 1.53 (m, 2H), 0.82 (s, 3H). **^{19}F NMR (376 MHz, $CDCl_3$):** δ = -86.96 (d, *J* = 35.7 Hz), -89.48 (d, *J* = 35.5 Hz). **^{13}C NMR (100 MHz, $CDCl_3$):** δ = 168.3, 166.8, 154.6 (dd, *J* = 292.3, 289.0 Hz),

140.4 (dd, J = 4.7, 2.7 Hz), 134.0, 132.3, 129.8, 128.9, 128.5, 123.3, 90.3 (dd, *J* = 22.0, 13.1 Hz), 52.2, 40.5, 39.6, 34.9, 34.2, 26.8. **HRMS (ESI, m/z):** calcd for $C_{24}H_{23}F_2NNaO_4$ [M+Na]$^+$: 428.1673, found: 428.1676.

methyl 4-(4-cyclohexyl-1,1-difluoro-4-methylpent-1-en-2-yl)benzoate 3n

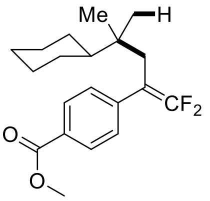

3n

Following general procedure, **3n** (48.4 mg, 0.144 mmol) was obtained as colorless liquid in 72% yield by preparative TLC on silica gel (petroleum ether/ethyl acetate = 50 : 1).

^{1}H NMR (400 MHz, CDCl$_3$): δ = 8.04 – 7.96 (m, 2H), 7.40 – 7.37 (m, 2H), 3.91 (s, 3H), 2.38 (t, *J* = 2.5 Hz, 2H), 1.73 – 1.61 (m, 4H), 1.62 – 1.54 (m, 1H), 1.05 – 0.85 (m, 6H), 0.67 (s, 6H). **^{19}F NMR (376 MHz, CDCl$_3$):** δ = -87.77 (d, *J* = 36.9 Hz), -89.99 (d, *J* = 36.9 Hz). **^{13}C NMR (100 MHz, CDCl$_3$):** δ = 166.9, 154.4 (dd, *J* = 291.8, 289.5 Hz), 141.0 (dd, *J* = 4.8, 2.7 Hz), 129.6, 128.7, 128.6 (t, *J* = 2.3 Hz), 90.9 (dd, *J* = 22.2, 12.5 Hz), 52.2, 47.1, 37.7, 37.1, 27.4, 27.1, 26.7, 24.9. **HRMS (ESI, m/z):** calcd for $C_{20}H_{27}F_2O_2$ [M+H]$^+$: 337.1979, found: 337.1976.

methyl 4-(1,1-difluoro-4,4-dimethylhex-1-en-2-yl)benzoate 3o

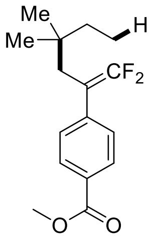

3o

Following general procedure, **3o** (26.5 mg, 0.094 mmol) was obtained as colorless liquid in 47% yield by preparative TLC on silica gel (petroleum ether/ethyl acetate = 50 : 1).

^{1}H NMR (400 MHz, CDCl$_3$): δ = 8.02 – 7.99 (m, 2H), 7.41 – 7.37 (m, 2H), 3.91 (s, 3H), 2.35 (t, *J* = 2.4 Hz, 2H), 1.15 (q, *J* = 7.5 Hz, 2H), 0.74 (t, *J* = 7.5 Hz, 3H), 0.71 (s, 6H). **^{19}F NMR (376 MHz, CDCl$_3$):** δ = -87.66 (d, *J* = 36.7 Hz), -90.20 (d, *J* = 36.6 Hz). **^{13}C NMR (100 MHz, CDCl$_3$):** δ = 166.9, 154.5 (dd, *J* = 291.8, 289.0 Hz), 140.8 (dd, *J* = 4.8, 2.8 Hz), 129.6, 128.8, 128.5 (t, *J* = 2.7 Hz), 90.8 (dd, *J* = 22.3, 12.6 Hz), 52.2, 39.2, 35.4, 34.7, 26.6, 8.4. **HRMS (ESI, m/z):** calcd for $C_{16}H_{21}F_2O_2$ [M+H]$^+$: 283.1510, found: 283.1514.

methyl 4-(1,1-difluoro-4,4-dimethylhept-1-en-2-yl)benzoate 3p

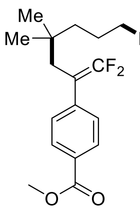
3p

Following general procedure, an inseparable mixture of **3p** and regioisomers (18.4 mg, 0.062 mmol) was obtained as colorless liquid in 31% yield by preparative TLC on silica gel (petroleum ether/ethyl acetate = 50 : 1).

^{1}H NMR (400 MHz, CDCl$_3$): δ = 8.06 – 7.96 (m, 2H), 7.40 – 7.38 (m, 2H), 3.92 (s, 3H), 2.35 (t, J = 2.2 Hz, 2H), 1.20 – 1.13 (m, 2H), 1.10 – 1.04 (m, 2H), 0.74 (t, J = 6.5 Hz, 3H), 0.72 (s, 6H). **^{19}F NMR (376 MHz, CDCl$_3$):** δ = -87.71 (d, J = 36.6 Hz), -90.22 (d, J = 36.7 Hz). **^{13}C NMR (100 MHz, CDCl$_3$):** δ = 166.9, 157.5 (dd, J = 291.4, 288.4 Hz), 140.8 (dd, J = 4.8, 2.8 Hz), 139.0 (dd, J = 4.5, 3.7 Hz), 129.7, 129.6, 128.9, 128.7, 128.6 (t, J = 2.3 Hz), 128.3 (t, J = 3.1 Hz), 91.39 (dd, J = 22.6, 12.2 Hz), 90.77 (dd, J = 22.1, 12.6 Hz), 52.3, 46.2, 44.9, 39.3, 35.4, 34.9, 28.9, 27.3, 25.3, 23.4, 22.2, 19.4, 17.3, 14.9 (signals of all isomers were shown). **HRMS (ESI, m/z):** calcd for $C_{17}H_{23}F_2O_2$ $[M+H]^+$: 297.1666, found: 297.1667.

methyl 4-(1,1-difluoro-4,4,6-trimethylhept-1-en-2-yl)benzoate 3q

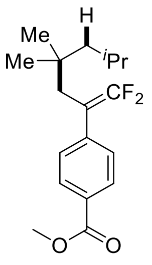
3q

Following general procedure, **3q** (39.1 mg, 0.126 mmol) was obtained as colorless liquid in 63% yield by preparative TLC on silica gel (petroleum ether/ethyl acetate = 50 : 1).

^{1}H NMR (400 MHz, CDCl$_3$): δ = 8.02 – 7.99 (m, 2H), 7.41 – 7.36 (m, 2H), 3.92 (s, 3H), 2.36 (t, J = 2.4 Hz, 2H), 1.65 – 1.55 (m, 1H), 1.08 (d, J = 5.2 Hz, 2H), 0.83 (d, J = 6.6 Hz, 6H), 0.74 (s, 6H). **^{19}F NMR (376 MHz, CDCl$_3$):** δ = -87.50 (d, J = 36.5 Hz), -90.17 (d, J = 36.3 Hz). **^{13}C NMR (100 MHz, CDCl$_3$):** δ = 166.9, 154.6 (dd, J = 291.7, 289.0 Hz), 140.9 (dd, J = 4.6, 3.0 Hz), 129.7, 128.8, 128.6 (t, J = 2.5 Hz), 90.7 (dd, J = 22.1, 12.4 Hz), 52.2, 51.8, 40.3, 36.2, 27.5, 25.5, 24.2. **HRMS (ESI, m/z):** calcd for $C_{18}H_{25}F_2O_2$ $[M+H]^+$: 311.1823, found: 311.1831.

methyl 4-(3-(1-ethylcyclohexyl)-1,1-difluoroprop-1-en-2-yl)benzoate 3r

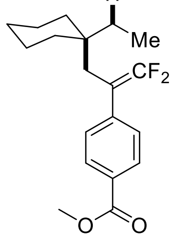
3r

Following general procedure, **3r** (31.6 mg, 0.098 mmol) was obtained as colorless liquid in 49% yield by preparative TLC on silica gel (petroleum ether/ethyl acetate = 50 : 1).

^{1}H NMR (400 MHz, CDCl$_3$): δ = 8.05 – 7.96 (m, 2H), 7.40 – 7.35 (m, 2H), 3.91 (s, 3H), 2.37 (t, J = 2.3 Hz, 2H), 1.43 – 1.22 (m, 5H), 1.25 – 1.12 (m, 5H), 1.09 – 1.01 (m, 2H), 0.64 (t, J = 7.5 Hz, 3H). **^{19}F NMR (376 MHz, CDCl$_3$):** δ = -87.55 (d, J =

37.4 Hz), -89.84 (d, *J* = 37.4 Hz). **^{13}C NMR (100 MHz, $CDCl_3$):** δ = 166.9, 154.4 (dd, *J* = 291.0, 289.9 Hz), 141.0 (dd, *J* = 5.0, 2.4 Hz), 129.6, 128.8, 128.7 (t, *J* = 2.4 Hz), 90.4 (dd, *J* = 22.3, 12.9 Hz), 52.2, 42.4, 37.6, 36.0, 35.2, 27.9, 26.2, 21.6, 15.5, 7.3. **HRMS (ESI, m/z):** calcd for $C_{19}H_{25}F_2O_2$ $[M+H]^+$: 323.1823, found: 323.1821.

methyl 4-(1,1-difluoro-5-(4-methoxyphenyl)-4,4-dimethylpent-1-en-2-yl)benzoate 3s

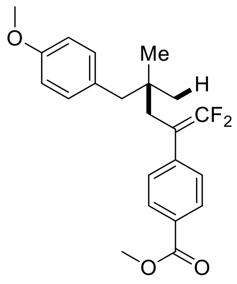

3s

Following general procedure, **3s** (41.2 mg, 0.110 mmol) was obtained as colorless liquid in 55% yield by preparative TLC on silica gel (petroleum ether/ethyl acetate = 20 : 1).

^{1}H NMR (400 MHz, $CDCl_3$): δ = 8.02 – 7.99 (m, 2H), 7.40 – 7.37 (m, 2H), 6.99 – 6.95 (m, 2H), 6.81 – 6.77 (m, 2H), 3.91 (s, 3H), 3.79 (s, 3H), 2.43 (t, *J* = 2.2 Hz, 2H), 2.41 (s, 2H), 0.69 (s, 6H). **^{19}F NMR (376 MHz, $CDCl_3$):** δ = -87.10 (d, *J* = 35.7 Hz), -89.69 (d, *J* = 35.6 Hz). **^{13}C NMR (100 MHz, $CDCl_3$):** δ = 166.8, 158.1, 154.6 (dd, *J* = 292.2, 289.2 Hz), 140.67 (dd, *J* = 4.4, 3.0 Hz), 131.6, 130.6, 129.7, 128.8, 128.5 (t, *J* = 2.4 Hz), 113.2, 90.6 (dd, *J* = 22.1, 12.5 Hz), 55.3, 52.2, 48.5, 40.3, 36.6, 26.5. **HRMS (ESI, m/z):** calcd for $C_{22}H_{24}F_2NaO_3$ $[M+Na]^+$: 397.1591, found: 397.1599.

methyl 4-(1,1-difluoro-4-methylpent-1-en-2-yl)benzoate 3t

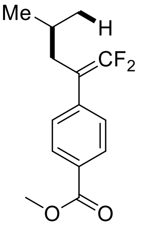

3t

Following general procedure, **3t** (27.0 mg, 0.106 mmol) was obtained as colorless liquid in 53% yield by preparative TLC on silica gel (petroleum ether/ethyl acetate = 50 : 1).

^{1}H NMR (400 MHz, $CDCl_3$): δ = 8.03 – 8.00 (m, 2H), 7.41 – 7.37 (m, 2H), 3.92 (s, 3H), 2.30 (dt, *J* = 7.3, 2.5 Hz, 2H), 1.61 – 1.51 (m, 1H), 0.87 (d, *J* = 6.6 Hz, 6H). **^{19}F NMR (376 MHz, $CDCl_3$):** δ = -89.14 (d, *J* = 39.2 Hz), -89.80 (d, *J* = 39.1 Hz). **^{13}C NMR (100 MHz, $CDCl_3$):** δ = 166.9, 154.3 (dd, *J* = 292.2, 287.5 Hz), 139.0 (t, *J* = 4.0 Hz), 129.7, 128.9, 128.3 (t, *J* = 3.2 Hz), 91.5 (dd, *J* = 22.6, 12.0 Hz), 52.2, 36.4, 26.6, 22.1. **HRMS (ESI, m/z):** calcd for $C_{14}H_{17}F_2O_2$ $[M+H]^+$: 255.1197, found: 255.1200.

methyl (R)-4-(1,1-difluoro-4-methylhex-1-en-2-yl)benzoate 3u

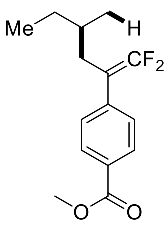

3u

Following general procedure, **3u** (15.0 mg, 0.056 mmol) was obtained as colorless liquid in 28% yield by preparative TLC on silica gel (petroleum ether/ethyl acetate = 50 : 1).

^{1}H NMR (400 MHz, $CDCl_3$): δ = 8.04 – 7.98 (m, 2H), 7.40 – 7.36 (m, 2H), 3.92 (s, 3H), 2.45 – 2.39 (m, 1H), 2.26 – 2.19 (m, 1H), 1.38 – 1.28 (m, 2H), 1.18 – 1.10 (m, 1H), 0.829 (t, *J* = 7.3 Hz, 3H), 0.828 (d, *J* = 6.5 Hz, 3H). **^{19}F NMR (376 MHz, $CDCl_3$):** δ = -89.24 (d, *J* = 39.4 Hz), -89.69 (d, *J* = 39.6 Hz). **^{13}C NMR (100 MHz, $CDCl_3$):** δ = 166.9, 154.3 (dd, *J* = 291.6, 286.7 Hz), 139.0 (t, *J* = 4.0 Hz), 129.7, 128.9, 128.3 (t, *J* = 3.2 Hz), 91.4 (dd, *J* = 22.5, 11.9 Hz), 52.2, 34.4, 32.9, 29.1, 18.7, 11.3. **HRMS (ESI, m/z):** calcd for $C_{15}H_{19}F_2O_2$ $[M+H]^+$: 269.1353, found: 269.1358.

methyl (S)-4-(1,1-difluoro-4-phenylpent-1-en-2-yl)benzoate 3v

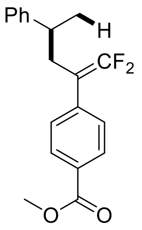

3v

Following general procedure, **3v** (56.9 mg, 0.180 mmol) was obtained as colorless liquid in 90% yield by preparative TLC on silica gel (petroleum ether/ethyl acetate = 50 : 1).

^{1}H NMR (400 MHz, $CDCl_3$): δ = 8.02 – 7.99 (m, 2H), 7.35 – 7.20 (m, 4H), 7.23 – 7.13 (m, 1H), 7.12 – 7.03 (m, 2H), 3.92 (s, 3H), 2.72 – 2.63 (m, 3H), 1.23 (d, *J* = 6.4 Hz, 3H). **^{19}F NMR (376 MHz, $CDCl_3$):** δ = -88.78 (d, *J* = 37.5 Hz), -89.63 (d, *J* = 37.5 Hz). **^{13}C NMR (100 MHz, $CDCl_3$):** δ = 166.9, 154.3 (dd, *J* = 292.2, 288.2 Hz), 145.8, 138.6 (t, *J* = 4.0 Hz), 129.8, 129.0, 128.5, 128.4 (t, *J* = 3.2 Hz), 127.0, 126.5, 91.2 (dd, *J* = 22.3, 12.9 Hz), 52.3, 38.0, 36.1, 21.3. **HRMS (ESI, m/z):** calcd for $C_{19}H_{19}F_2O_2$ $[M+H]^+$: 317.1353, found: 317.1349.

methyl 4-(1,1-difluoro-4-methyl-4-phenylpent-1-en-2-yl)benzoate 3w

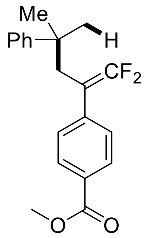

3w

Following general procedure, **3w** (33.0 mg, 0.100 mmol) was obtained as colorless liquid in 50% yield by preparative TLC on silica gel (petroleum ether/ethyl acetate = 50 : 1).

^{1}H NMR (400 MHz, $CDCl_3$): δ = 7.89 – 7.86 (m, 2H), 7.23 – 7.20 (m, 2H), 7.19 – 7.13 (m, 4H), 7.11 – 7.03 (m, 1H), 3.91 (s, 3H), 2.74 (t, *J* = 2.3 Hz, 2H), 1.23 (s, 6H). **^{19}F NMR (376 MHz, $CDCl_3$):** δ = -87.61 (d, *J* = 35.1 Hz), -89.90 (d, *J* = 35.4 Hz). **^{13}C NMR (100 MHz, $CDCl_3$):** δ = 166.9, 154.7 (dd, *J* = 291.8, 289.8 Hz), 148.1, 140.0 (dd, *J* = 4.6, 3.0 Hz), 129.4, 128.5 (t, *J*

= 2.4 Hz), 128.0, 127.0, 125.94, 125.88, 90.7 (dd, J = 21.7, 13.3 Hz), 52.2, 42.2, 39.3, 28.5. **HRMS (ESI, m/z):** calcd for $C_{20}H_{21}F_2O_2$ $[M+H]^+$: 331.1510, found: 331.1502.

methyl 4-(1,1-difluoro-3-(4-methyl-1-(2-(11-oxo-6,11-dihydrodibenzo[b,e]oxepin-2-yl)acetyl) piperidin-4-yl)prop-1-en-2-yl)benzoate 3x

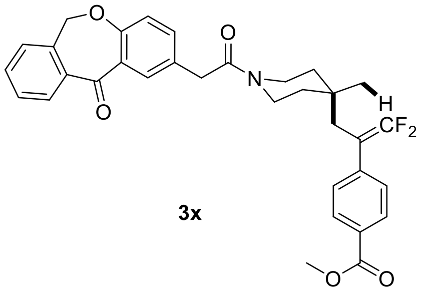

3x

Following general procedure, **3x** (78.3 mg, 0.140 mmol) was obtained as colorless liquid in 70% yield by preparative TLC on silica gel (petroleum ether/ethyl acetate = 1 : 1).

^{1}H NMR (400 MHz, CDCl$_3$): δ = 8.03 – 7.95 (m, 3H), 7.88 – 7.86 (m, 1H), 7.57 – 7.53 (m, 1H), 7.48 – 7.44 (m, 1H), 7.39 – 7.34 (m, 4H), 7.02 – 6.99 (m, 1H), 5.17 (s, 2H), 3.95 – 3.91 (m, 1H), 3.90 (s, 3H), 3.65 (s, 2H), 3.51 – 3.45 (m, 1H), 3.20 – 3.14 (m, 1H), 3.09 – 3.03 (m, 1H), 2.40 (s, 2H), 1.33 – 1.27 (m, 2H), 1.19 – 1.10 (m, 2H), 0.83 (s, 3H). **^{19}F NMR (376 MHz, CDCl$_3$):** δ = -86.62 (d, J = 35.1 Hz), -89.15 (d, J = 35.0 Hz). **^{13}C NMR (100 MHz, CDCl$_3$):** δ = 191.0, 169.1, 166.7, 160.4, 157.7 (dd, J = 292.9, 289.3 Hz), 140.5, 140.1 (dd, J = 4.5, 3.3 Hz), 136.1, 135.7, 132.9, 131.8, 129.9, 129.6, 129.4, 129.1, 128.4, 127.9, 125.2, 121.3, 89.66 (dd, J = 22.0, 13.3 Hz), 73.7, 52.3, 42.4, 39.8, 39.7, 38.1, 37.4, 36.8, 34.2, 23.2. **HRMS (ESI, m/z):** calcd for $C_{33}H_{32}F_2NO_5$ $[M+H]^+$: 560.2249, found: 560.2244.

methyl 4-(3-(1-(2-(1-(4-chlorobenzoyl)-5-methoxy-2-methyl-1H-indol-3-yl)acetyl) -4-methylpiperidin-4-yl)-1,1-difluoroprop-1-en-2-yl)benzoate 3y

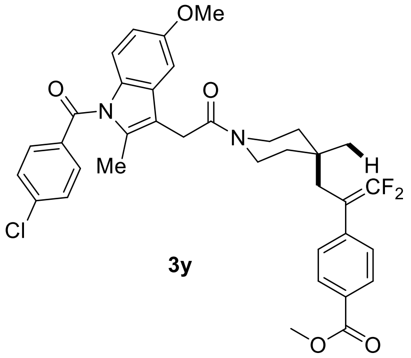

3y

Following general procedure, **3y** (84.3 mg, 0.130 mmol) was obtained as colorless liquid in 65% yield by preparative TLC on silica gel (petroleum ether/ethyl acetate = 1 : 1).

^{1}H NMR (400 MHz, CDCl$_3$): δ = 8.00 - 7.97 (m, 2H), 7.68 – 7.59 (m, 2H), 7.46 – 7.44 (m, 2H), 7.38 – 7.25 (m, 2H), 7.03 – 6.94 (m, 1H), 6.84 – 6.81 (m, 1H), 6.66 – 6.62 (m, 1H), 4.00 – 3.94 (m, 1H), 3.90 (s, 3H), 3.79 (s, 3H), 3.72 – 3.61 (m, 2H), 3.52 – 3.47 (m, 1H), 3.22 – 3.15 (m, 1H), 3.06 – 2.99 (m, 1H), 2.35 – 2.32 (m, 5H), 1.26 – 1.13 (m, 2H), 1.09 – 1.02 (m, 2H), 0.81 (s, 3H). **^{19}F NMR (376 MHz, CDCl$_3$):** δ = -86.61 (d, J = 34.9 Hz), -89.11 (d, J = 34.9 Hz). **^{13}C NMR (100 MHz, CDCl$_3$):** δ = 168.5, 168.3, 166.7, 156.1, 154.6 (dd, J = 292.2, 289.3 Hz), 140.1 (dd, J = 4.6, 2.9 Hz),

139.3, 135.0, 134.0, 131.3, 130.9, 130.8, 129.9, 129.2, 129.1, 128.4, 115.0, 113.6, 111.6, 101.6, 89.6 (dd, *J* = 22.2, 13.0 Hz), 55.7, 52.3, 42.4, 39.9, 38.3, 37.2, 36.8, 34.2, 30.6, 23.1, 13.5. **HRMS (ESI, m/z):** calcd for $C_{36}H_{36}ClF_2N_2O_5$ $[M+H]^+$: 649.2281, found: 649.2280.

methyl 4-(1,1-difluoro-3-((1S,2R)-2-(methoxycarbonyl)-1-methylcyclohexyl)prop-1-en-2-yl)benzoate 3z

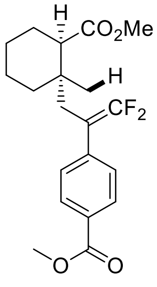

3z

Following general procedure, **3z** (40.3 mg, 0.110 mmol) was obtained as colorless liquid in 55% yield by preparative TLC on silica gel (petroleum ether/ethyl acetate = 20 : 1). **^{1}H NMR (400 MHz, $CDCl_3$):** δ = 8.00 – 7.98 (m, 2H), 7.37 – 7.34 (m, 2H), 3.90 (s, 3H), 3.58 (s, 3H), 2.57 – 2.44 (m, 2H), 2.22 – 2.13 (m, 1H), 1.70 – 1.58 (m, 3H), 1.41 – 1.27 (m, 3H), 1.16 – 1.06 (m, 1H), 1.03 - 0.95 (m, 1H), 0.85 (s, 3H). **^{19}F NMR (376 MHz, $CDCl_3$):** δ = -86.63 (d, *J* = 34.8 Hz), -89.26 (d, *J* = 34.8 Hz). **^{13}C NMR (100 MHz, $CDCl_3$):** δ = 175.0, 166.9, 154.6 (dd, *J* = 291.7, 289.3 Hz), 140.5 (dd, *J* = 4.6, 2.9 Hz), 129.7, 128.9, 128.6 (t, *J* = 2.4 Hz), 89.8 (dd, *J* = 21.9, 13.1 Hz), 52.3, 51.5, 51.3, 40.1, 37.9, 37.6, 25.5, 25.1, 21.3, 19.3. **HRMS (ESI, m/z):** calcd for $C_{20}H_{25}F_2O_4$ $[M+H]^+$: 367.1721, found: 367.1717.

methyl 4-(1,1-difluoro-4,4-dimethylpent-1-en-2-yl)benzoate 3g

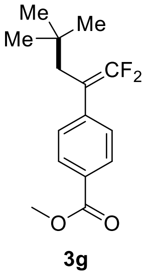

3g

Following general procedure, **3g** (45.1 mg, 0.168 mmol) was obtained as colorless liquid in 84% yield by preparative TLC on silica gel (petroleum ether/ethyl acetate = 50 : 1).

1-(4-(1,1-difluoro-3-(1-methylcyclohexyl)prop-1-en-2-yl)phenyl)ethan-1-one 3aa

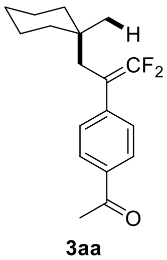

3aa

Following general procedure, **3aa** (40.3 mg, 0.138 mmol) was obtained as colorless liquid in 69% yield by preparative TLC on silica gel (petroleum ether/ethyl acetate = 50 : 1).

^{1}H NMR (400 MHz, $CDCl_3$): δ = 7.94 – 7.91 (m, 2H), 7.44 – 7.40 (m, 2H), 2.60 (s, 3H), 2.38 (t, *J* = 2.4 Hz, 2H), 1.45 – 1.12 (m, 10H), 0.75 (s, 3H). **^{19}F NMR (376 MHz, $CDCl_3$):** δ = -87.10 (d, *J* = 36.1 Hz), -90.01 (d, *J* = 36.0 Hz). **^{13}C NMR (100 MHz, $CDCl_3$):** δ = 197.7,

154.6 (dd, J = 292.8, 288.3 Hz), 141.2 (dd, *J* = 5.1, 3.0 Hz), 135.7, 128.7 (t, *J* = 2.6 Hz), 128.4, 90.4 (dd, *J* = 22.5, 12.4 Hz), 40.2, 38.0, 35.4, 26.7, 26.3, 24.7, 22.0. **HRMS (ESI, m/z):** calcd for $C_{18}H_{23}F_2O$ $[M+H]^+$: 293.1717, found: 293.1716.

4-(1,1-difluoro-3-(1-methylcyclohexyl)prop-1-en-2-yl)-N,N-dimethylbenzamide 3ab

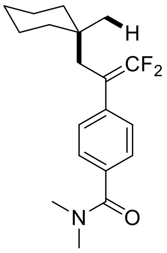

3ab

Following general procedure, **3ab** (48.2 mg, 0.150 mmol) was obtained as colorless liquid in 75% yield by preparative TLC on silica gel (petroleum ether/ethyl acetate = 5 : 1).

^{1}H NMR (400 MHz, CDCl$_3$): δ = 7.40 – 7.38 (m, 2H), 7.35 – 7.33 (m, 2H), 3.04 (d, *J* = 47.4 Hz, 6H), 2.35 (t, *J* = 2.3 Hz, 2H), 1.42 – 1.10 (m, 10H), 0.75 (s, 3H). **^{19}F NMR (376 MHz, CDCl$_3$):** δ = -88.17 (d, *J* = 38.8 Hz), -91.13 (d, *J* = 38.7 Hz). **^{13}C NMR (100 MHz, CDCl$_3$):** δ = 171.4, 154.5 (dd, *J* = 290.8, 288.0 Hz), 137.4 (dd, *J* = 4.7, 2.7 Hz), 134.8, 128.5 (t, *J* = 2.4 Hz), 127.3, 90.3 (dd, *J* = 22.1, 12.6 Hz), 40.3, 39.7, 38.1, 35.5, 35.3, 26.3, 24.7, 22.0. **HRMS (ESI, m/z):** calcd for $C_{19}H_{26}F_2NO$ $[M+H]^+$: 322.1982, found: 322.1983.

4-(1,1-difluoro-3-(1-methylcyclohexyl)prop-1-en-2-yl)benzonitrile 3ac

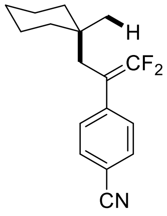

3ac

Following general procedure, **3ac** (38.1 mg, 0.138 mmol) was obtained as colorless liquid in 69% yield by preparative TLC on silica gel (petroleum ether/ethyl acetate = 5 : 1).

^{1}H NMR (400 MHz, CDCl$_3$): δ = 7.64 – 7.59 (m, 2H), 7.45 – 7.40 (m, 2H), 2.36 (t, J = 2.4 Hz, 2H), 1.45 – 1.09 (m, 10H), 0.74 (s, 3H). **^{19}F NMR (376 MHz, CDCl$_3$):** δ = -86.12 (d, *J* = 34.3 Hz), -89.30 (d, *J* = 34.4 Hz). **^{13}C NMR (100 MHz, CDCl$_3$):** δ = 154.7 (dd, J = 292.2, 289.3 Hz), 141.1 (dd, *J* = 5.0, 2.6 Hz), 132.2, 129.2 (t, *J* = 2.5 Hz), 118.8, 110.8, 90.1 (dd, *J* = 23.2, 12.1 Hz), 40.2, 38.0, 35.4, 26.2, 24.7, 22.0. **HRMS (ESI, m/z):** calcd for $C_{17}H_{19}F_2NNa$ $[M+Na]^+$: 298.1383, found: 298.1384.

1-(1,1-difluoro-3-(1-methylcyclohexyl)prop-1-en-2-yl)-4-(trifluoromethyl)benzene 3ad

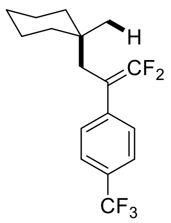

3ad

Following general procedure, **3ad** (48.3 mg, 0.152 mmol) was obtained as colorless liquid in 76% yield by preparative TLC on silica gel (petroleum ether/ethyl acetate = 50 : 1).

^{1}H NMR (400 MHz, CDCl$_3$): δ = 7.59 (d, *J* = 8.2 Hz, 2H), 7.43 (d, *J* = 8.1 Hz, 2H), 2.37 (t, *J* = 2.4 Hz, 2H), 1.46 – 1.11 (m, 10H), 0.75 (s, 3H). **^{19}F NMR (376 MHz, CDCl$_3$):** δ = -62.44, -87.38 (d, *J* = 37.0 Hz), -90.47 (d, *J* = 37.1 Hz). **^{13}C NMR (100 MHz, CDCl$_3$):** δ = 154.6 (dd, *J* = 291.3, 289.3 Hz), 139.9, 129.1 (q, *J* = 32.6 Hz), 128.9 (t, *J* = 2.7 Hz), 125.3 (q, *J* = 3.6 Hz), 124.2 (q, J = 271.8 Hz), 90.1 (dd, *J* = 22.7, 12.4 Hz), 40.3, 38.0, 35.4, 26.3, 24.7, 22.0. **HRMS (ESI, m/z):** calcd for $C_{17}H_{20}F_5$ [M+H]$^+$: 319.1485, found: 319.1497.

1-(1,1-difluoro-3-(1-methylcyclohexyl)prop-1-en-2-yl)-4-(trifluoromethoxy)benzene 3ae

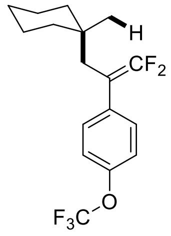

3ae

Following general procedure, **3ae** (31.4 mg, 0.094 mmol) was obtained as colorless liquid in 47% yield by preparative TLC on silica gel (petroleum ether/ethyl acetate = 50 : 1).

^{1}H NMR (400 MHz, CDCl$_3$): δ = 7.38 – 7.28 (m, 2H), 7.23 – 7.12 (m, 2H), 2.34 (t, *J* = 2.4 Hz, 2H), 1.44 -1.09 (m, 10H), 0.75 (s, 3H). **^{19}F NMR (376 MHz, CDCl$_3$):** δ = -57.75, -88.48 (d, *J* = 39.5 Hz), -91.34 (d, *J* = 39.6 Hz). **^{13}C NMR (100 MHz, CDCl$_3$):** δ = 154.5 (dd, *J* = 290.6, 288.8 Hz), 148.0, 134.7 (dd, *J* = 5.1, 2.6 Hz), 129.9, 120.8, 120.5 (q, *J* = 256.8 Hz), 89.8 (dd, *J* = 22.5, 13.1 Hz), 40.4, 38.1, 35.3, 26.3, 24.7, 22.0. **HRMS (ESI, m/z):** calcd for $C_{17}H_{20}F_5O$ [M+H]$^+$: 335.1434, found: 335.1427.

1-(1,1-difluoro-3-(1-methylcyclohexyl)prop-1-en-2-yl)-4-(methylsulfonyl)benzene 3af

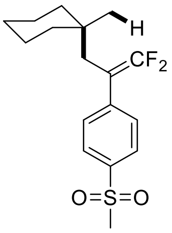

3af

Following general procedure, **3af** (48.0 mg, 0.146 mmol) was obtained as colorless liquid in 73% yield by preparative TLC on silica gel (petroleum ether/ethyl acetate = 5 : 1).

^{1}H NMR (400 MHz, CDCl$_3$): δ = 7.90 (d, *J* = 8.4 Hz, 2H), 7.52 (d, *J* = 7.8 Hz, 2H), 3.07 (s, 3H), 2.39 (s, 2H), 1.45 – 1.07 (m, 10H), 0.74 (s, 3H). **^{19}F NMR (376 MHz, CDCl$_3$):** δ = -86.09 (d, *J* = 34.4 Hz), -89.45 (d, *J* = 34.4 Hz). **^{13}C NMR (100 MHz, CDCl$_3$):** δ = 154.7 (dd, *J* = 292.4, 290.1 Hz), 142.1 (dd, *J* = 5.2, 3.1 Hz), 138.9, 129.4 (t, *J* = 2.7 Hz), 127.5, 90.1 (dd, *J* =

23.2, 12.2 Hz), 44.6, 40.4, 38.0, 35.4, 26.2, 24.6, 22.0. **HRMS (ESI, m/z):** calcd for $C_{17}H_{23}F_2O_2S$ $[M+H]^+$: 329.1387, found: 329.1393.

1-(1,1-difluoro-3-(1-methylcyclohexyl)prop-1-en-2-yl)-4-methylbenzene 3ag

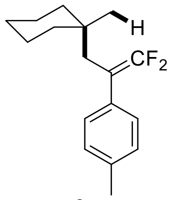

3ag

Following general procedure, **3ag** (29.5 mg, 0.112 mmol) was obtained as colorless liquid in 56% yield by preparative TLC on silica gel (petroleum ether/ethyl acetate = 50 : 1).

^{1}H NMR (400 MHz, CDCl$_3$): δ = 7.22 – 7.18 (m, 2H), 7.14 (d, *J* = 8.1 Hz, 2H), 2.344 (s, 3H), 2.337 (t, *J* = 2.4 Hz, 2H), 1.44 – 1.15 (m, 10H), 0.77 (s, 3H). **^{19}F NMR (376 MHz, CDCl$_3$):** δ = -89.88 (d, *J* = 42.5 Hz), -92.50 (d, *J* = 42.4 Hz). **^{13}C NMR (100 MHz, CDCl$_3$):** δ = 157.4 (dd, *J* = 288.9, 287.1 Hz), 136.7, 132.9 (dd, *J* = 4.4, 2.5 Hz), 129.0, 128.4 (t, *J* = 2.4 Hz), 90.5 (dd, *J* = 21.4, 13.3 Hz), 40.4, 38.1, 35.2, 26.4, 24.7, 22.1, 21.2. **HRMS (ESI, m/z):** calcd for $C_{17}H_{23}F_2$ $[M+H]^+$: 265.1768, found: 265.1770.

1-(1,1-difluoro-3-(1-methylcyclohexyl)prop-1-en-2-yl)-3-methoxybenzene 3ah

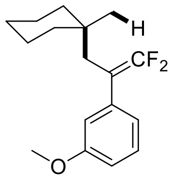

3ah

Following general procedure, **3ah** (45.4 mg, 0.162 mmol) was obtained as colorless liquid in 81% yield by preparative TLC on silica gel (petroleum ether/ethyl acetate = 50 : 1).

^{1}H NMR (400 MHz, CDCl$_3$): δ = 7.25 (t, *J* = 8.0 Hz, 1H), 6.91 (d, *J* = 7.6 Hz, 1H), 6.86 (s, 1H), 6.79 (dd, *J* = 8.2, 2.1 Hz, 1H), 3.82 (s, 3H), 2.34 (t, J =2.4 Hz, 2H), 1.45 – 1.15 (m, 10H), 0.77 (s, 3H). **^{19}F NMR (376 MHz, CDCl$_3$):** δ = -89.18 (d, *J* = 40.6 Hz), -91.38 (d, *J* = 40.6 Hz). **^{13}C NMR (100 MHz, CDCl$_3$):** δ = 159.4, 154.4 (dd, *J* = 289.9, 287.2 Hz), 137.4 (dd, *J* = 4.5, 2.5 Hz), 129.2, 121.1 (t, *J* = 2.4 Hz), 114.7 (t, *J* = 2.6 Hz), 112.2, 90.6 (dd, *J* = 21.8, 12.9 Hz), 55.3, 40.4, 38.0, 35.3 (t, *J* = 2.2 Hz), 26.4, 24.6, 22.1. **HRMS (ESI, m/z):** calcd for $C_{17}H_{23}F_2O$ $[M+H]^+$: 281.1717, found: 281.1713.

5-(1,1-difluoro-3-(1-methylcyclohexyl)prop-1-en-2-yl)-1,2,3-trimethoxybenzene 3ai

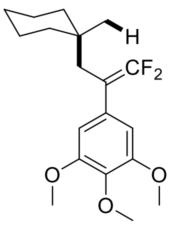

3ai

Following general procedure, **3ai** (47.7 mg, 0.140 mmol) was obtained as colorless liquid in 70% yield by preparative TLC on silica gel (petroleum ether/ethyl acetate = 10 : 1).

^{1}H NMR (400 MHz, CDCl$_3$): δ = 6.51 (s, 2H), 3.85 (s, 9H), 2.31 (t, *J* = 2.1 Hz, 2H), 1.46 – 1.15 (m, 10H), 0.78 (s, 3H). **^{19}F NMR (376 MHz, CDCl$_3$):** δ = -89.35 (d, *J* = 41.5 Hz), -91.11 (d, *J* = 41.4 Hz). **^{13}C NMR (100 MHz, CDCl$_3$):** δ = 154.4 (dd, *J* = 289.4, 287.4 Hz), 153.0, 137.1, 131.4 (dd, *J* = 4.7, 2.5 Hz), 105.9 (t, *J* = 2.5 Hz), 90.8 (dd, *J* = 21.9, 12.8 Hz), 61.0, 56.3, 40.4, 38.0, 35.2 (t, *J* = 2.2 Hz), 26.4, 24.7, 22.1. **HRMS (ESI, m/z):** calcd for $C_{19}H_{27}F_2O_3$ $[M+H]^+$: 341.1928, found: 341.1927.

1-chloro-4-(1,1-difluoro-3-(1-methylcyclohexyl)prop-1-en-2-yl)benzene 3aj

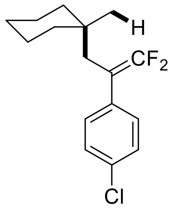

3aj

Following general procedure, **3aj** (44.5 mg, 0.156 mmol) was obtained as colorless liquid in 78% yield by preparative TLC on silica gel (petroleum ether/ethyl acetate = 50 : 1).

^{1}H NMR (400 MHz, CDCl$_3$): δ = 7.32 – 7.27 (m, 2H), 7.25 – 7.22 (m, 2H), 2.32 (t, *J* = 2.4 Hz, 2H), 1.44 – 1.08 (m, 10H), 0.74 (s, 3H). **^{19}F NMR (376 MHz, CDCl$_3$):** δ = -88.59 (d, *J* = 39.7 Hz), -91.35 (d, *J* = 39.6 Hz). **^{13}C NMR (100 MHz, CDCl$_3$):** δ = 154.4 (dd, *J* = 290.2, 288.4 Hz), 134.5 (dd, *J* = 4.8, 2.7 Hz), 132.8, 129.9 (t, *J* = 2.6 Hz), 128.6, 89.9 (dd, *J* = 22.4, 12.9 Hz), 40.3, 38.1, 35.3, 26.3, 24.7, 22.0. **HRMS (ESI, m/z):** calcd for $C_{16}H_{20}ClF_2$ $[M+H]^+$: 285.1222, found: 285.1236.

1,2-dichloro-4-(1,1-difluoro-3-(1-methylcyclohexyl)prop-1-en-2-yl)benzene 3ak

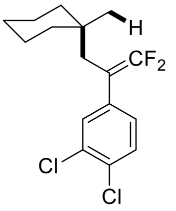

3ak

Following general procedure, **3ak** (48.4 mg, 0.152 mmol) was obtained as colorless liquid in 76% yield by preparative TLC on silica gel (petroleum ether/ethyl acetate = 50 : 1).

^{1}H NMR (400 MHz, CDCl$_3$): δ = 7.42 – 7.38 (m, 2H), 7.17 – 7.14 (m, 1H), 2.31 (t, *J* = 2.5 Hz, 2H), 1.50 – 1.08 (m, 10H), 0.76 (s, 3H). **^{19}F NMR (376 MHz, CDCl$_3$):** δ = -87.32 (d, *J* = 37.0 Hz), -90.04 (d, *J* = 36.9 Hz). **^{13}C NMR (100 MHz, CDCl$_3$):** δ = 154.6 (dd, *J* = 291.7, 289.2 Hz), 136.1 (dd, *J* = 5.0, 2.9 Hz), 132.5, 131.1, 130.4 (t, *J* = 2.8 Hz), 130.3, 127.9 (t, *J* = 2.7 Hz),

89.3 (dd, J = 23.2, 12.6 Hz), 40.3, 38.1, 35.4, 26.3, 24.6, 22.0. **HRMS (ESI, m/z):** calcd for $C_{16}H_{19}Cl_2F_2$ $[M+H]^+$: 319.0832, found: 319.0826.

1-(1,1-difluoro-3-(1-methylcyclohexyl)prop-1-en-2-yl)-2-fluorobenzene 3al

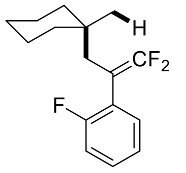

3al

Following general procedure, **3al** (27.3 mg, 0.102 mmol) was obtained as colorless liquid in 51% yield by preparative TLC on silica gel (petroleum ether/ethyl acetate = 50 : 1).

^{1}H NMR (400 MHz, CDCl$_3$): δ = 7.28 – 7.21 (m, 2H), 7.13 – 7.01 (m, 2H), 2.33 (t, J = 2.2 Hz, 2H), 1.42 – 1.10 (m, 10H), 0.77 (s, 3H). **^{19}F NMR (376 MHz, CDCl$_3$):** δ = -87.38 (dd, J = 35.5, 14.8 Hz), -89.14 (d, J = 35.4 Hz), -113.20 – -113.32 (m). **^{13}C NMR (100 MHz, CDCl$_3$):** δ = 161.0, 158.6, 154.1 (t, J = 289.0 Hz), 130.8, 129.2 (d, J = 8.3 Hz), 124.0 (d, J = 3.5 Hz), 115.9 (d, J = 22.4 Hz), 84.8 (dd, J = 24.9, 15.5 Hz), 40.3, 37.9, 35.2, 26.4, 24.6, 22.0. **HRMS (ESI, m/z):** calcd for $C_{16}H_{20}F_3$ $[M+H]^+$: 269.1517, found: 269.1525.

6-(1,1-difluoro-3-(1-methylcyclohexyl)prop-1-en-2-yl)-2,3-dihydrobenzo[b][1,4]dioxine 3am

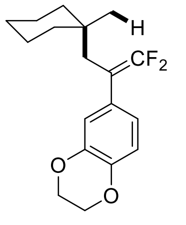

3am

Following general procedure, **3am** (43.7 mg, 0.142 mmol) was obtained as colorless liquid in 71% yield by preparative TLC on silica gel (petroleum ether/ethyl acetate = 20 : 1).

^{1}H NMR (400 MHz, CDCl$_3$): δ = 6.83 – 6.76 (m, 3H), 4.26 (s, 4H), 2.28 (t, J = 2.4 Hz, 2H), 1.47 – 1.11 (m, 10H), 0.76 (s, 3H). **^{19}F NMR (376 MHz, CDCl$_3$):** δ = -89.88 (d, J = 42.4 Hz), -92.28 (d, J = 42.5 Hz). **^{13}C NMR (100 MHz, CDCl$_3$):** δ = 154.4 (dd, J = 289.3, 287.5 Hz), 143.2, 142.6, 129.1 (dd, J = 4.6, 2.5 Hz), 121.7 (t, J = 2.5 Hz), 117.4 (t, J = 2.6 Hz), 117.0, 90.0 (dd, J = 22.0, 13.0 Hz), 64.44, 64.42, 40.4, 38.0, 35.2, 26.4, 24.6, 22.1. **HRMS (ESI, m/z):** calcd for $C_{18}H_{22}F_2NaO_2$ $[M+Na]^+$: 331.1486, found: 331.1496.

3-(1,1-difluoro-3-(1-methylcyclohexyl)prop-1-en-2-yl)quinoline 3an

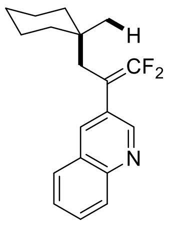

3an

Following general procedure, **3an** (27.0 mg, 0.090 mmol) was obtained as colorless liquid in 45% yield by preparative TLC on silica gel (petroleum ether/ethyl acetate = 20 : 1).

^{1}H NMR (400 MHz, CDCl$_3$): δ = 8.90 (s, 1H), 8.11 – 8.03 (m, 2H), 7.81 (d, *J* = 8.1 Hz, 1H), 7.71 (t, *J* = 7.7 Hz, 1H), 7.56 (t, *J* = 7.5 Hz, 1H), 2.48 (t, *J* = 2.5 Hz, 2H), 1.44 – 1.11 (m, 10H), 0.79 (s, 3H). **^{19}F NMR (376 MHz, CDCl$_3$):** δ = -86.75 (d, *J* = 36.6 Hz), -90.46 (d, *J* = 36.7 Hz). **^{13}C NMR (100 MHz, CDCl$_3$):** δ = 155.0 (t, *J* = 291.1, 290.0 Hz), 150.7, 147.0, 134.9 (t, *J* = 2.4 Hz), 129.7, 129.6, 129.3, 127.8, 127.7, 127.1, 88.2 (dd, *J* = 23.4, 13.1 Hz), 40.4, 38.1, 35.5, 26.3, 24.8, 22.0. **HRMS (ESI, m/z):** calcd for $C_{19}H_{22}F_2N$ [M+H]$^+$: 302.1720, found: 302.1722.

5-(1,1-difluoro-3-(1-methylcyclohexyl)prop-1-en-2-yl)benzofuran 3ao

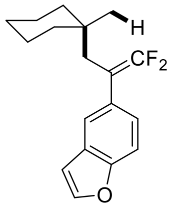

3ao

Following general procedure, **3ao** (46.5 mg, 0.160 mmol) was obtained as colorless liquid in 80% yield by preparative TLC on silica gel (petroleum ether/ethyl acetate = 20 : 1).

^{1}H NMR (400 MHz, CDCl$_3$): δ = 7.62 (d, *J* = 2.2 Hz, 1H), 7.53 (s, 1H), 7.46 (d, *J* = 8.6 Hz, 1H), 7.24 (dt, *J* = 8.6, 1.5 Hz, 1H), 6.75 (dd, *J* = 2.1, 0.7 Hz, 1H), 2.40 (t, *J* = 2.4 Hz, 2H), 1.43 – 1.13 (m, 10H), 0.77 (s, 3H). **^{19}F NMR (376 MHz, CDCl$_3$):** δ = -90.11 (d, *J* = 43.0 Hz), -92.83 (d, *J* = 42.9 Hz). **^{13}C NMR (100 MHz, CDCl$_3$):** δ = 154.5 (dd, J = 288.6, 287.4 Hz), 154.0, 145.5, 130.6 (dd, *J* = 4.6, 2.2 Hz), 127.5, 125.1 (t, *J* = 2.2 Hz), 121.3 (t, *J* = 2.4 Hz), 111.2, 106.7, 90.7 (dd, *J* = 21.6, 13.4 Hz), 41.0, 38.1, 35.3, 26.4, 24.8, 22.1. **HRMS (ESI, m/z):** calcd for $C_{18}H_{21}F_2O$ [M+H]$^+$: 291.1560, found: 291.1568.

5-(1,1-difluoro-3-(1-methylcyclohexyl)prop-1-en-2-yl)-2-methoxypyridine 3ap

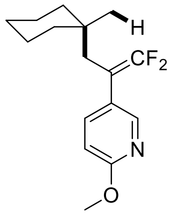

3ap

Following general procedure, **3ap** (37.1 mg, 0.132 mmol) was obtained as colorless liquid in 66% yield by preparative TLC on silica gel (petroleum ether/ethyl acetate = 20 : 1).

^{1}H NMR (400 MHz, CDCl$_3$): δ = 8.11 – 8.10 (m, 1H), 7.52 – 7.49 (m, 1H), 6.73 – 6.71 (m, 1H), 3.93 (s, 3H), 2.29 (t, *J* = 2.4 Hz, 2H), 1.44 – 1.26 (m, 6H), 1.16 – 1.13 (m, 4H), 0.76 (s, 3H). **^{19}F NMR (376 MHz, CDCl$_3$):** δ = -88.64 (d, *J* = 40.9 Hz), -91.82 (d, *J* = 40.8 Hz).

^{13}C NMR (100 MHz, $CDCl_3$): δ = 163.1, 154.5 (dd, *J* = 289.6, 288.5 Hz), 146.5 (dd, *J* = 3.3, 2.7 Hz), 138.7 (t, *J* = 2.3 Hz), 124.77 (dd, *J* = 4.7, 2.8 Hz), 110.6, 87.5 (dd, *J* = 23.1, 13.5 Hz), 53.5, 40.2, 38.1, 35.3 (t, *J* = 2.2 Hz), 26.3, 24.8, 22.0. **HRMS (ESI, m/z):** calcd for $C_{16}H_{22}F_2NO$ $[M+H]^+$: 282.1669, found: 282.1664.

1 mmol scale reaction

To an oven-dried 50 mL long-necked flask (see below) with branch equipped with a magnetic stirring bar was added $NiBr_2$·glyme (15.4 mg, 0.05 mmol, 5.0 mol%), **L1** (11.1 mg, 0.06 mmol, 6.0 mol%), Mn powder (82.4 mg, 1.5 mmol, 1.5 equiv). After flask was evacuated and refilled with nitrogen (3 times), THF (5.0 mL), **1a** (531.2 mg, 3.0 mmol, 3.0 equiv), and compound **2a** (230.2 mg, 1.0 mmol, 1.0 equiv) were added under nitrogen atmosphere. The flask was capped and stirred at 25 ℃ for 16 h. The reaction mixture was diluted with H_2O (10 mL) and extracted with ethyl acetate (3 × 10 mL). The combined organic layers were washed with water (10 mL), brine (10 mL), dried over anhydrous Na_2SO_4, filtered and evaporated under reduced pressure. The regioisomeric ratio of the product was determined as > 20:1 by ^{19}F NMR analysis of crude residue. **3a** (205.8 mg, 0.65 mmol) was isolated in 65% yield by column chromatography on silica gel (petroleum ether/ethyl acetate = 50:1).

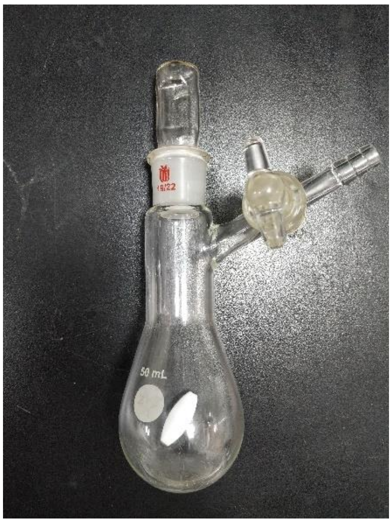

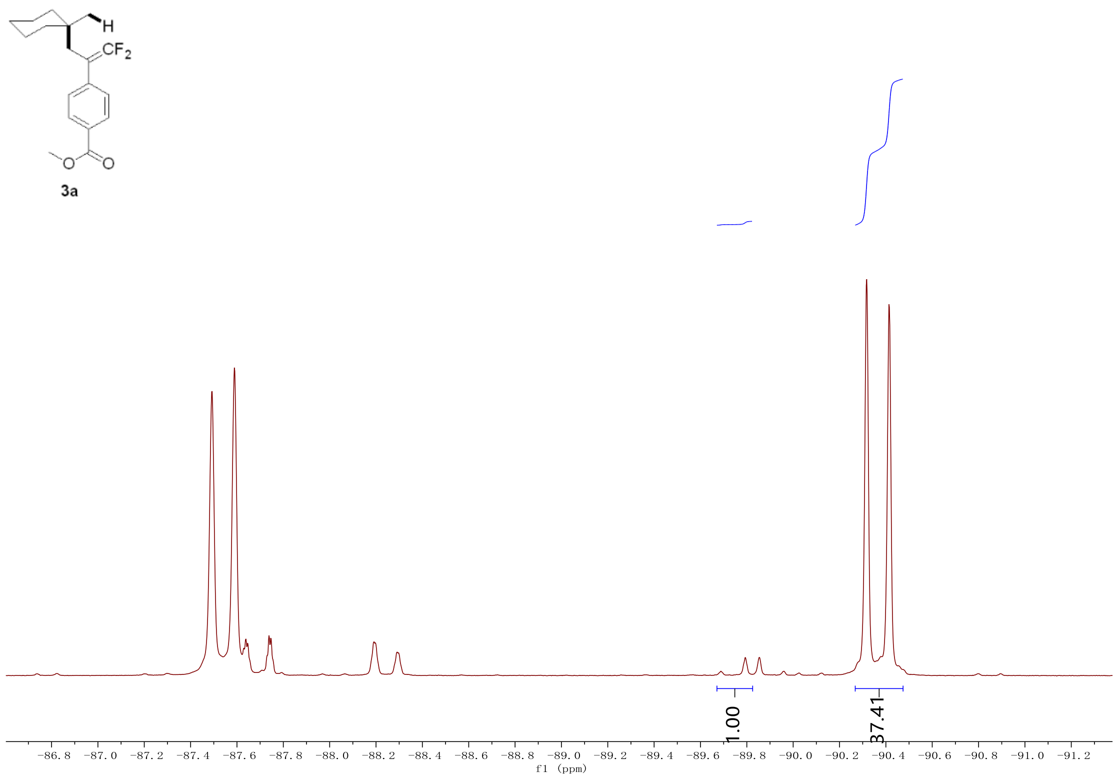

Supplementary Figure 2. Crude ^{19}F NMR spectrum of 1 mmol scale reaction

6-(benzyloxy)-3,3-dimethyl-6-oxohexyl benzoate 5a

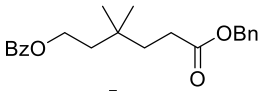

5a

Following general procedure, **5a** (44.6 mg, 0.126 mmol) was obtained as colorless liquid in 63% yield by preparative TLC on silica gel (petroleum ether/ethyl acetate = 20 : 1).

^{1}H NMR (400 MHz, CDCl$_3$): δ = 8.08 – 7.99 (m, 2H), 7.60 – 7.50 (m, 1H), 7.45 – 7.40 (m, 2H), 7.38 – 7.32 (m, 5H), 5.11 (s, 2H), 4.38 (t, J = 7.2 Hz, 2H), 2.42 – 2.35 (m, 2H), 1.73 – 1.70 (m, 2H), 1.70 – 1.66 (m, 2H), 0.98 (s, 6H). **^{13}C NMR (100 MHz, CDCl$_3$):** δ = 174.0, 166.8, 136.0, 133.0, 130.4, 129.6, 128.7, 128.5, 128.4, 128.4, 66.4, 62.2, 39.7, 36.9, 32.1, 29.7, 27.0. **HRMS (ESI, m/z):** calcd for $C_{22}H_{26}NaO_4$ [M+Na]$^+$: 377.1729, found: 377.1726.

3,3-dimethyl-6-oxooctyl benzoate 5b

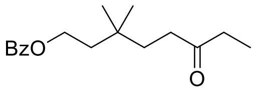

5b

Following general procedure, **5b** (28.7 mg, 0.104 mmol) was obtained as colorless liquid in 52% yield by preparative TLC on silica gel (petroleum ether/ethyl acetate = 20 : 1).

^{1}H NMR (400 MHz, CDCl$_3$): δ = 8.07 – 7.96 (m, 2H), 7.57 – 7.53 (m, 1H), 7.48 – 7.38 (m, 2H), 4.36

(t, J = 7.3 Hz, 2H), 2.48 – 2.36 (m, 4H), 1.69 (t, J = 7.3 Hz, 2H), 1.61 – 1.55 (m, 2H), 1.04 (t, J = 7.3 Hz, 3H), 0.96 (s, 6H). **^{13}C NMR (100 MHz, CDCl$_3$):** δ = 211.9, 166.8, 133.0, 130.4, 129.6, 128.5, 62.3, 39.7, 37.5, 36.1, 35.8, 32.0, 27.2, 8.0. **HRMS (ESI, m/z):** calcd for $C_{17}H_{24}NaO_3$ [M+Na]$^+$: 299.1623, found: 299.1620.

5-cyano-3,3-dimethylpentyl benzoate 5c

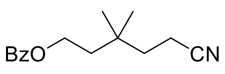

5c

Following general procedure, **5c** (23.0 mg, 0.094 mmol) was obtained as colorless liquid in 47% yield by preparative TLC on silica gel (petroleum ether/ethyl acetate = 20 : 1).

^{1}H NMR (400 MHz, CDCl$_3$): δ = 8.03 – 8.01 (m, 2H), 7.59 – 7.54 (m, 1H), 7.47 – 7.42 (m, 2H), 4.37 (t, J = 7.1 Hz, 2H), 2.37 – 2.32 (m, 2H), 1.75 – 1.70 (m, 4H), 1.01 (s, 6H). **^{13}C NMR (100 MHz, CDCl$_3$):** δ = 166.7, 133.2, 130.2, 129.6, 128.6, 120.4, 61.8, 39.3, 37.8, 32.5, 26.7, 12.5. **HRMS (ESI, m/z):** calcd for $C_{15}H_{19}NNaO_2$ [M+Na]$^+$: 268.1313, found: 268.1310.

3,3-dimethyl-5-(phenylsulfonyl)pentyl benzoate 5d

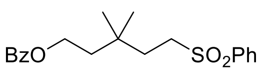

5d

Following general procedure, **5d** (23.8 mg, 0.066 mmol) was obtained as colorless liquid in 33% yield by preparative TLC on silica gel (petroleum ether/ethyl acetate = 2 : 1).

^{1}H NMR (400 MHz, CDCl$_3$): δ = 8.06 – 7.97 (m, 2H), 7.94 – 7.85 (m, 2H), 7.70 – 7.60 (m, 1H), 7.62 – 7.49 (m, 3H), 7.50 – 7.39 (m, 2H), 4.29 (t, J = 7.2 Hz, 2H), 3.16 – 3.06 (m, 2H), 1.75 – 1.69 (m, 2H), 1.66 (t, J = 7.2 Hz, 2H), 0.95 (s, 6H). **^{13}C NMR (100 MHz, CDCl$_3$):** δ = 166.7, 139.1, 133.9, 133.1, 130.2, 129.7, 129.4, 128.5, 128.1, 61.7, 52.4, 39.7, 34.2, 32.1, 26.9. **HRMS (ESI, m/z):** calcd for $C_{20}H_{24}NaO_4S$ [M+Na]$^+$: 383.1293, found: 383.1290.

5-(diethoxyphosphoryl)-3,3-dimethylpentyl benzoate 5e

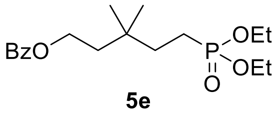

5e

Following general procedure, **5e** (35.6 mg, 0.100 mmol) was obtained as colorless liquid in 50% yield by preparative TLC on silica gel (petroleum ether/ethyl acetate = 2 : 1).

^{1}H NMR (400 MHz, CDCl$_3$): δ = 8.03 – 8.00 (m, 2H), 7.56 – 7.52 (m, 1H), 7.44 – 7.40 (m, 2H), 4.35

(t, *J* = 7.3 Hz, 2H), 4.14 – 4.01 (m, 4H), 1.76 – 1.65 (m, 4H), 1.60 – 1.54 (m, 2H), 1.30 (t, *J* = 7.1 Hz, 6H), 0.96 (s, 6H). Unknown NMR (162 MHz, Chloroform-d) δ 33.89. **^{13}C NMR (100 MHz, CDCl$_3$):** δ = 166.8, 133.0, 130.4, 129.6, 128.5, 62.1, 61.7 (d, *J* = 6.5 Hz), 39.4, 34.4 (d, *J* = 5.0 Hz), 32.4 (d, *J* = 17.1 Hz), 26.7, 20.8 (d, *J* = 141.6 Hz), 16.6 (d, *J* = 6.0 Hz). **HRMS (ESI, m/z):** calcd for $C_{18}H_{29}NaO_5P$ $[M+Na]^+$: 379.1650, found: 379.1648.

Supplementary Discussion

Mechanistic study

a) D-labelling experiment

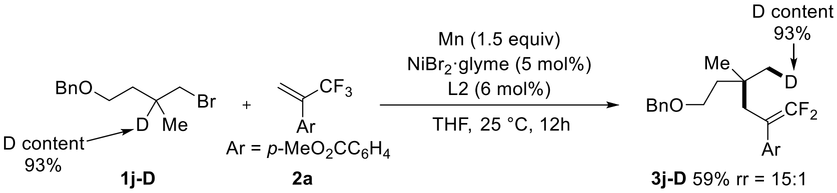

To an oven-dried 10 mL Schlenk tube equipped with a magnetic stirring bar was added $NiBr_2$·glyme (3.1 mg, 0.01 mmol, 5.0 mol%), **L2** (2.5 mg, 0.012 mmol, 6.0 mol%), Mn powder (16.5 mg, 0.3 mmol, 1.5 equiv). After tube was evacuated and refilled with nitrogen (3 times), freshly distilled THF (1.0 mL), compound **1j-D** (154.9 mg, 0.6 mmol, 3.0 equiv), compound **2a** (46.0 mg, 0.2 mmol, 1.0 equiv) was added under nitrogen atmosphere. The tube was capped and stirred at 25 ℃ for 12 h. The reaction mixture was diluted with H_2O (5 mL) and extracted with ethyl acetate (3 × 5 mL). The combined organic layers were washed with water (10 mL), brine (10 mL), dried over anhydrous Na_2SO_4, filtered and evaporated to dryness under reduced pressure. The crude residue was purified by preparative TLC on silica gel (petroleum ether/ethyl acetate = 20 : 1) to afford the compound **3j-D** in 59% yield as colorless liquid. **^{1}H NMR (400 MHz, $CDCl_3$):** δ = 8.05 – 7.96 (m, 2H), 7.42 – 7.25 (m, 7H), 4.42 (s, 2H), 3.92 (s, 3H), 3.45 (t, J = 7.2 Hz, 2H), 2.41 (t, J = 2.4 Hz, 2H), 1.53 (t, J = 7.2 Hz, 2H), 0.77 (s, 3H), 0.76 (s, 2H).

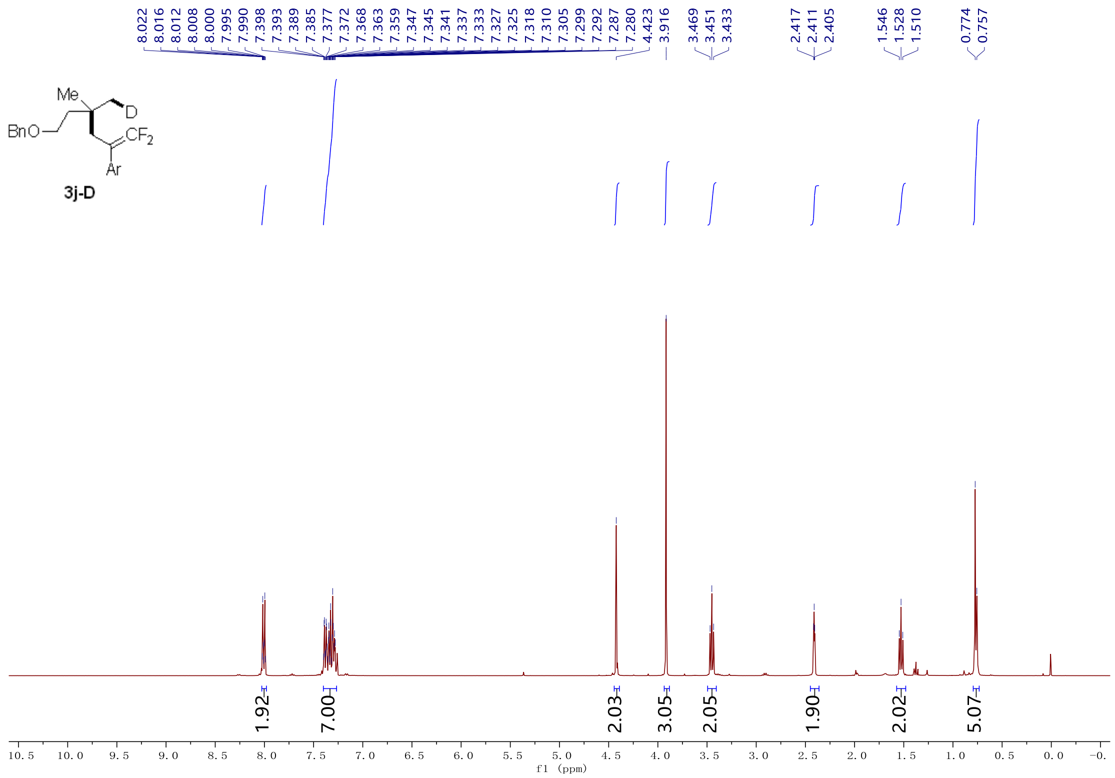

Supplementary Figure 3. ^{1}H NMR spectrum of compound **3j-D**

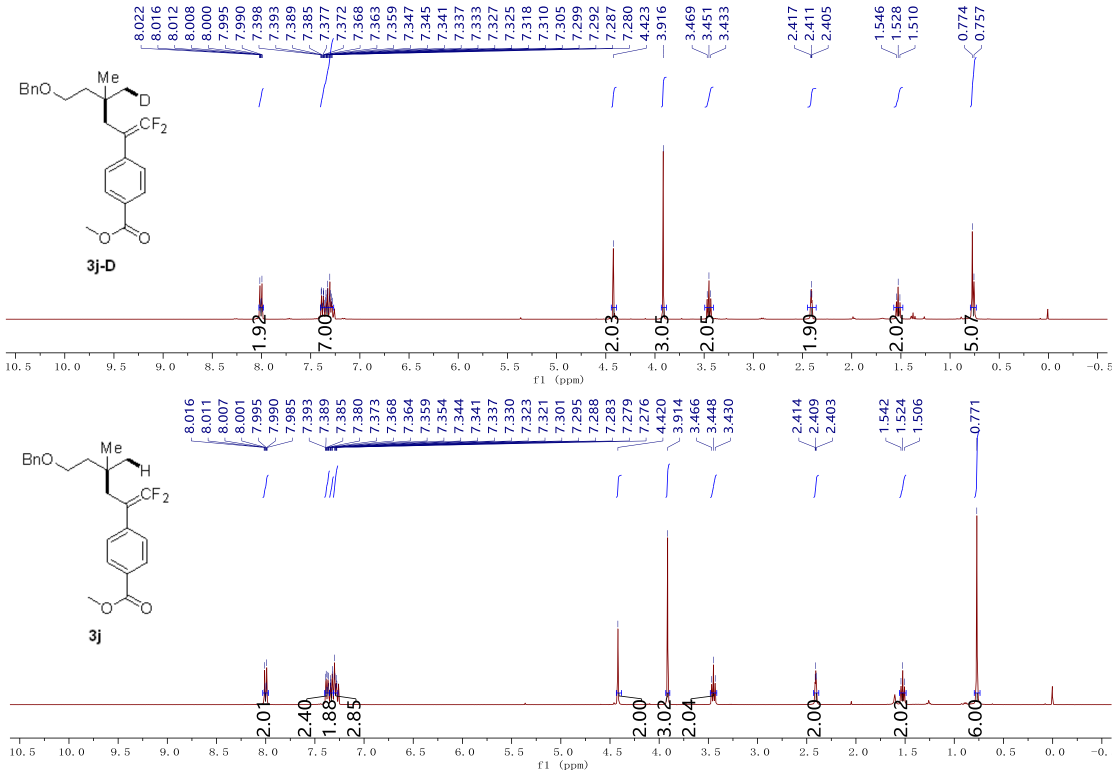

Supplementary Figure 4. Comparison of ^{1}H NMR of **3j-D** and **3j**

Preparation of compound 1j-D

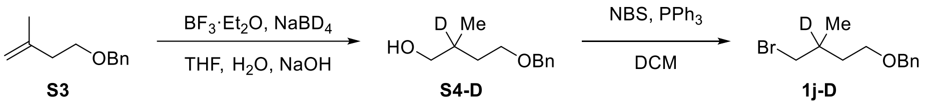

1) To an oven-dried 50 mL round-bottom flask was charged $NaBD_4$ (0.0840 g, 2 mmol, 0.4 equiv). The flask was sealed, evacuated and refilled with nitrogen (3 times). After **S3** (0.88 g, 5 mmol, 1.0 equiv) and anhydrous THF (2 mL) were added, the resulting solution was cooled to 0 °C and stirred for 5 min. $BF_3{\cdot}Et_2O$ (0.39 g, 2.75 mmol, 0.55 equiv) was then added dropwise. The reaction mixture was allowed to stir at room temperature for 3 h. After quenching by adding aqueous solution of NaOH (3 mol/L, 5 mL) followed by H_2O_2 (36%, 5 mL), the mixture was extracted with ethyl acetate (3 × 10 mL). The combined organic layers were washed with water (10 mL), brine (10 mL) and dried over Na_2SO_4. After solvent was removed under reduced pressure, the crude residue was purified by column chromatography on silica gel (petroleum ether/ethyl acetate = 5 : 1) to afford compound **S4-D** in 39% yield as colorless liquid. **^{1}H NMR (400 MHz, $CDCl_3$):** δ = 7.38 – 7.27 (m, 5H), 4.52 (s, 2H), 3.65 – 3.39 (m, 4H), 2.51 (s, 1H), 1.72 – 1.65 (m, 1H), 1.64 – 1.51 (m, 1H), 0.91 (s, 3H).

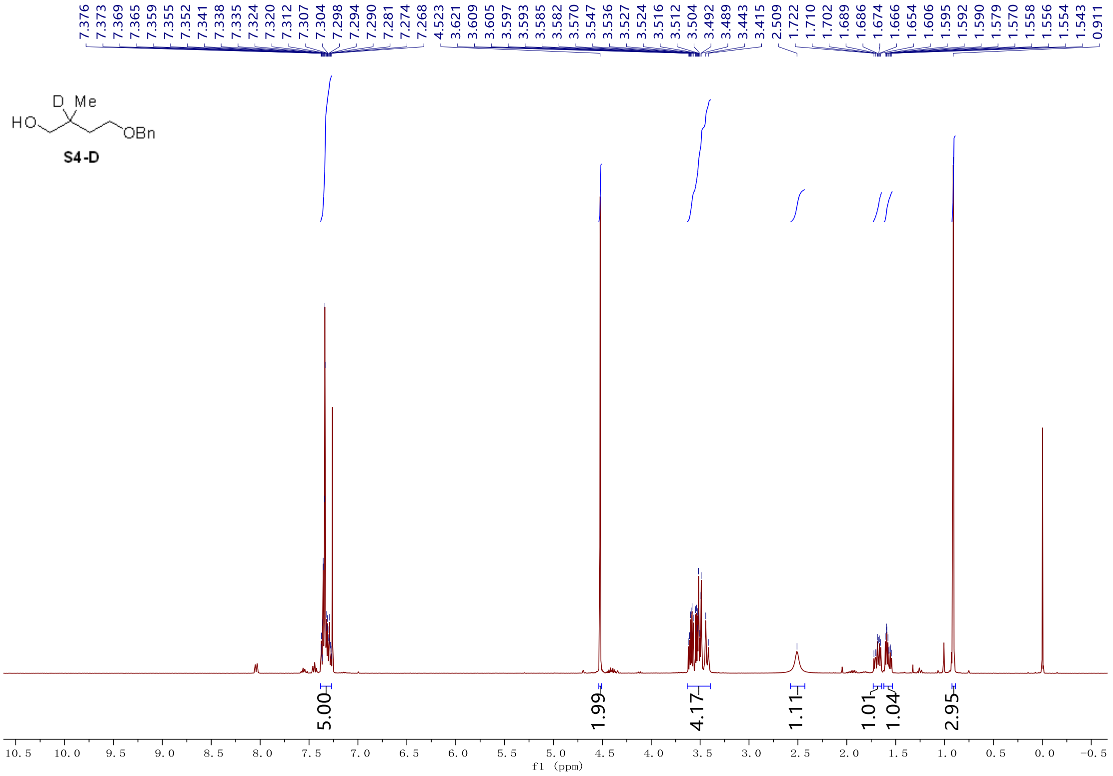

Supplementary Figure 5. ^{1}H NMR spectrum of compound **S4-D**

2) An oven-dried 25 mL round-bottom flask equipped with magnetic stirring bar was evacuated and refilled with nitrogen (3 times). To the flask were charged **S4-D** (0.39 g, 2.0 mmol, 1.0 equiv), PPh_3 (0.63

g, 2.4 mmol, 1.2 equiv) and DCM (5 ml) under nitrogen atmosphere. The resulting clear solution was cooled to 0 °C and stirred for 5 min. NBS (0.43 g, 2.4 mmol, 1.2 equiv) was then added by portions at 0 ℃ over 5 min. The reaction was allowed to stir at room temperature for 3 h. Then the mixture was quenched by adding H_2O (10 mL) and extracted with DCM (3 × 10 mL). The combined organic layers were dried with anhydrous Na_2SO_4, filtered and evaporated to dryness under reduced pressure. The crude residue was purified by column chromatography on silica gel (petroleum ether/ethyl acetate = 20 : 1) to afford compound **1j-D** in 78% yield as colorless liquid. **^{1}H NMR (400 MHz, $CDCl_3$):** δ = 7.38 – 7.27 (m, 5H), 4.51 (s, 2H), 3.55 – 3.50 (m, 2H), 3.48 – 3.32 (m, 2H), 1.86 – 1.76 (m, 1H), 1.58 – 1.52 (m, 1H), 1.03 (s, 3H).

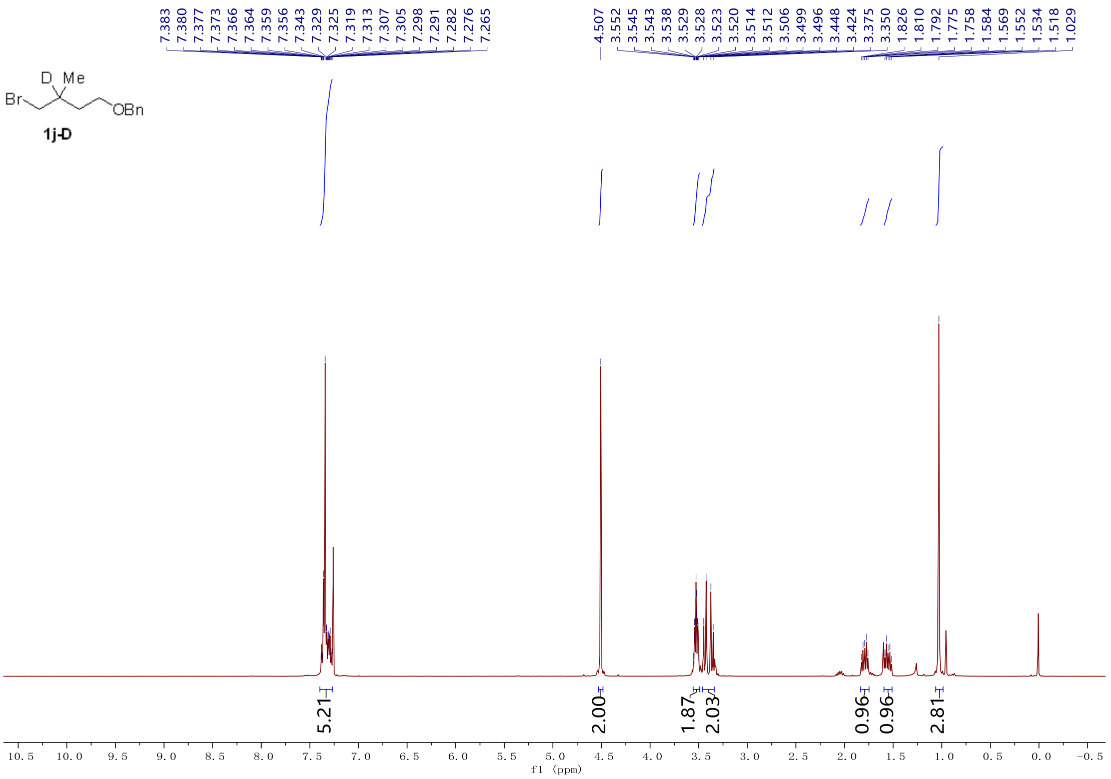

Supplementary Figure 6. ^{1}H NMR spectrum of compound **1j-D**

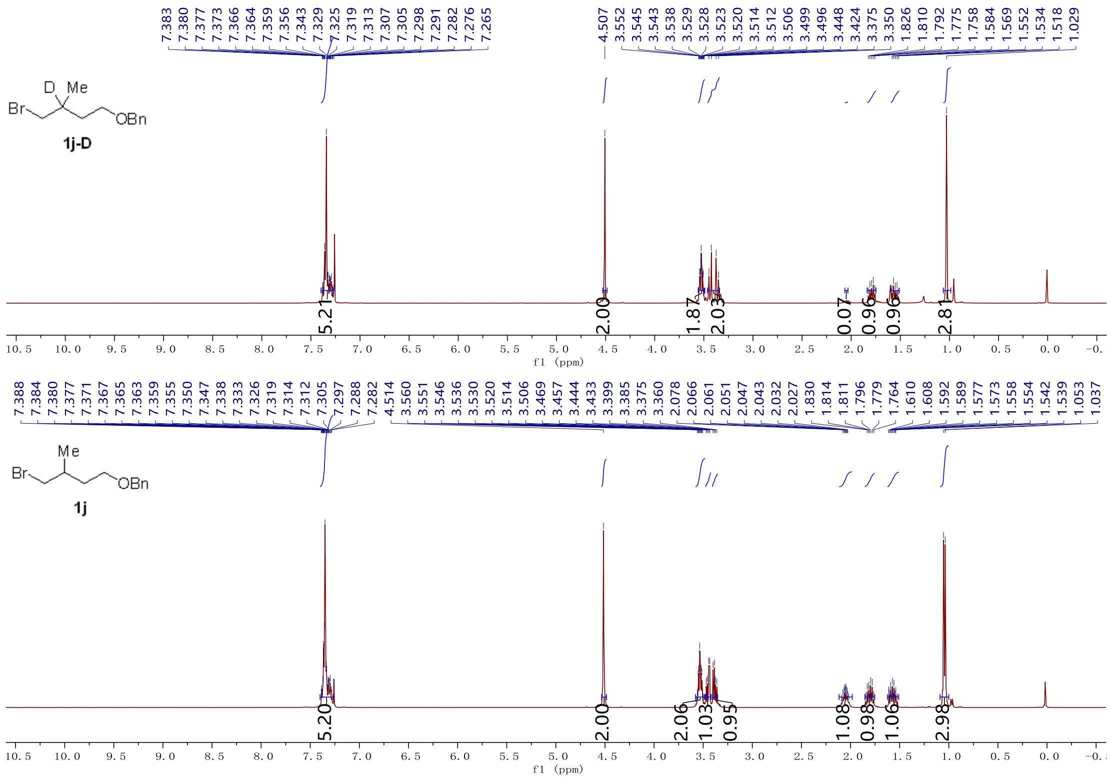

Supplementary Figure 7. Comparison of ^{1}H NMR of **1j-D** and **1j**

b) radical trapping experiments

Eq. 1

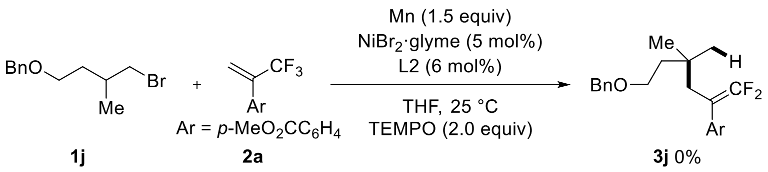

To an oven-dried 10 mL Schlenk tube equipped with a magnetic stirring bar was added $NiBr_2$·glyme (3.1 mg, 0.01 mmol, 5.0 mol%), L2 (2.5 mg, 0.012 mmol, 6.0 mol%), Mn powder (16.5 mg, 0.3 mmol, 1.5 equiv), TEMPO (62.5 mg, 0.4 mmol, 2.0 equiv). After tube was evacuated and refilled with nitrogen (3 times), THF (1.0 mL), compound **1j** (154.9 mg, 0.6 mmol, 3.0 equiv), compound **2a** (46.0 mg, 0.2 mmol, 1.0 equiv) was added under nitrogen atmosphere. The tube was capped and stirred at 25 ℃ for 12 h. The reaction mixture was diluted with H_2O (5 mL) and extracted with ethyl acetate (3 × 5 mL). The combined organic layers were washed with water (10 mL), brine (10 mL), dried over anhydrous Na_2SO_4, filtered and evaporated to dryness under reduced pressure. The crude residue was analyzed by ^{19}F NMR which indicated no desired product formed and 97% of **2a** remained.

Eq. 2

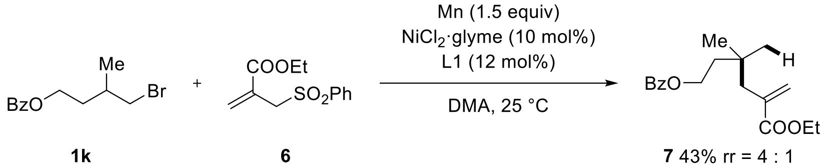

To an oven-dried 10 mL Schlenk tube equipped with a magnetic stirring bar was added $NiCl_2$·glyme (4.4 mg, 0.02 mmol, 10 mol%), L1 (4.4 mg, 0.024 mmol, 12 mol%), Mn powder (16.5 mg, 0.3 mmol, 1.5 equiv). After tube was evacuated and refilled with nitrogen (3 times), DMA (1.0 mL), compound **1k** (108.5 mg, 0.4 mmol, 2.0 equiv), compound **6** (50.9 mg, 0.2 mmol, 1.0 equiv) was added under nitrogen atmosphere. The tube was capped and stirred at 25 ℃ for 12 h. The reaction mixture was diluted with H_2O (5 mL) and extracted with ethyl acetate (3 × 5 mL). The combined organic layers were washed with water (10 mL), brine (10 mL), dried over anhydrous Na_2SO_4, filtered and evaporated to dryness under reduced pressure. The crude residue was purified by preparative TLC on silica gel to afford the compound **7** in 43% yield as colorless liquid. **^1H NMR (400 MHz, $CDCl_3$): ^1H NMR (400 MHz, $CDCl_3$):** δ = 8.04 – 8.01 (m, 2H), 7.57 – 7.53 (m, 1H), 7.45 – 7.42 (m, 2H), 6.24 (s, 1H), 5.51 (s, 1H), 4.41 (t, *J* = 7.3 Hz, 2H), 4.20 (q, *J* = 7.1 Hz, 2H), 2.39 (s, 2H), 1.72 (t, *J* = 7.2 Hz, 2H), 1.29 (t, *J* = 7.1 Hz, 3H), 0.95 (s, 6H).

^{13}C NMR (100 MHz, $CDCl_3$): δ = 168.2, 166.8, 138.3, 133.0, 129.6, 128.5, 127.9, 124.46, 62.3, 60.9, 43.2, 40.0, 33.5, 26.7, 14.3. **HRMS (ESI, m/z):** calcd for $C_{18}H_{24}NaO_4$ $[M+Na]^+$: 327.1572, found: 327.1579.

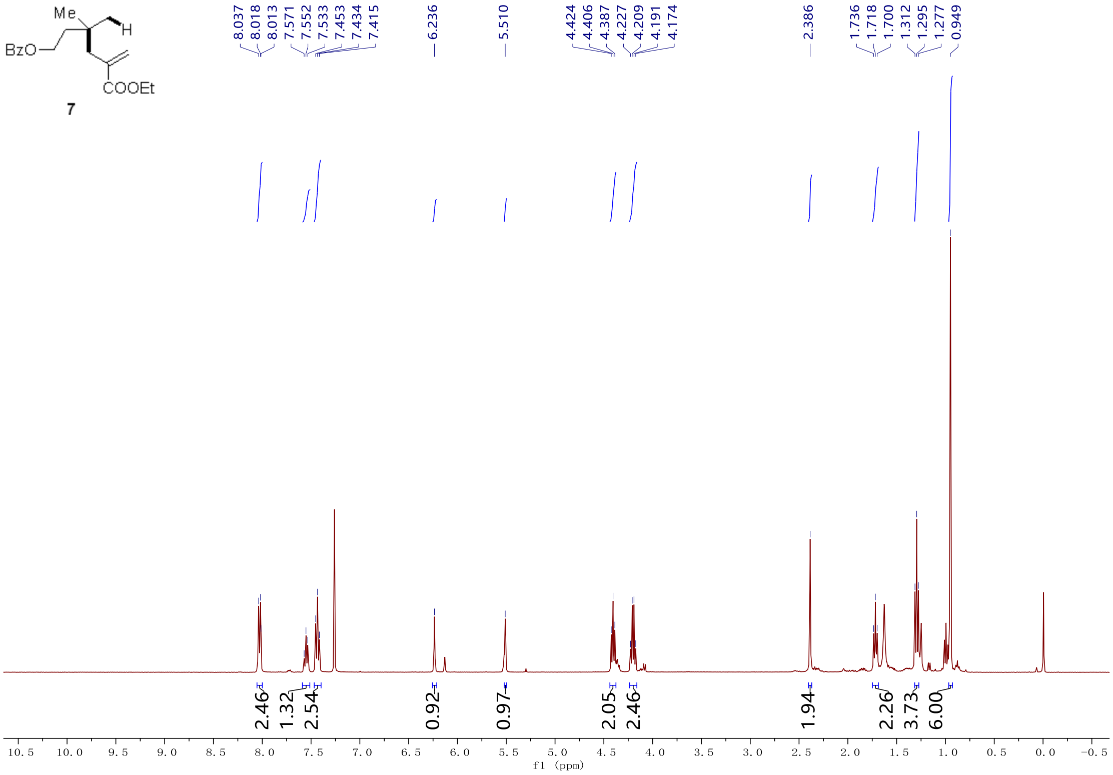

Supplementary Figure 8. ^{1}H NMR spectrum of compound **7**

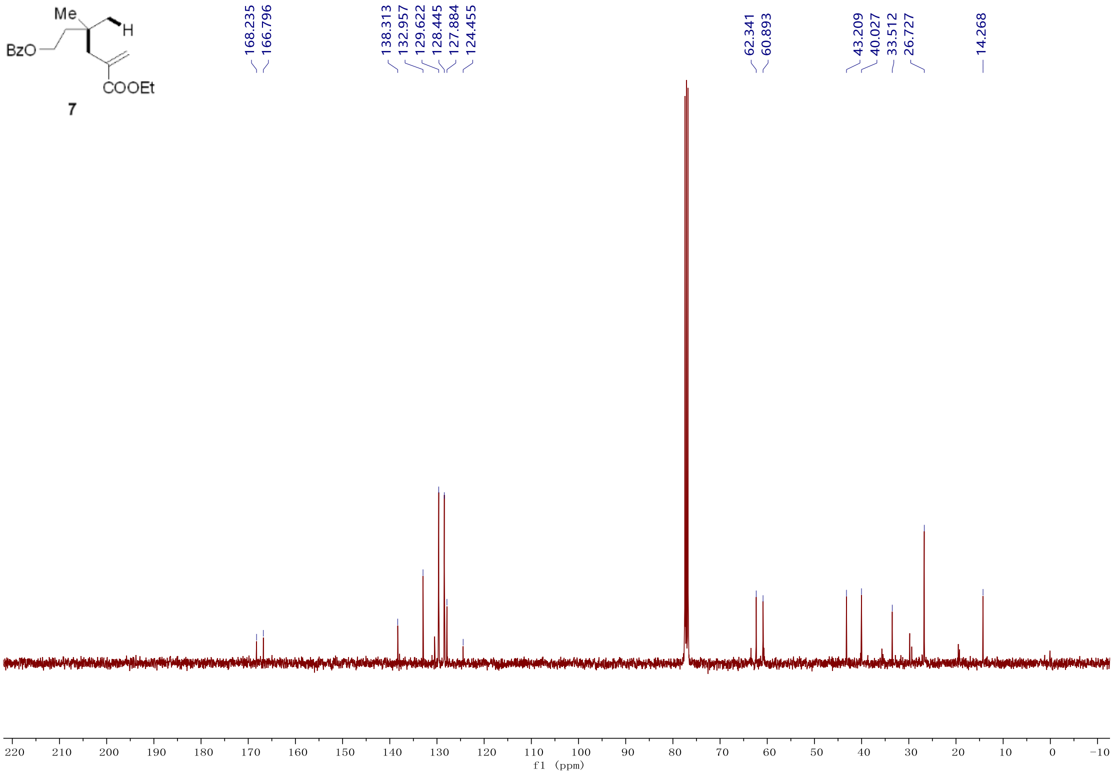

Supplementary Figure 9. ^{13}C NMR spectrum of compound **7**

c) ring opening experiment

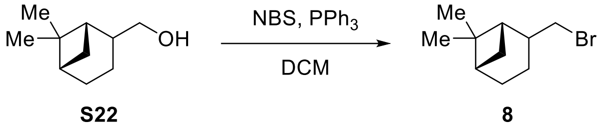

An oven-dried 50 mL round-bottom flask equiped with magnetic stirring bar was evacuated and refilled with nitrogen (3 times). To the flask was charged the alcohol **S22**[14] (0.77 g, 5.0 mmol, 1.0 equiv), PPh_3 (1.57 g, 6.0 mmol, 1.2 equiv) and DCM (10 mL) under nitrogen atmosphere. The resulting clear solution was cooled to 0 °C and stirred for 5 min. NBS (1.07 g, 6 mmol, 1.2 equiv) was then added by portions at 0 °C over 5 min against a nitrogen flow. The reaction was allowed to warm to room temperature and stirred for 3 hours. Then the reaction mixture was quenched by adding H_2O (10 mL) followed by extraction with DCM (3 × 10 mL). The combined organic layers were dried with anhydrous Na_2SO_4, filtered, and evaporated to dryness under reduced pressure. The crude residue was purified by column chromatography on silica gel (petroleum ether) to afford compound **8** (0.80 g, 3.7 mmol) in 74% yield as colorless liquid. **^1H NMR (400 MHz, $CDCl_3$): ^1H NMR (400 MHz, $CDCl_3$):** δ = 3.48 – 3.34 (m, 2H), 2.48 – 2.41 (m, 1H), 2.40 – 2.33 (m, 1H), 2.09 – 2.02 (m, 2H), 1.95 – 1.84 (m, 3H), 1.54 – 1.46 (m, 1H), 1.18 (s, 3H), 0.97 (s, 3H), 0.93 – 0.86 (m, 1H). **^{13}C NMR (100 MHz, $CDCl_3$):** δ = 45.2, 44.0, 41.2, 40.1, 38.6, 33.1, 27.9, 25.9, 23.3, 21.6. **MS (EI, m/z):** calcd for $C_{10}H_{17}Br$ $[M]^+$: 216.05, found: 216.1.

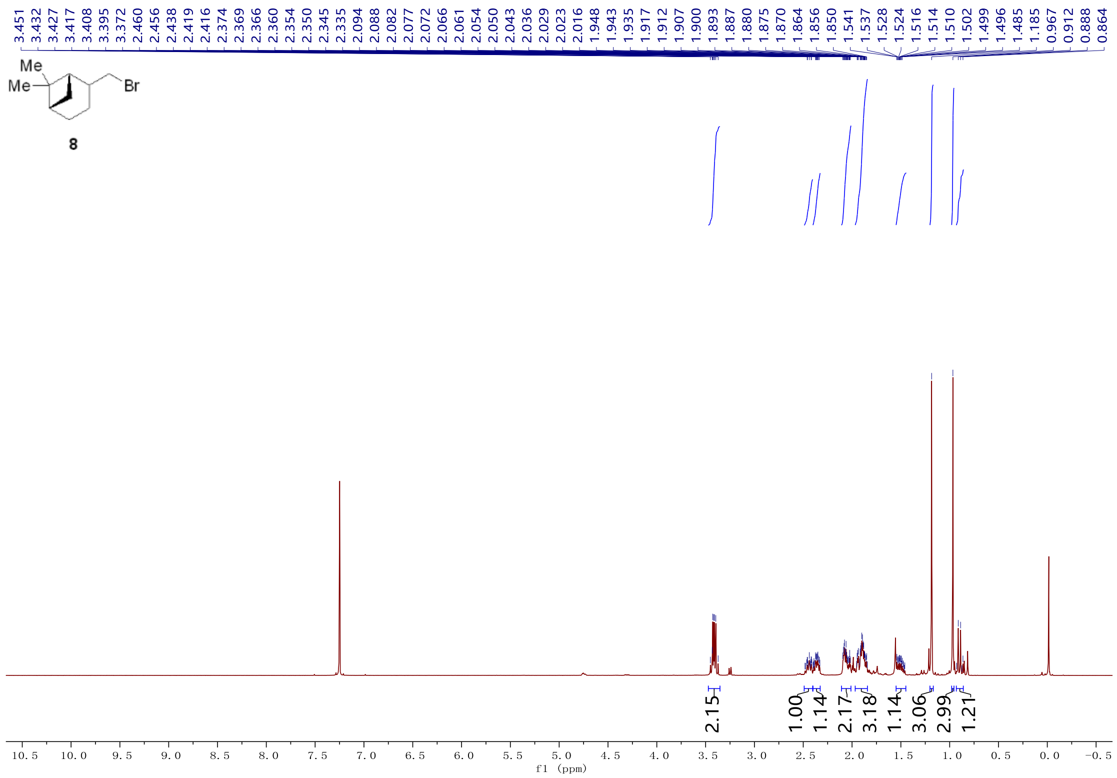

Supplementary Figure 10. ^{1}H NMR spectrum of compound **8**

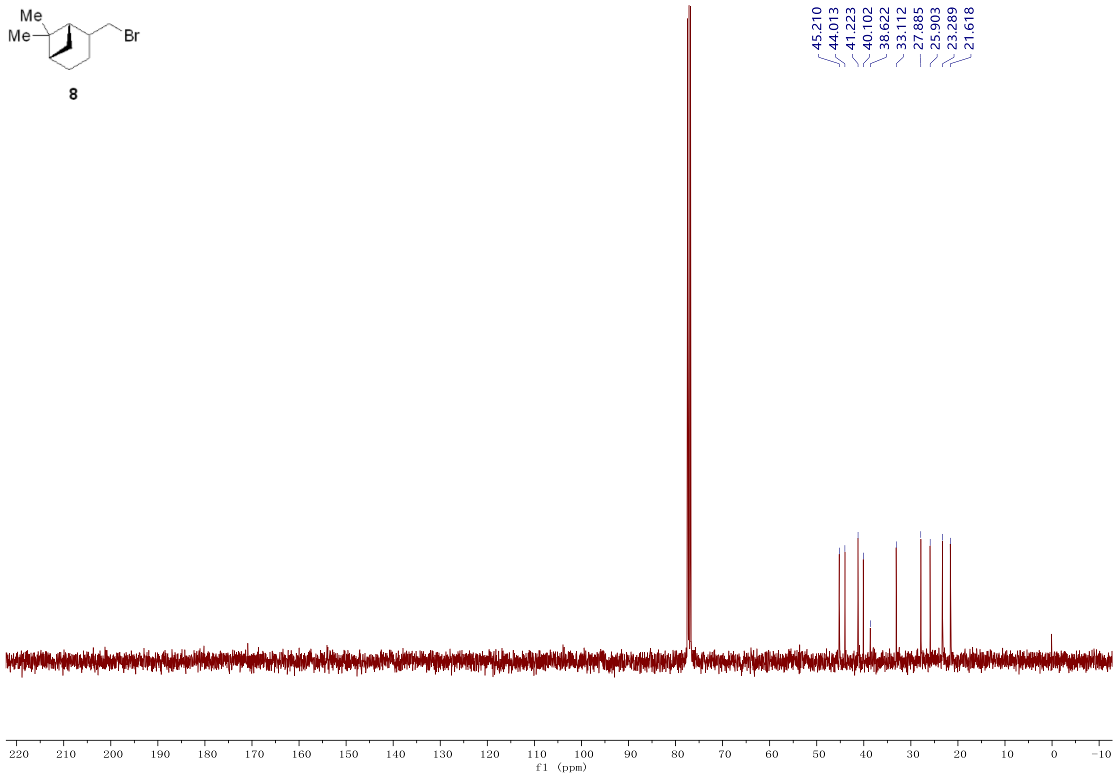

Supplementary Figure 11. ^{13}C NMR spectrum of compound **8**

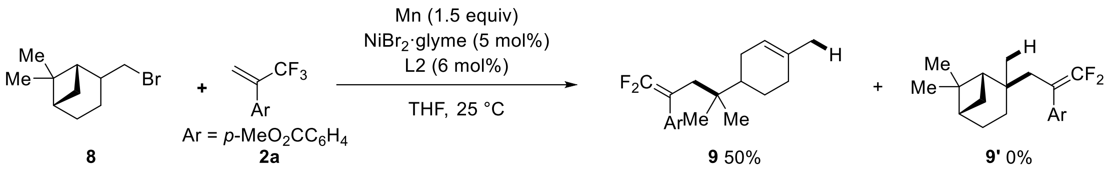

To an oven-dried Schlenk tube equipped with a magnetic stir bar was added NiBr$_2$·glyme (3.1 mg, 0.01 mmol, 5.0 mol%), L2 (2.5 mg, 0.012 mmol, 6.0 mol%), Mn powder (16.5 mg, 0.3 mmol, 1.5 equiv). After tube was evacuated and refilled with nitrogen (3 times), THF (1.0 mL), compound **8** (130.3 mg, 0.6 mmol, 3.0 equiv), compound **2a** (46.0 mg, 0.2 mmol, 1.0 equiv) was added under nitrogen atmosphere. The tube was capped and stirred at 25 ℃ for 12 h. The reaction mixture was diluted with H_2O (5 mL) and extracted with ethyl acetate (3 × 5 mL). The combined organic layers were washed with water (10 mL), brine (10 mL), dried over anhydrous Na_2SO_4, filtered and evaporated to dryness under reduced pressure. The crude residue was purified by preparative TLC on silica gel to afford the compound **9** in 50% yield as colorless liquid. **^{1}H NMR (400 MHz, CDCl$_3$): ^{1}H NMR (400 MHz, CDCl$_3$):** δ = 8.03 – 7.99 (m, 2H), 7.41 – 7.31 (m, 2H), 5.35- 5.31 (m, 1H), 3.92 (s, 3H), 2.52 – 2.33 (m, 2H), 1.96 – 1.82 (m, 2H), 1.79 – 1.68 (m, 3H), 1.61 (s, 3H), 1.28 – 1.12 (m, 2H), 0.68 (d, J = 6.3 Hz, 6H). **^{19}F NMR (376 MHz, CDCl$_3$):** δ = -87.60 (d, J = 36.5 Hz), -89.80 (d, J = 35.9 Hz). **^{13}C NMR (100 MHz, CDCl$_3$):** δ = 166.9, 154.5 (dd, J = 291.7, 288.9 Hz), 140.9 (dd, J = 4.8, 2.9 Hz), 134.1, 129.7, 128.8, 128.6 (t, J = 2.5 Hz), 121.0, 90.7 (dd, J = 22.2, 12.6 Hz), 52.2, 42.7, 37.4, 37.2, 31.4, 26.6, 25.1, 24.2, 24.0, 23.4.

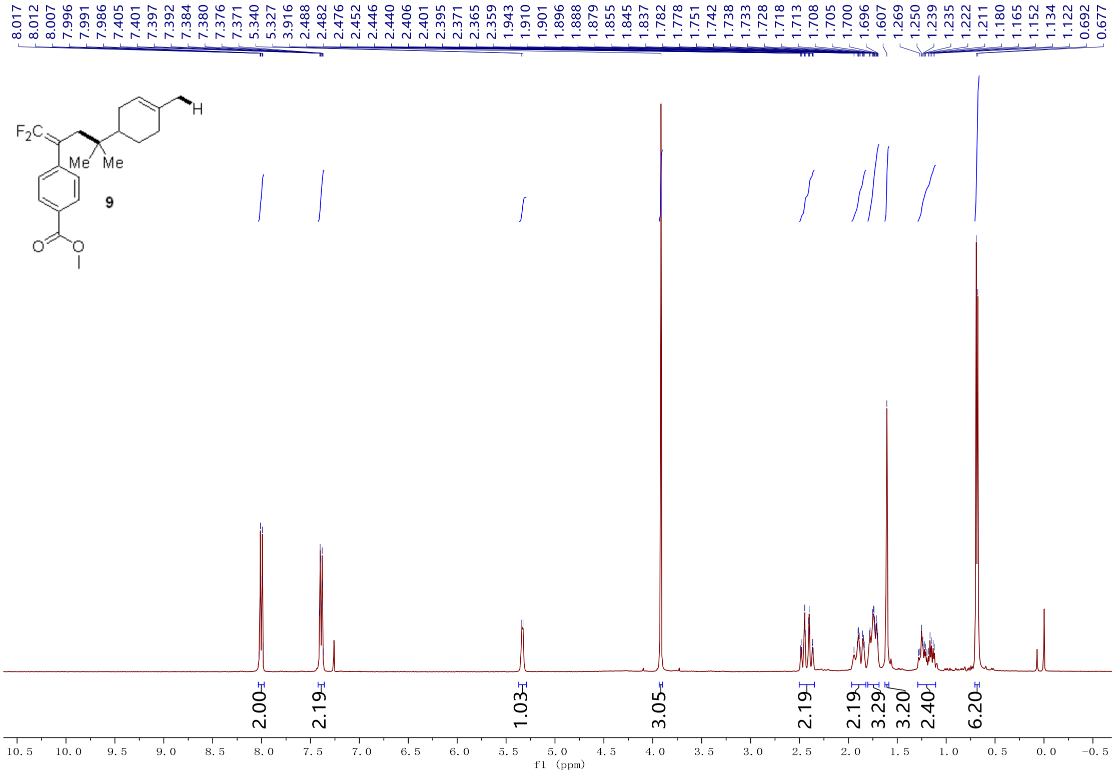

Supplementary Figure 12. ^{1}H NMR spectrum of compound **9**

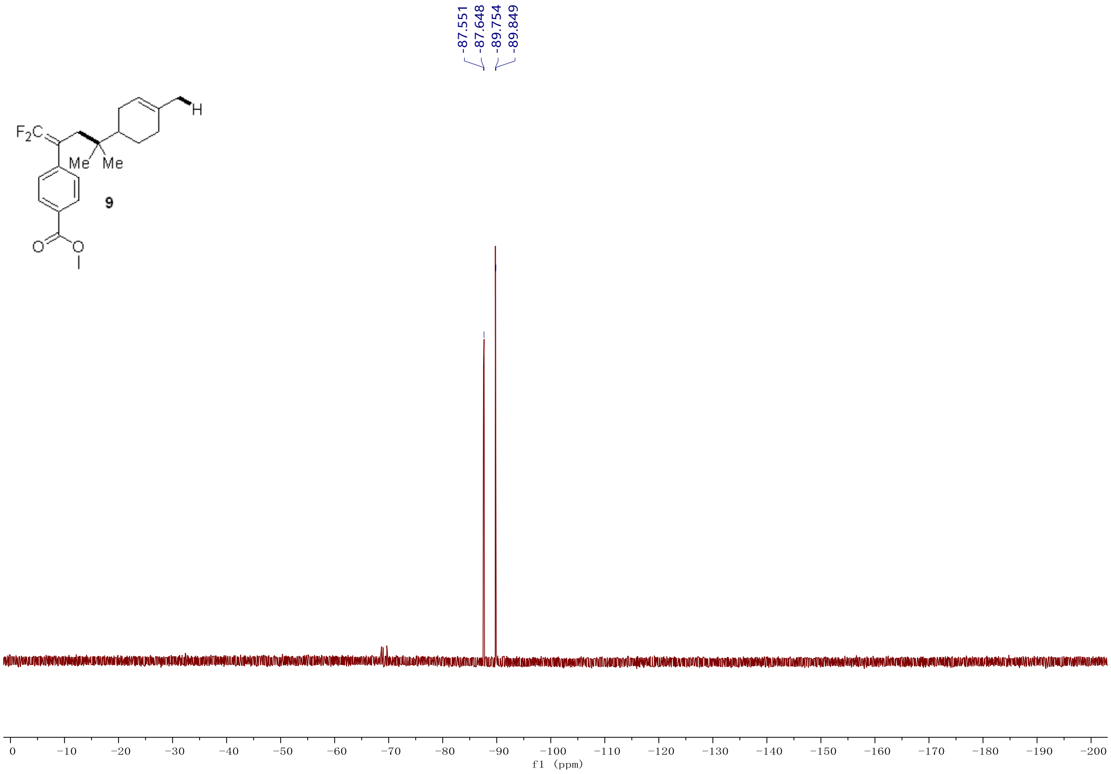

Supplementary Figure 13. ^{19}F NMR spectrum of compound **9**

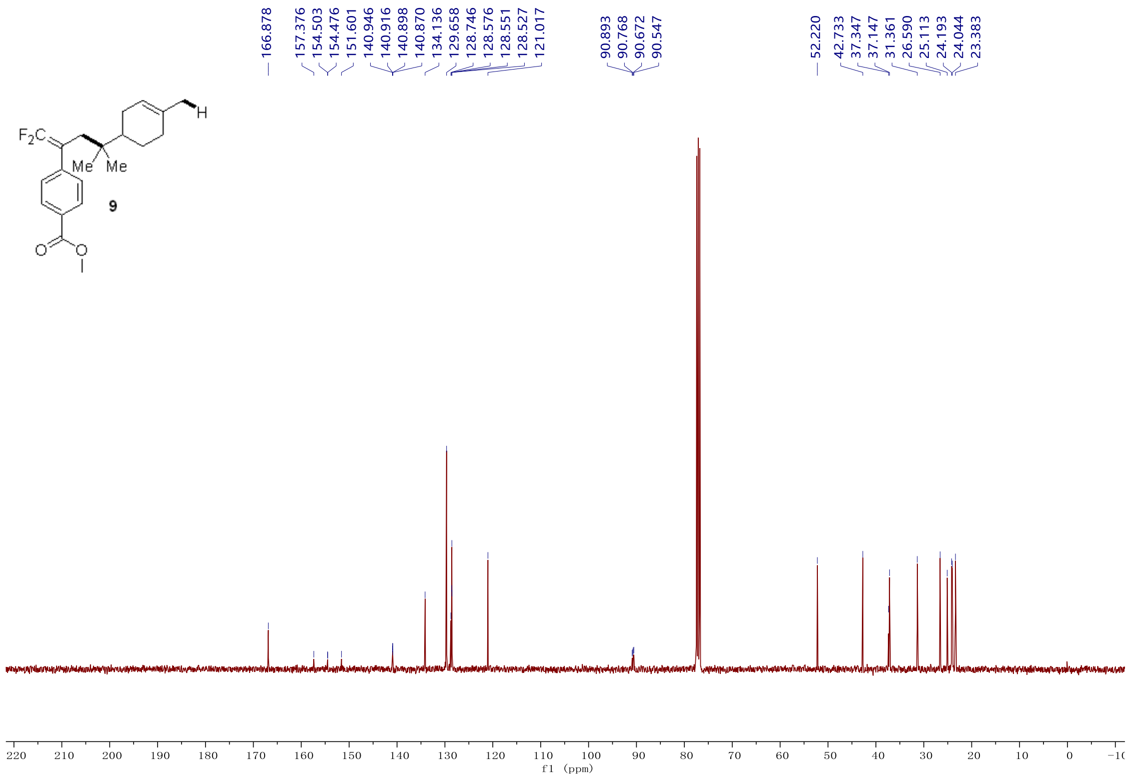

Supplementary Figure 14. ^{13}C NMR spectrum of compound **9**

Spectra Data

Note:

1) GC-MS analysis was performed on an Agilent 7890B gas chromatograph with 5977B GC/MSD mass spectrum using an HP-5 MS column (30 m, 0.25 mm I.D.).

2) GC method: Starts at 50 °C holds the oven at this temperature for 1 minute, then ramp of 15 °C/min till 300 °C and hold the oven at this temperature for 10 minutes.

3) rr refers to regioisomeric ratio, represents the ratio of major product to the sum of all other isomers as determined by crude ^{19}F NMR or GC, all isomers' peaks were confirmed by GC-MS analysis.

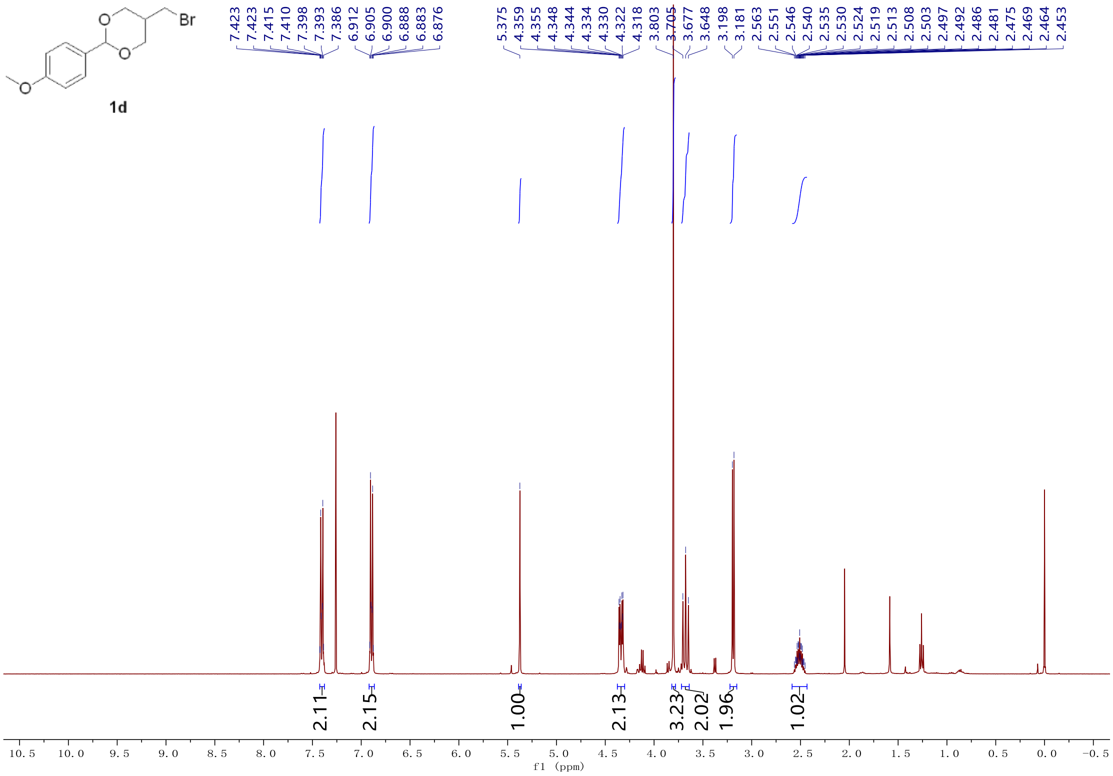

Supplementary Figure 15. ^{1}H NMR spectrum of compound **1d**

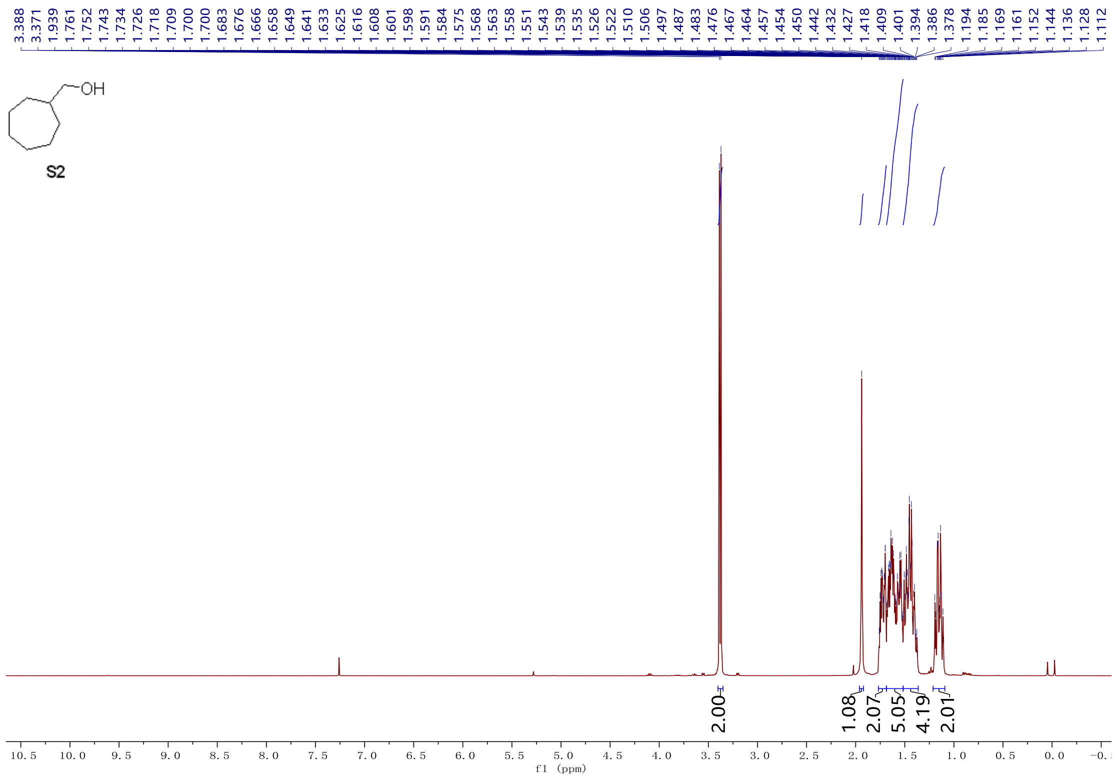

Supplementary Figure 16. ^{1}H NMR spectrum of compound **S2**

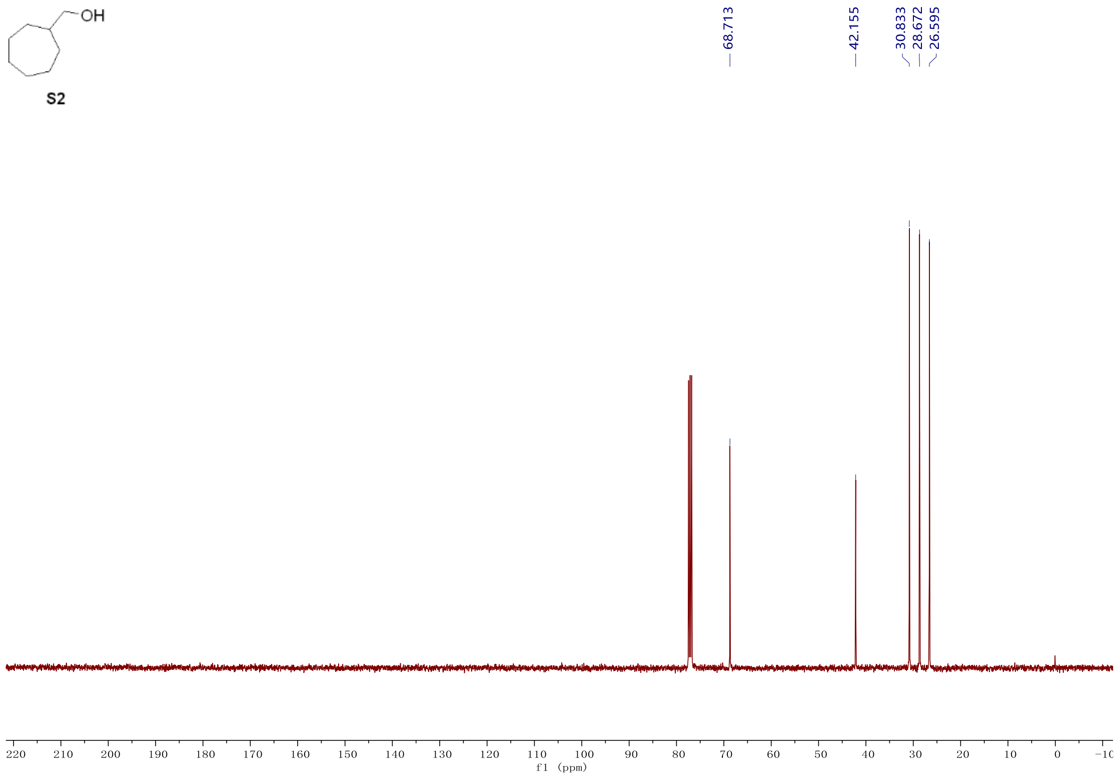

Supplementary Figure 17. ^{13}C NMR spectrum of compound **S2**

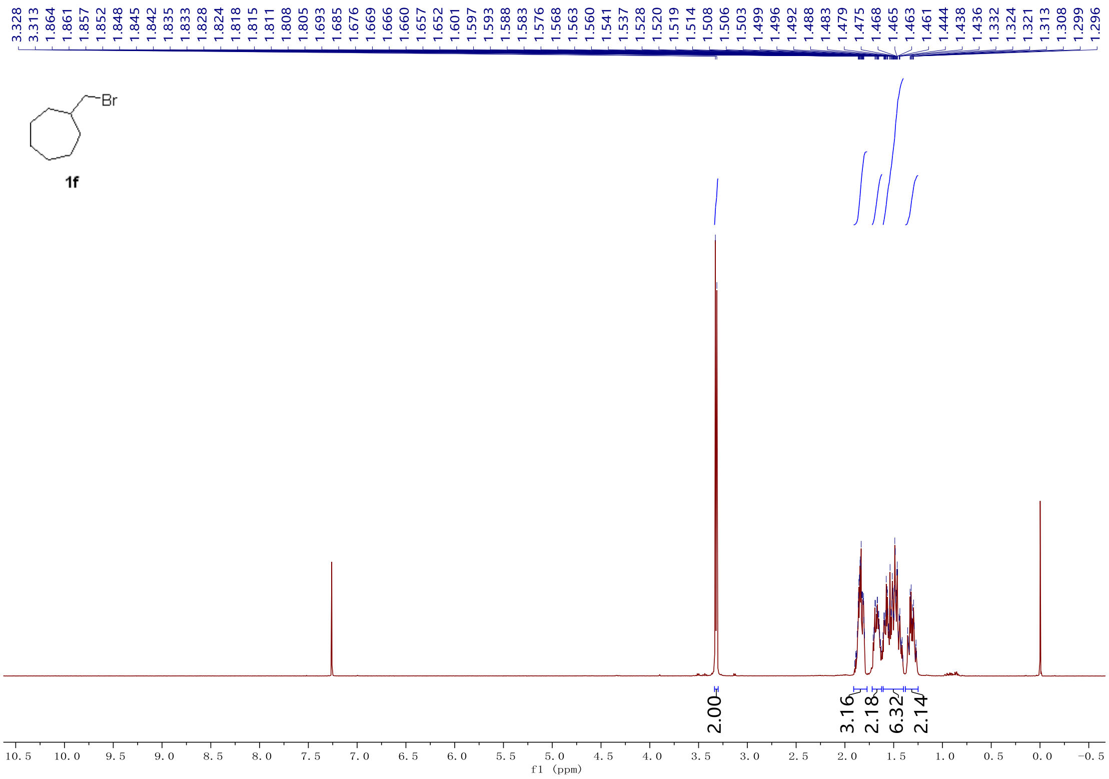

Supplementary Figure 18. ^{1}H NMR spectrum of compound **1f**

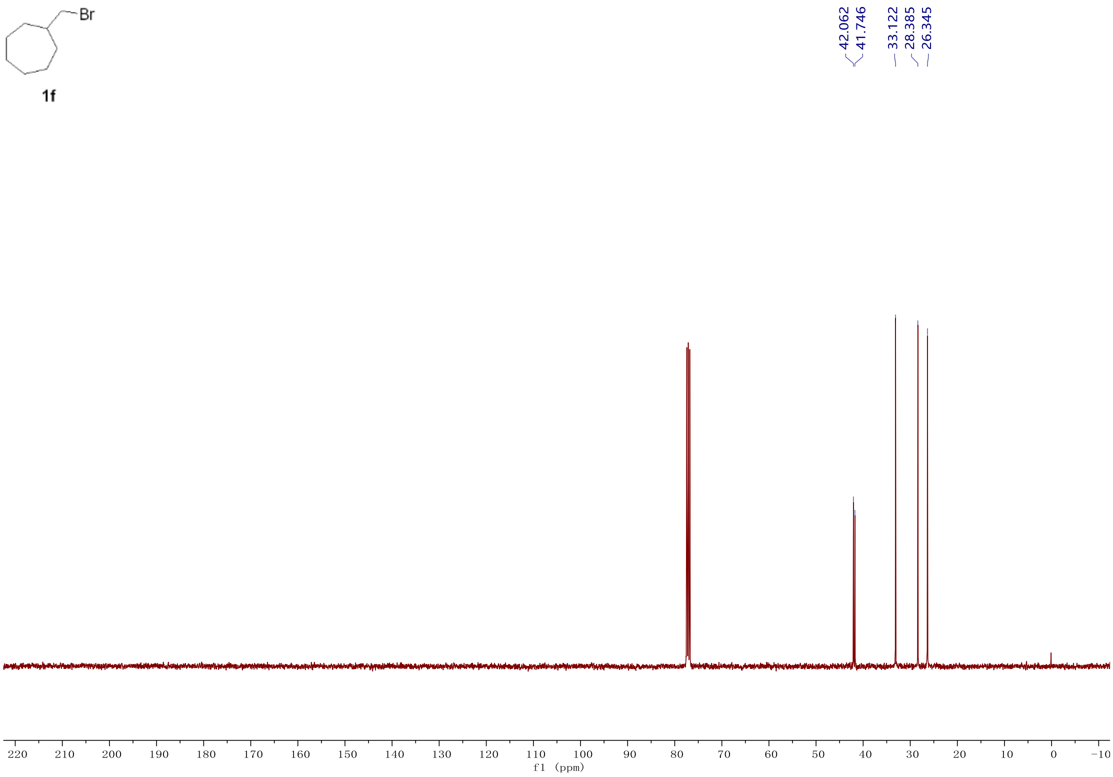

Supplementary Figure 19. ^{13}C NMR spectrum of compound **1f**

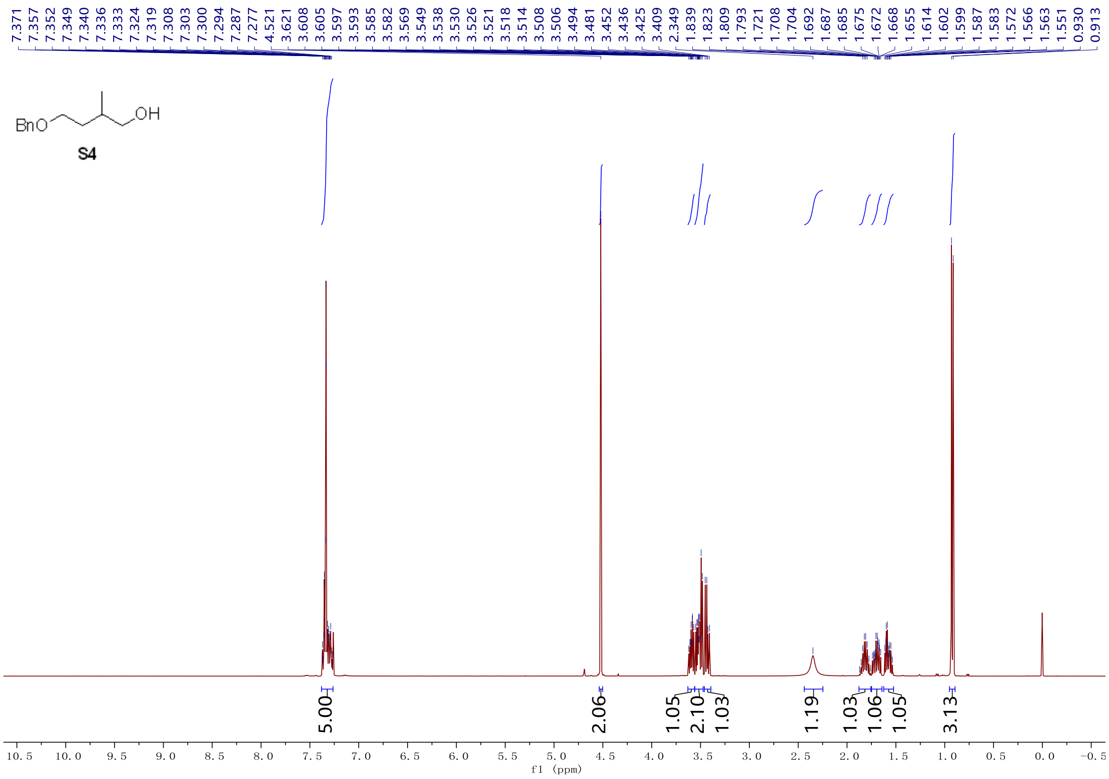

Supplementary Figure 20. ^{1}H NMR spectrum of compound **S4**

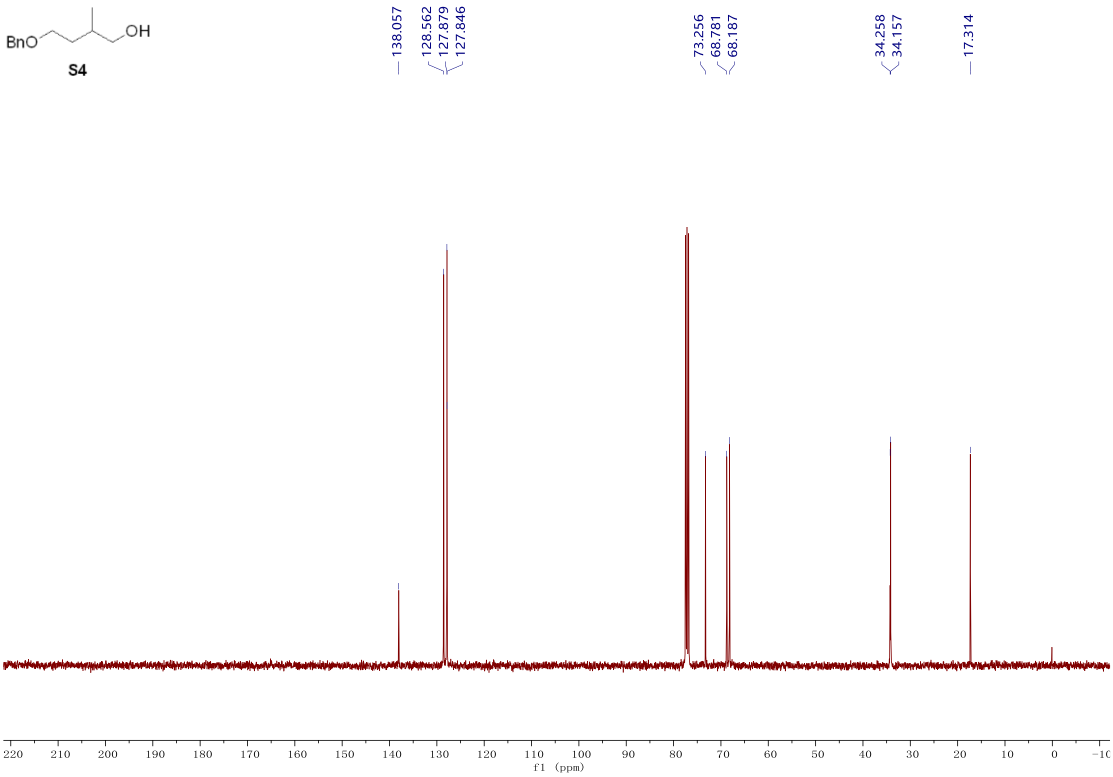

Supplementary Figure 21. ^{13}C NMR spectrum of compound **S4**

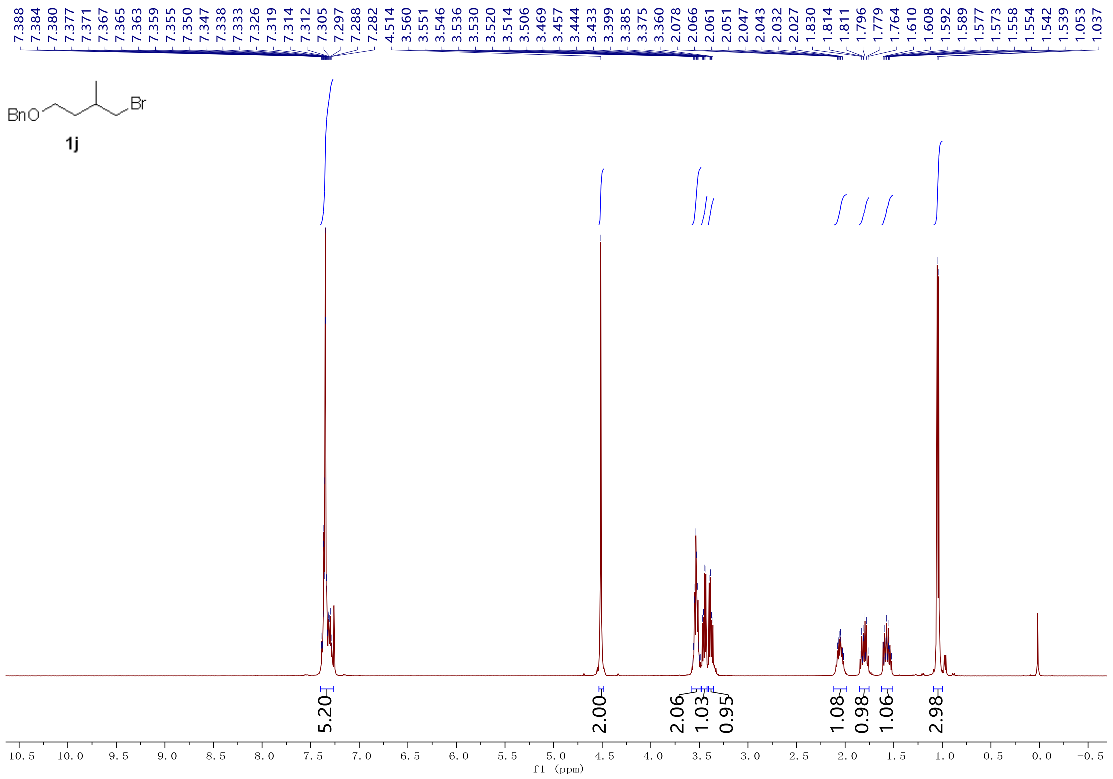

Supplementary Figure 22. ^{1}H NMR spectrum of compound **1j**

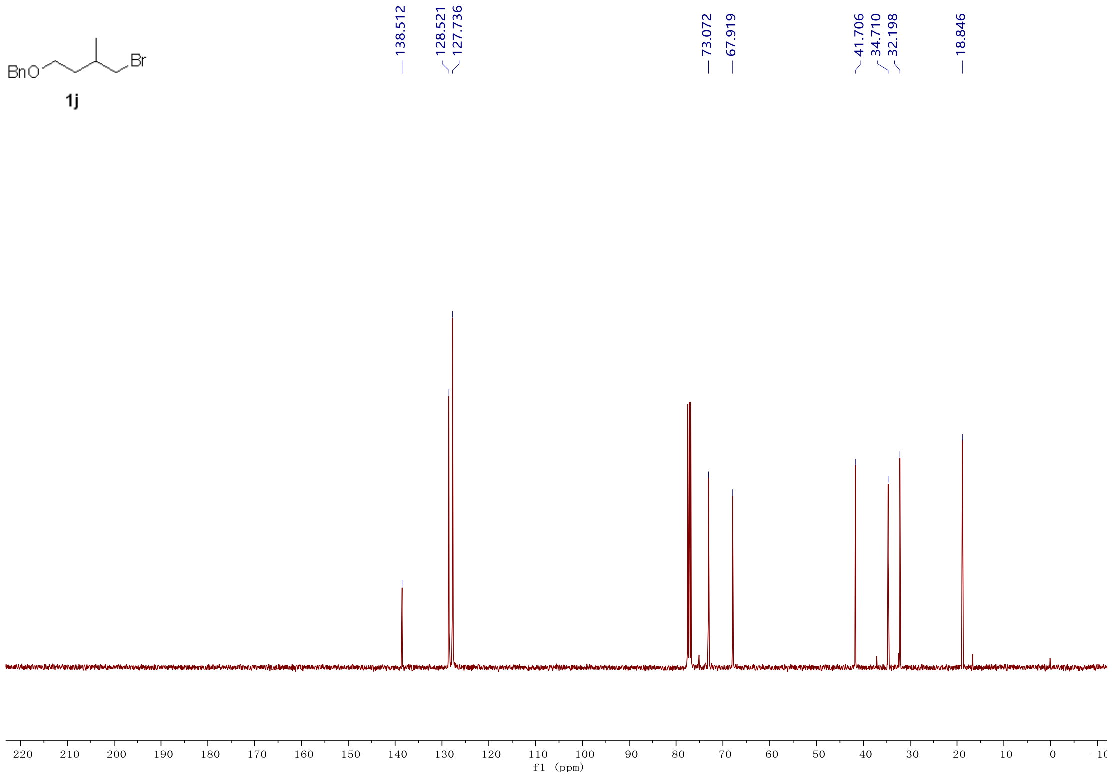

Supplementary Figure 23. ^{13}C NMR spectrum of compound **1j**

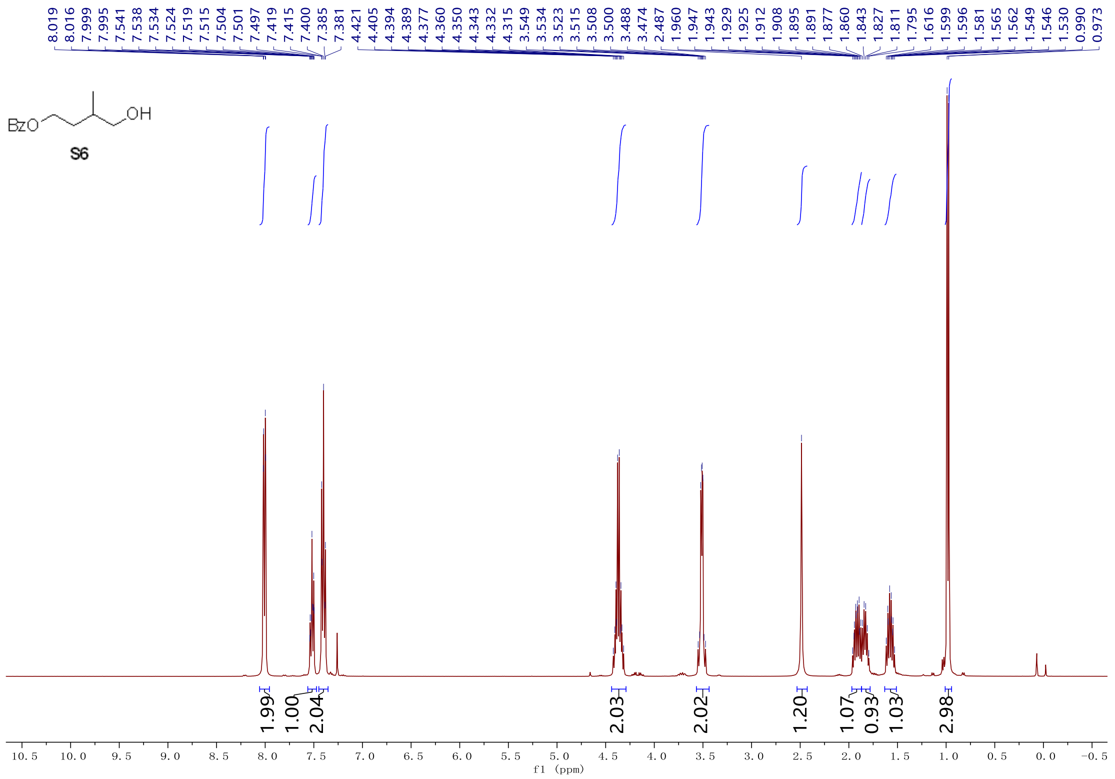

Supplementary Figure 24. ^{1}H NMR spectrum of compound **S6**

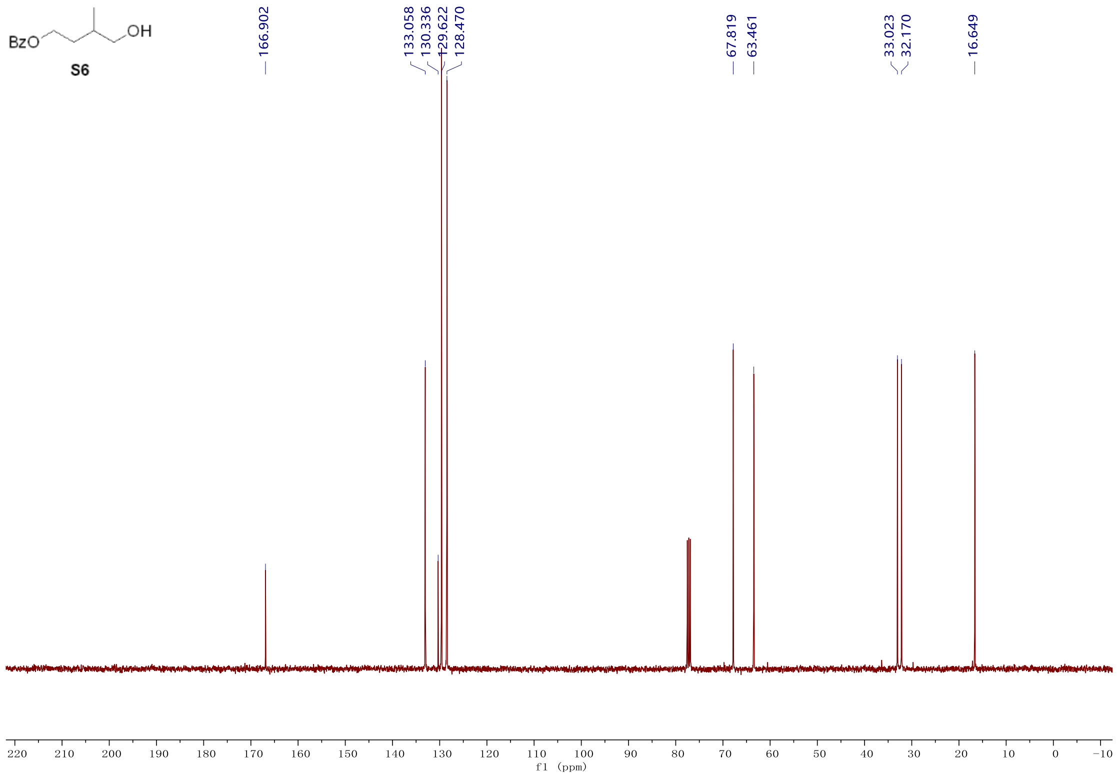

Supplementary Figure 25. ^{13}C NMR spectrum of compound **S6**

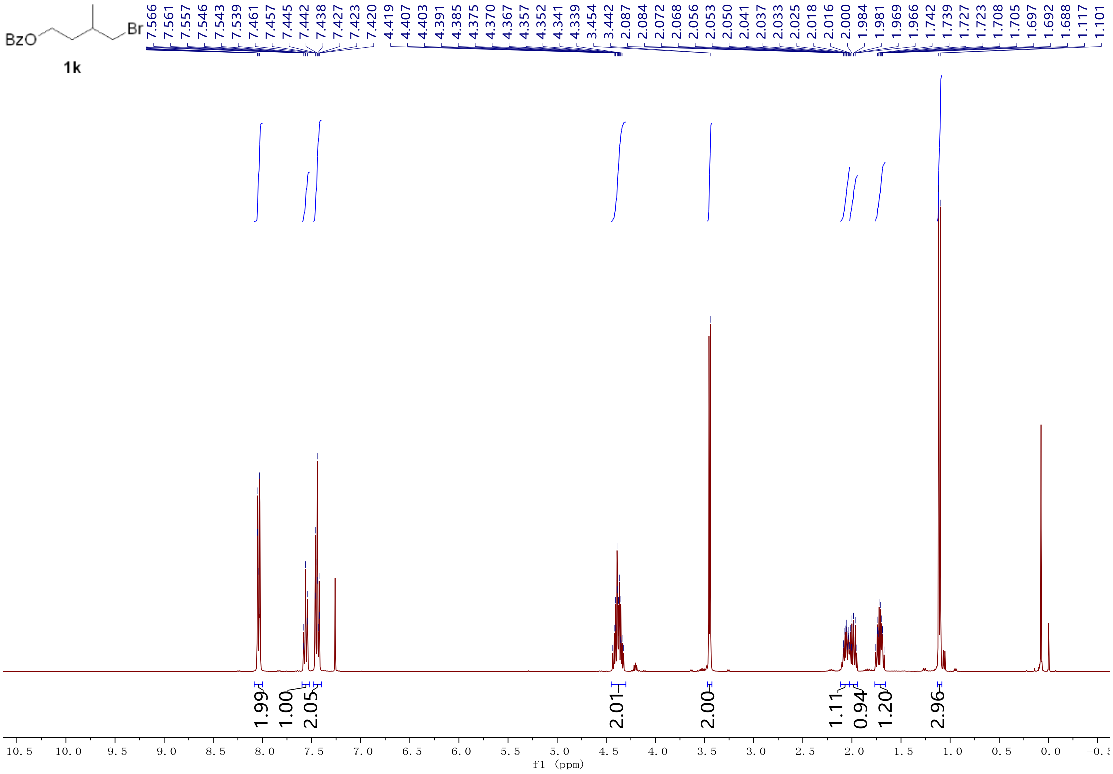

Supplementary Figure 26. ^{1}H NMR spectrum of compound **1k**

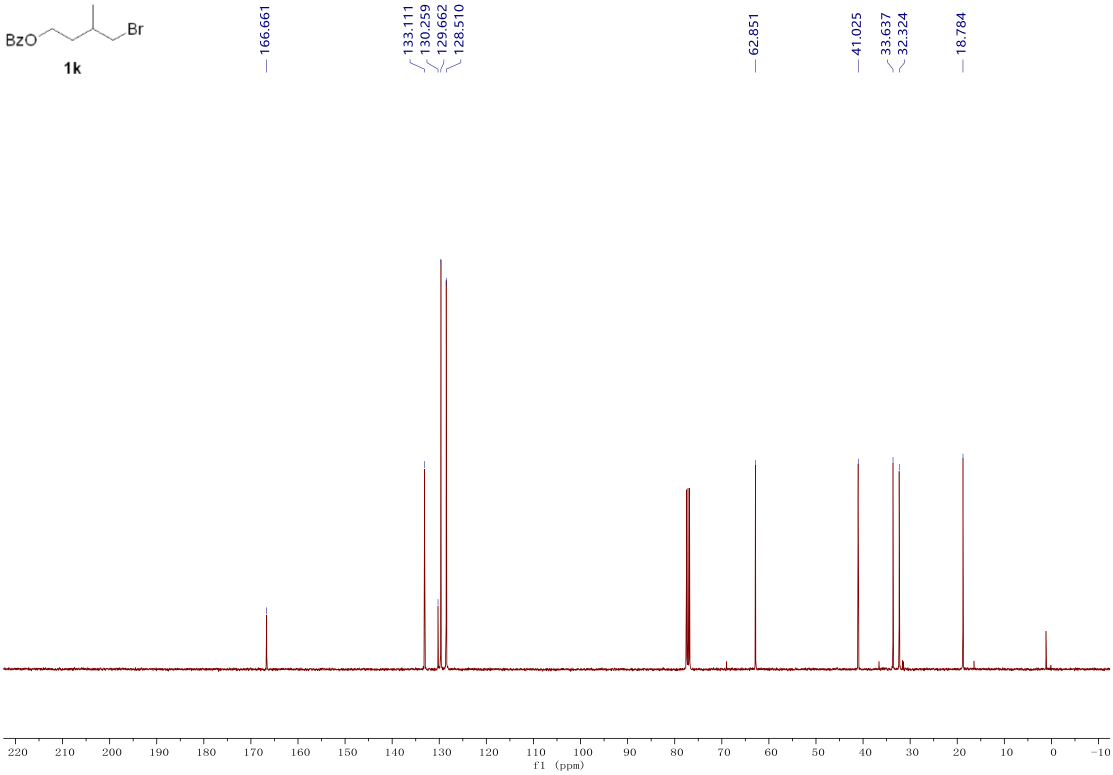

Supplementary Figure 27. ^{13}C NMR spectrum of compound **1k**

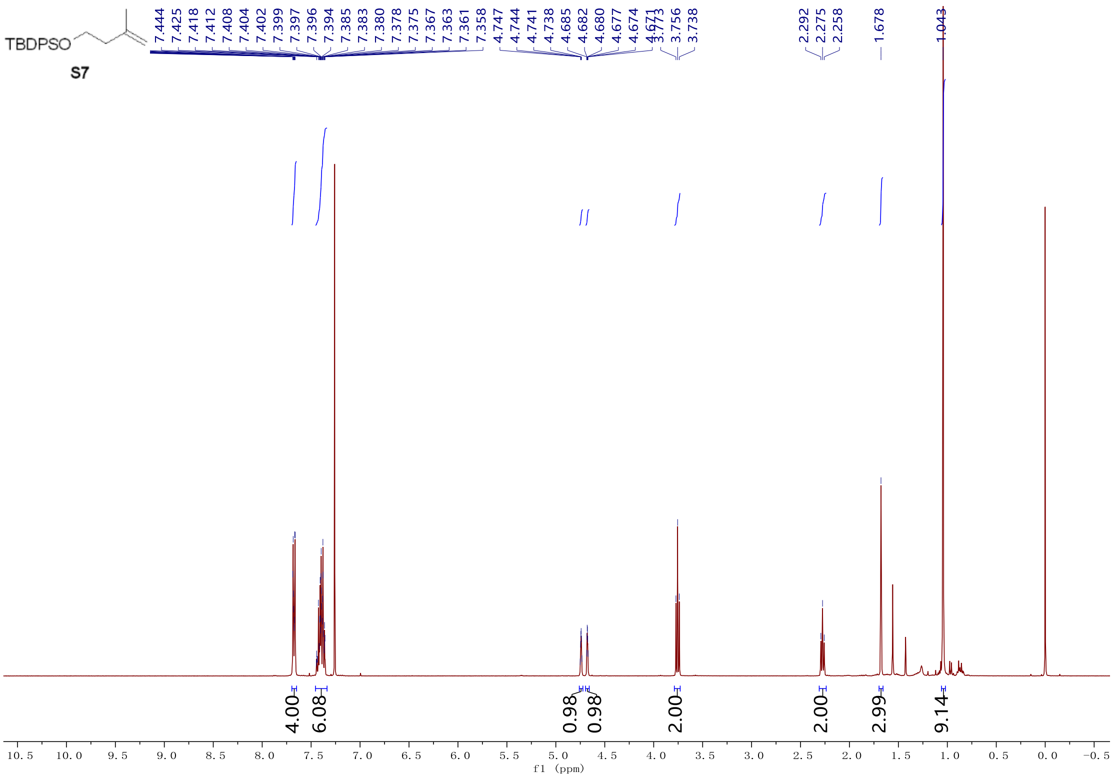

Supplementary Figure 28. ^{1}H NMR spectrum of compound **S7**

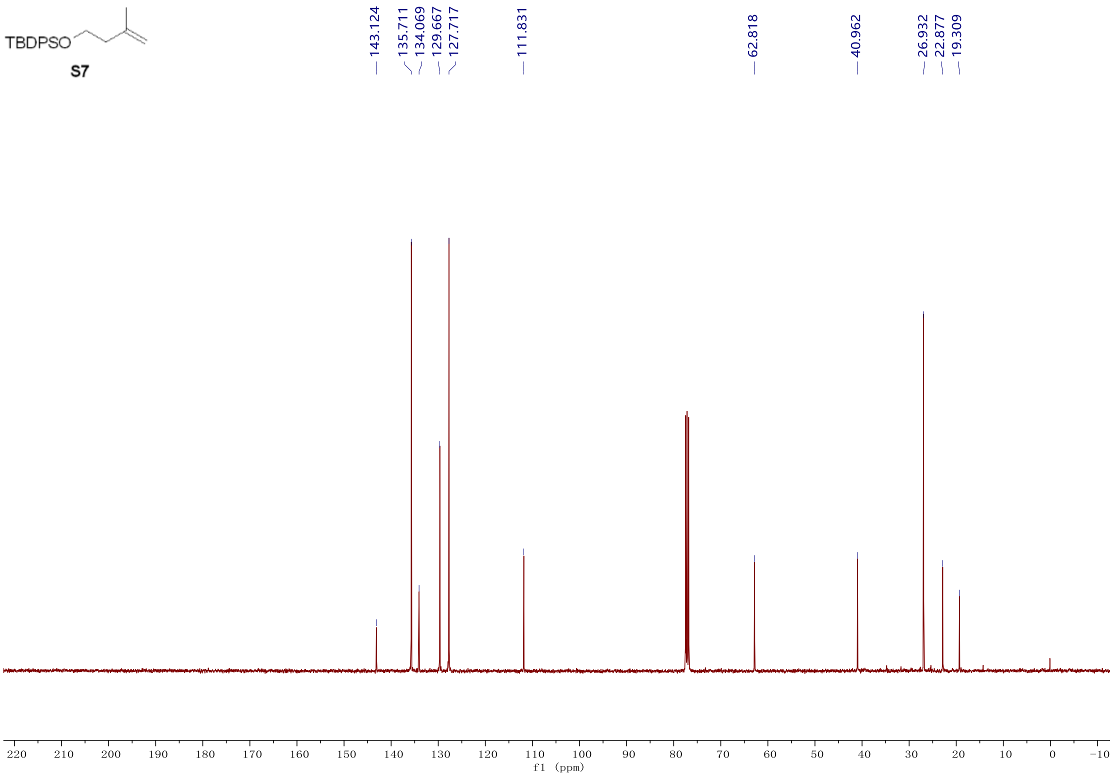

Supplementary Figure 29. ^{13}C NMR spectrum of compound **S7**

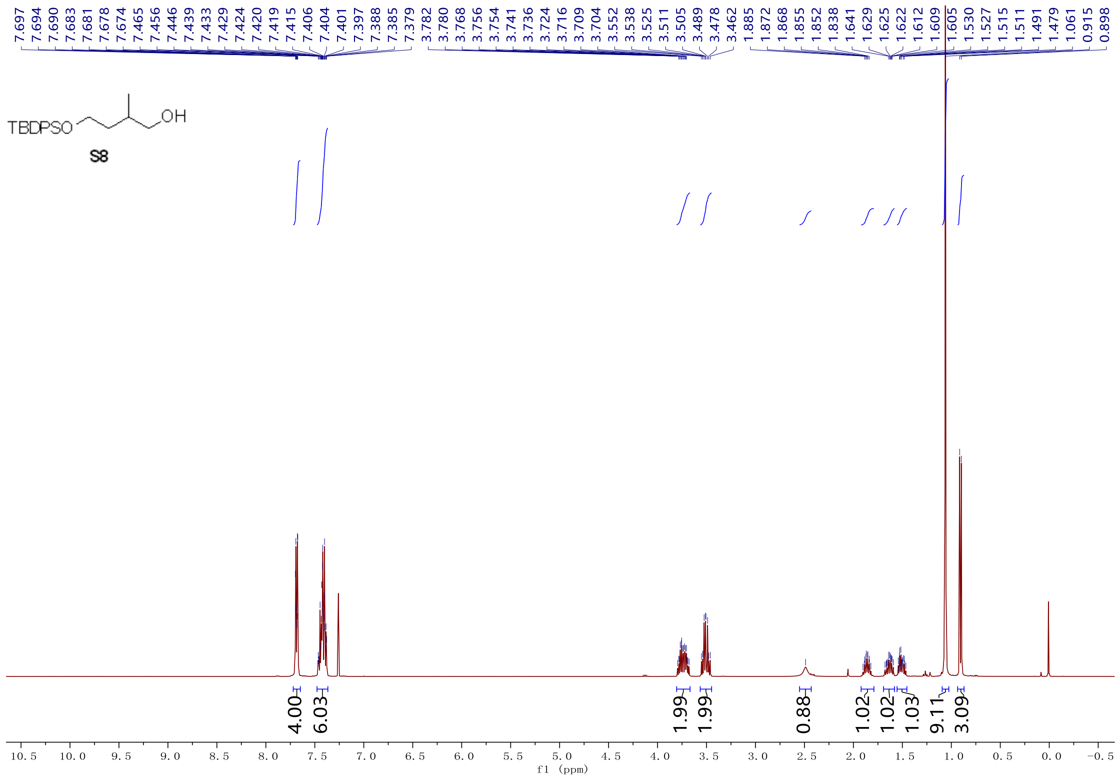

Supplementary Figure 30. ^{1}H NMR spectrum of compound **S8**

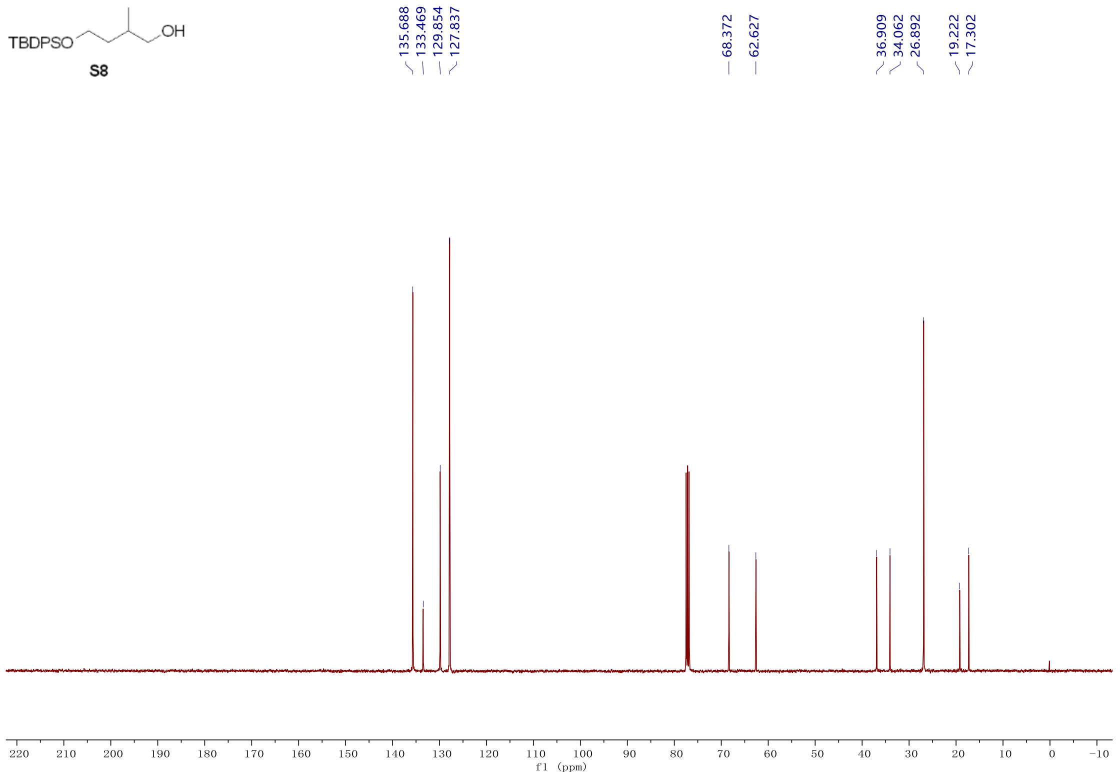

Supplementary Figure 31. ^{13}C NMR spectrum of compound **S8**

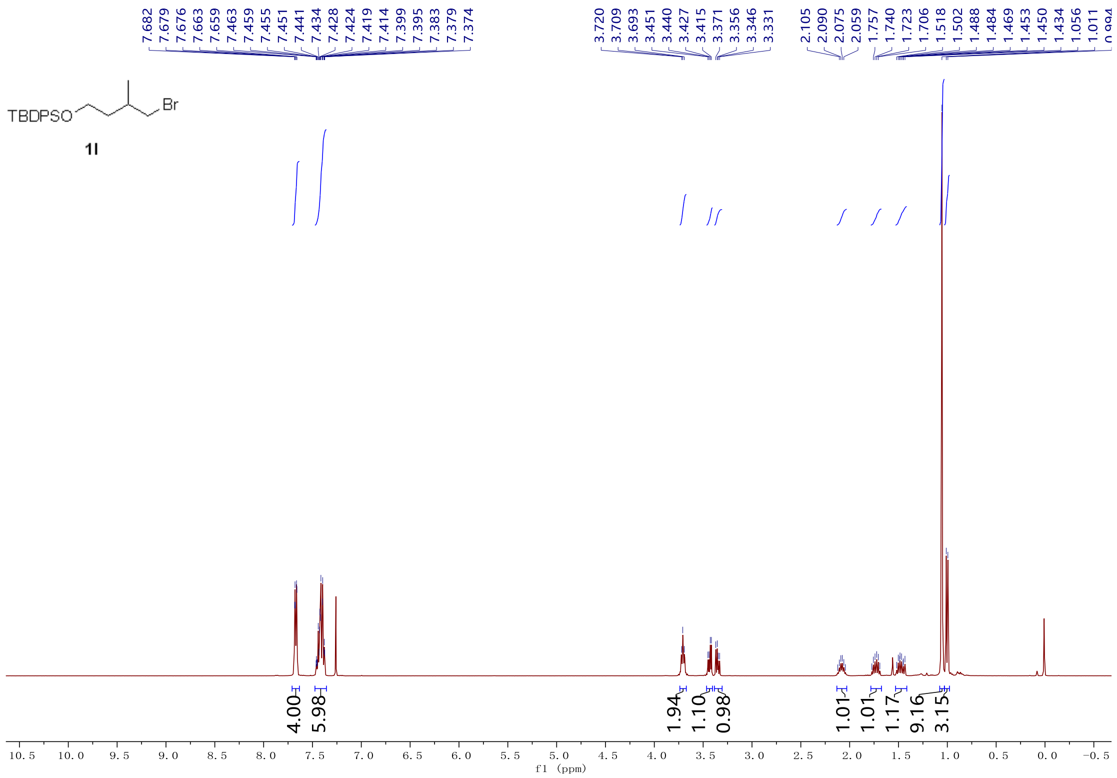

Supplementary Figure 32. ^{1}H NMR spectrum of compound **1l**

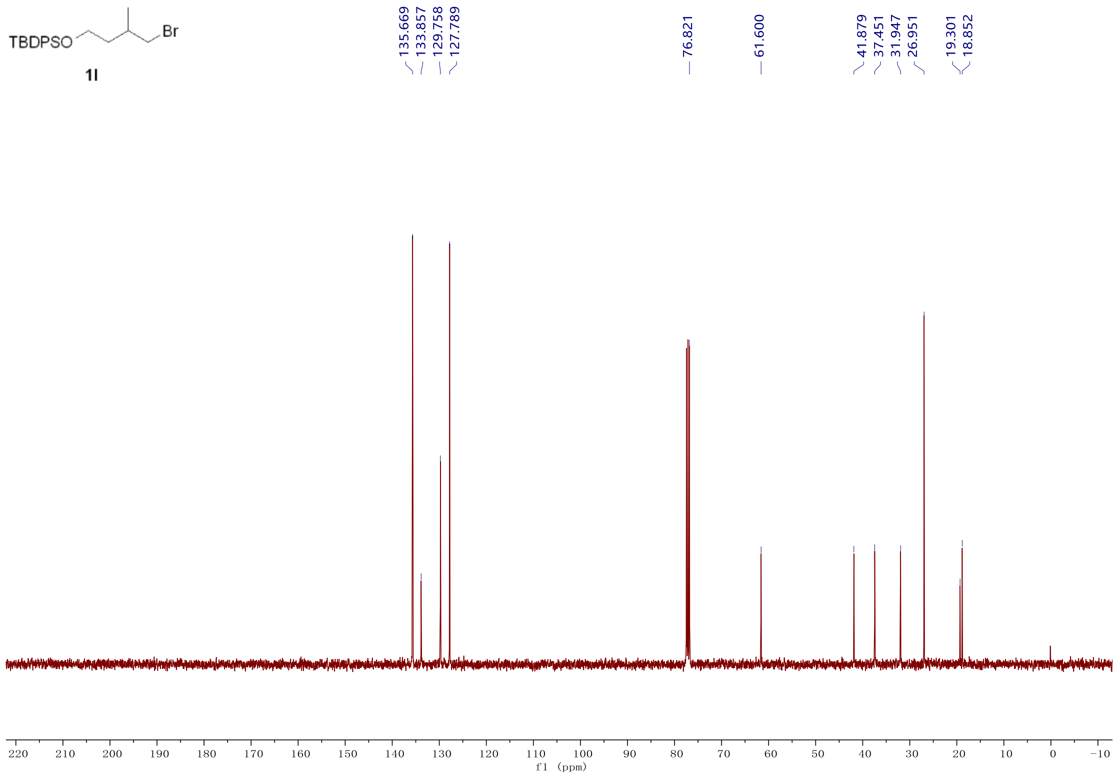

Supplementary Figure 33. ^{13}C NMR spectrum of compound **1l**

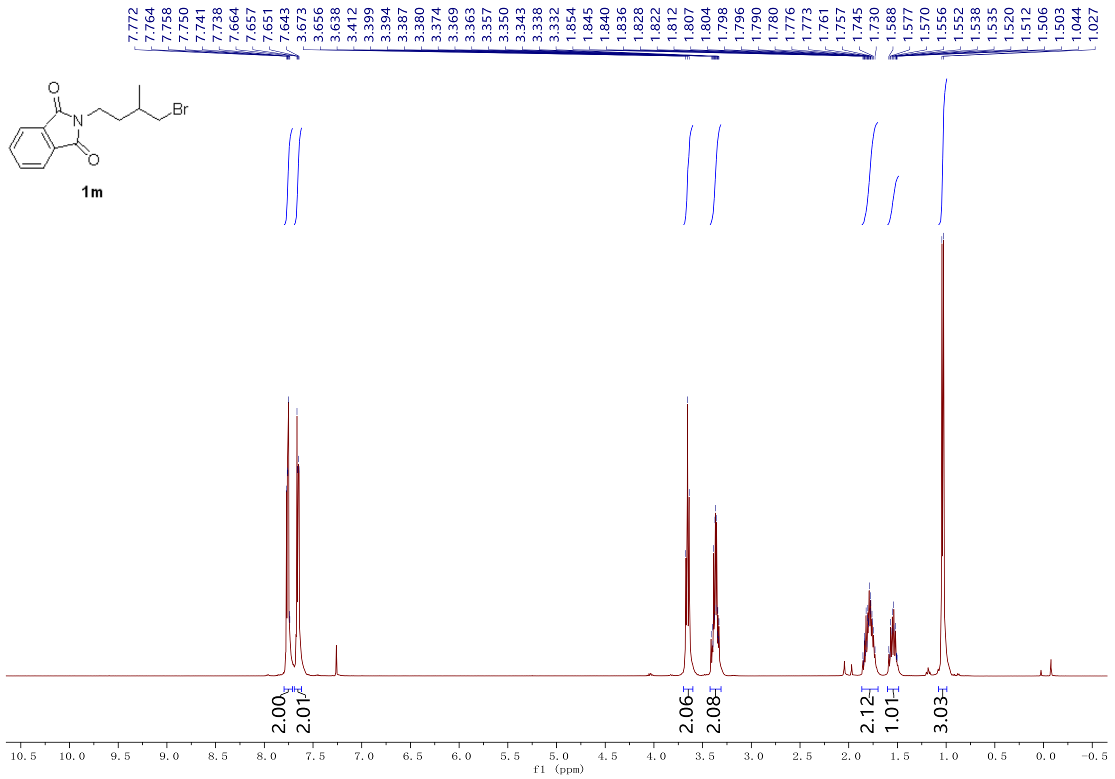

Supplementary Figure 34. ^{1}H NMR spectrum of compound **1m**

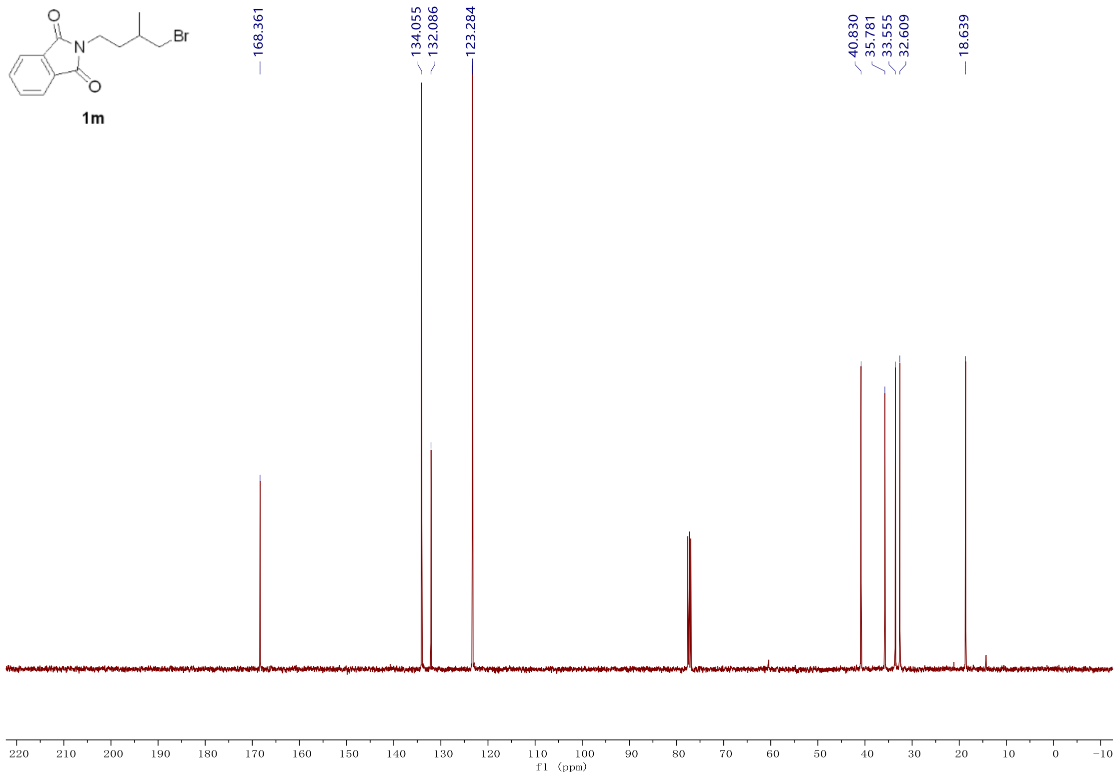

Supplementary Figure 35. ^{13}C NMR spectrum of compound **1m**

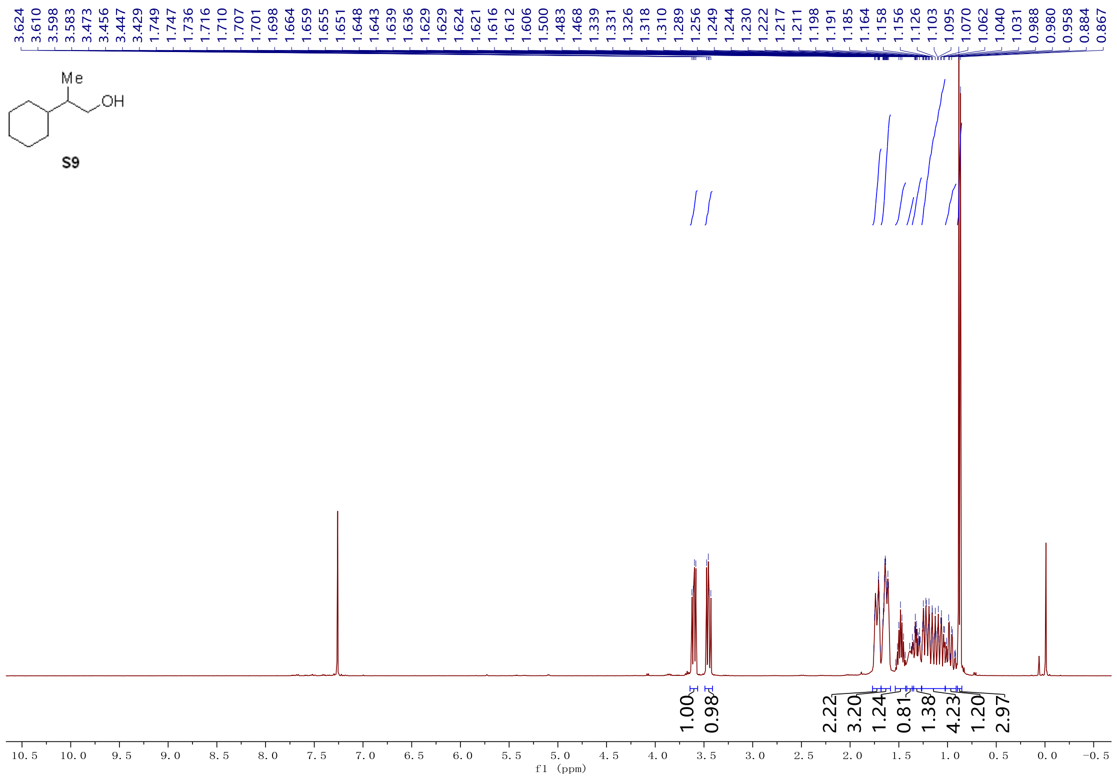

Supplementary Figure 36. ^{1}H NMR spectrum of compound **S9**

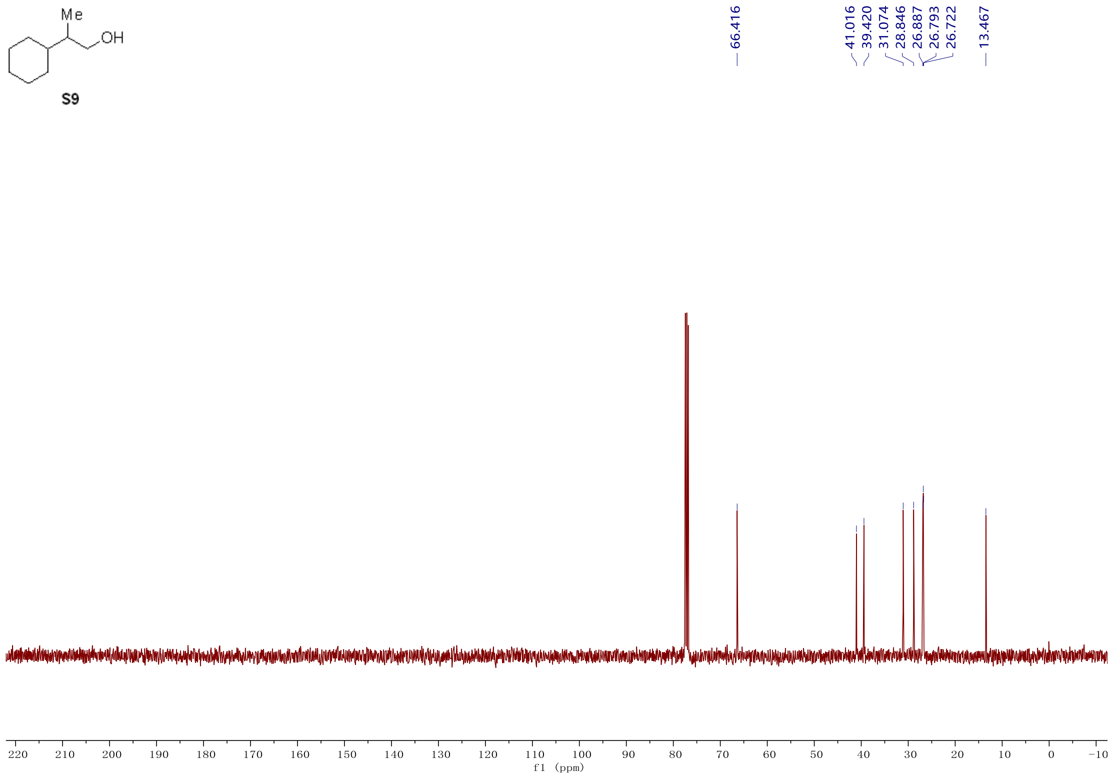

Supplementary Figure 37. ^{13}C NMR spectrum of compound **S9**

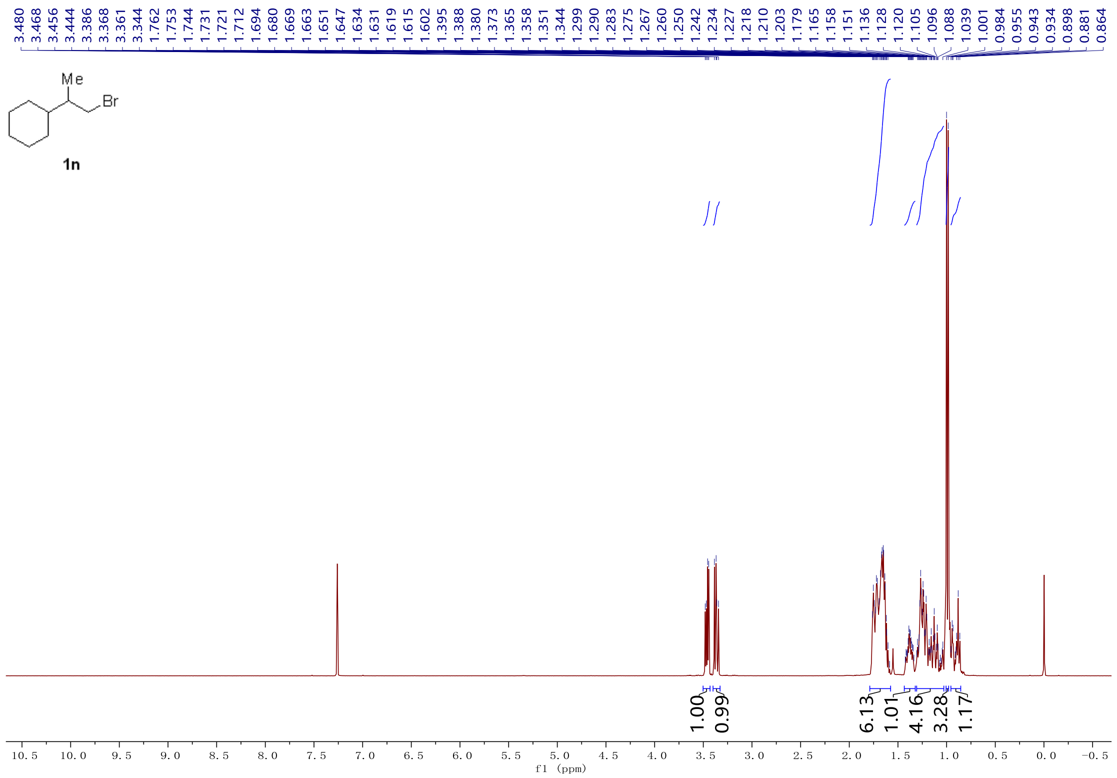

Supplementary Figure 38. ^{1}H NMR spectrum of compound **1n**

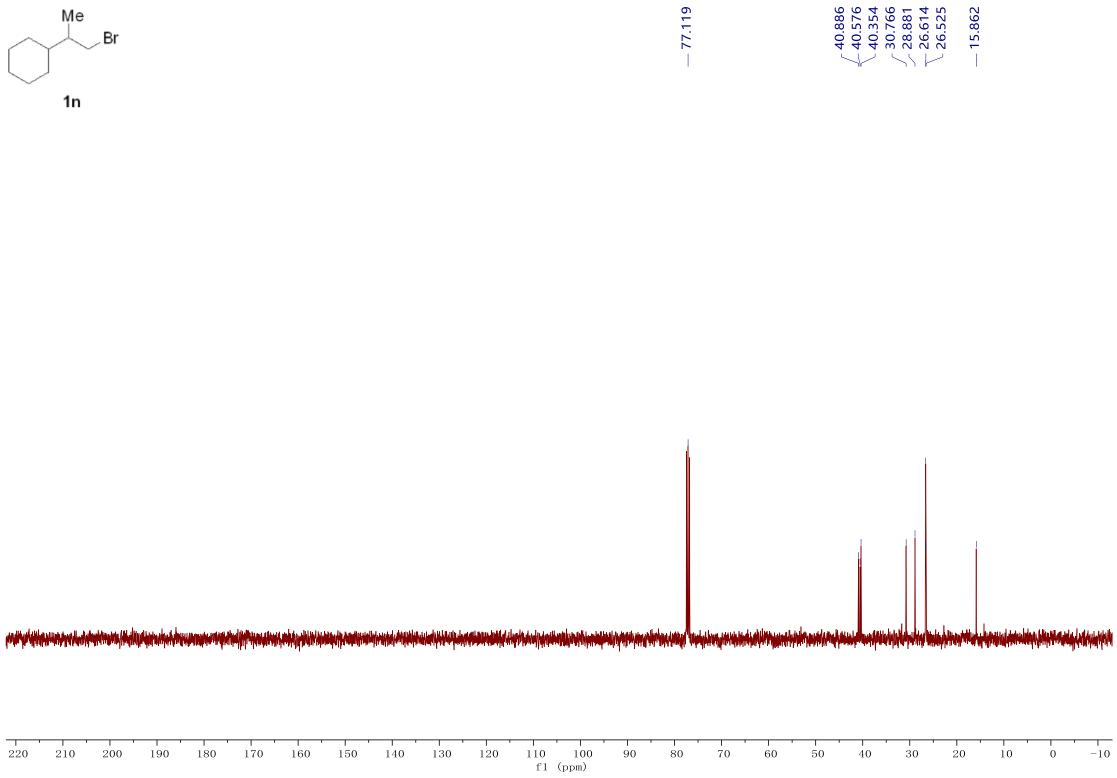

Supplementary Figure 39. ^{13}C NMR spectrum of compound **1n**

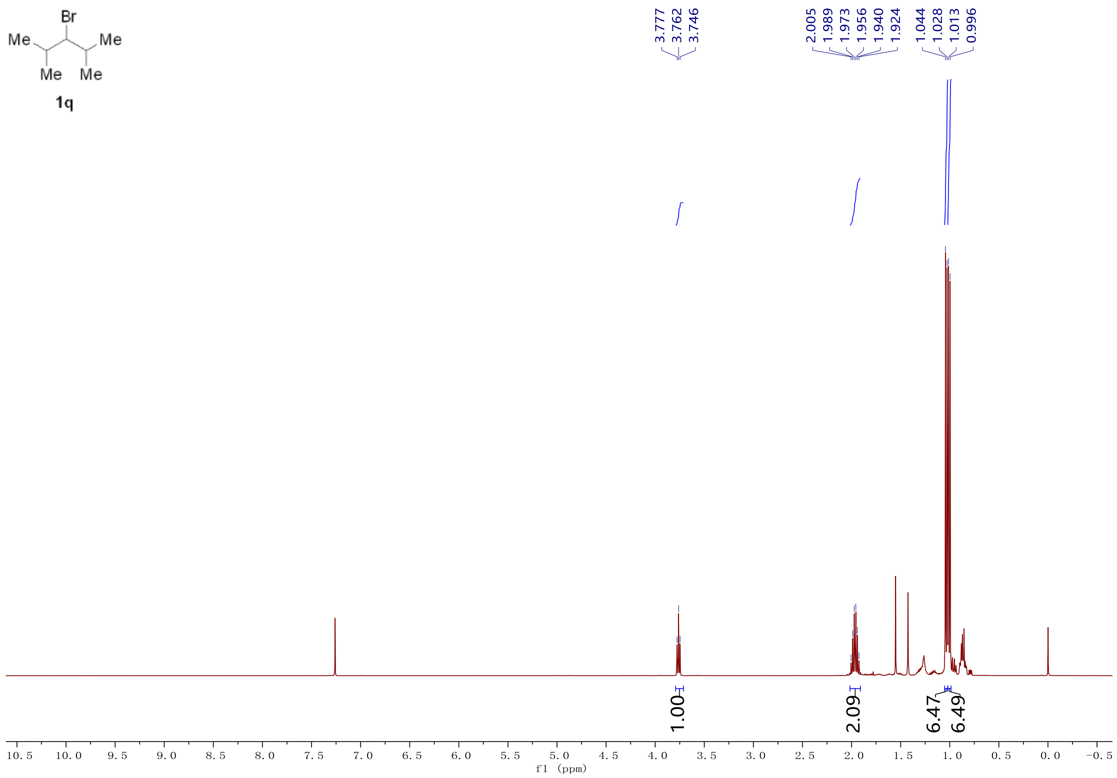

Supplementary Figure 40. ^{1}H NMR spectrum of compound **1q**

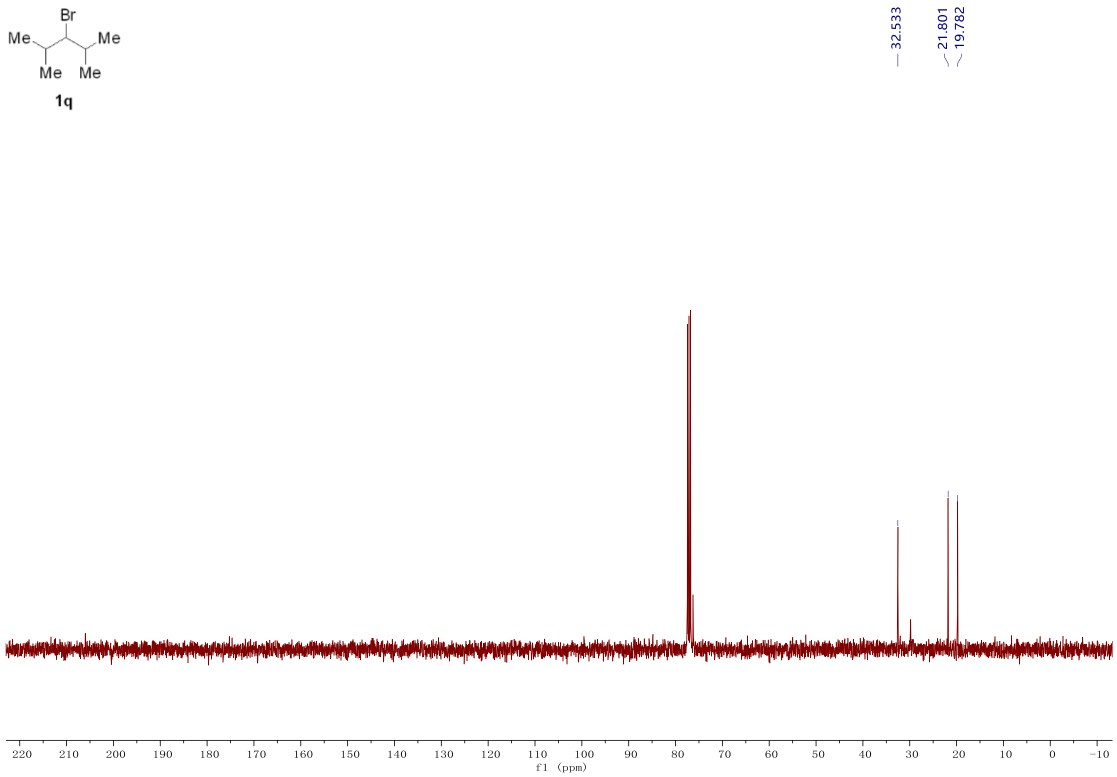

Supplementary Figure 41. ^{13}C NMR spectrum of compound **1q**

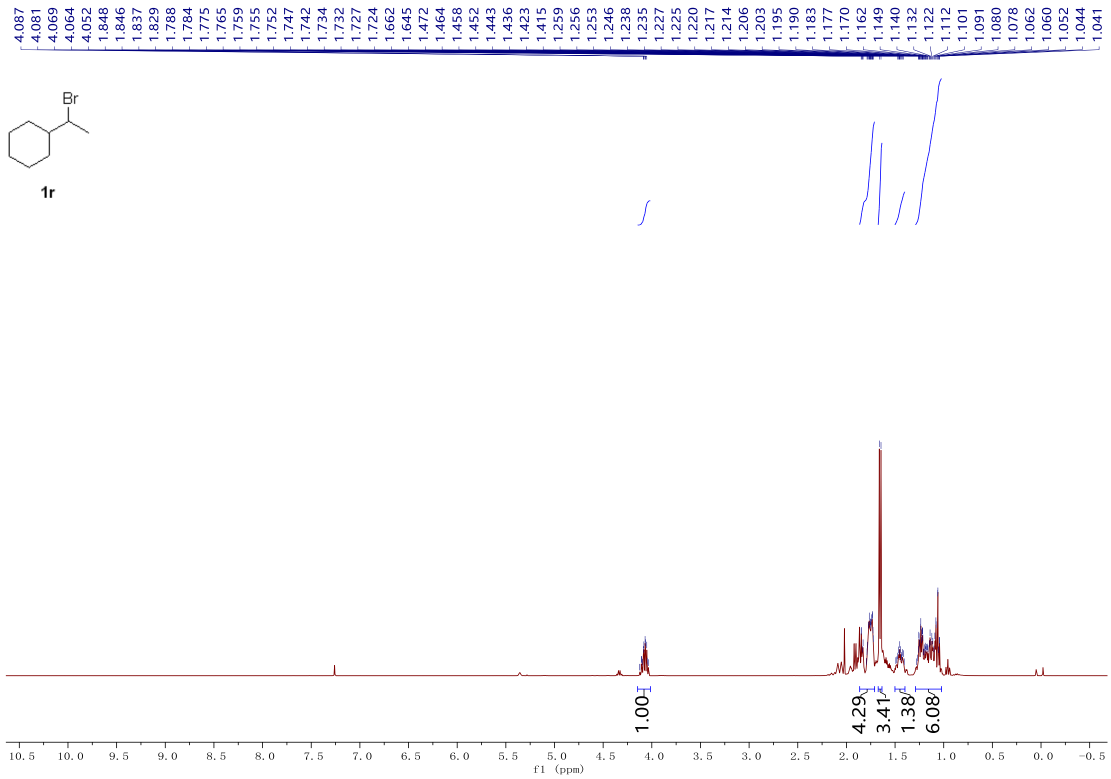

Supplementary Figure 42. ^{1}H NMR spectrum of compound **1r**

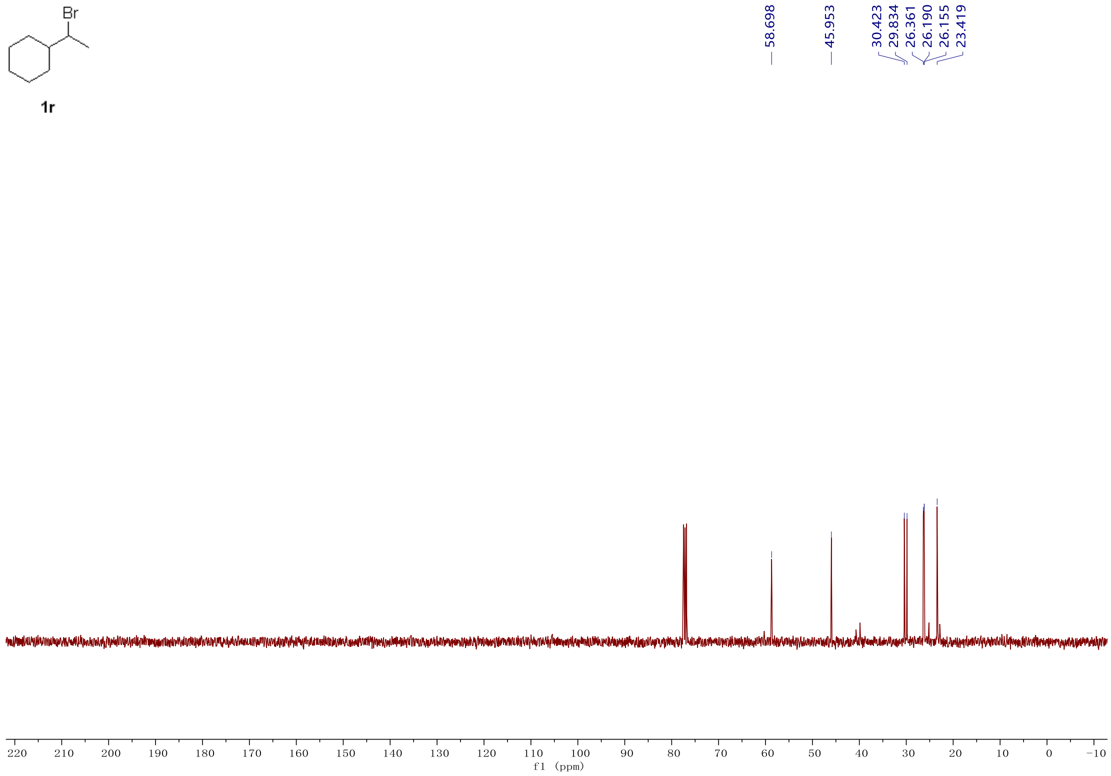

Supplementary Figure 43. ^{13}C NMR spectrum of compound **1r**

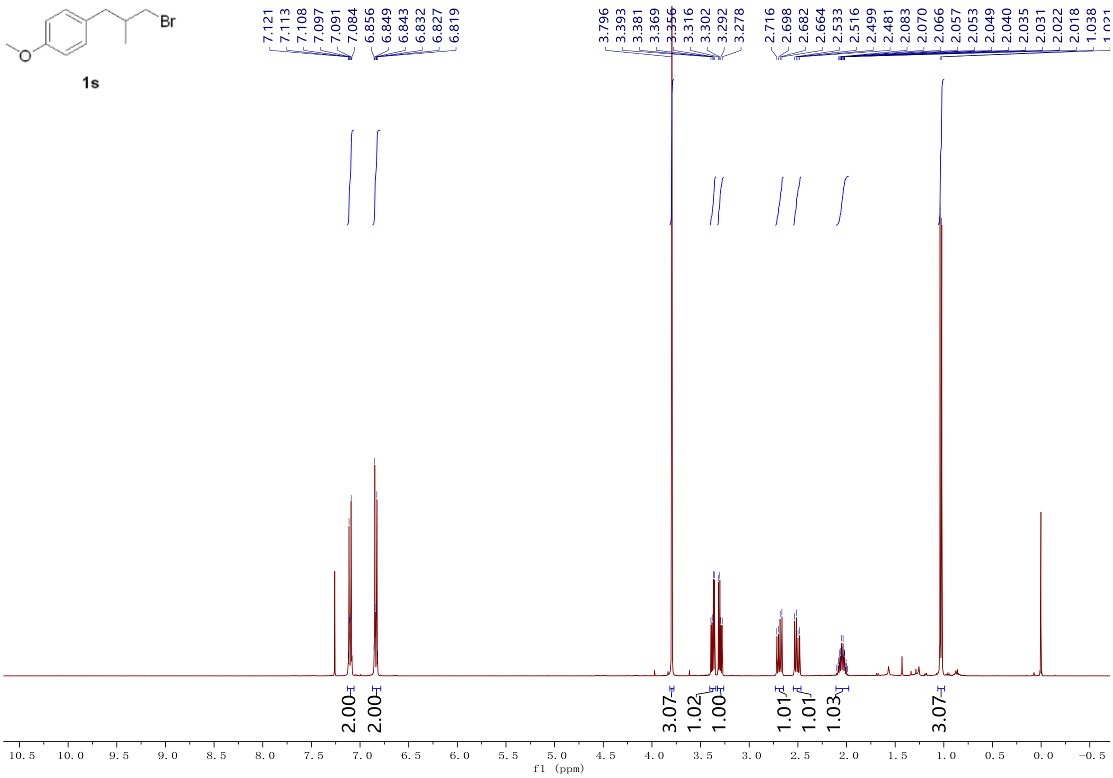

Supplementary Figure 44. ^{1}H NMR spectrum of compound **1s**

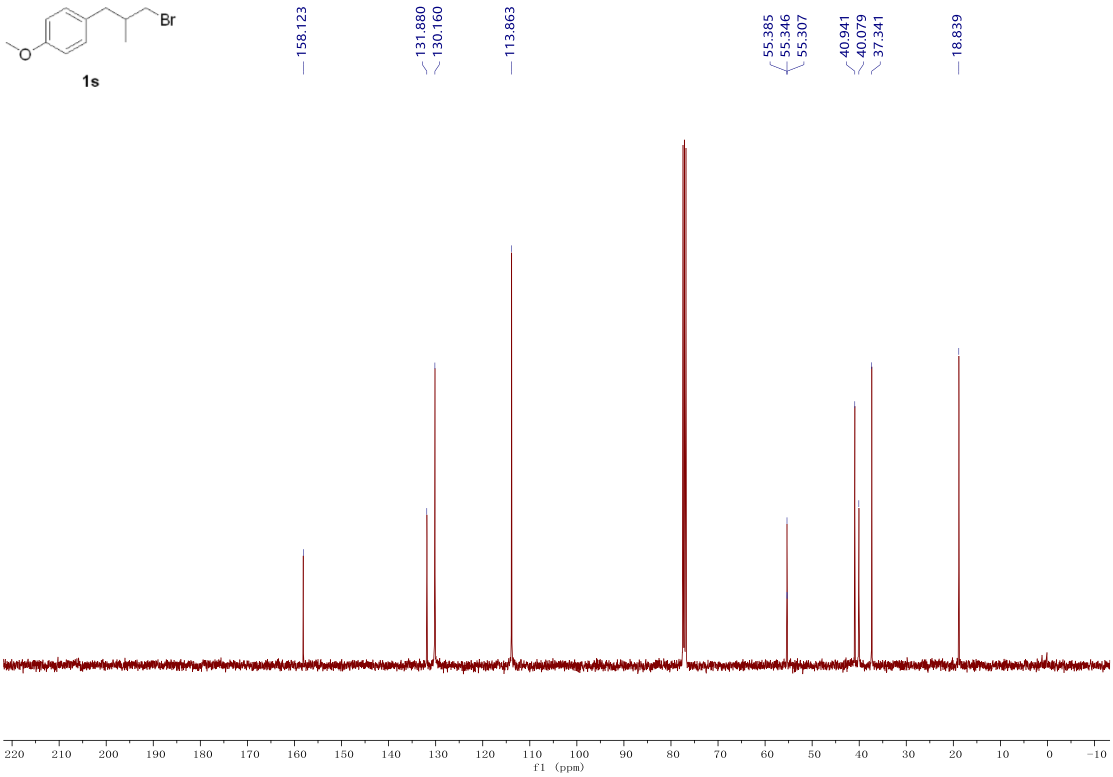

Supplementary Figure 45. ^{13}C NMR spectrum of compound **1s**

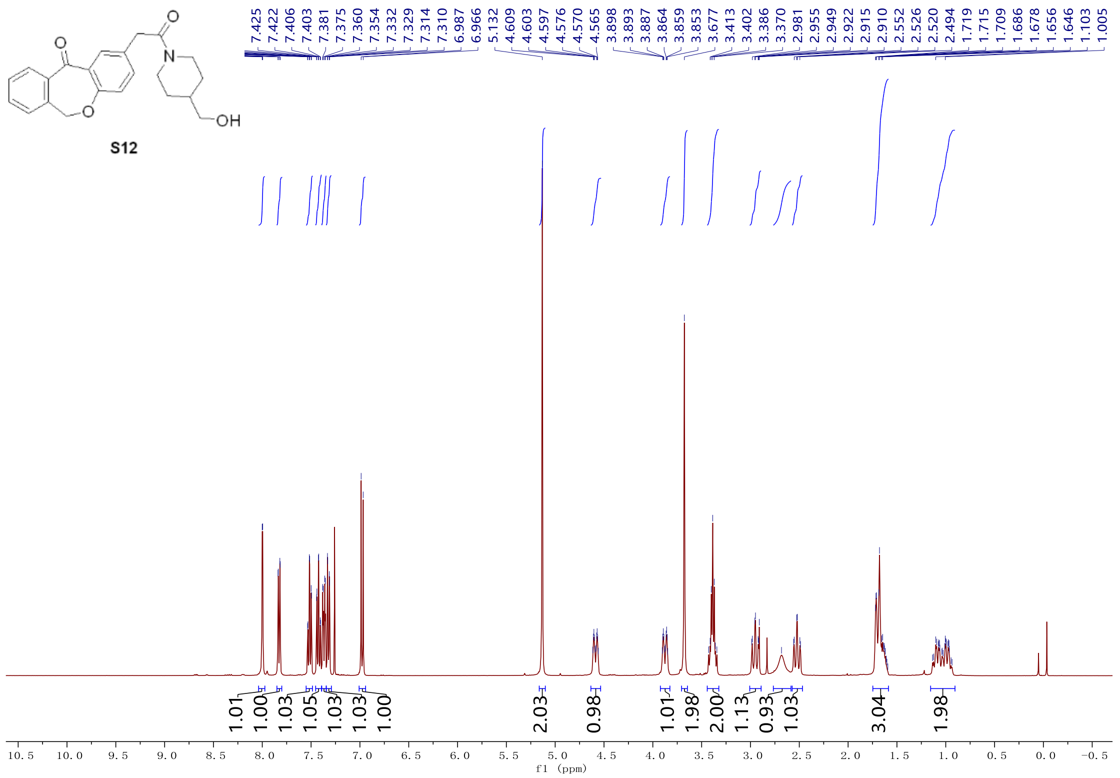

Supplementary Figure 46. ^{1}H NMR spectrum of compound **S12**

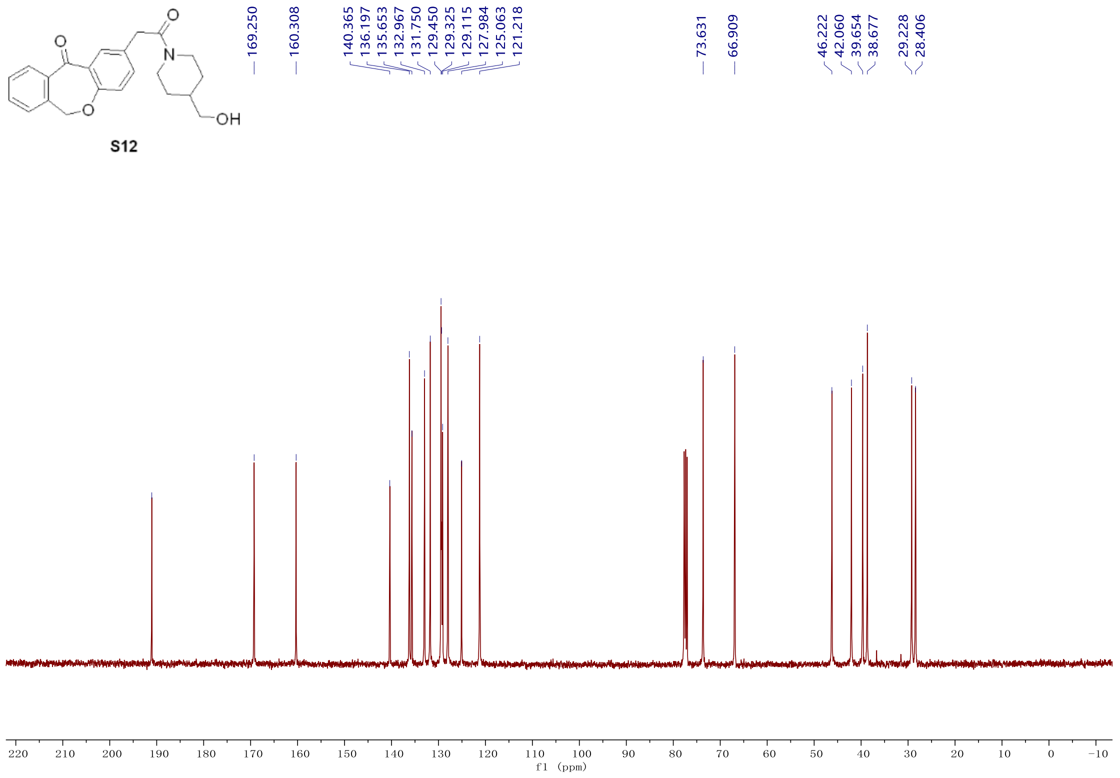

Supplementary Figure 47. ^{13}C NMR spectrum of compound **S12**

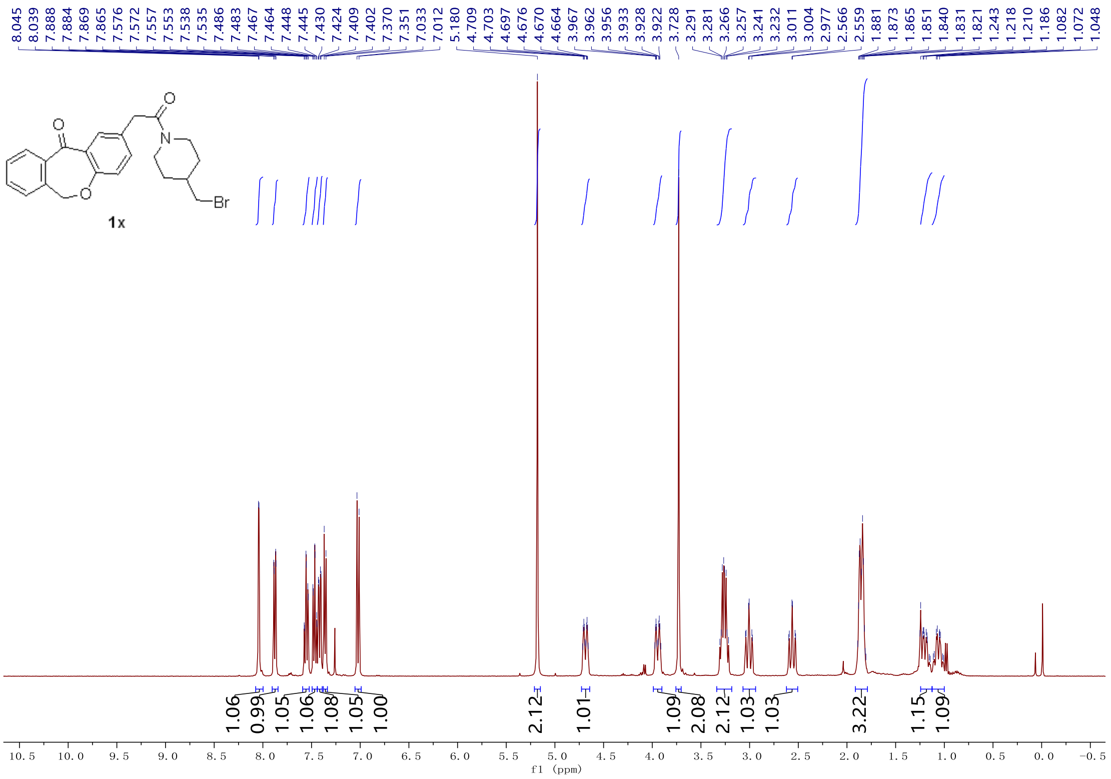

Supplementary Figure 48. ^{1}H NMR spectrum of compound **1x**

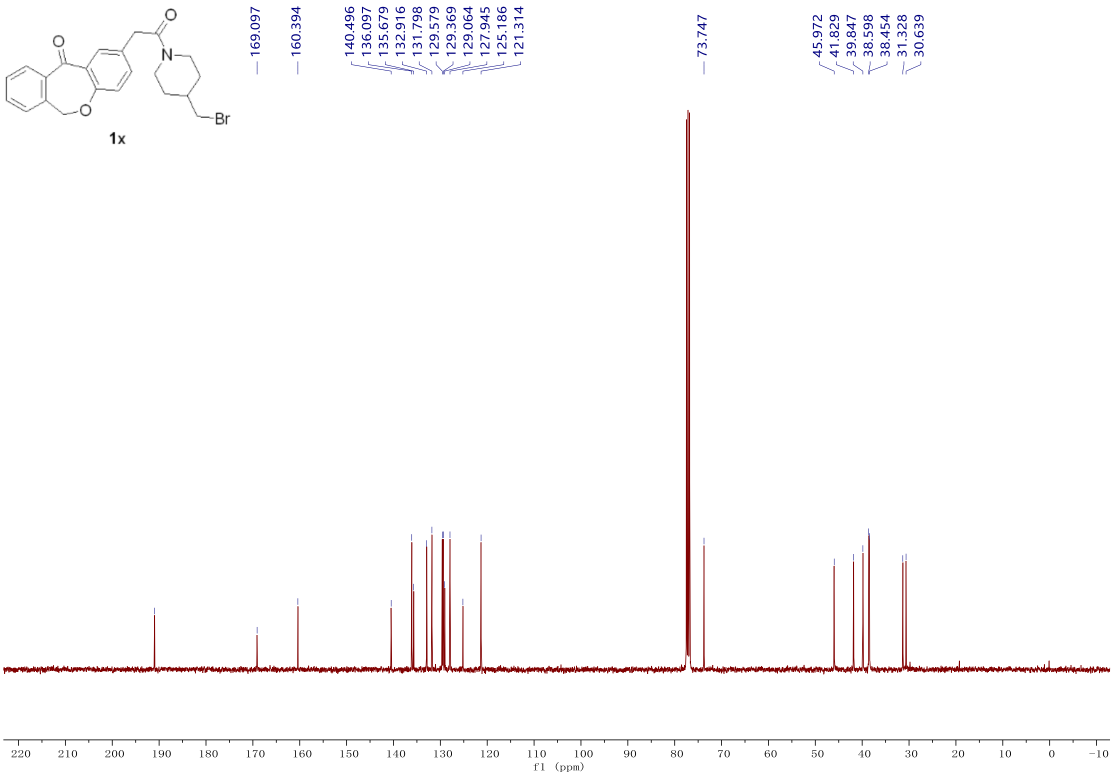

Supplementary Figure 49. ^{13}C NMR spectrum of compound **1x**

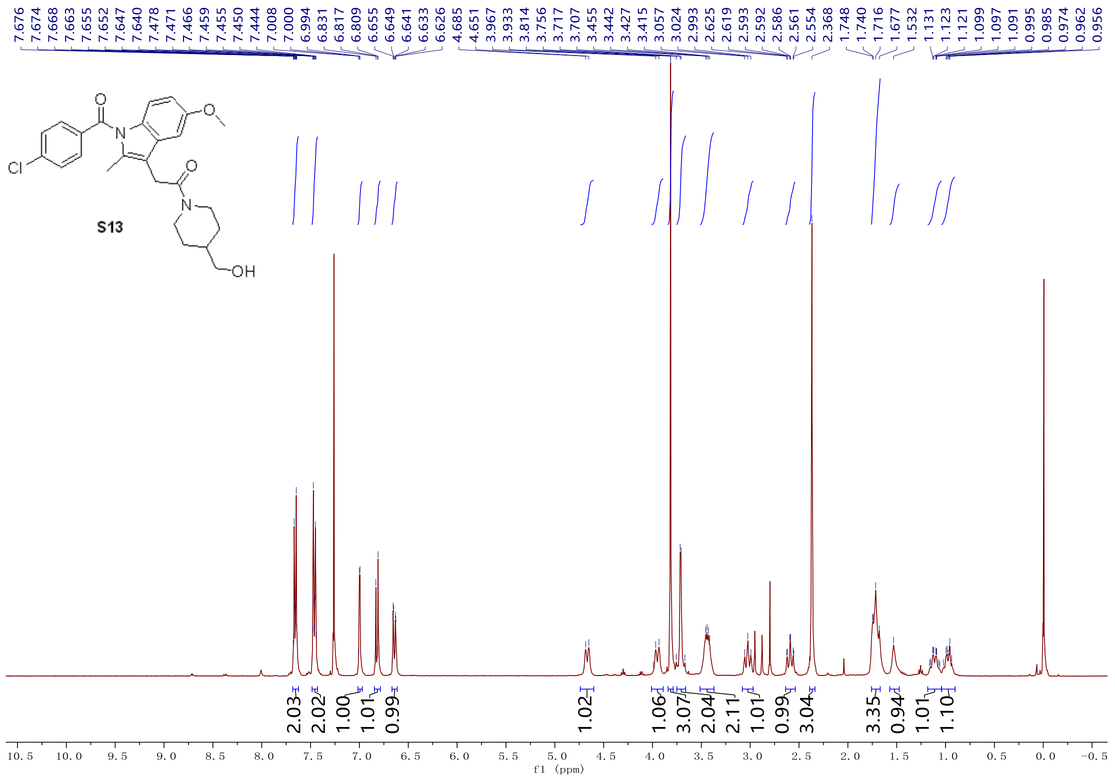

Supplementary Figure 50. ^{1}H NMR spectrum of compound **S13**

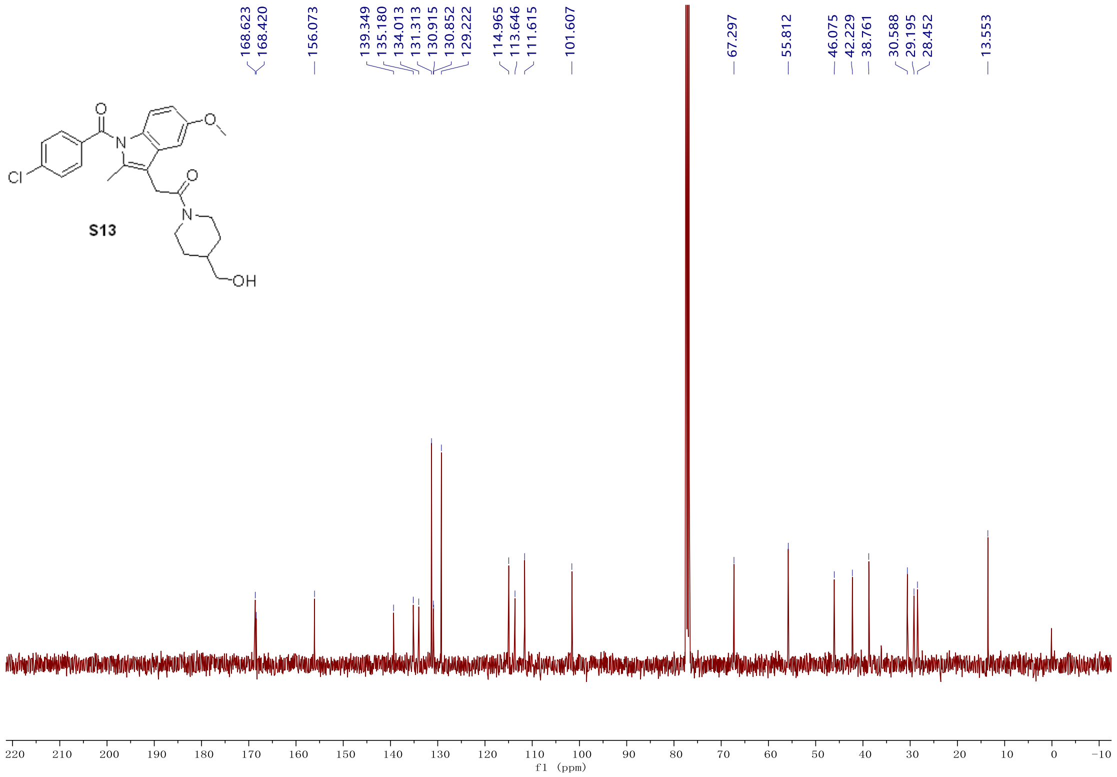

Supplementary Figure 51. ^{13}C NMR spectrum of compound **S14**

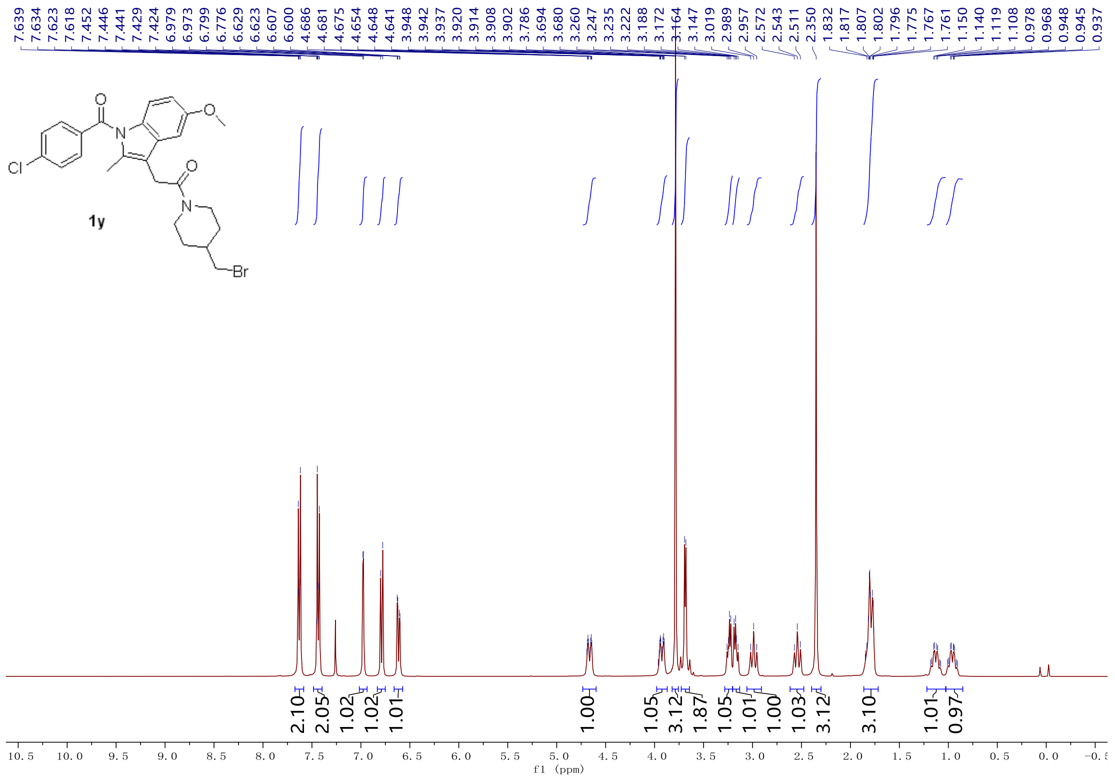

Supplementary Figure 52. ^{1}H NMR spectrum of compound **1y**

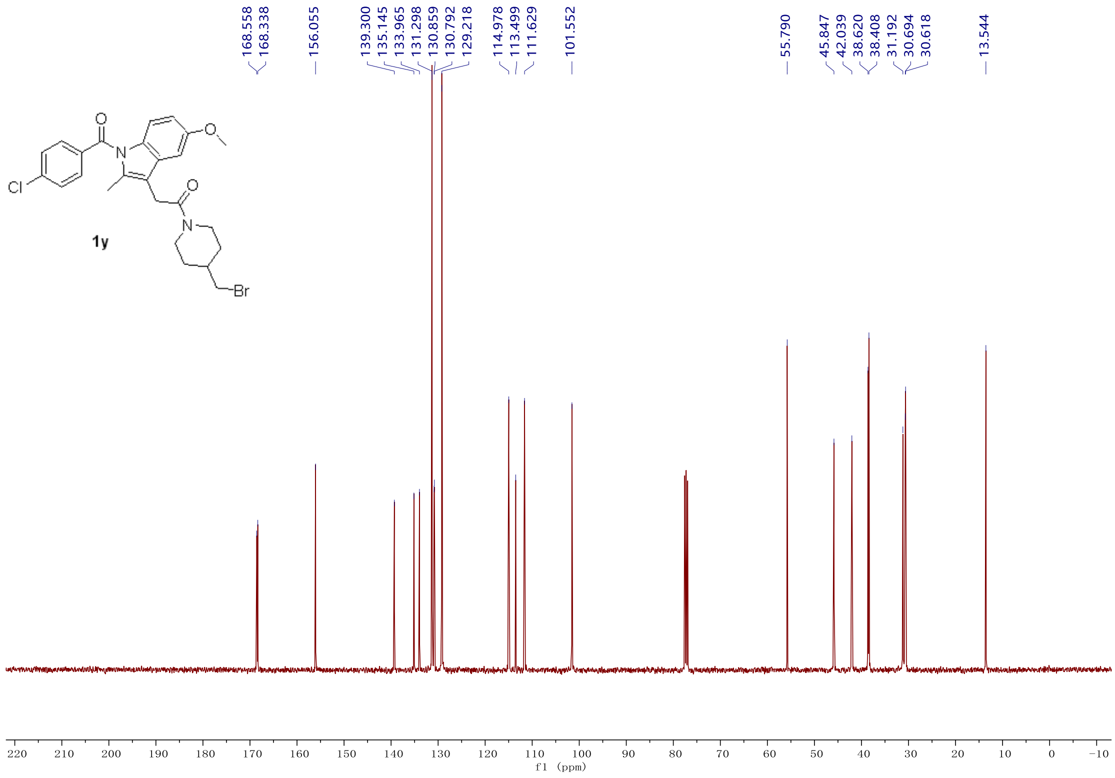

Supplementary Figure 53. ^{13}C NMR spectrum of compound **1y**

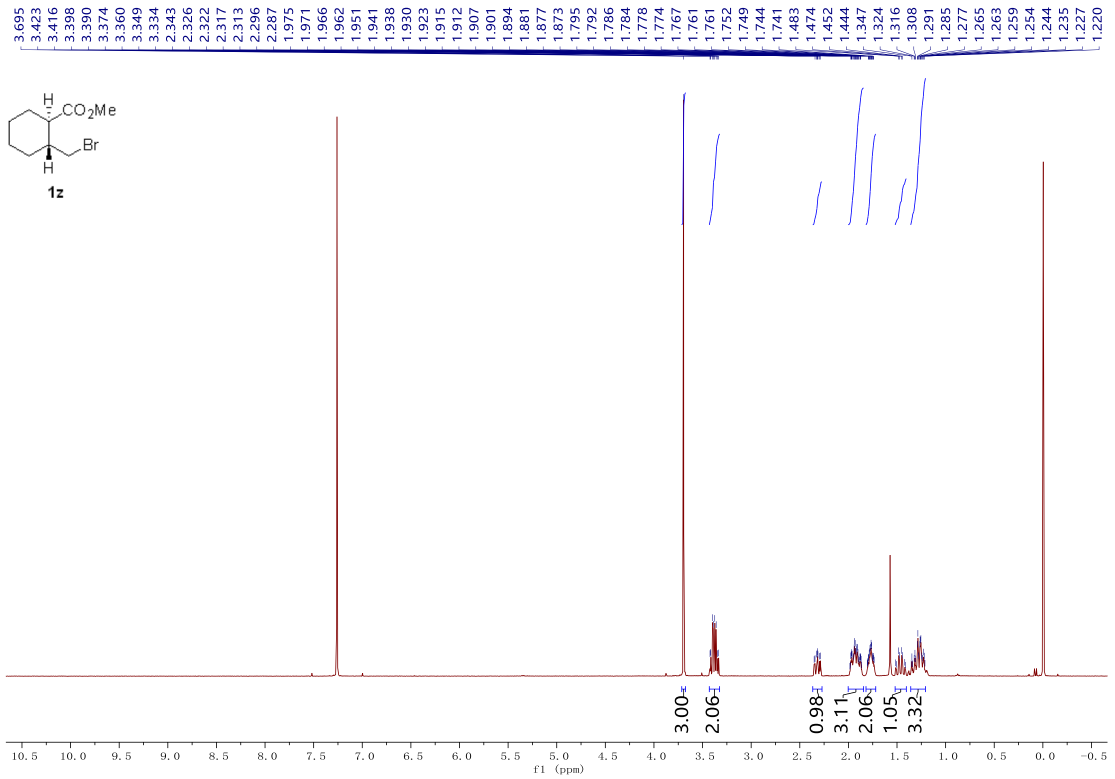

Supplementary Figure 54. ^{1}H NMR spectrum of compound **1z**

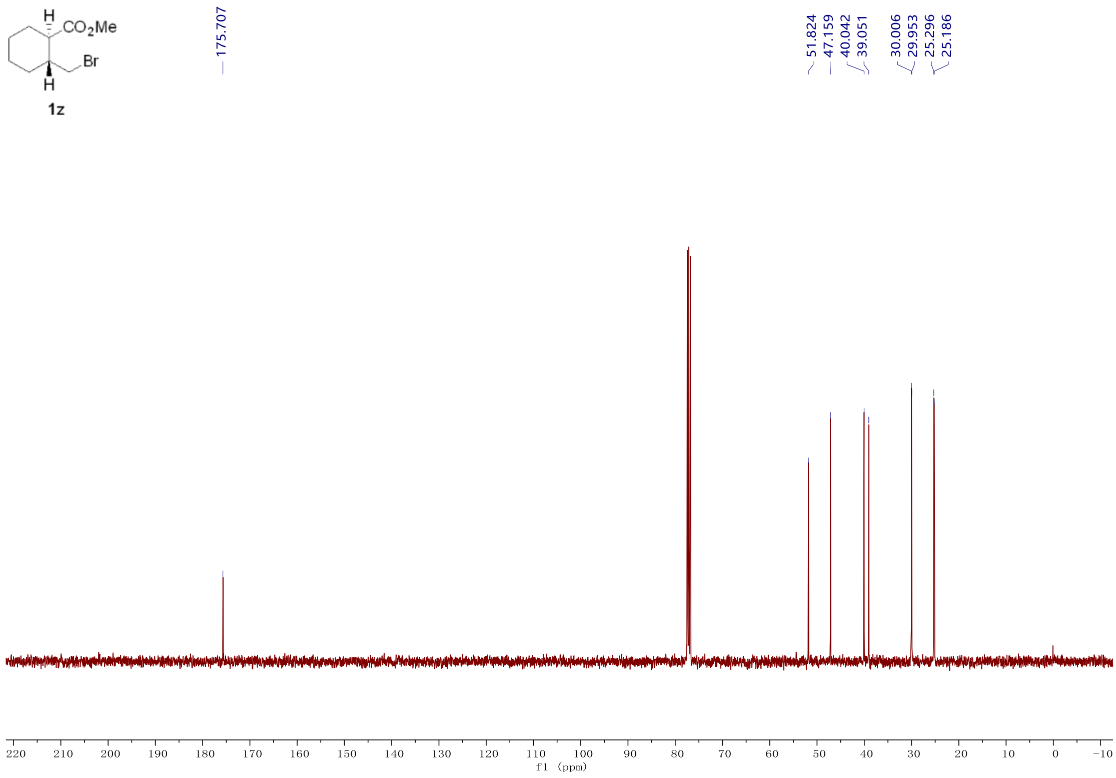

Supplementary Figure 55. ^{13}C NMR spectrum of compound **1z**

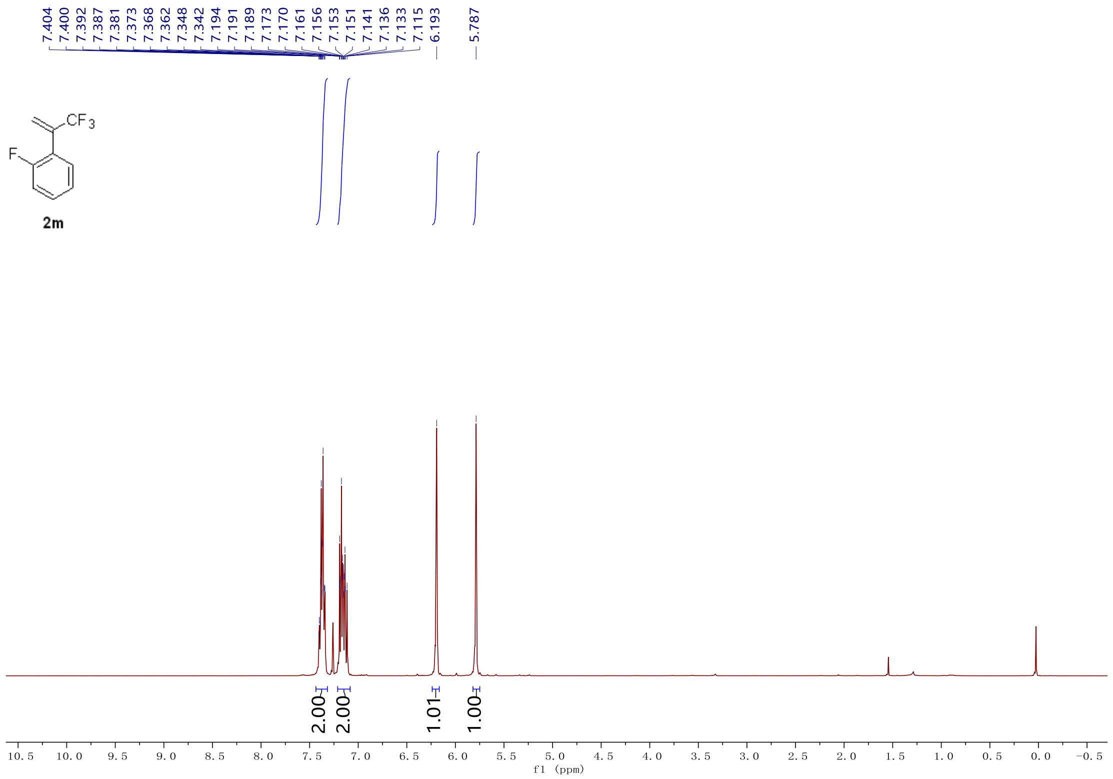

Supplementary Figure 56. ^{1}H NMR spectrum of compound **2m**

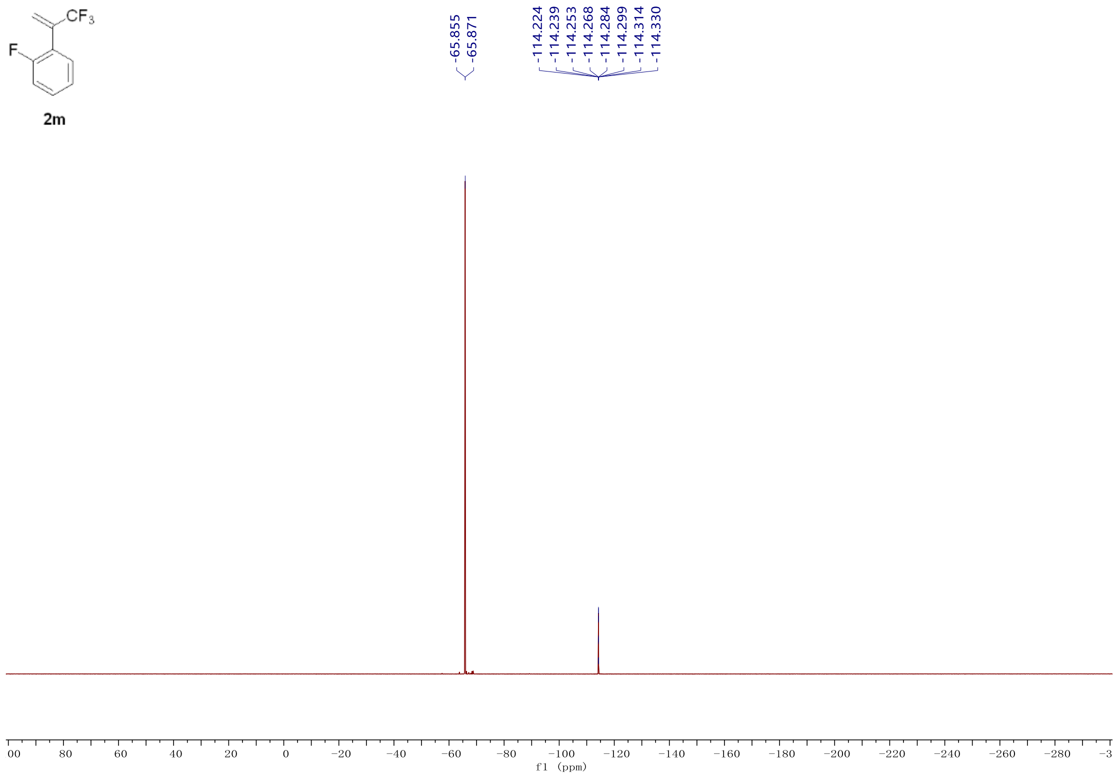

Supplementary Figure 57. ^{19}F NMR spectrum of compound **2m**

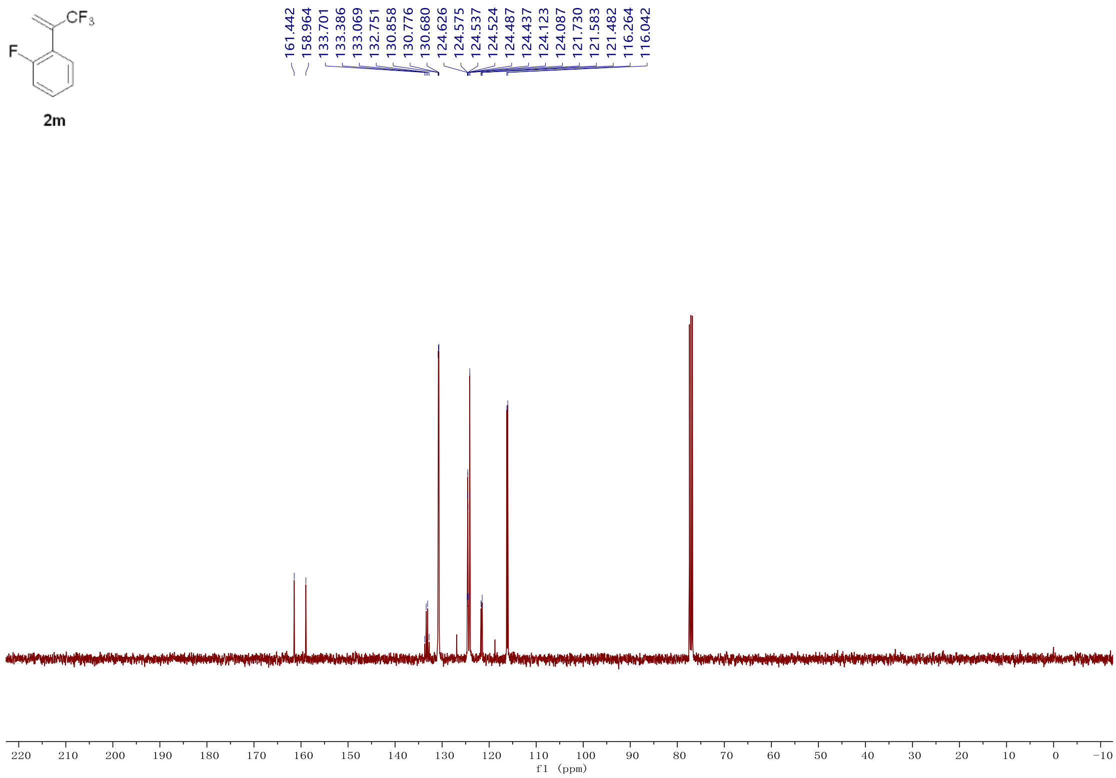

Supplementary Figure 58. ^{13}C NMR spectrum of compound **2m**

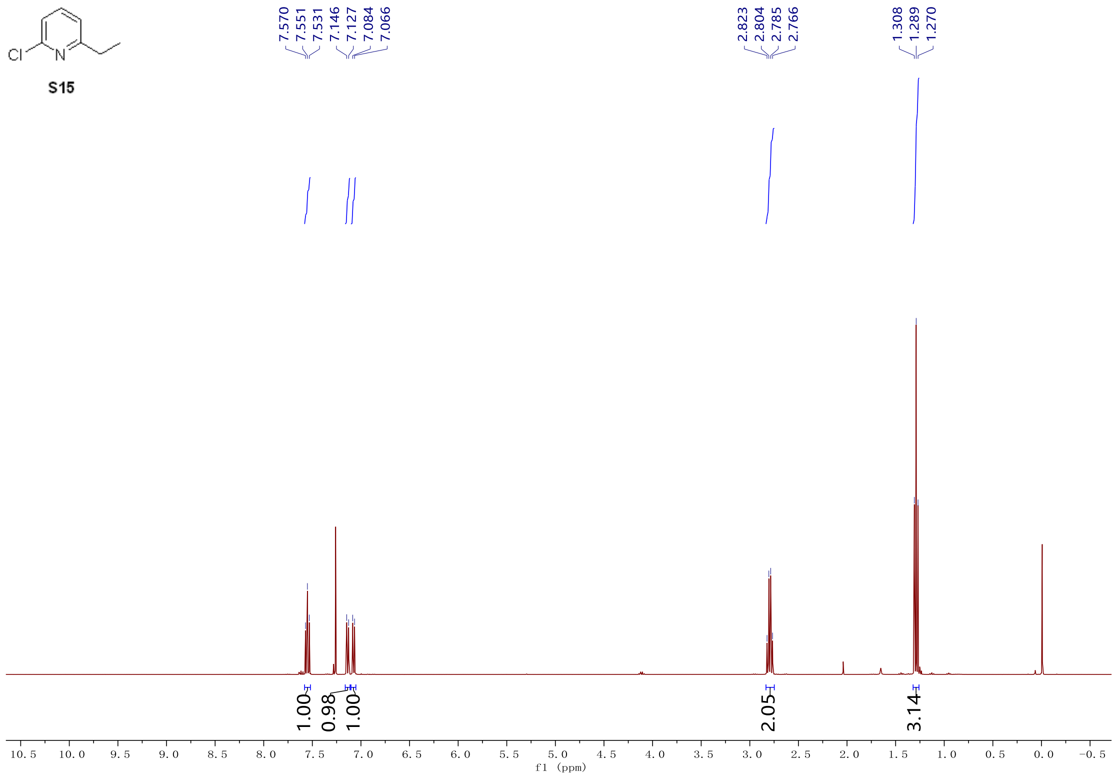

Supplementary Figure 59. ^{1}H NMR spectrum of compound **S15**

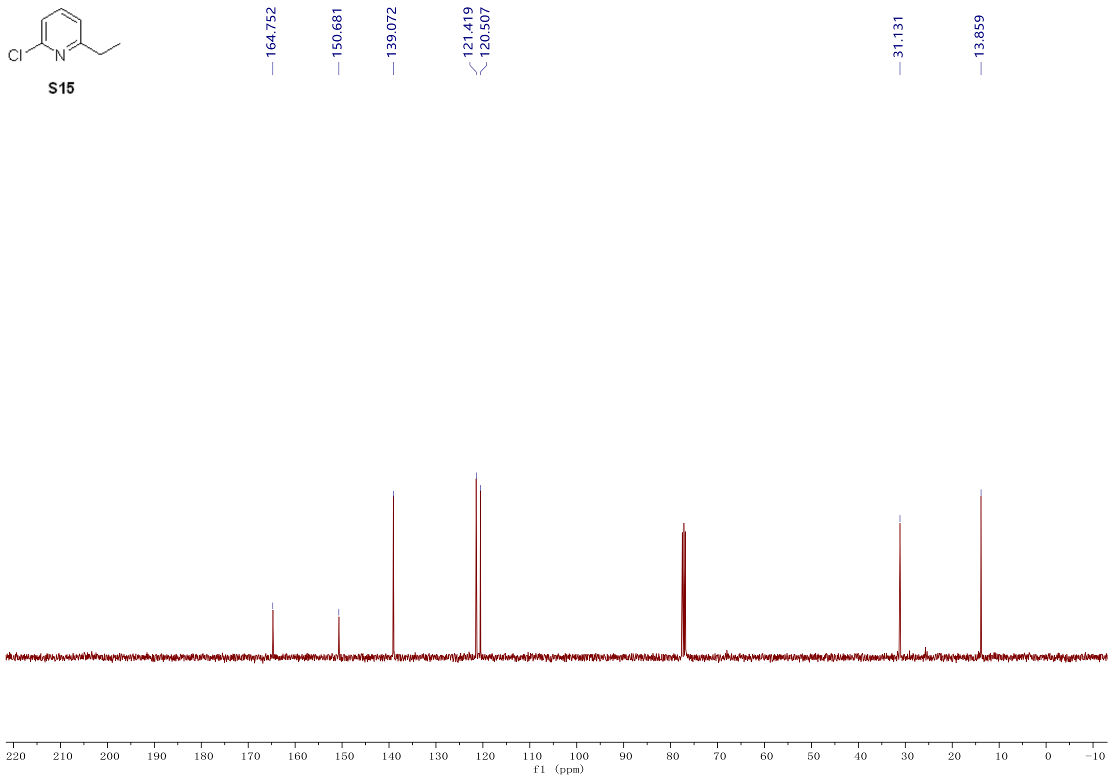

Supplementary Figure 60. ^{13}C NMR spectrum of compound **S15**

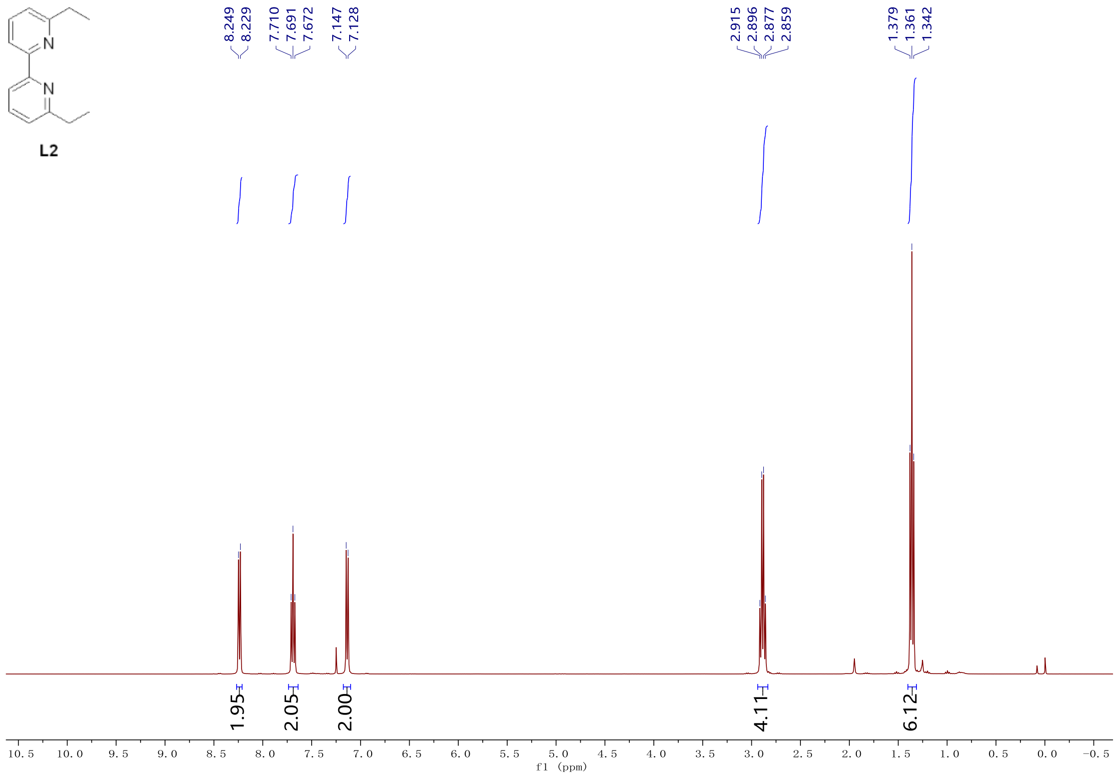

Supplementary Figure 61. ^{1}H NMR spectrum of compound **L2**

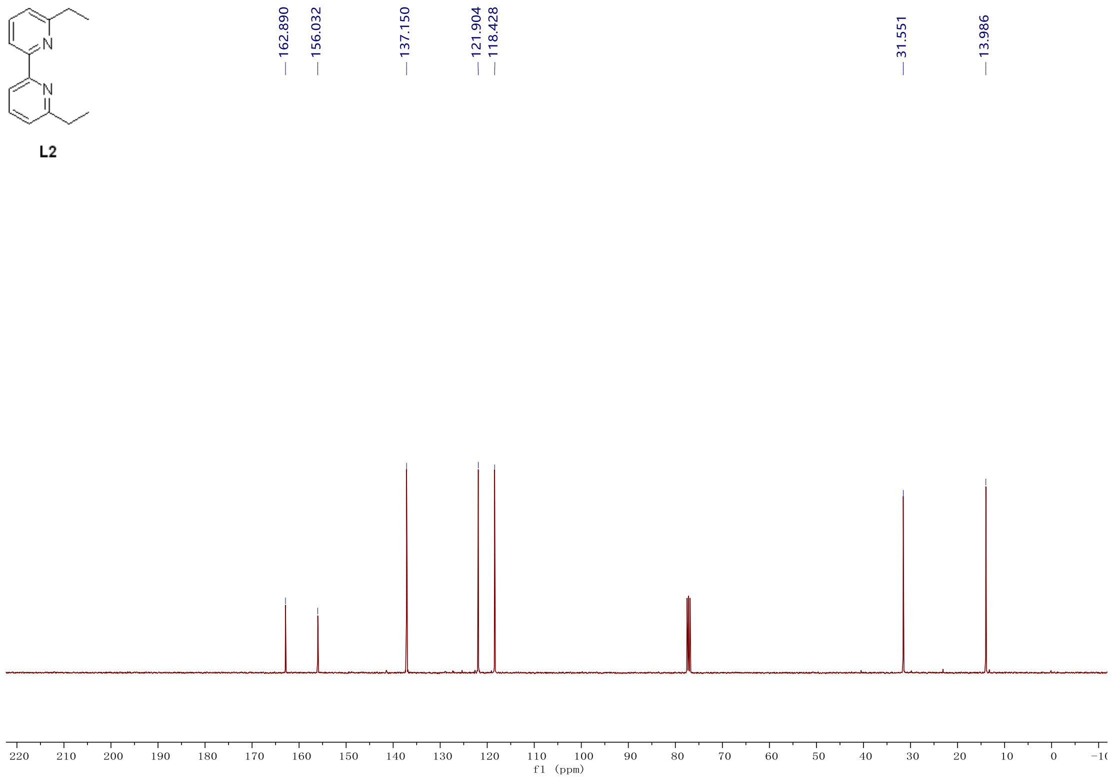

Supplementary Figure 62. ^{13}C NMR spectrum of compound **L2**

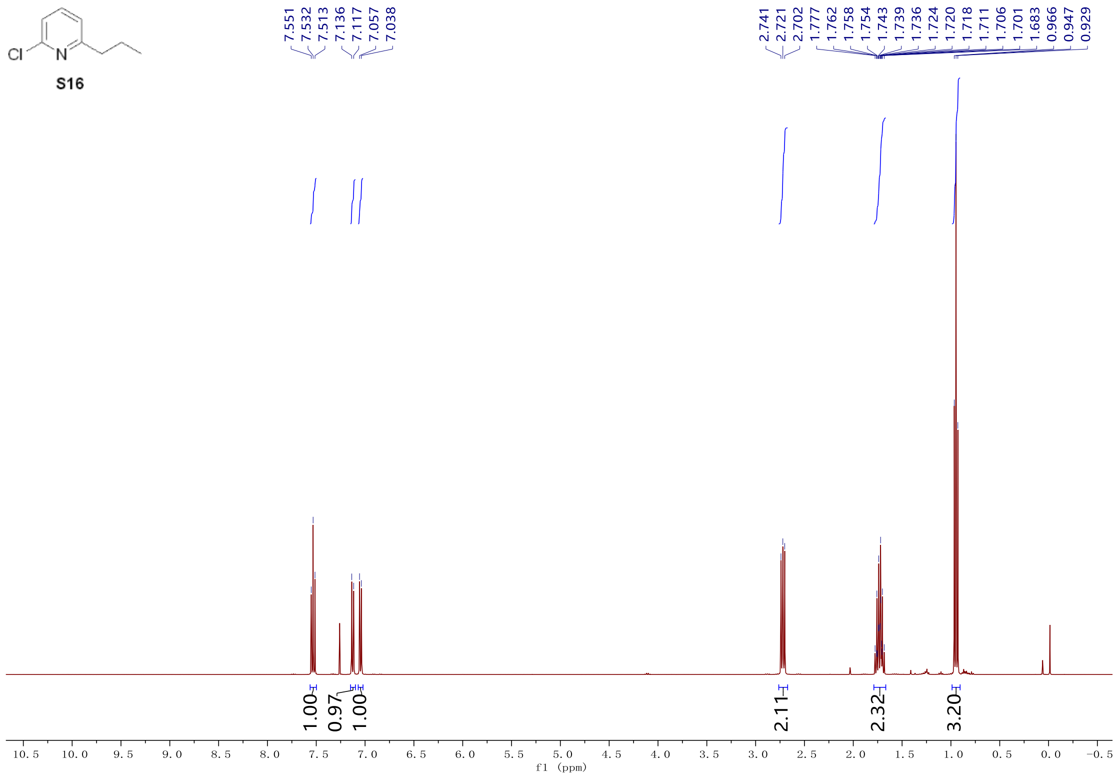

Supplementary Figure 63. ^{1}H NMR spectrum of compound **S16**

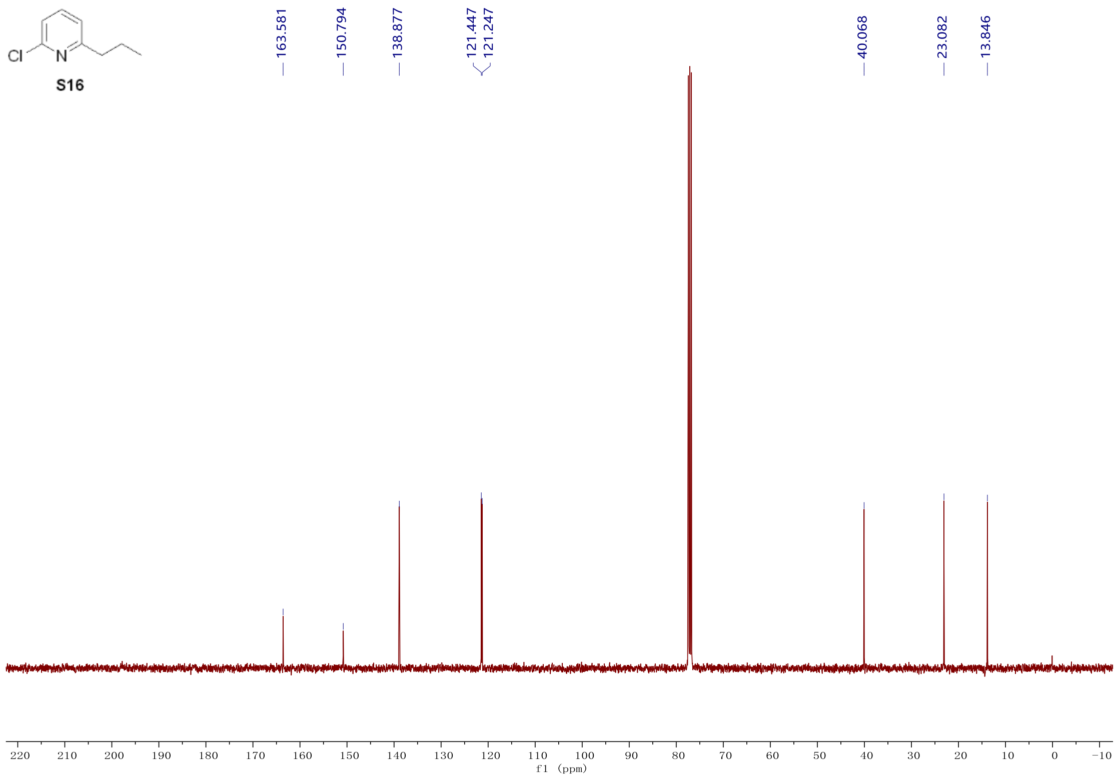

Supplementary Figure 64. ^{13}C NMR spectrum of compound **S16**

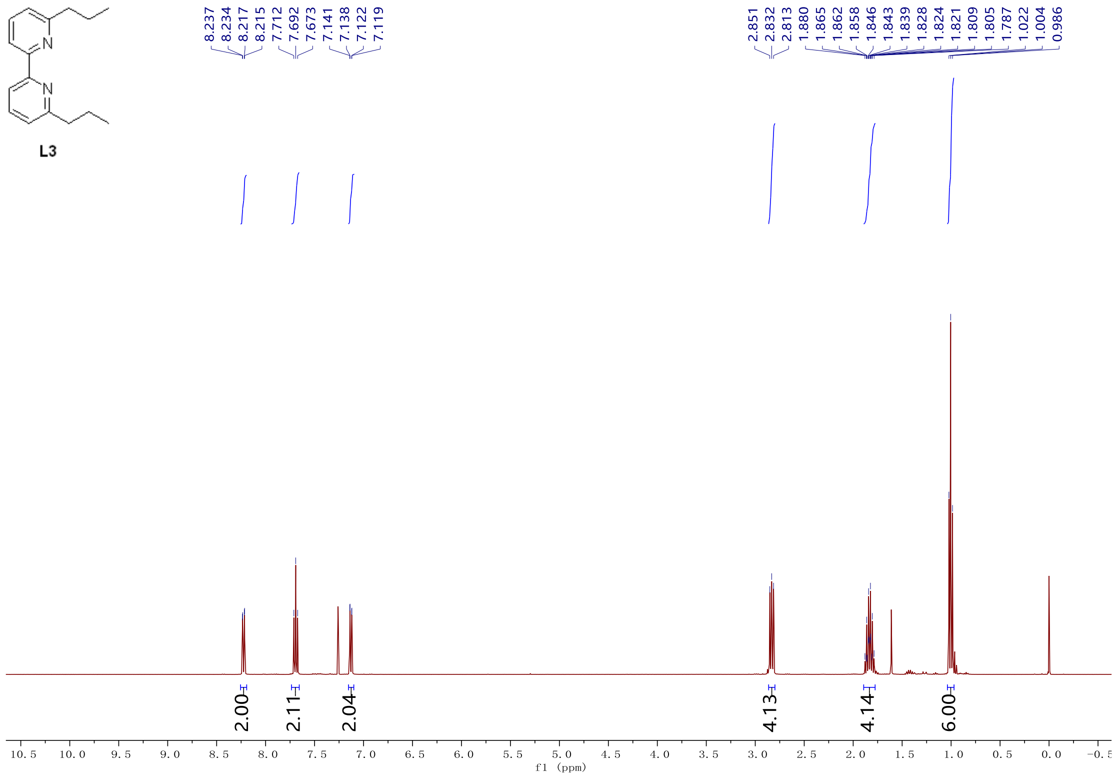

Supplementary Figure 65. ^{1}H NMR spectrum of compound **L3**

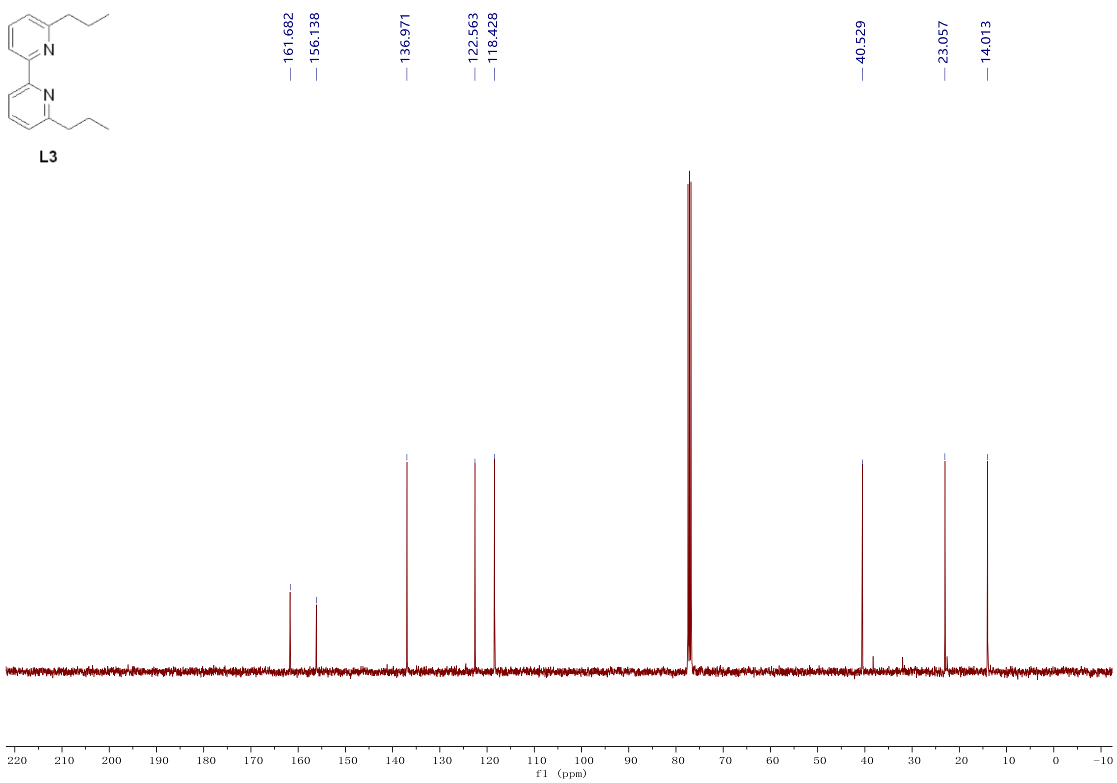

Supplementary Figure 66. ^{13}C NMR spectrum of compound **L3**

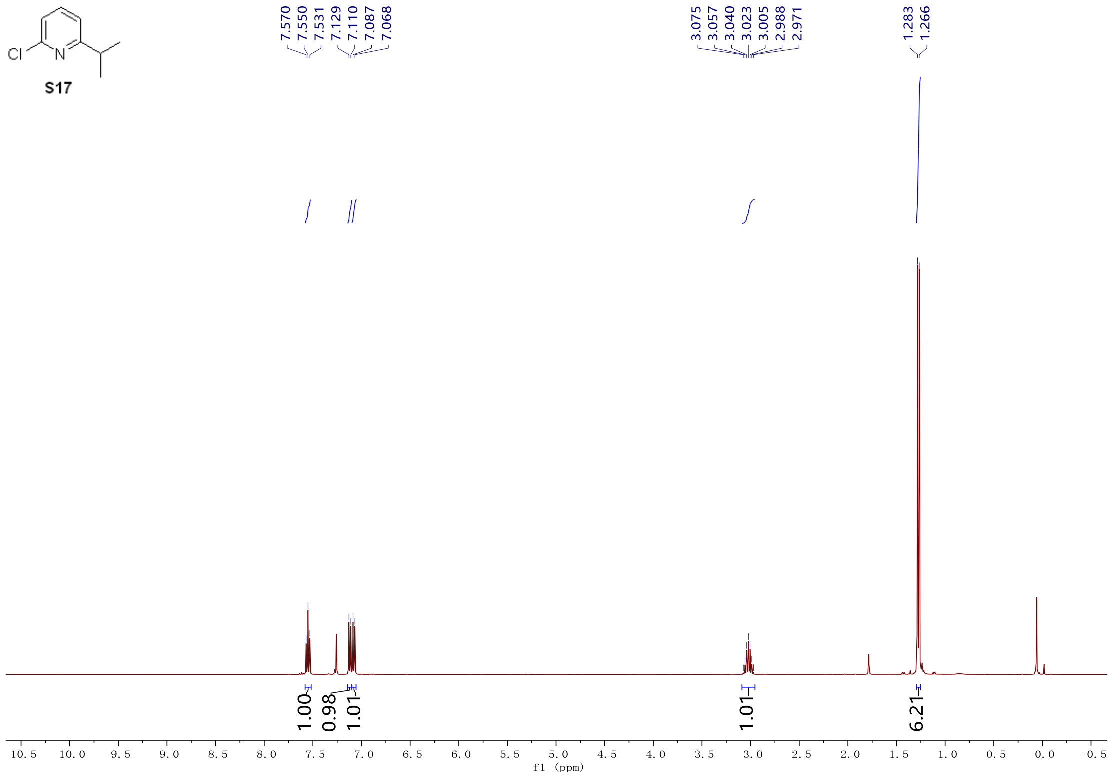

Supplementary Figure 67. ^{1}H NMR spectrum of compound **S17**

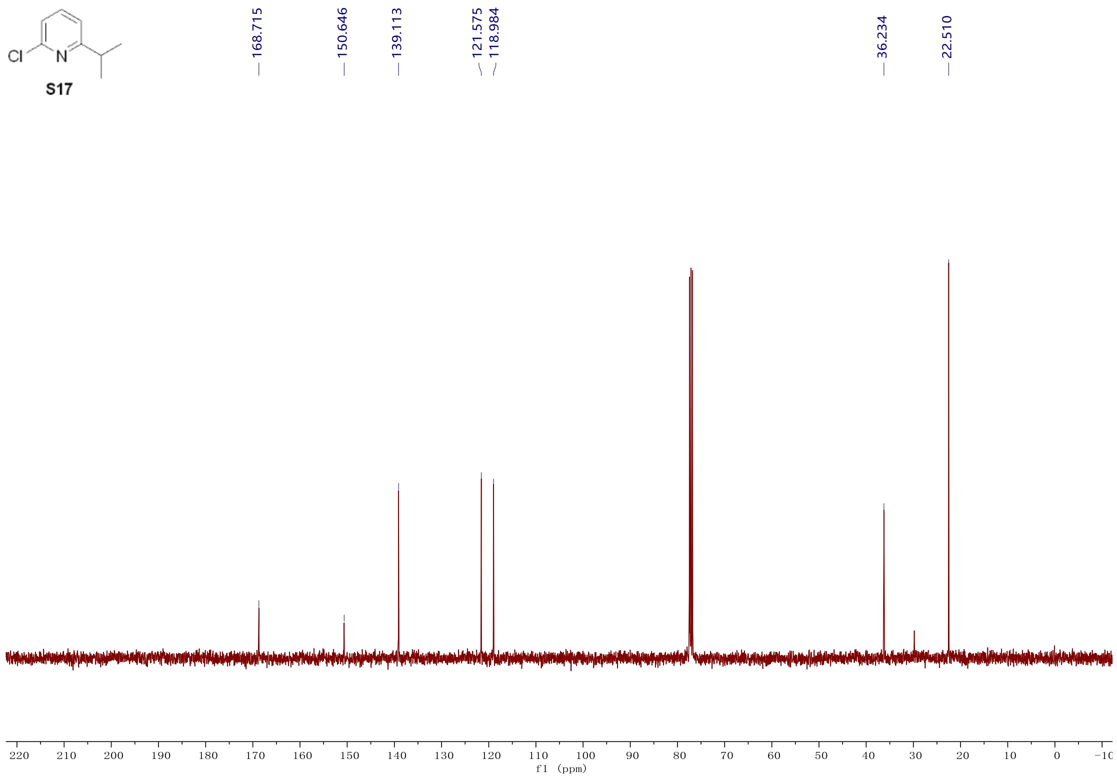

Supplementary Figure 68. ^{13}C NMR spectrum of compound **S17**

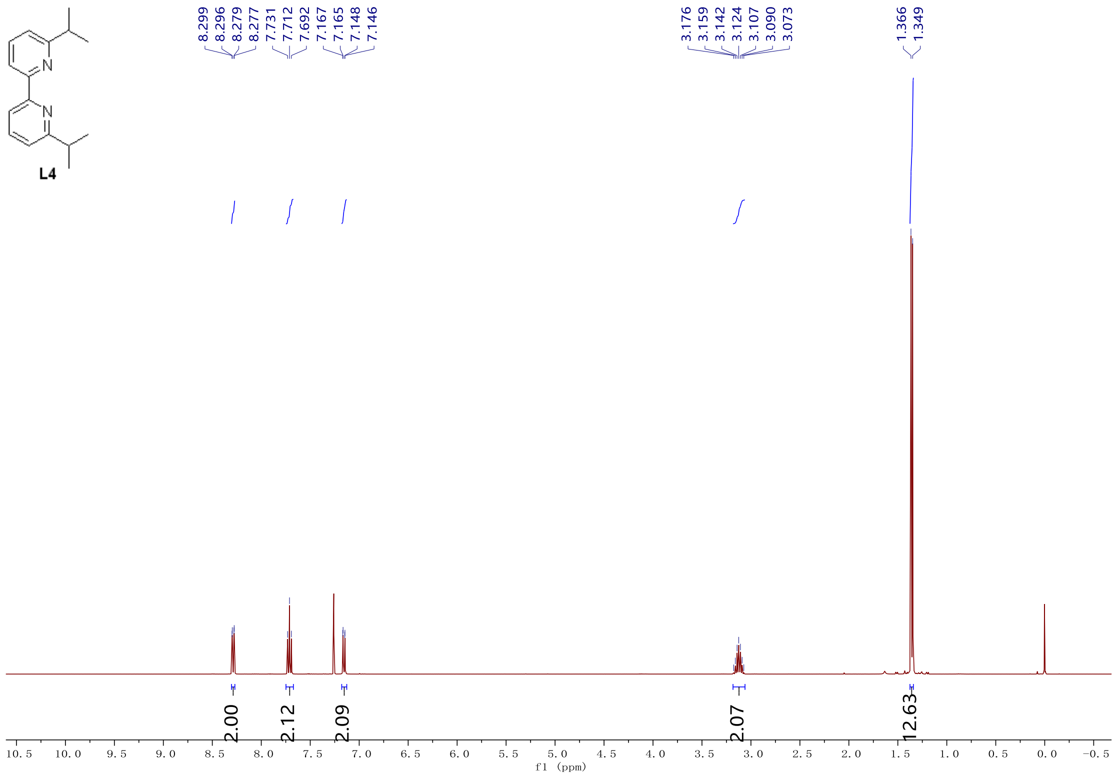

Supplementary Figure 69. ^{1}H NMR spectrum of compound **L4**

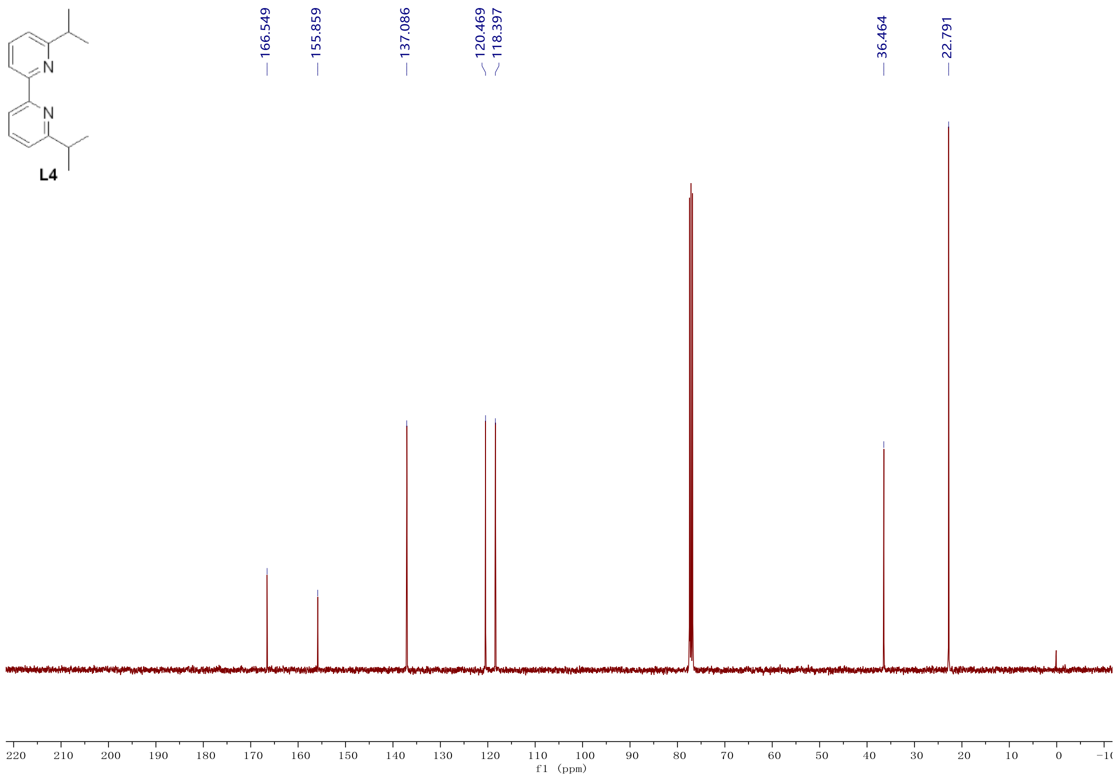

Supplementary Figure 70. ^{13}C NMR spectrum of compound **L4**

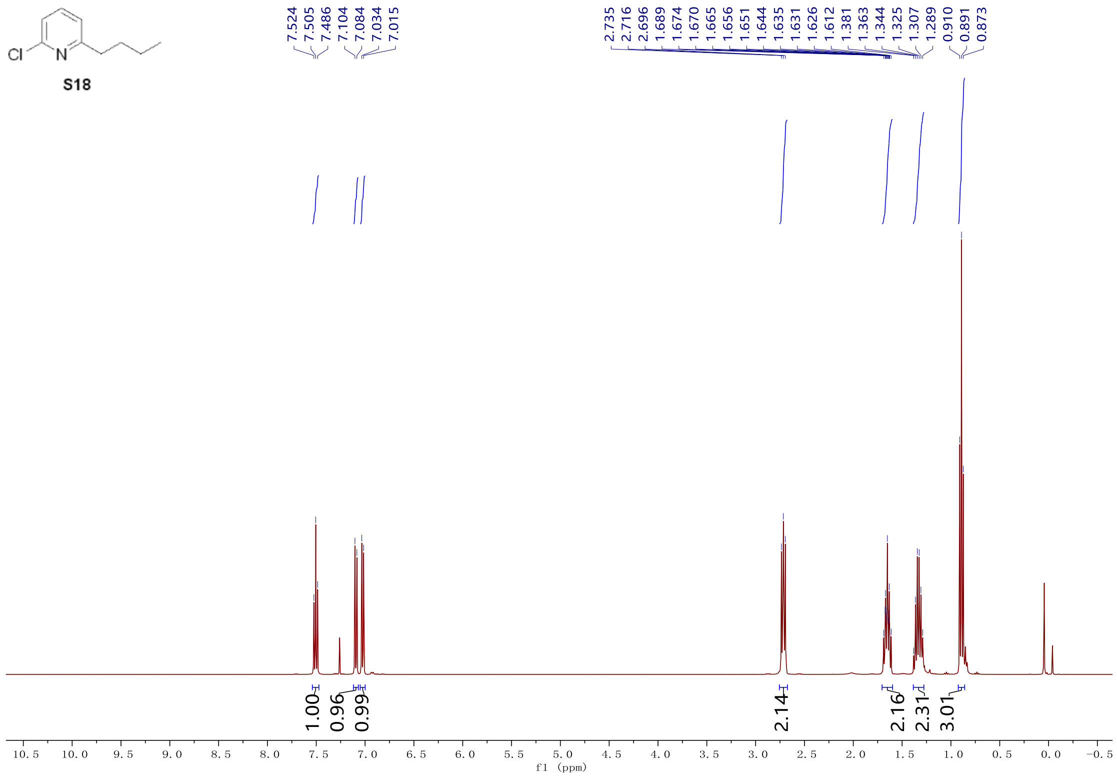

Supplementary Figure 71. ^{1}H NMR spectrum of compound **S18**

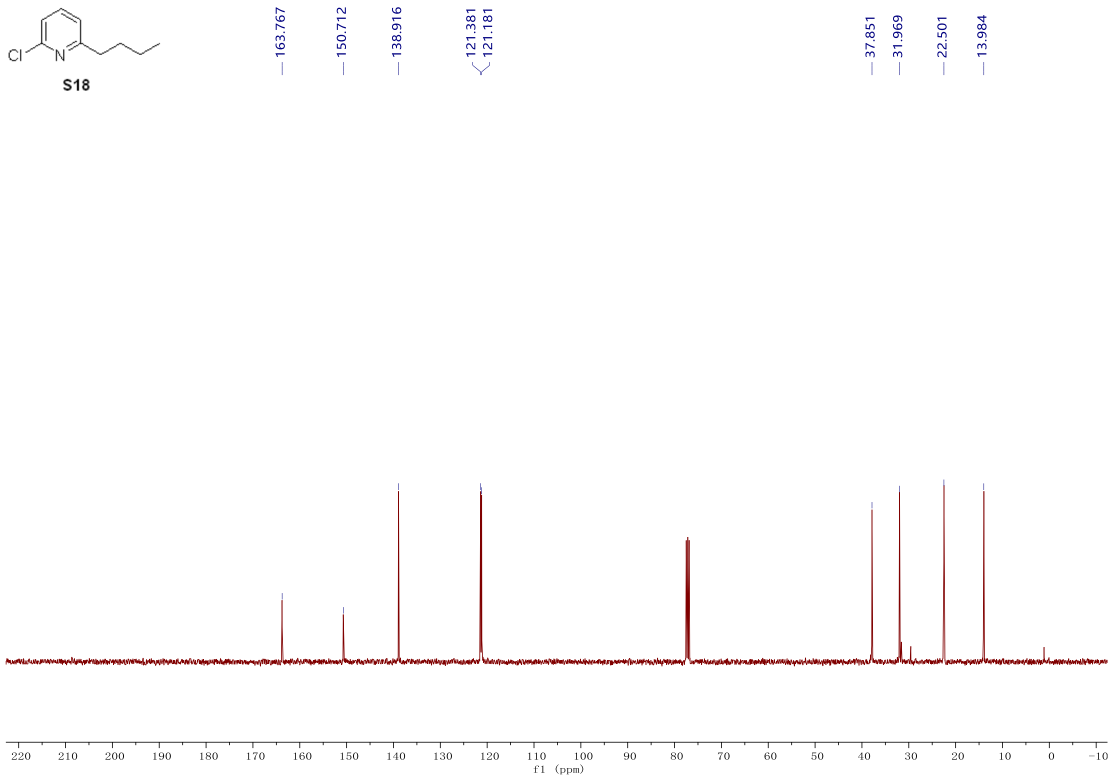

Supplementary Figure 72. ^{13}C NMR spectrum of compound **S18**

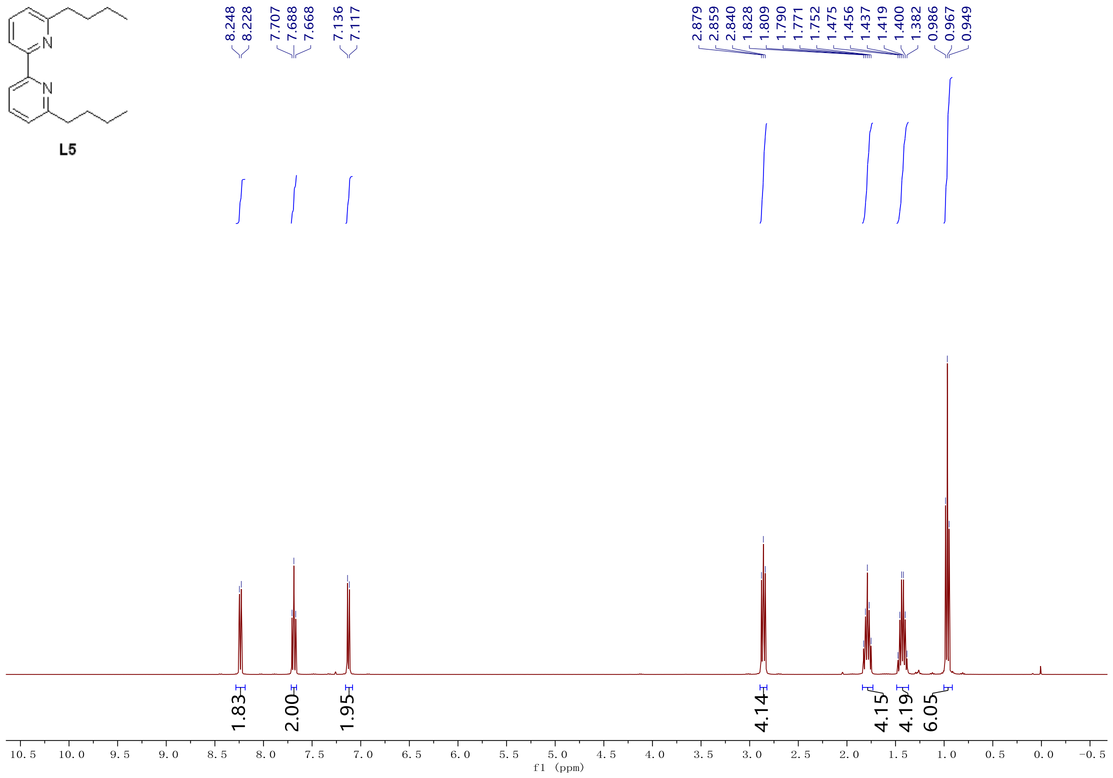

Supplementary Figure 73. ^{1}H NMR spectrum of compound **L5**

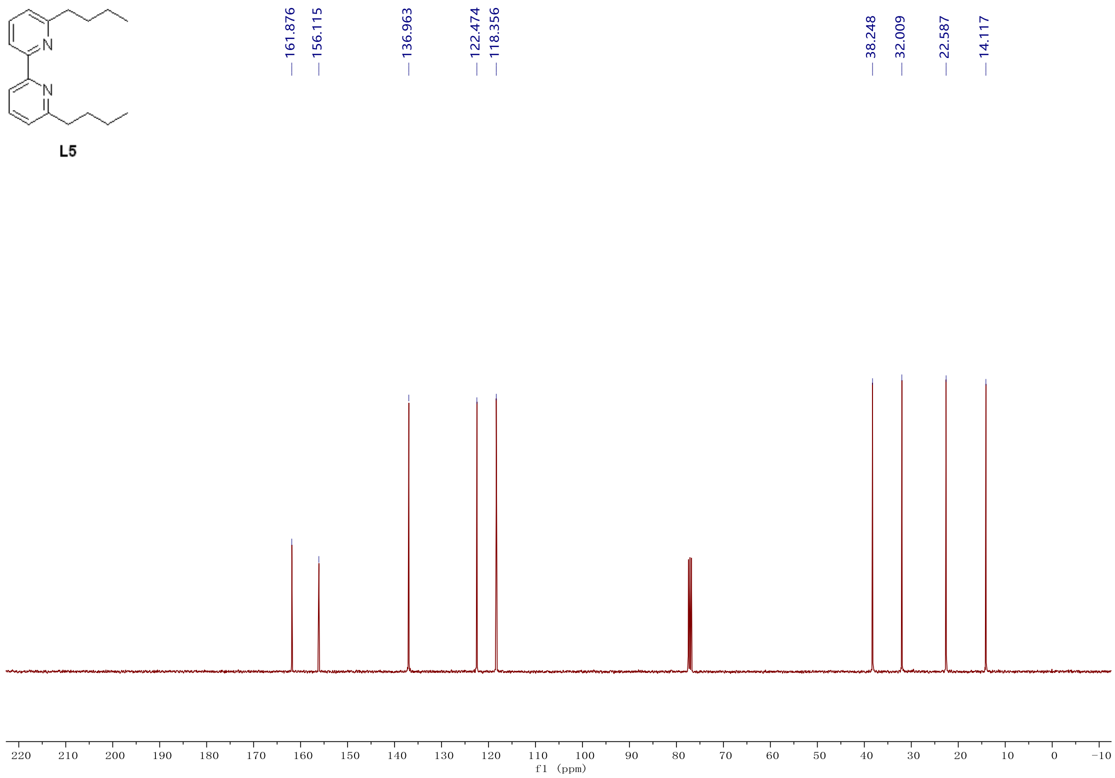

Supplementary Figure 74. ^{13}C NMR spectrum of compound **L5**

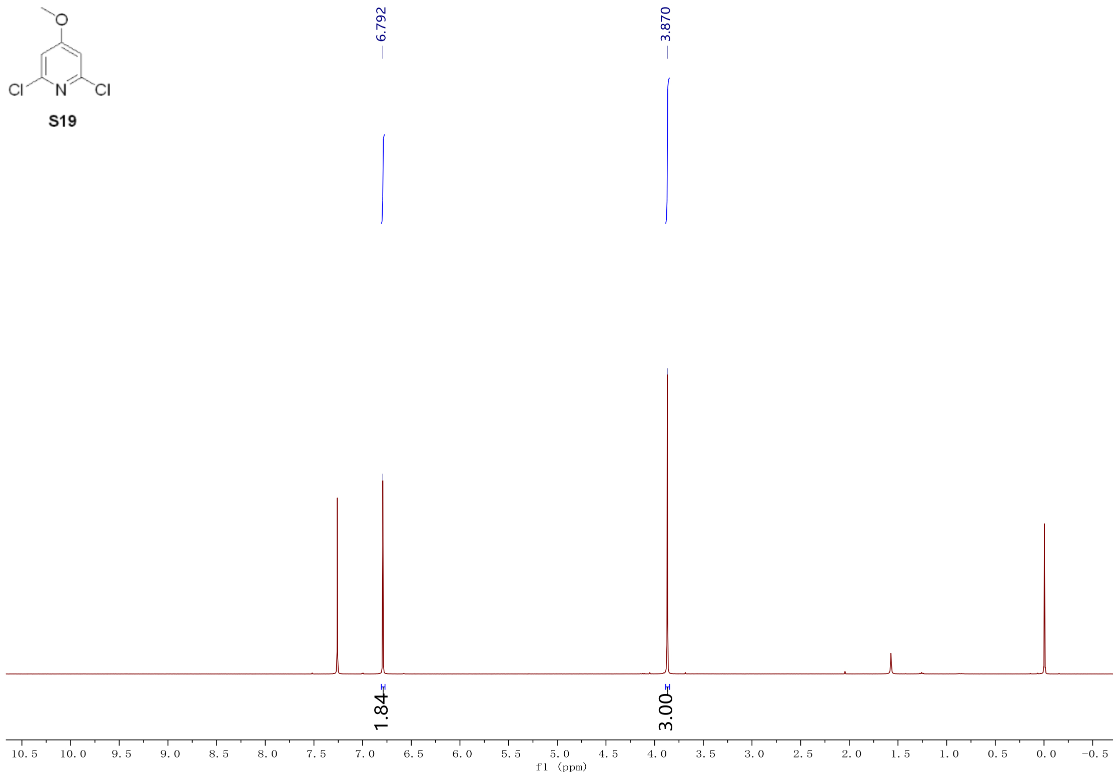

Supplementary Figure 75. ^{1}H NMR spectrum of compound **S19**

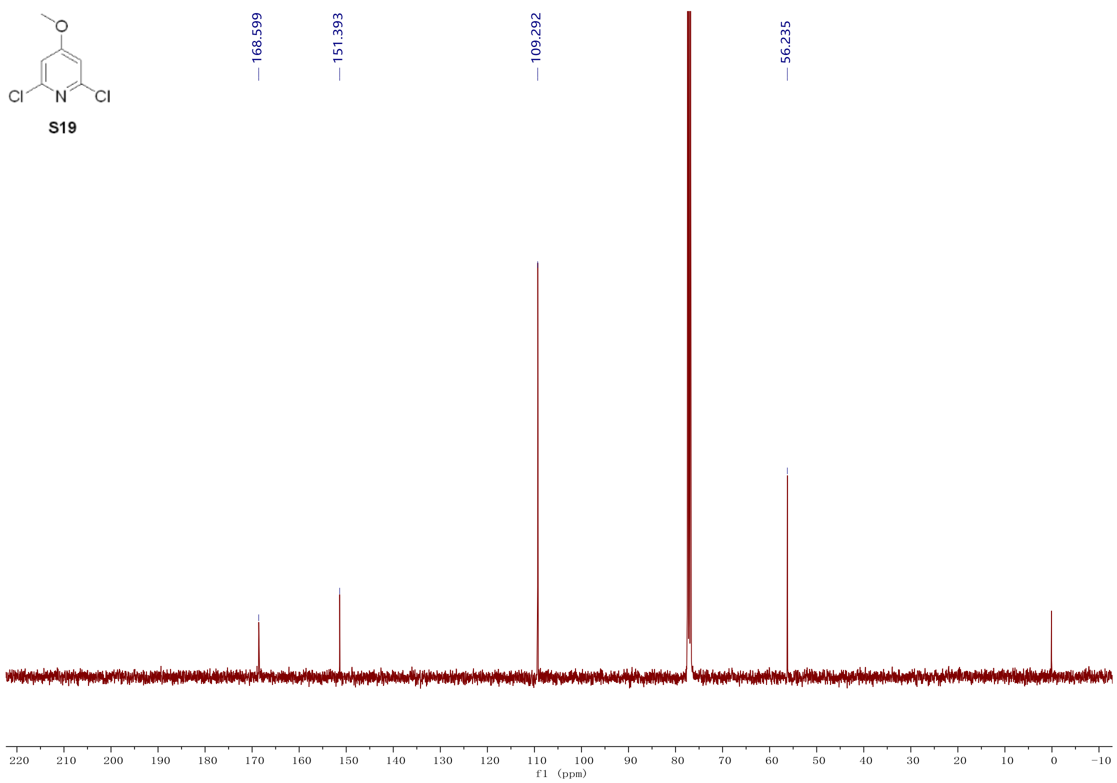

Supplementary Figure 76. ^{13}C NMR spectrum of compound **S19**

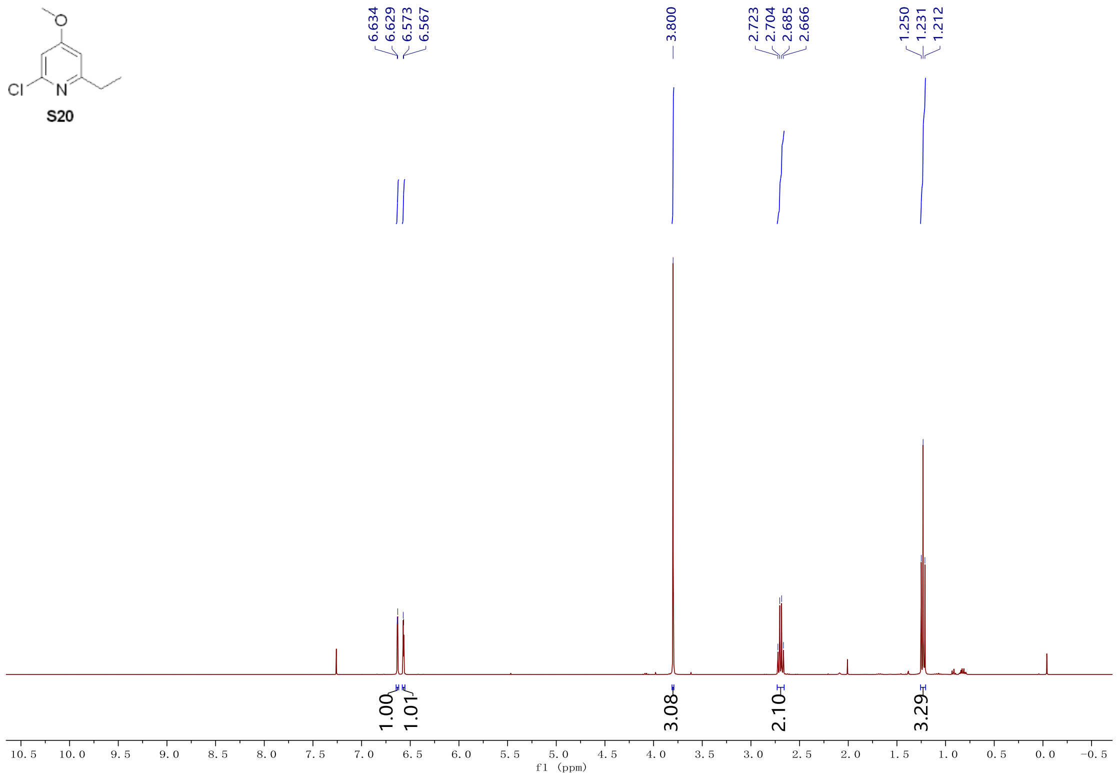

Supplementary Figure 77. ^{1}H NMR spectrum of compound **S20**

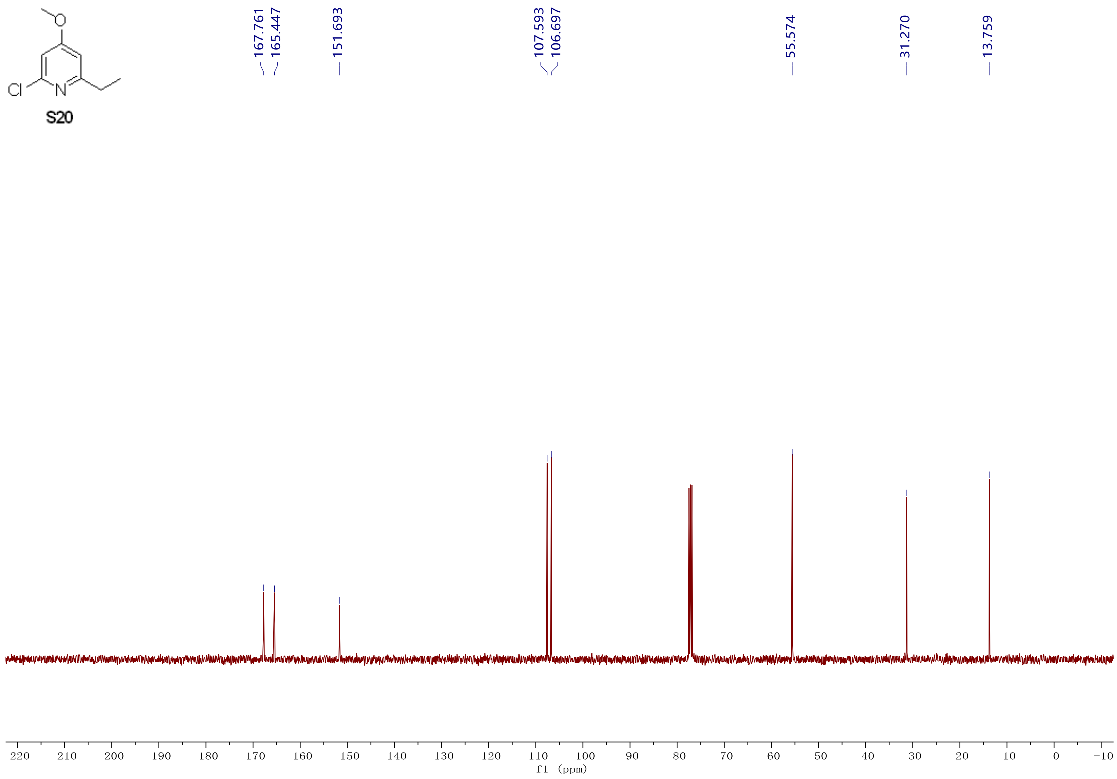

Supplementary Figure 78. ^{13}C NMR spectrum of compound **S20**

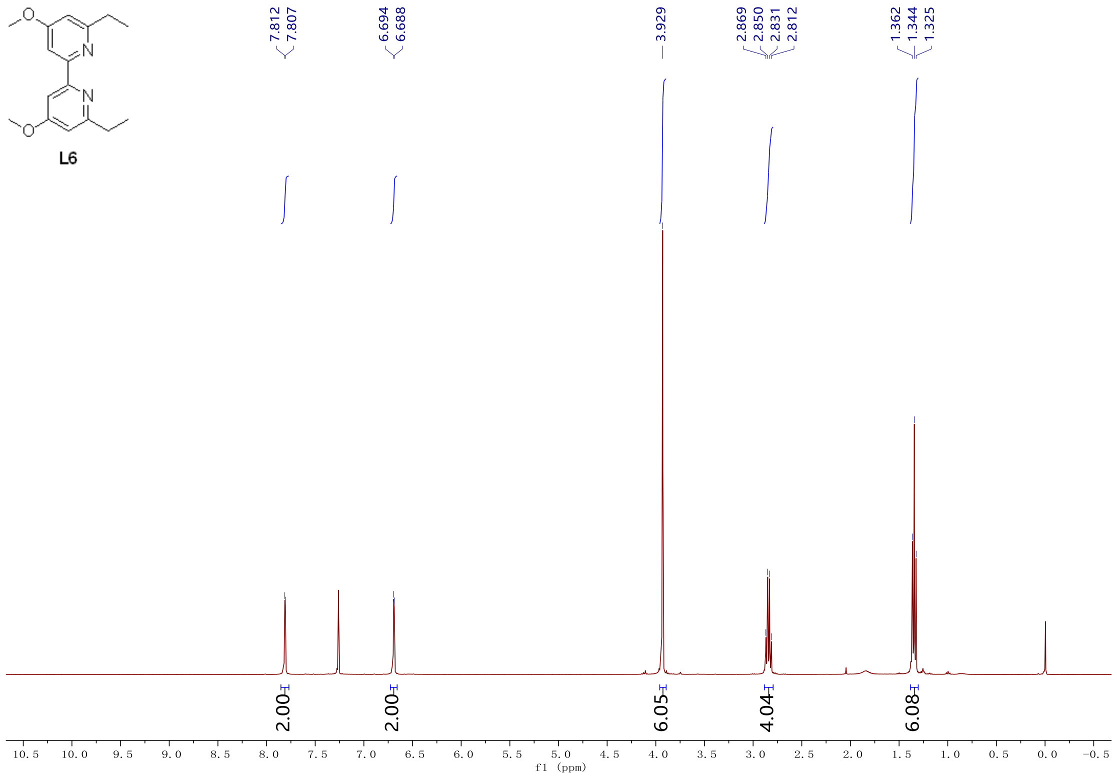

Supplementary Figure 79. ^{1}H NMR spectrum of compound **L6**

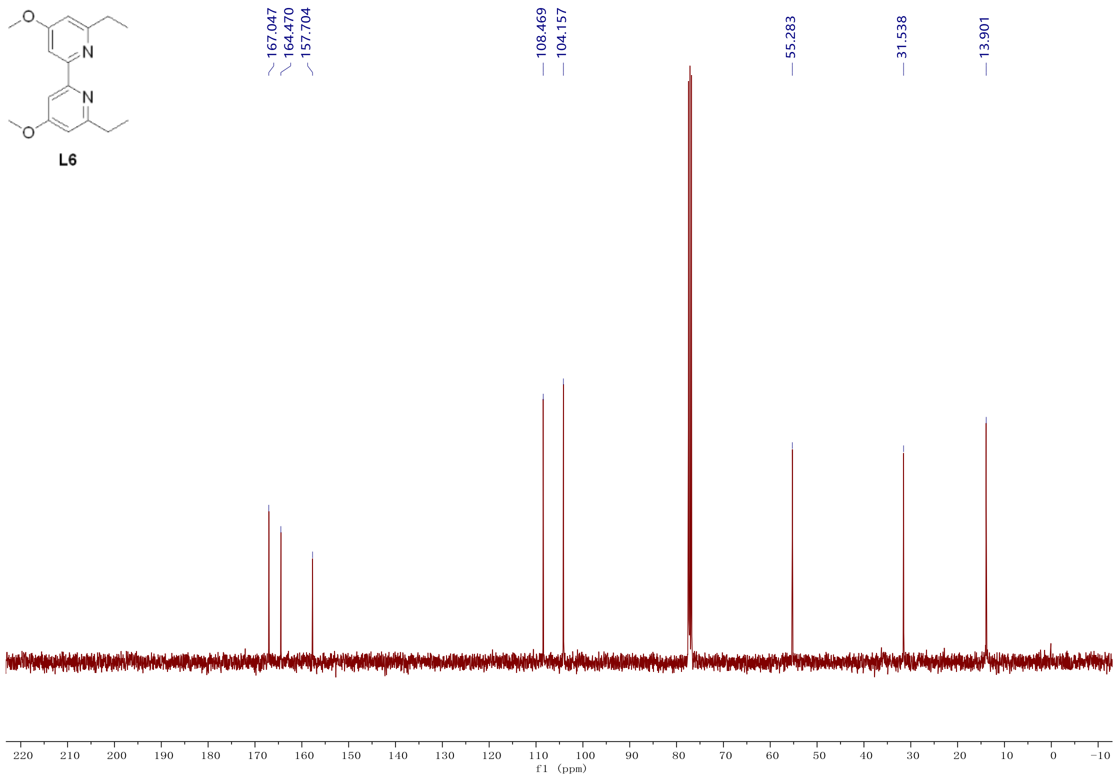

Supplementary Figure 80. ^{13}C NMR spectrum of compound **L6**

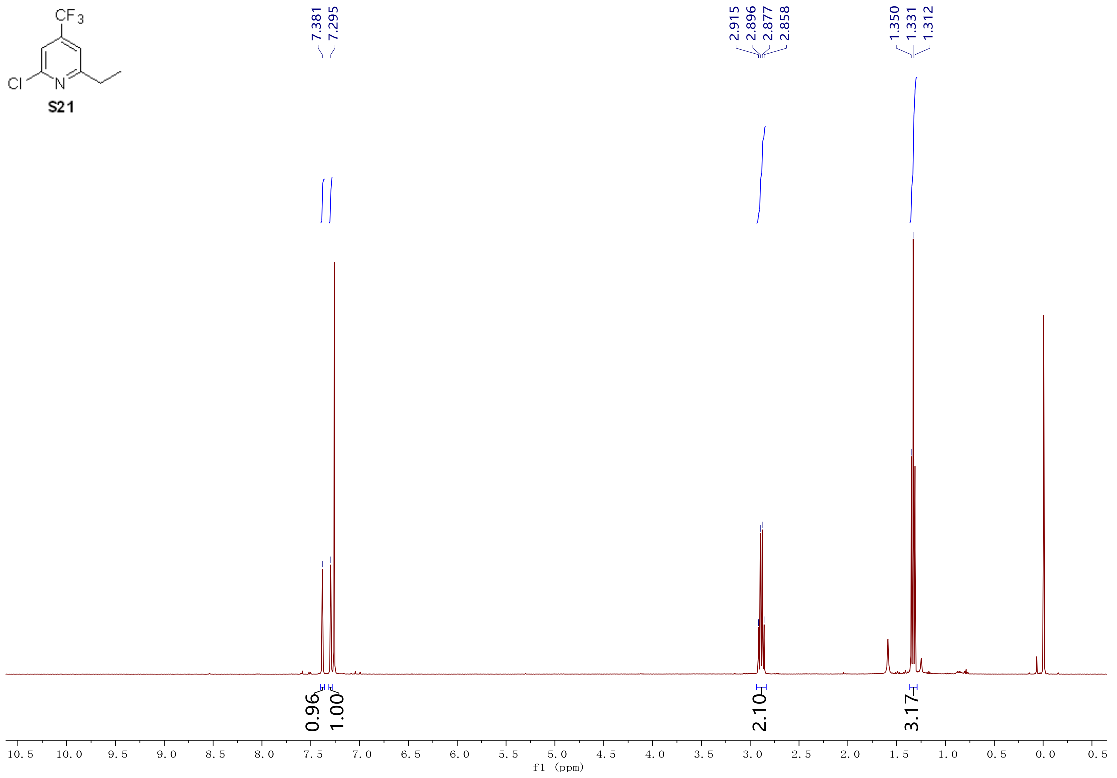

Supplementary Figure 81. ^{1}H NMR spectrum of compound **S21**

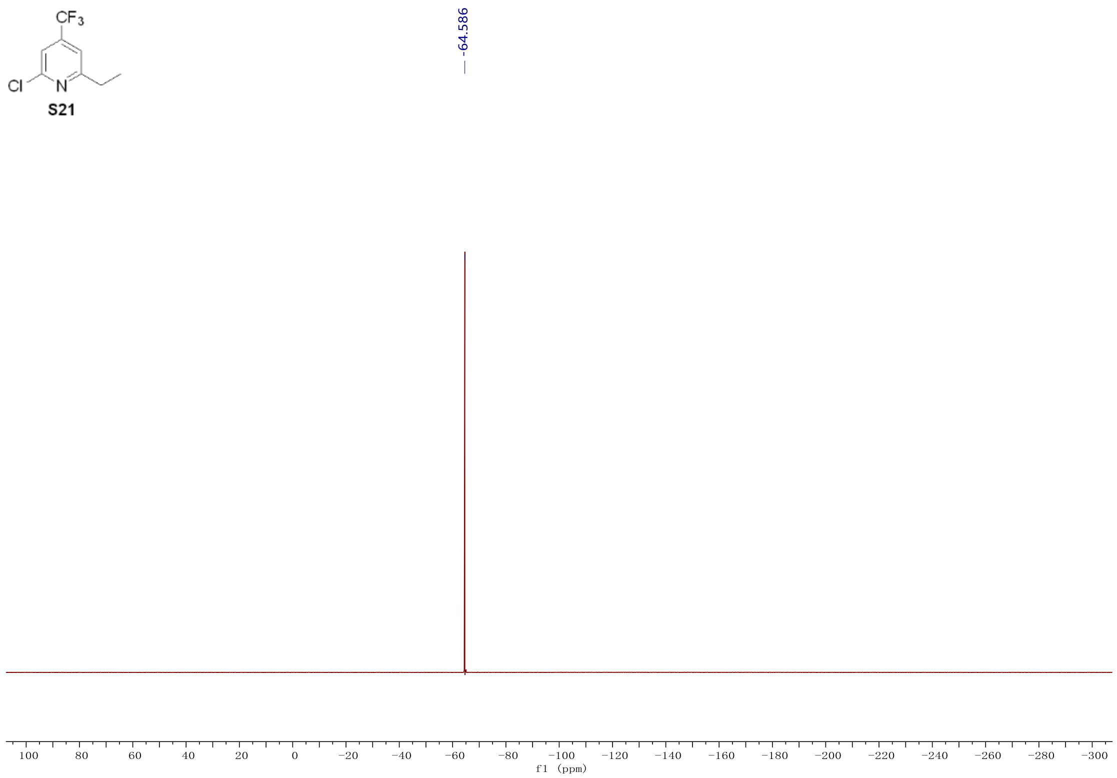

Supplementary Figure 82. ^{19}F NMR spectrum of compound **S21**

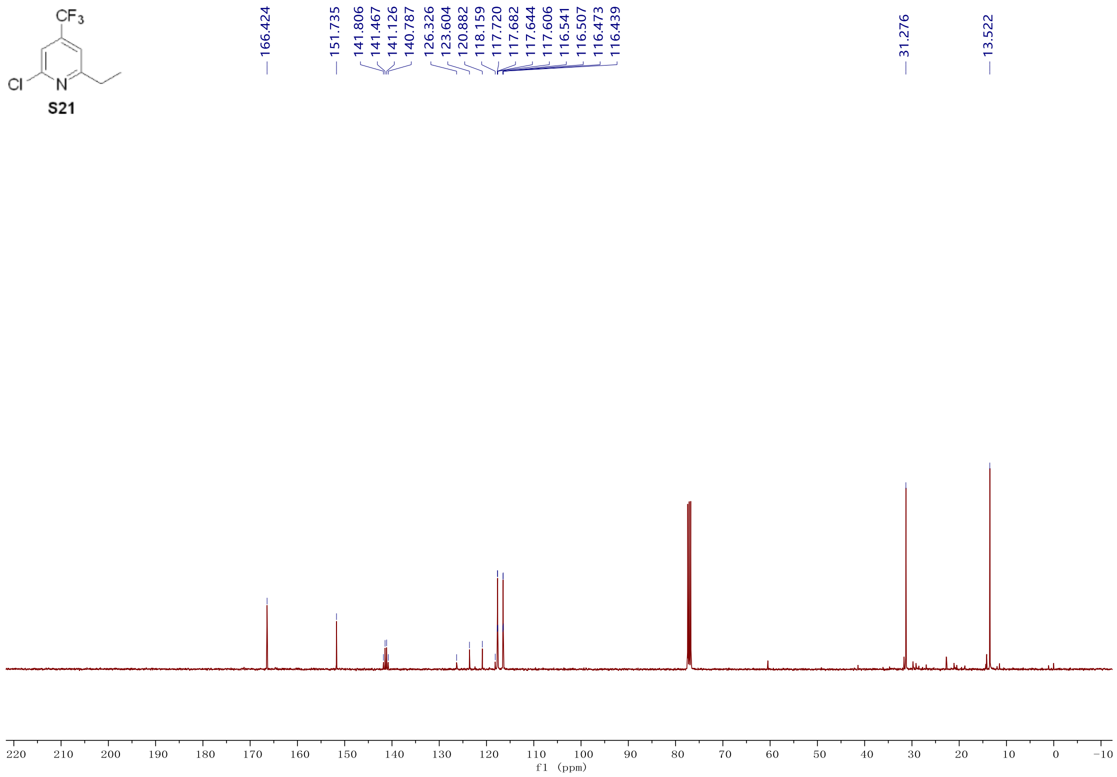

Supplementary Figure 83. ^{13}C NMR spectrum of compound **S21**

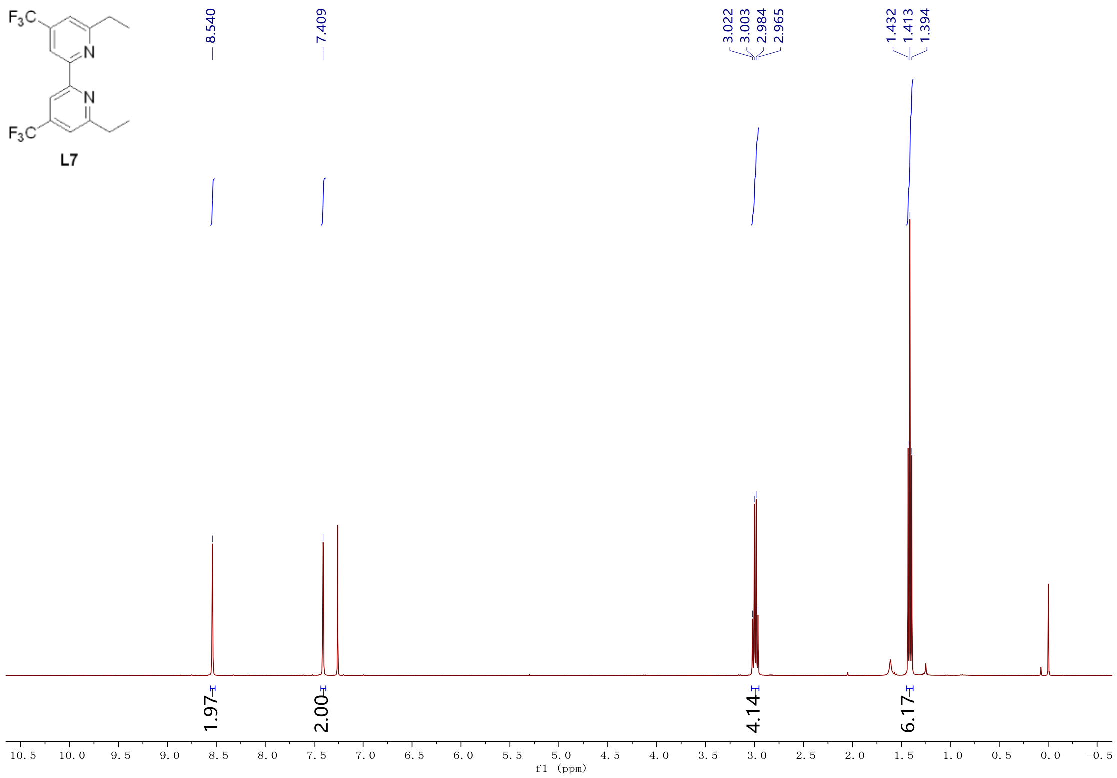

Supplementary Figure 84. ^{1}H NMR spectrum of compound **L7**

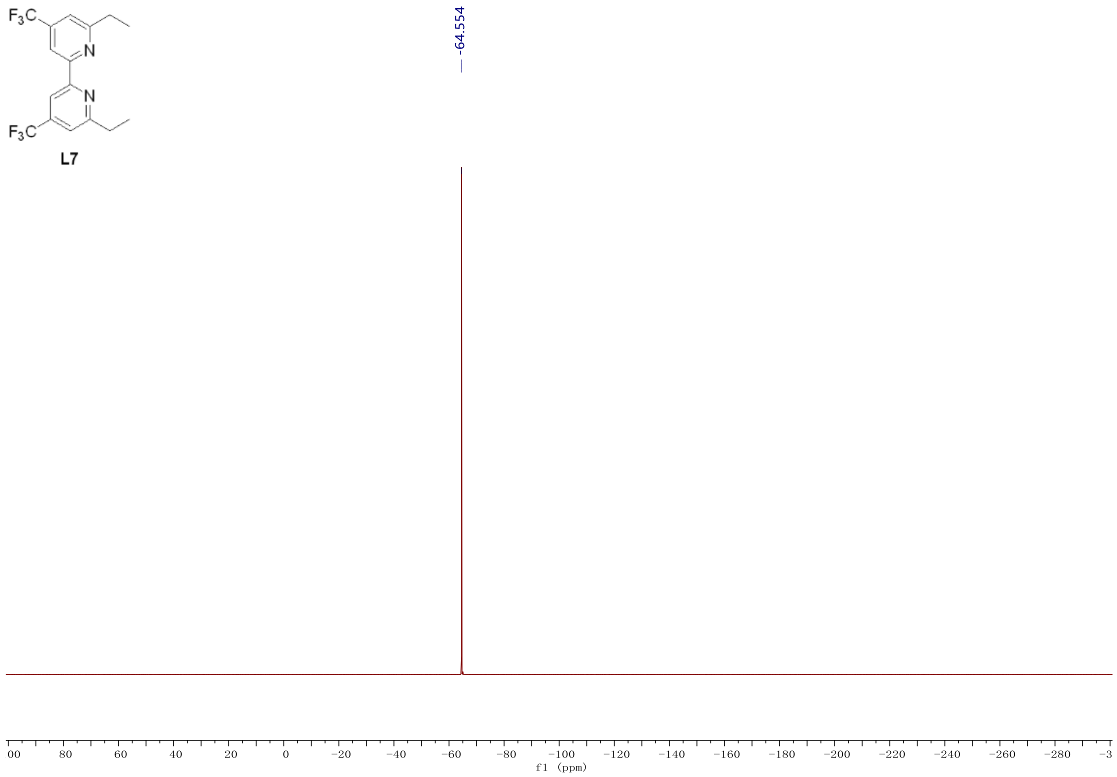

Supplementary Figure 85. ^{19}F NMR spectrum of compound **L7**

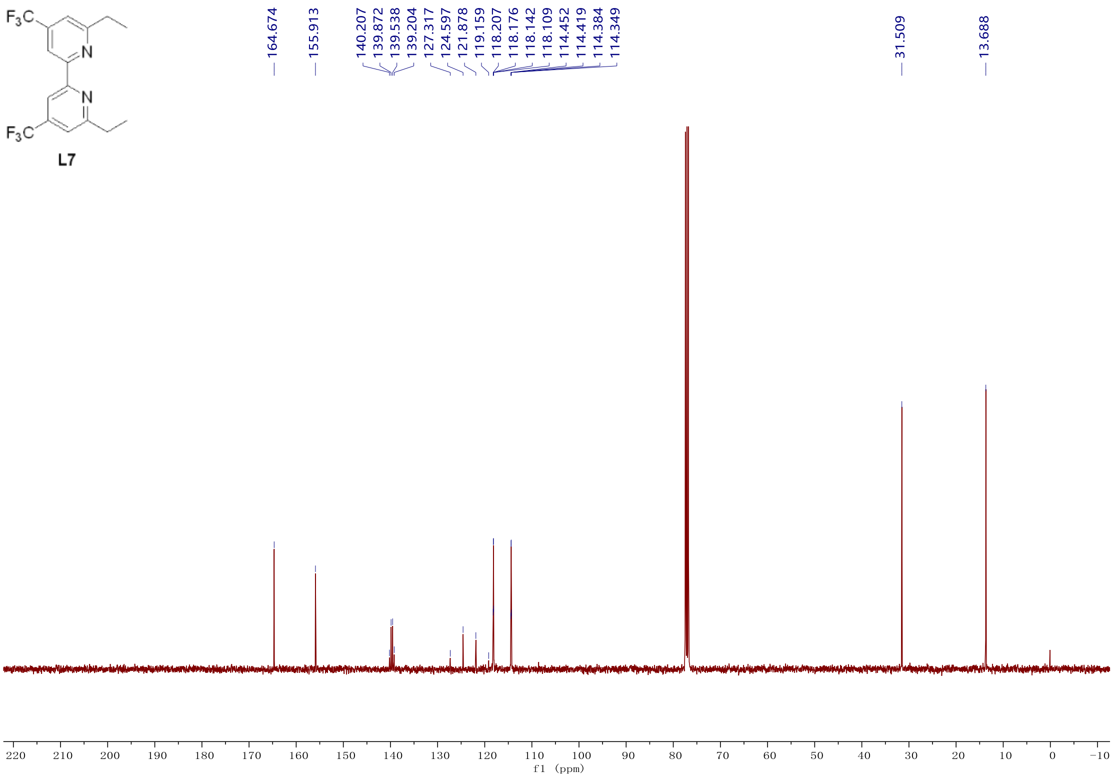

Supplementary Figure 86. ^{13}C NMR spectrum of compound **L7**

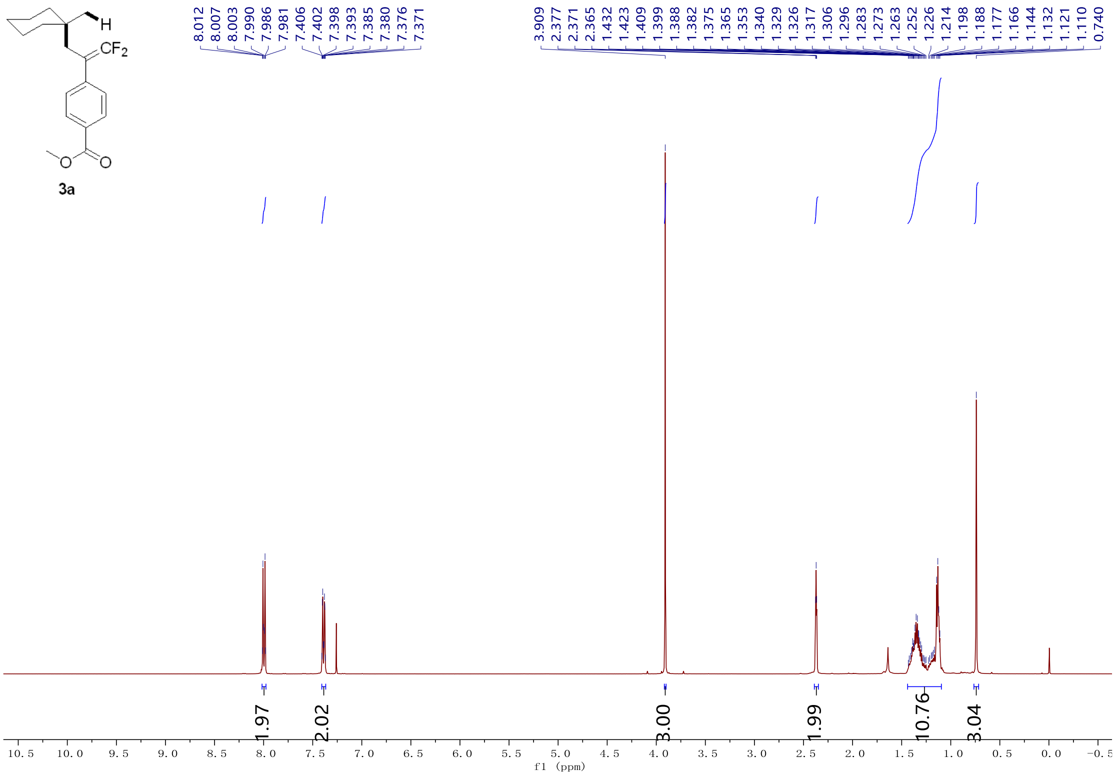

Supplementary Figure 87. ^{1}H NMR spectrum of compound **3a**

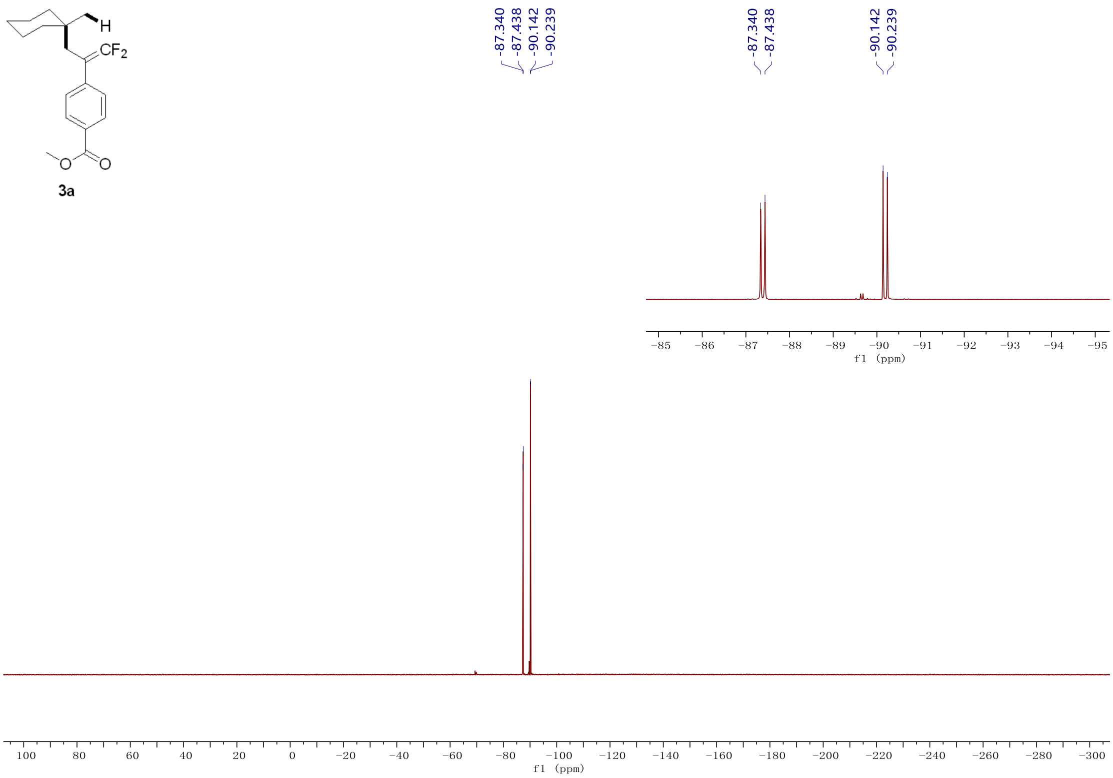

Supplementary Figure 88. ^{19}F NMR spectrum of compound **3a**

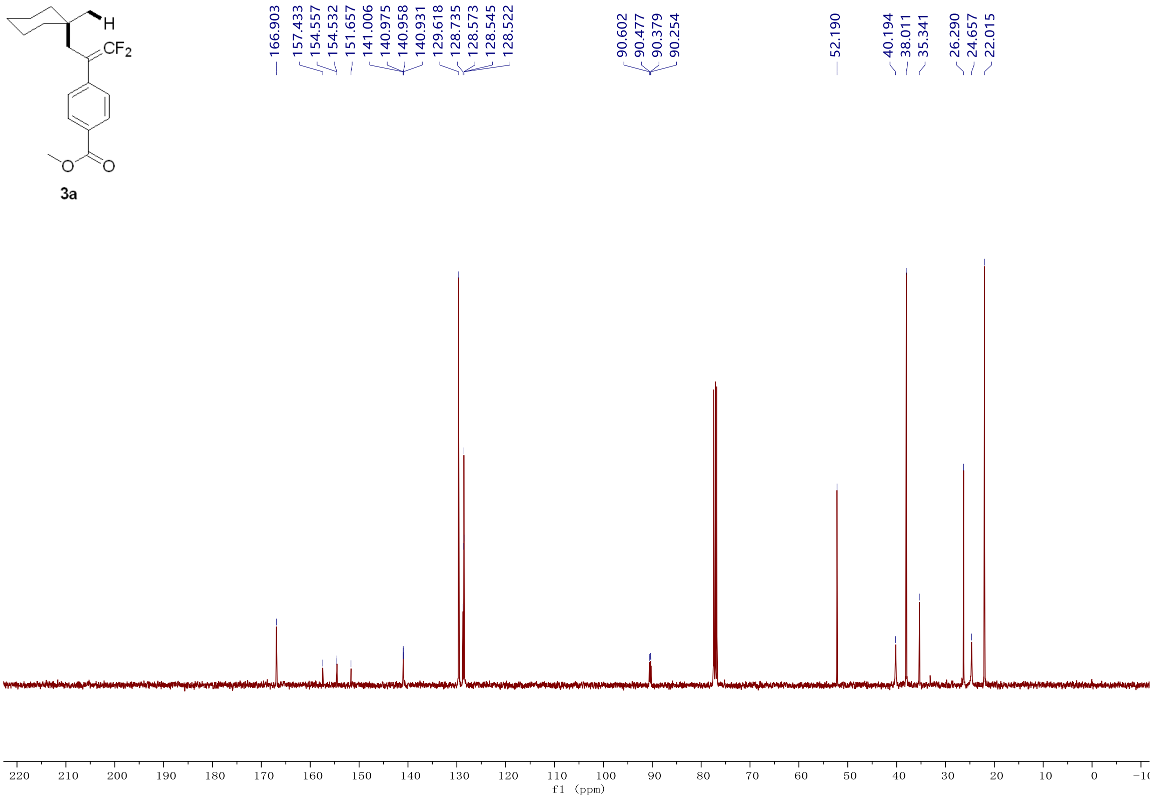

Supplementary Figure 89. ^{13}C NMR spectrum of compound **3a**

Determination of regioisomeric ratio by ^{19}F NMR

To identify the regioisomeric ratio of product obtained from the present reaction, the ipso-difluoroallylated product **3a'** was isolated. The rr of **3a** was calculated by comparing the integral of respective peak in the crude ^{19}F NMR.

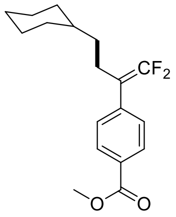

3a'

^{1}H NMR (400 MHz, CDCl$_3$): δ = 8.03 – 8.00 (m, 2H), 7.40 – 7.36 (m, 2H), 3.92 (s, 3H), 2.45 – 2.39 (m, 2H), 1.71 – 1.60 (m, 5H), 1.25 – 1.08 (m, 6H), 0.91 – 0.79 (m, 2H). **^{19}F NMR (376 MHz, CDCl$_3$):** δ = -89.57 (d, *J* = 39.7 Hz), -89.73 (d, *J* = 40.0 Hz). **^{13}C NMR (100 MHz, CDCl$_3$):** δ = 166.9, 153.7 (dd, *J* = 291.7, 288.2 Hz), 138.96 – 138.85 (m), 129.7, 128.9, 128.2 (t, *J* = 3.4 Hz), 92.6 (dd, *J* = 21.3, 13.0 Hz), 52.2, 37.3, 35.6, 33.1, 29.8, 26.7, 26.3, 24.9.

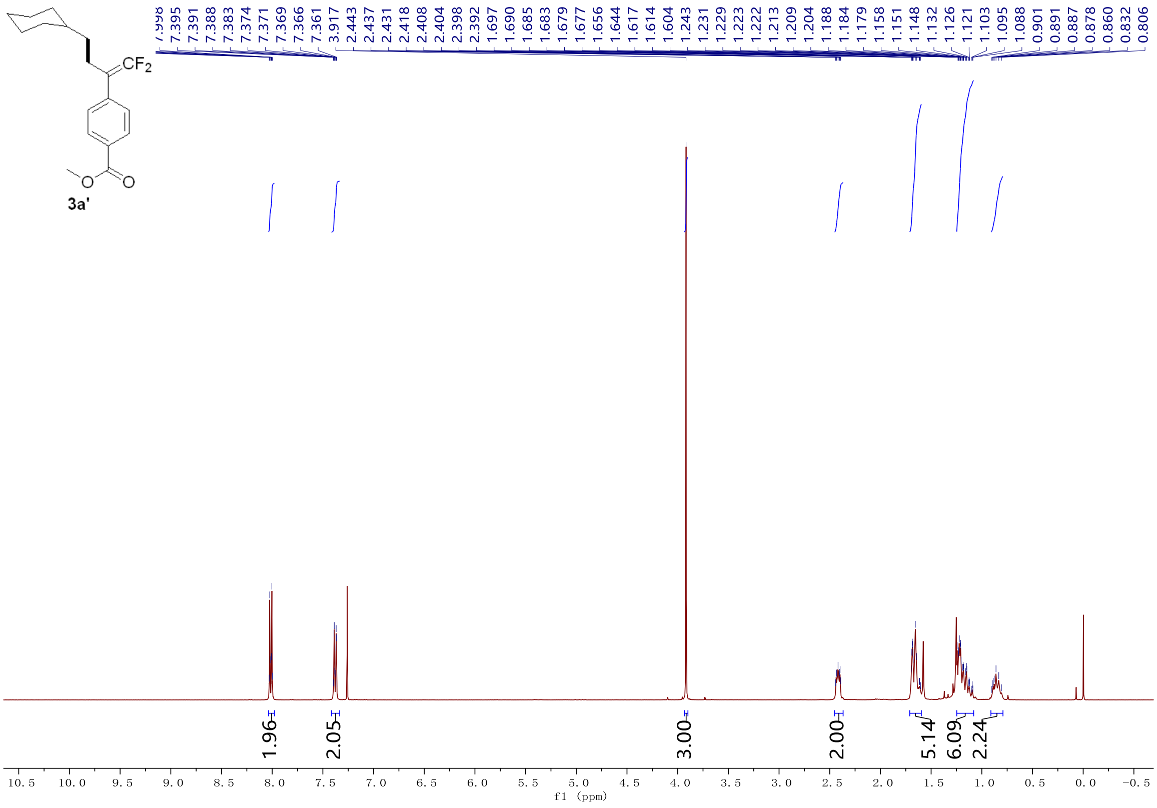

Supplementary Figure 90. ^{1}H NMR spectrum of compound **3a'**

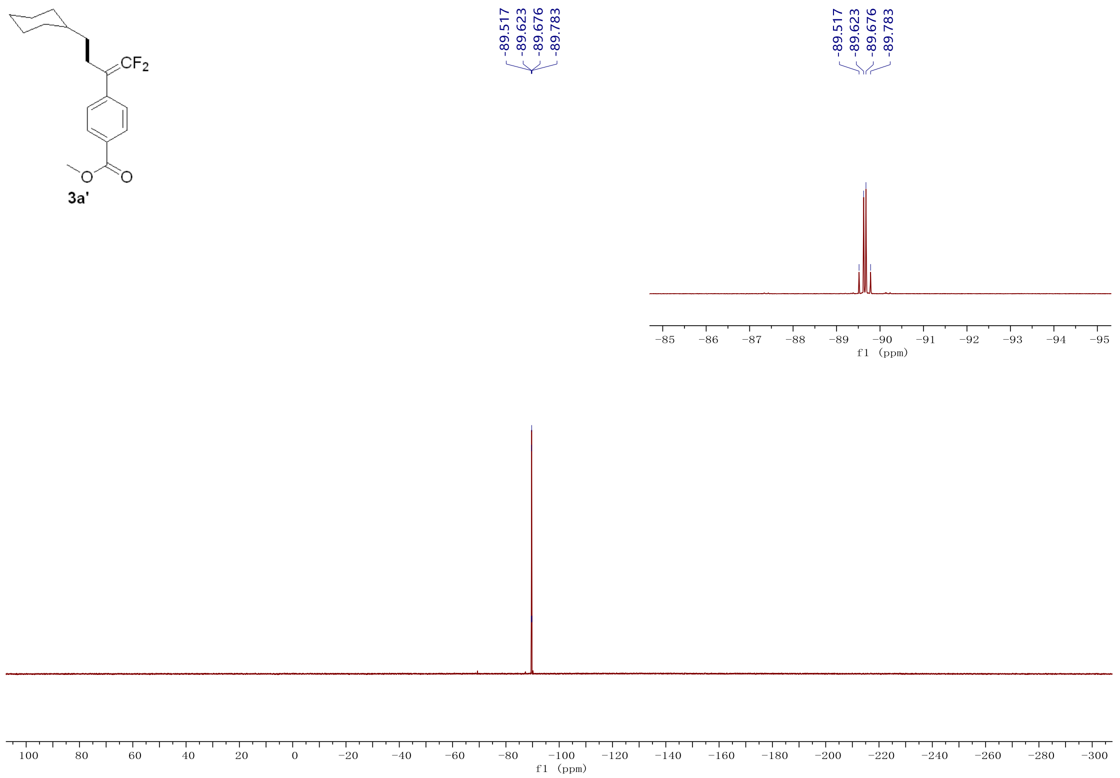

Supplementary Figure 91. ^{19}F NMR spectrum of compound **3a’**

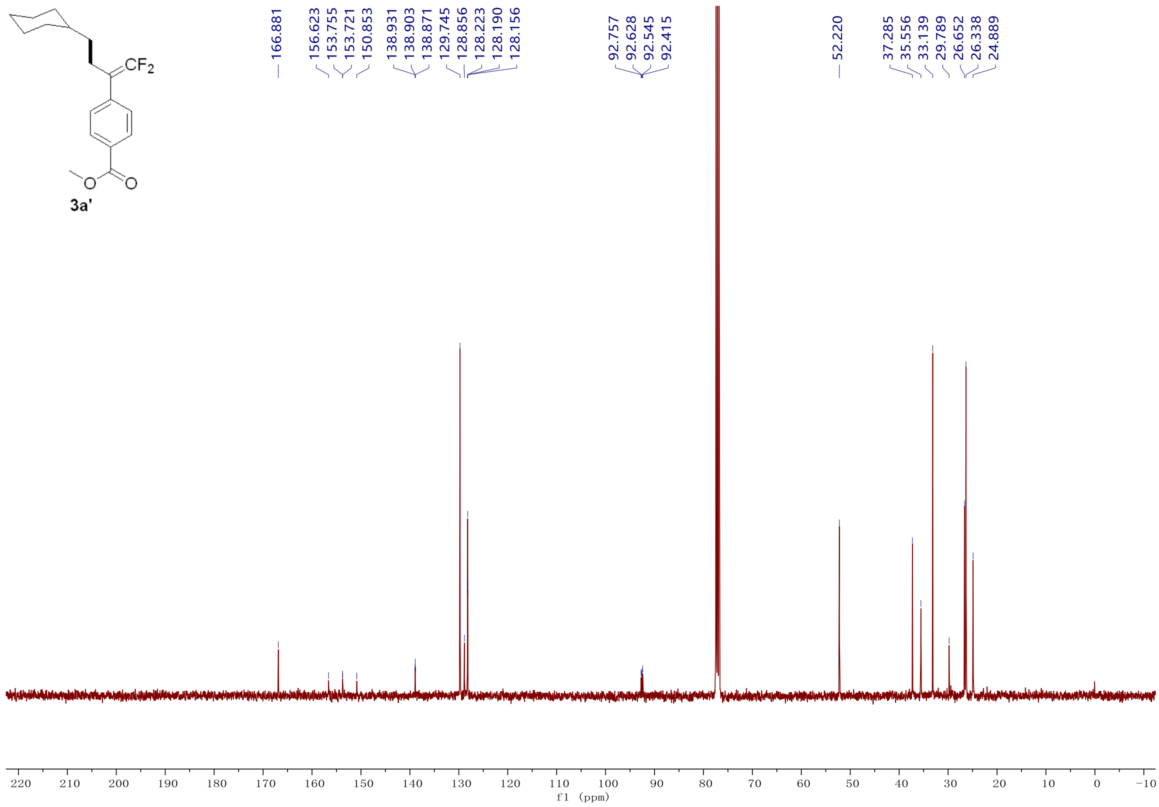

Supplementary Figure 92. ^{13}C NMR spectrum of compound **3a’**

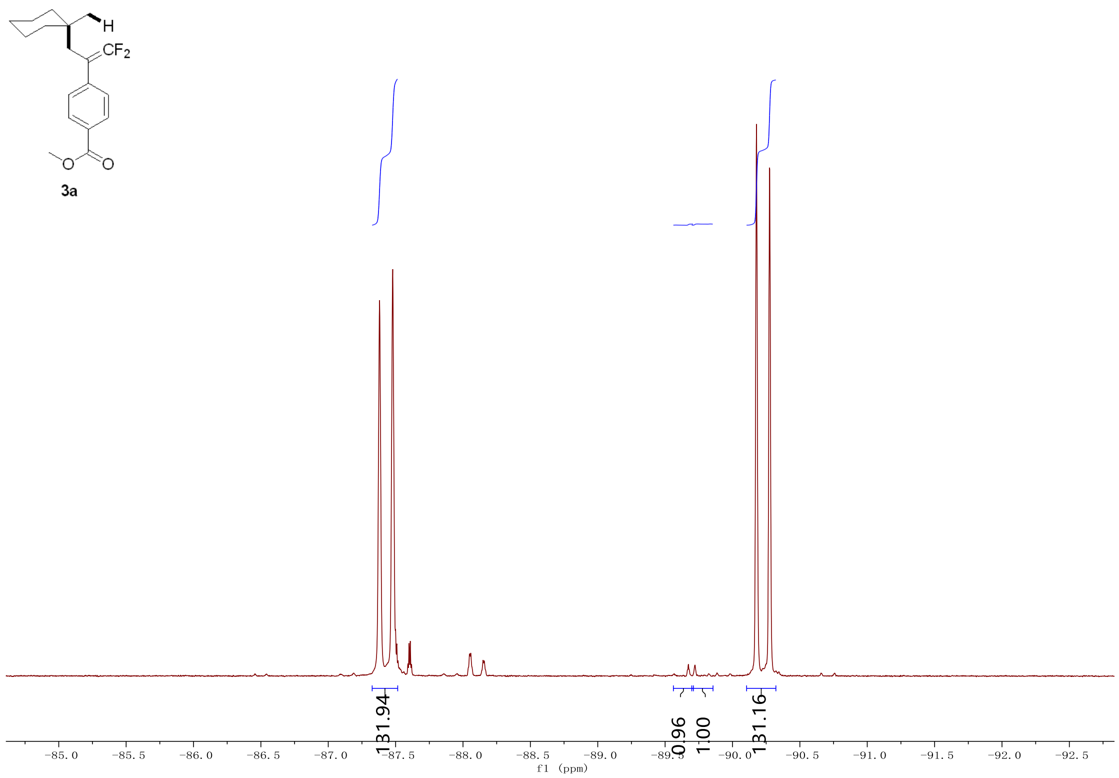

Supplementary Figure 93. Crude ^{19}F NMR spectrum of compound **3a**

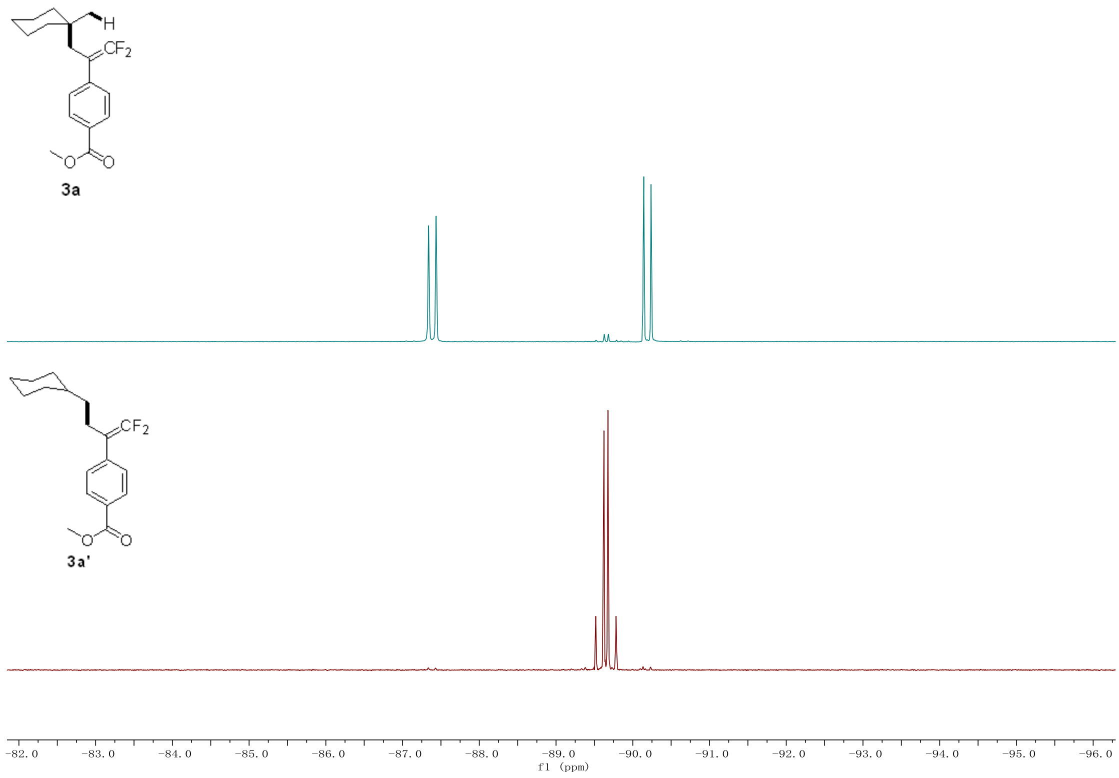

Supplementary Figure 94. Comparison of ^{19}F NMR spectrum of compound **3a** and **3a'**

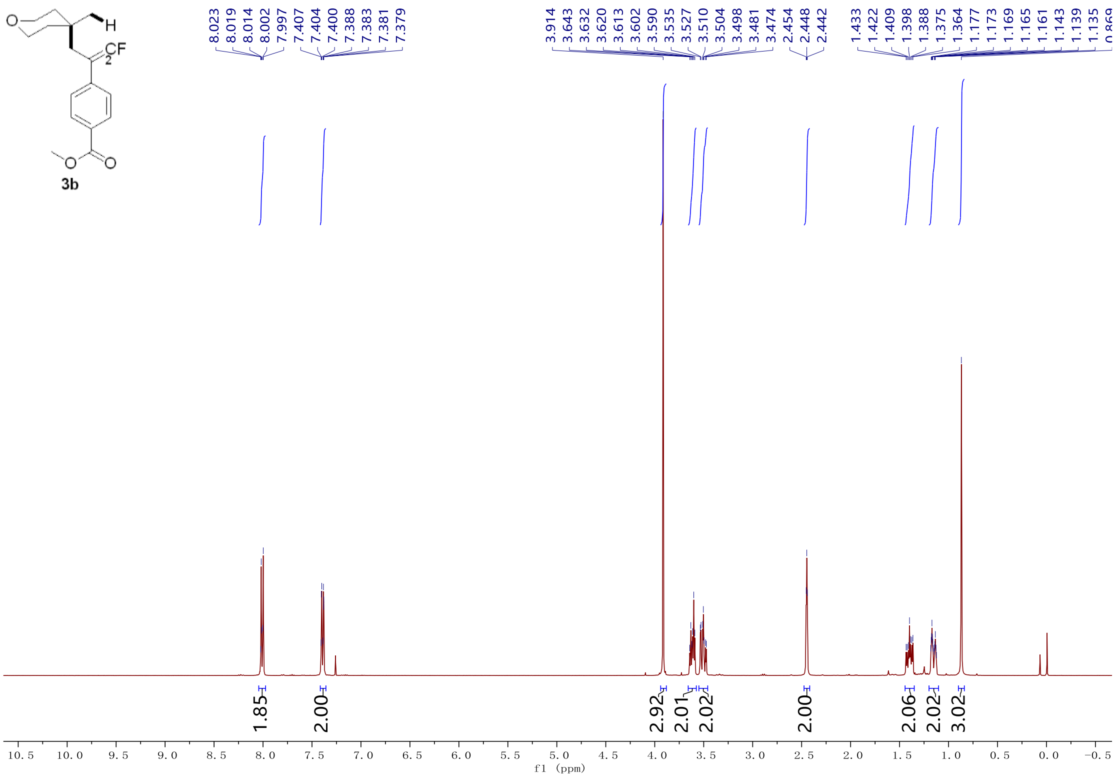

Supplementary Figure 95. ^{1}H NMR spectrum of compound **3b**

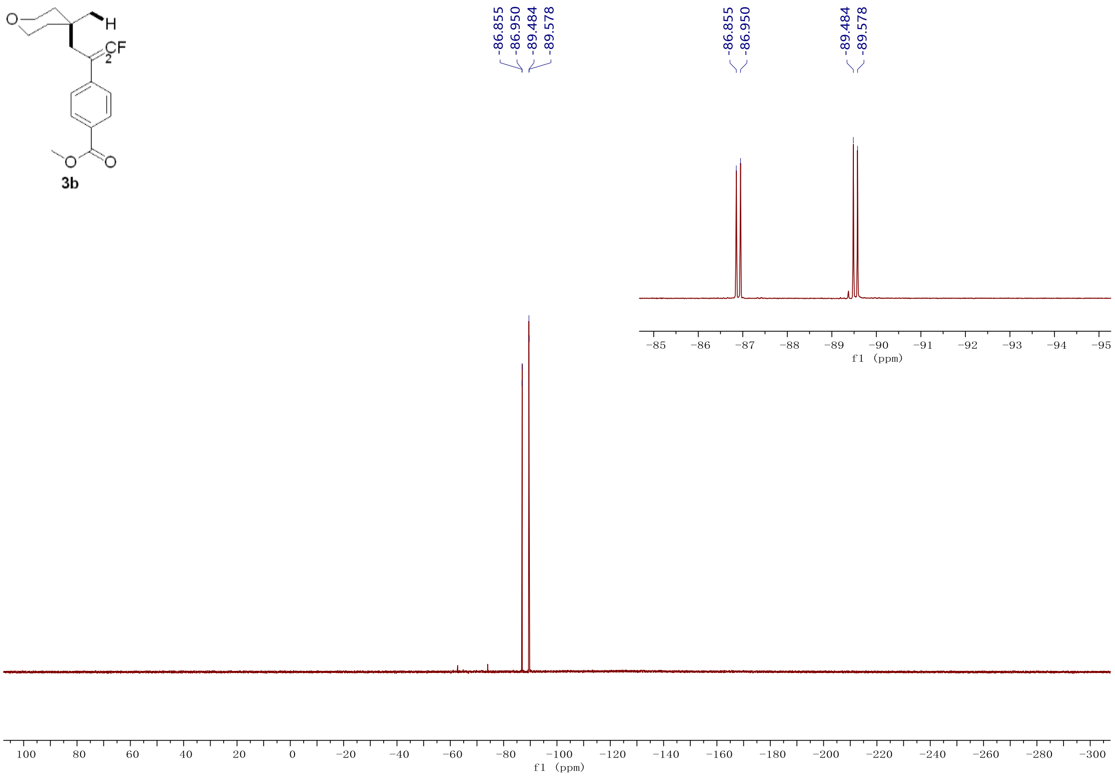

Supplementary Figure 96. ^{19}HF NMR spectrum of compound **3b**

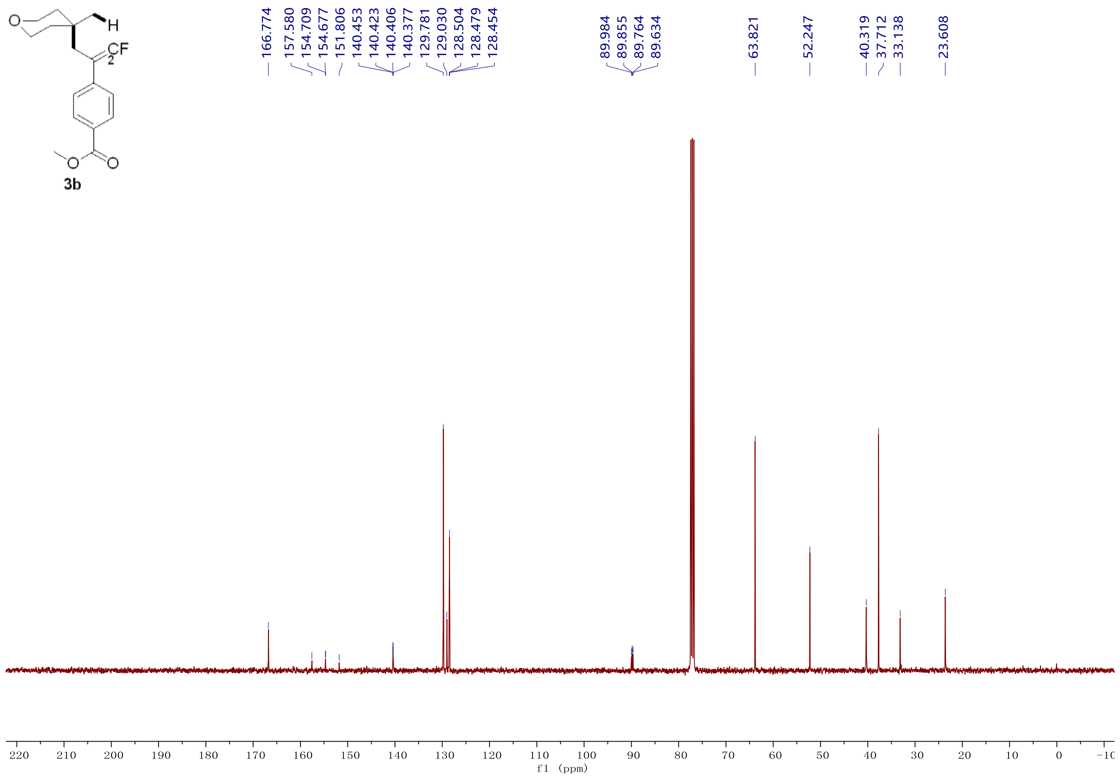

Supplementary Figure 97. ^{13}C NMR spectrum of compound **3b**

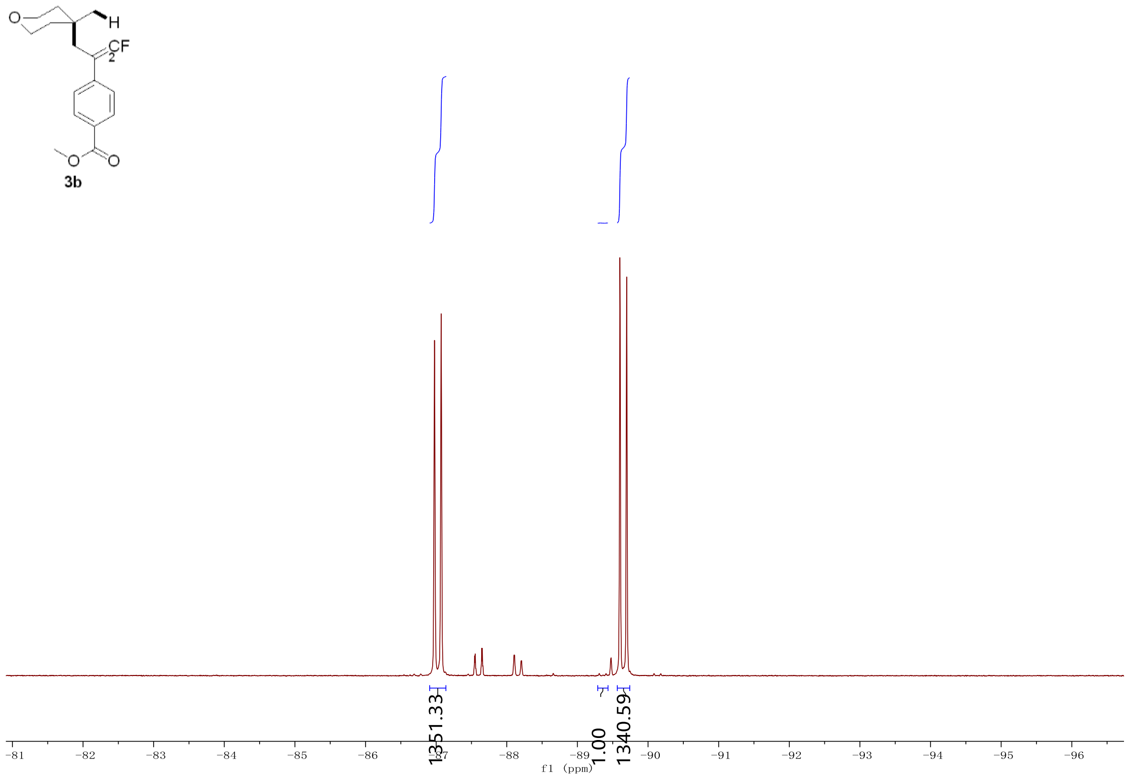

Supplementary Figure 98. Crude ^{19}F NMR spectrum of compound **3b** rr > 100 : 1

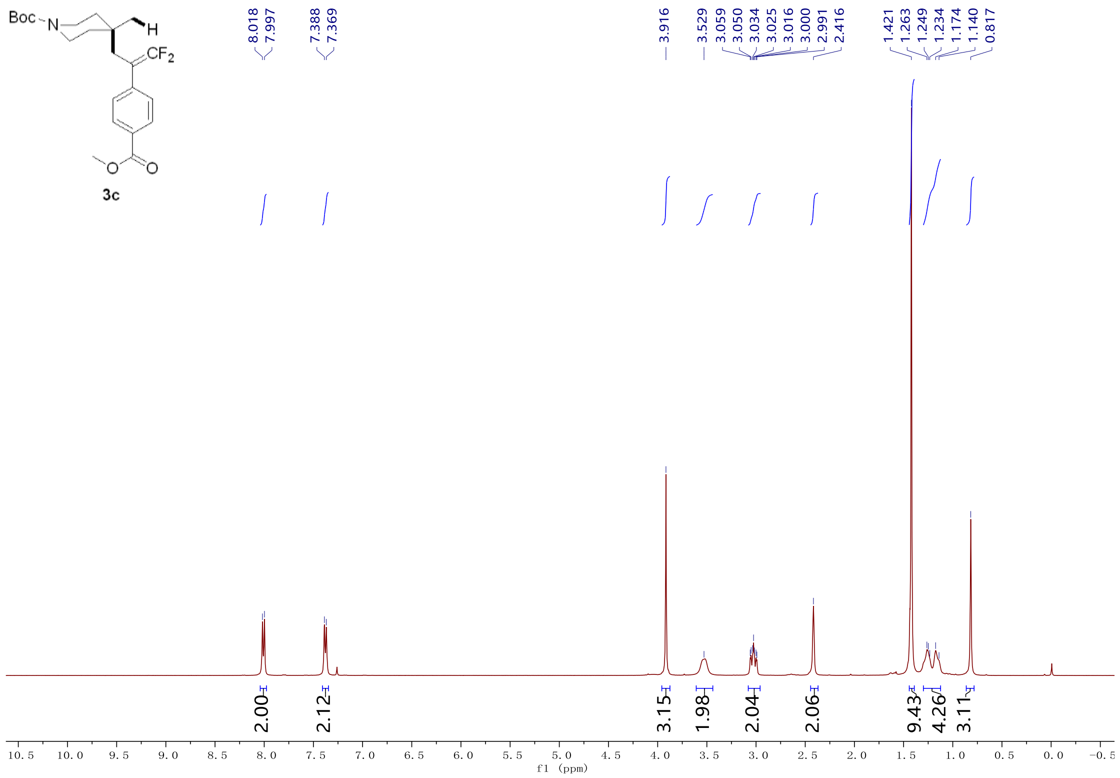

Supplementary Figure 99. ^{1}H NMR spectrum of compound **3c**

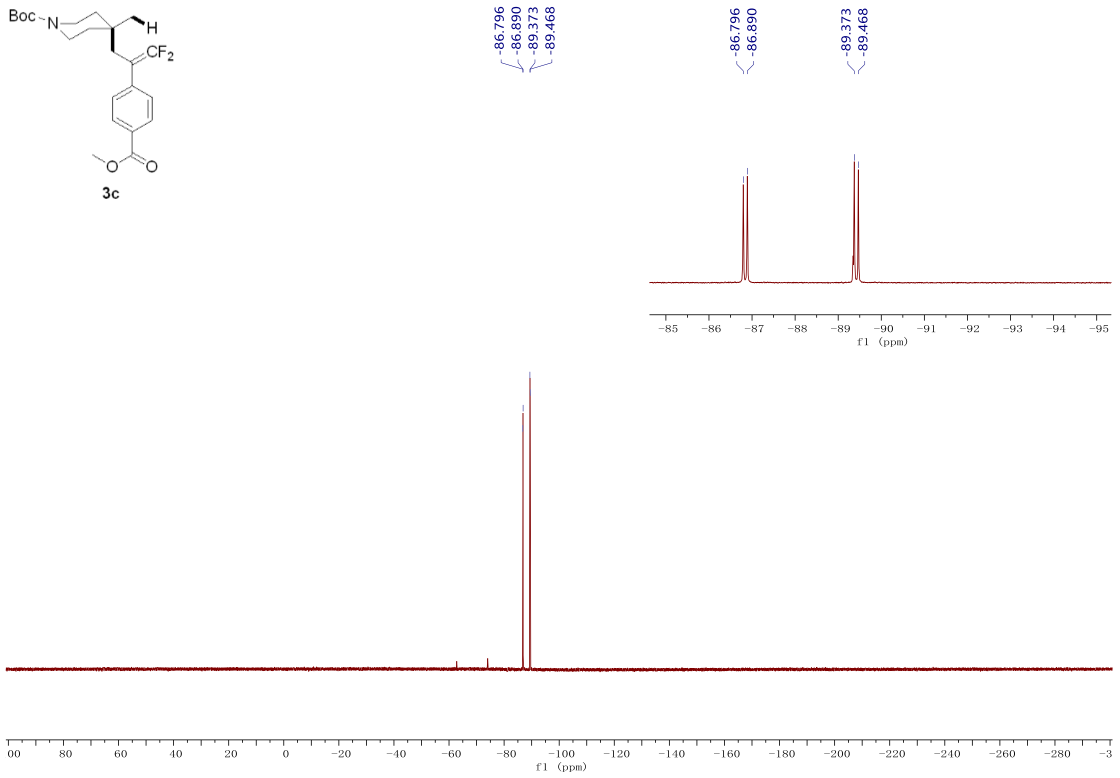

Supplementary Figure 100. ^{19}F NMR spectrum of compound **3c**

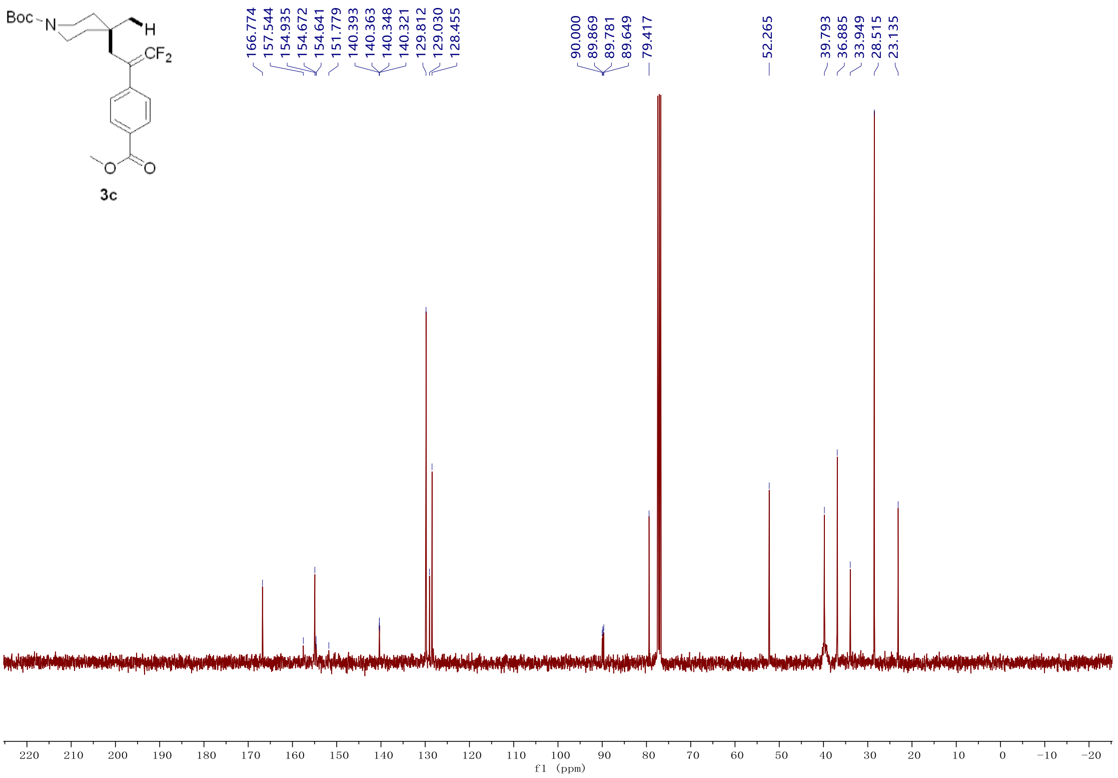

Supplementary Figure 101. ^{13}C NMR spectrum of compound **3c**

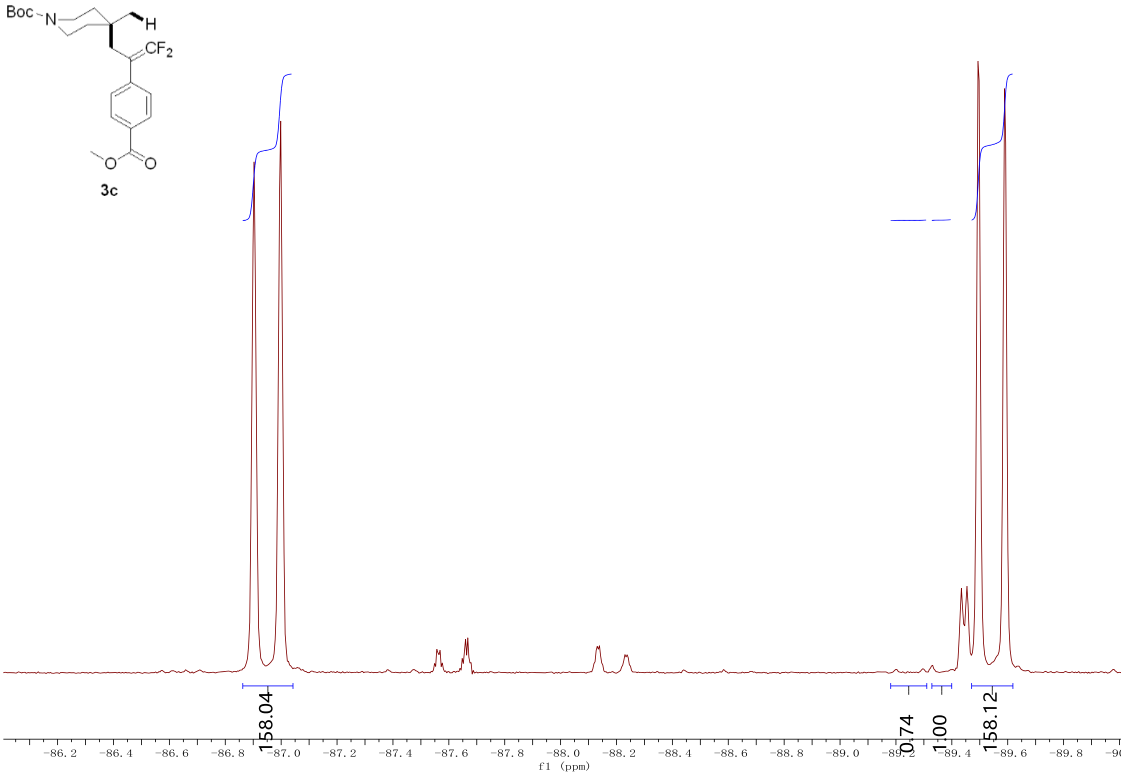

Supplementary Figure 102. Crude ^{19}F NMR spectrum of compound **3c** rr > 100 : 1

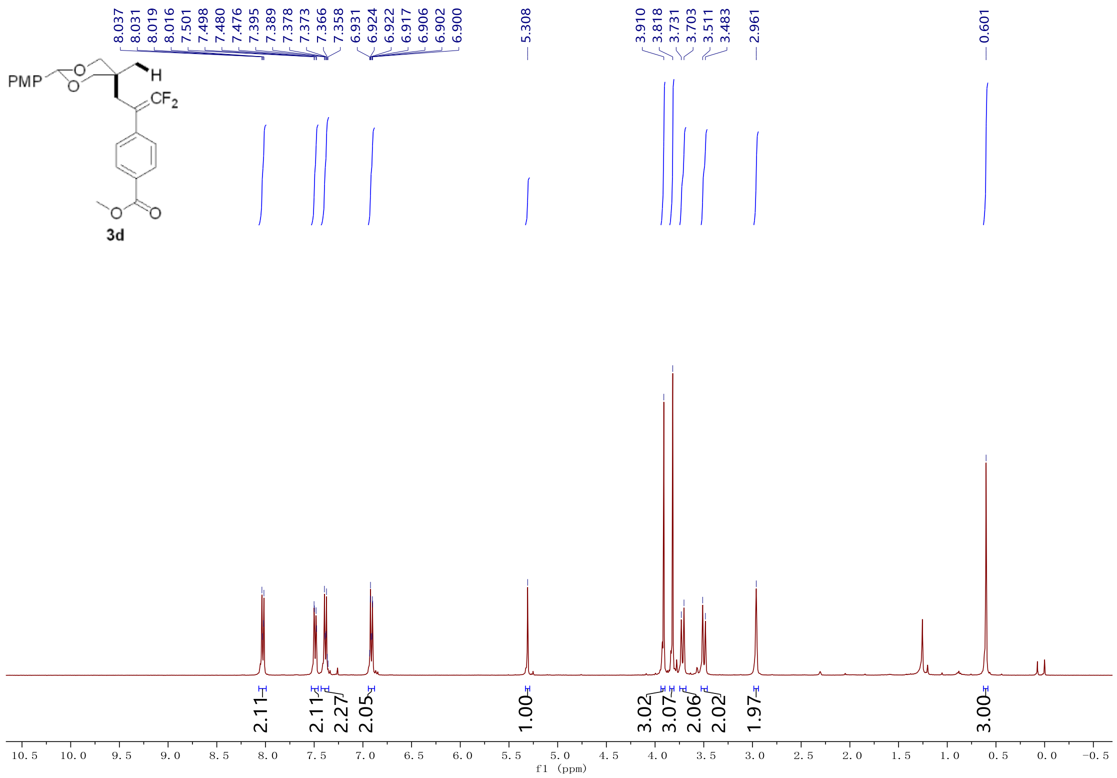

Supplementary Figure 103. ^{1}H NMR spectrum of compound **3d**

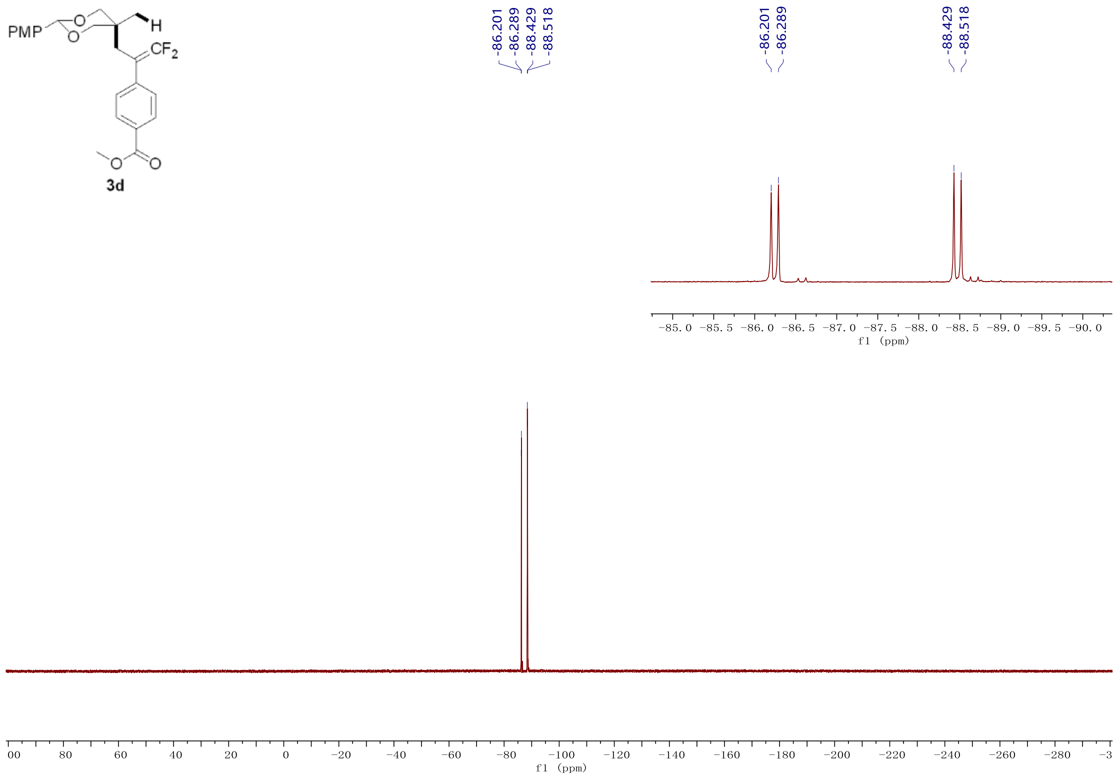

Supplementary Figure 104. ^{19}F NMR spectrum of compound **3d**

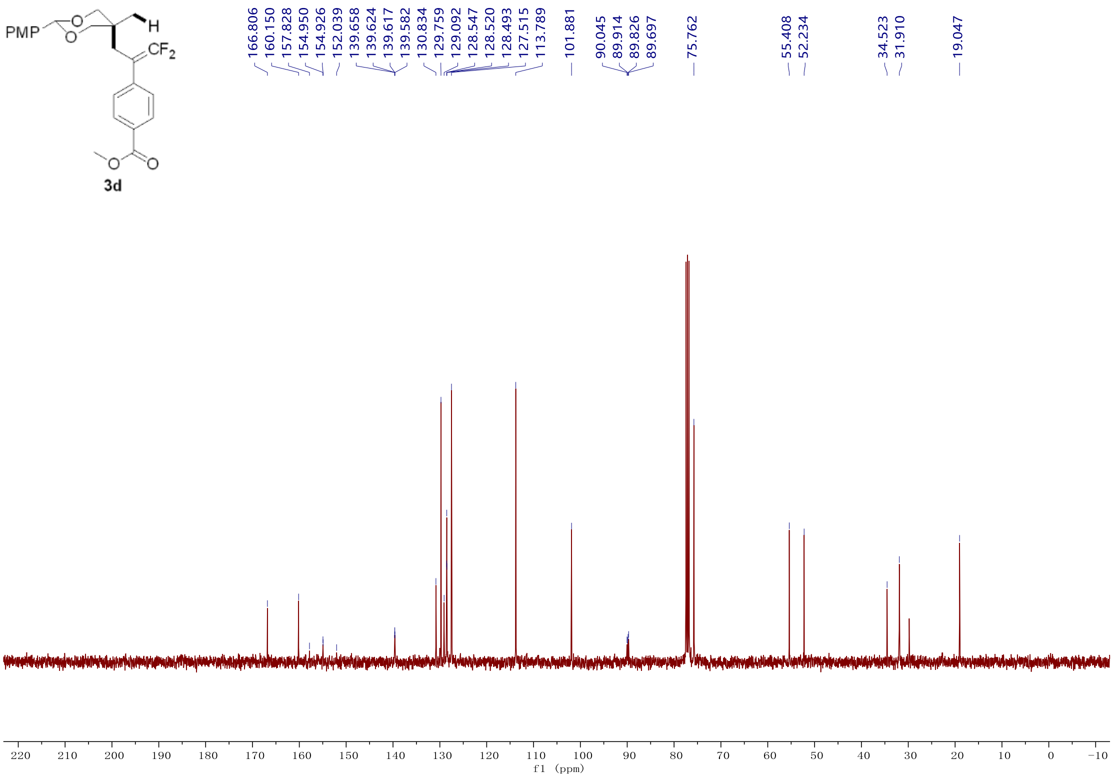

Supplementary Figure 105. ^{13}C NMR spectrum of compound **3d**

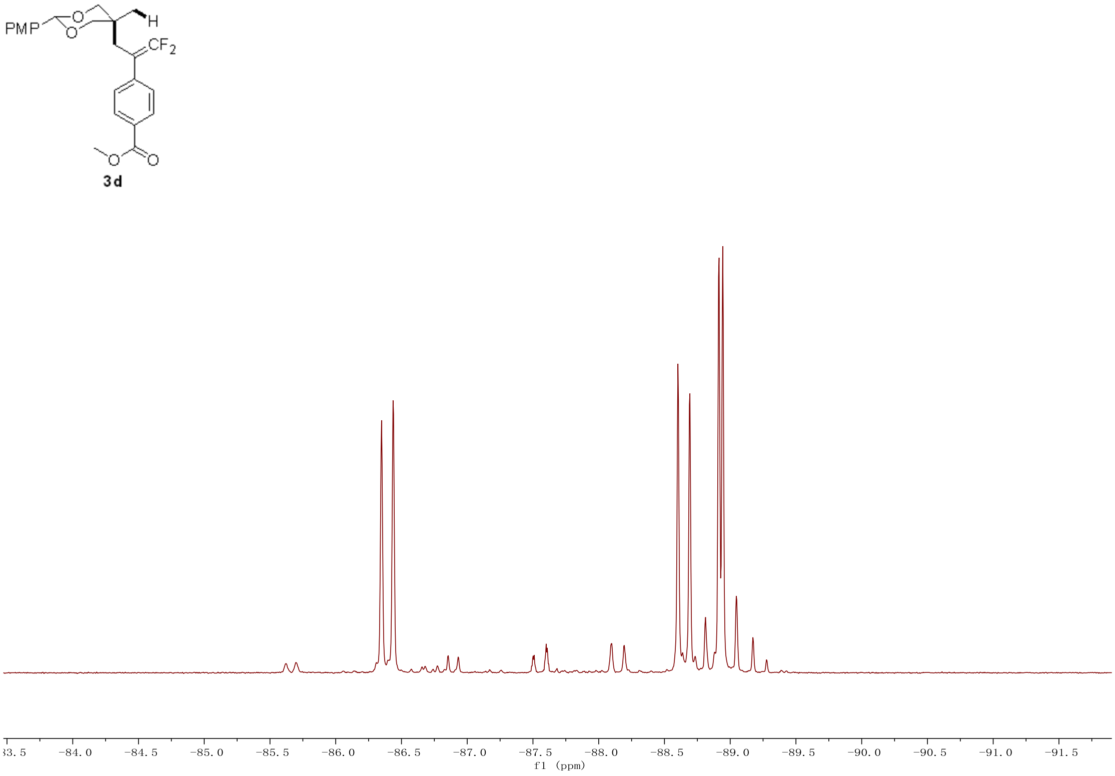

Supplementary Figure 106. Crude ^{19}F NMR spectrum of compound **3d** rr > 20 : 1

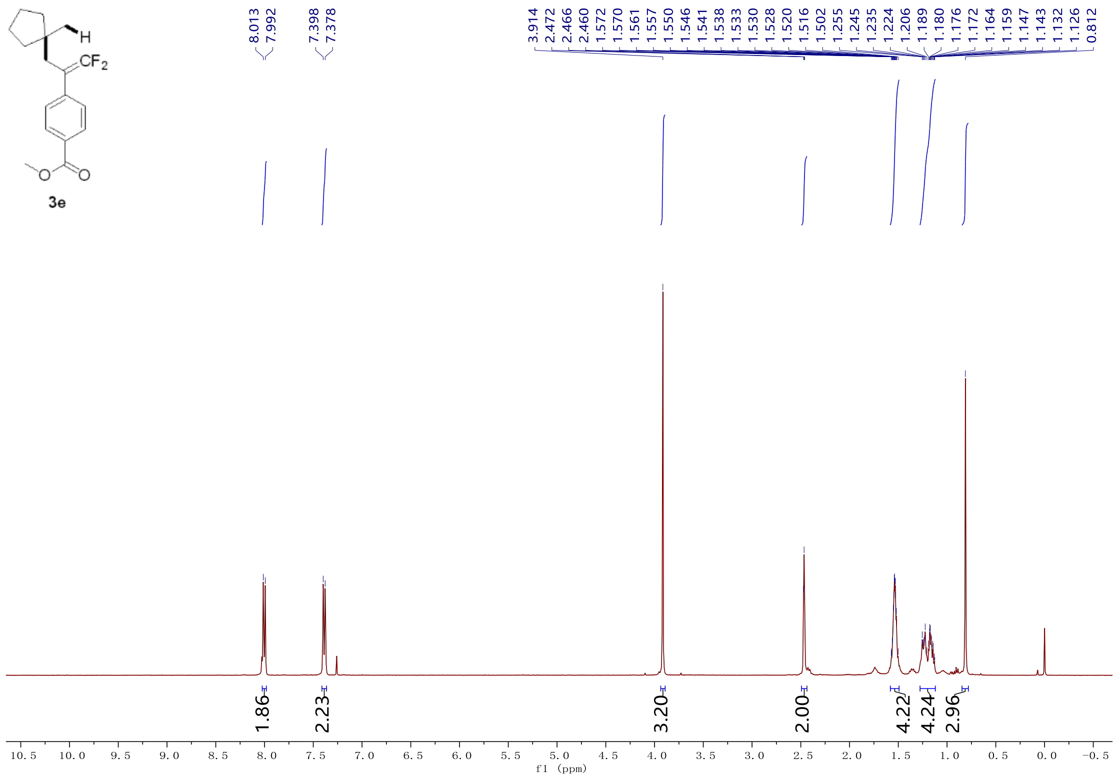

Supplementary Figure 107. ^{1}H NMR spectrum of compound **3e**

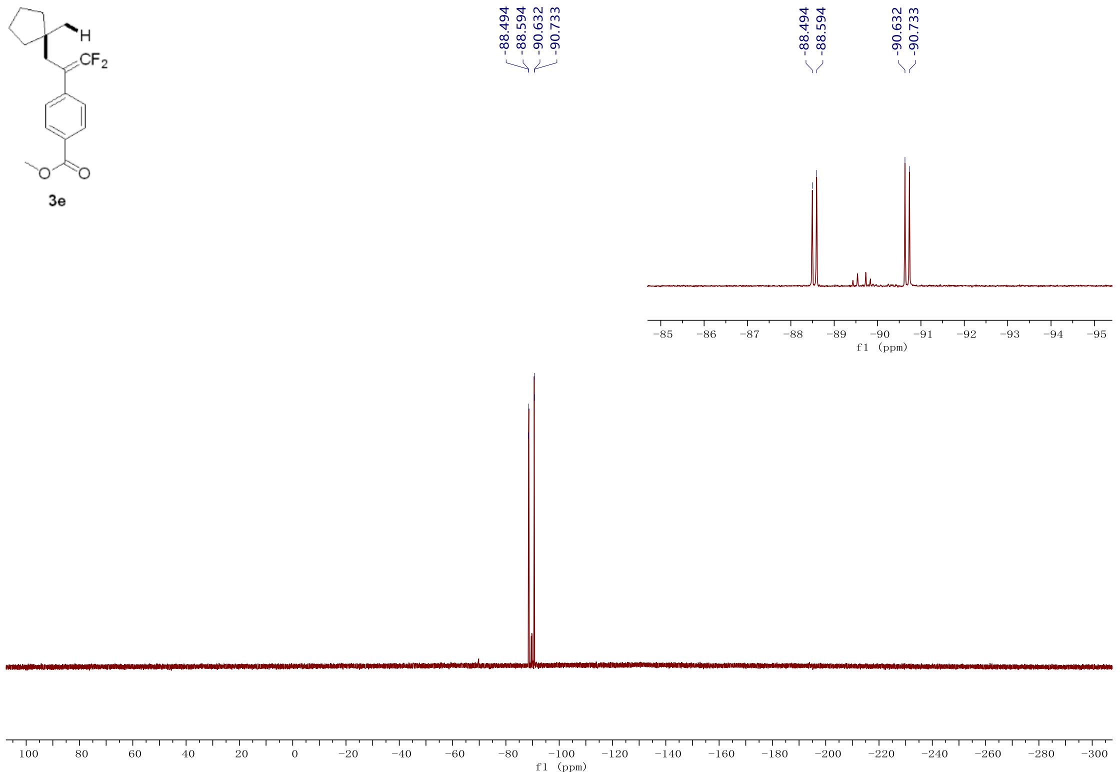

Supplementary Figure 108. ^{19}F NMR spectrum of compound **3e**

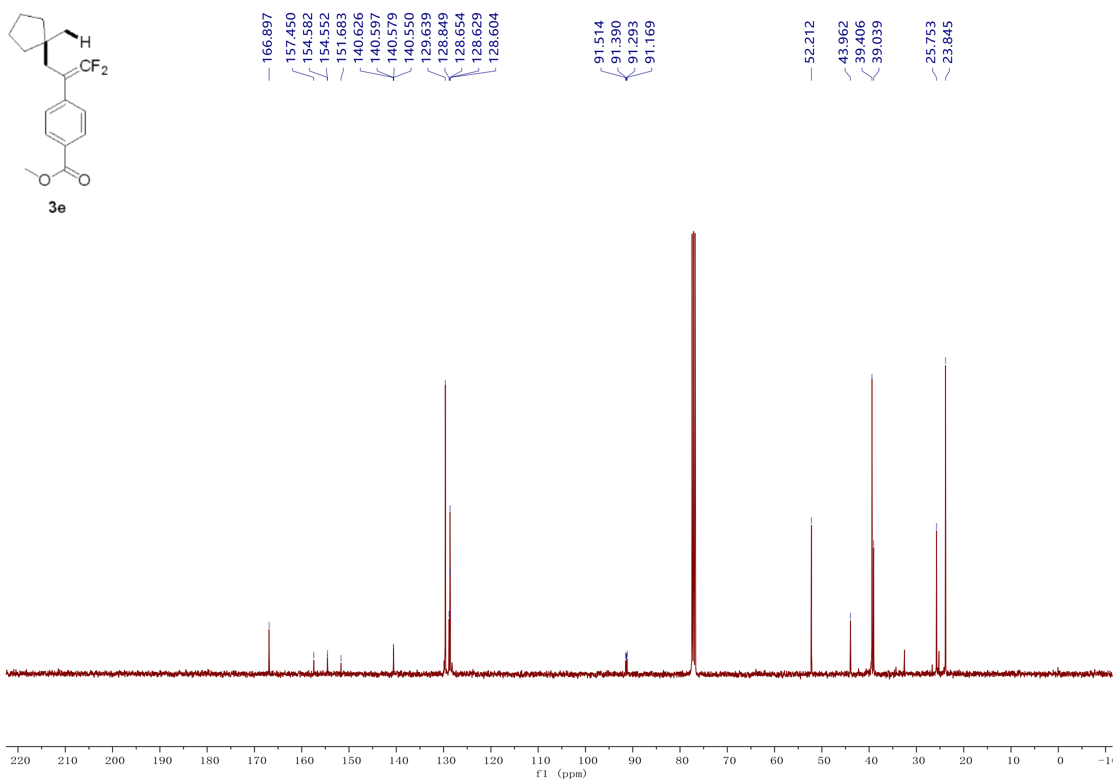

Supplementary Figure 109. ^{13}C NMR spectrum of compound **3e**

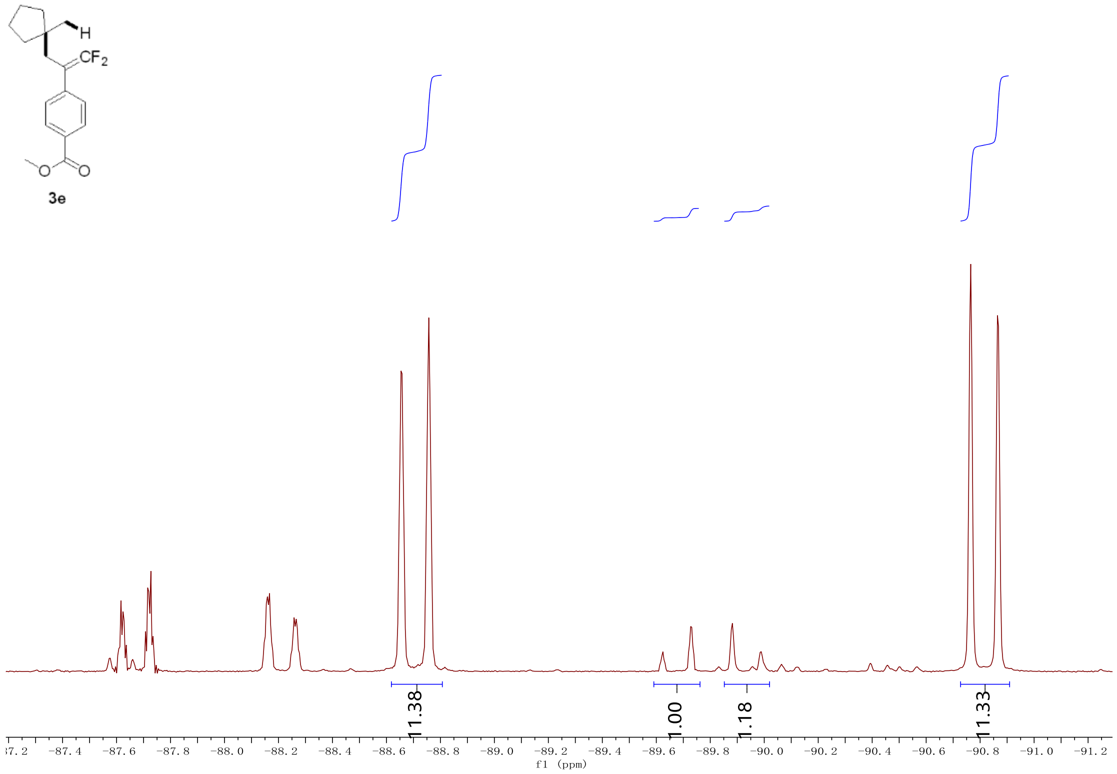

Supplementary Figure 110. Crude ^{19}F NMR spectrum of compound **3e** rr = 11 : 1

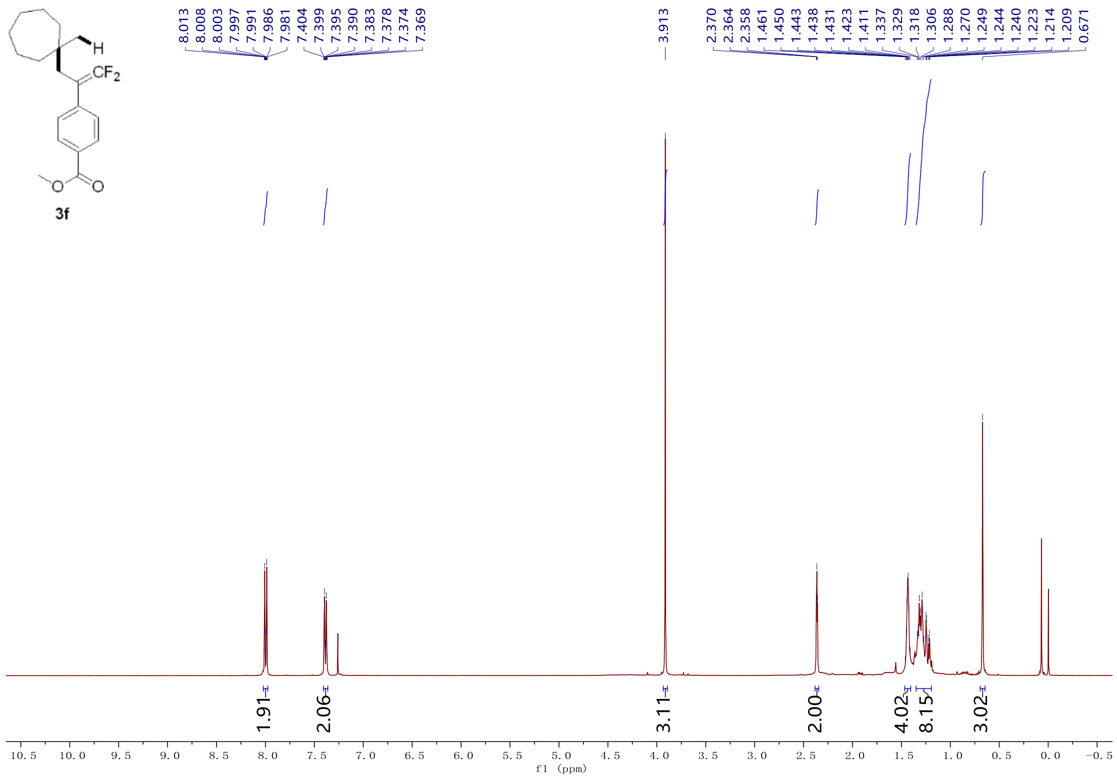

Supplementary Figure 111. ^{1}H NMR spectrum of compound **3f**

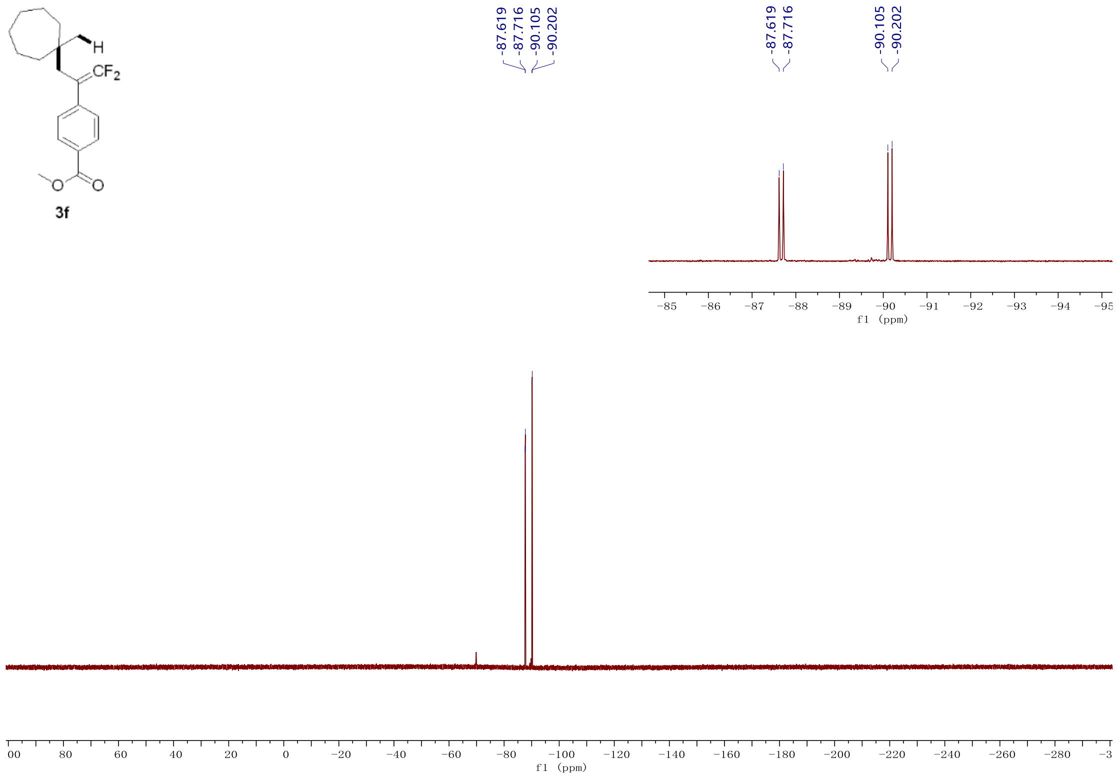

Supplementary Figure 112. ^{19}F NMR spectrum of compound **3f**

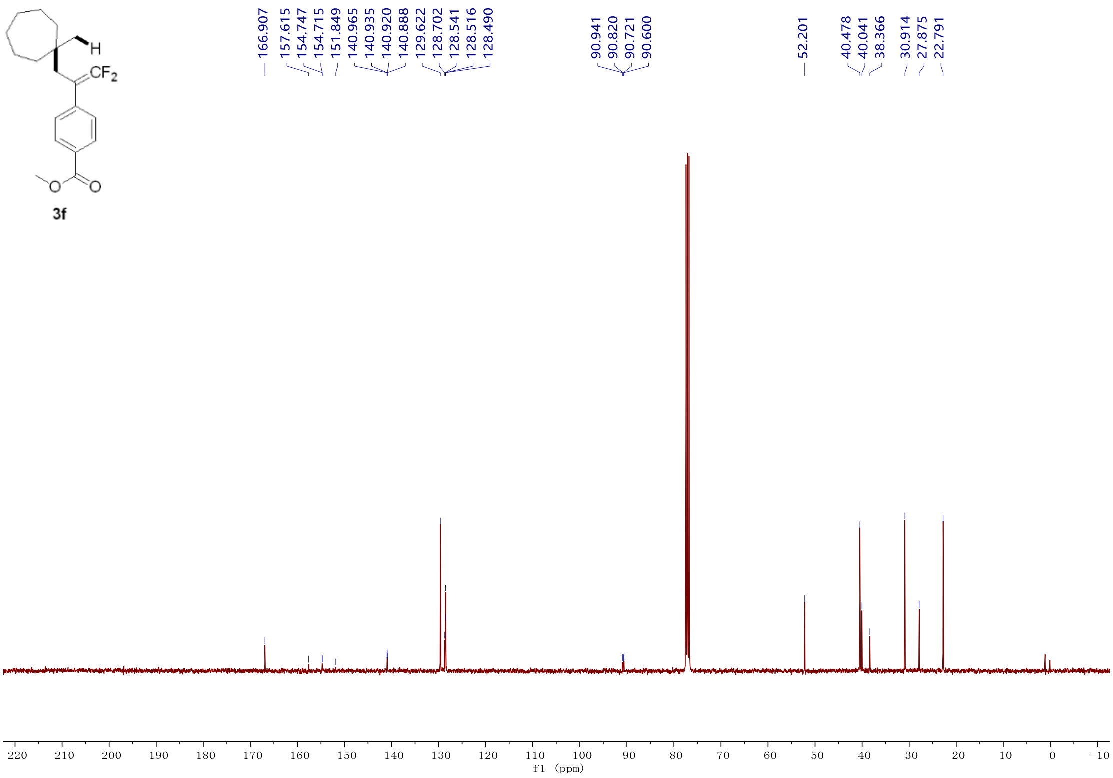

Supplementary Figure 113. ^{13}C NMR spectrum of compound **3f**

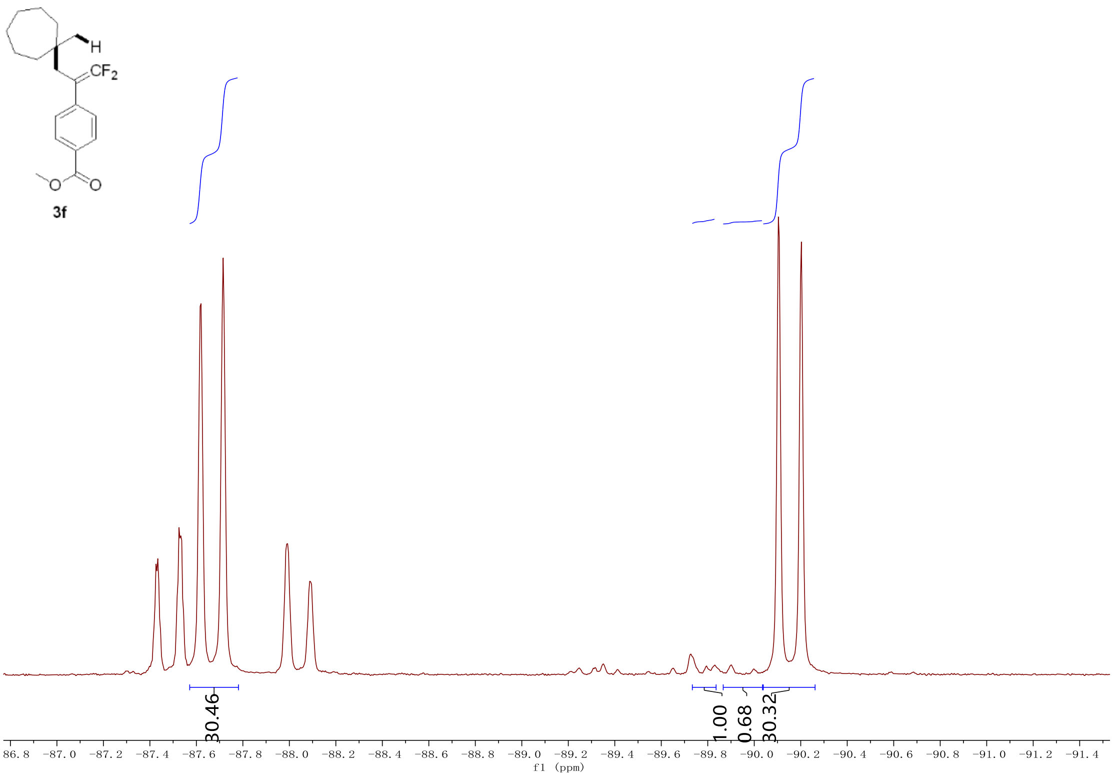

Supplementary Figure 114. Crude ^{19}F NMR spectrum of compound **3f** rr = 30 : 1

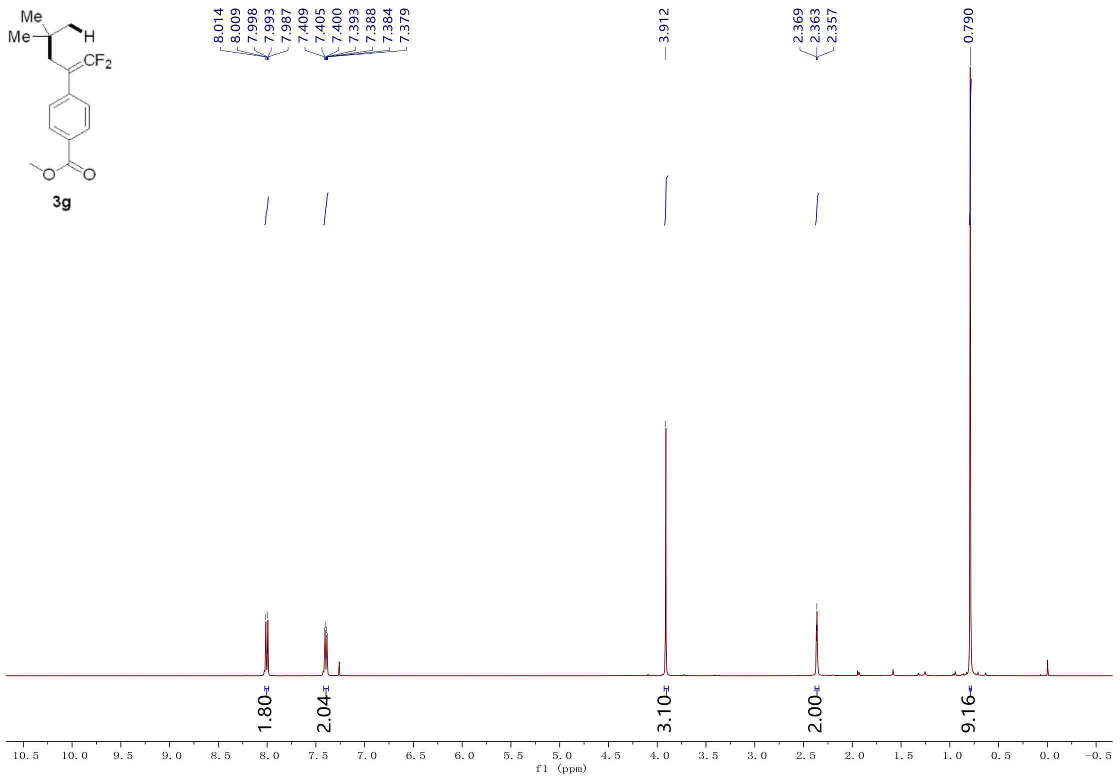

Supplementary Figure 115. ^{1}H NMR spectrum of compound **3g**

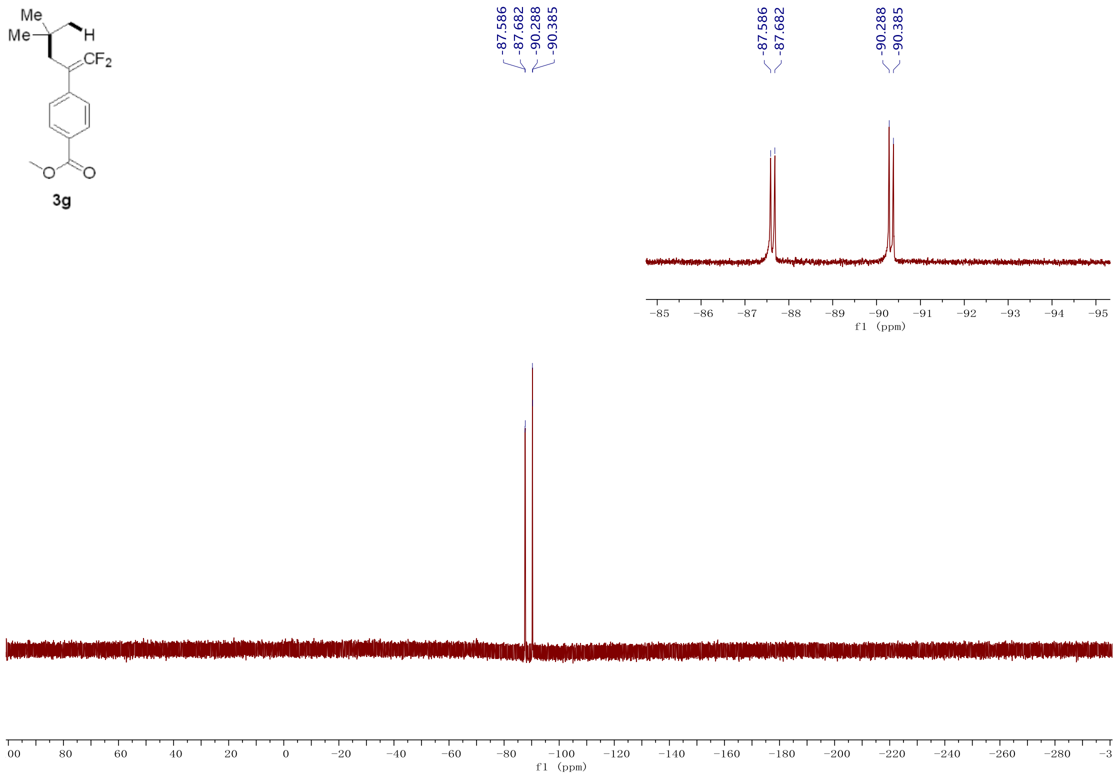

Supplementary Figure 116. ^{19}F NMR spectrum of compound **3g**

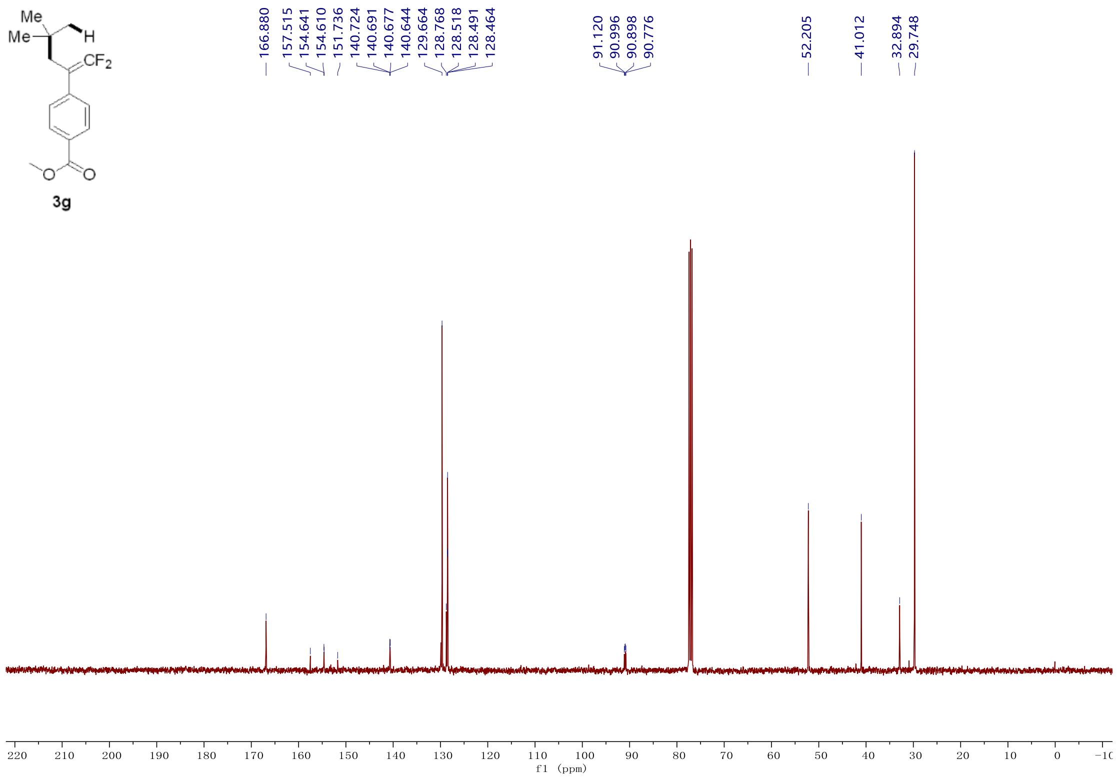

Supplementary Figure 117. ^{13}C NMR spectrum of compound **3g**

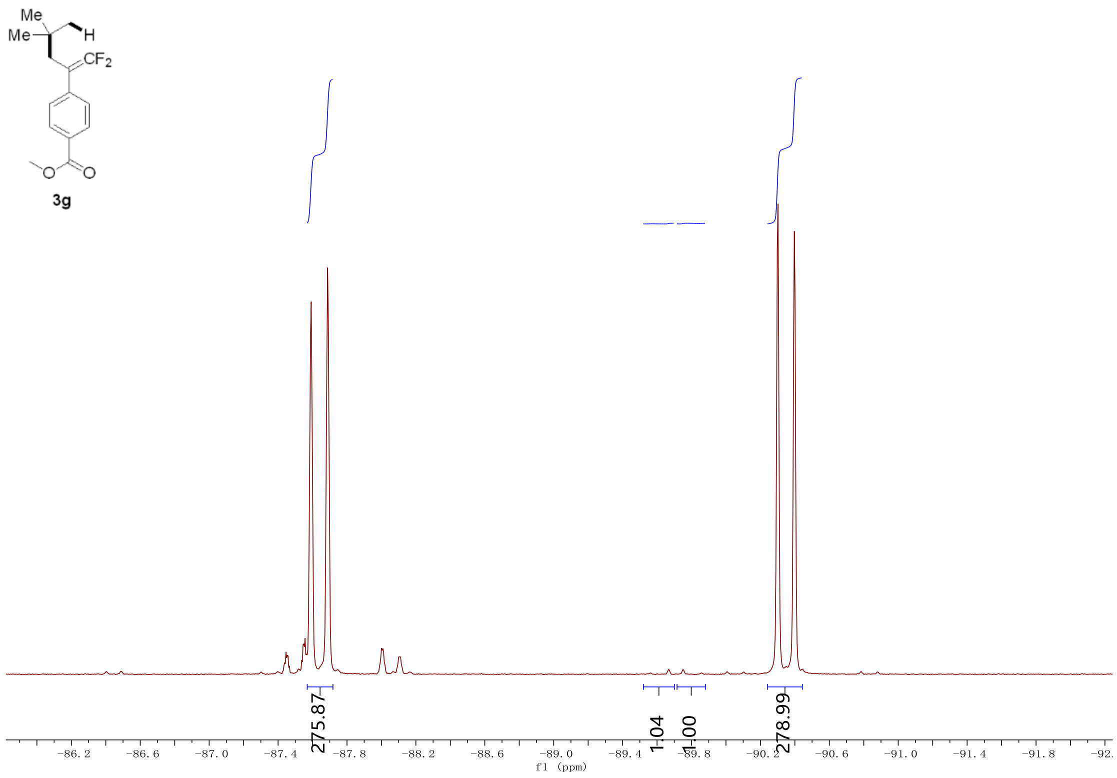

Supplementary Figure 118. Crude ^{19}F NMR spectrum of compound **3g** rr > 100 : 1

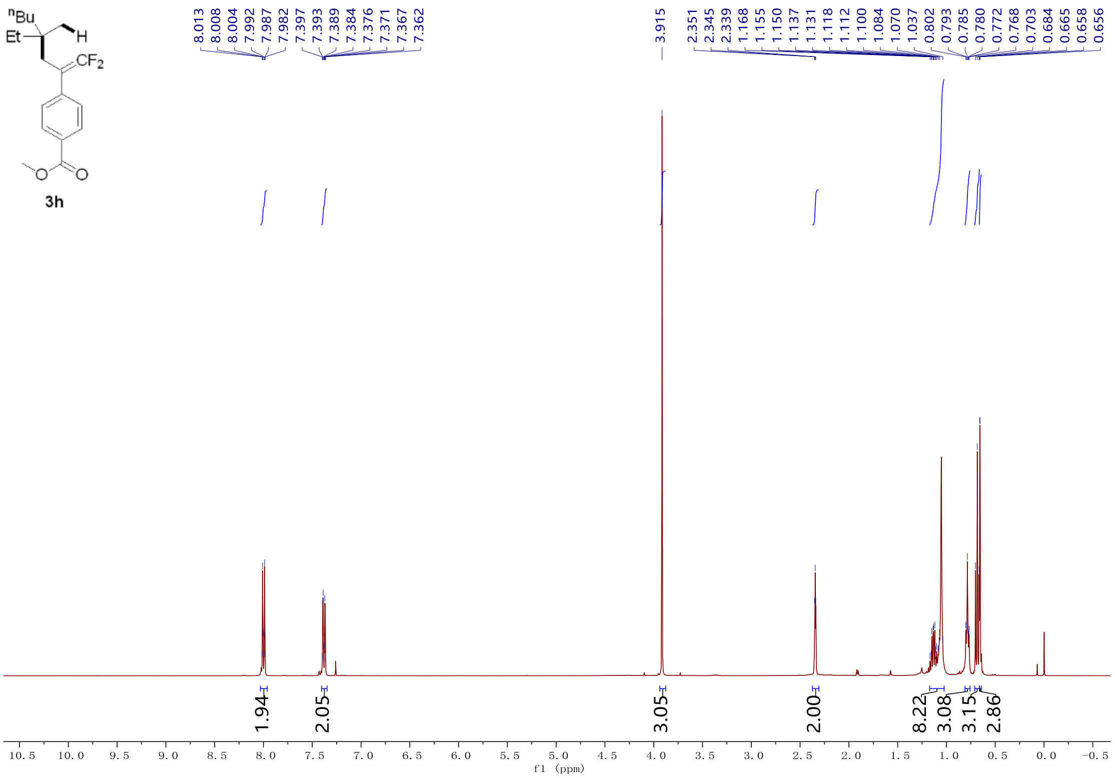

Supplementary Figure 119. ^{1}H NMR spectrum of compound **3h**

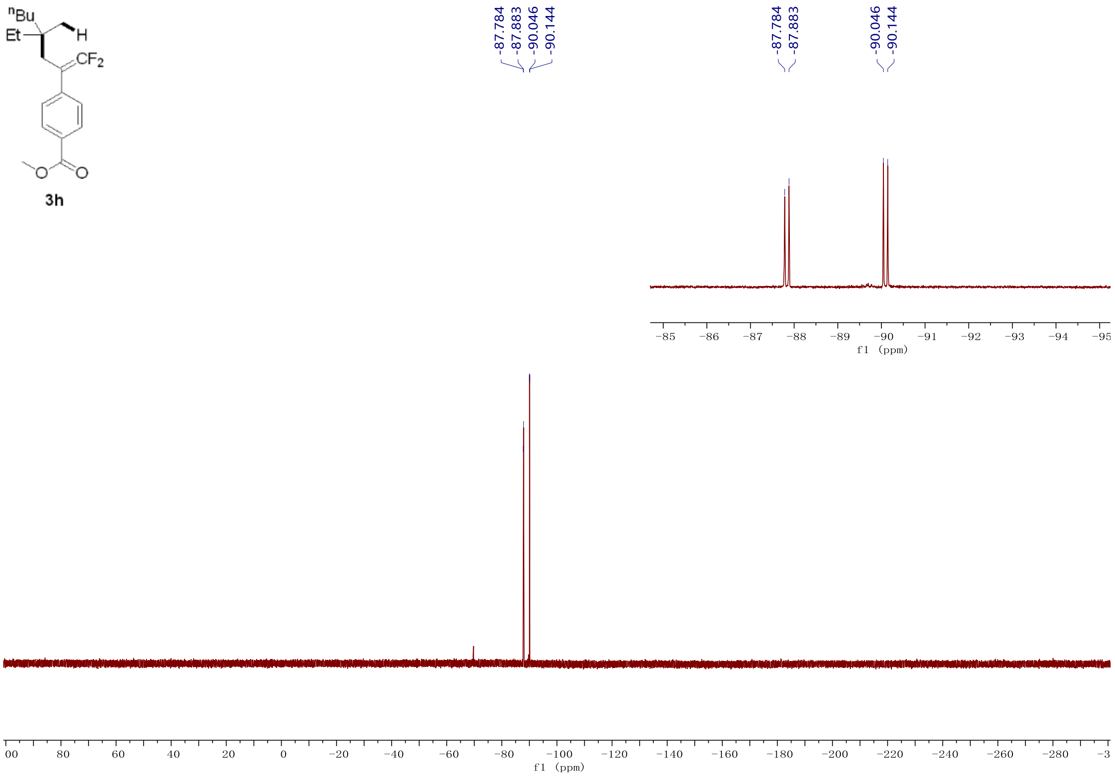

Supplementary Figure 120. ^{19}F NMR spectrum of compound **3h**

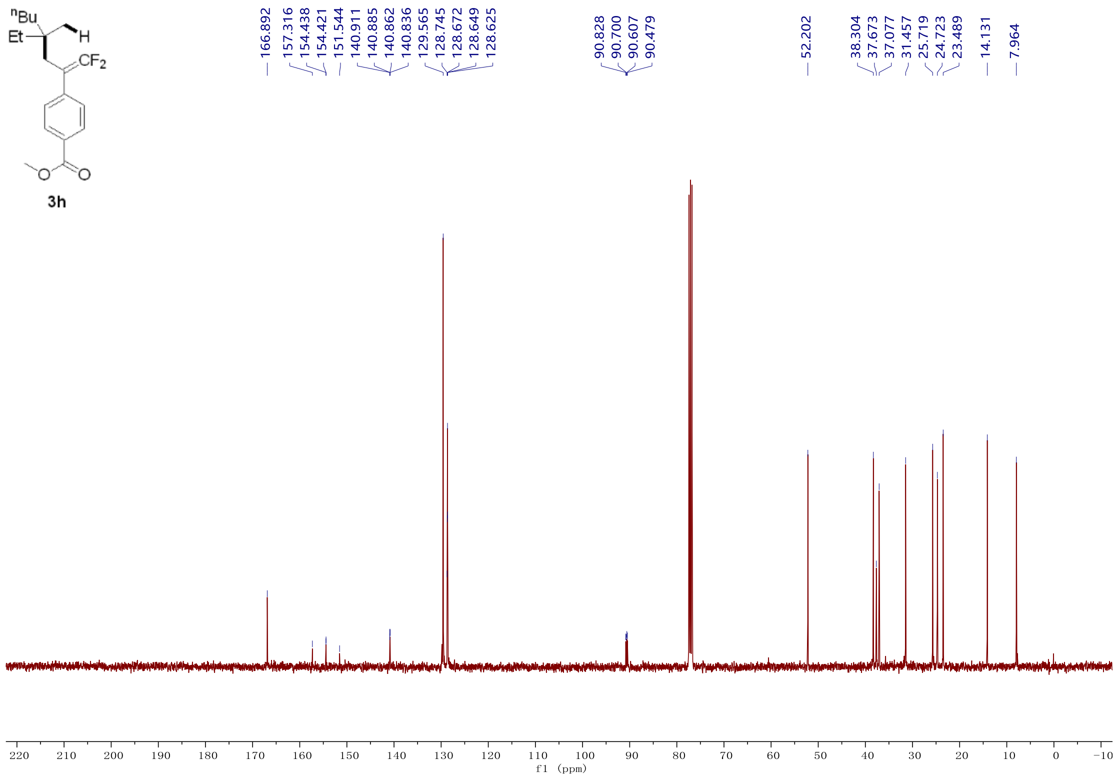

Supplementary Figure 121. ^{13}C NMR spectrum of compound **3h**

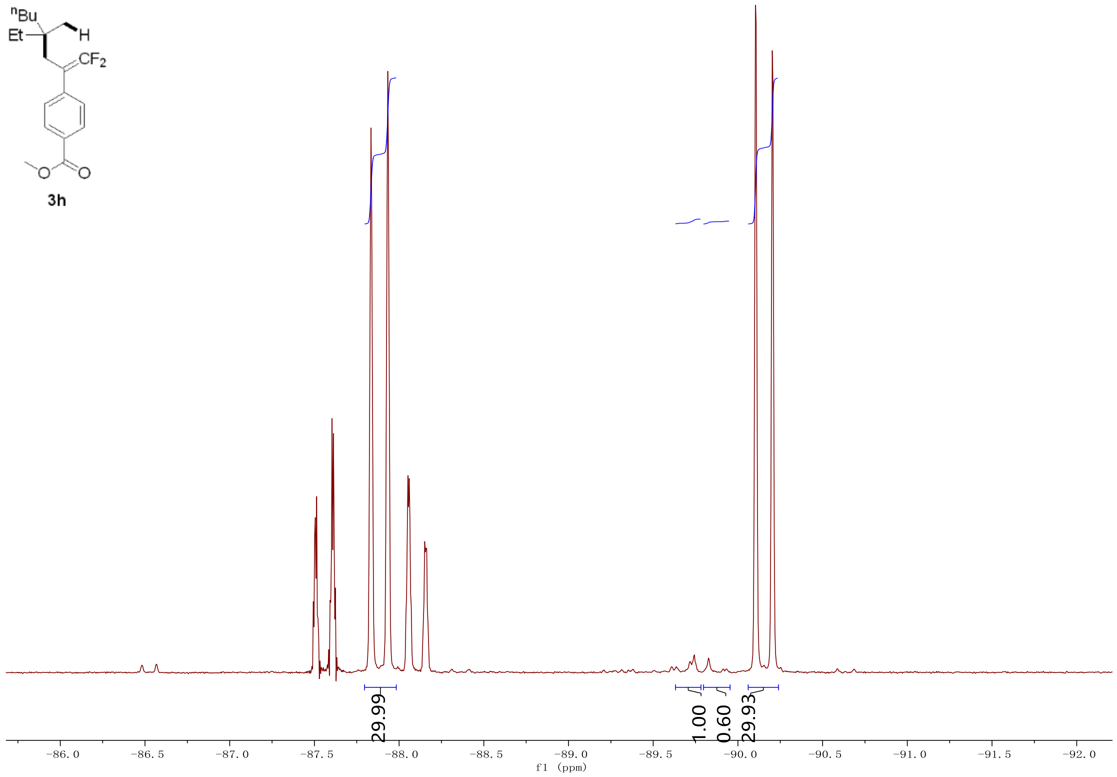

Supplementary Figure 122. Crude ^{19}F NMR spectrum of compound **3h** rr = 30 : 1

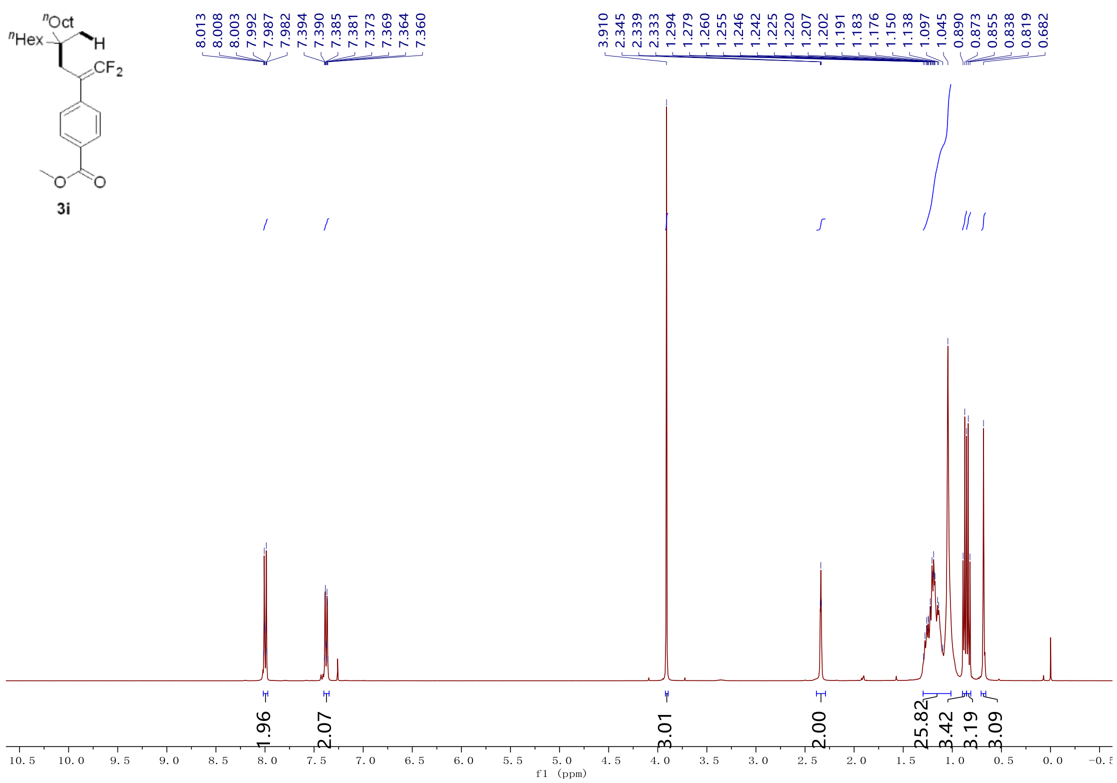

Supplementary Figure 123. ^{1}H NMR spectrum of compound **3i**

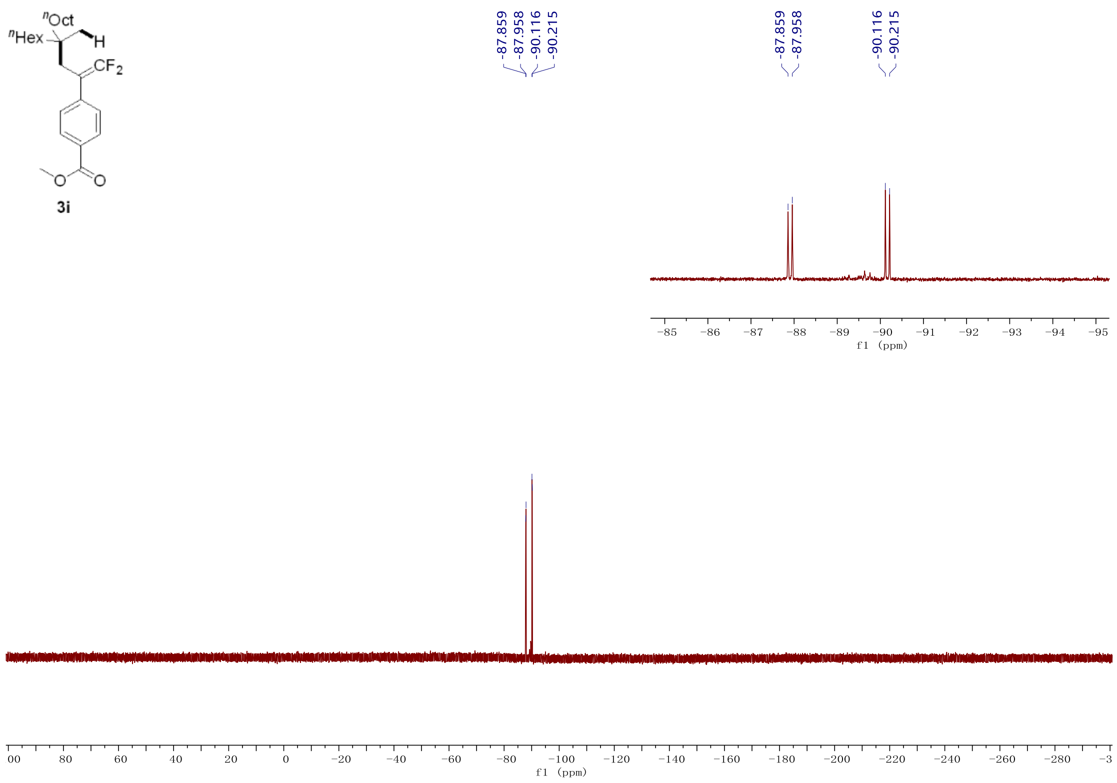

Supplementary Figure 124. ^{19}F NMR spectrum of compound **3i**

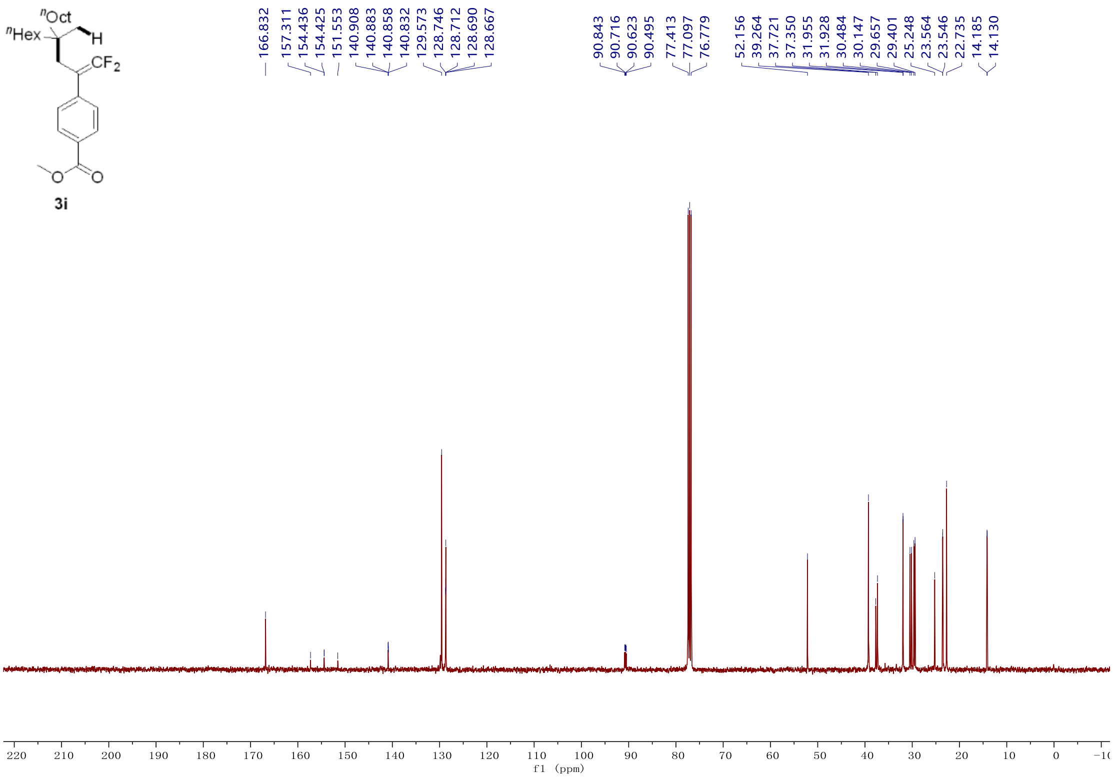

Supplementary Figure 125. ^{13}C NMR spectrum of compound **3i**

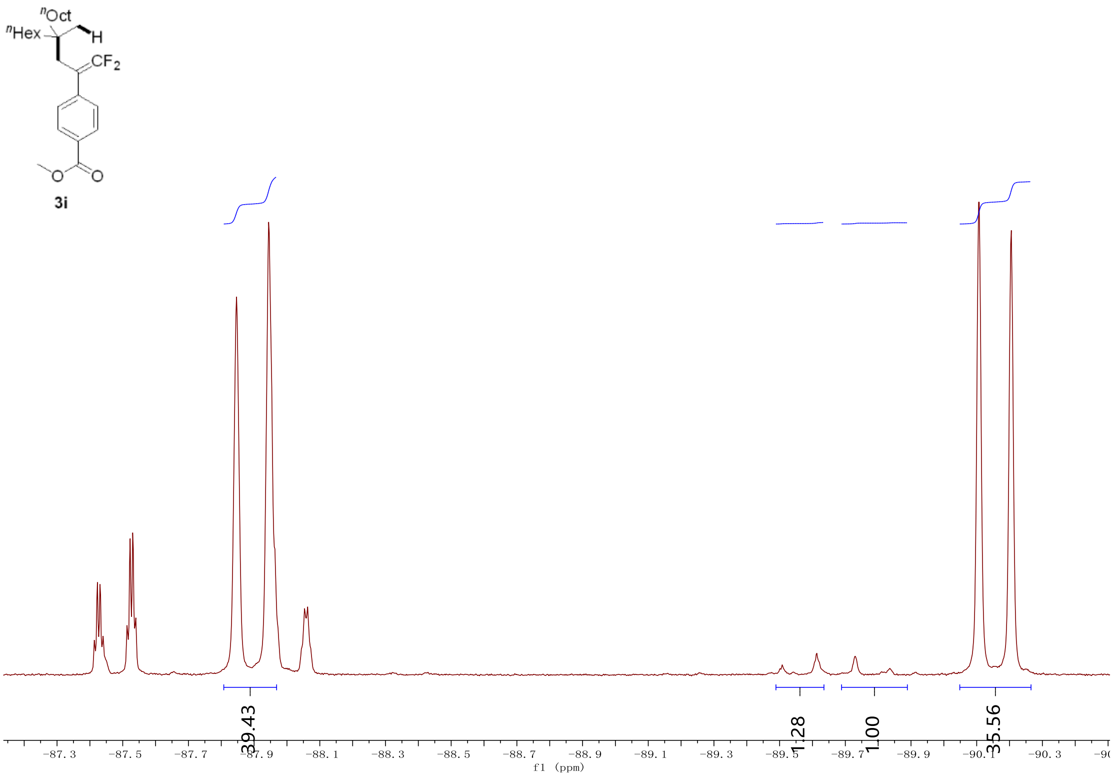

Supplementary Figure 126. Crude ^{19}F NMR spectrum of compound **3i** rr = 35 : 1

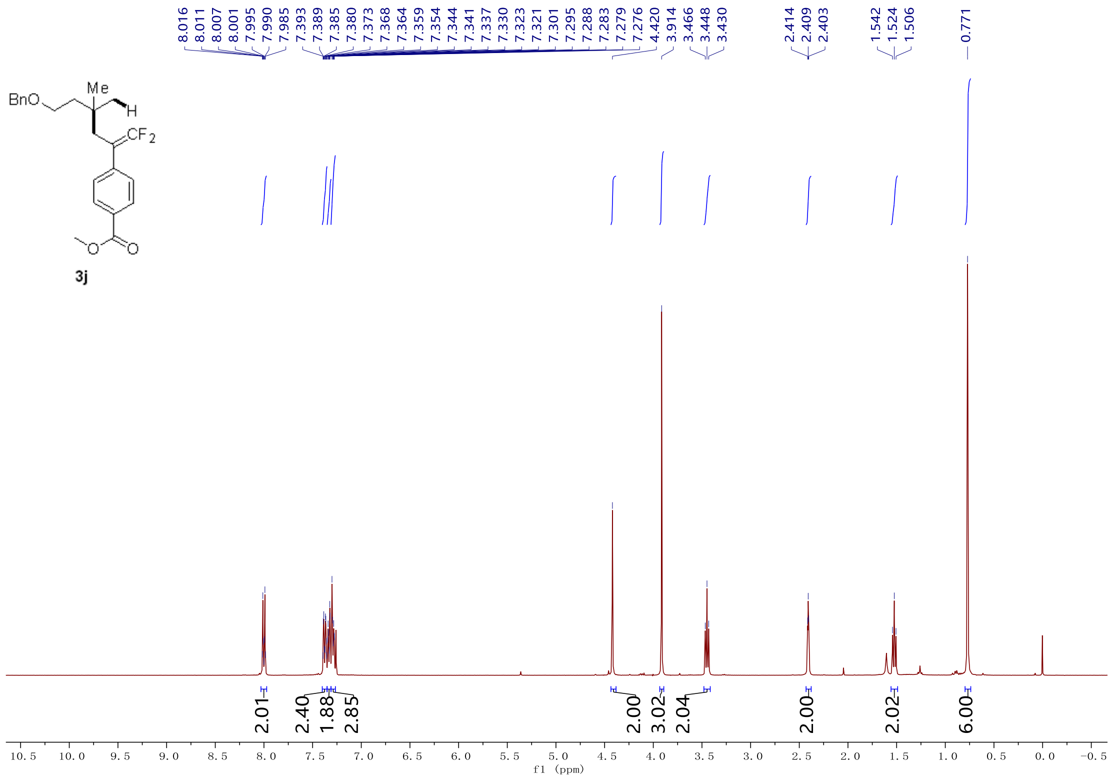

Supplementary Figure 127. ^{1}H NMR spectrum of compound **3j**

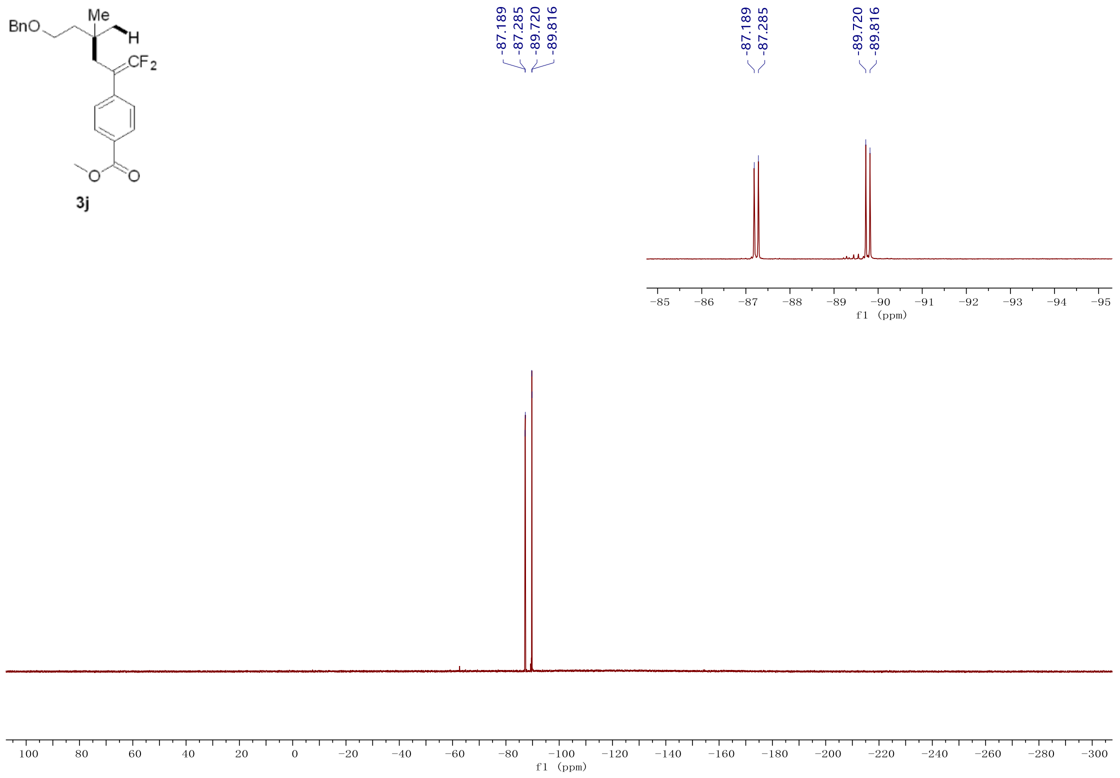

Supplementary Figure 128. ^{19}F NMR spectrum of compound **3j**

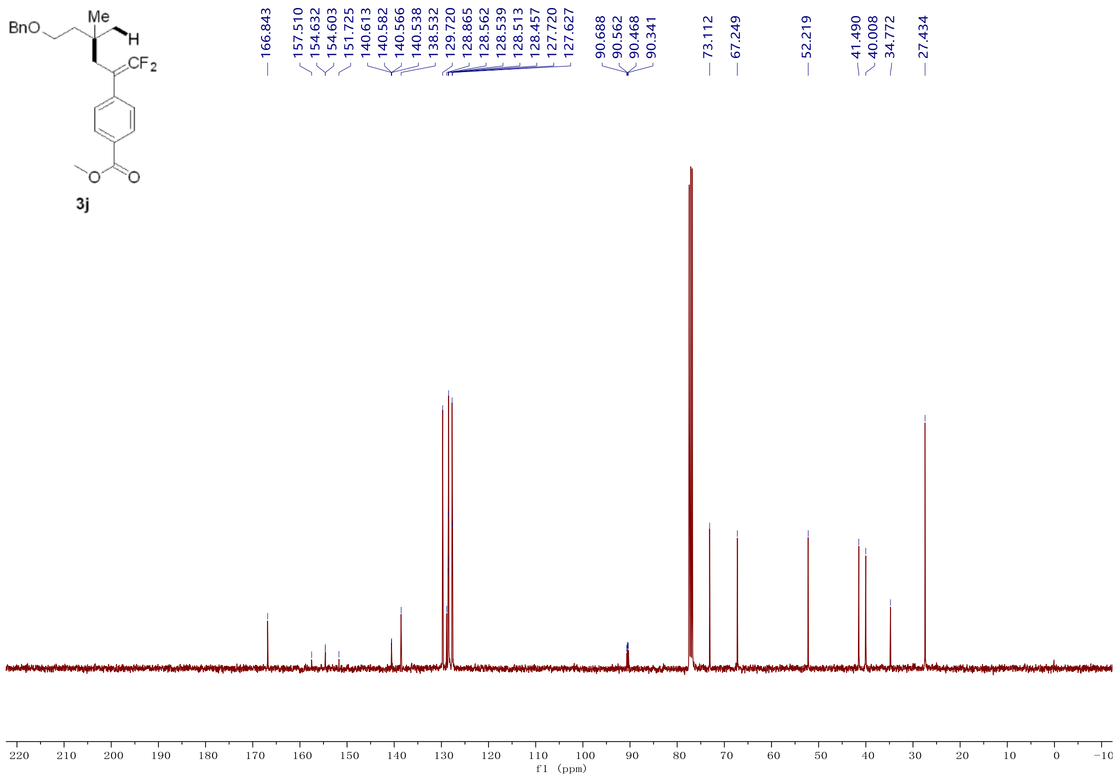

Supplementary Figure 129. ^{13}C NMR spectrum of compound **3j**

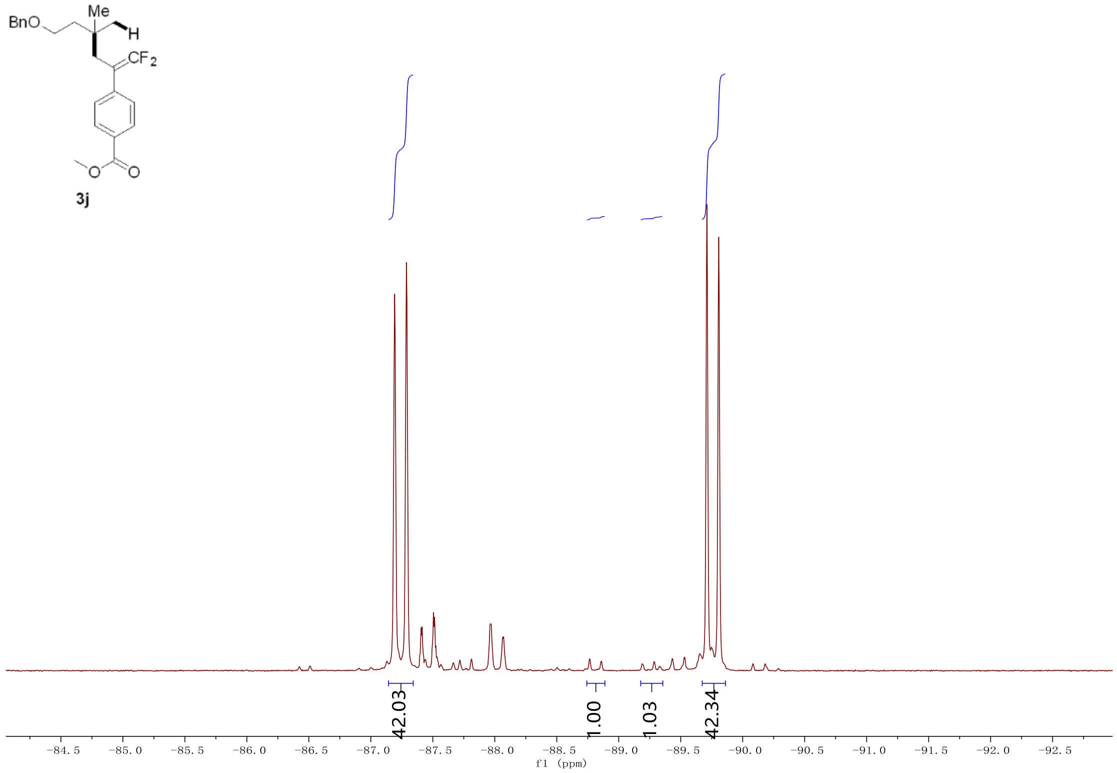

Supplementary Figure 130. Crude ^{19}F NMR spectrum of compound **3j** rr = 42 : 1

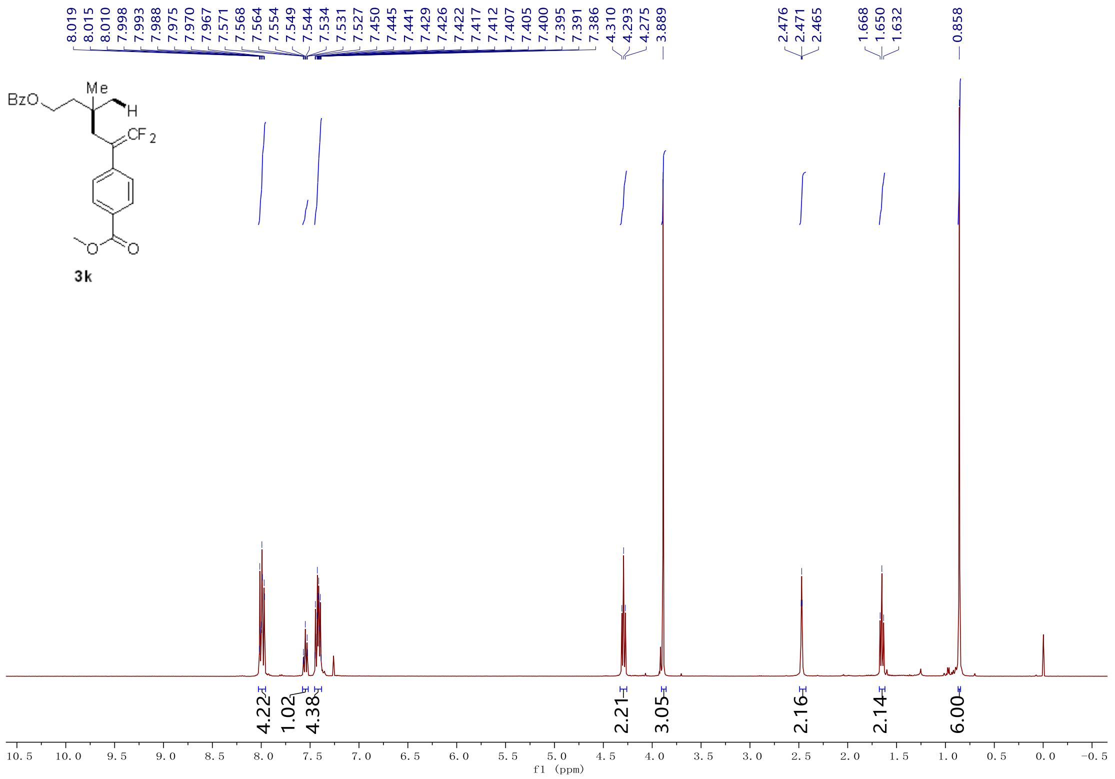

Supplementary Figure 131. ^{1}H NMR spectrum of compound **3k**

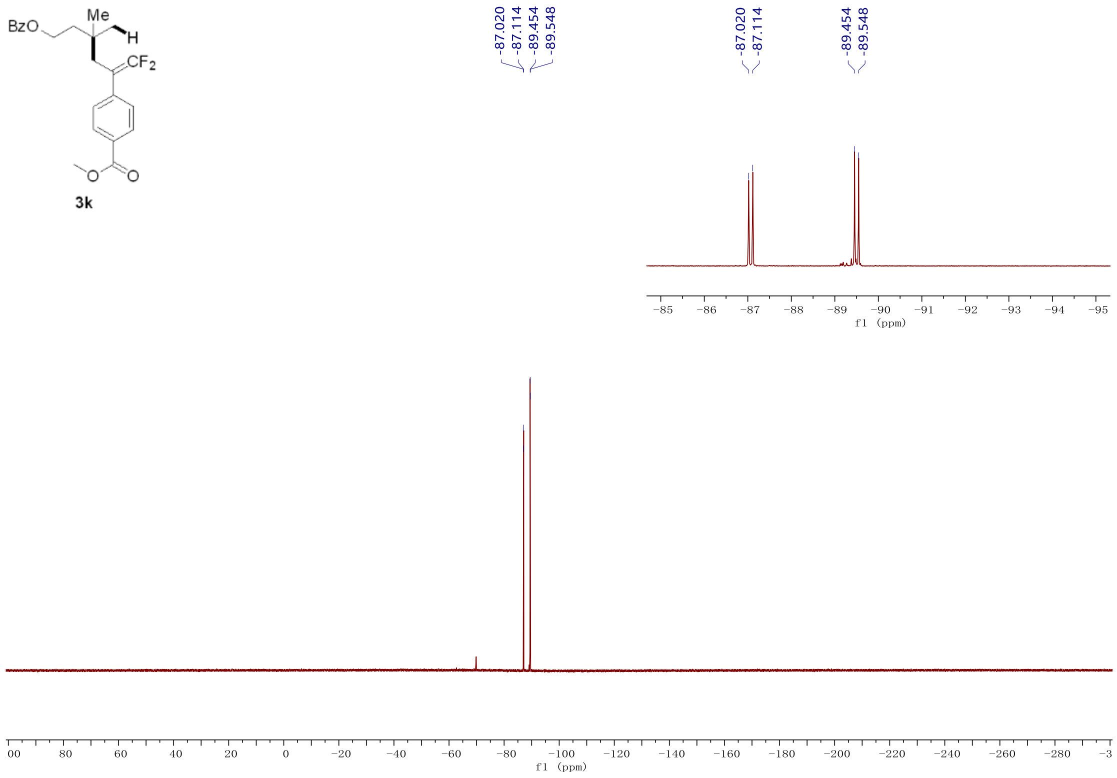

Supplementary Figure 132. ^{19}F NMR spectrum of compound **3k**

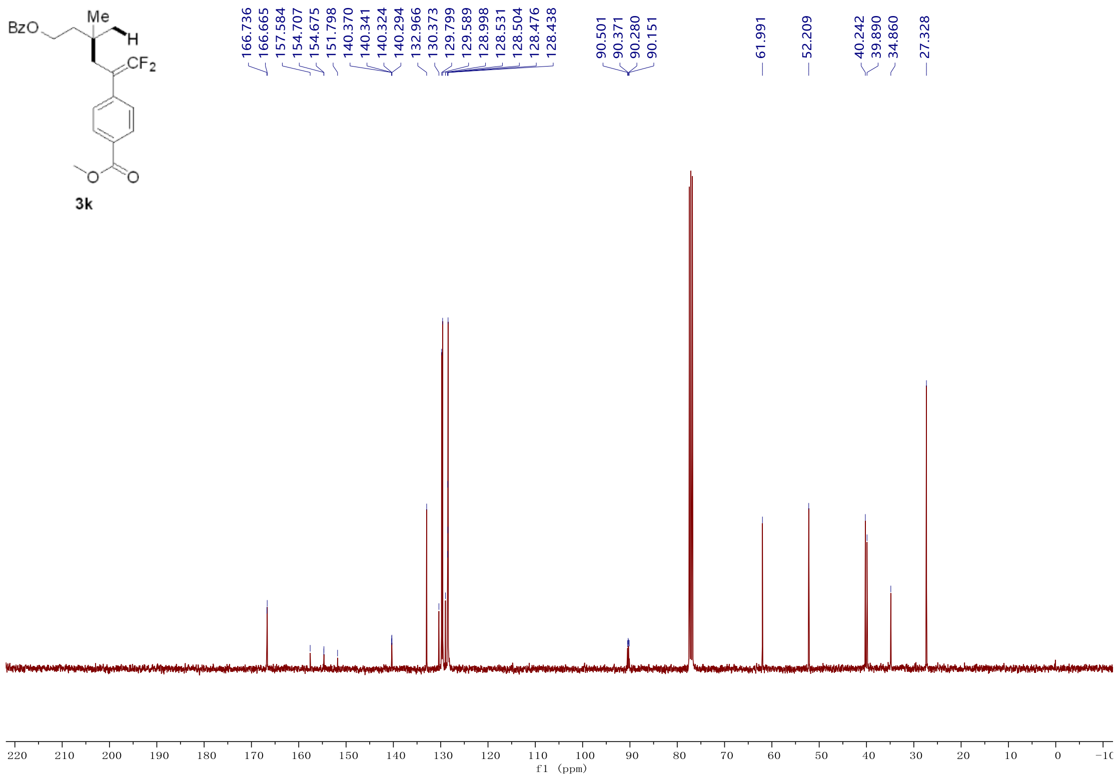

Supplementary Figure 133. ^{13}C NMR spectrum of compound **3k**

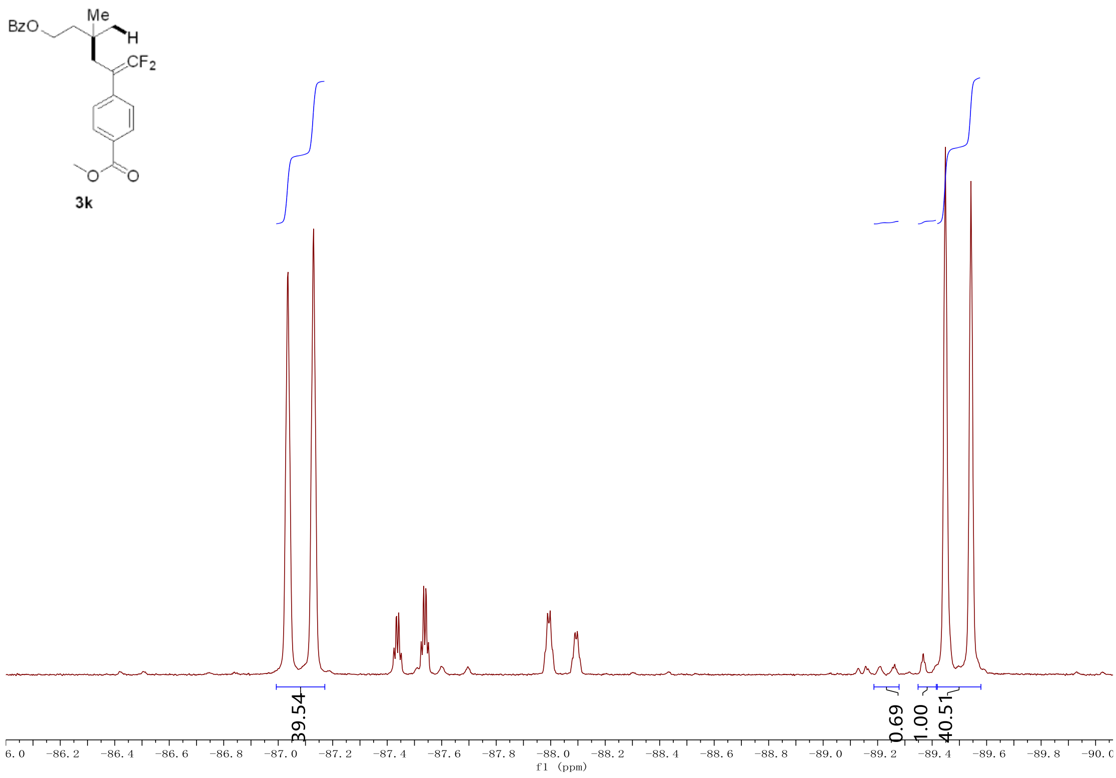

Supplementary Figure 134. Crude ^{19}F NMR spectrum of compound **3k** rr = 40 : 1

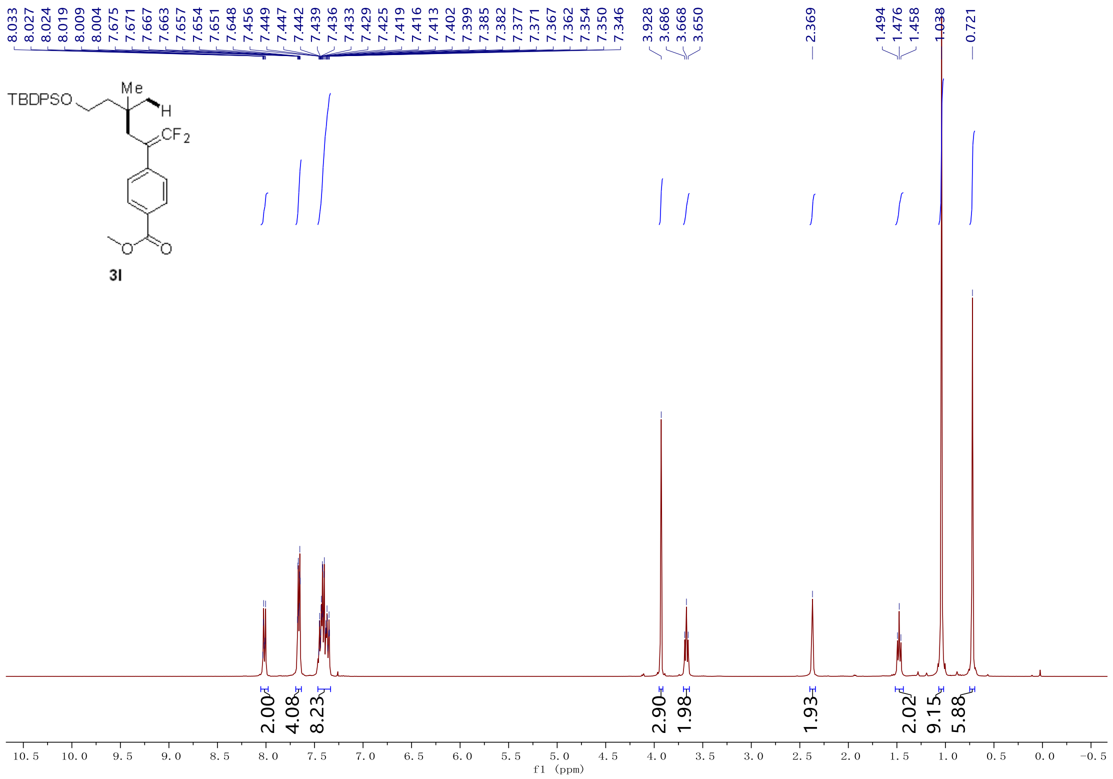

Supplementary Figure 135. ^{1}H NMR spectrum of compound **3l**

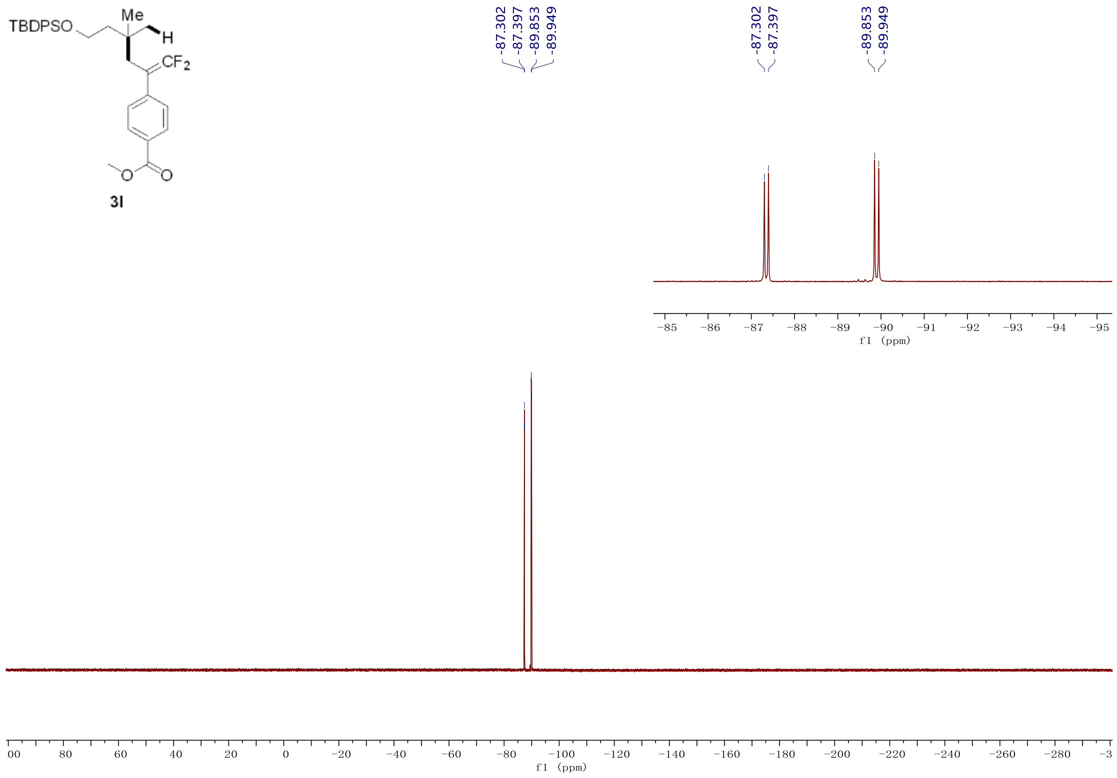

Supplementary Figure 136. ^{19}F NMR spectrum of compound **3l**

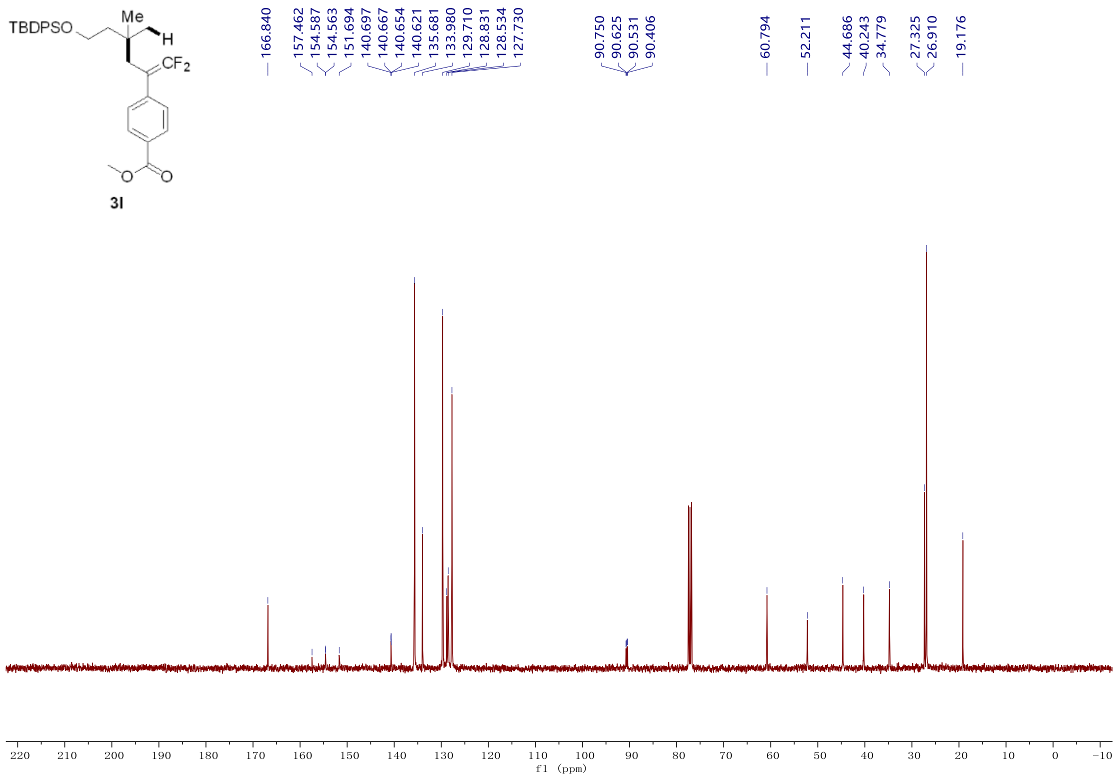

Supplementary Figure 137. ^{13}C NMR spectrum of compound **3l**

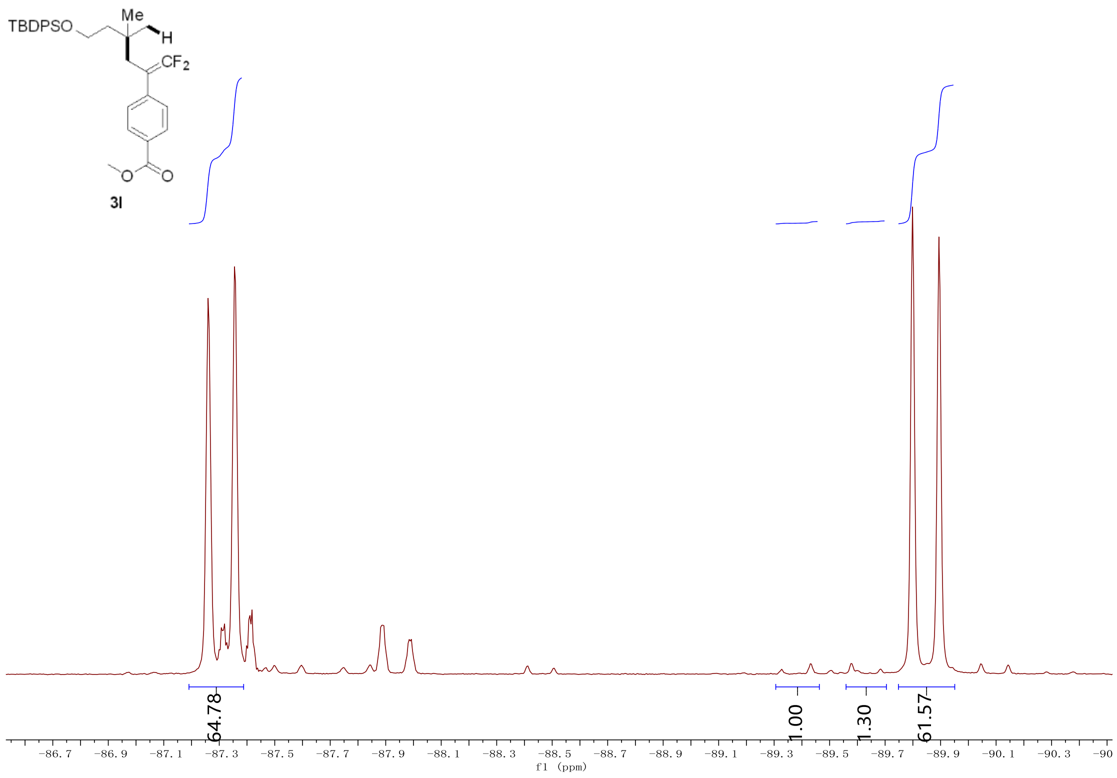

Supplementary Figure 138. Crude ^{19}F NMR spectrum of compound **3l** rr = 61 : 1

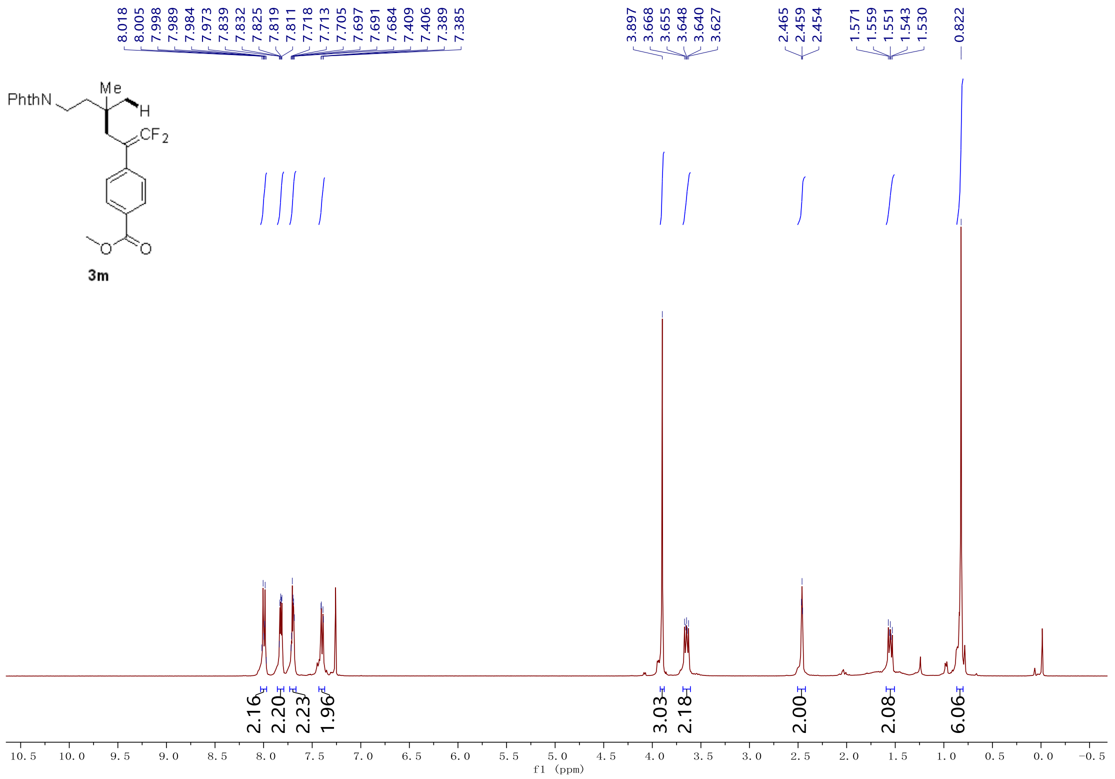

Supplementary Figure 139. ^{1}H NMR spectrum of compound **3m**

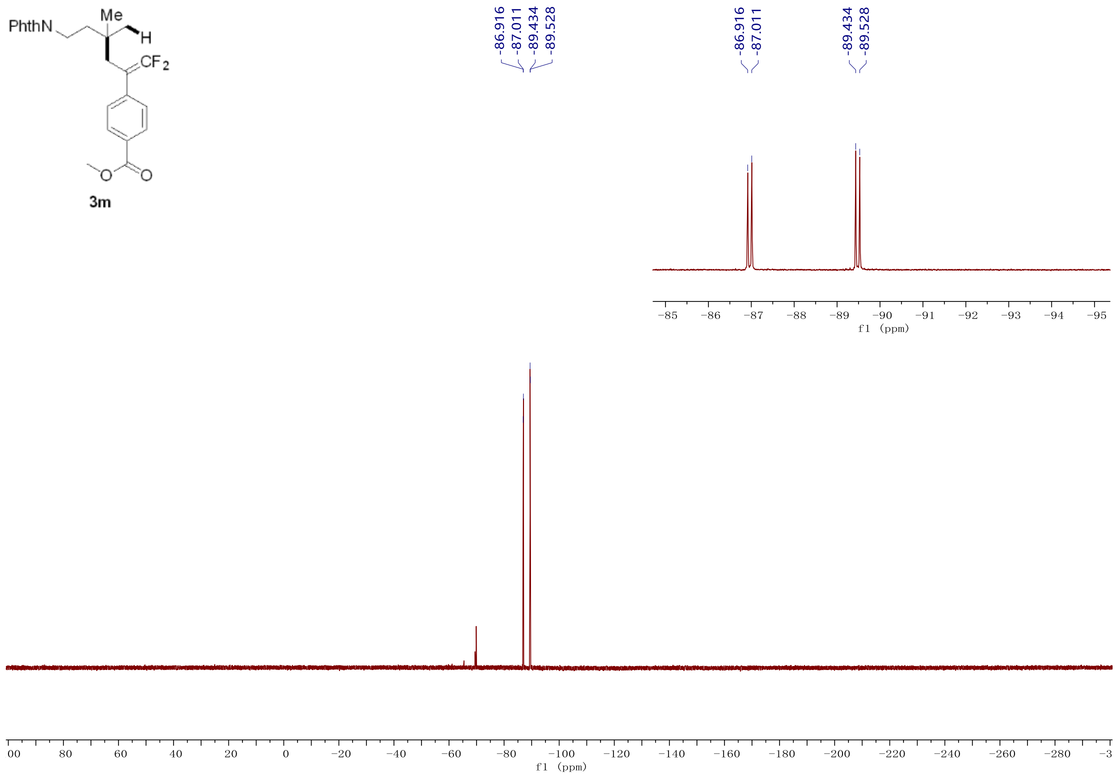

Supplementary Figure 140. ^{19}F NMR spectrum of compound **3m**

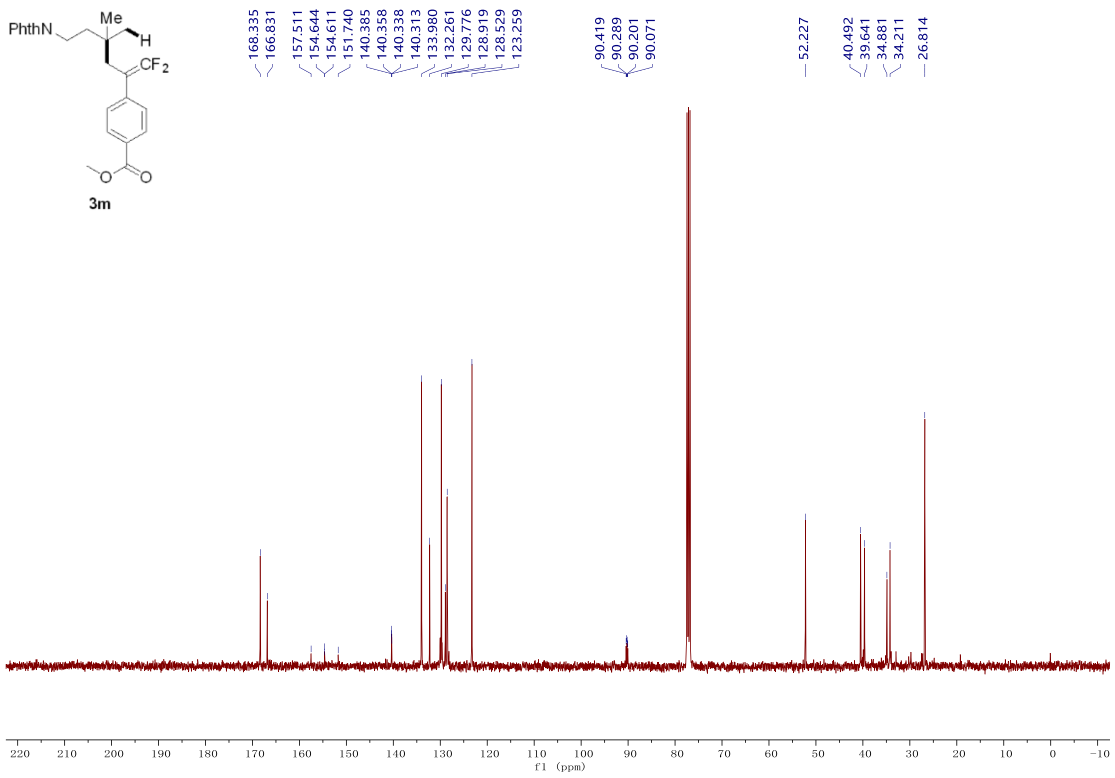

Supplementary Figure 141. ^{13}C NMR spectrum of compound **3m**

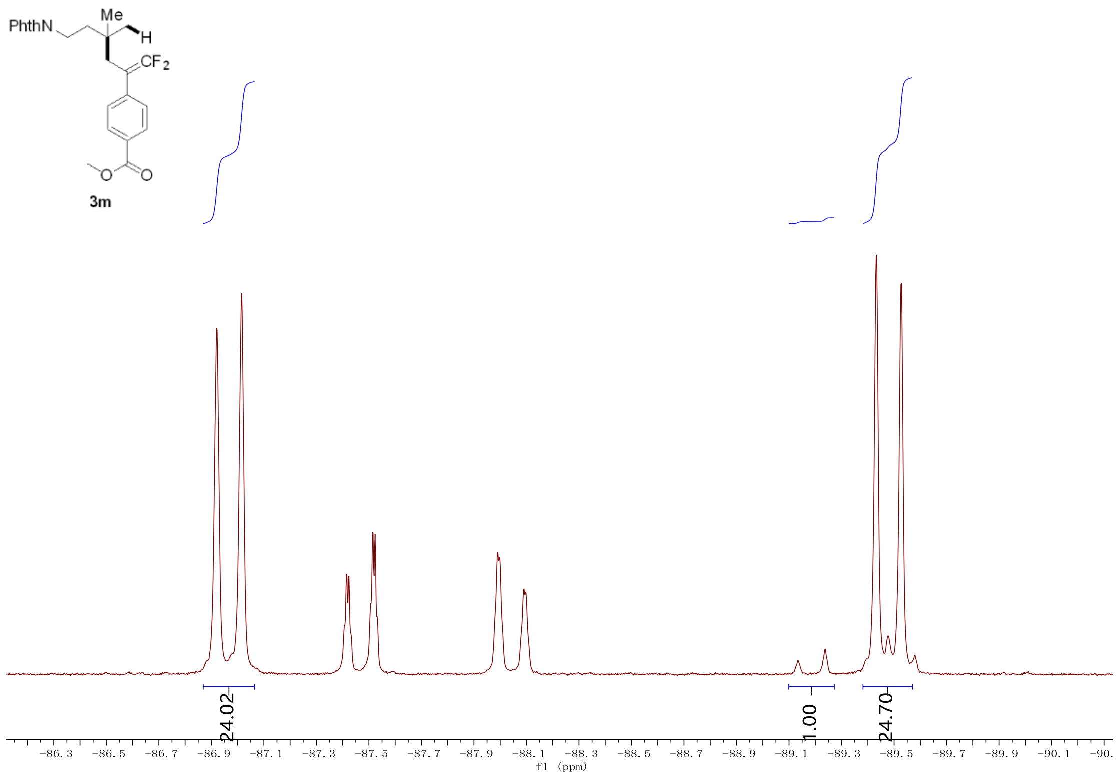

Supplementary Figure 142. Crude ^{19}F NMR spectrum of compound **3m** rr = 24 : 1

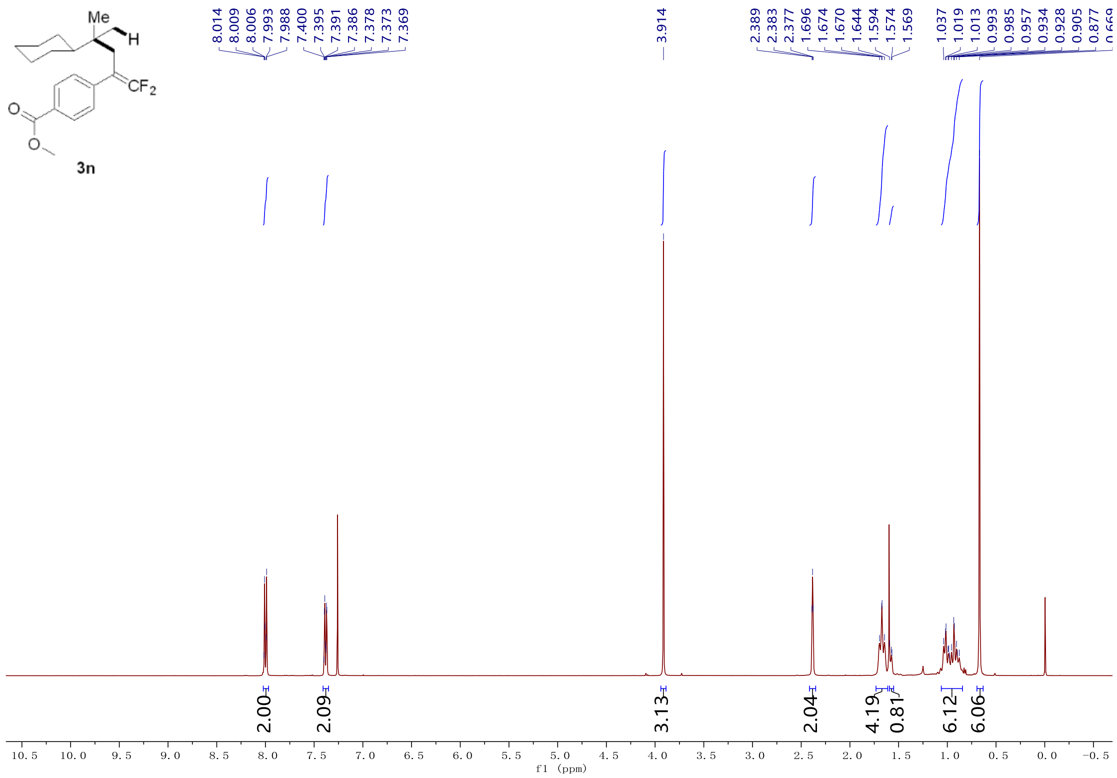

Supplementary Figure 143. ^{1}H NMR spectrum of compound **3n**

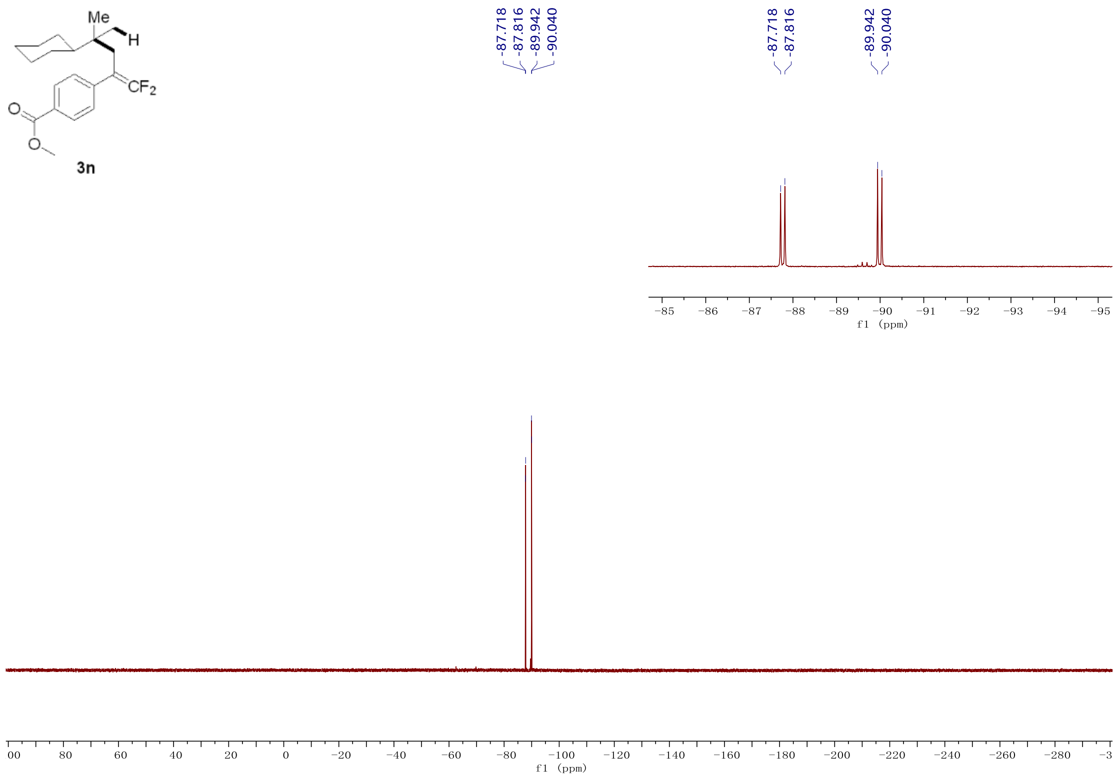

Supplementary Figure 144. ^{19}F NMR spectrum of compound **3n**

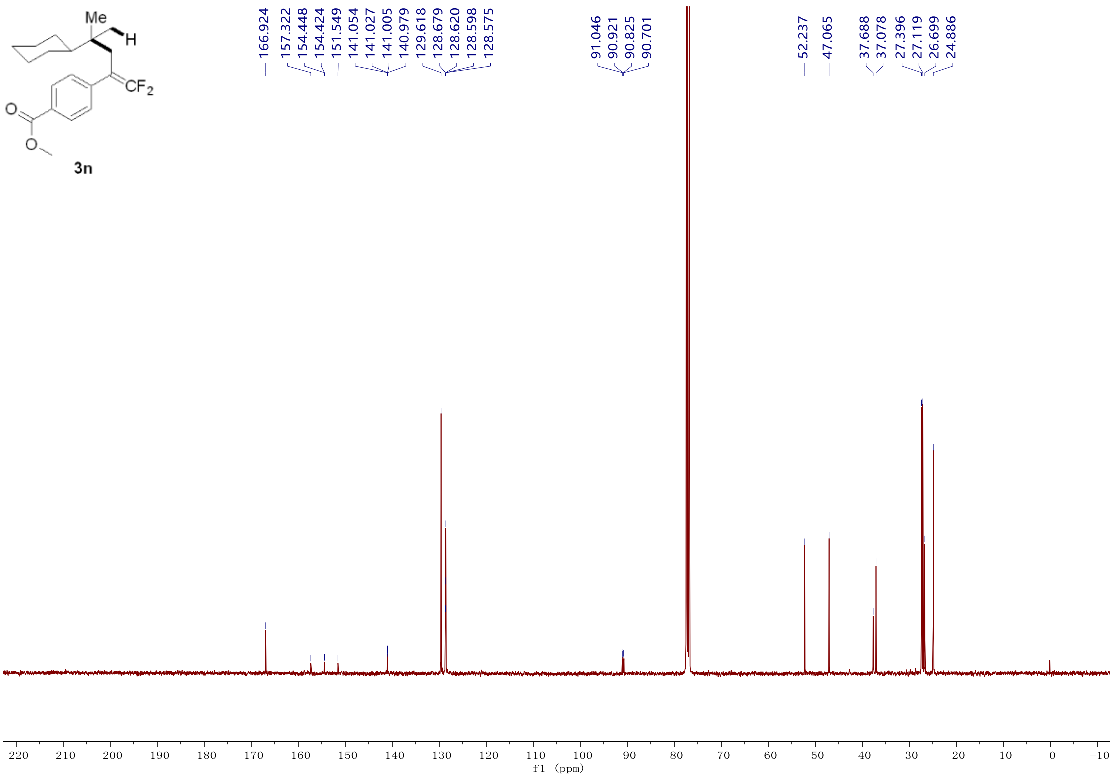

Supplementary Figure 145. ^{13}C NMR spectrum of compound **3n**

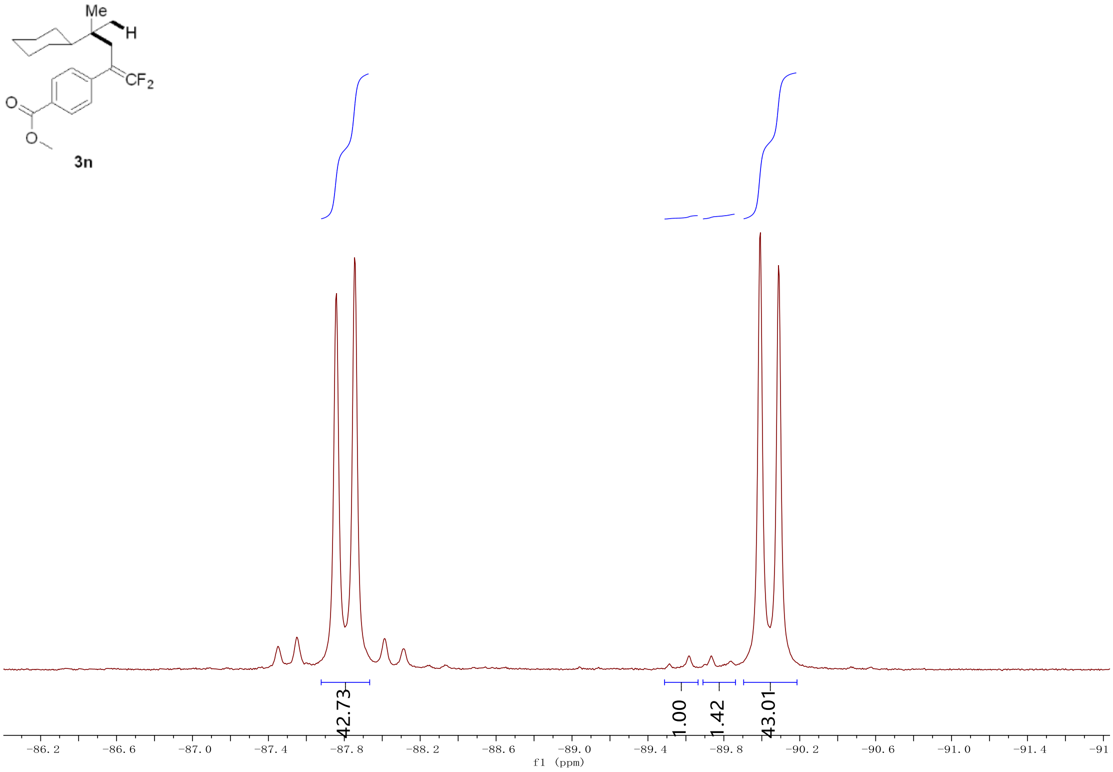

Supplementary Figure 146. Crude ^{19}F NMR spectrum of compound **3n** rr = 43 : 1

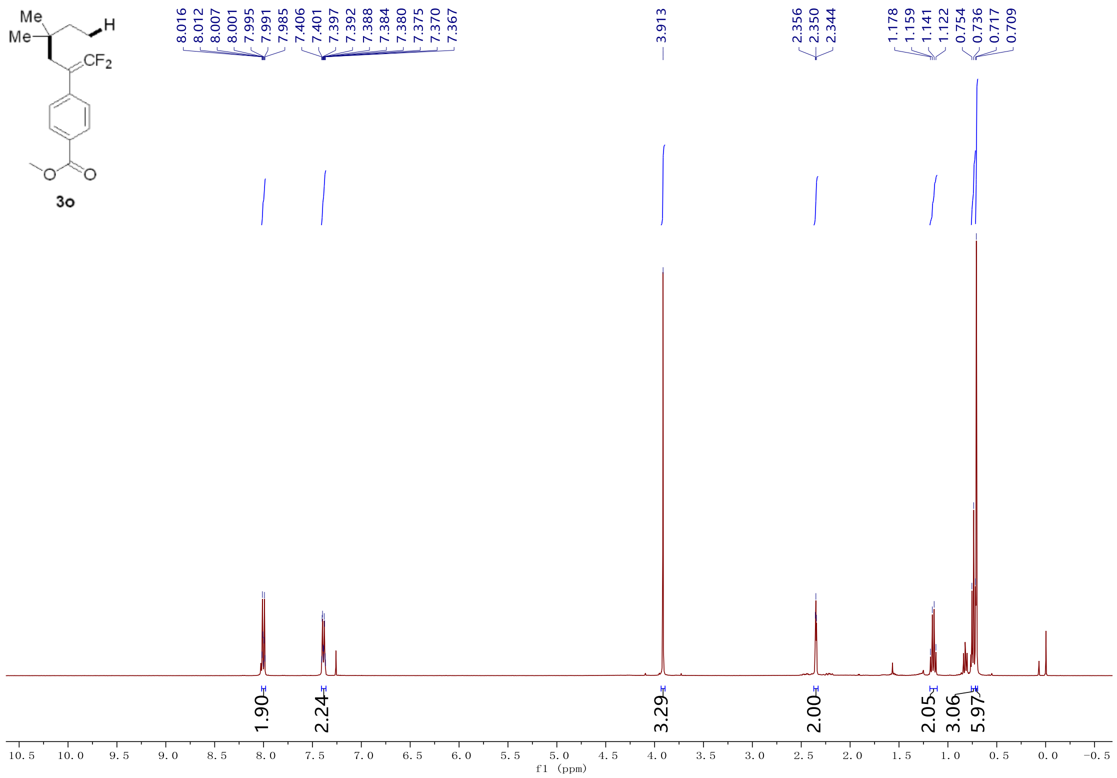

Supplementary Figure 147. ^{1}H NMR spectrum of compound **3o**

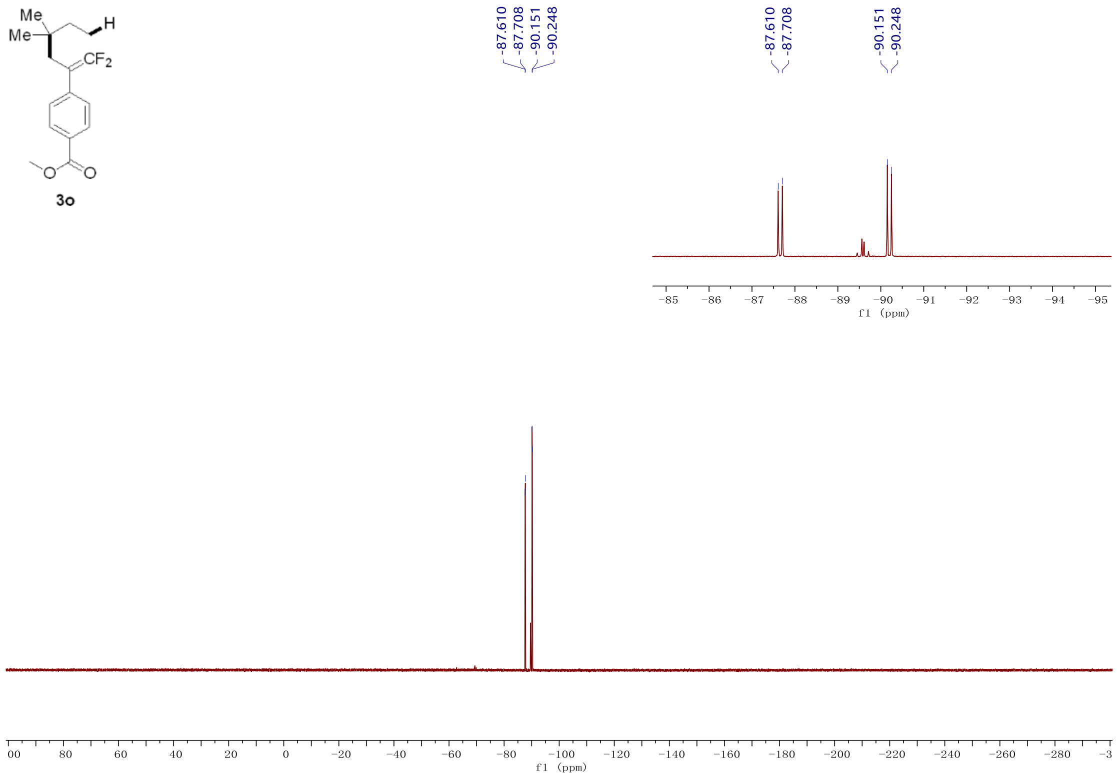

Supplementary Figure 148. ^{19}F NMR spectrum of compound **3o**

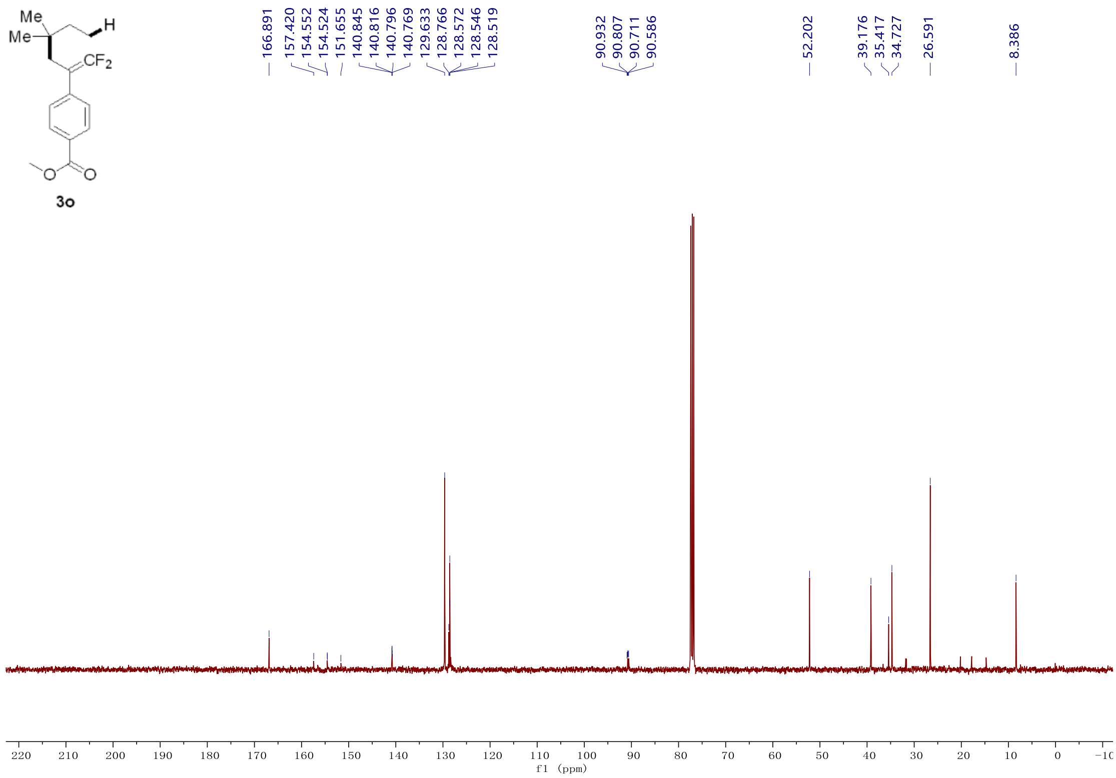

Supplementary Figure 149. ^{13}C NMR spectrum of compound **3o**

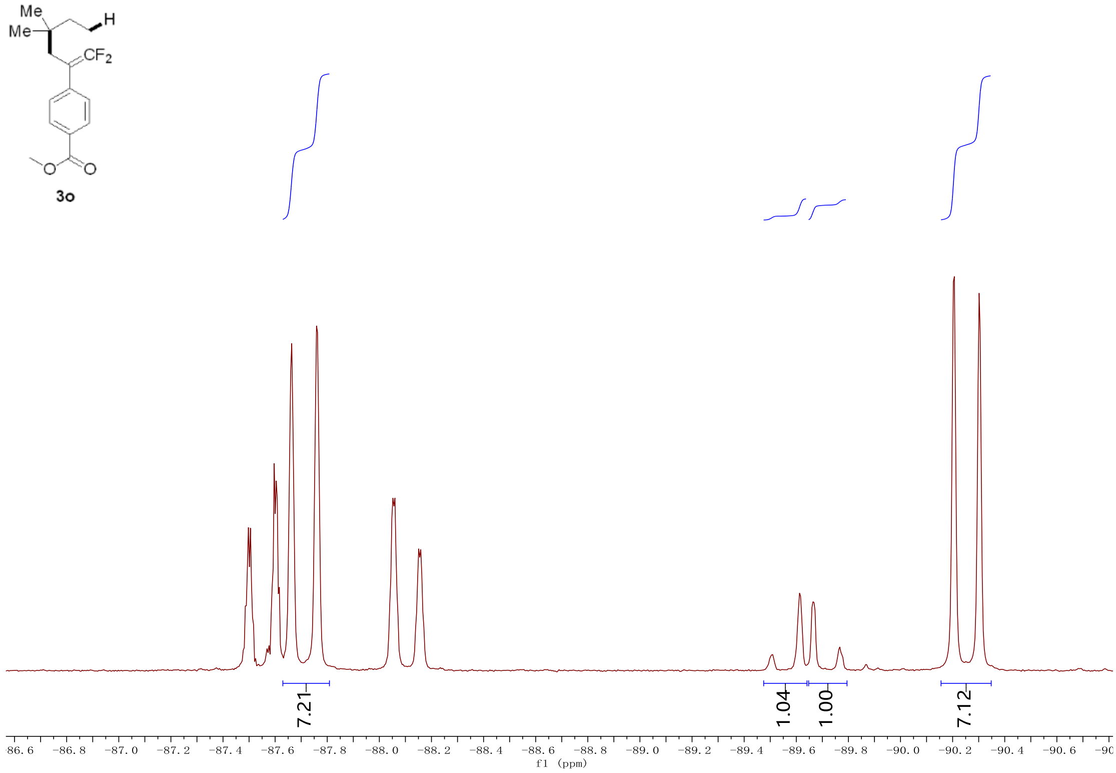

Supplementary Figure 150. Crude ^{19}F NMR spectrum of compound **3o** rr = 7 : 1

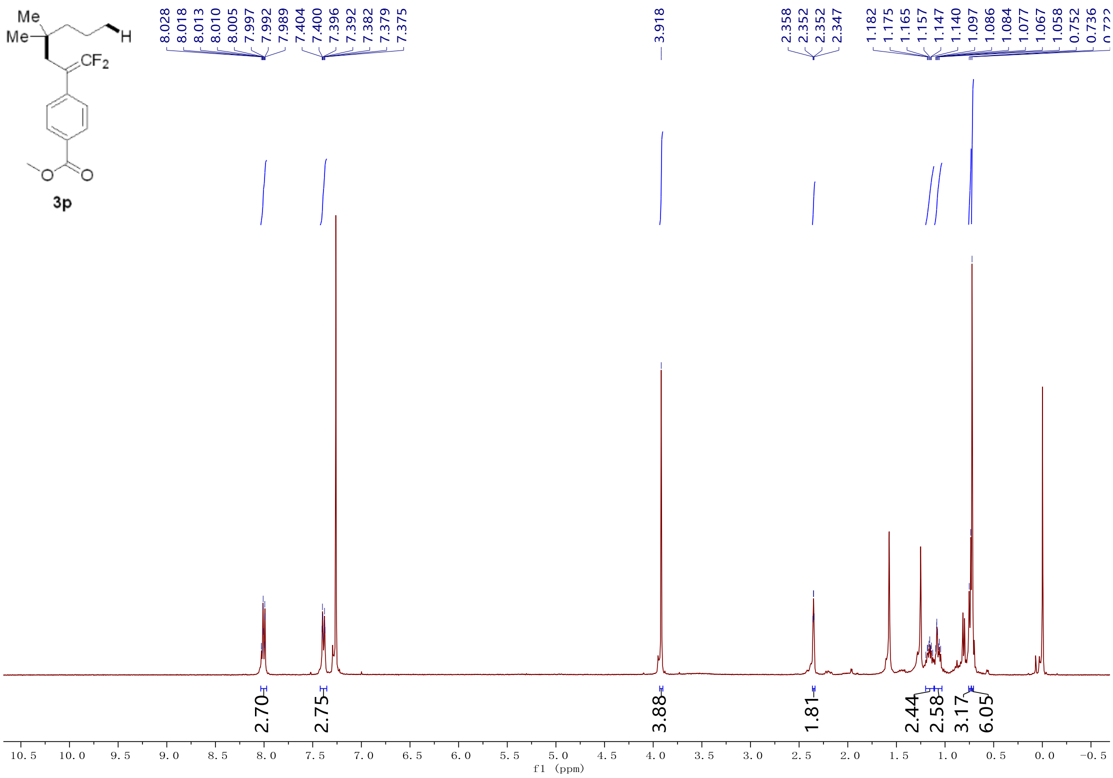

Supplementary Figure 151. ^{1}H NMR spectrum of compound **3p**

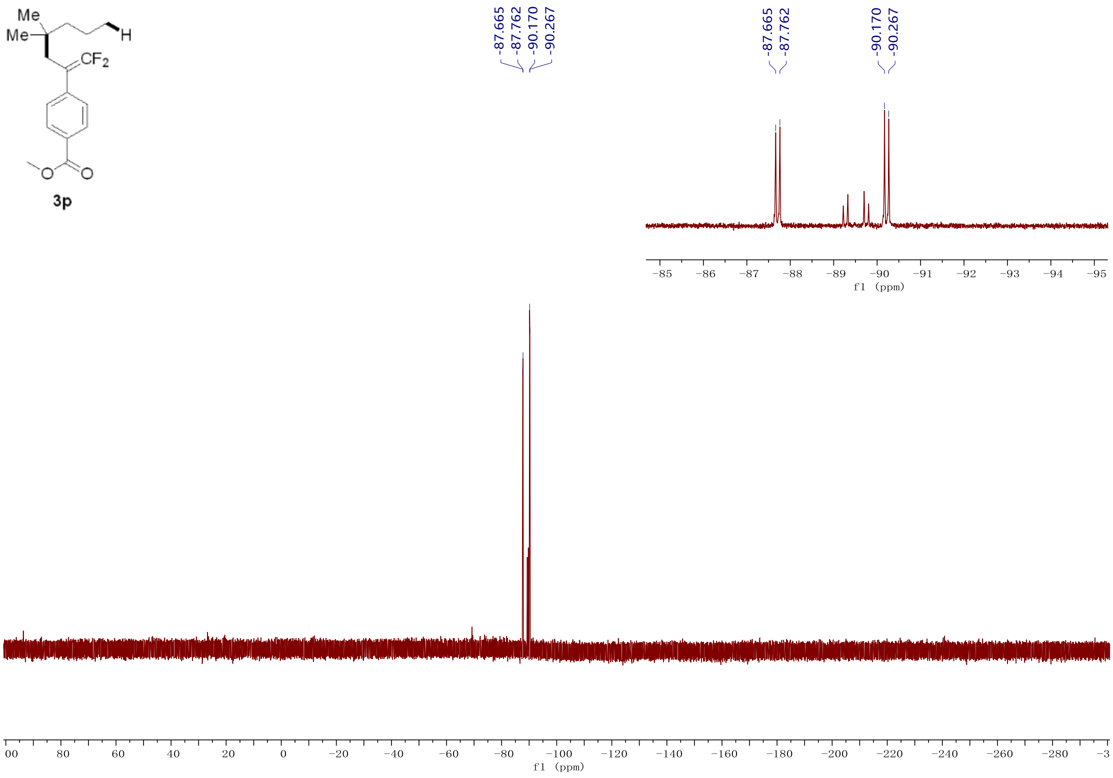

Supplementary Figure 152. ^{19}F NMR spectrum of compound **3p**

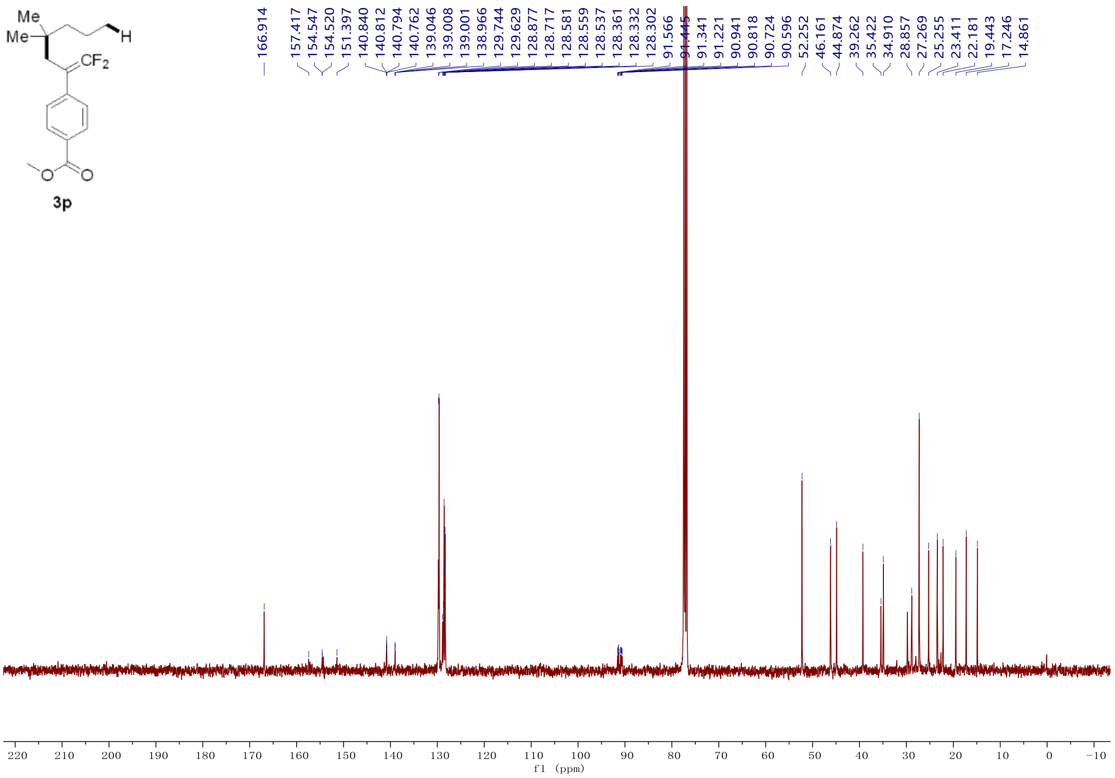

Supplementary Figure 153. ^{13}C NMR spectrum of compound **3p**

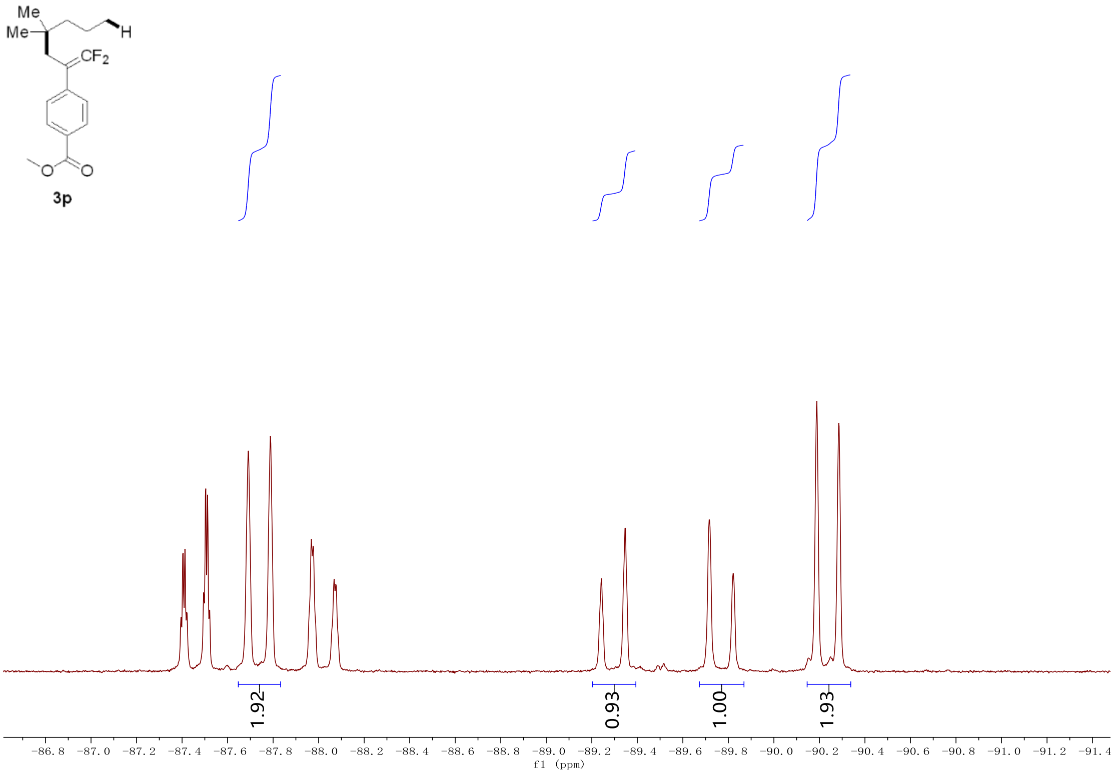

Supplementary Figure 154. Crude ^{19}F NMR spectrum of compound **3p** rr = 2 : 1

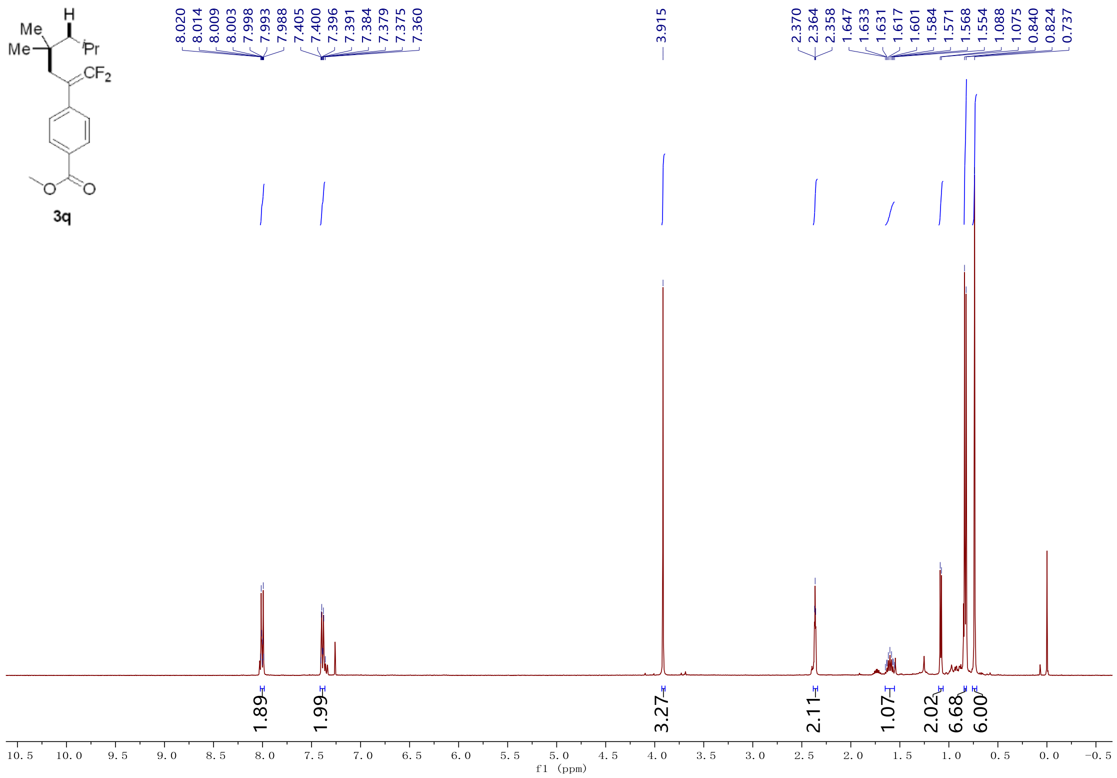

Supplementary Figure 155. ^{1}H NMR spectrum of compound **3q**

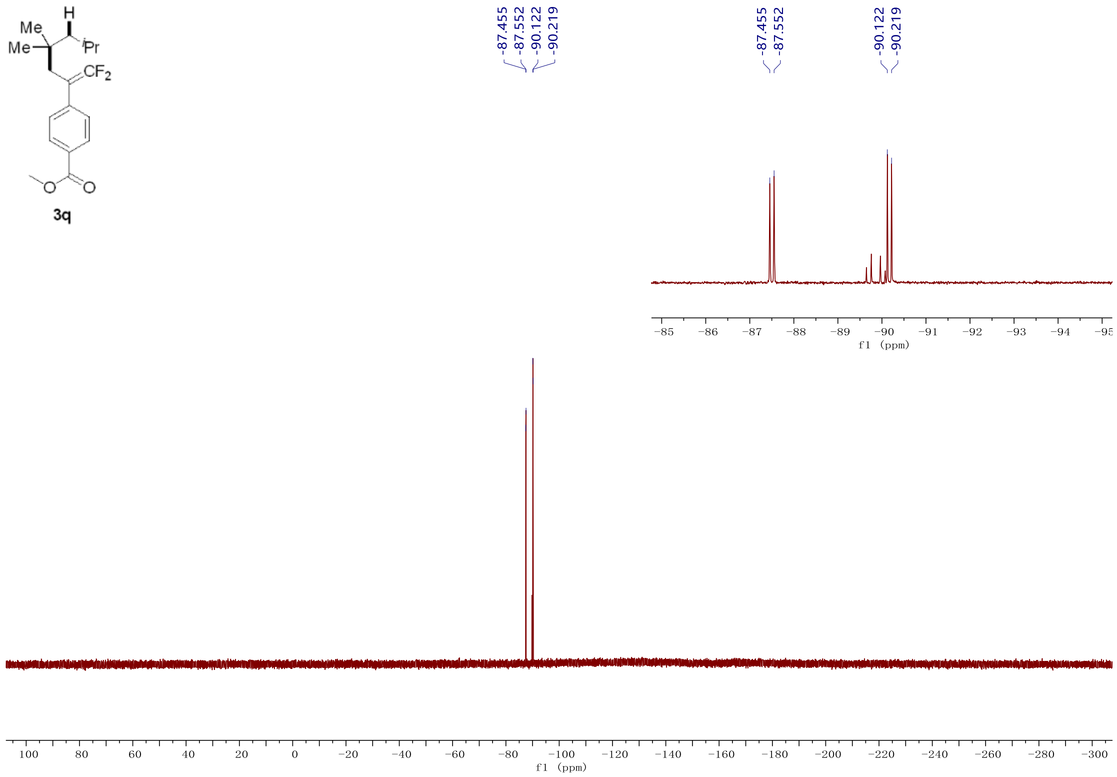

Supplementary Figure 156. ^{19}F NMR spectrum of compound **3q**

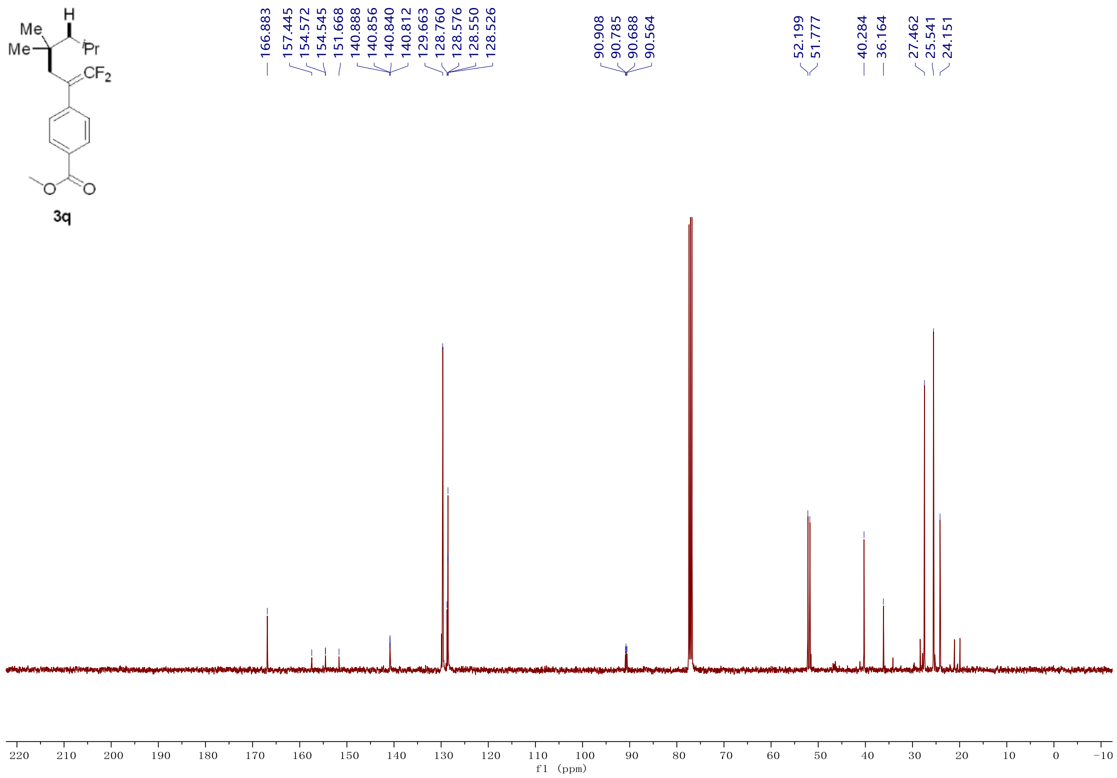

Supplementary Figure 157. ^{13}C NMR spectrum of compound **3q**

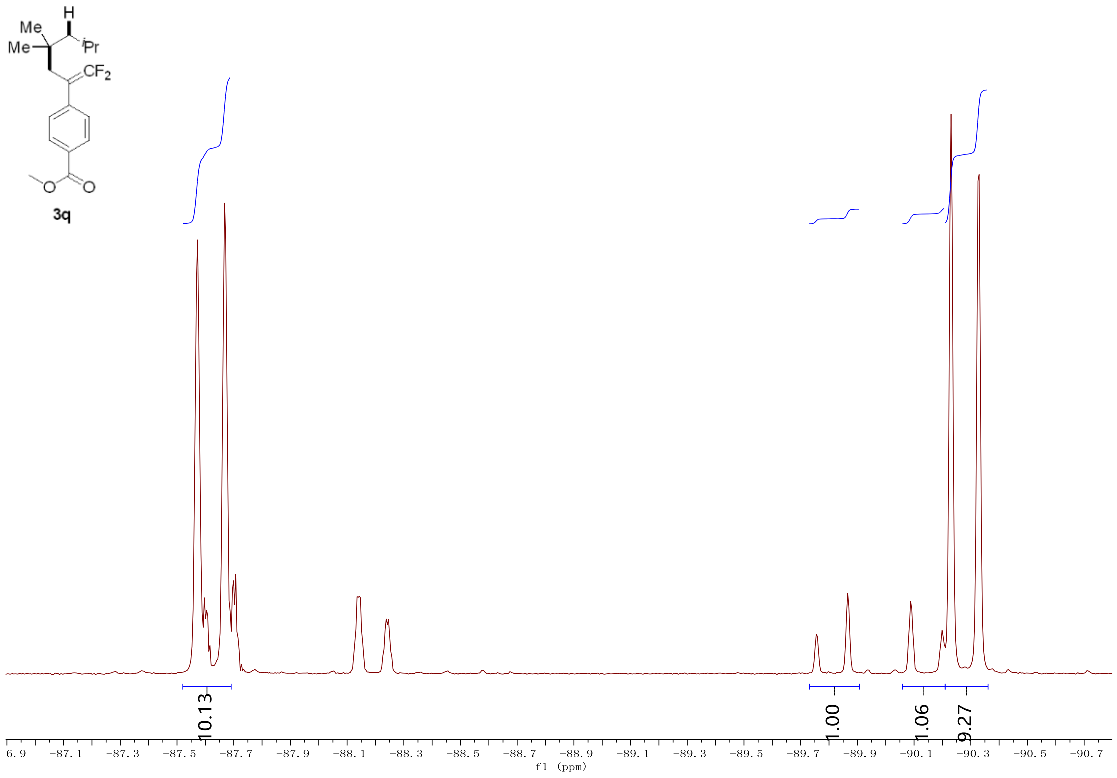

Supplementary Figure 158. Crude ^{19}F NMR spectrum of compound **3q** rr = 9 : 1

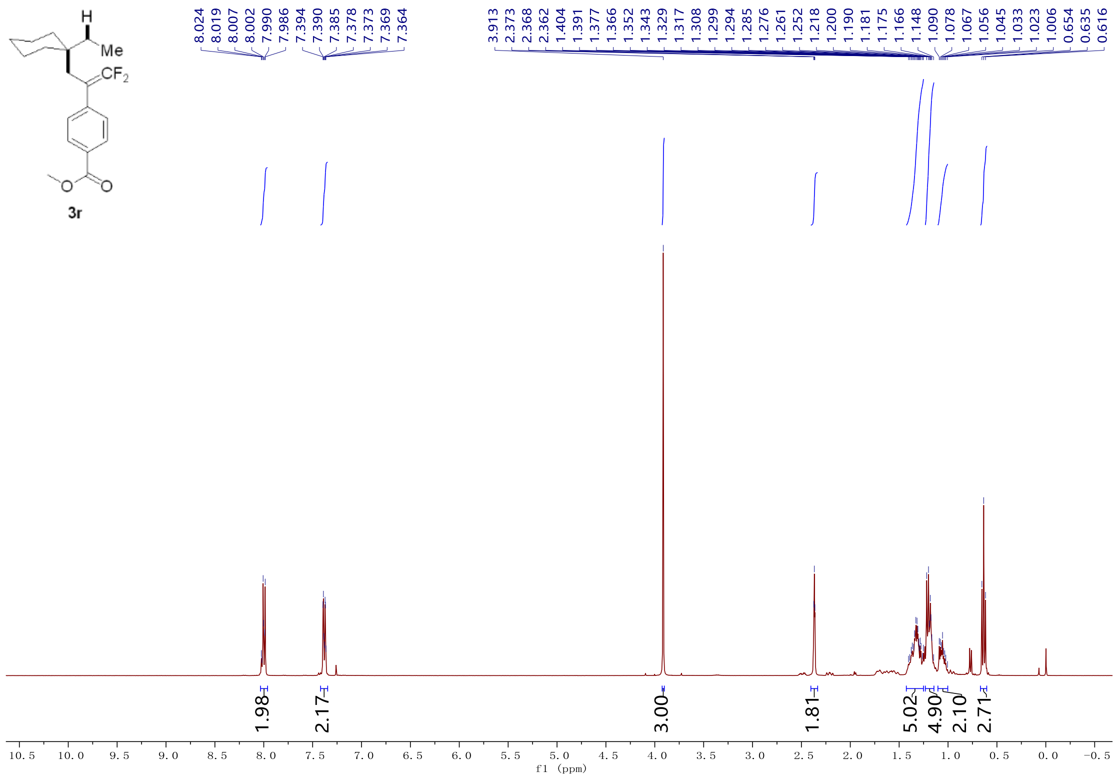

Supplementary Figure 159. ^{1}H NMR spectrum of compound **3r**

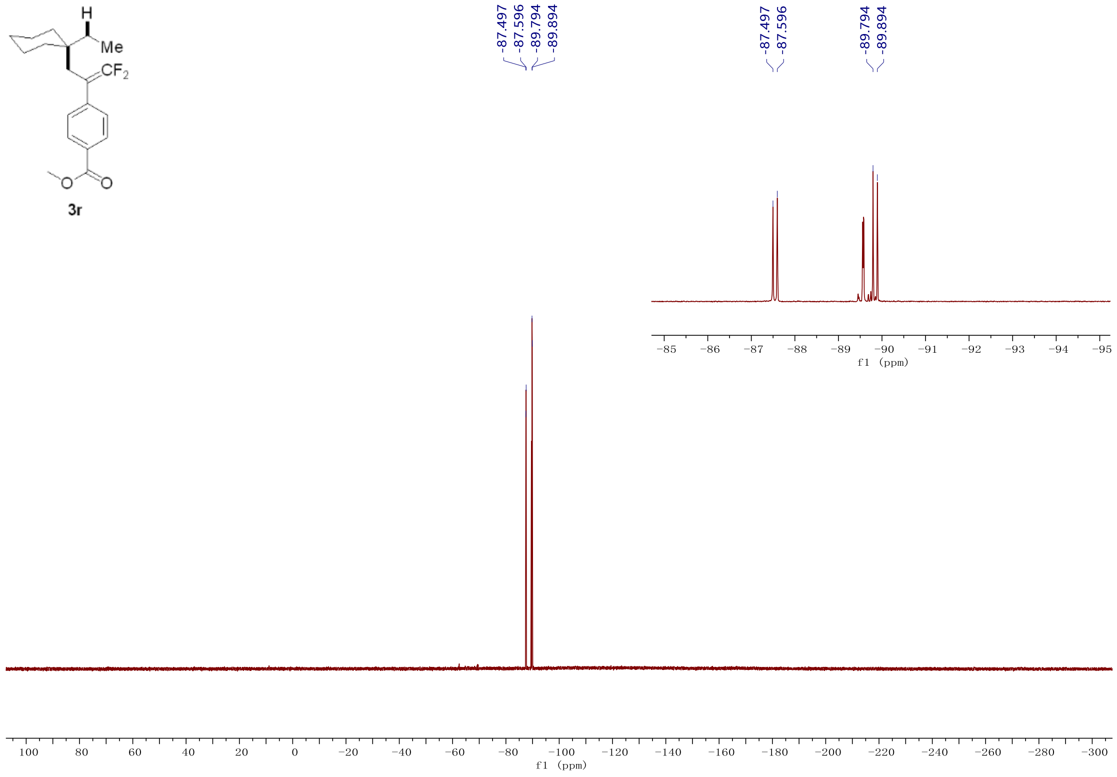

Supplementary Figure 160. ^{19}F NMR spectrum of compound **3r**

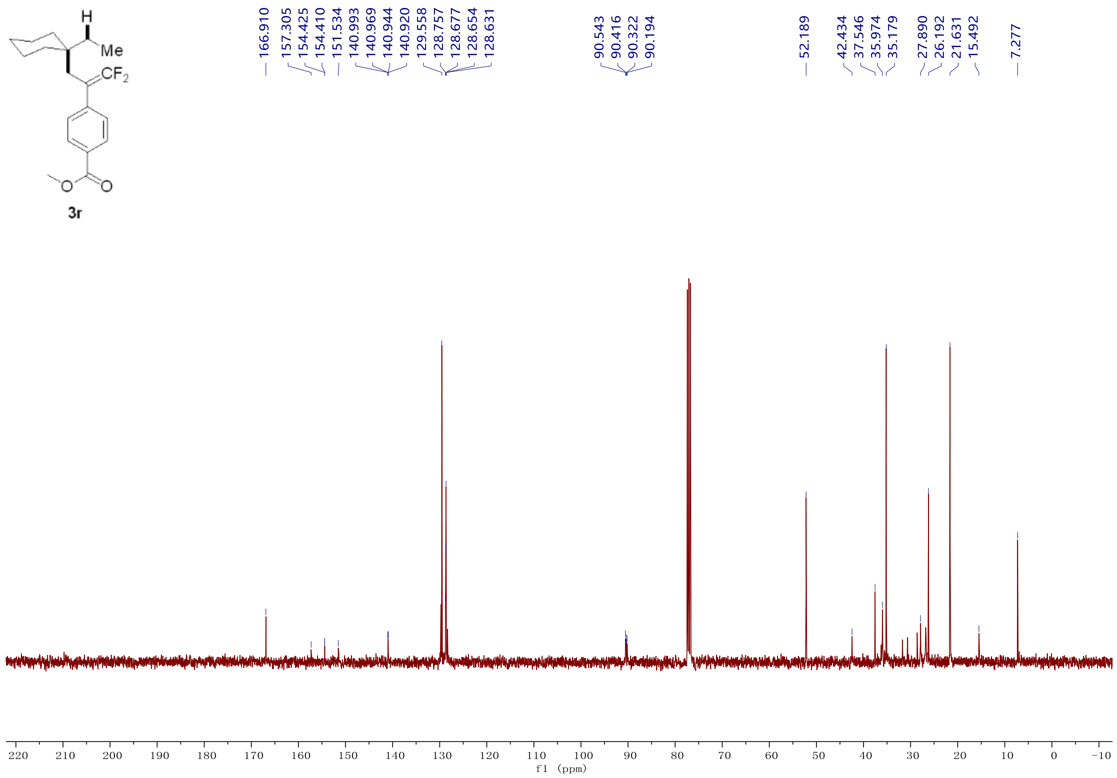

Supplementary Figure 161. ^{13}C NMR spectrum of compound **3r**

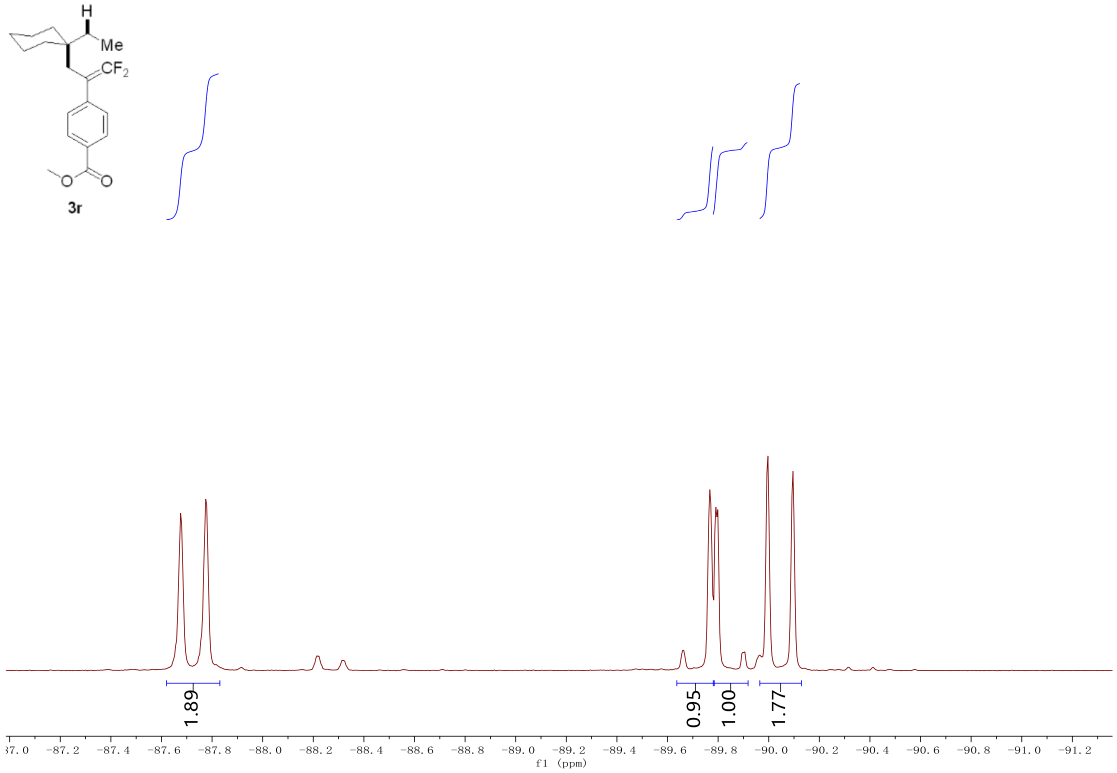

Supplementary Figure 162. Crude ^{19}F NMR spectrum of compound **3r** rr = 2 : 1

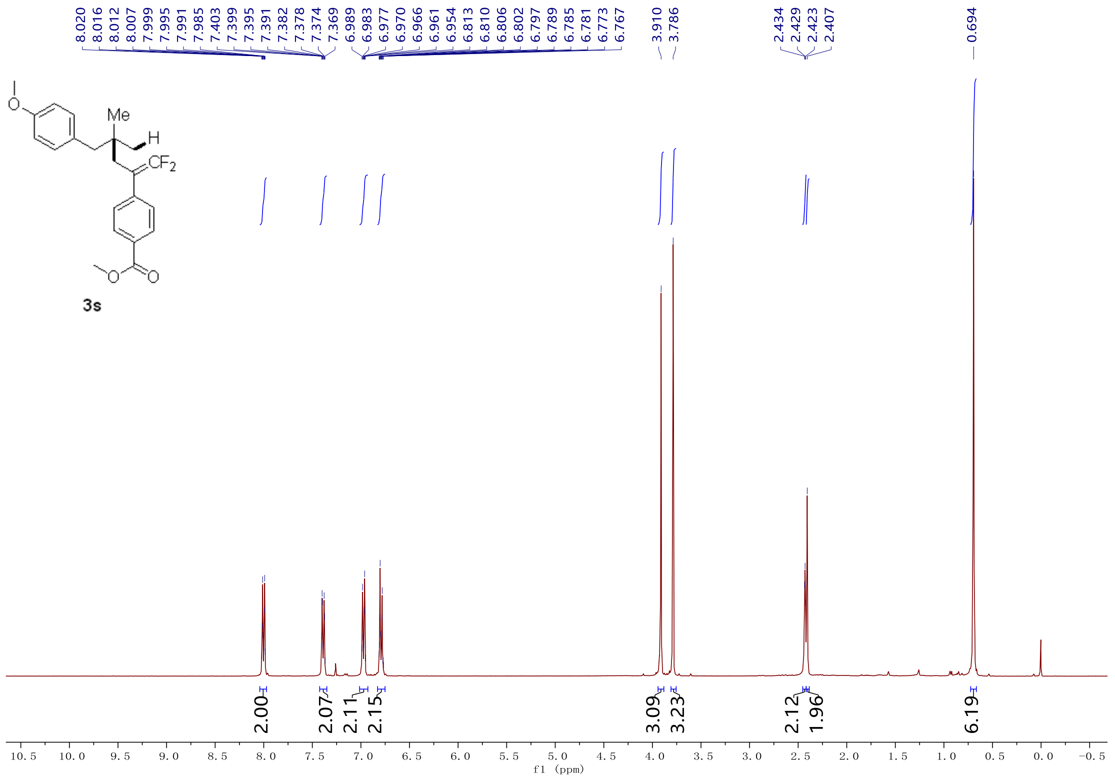

Supplementary Figure 163. ^{1}H NMR spectrum of compound **3s**

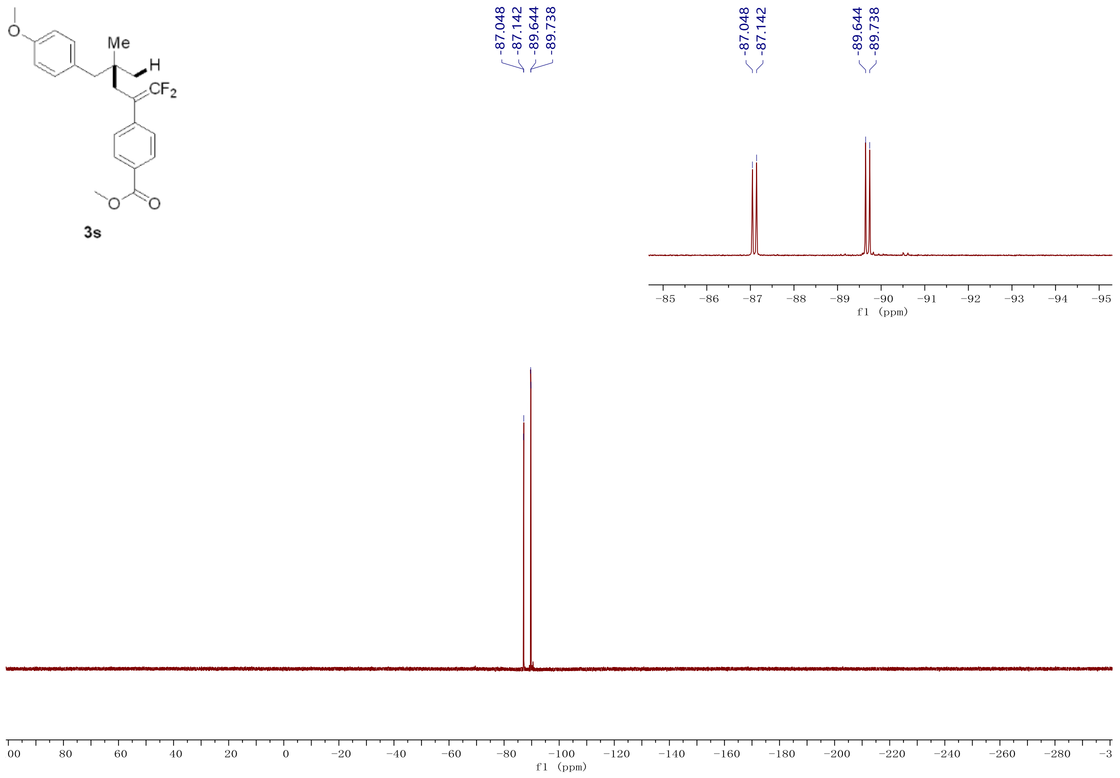

Supplementary Figure 164. ^{19}F NMR spectrum of compound **3s**

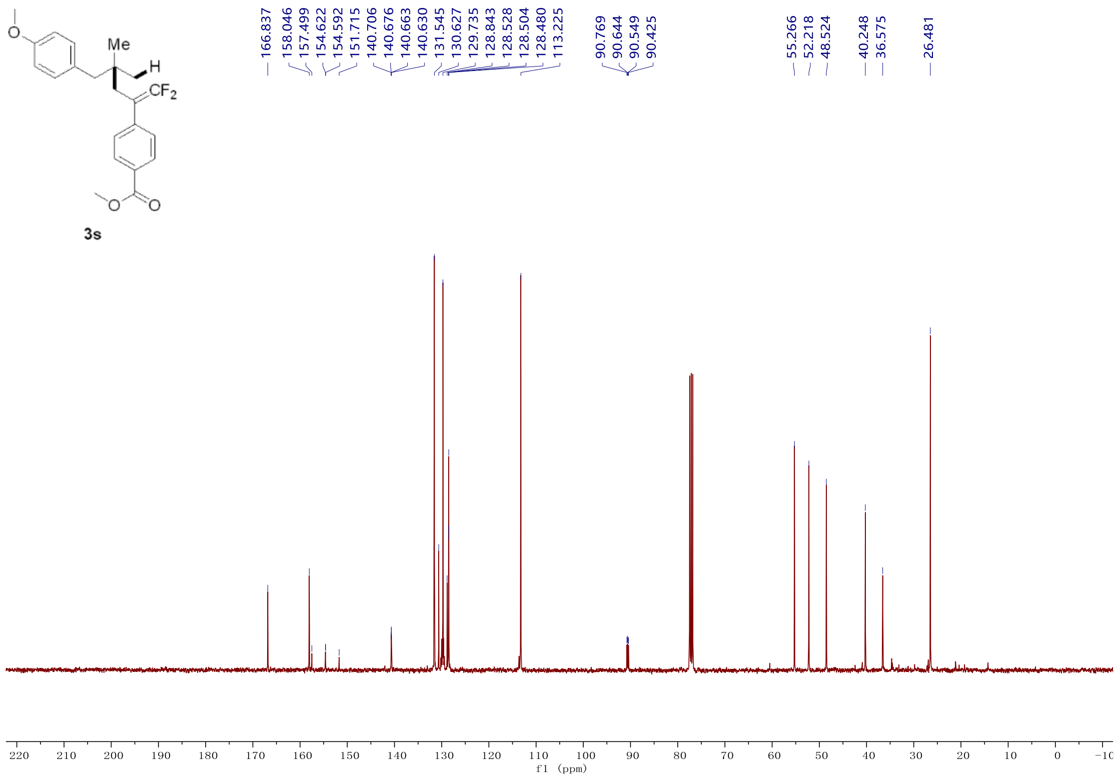

Supplementary Figure 165. ^{13}C NMR spectrum of compound **3s**

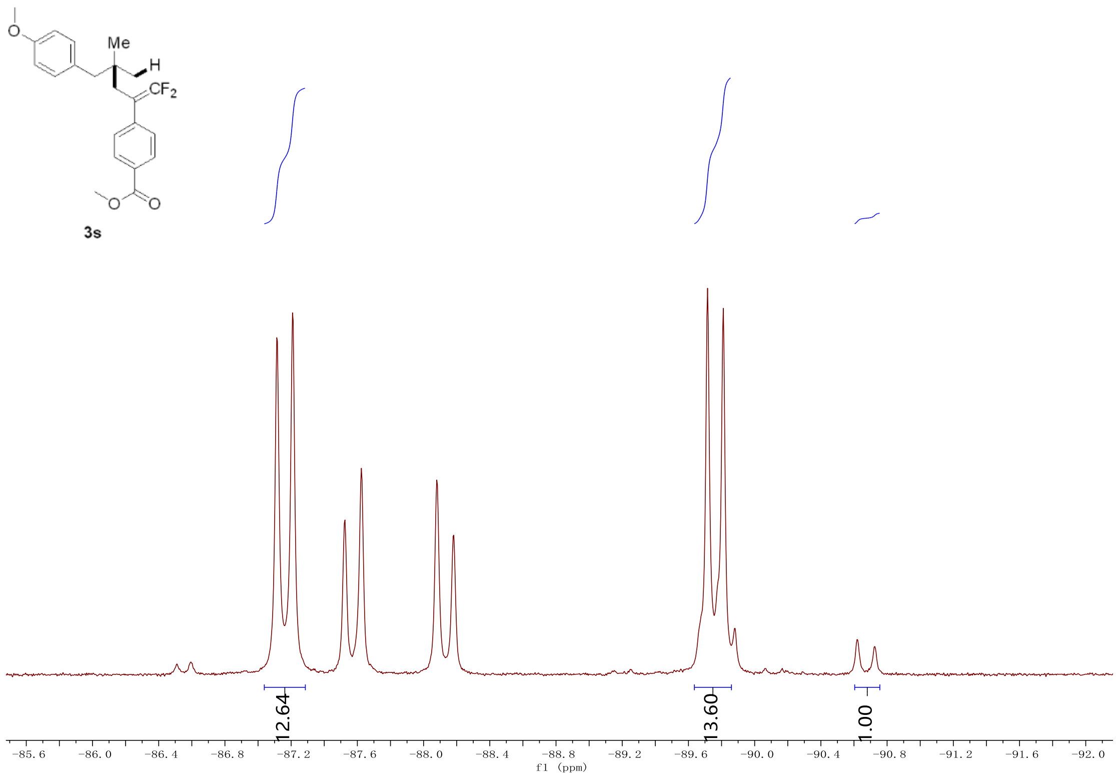

Supplementary Figure 166. Crude ^{19}F NMR spectrum of compound **3s** rr = 12： 1

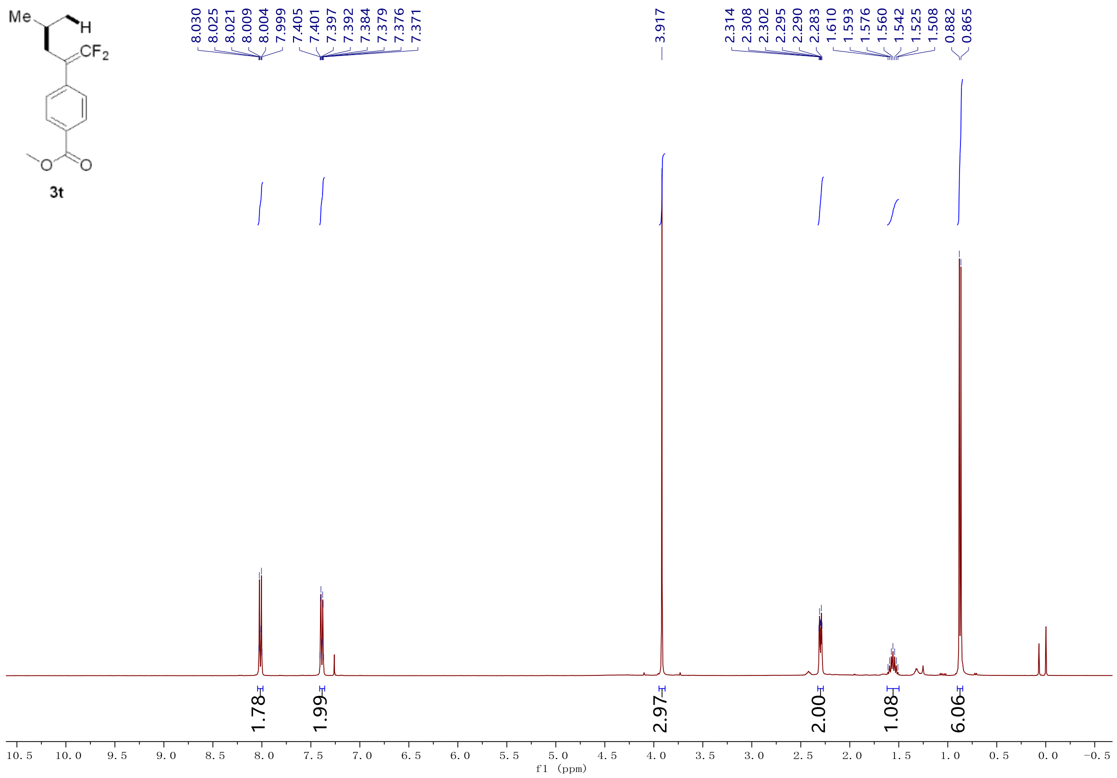

Supplementary Figure 167. ^{1}H NMR spectrum of compound **3t**

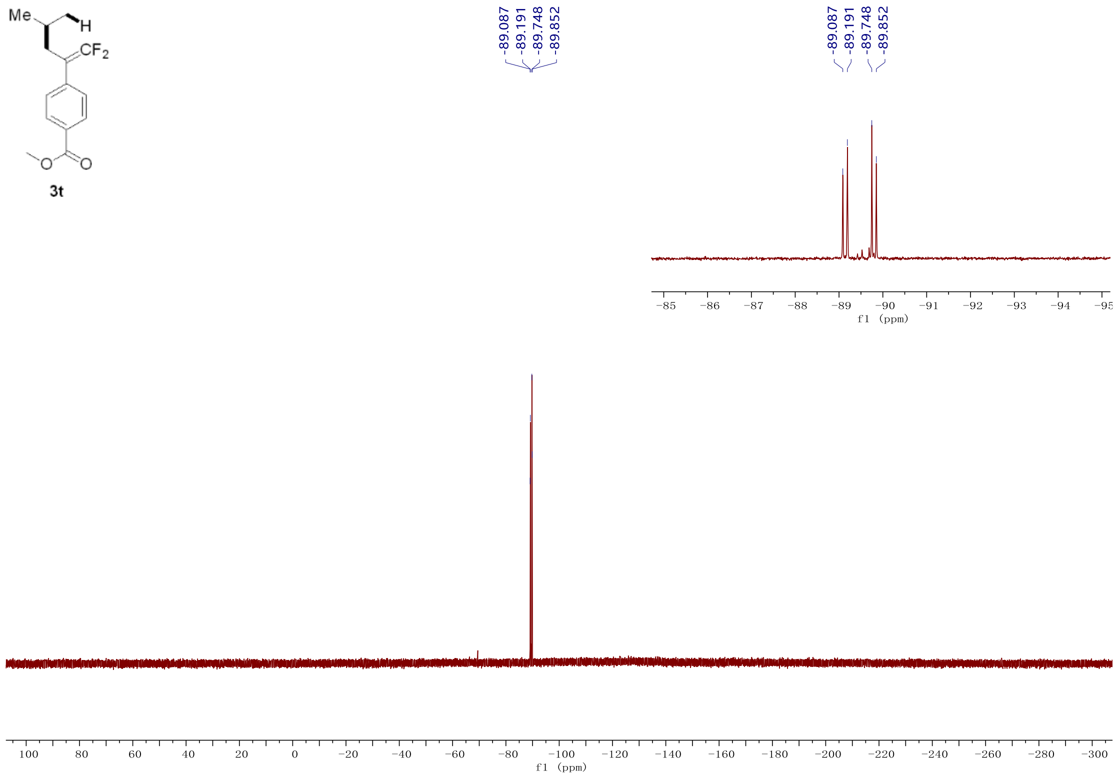

Supplementary Figure 168. ^{19}F NMR spectrum of compound **3t**

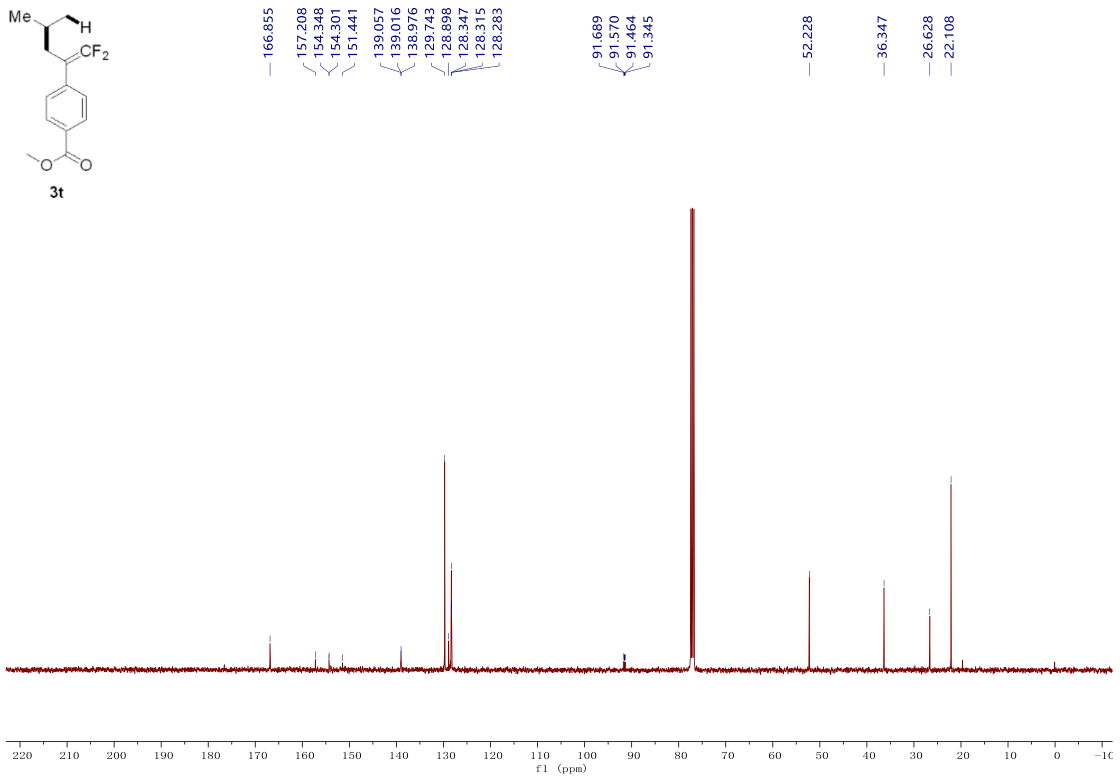

Supplementary Figure 169. ^{13}C NMR spectrum of compound **3t**

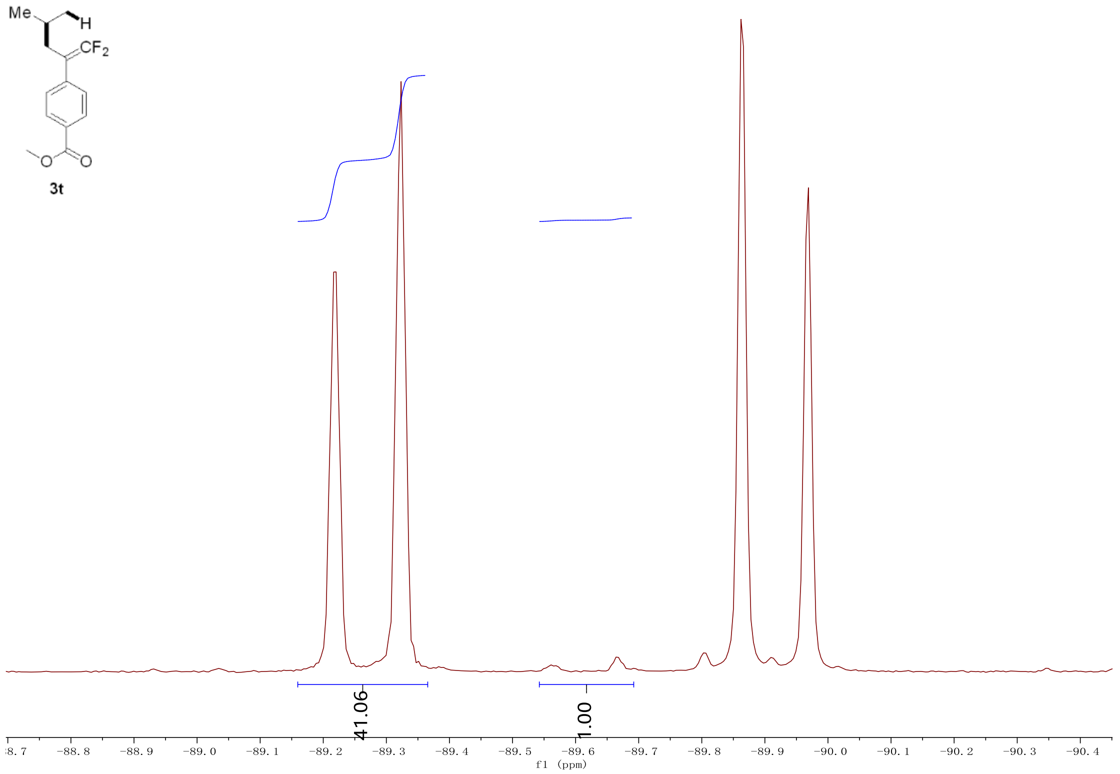

Supplementary Figure 170. Crude ^{19}F NMR spectrum of compound **3t** rr = 41 : 1

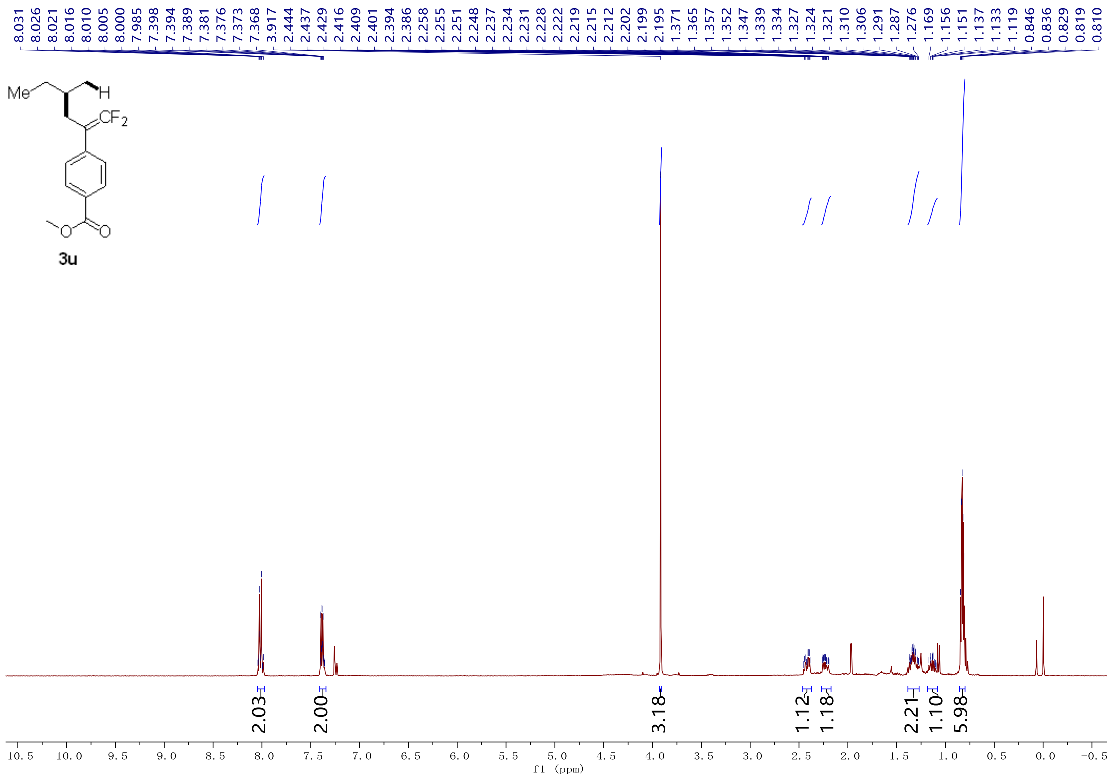

Supplementary Figure 171. ^{1}H NMR spectrum of compound **3u**

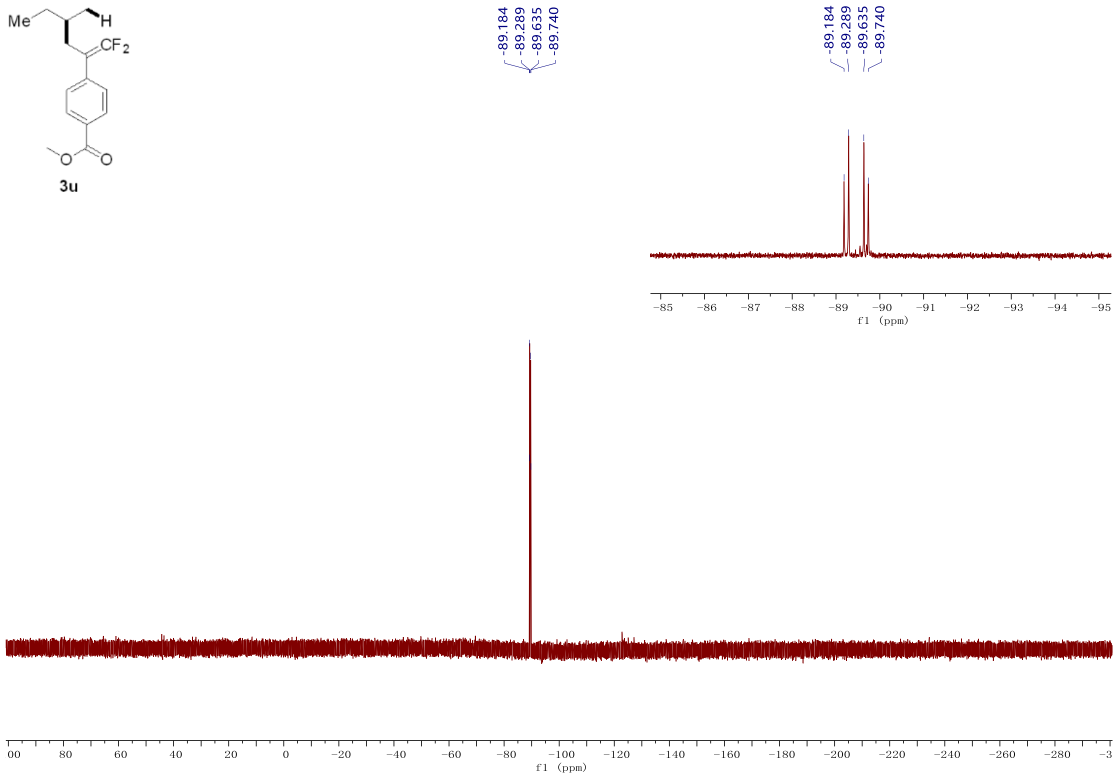

Supplementary Figure 172. ^{19}F NMR spectrum of compound **3u**

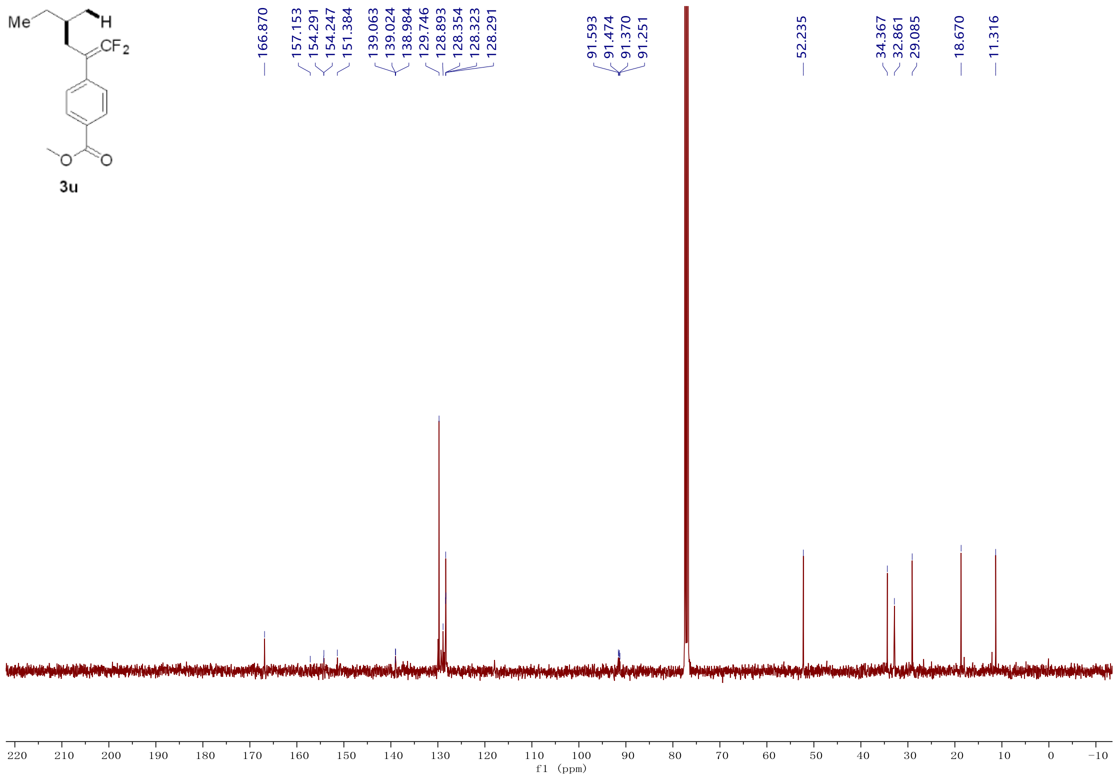

Supplementary Figure 173. ^{13}C NMR spectrum of compound **3u**

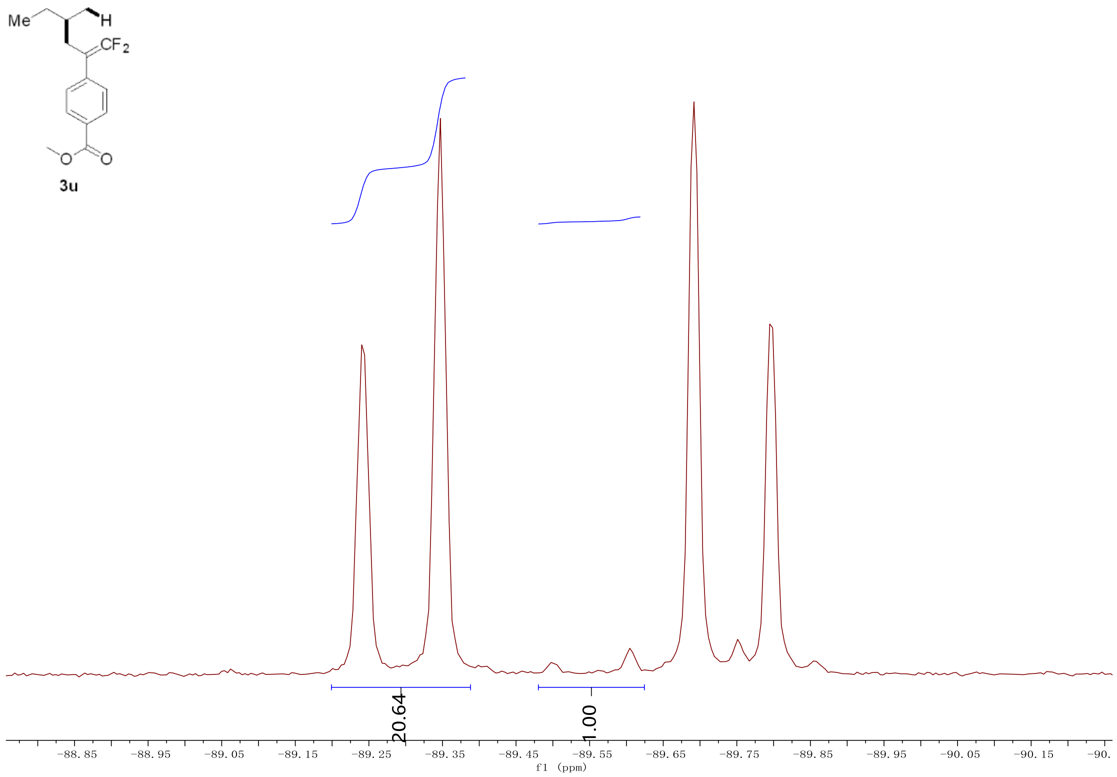

Supplementary Figure 174. Crude ^{19}F NMR spectrum of compound **3u** rr = 20 : 1

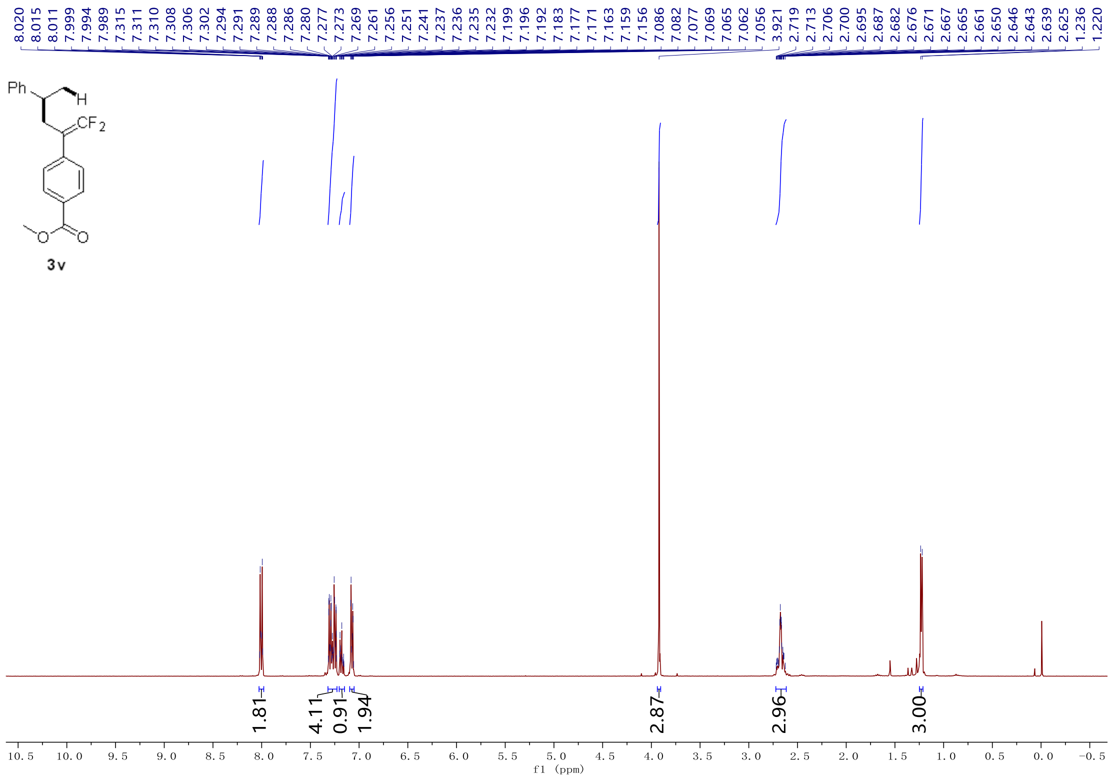

Supplementary Figure 175. ^{1}H NMR spectrum of compound **3v**

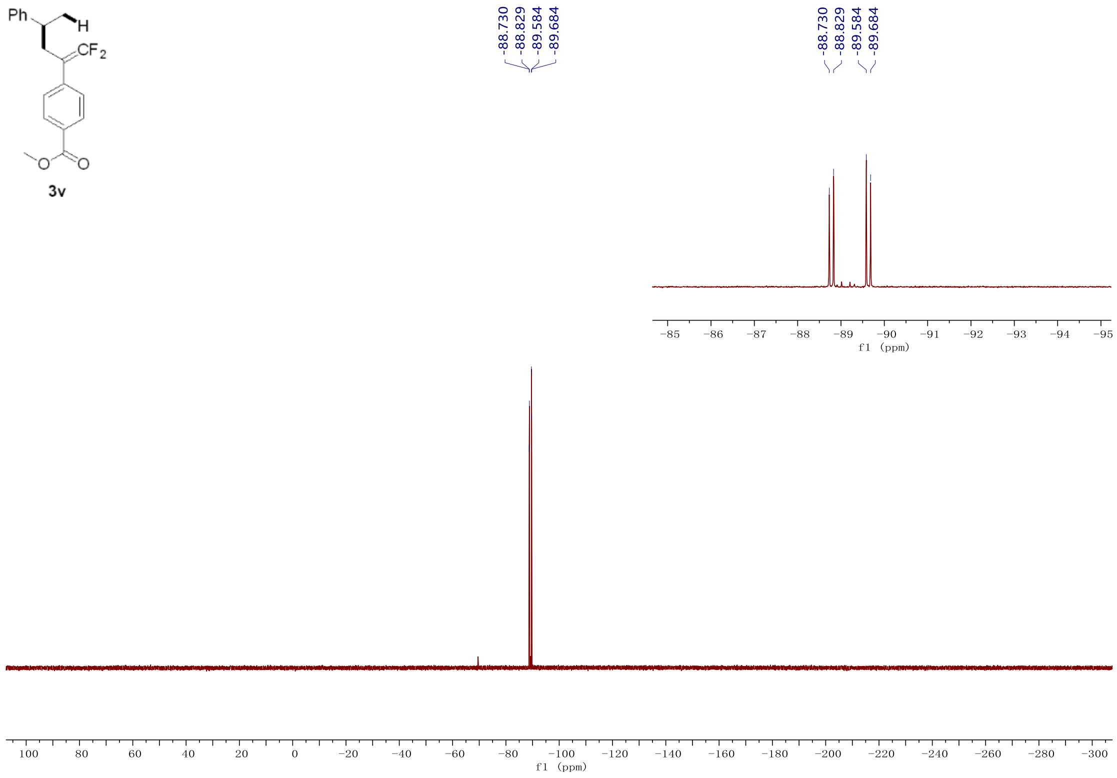

Supplementary Figure 176. ^{13}C NMR spectrum of compound **3v**

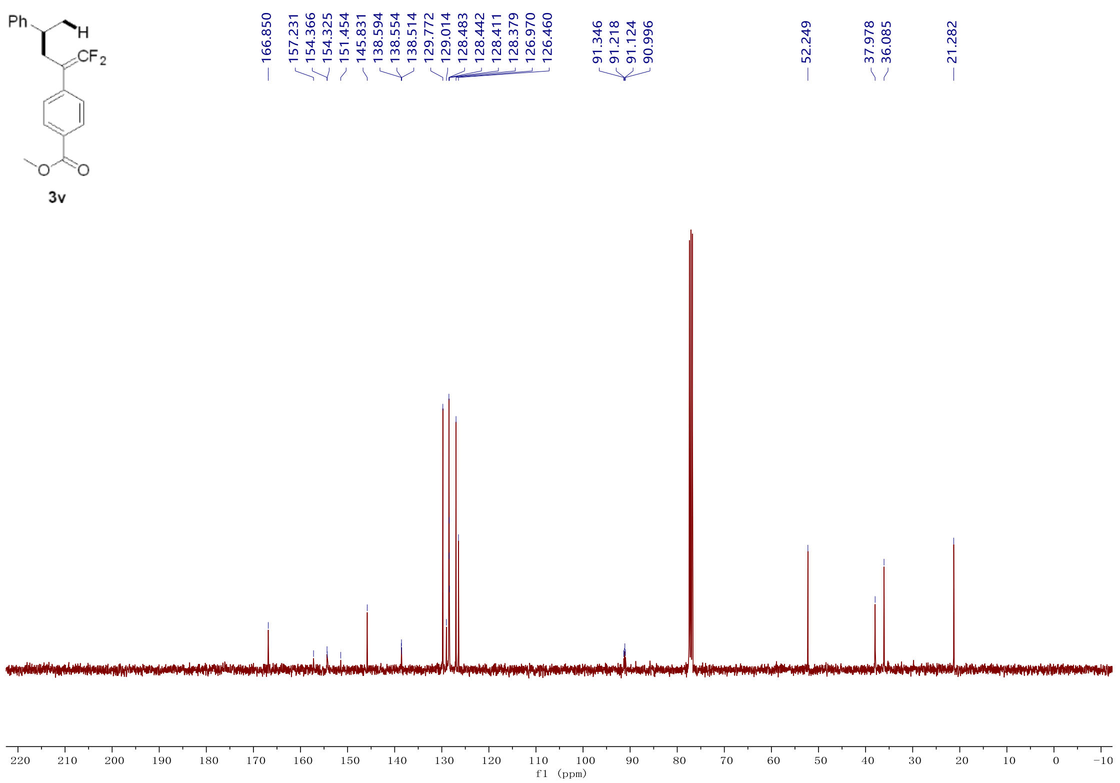

Supplementary Figure 177. ^{13}C NMR spectrum of compound **3v**

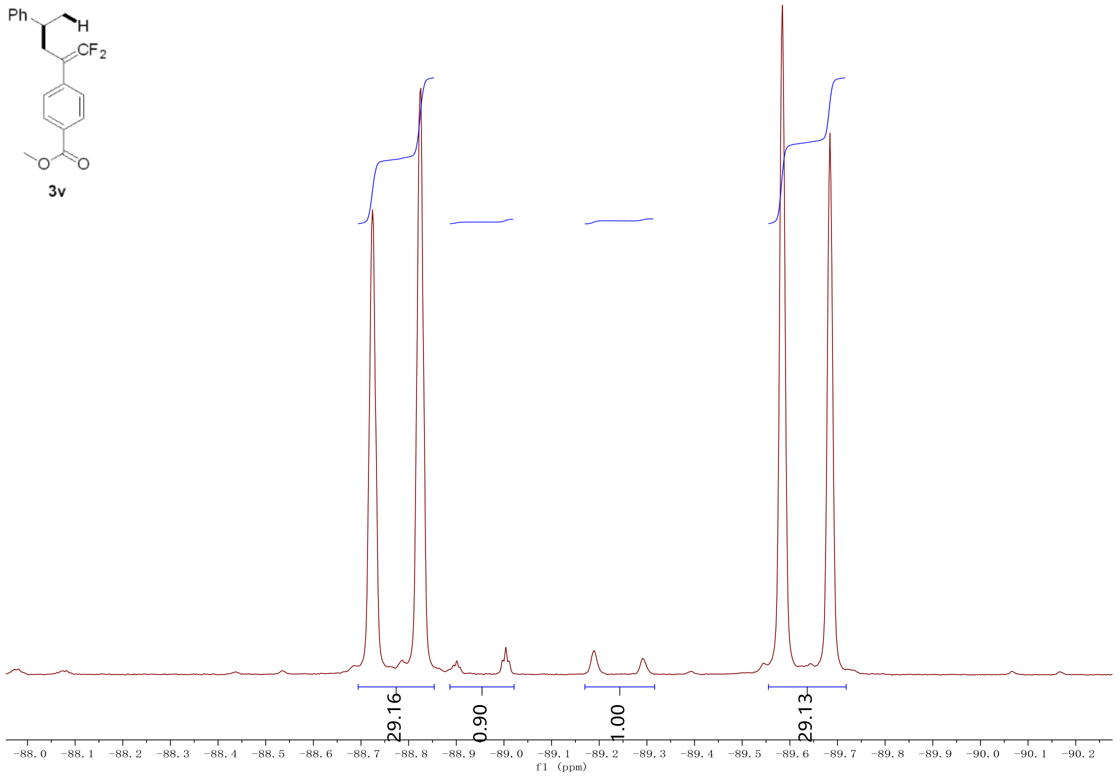

Supplementary Figure 178. Crude ^{19}F NMR spectrum of compound **3v** rr = 29 : 1

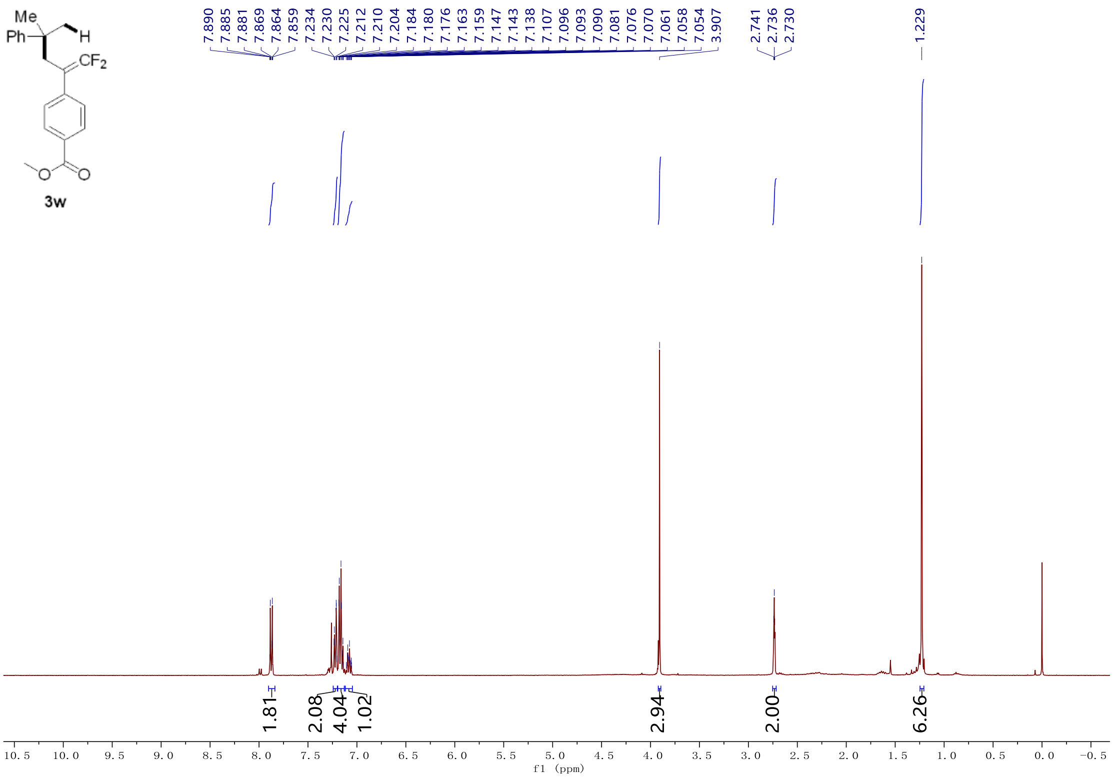

Supplementary Figure 179. ^{1}H NMR spectrum of compound **3w**

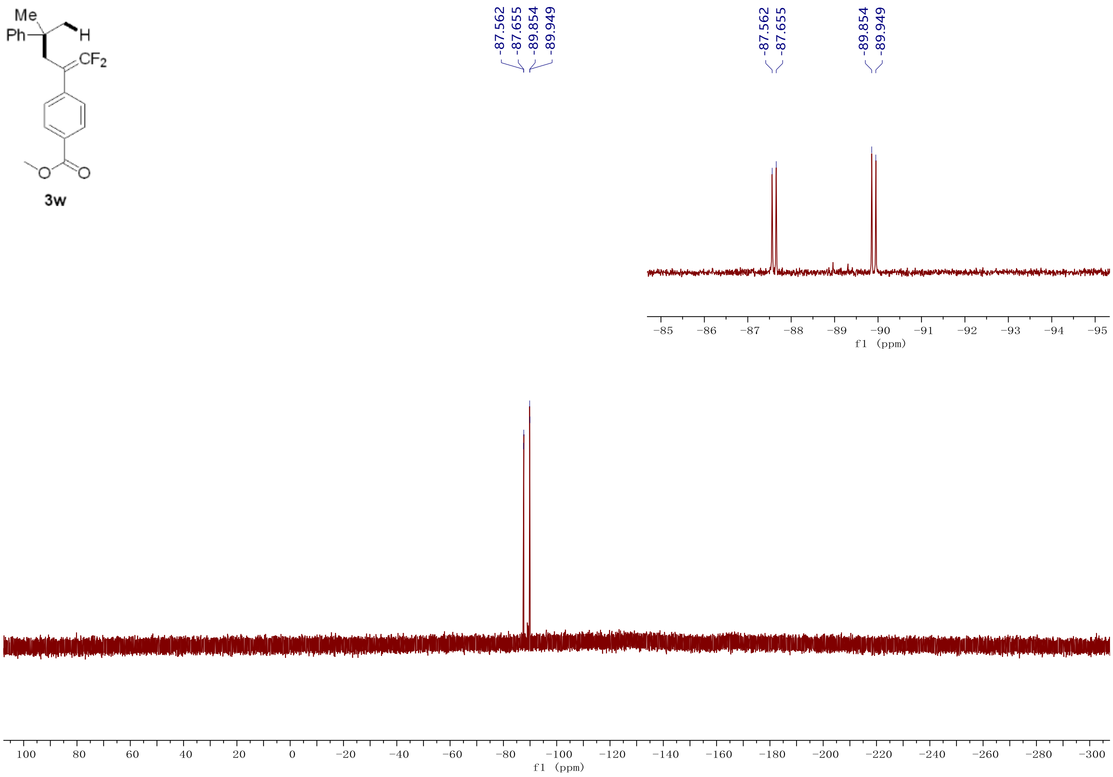

Supplementary Figure 180. ^{19}F NMR spectrum of compound **3w**

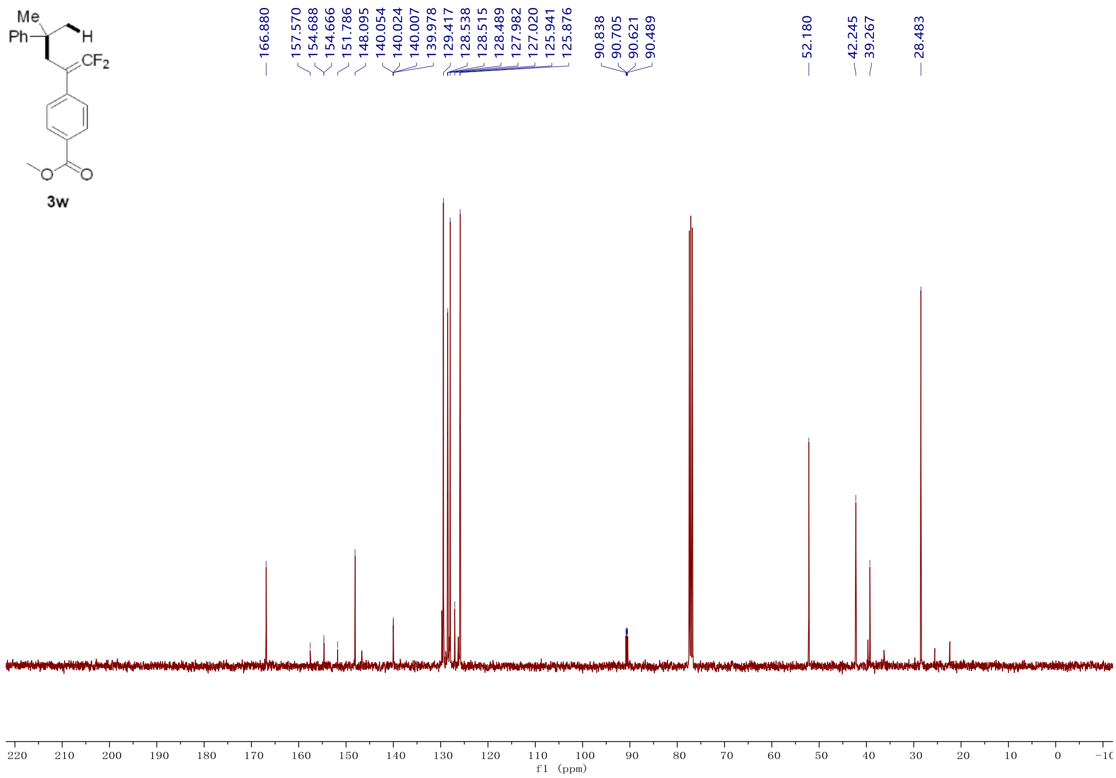

Supplementary Figure 181. ^{13}C NMR spectrum of compound **3w**

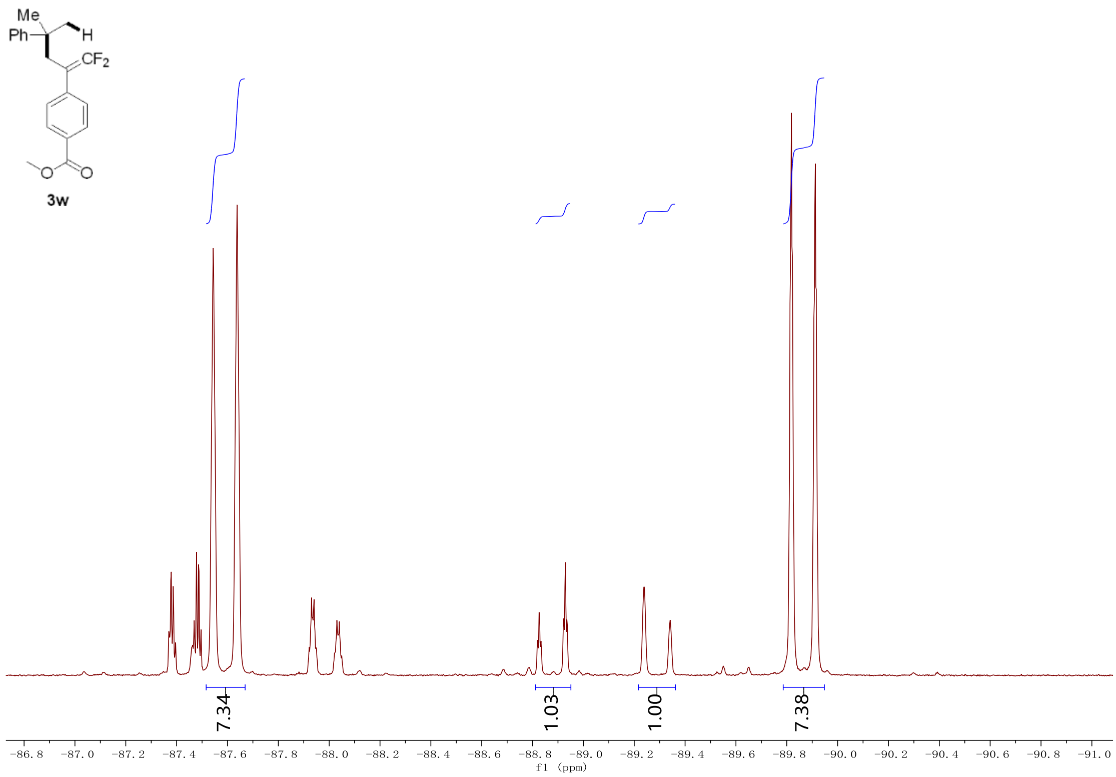

Supplementary Figure 182. Crude ^{19}F NMR spectrum of compound **3w** rr = 7 : 1

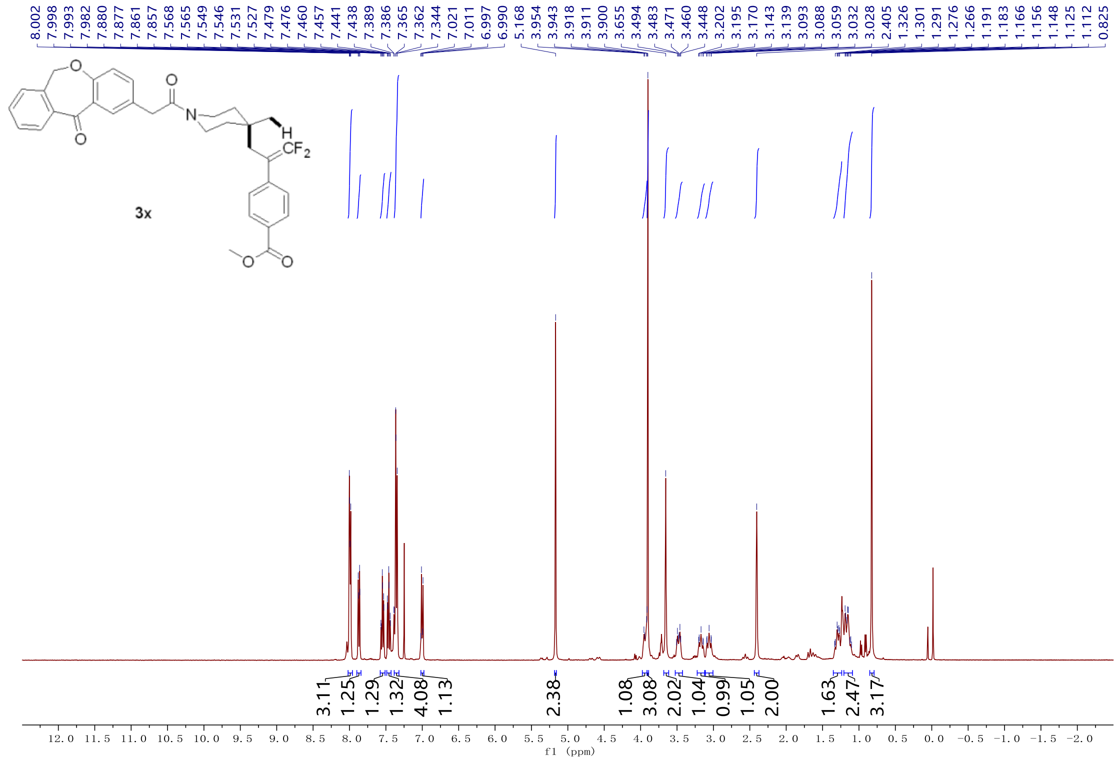

Supplementary Figure 183. ^{1}H NMR spectrum of compound **3x**

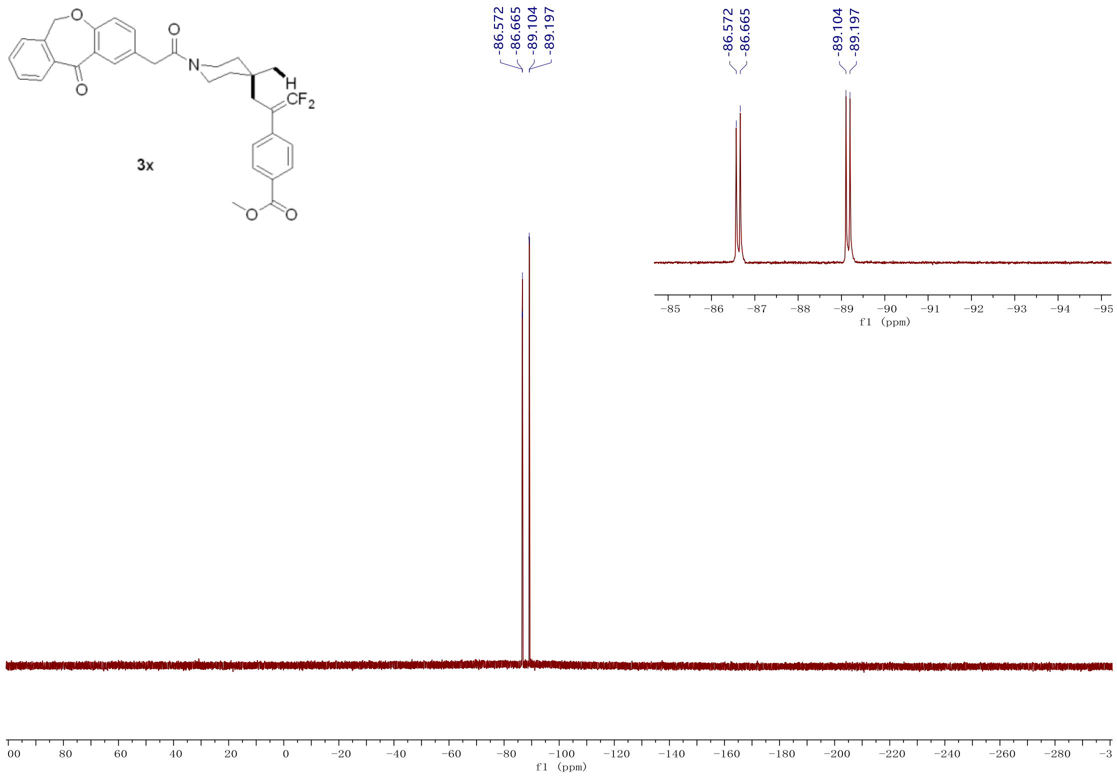

Supplementary Figure 184. ^{19}F NMR spectrum of compound **3x**

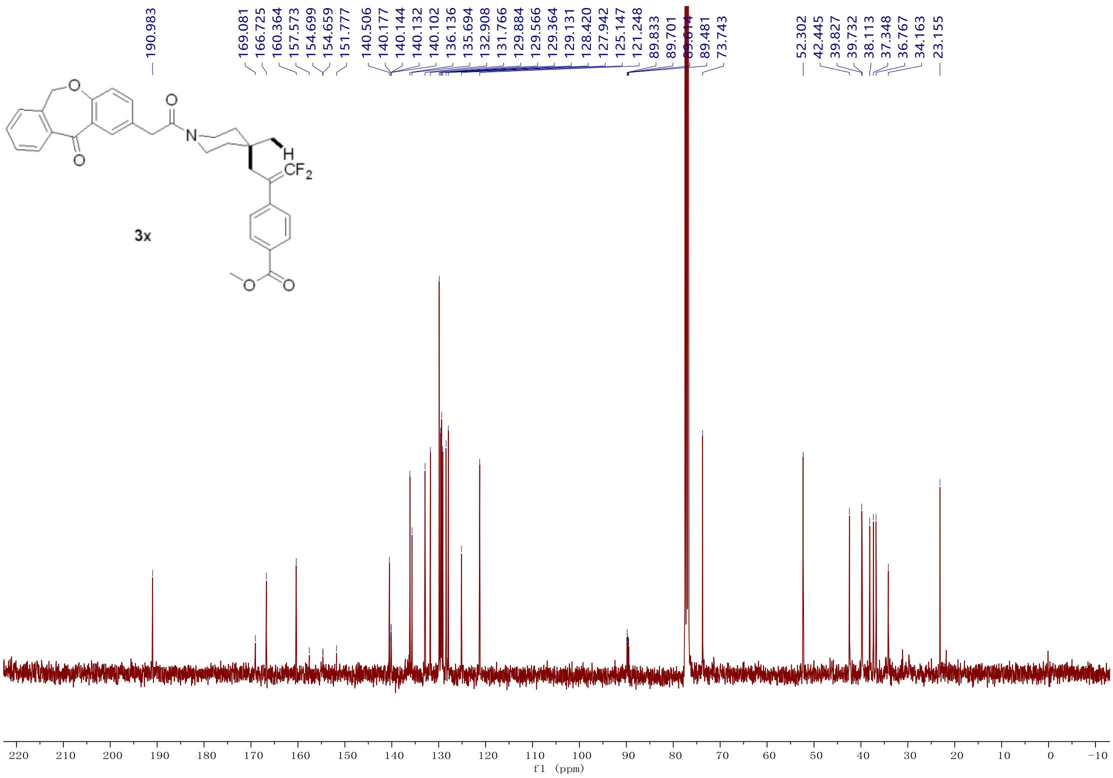

Supplementary Figure 185. ^{13}C NMR spectrum of compound **3x**

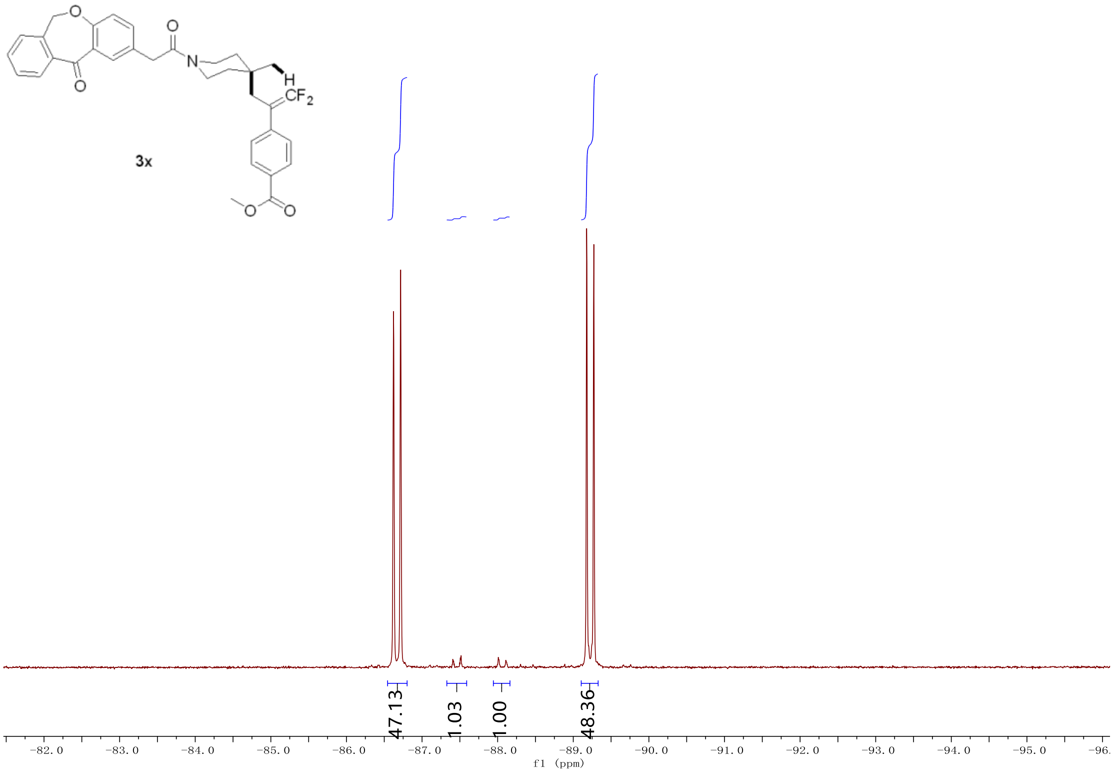

Supplementary Figure 186. Crude ^{19}F NMR spectrum of compound **3x** rr = 48 : 1

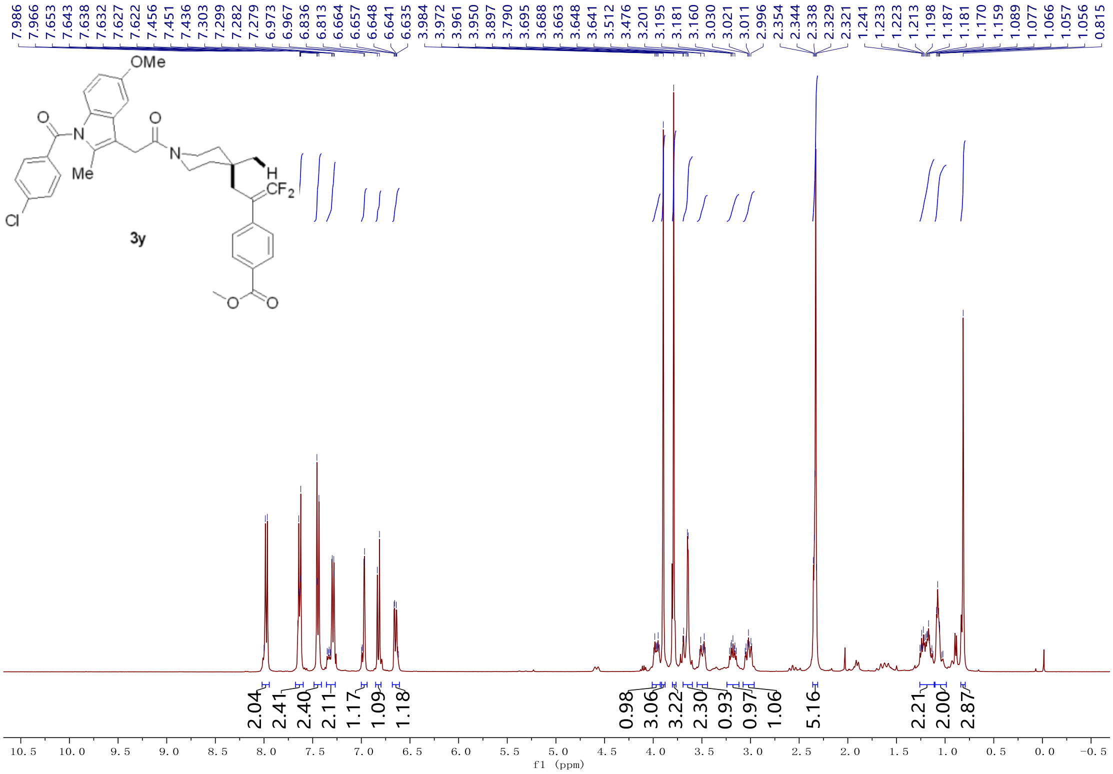

Supplementary Figure 187. ^{1}H NMR spectrum of compound **3y**

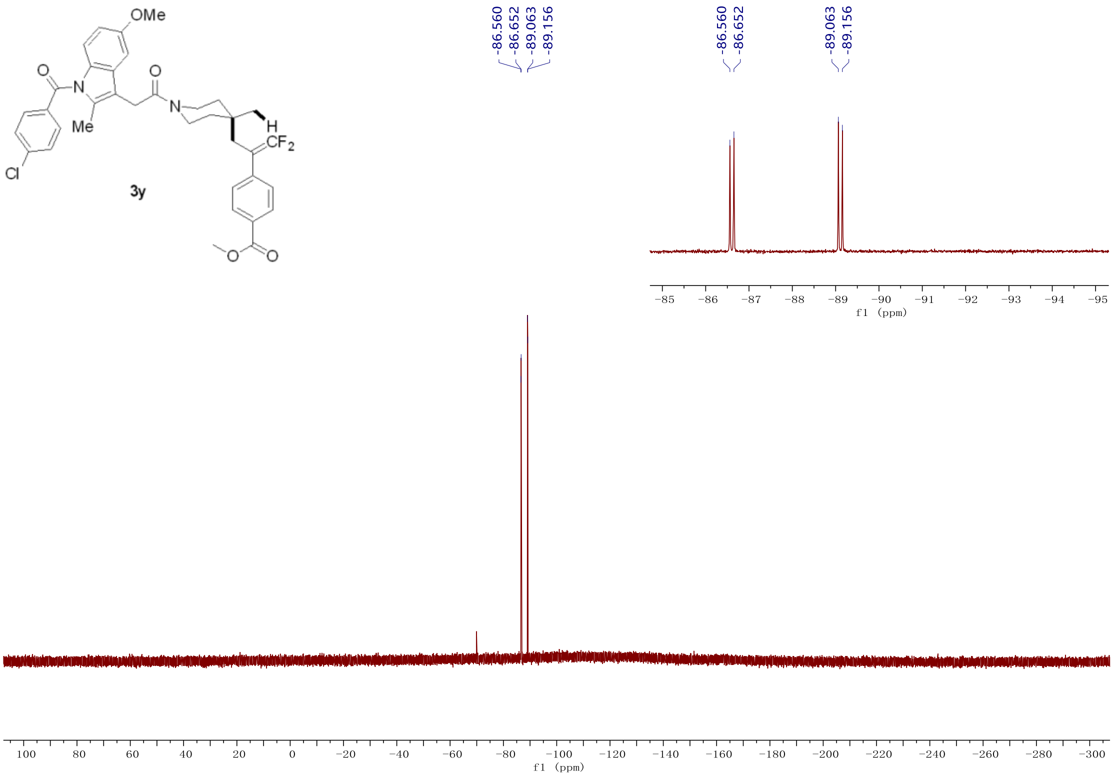

Supplementary Figure 188. ^{19}F NMR spectrum of compound **3y**

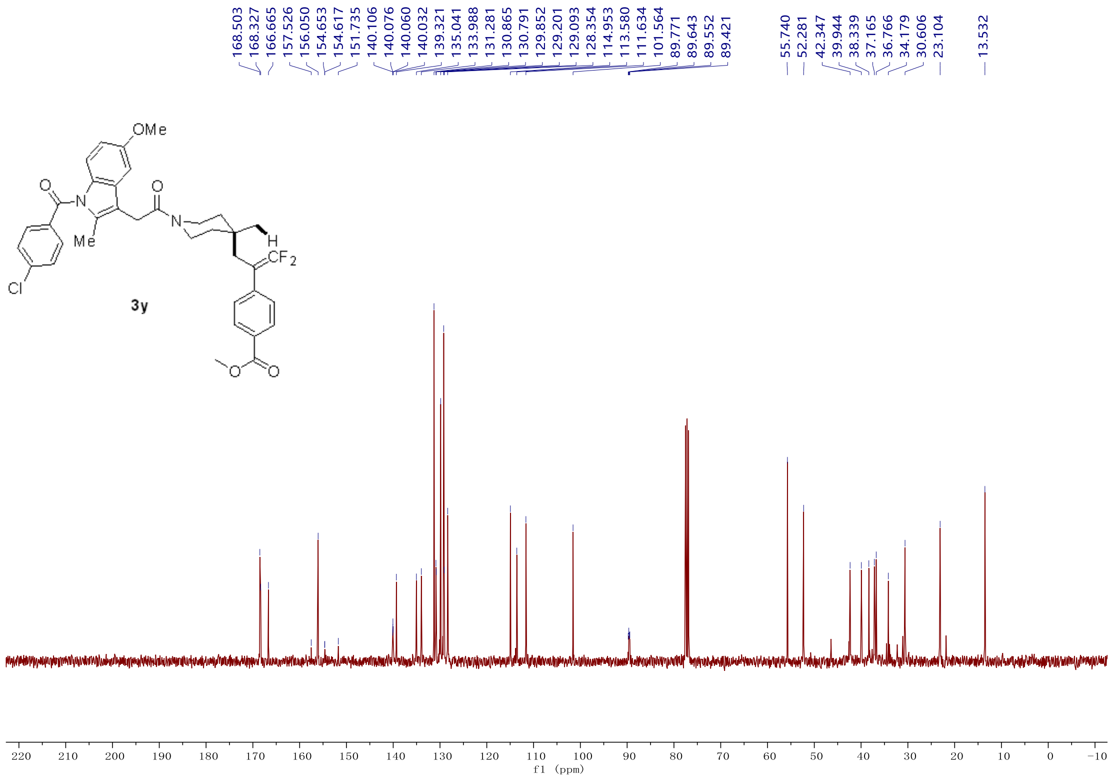

Supplementary Figure 189. ^{13}C NMR spectrum of compound **3y**

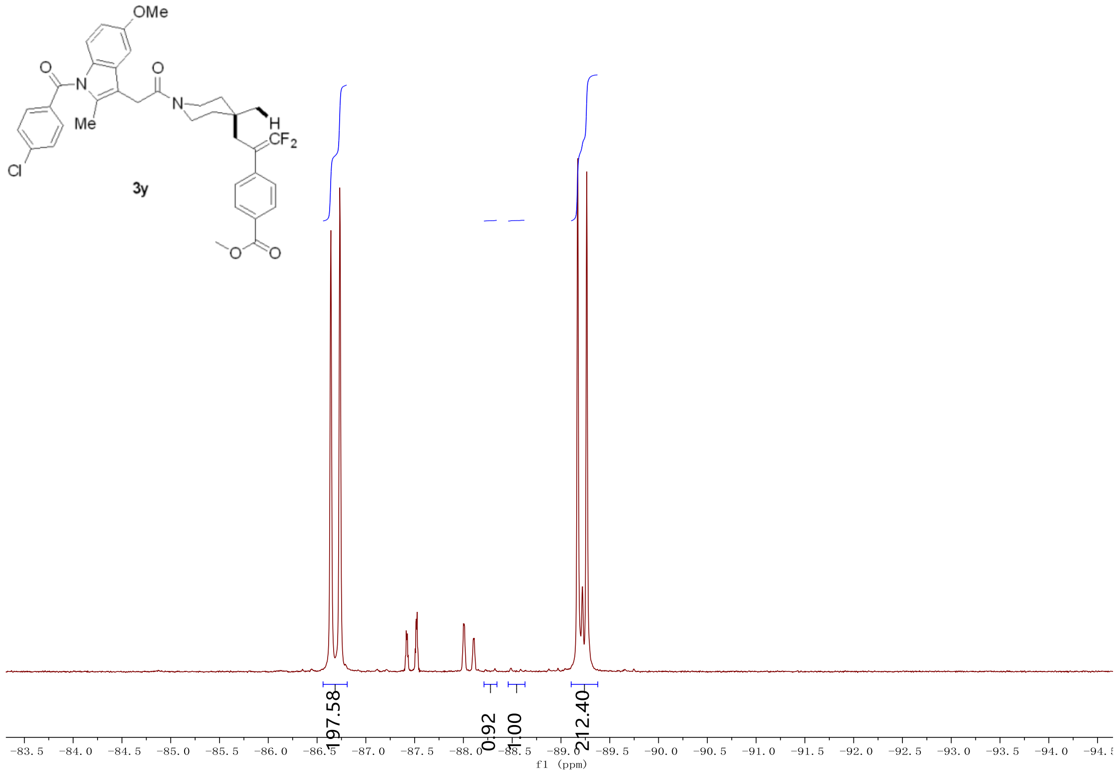

Supplementary Figure 190. Crude ^{19}F NMR spectrum of compound **3y** rr > 100 : 1

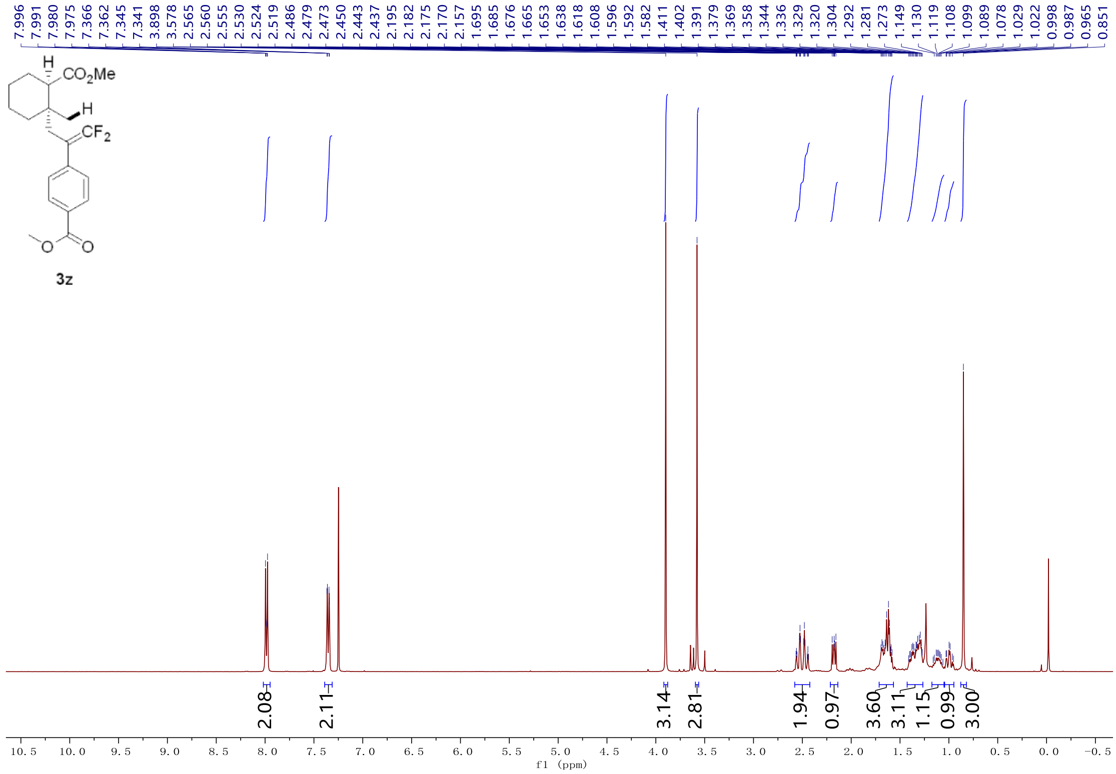

Supplementary Figure 191. ^{1}H NMR spectrum of compound **3z**

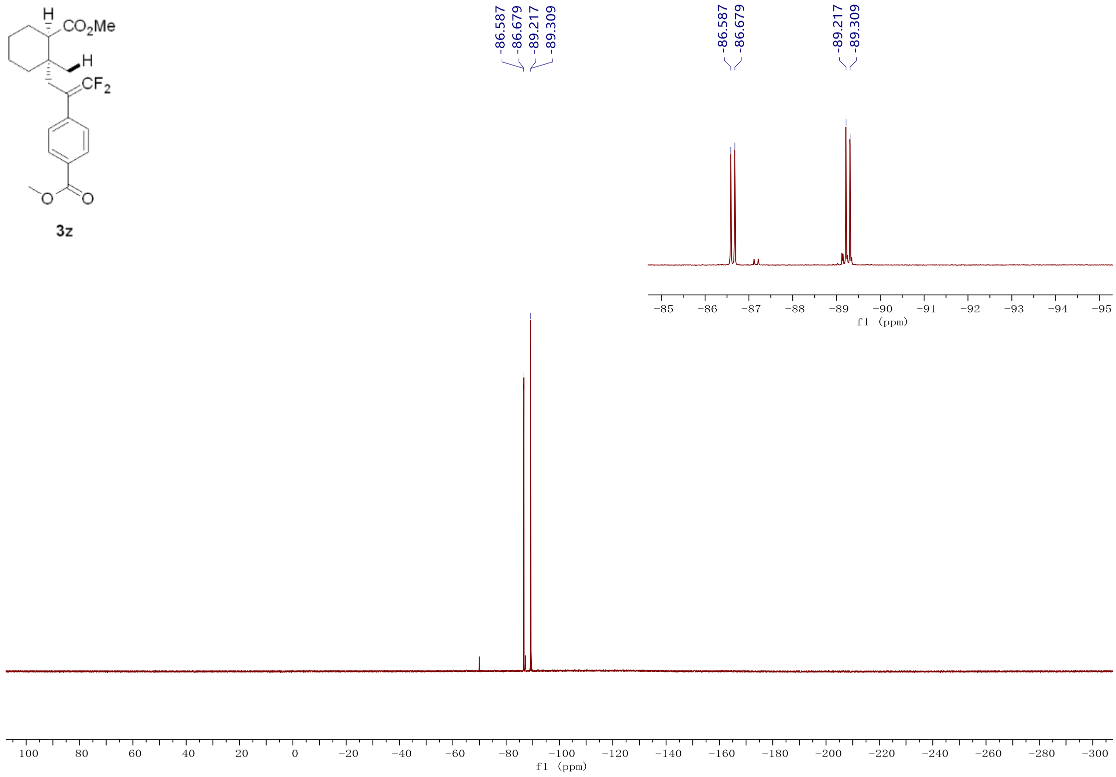

Supplementary Figure 192. ^{19}F NMR spectrum of compound **3z**

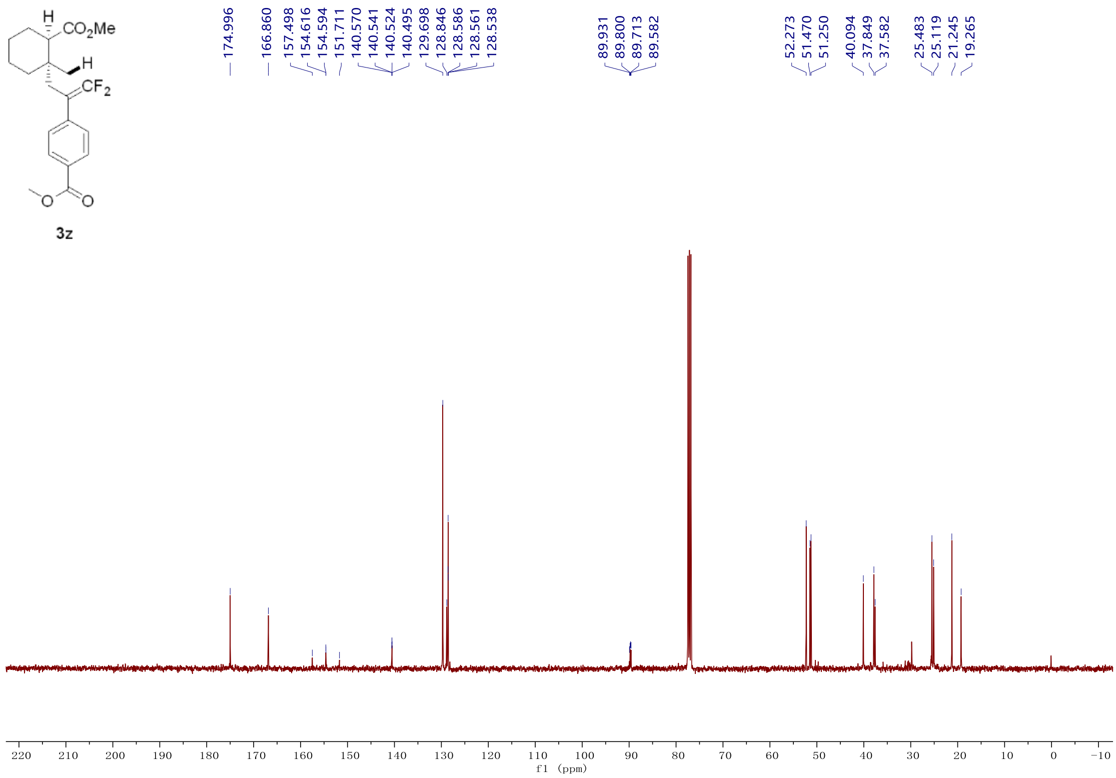

Supplementary Figure 193. ^{13}C NMR spectrum of compound **3z**

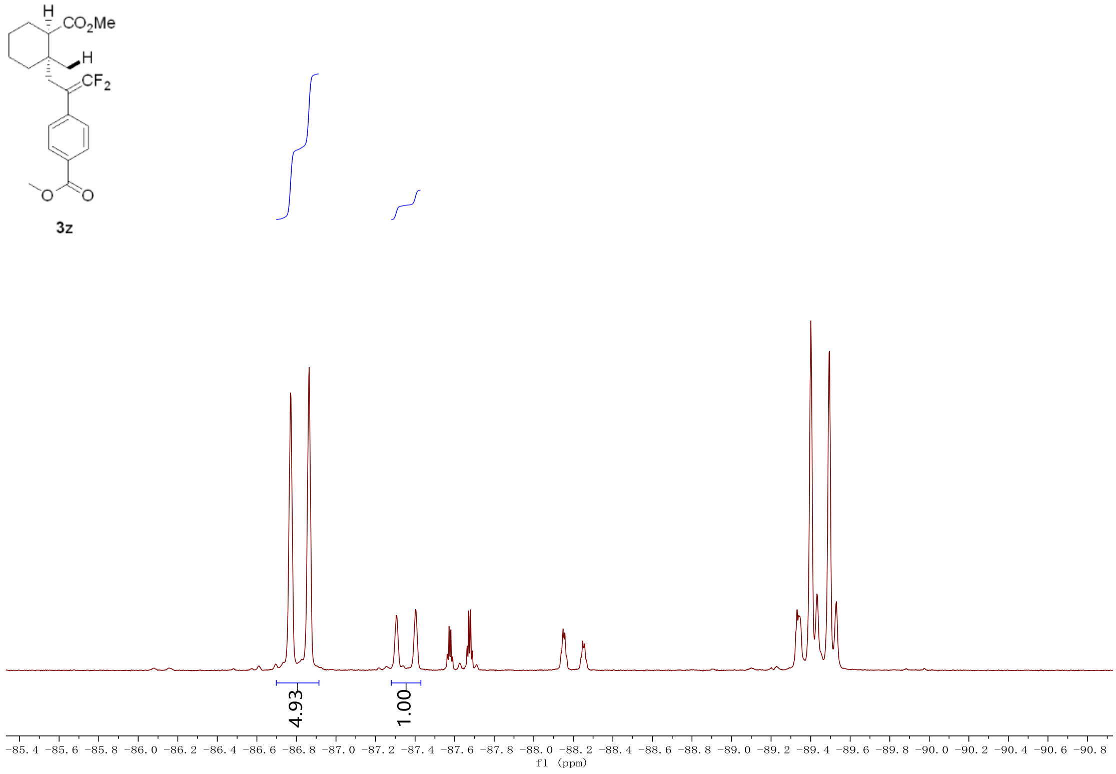

Supplementary Figure 194. Crude ^{19}F NMR spectrum of compound **3z** rr > 20 : 1, dr = 5 : 1

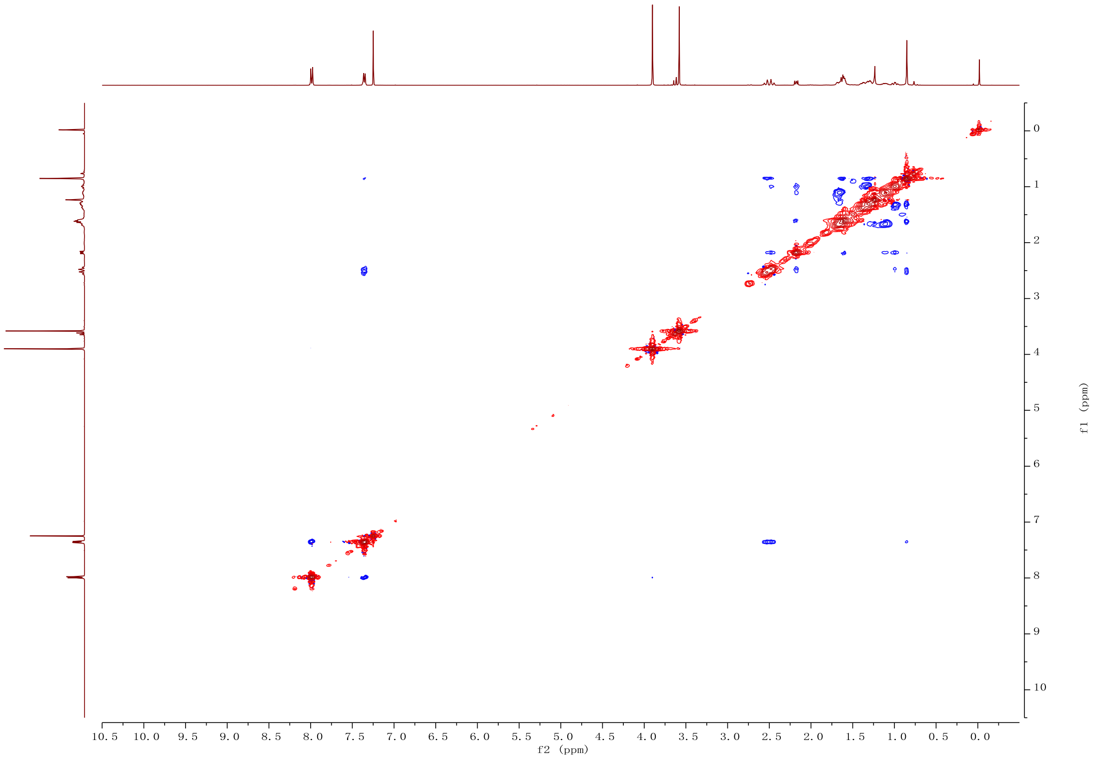

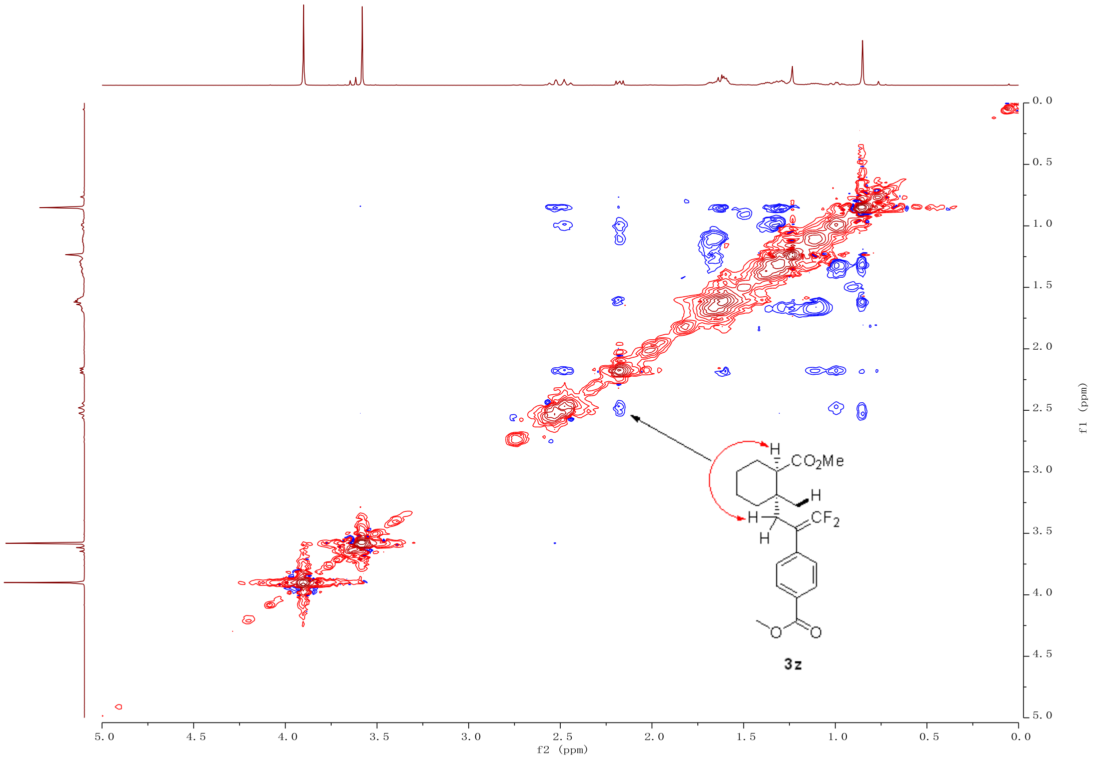

Supplementary Figure 195. NOESY spectrum of compound **3z**

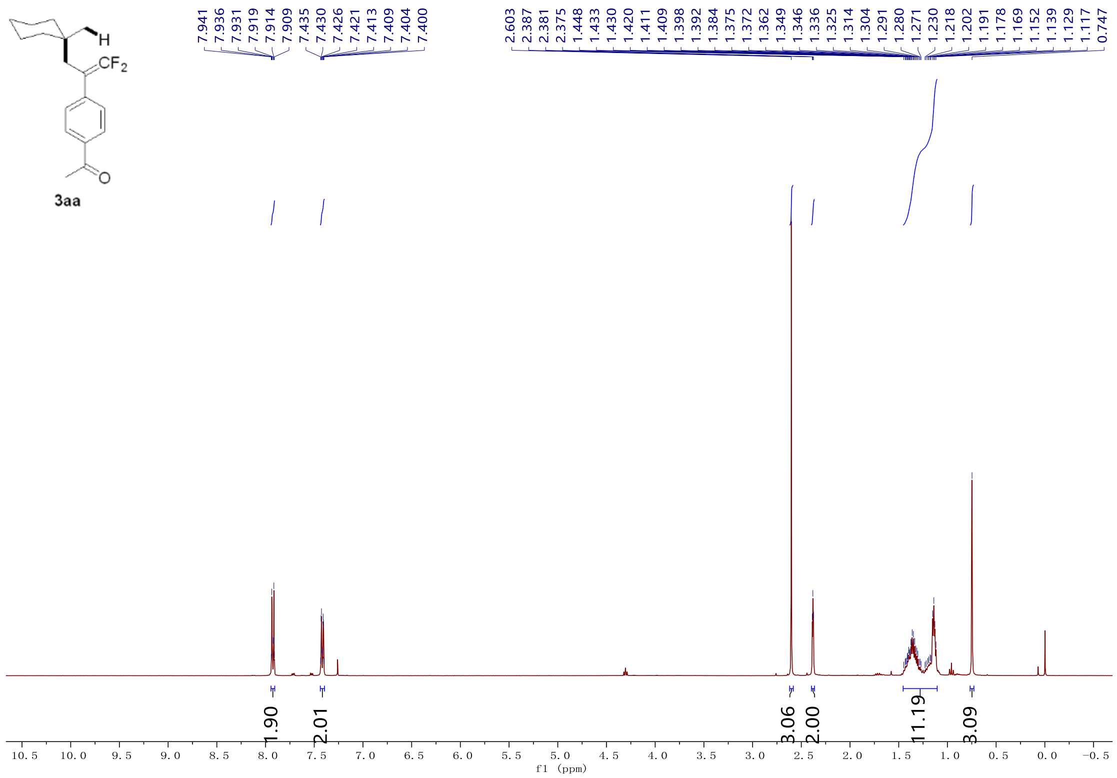

Supplementary Figure 196. ^{1}H NMR spectrum of compound **3aa**

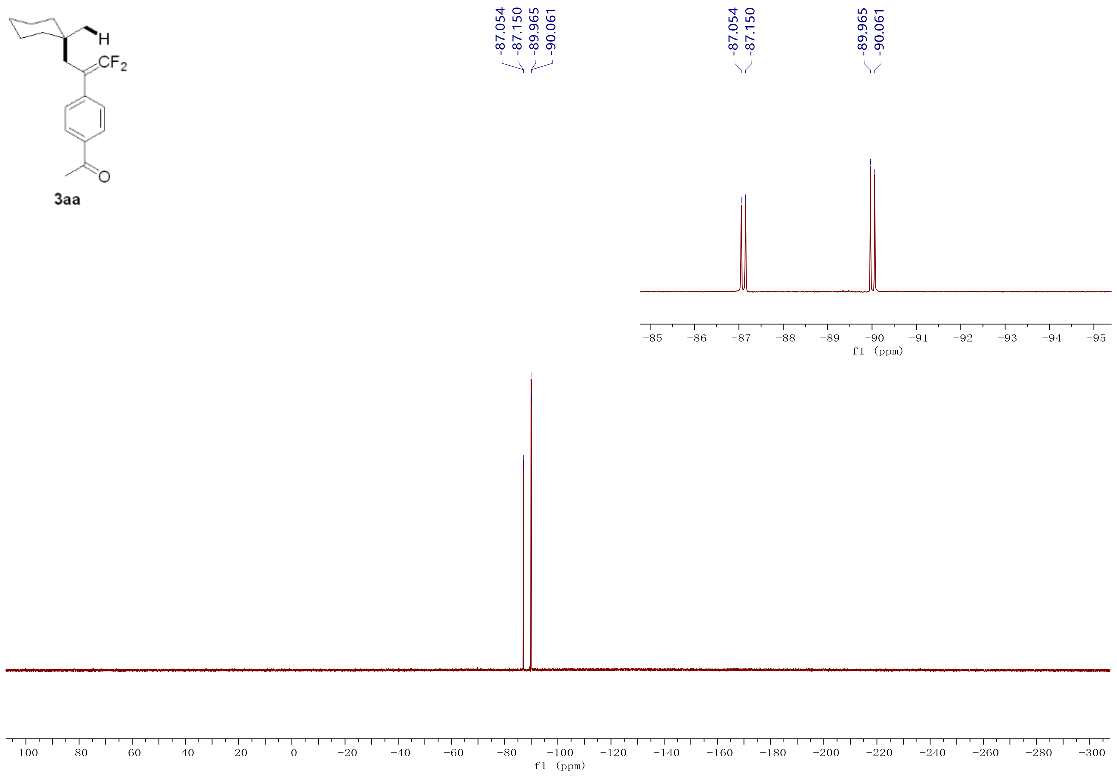

Supplementary Figure 197. ^{19}F NMR spectrum of compound **3aa**

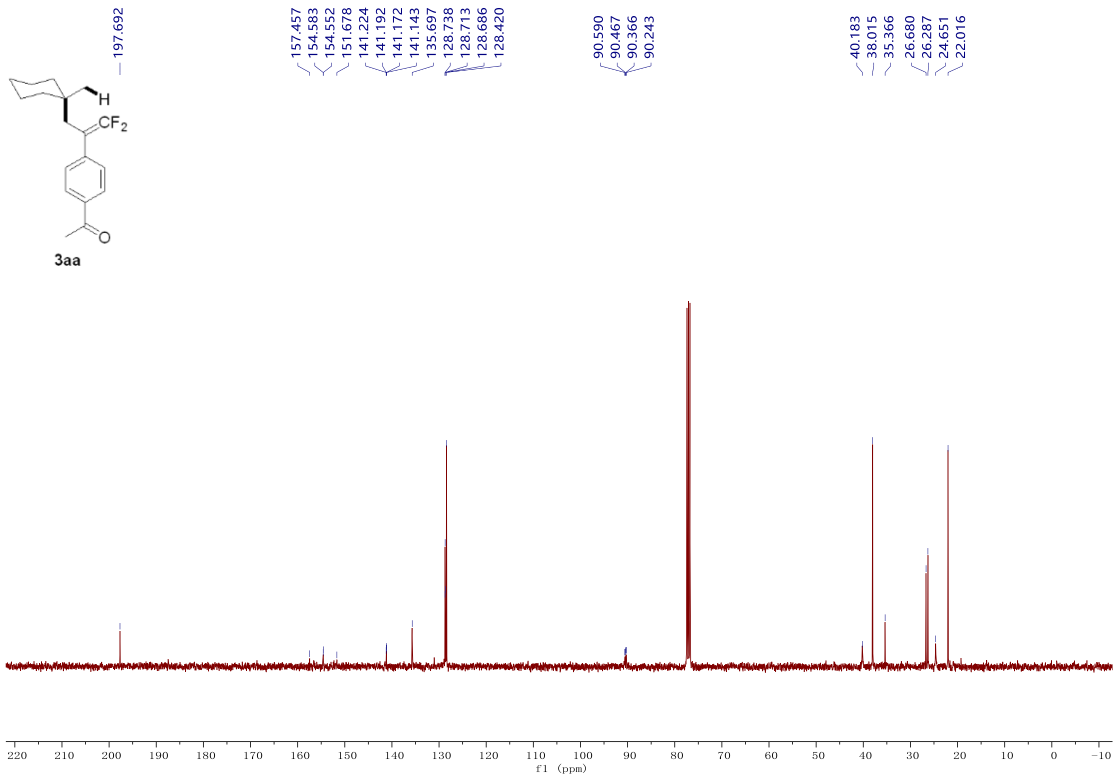

Supplementary Figure 198. ^{13}C NMR spectrum of compound **3aa**

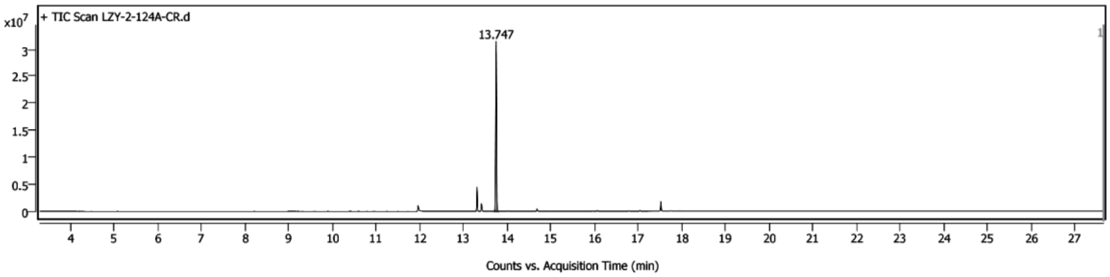

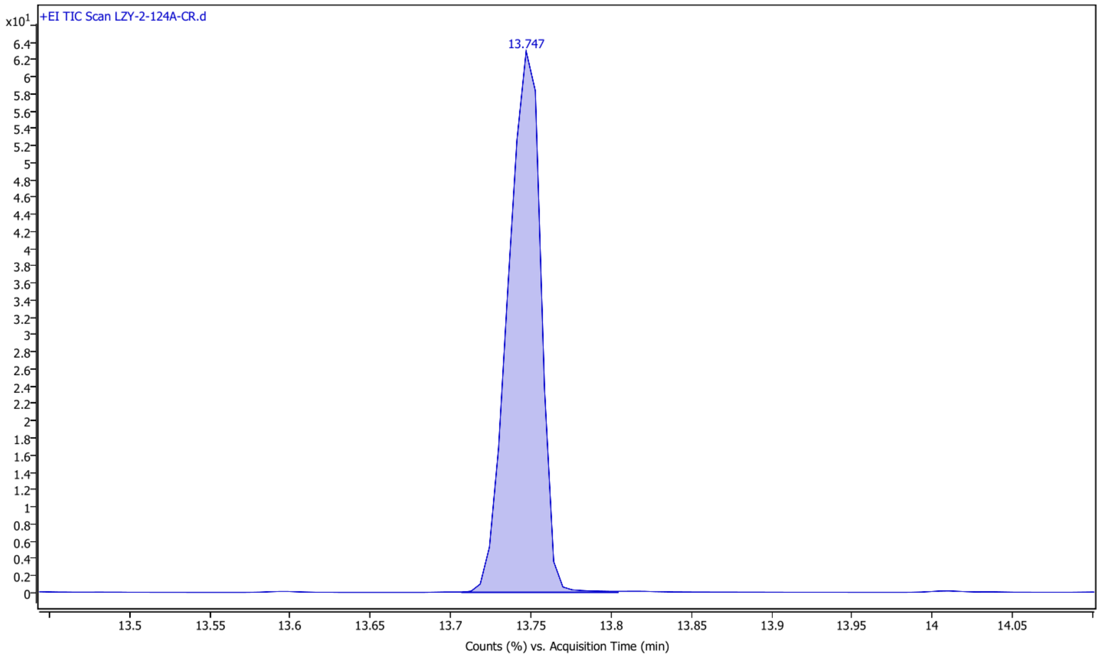

Chromatogram Peaks

Peak	Start	RT	End	Height	Area	Area %	SNR
1	13.707	13.747	13.804	31472908	44838254	100.00	

Supplementary Figure 199. GC spectrum of crude products of **3aa** rr > 20 : 1

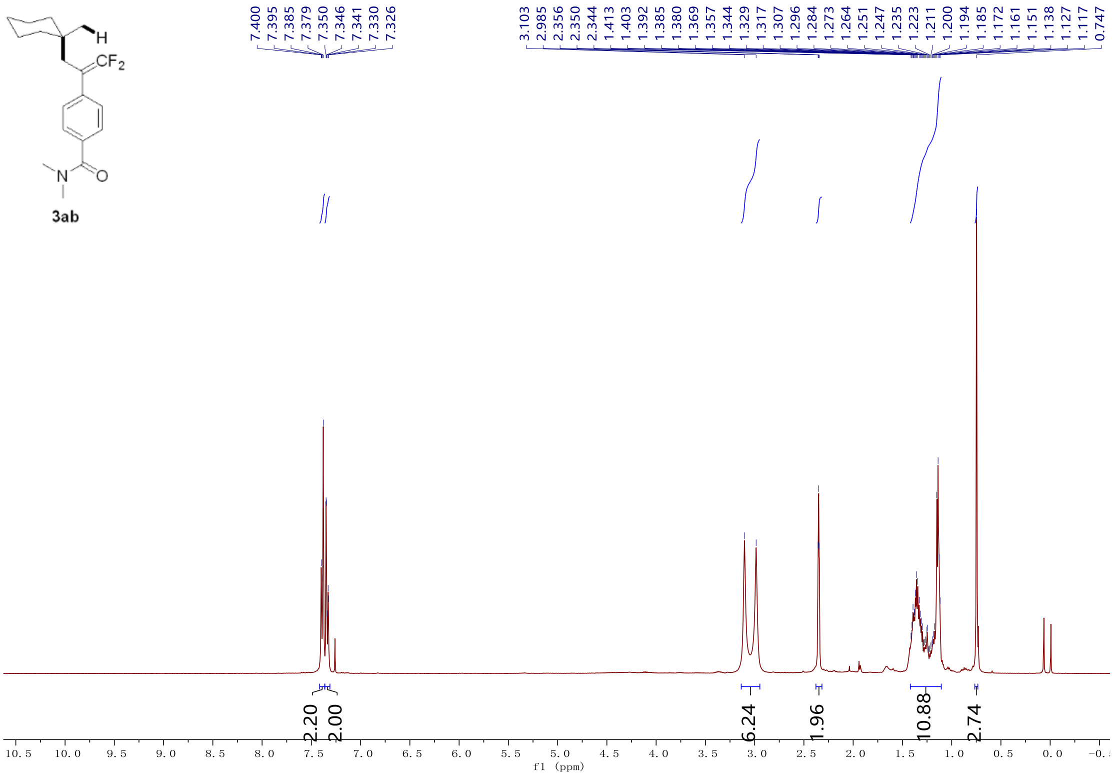

Supplementary Figure 200. ^{1}H NMR spectrum of compound **3ab**

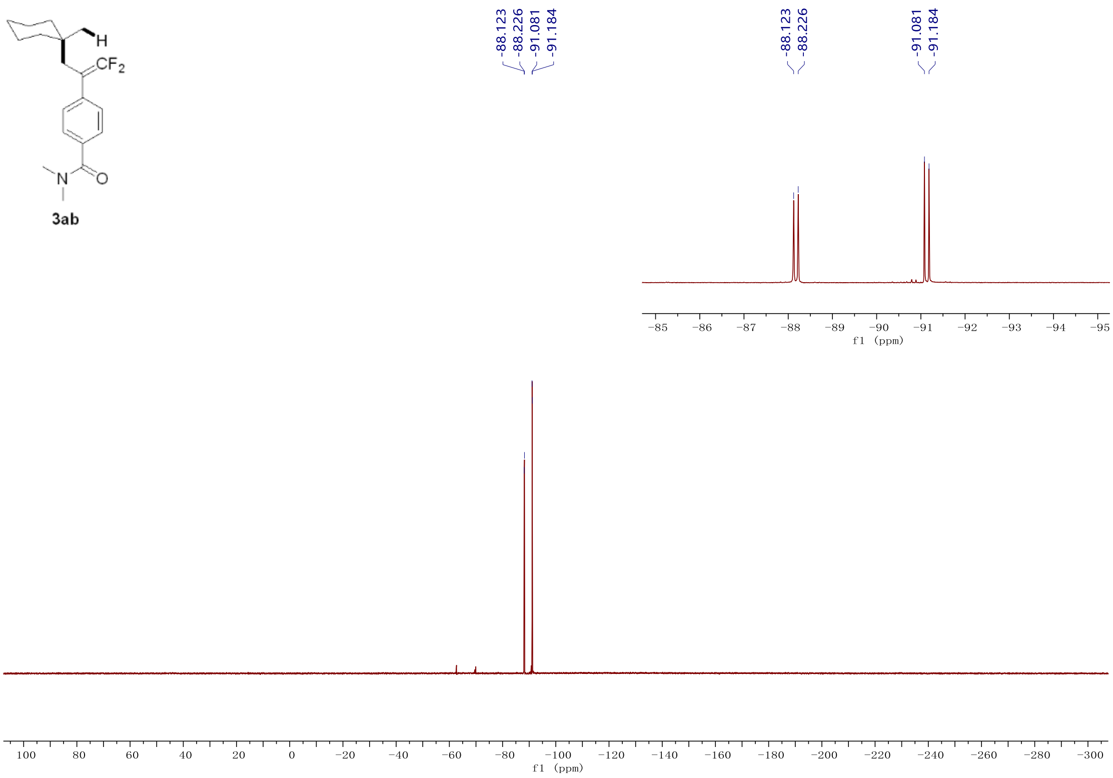

Supplementary Figure 201. ^{19}F NMR spectrum of compound **3ab**

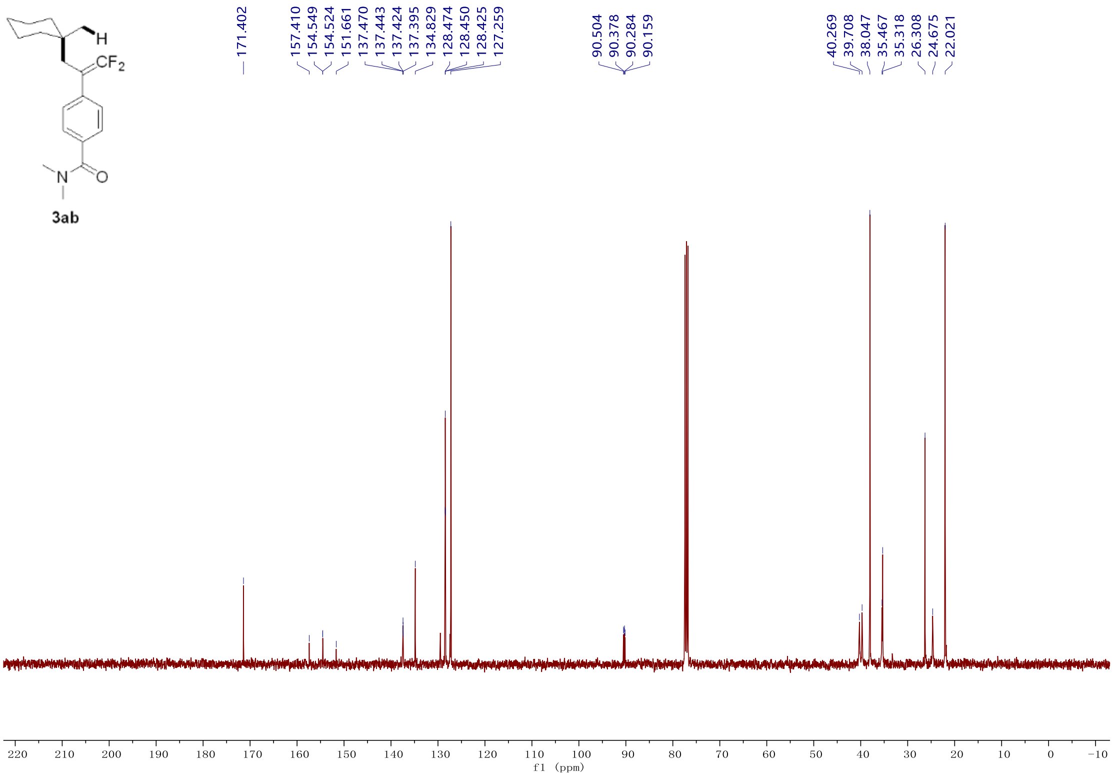

Supplementary Figure 202. ^{13}C NMR spectrum of compound **3ab**

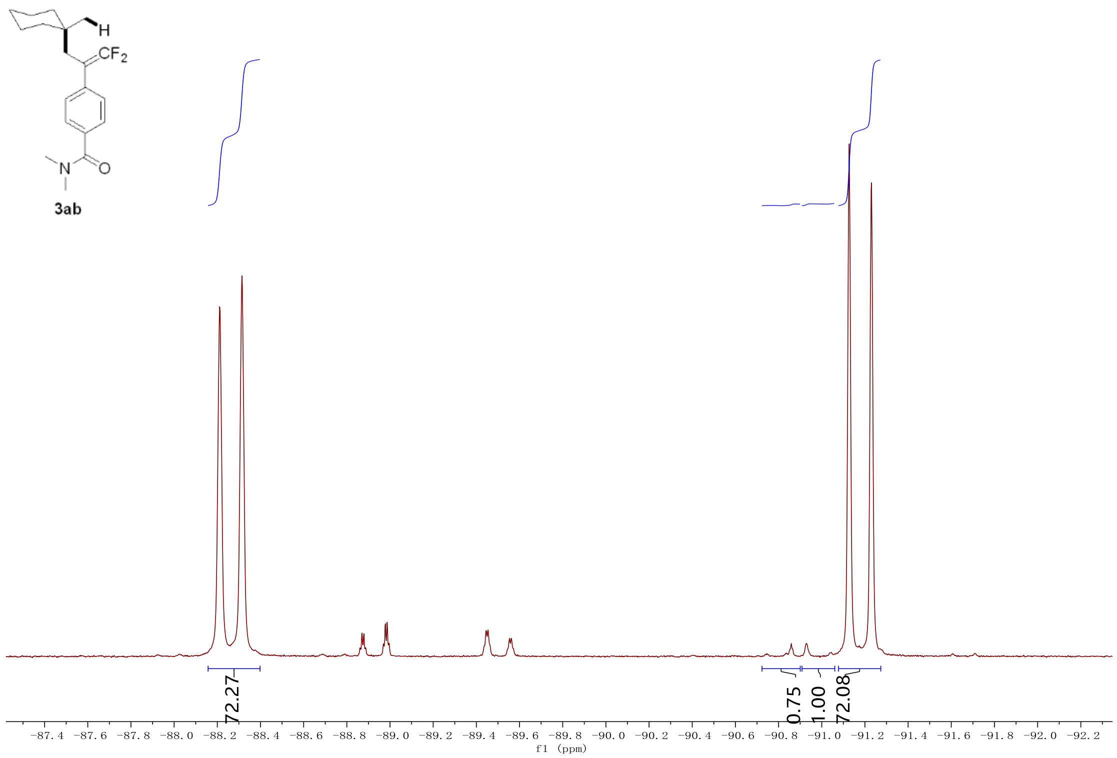

Supplementary Figure 203. Crude ^{19}F NMR spectrum of compound **3ab** rr = 72 : 1

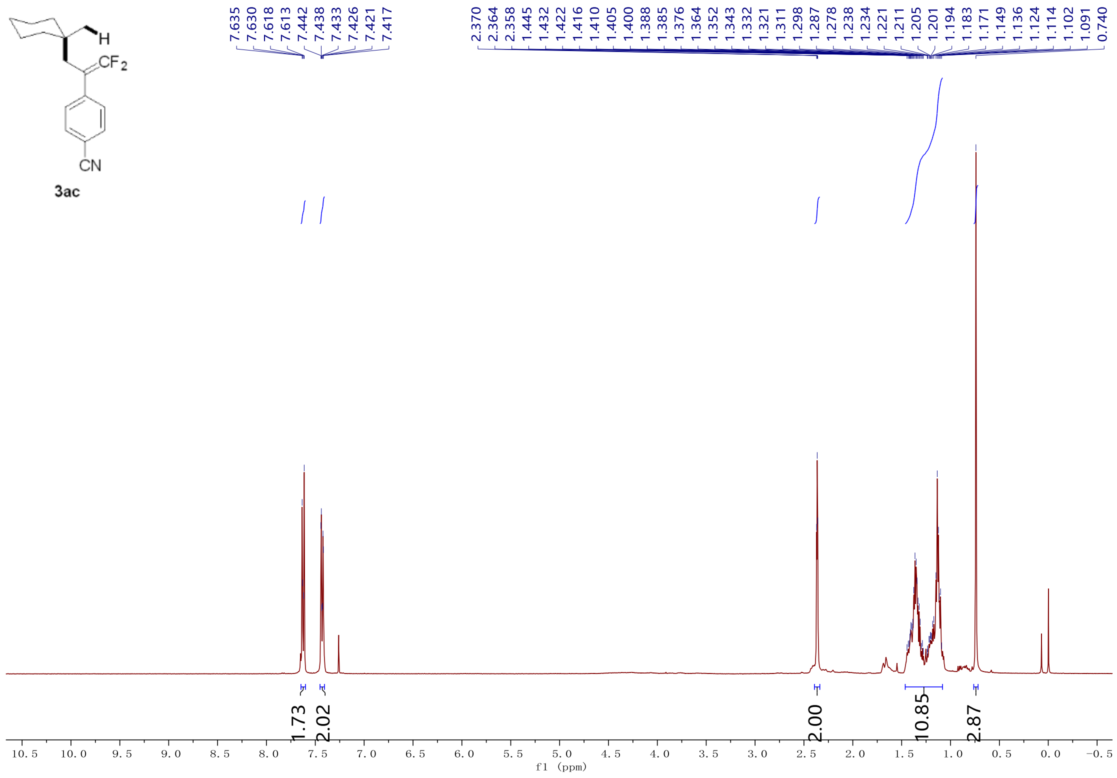

Supplementary Figure 204. ^{1}H NMR spectrum of compound **3ac**

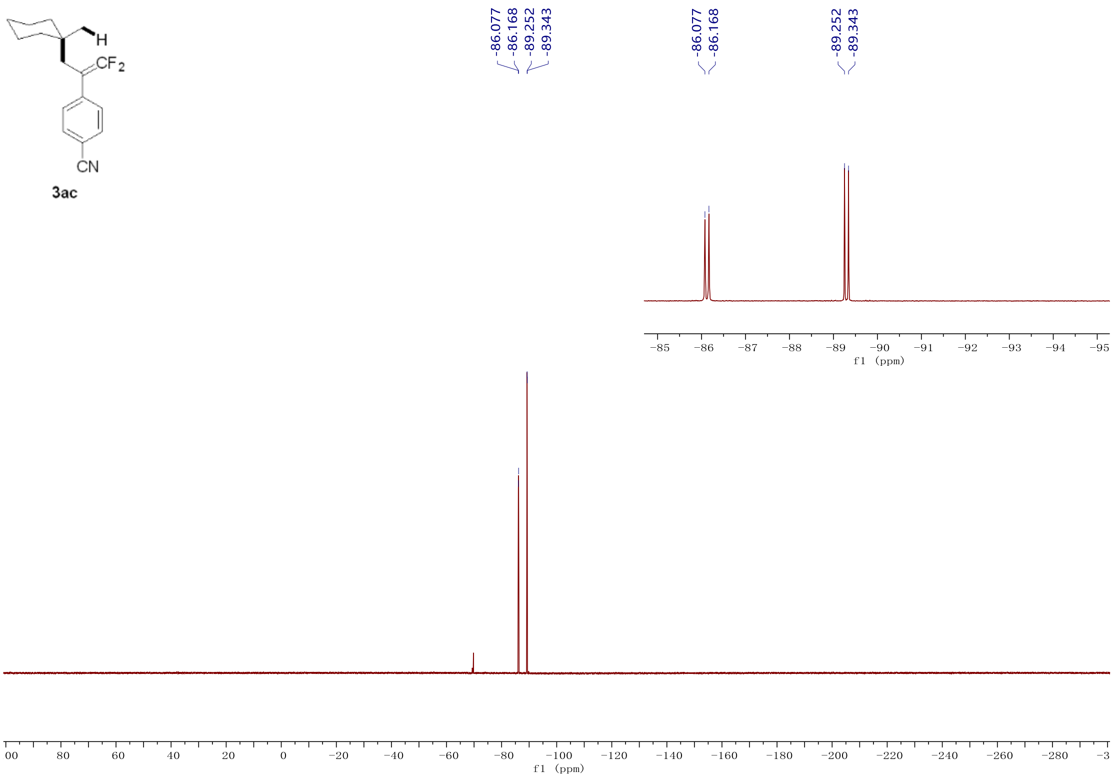

Supplementary Figure 205. ^{19}F NMR spectrum of compound **3ac**

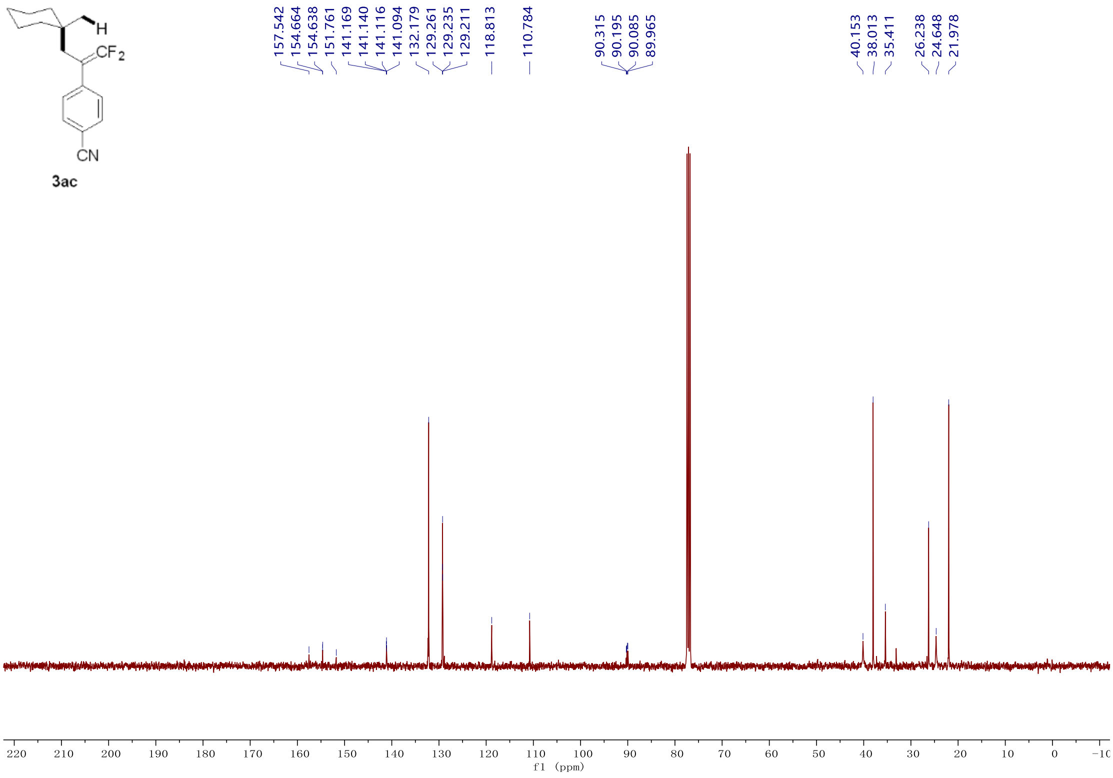

Supplementary Figure 206. ^{13}C NMR spectrum of compound **3ac**

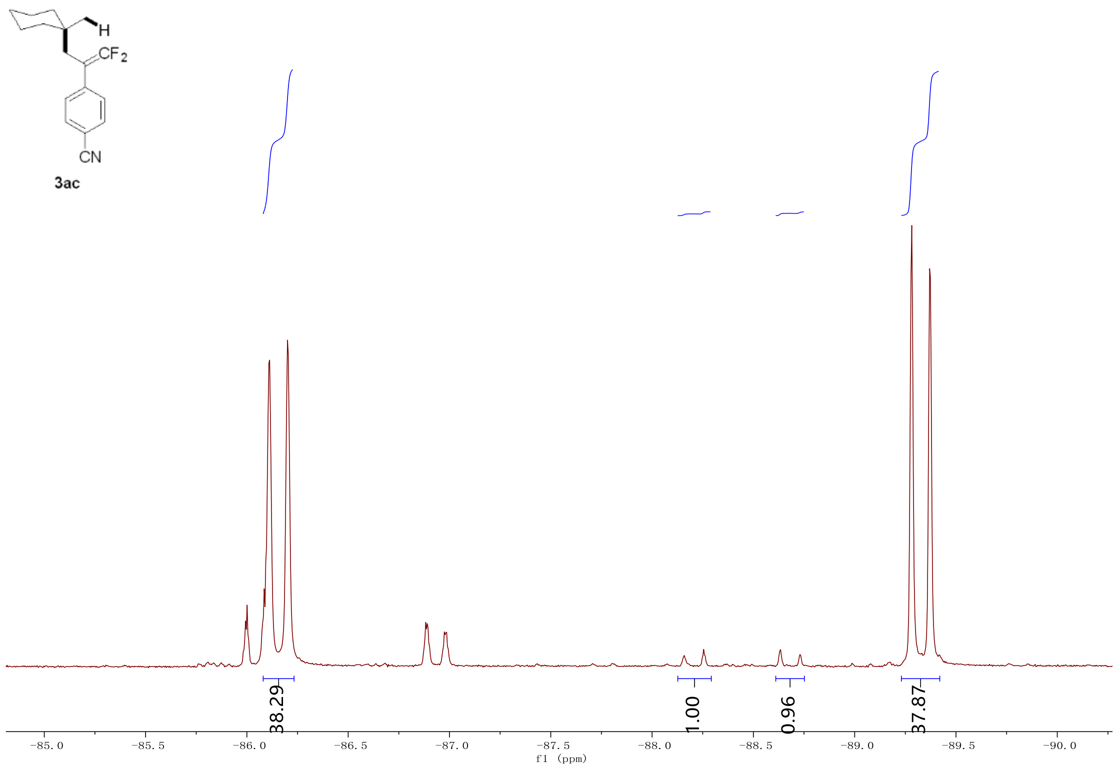

Supplementary Figure 207. Crude ^{19}F NMR spectrum of compound **3ac** rr = 38 : 1

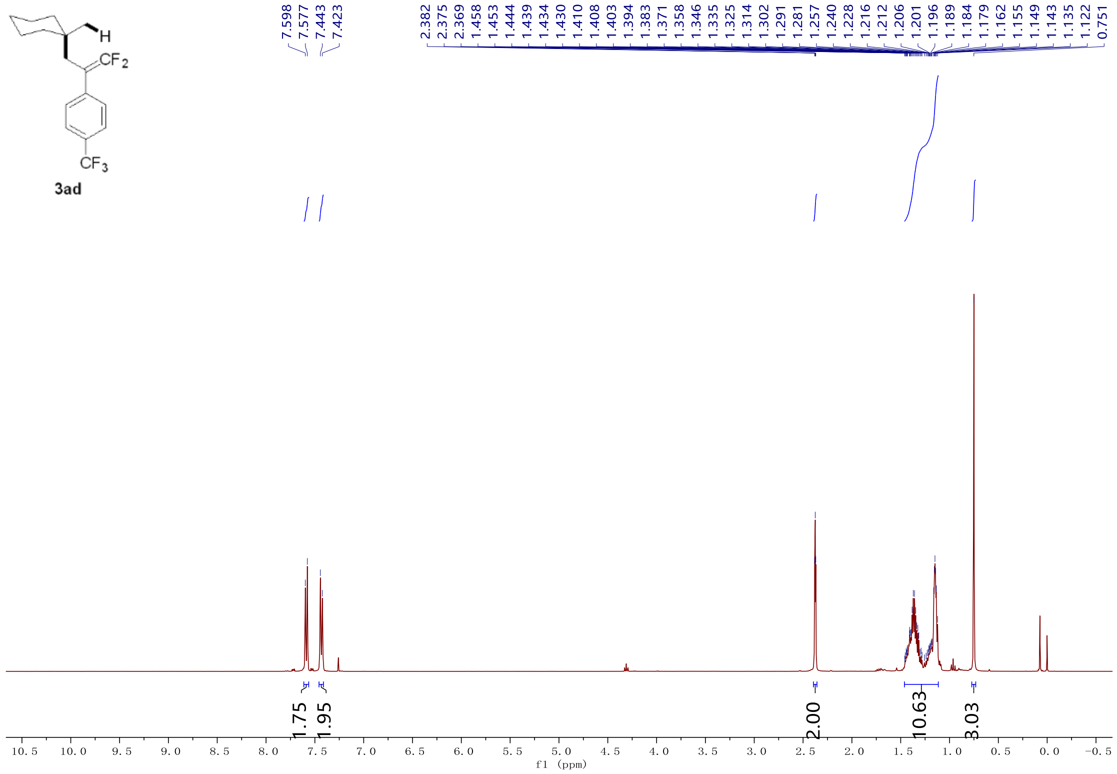

Supplementary Figure 208. ^{1}H NMR spectrum of compound **3ad**

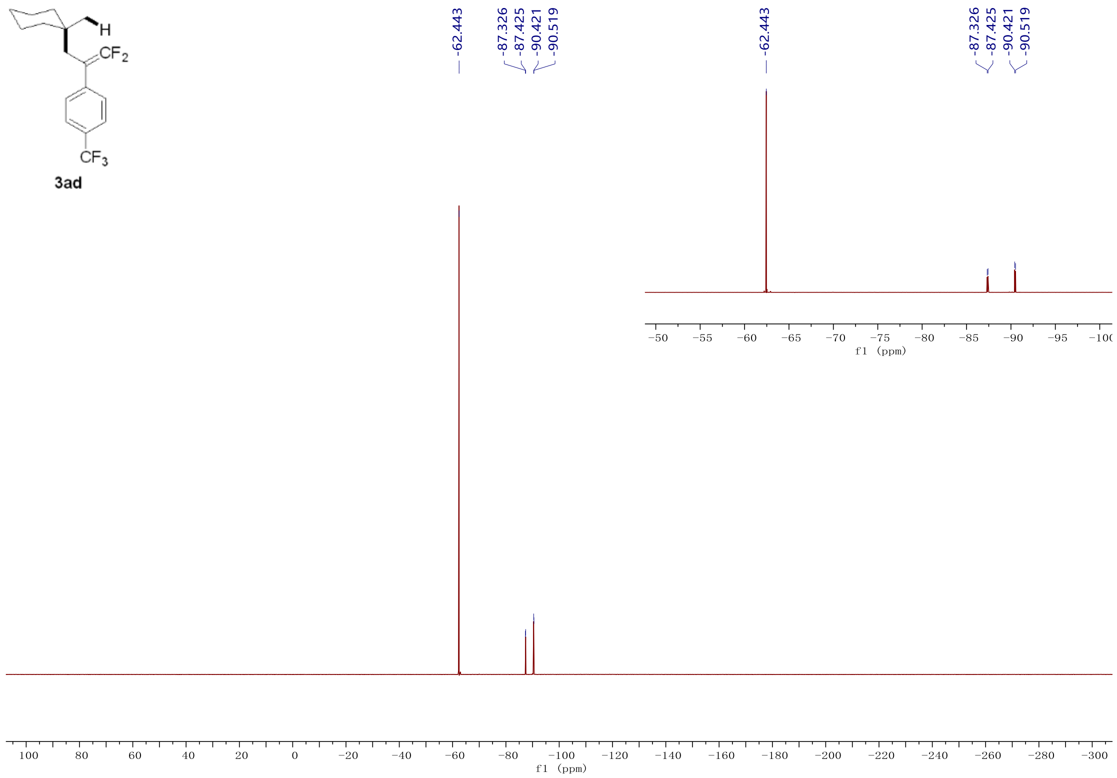

Supplementary Figure 209. ^{19}F NMR spectrum of compound **3ad**

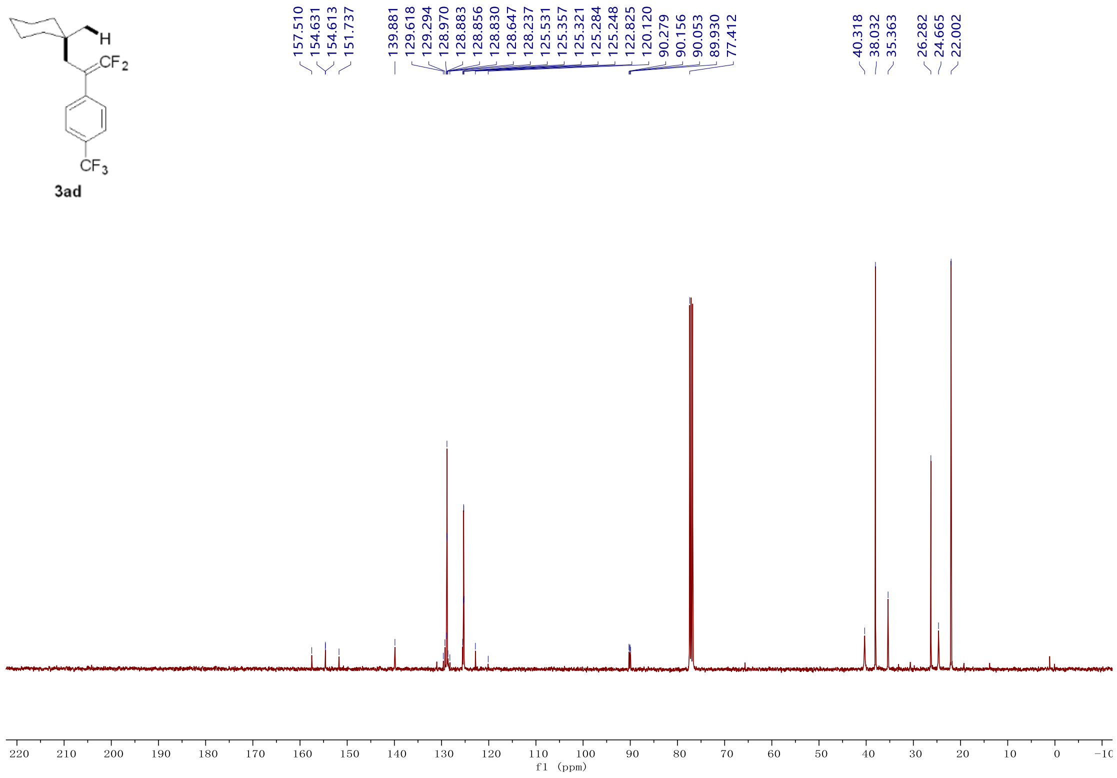

Supplementary Figure 210. ^{13}C NMR spectrum of compound **3ad**

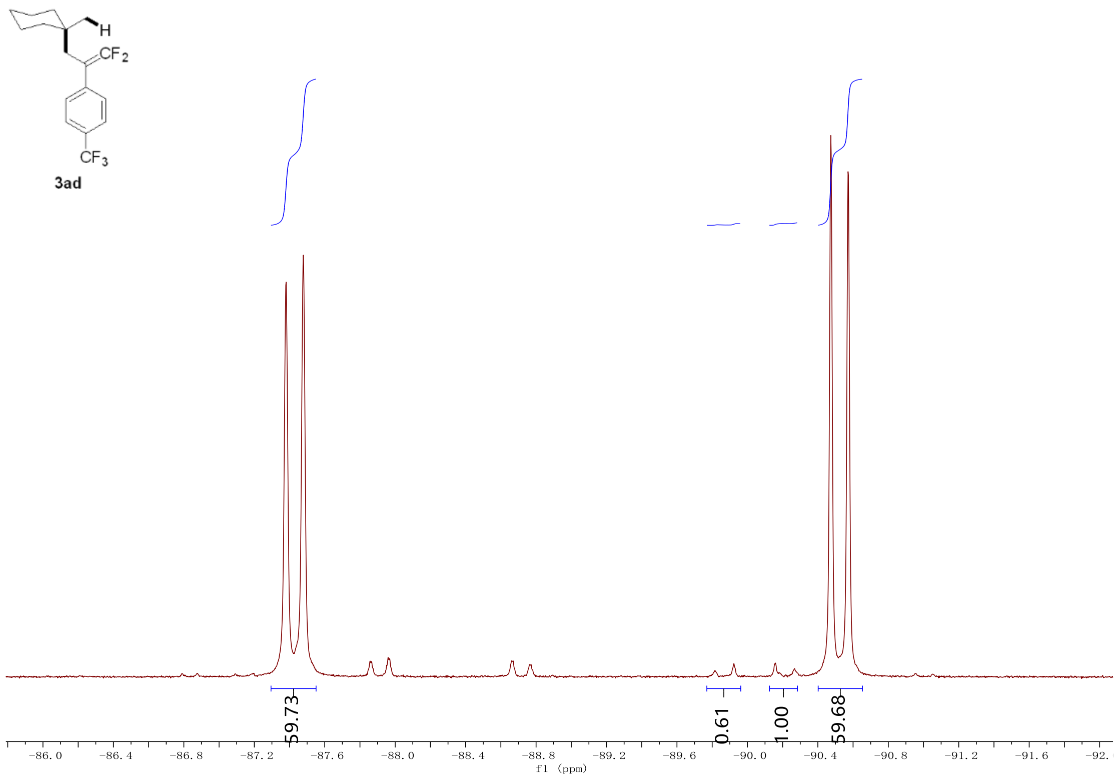

Supplementary Figure 211. Crude ^{19}F NMR spectrum of compound **3ad** rr = 60 : 1

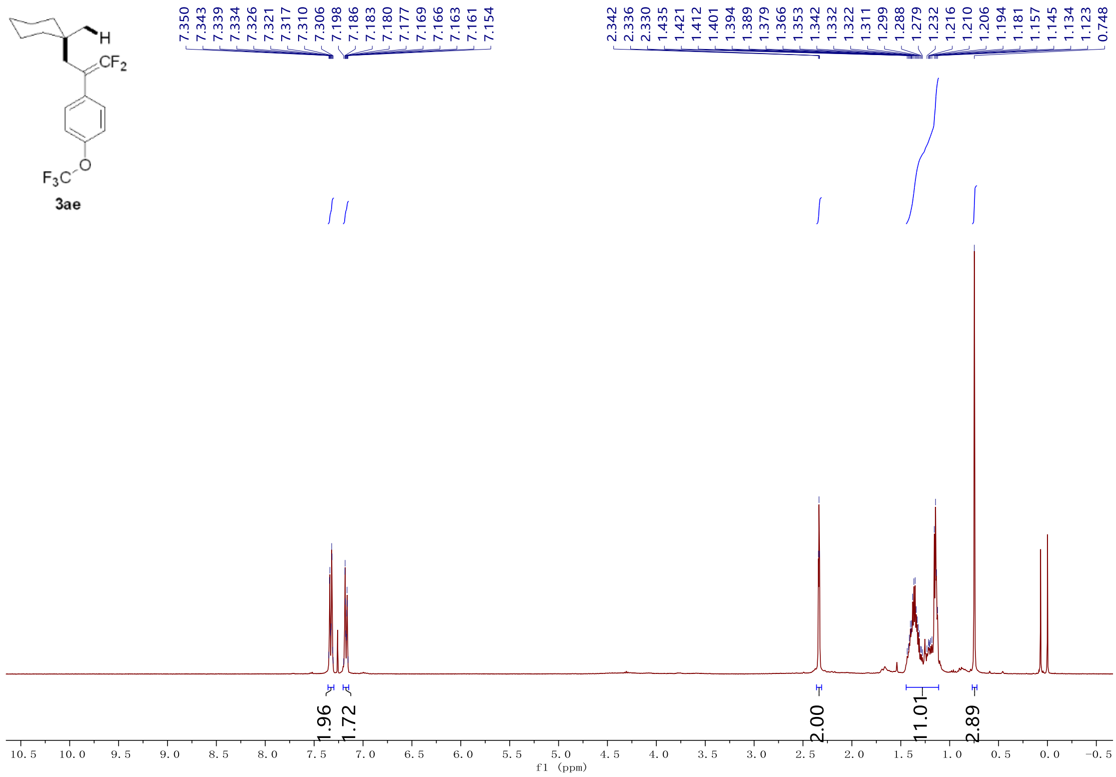

Supplementary Figure 212. ^{1}H NMR spectrum of compound **3ae**

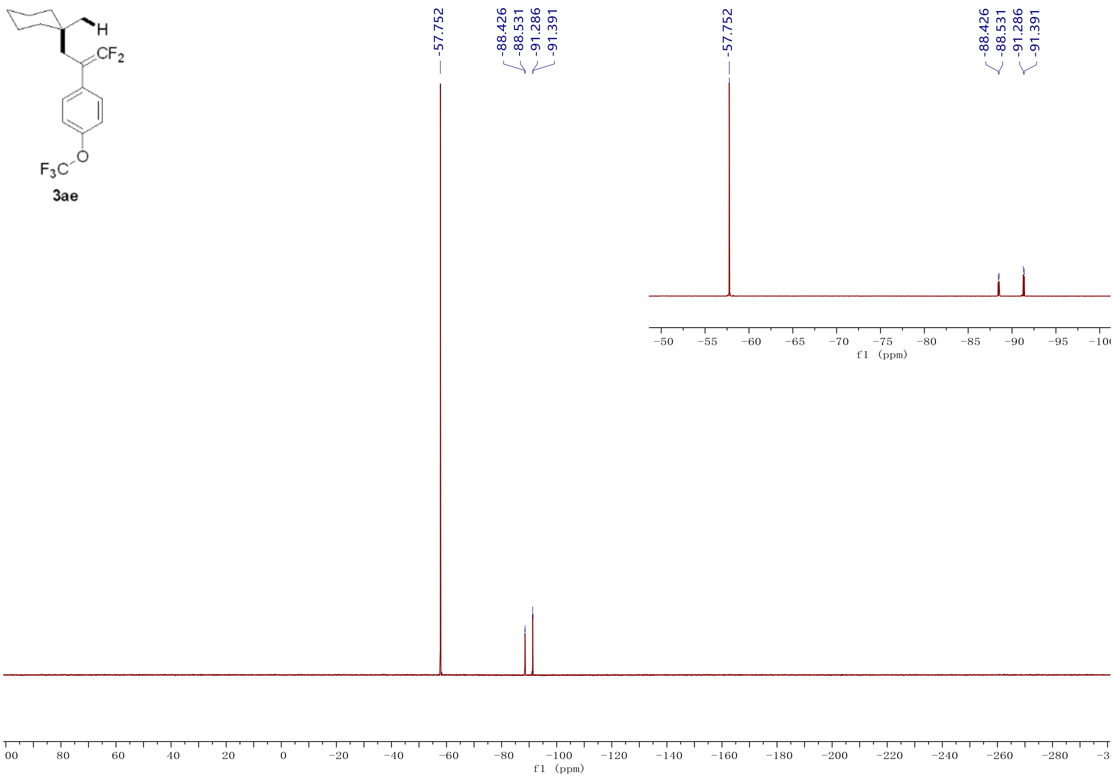

Supplementary Figure 213. ^{19}F NMR spectrum of compound **3ae**

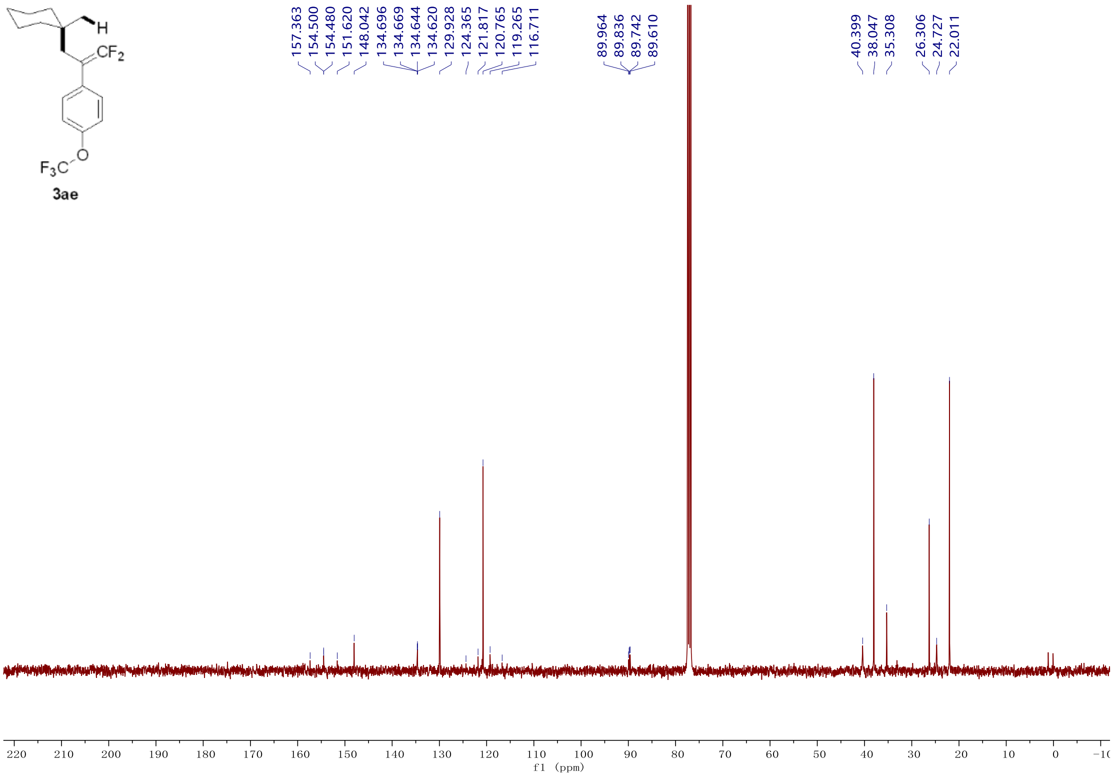

Supplementary Figure 214. ^{13}C NMR spectrum of compound **3ae**

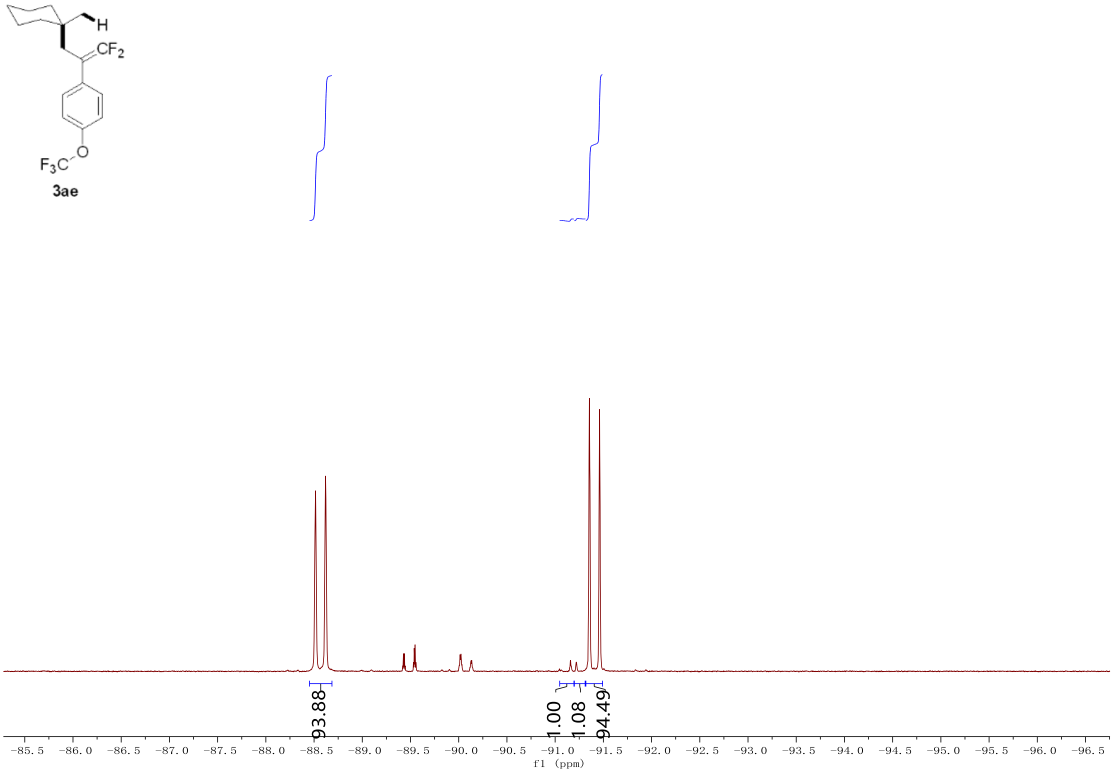

Supplementary Figure 215. Crude ^{19}F NMR spectrum of compound **3ae** rr = 90 : 1

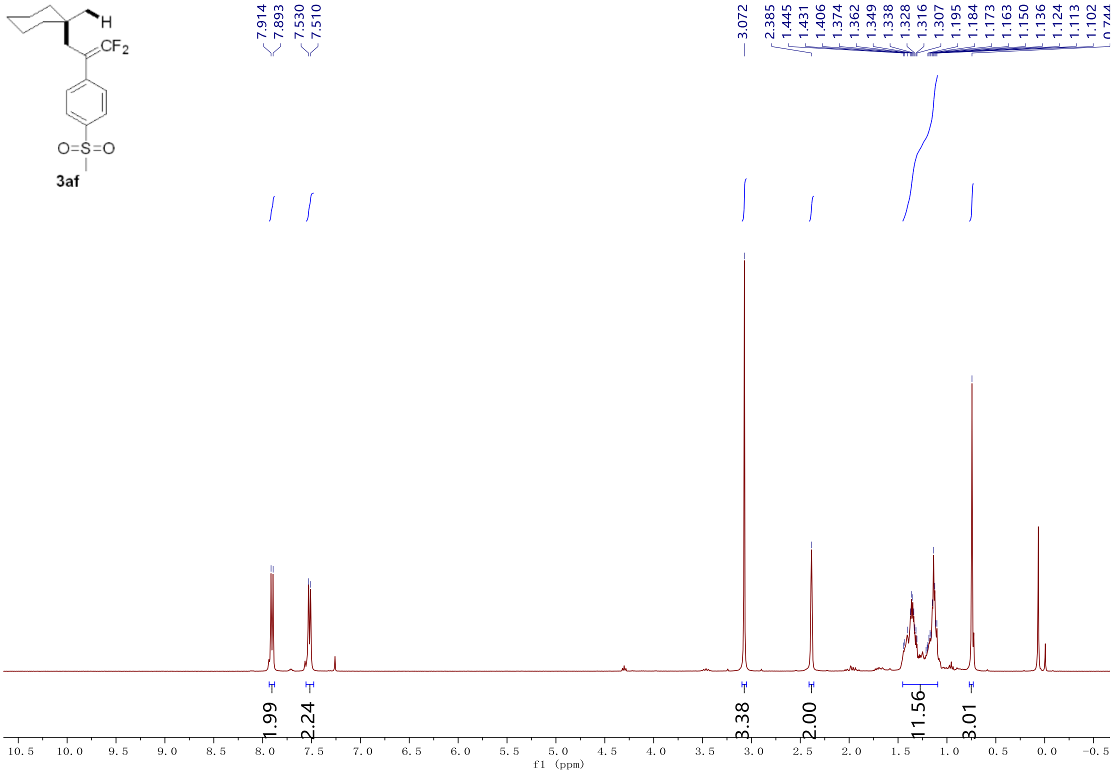

Supplementary Figure 216. ^{1}H NMR spectrum of compound **3af**

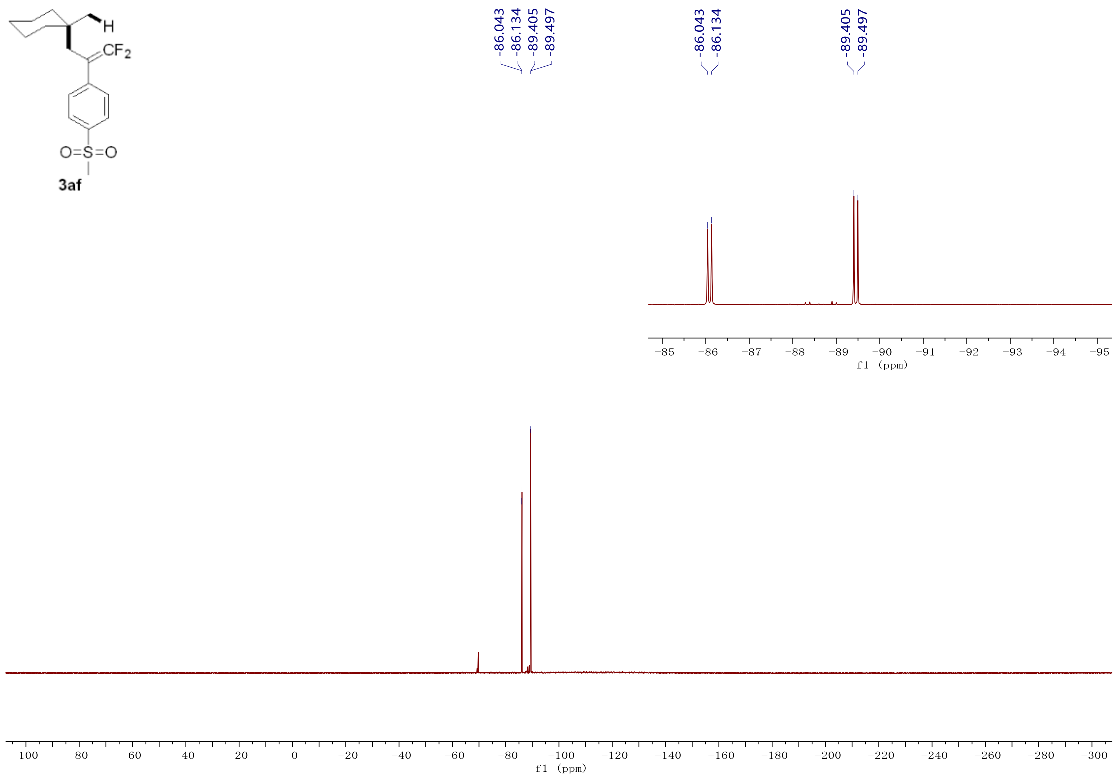

Supplementary Figure 217. ^{19}F NMR spectrum of compound **3af**

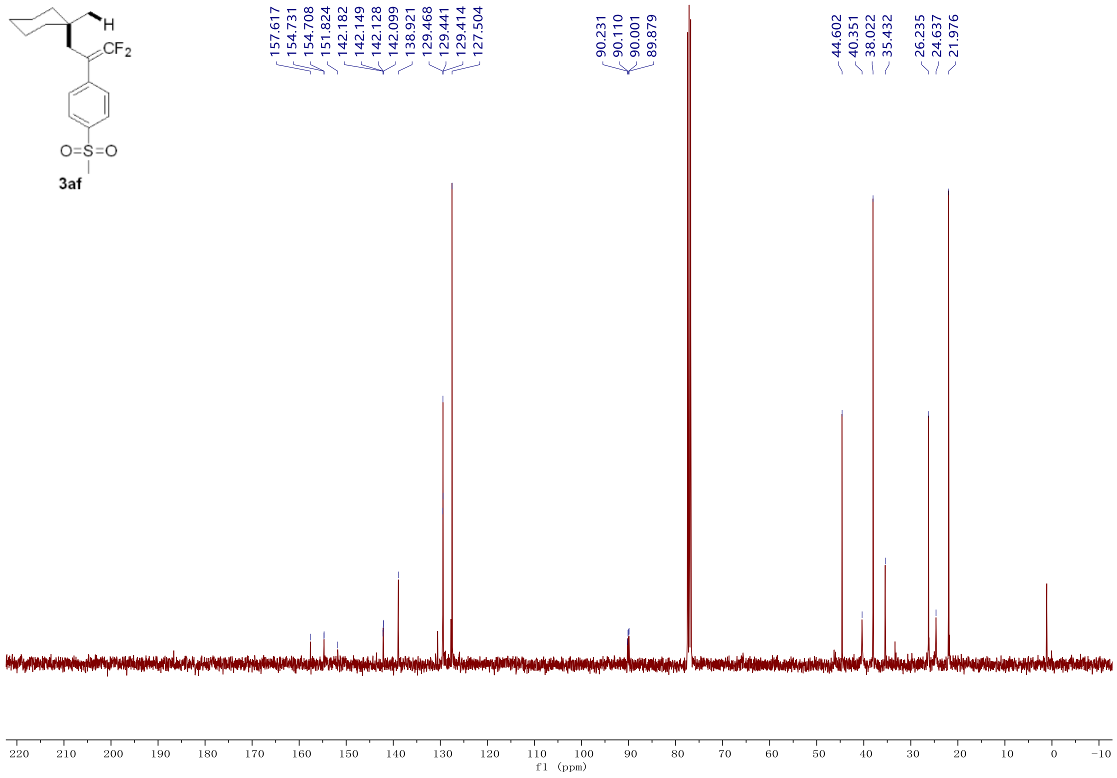

Supplementary Figure 218. ^{13}C NMR spectrum of compound **3af**

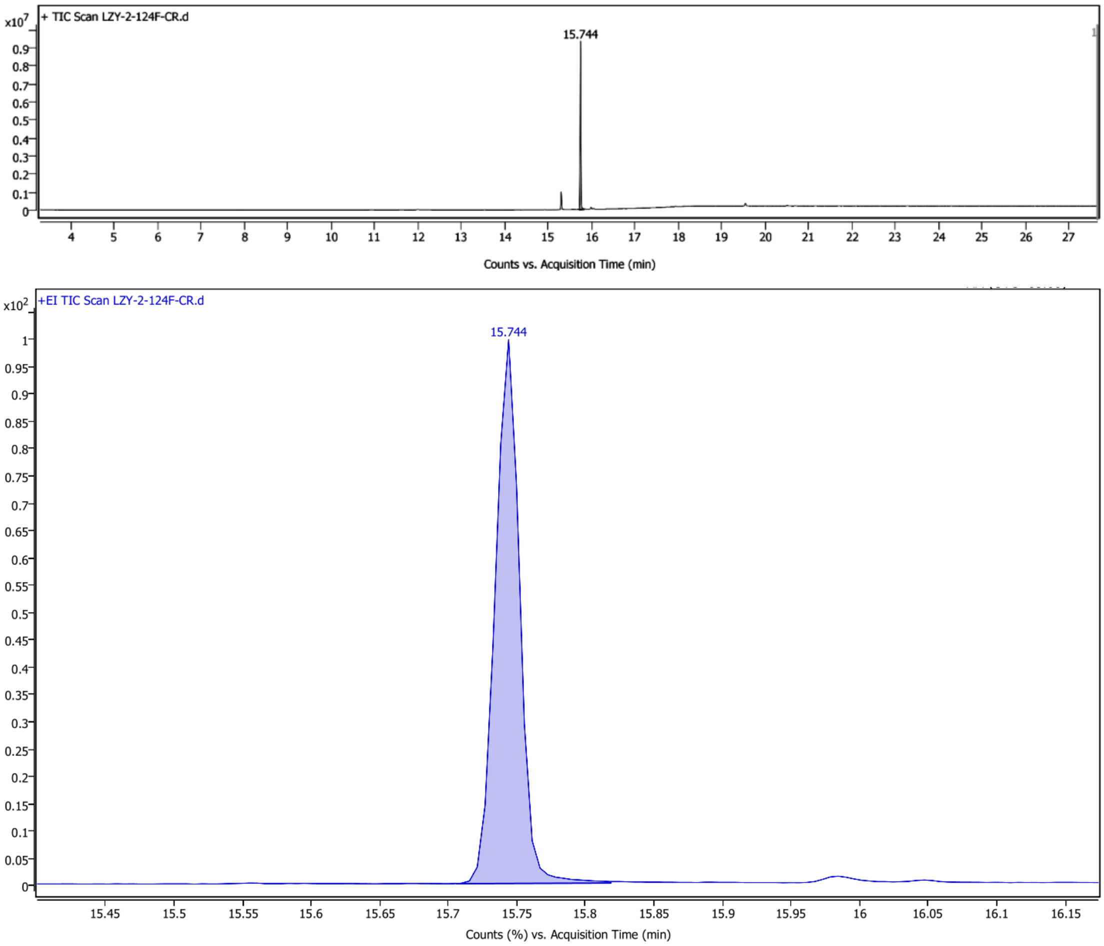

Chromatogram Peaks

Peak	Start	RT	End	Height	Area	Area %	SNR
1	15.706	15.744	15.818	9340737	11617886	100.00	

Supplementary Figure 219. GC spectrum of crude products of **3af** rr > 20 : 1

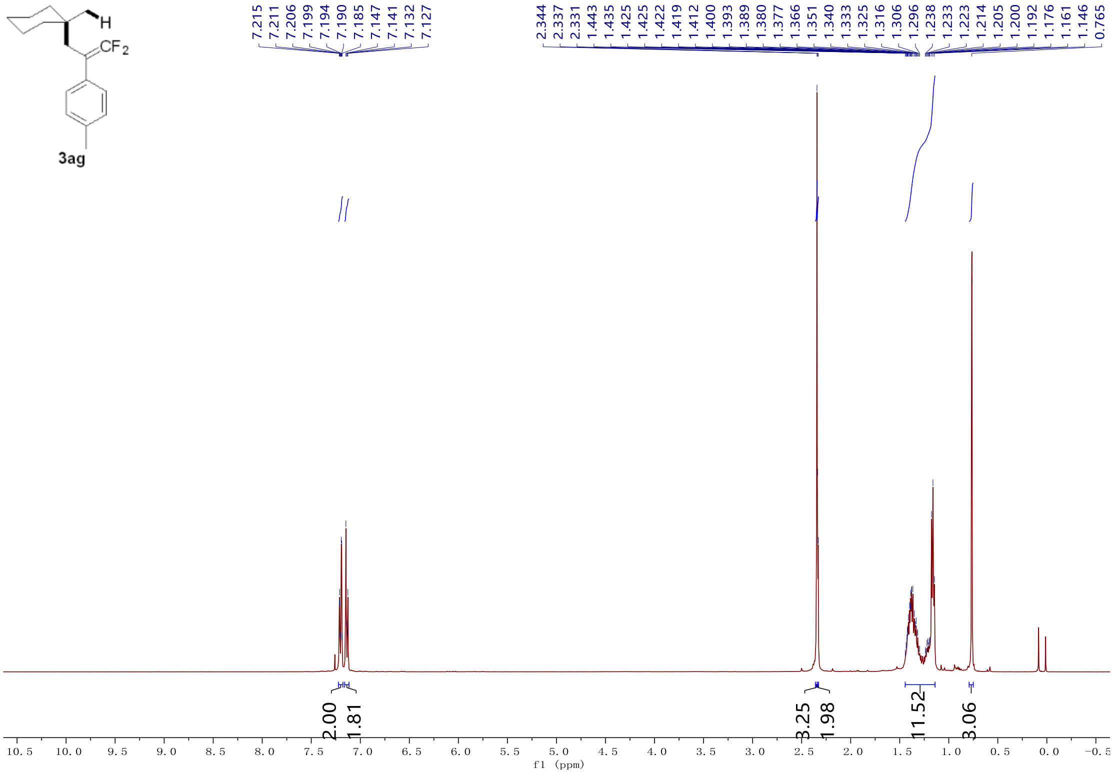

Supplementary Figure 220. ^{1}H NMR spectrum of compound **3ag**

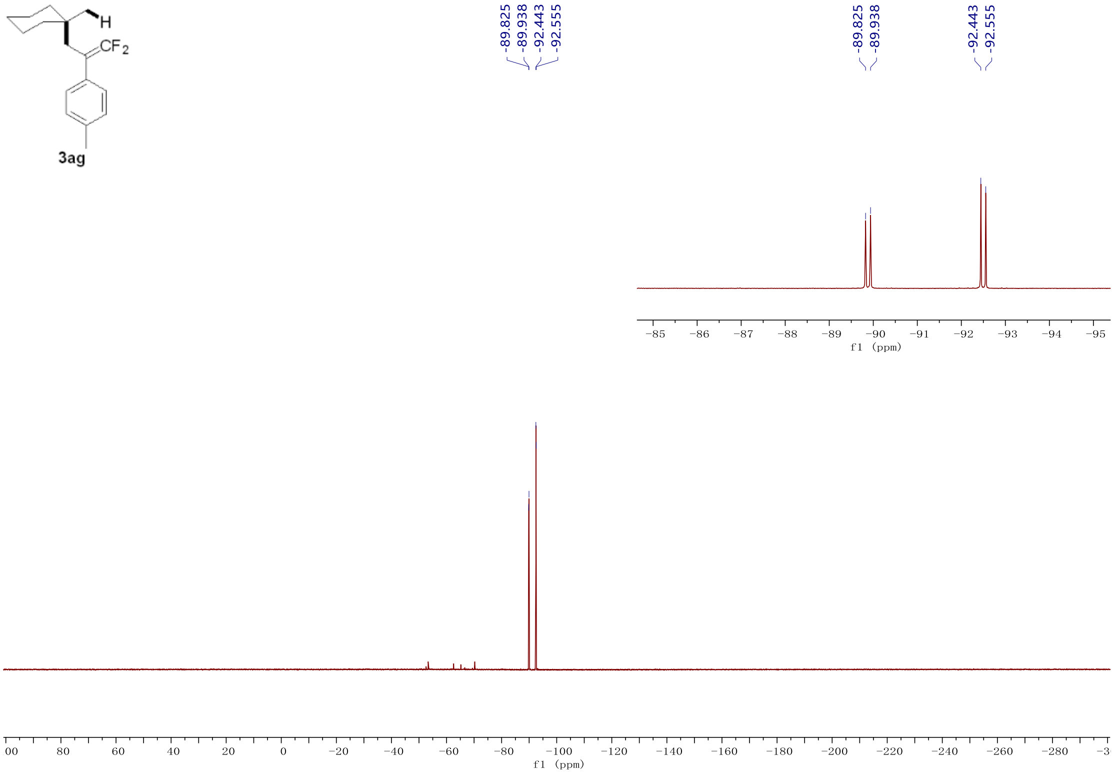

Supplementary Figure 221. ^{19}F NMR spectrum of compound **3ag**

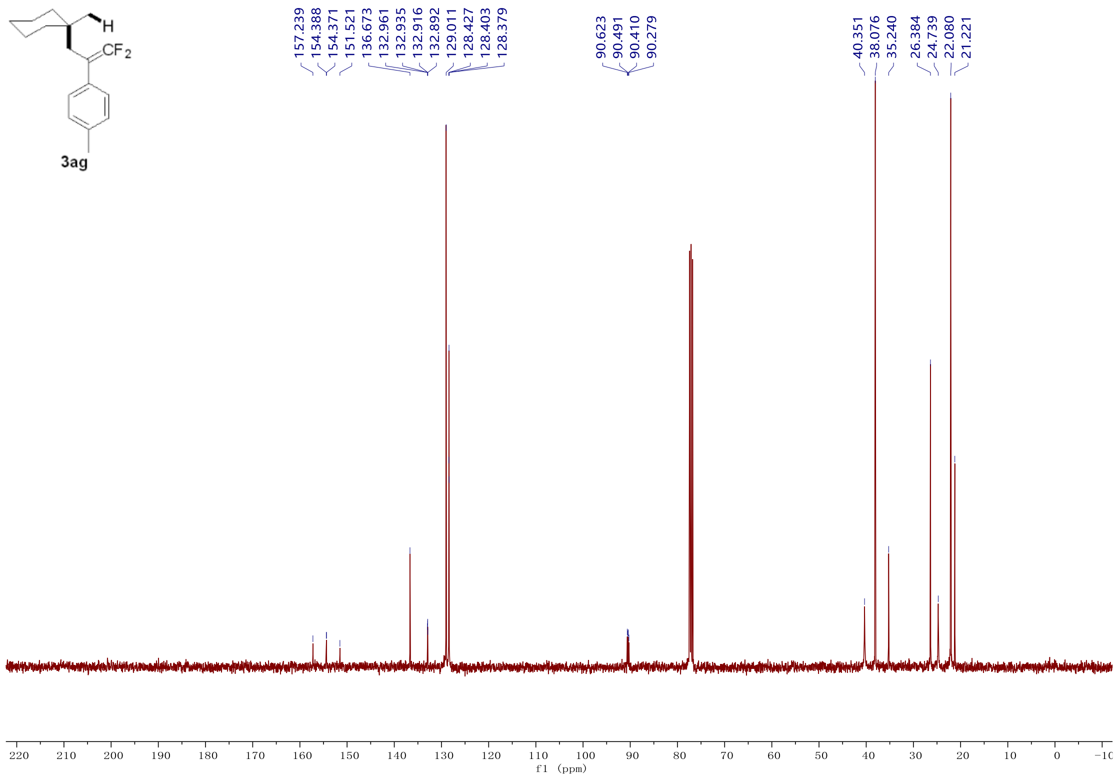

Supplementary Figure 222. ^{13}C NMR spectrum of compound **3ag**

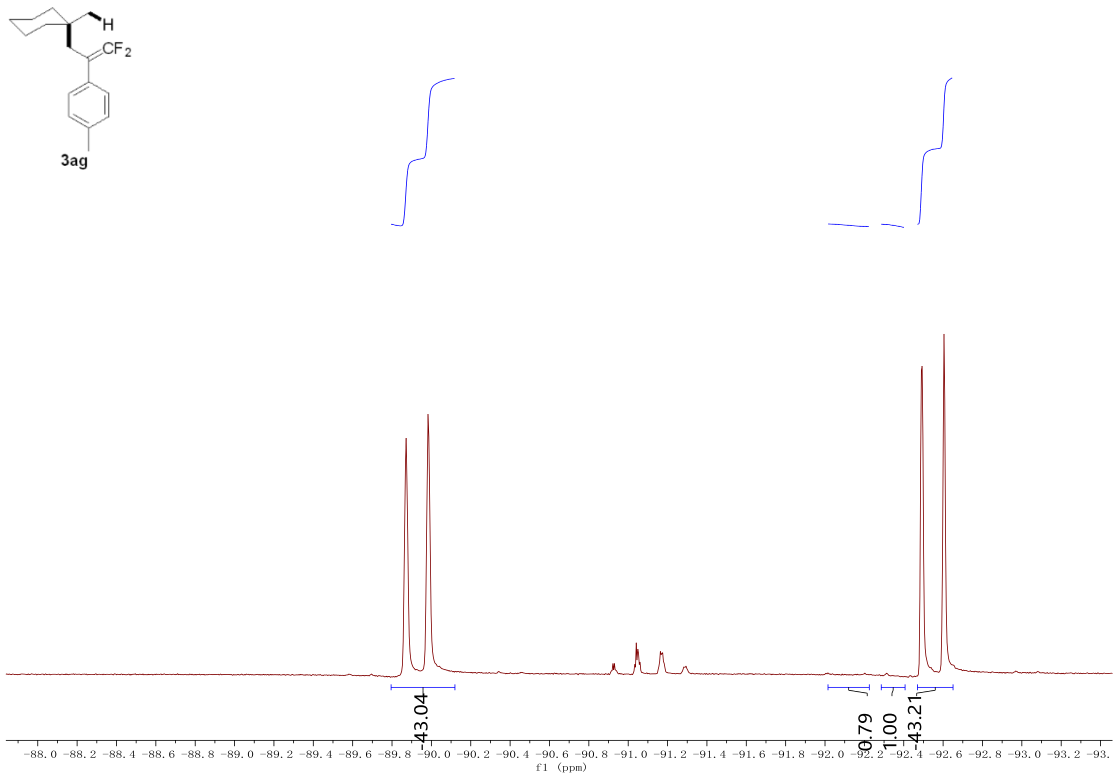

Supplementary Figure 223. Crude ^{19}F NMR spectrum of compound **3ag** rr = 43 : 1

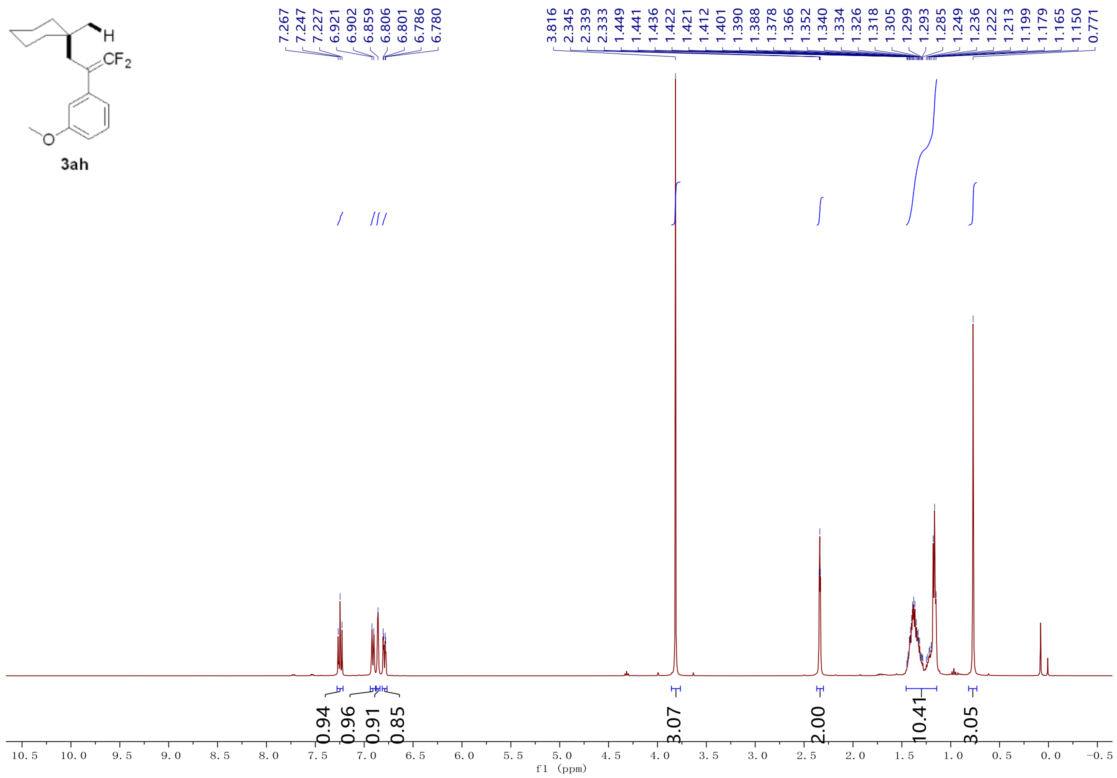

Supplementary Figure 224. ^{1}H NMR spectrum of compound **3ah**

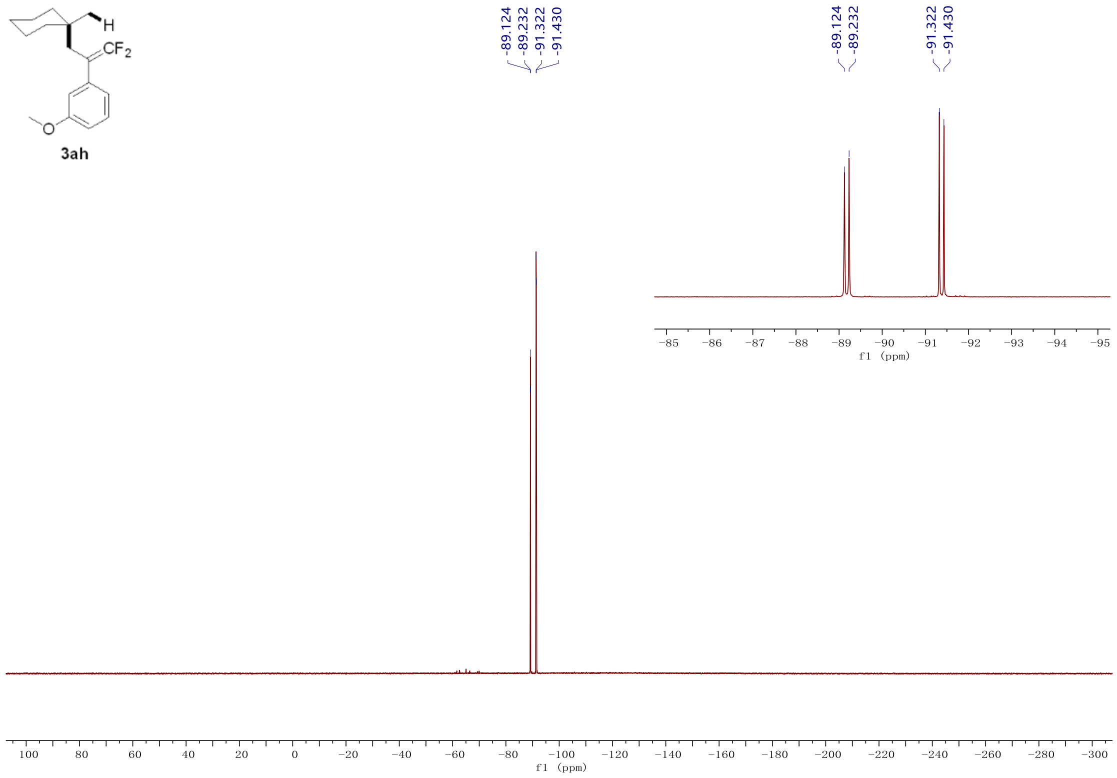

Supplementary Figure 225. ^{19}F NMR spectrum of compound **3ah**

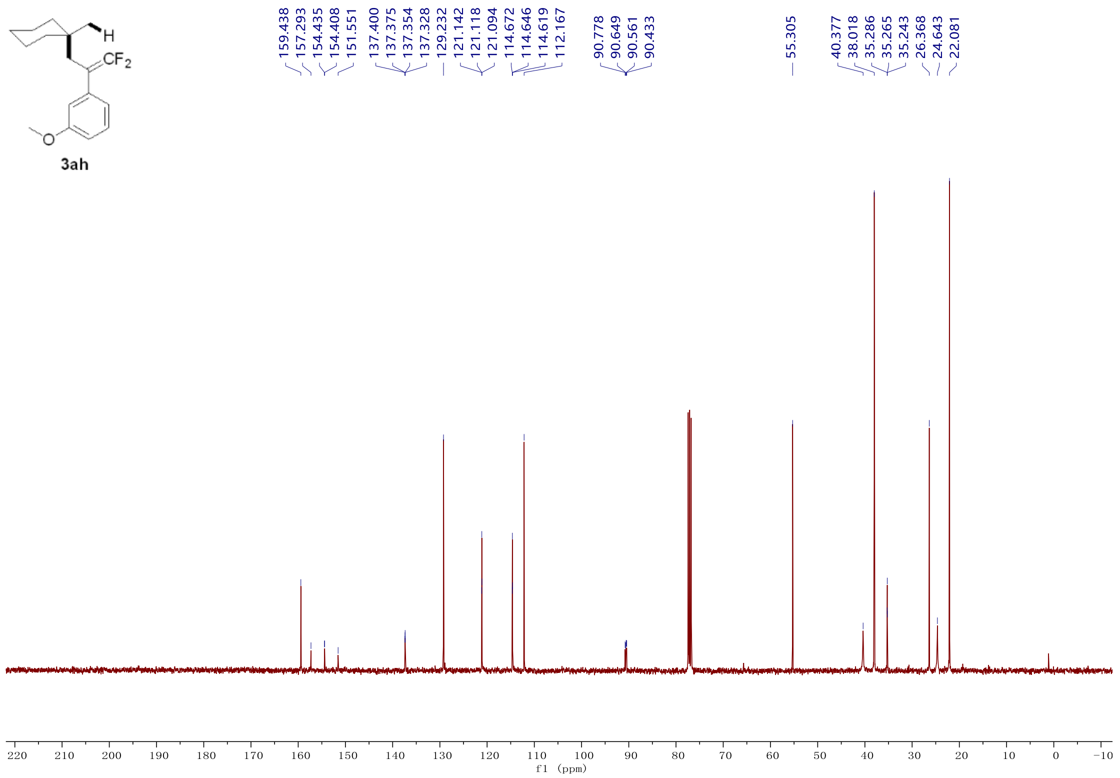

Supplementary Figure 226. ^{13}C NMR spectrum of compound **3ah**

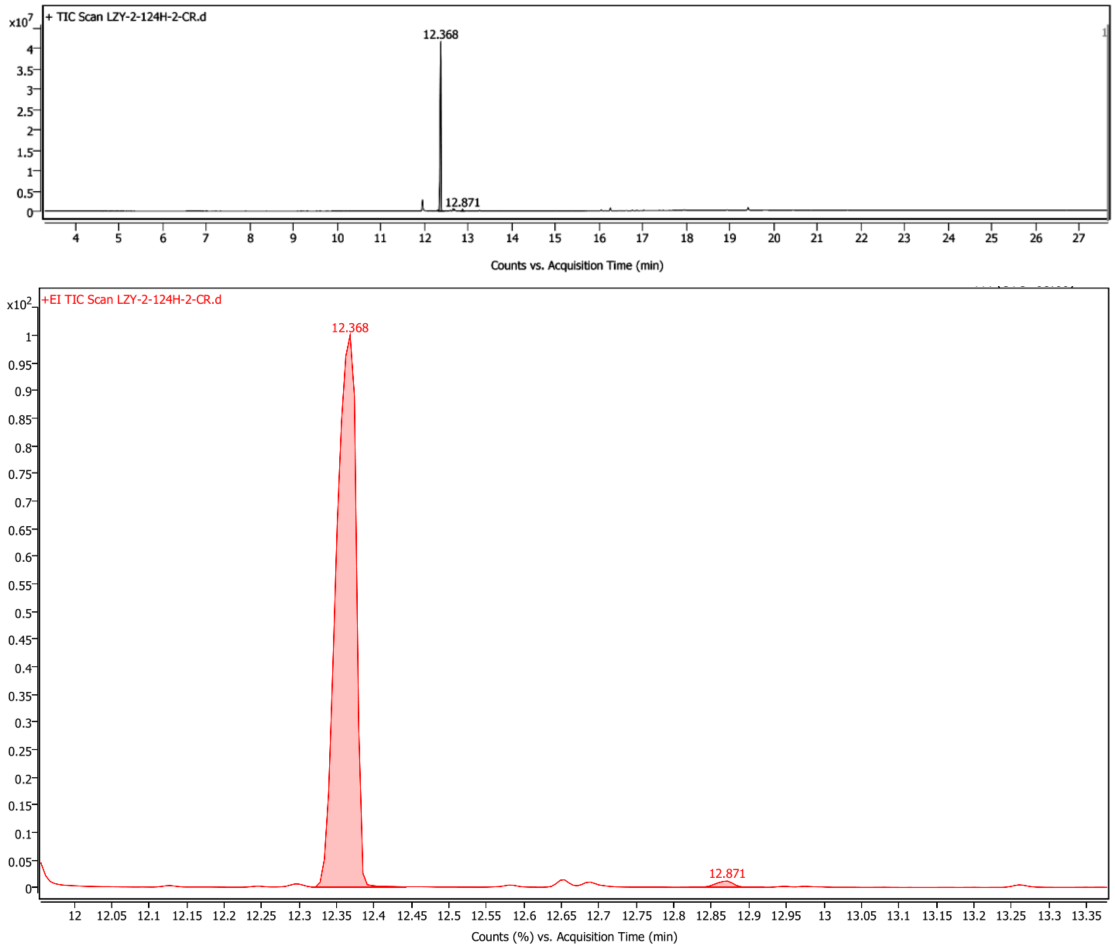

Chromatogram Peaks

Peak	Start	RT	End	Height	Area	Area %	SNR
1	12.316	12.368	12.442	41890355	76586237	100.00	
2	12.821	12.871	12.921	471115	783273	1.02	

Supplementary Figure 227. GC spectrum of crude products of **3ah** rr > 20 : 1

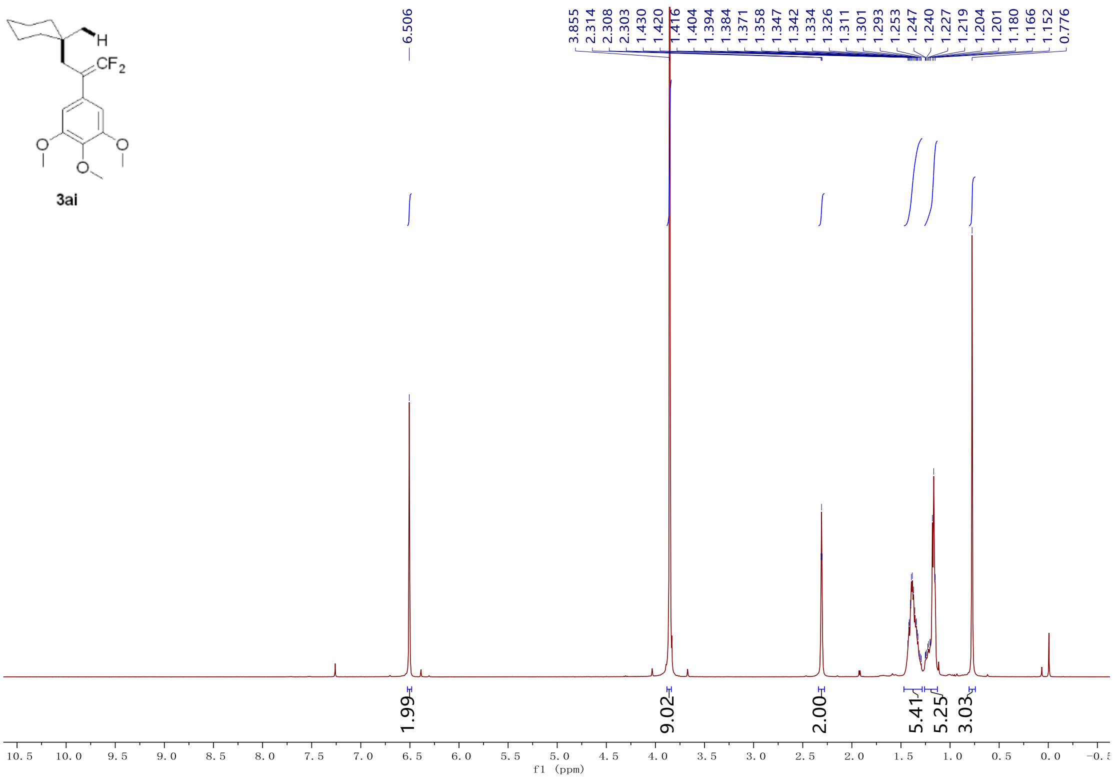

Supplementary Figure 228. ^{1}H NMR spectrum of compound **3ai**

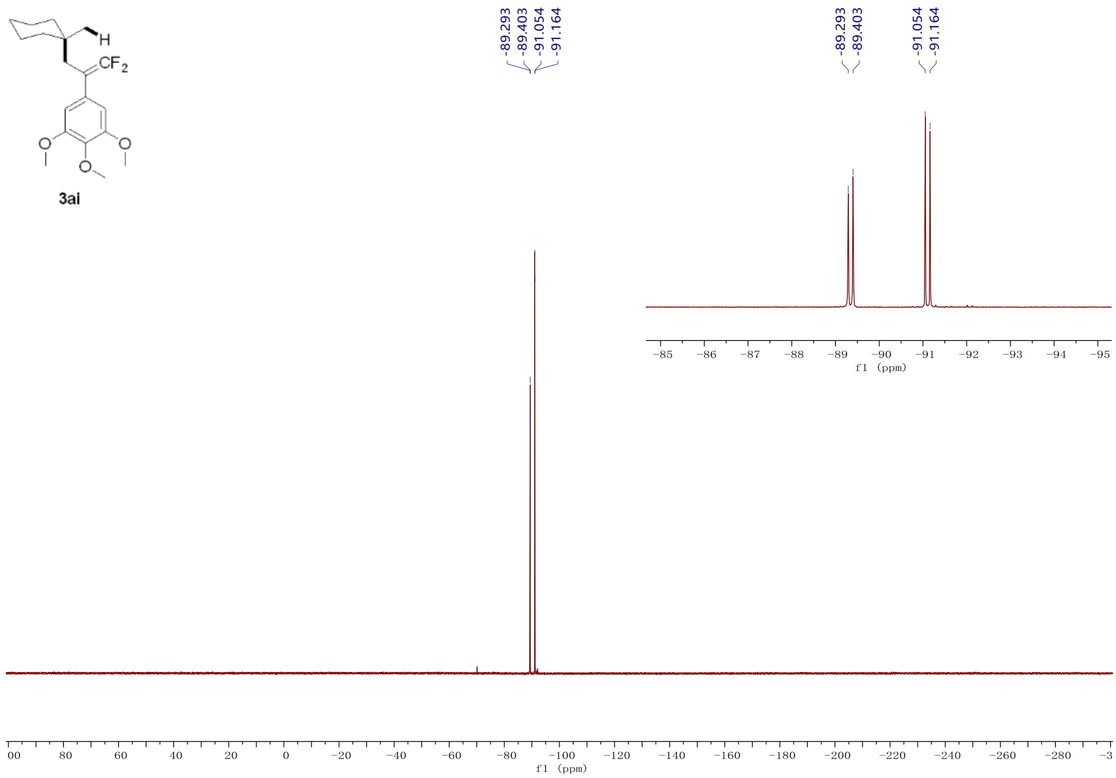

Supplementary Figure 229. ^{19}F NMR spectrum of compound **3ai**

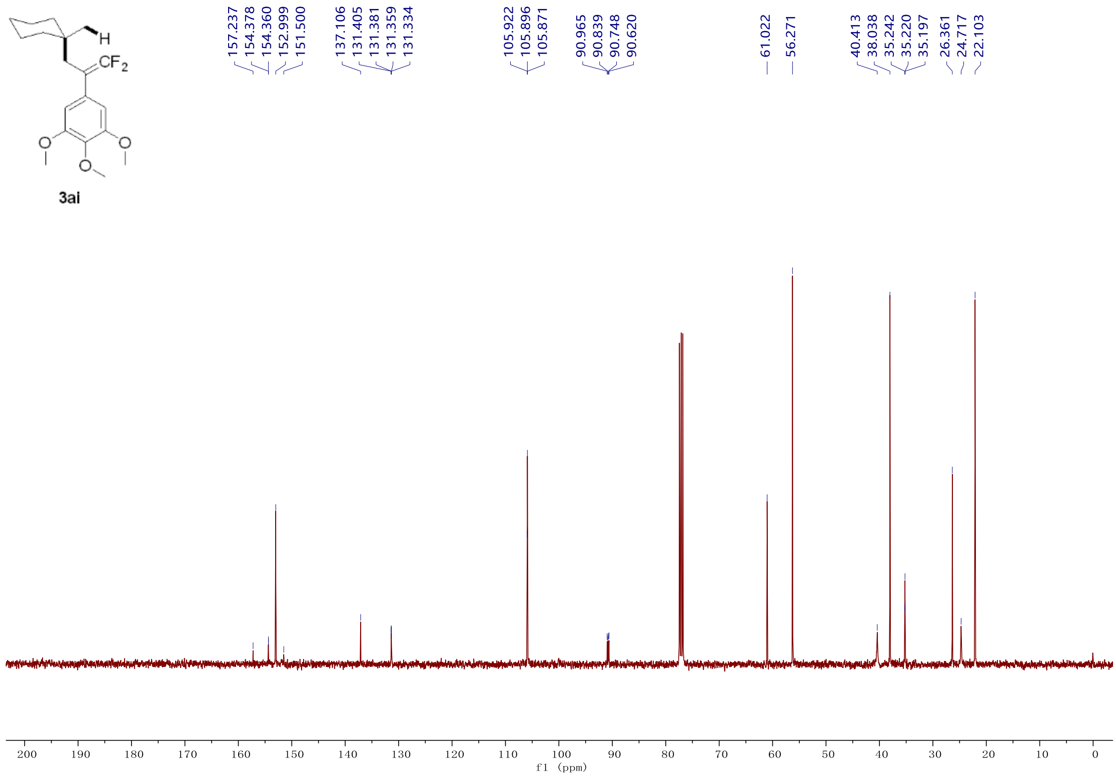

Supplementary Figure 230. ^{13}C NMR spectrum of compound **3ai**

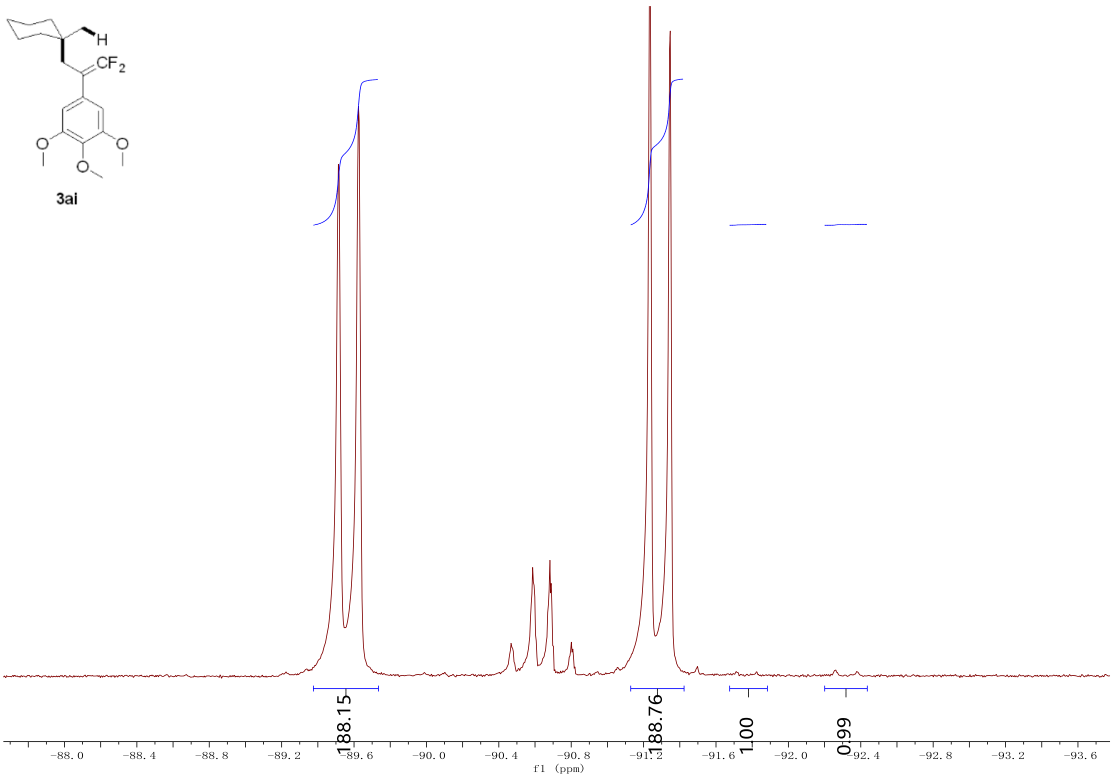

Supplementary Figure 231. Crude ^{19}F NMR spectrum of compound **3ai** rr > 100 : 1

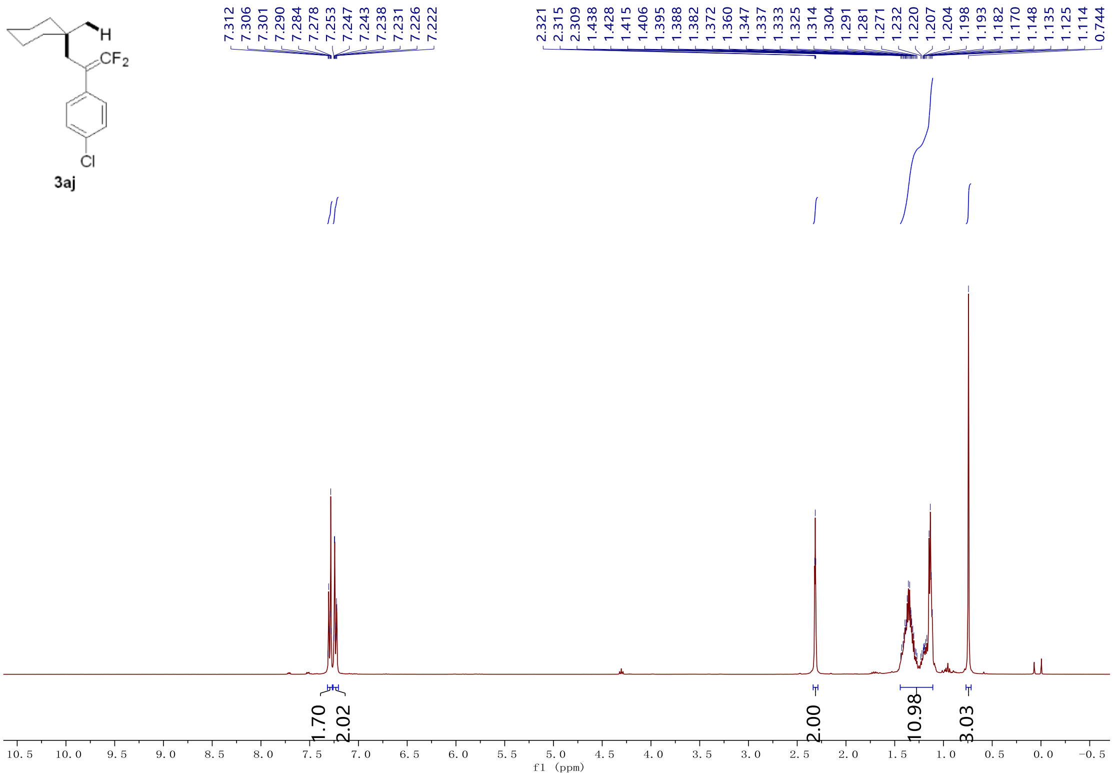

Supplementary Figure 232. ^{1}H NMR spectrum of compound **3aj**

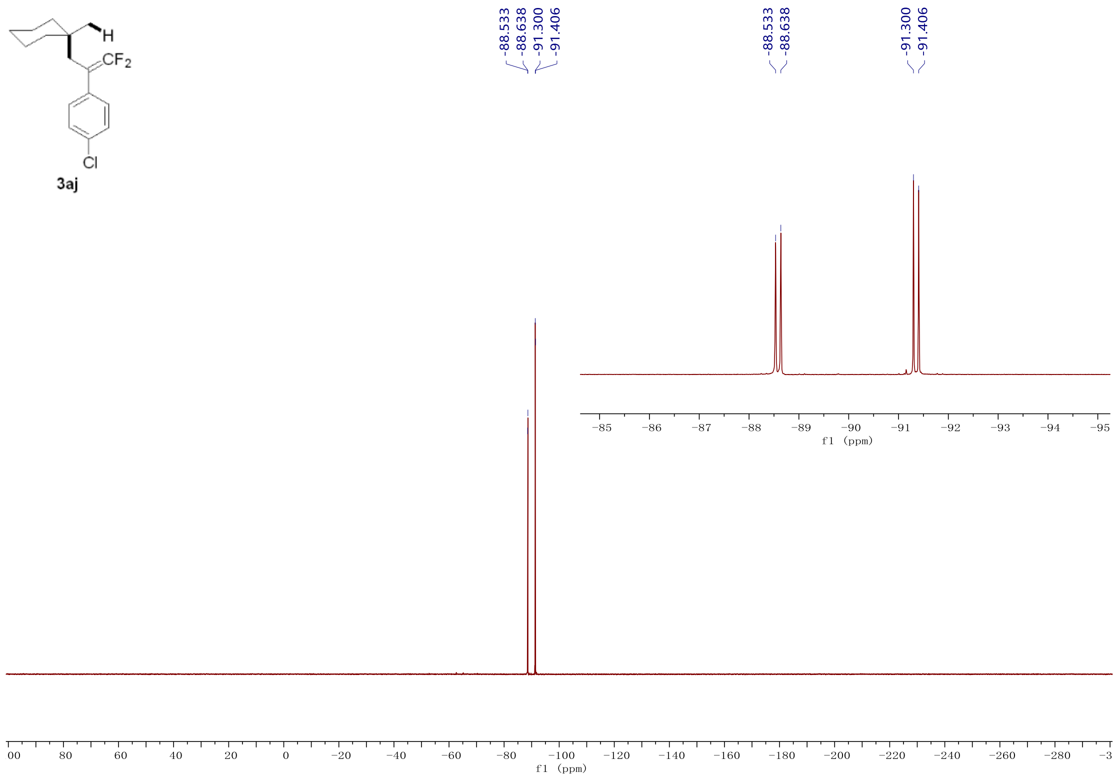

Supplementary Figure 233. ^{19}F NMR spectrum of compound **3aj**

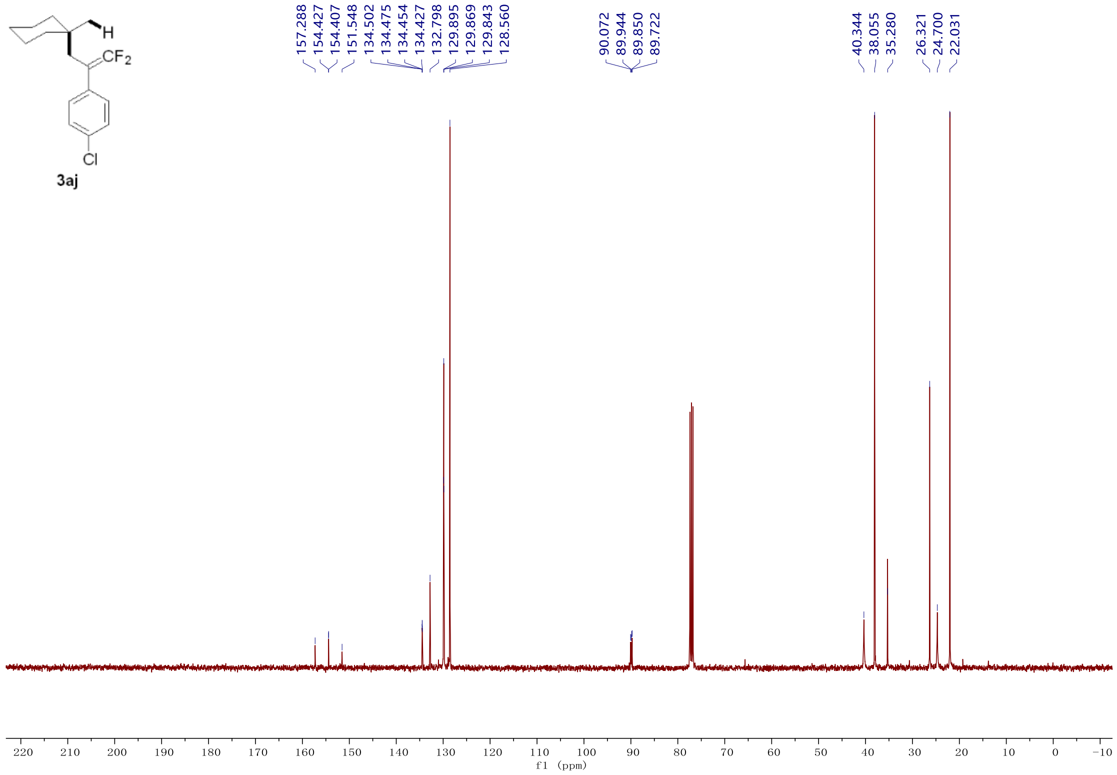

Supplementary Figure 234. ^{13}C NMR spectrum of compound **3aj**

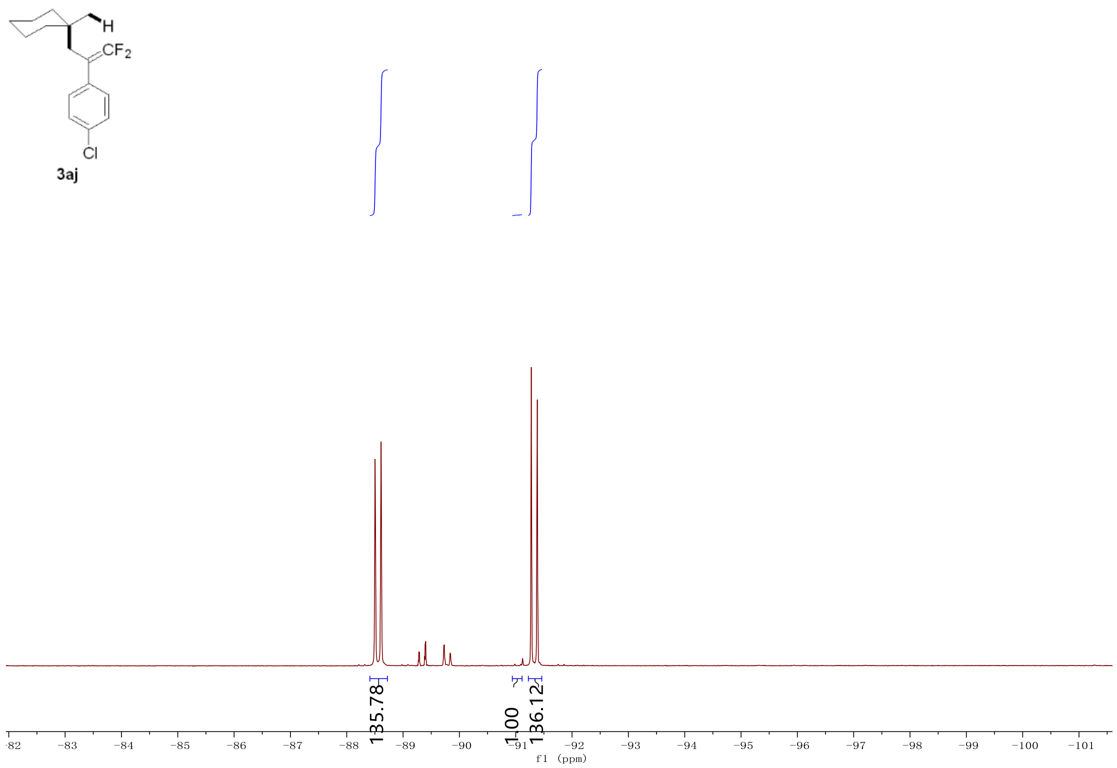

Supplementary Figure 235. Crude ^{13}C NMR spectrum of compound **3aj** rr > 100 : 1

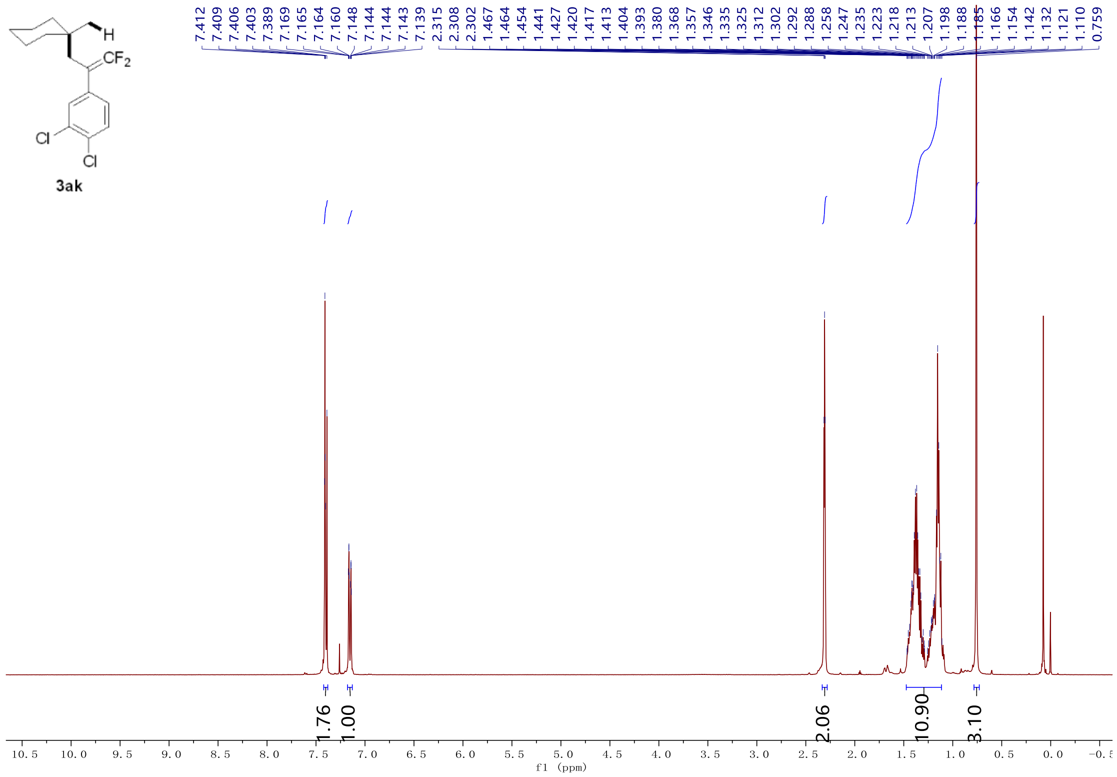

Supplementary Figure 236. ^{1}H NMR spectrum of compound **3ak**

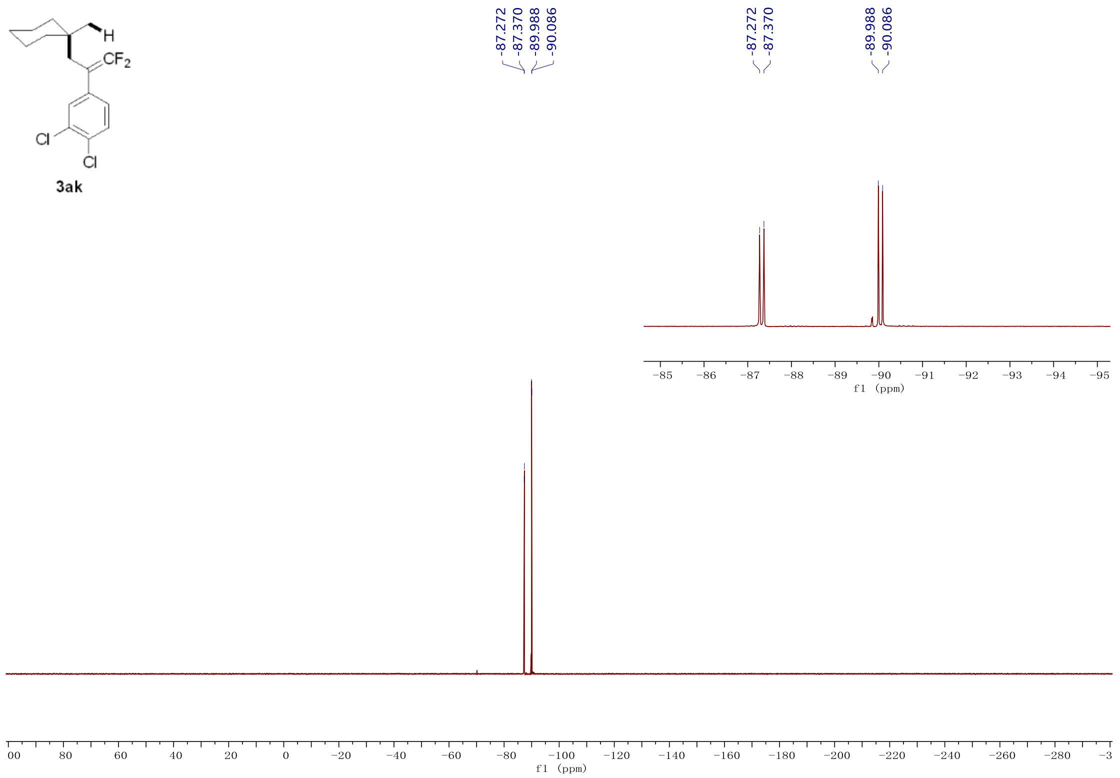

Supplementary Figure 237. ^{19}F NMR spectrum of compound **3ak**

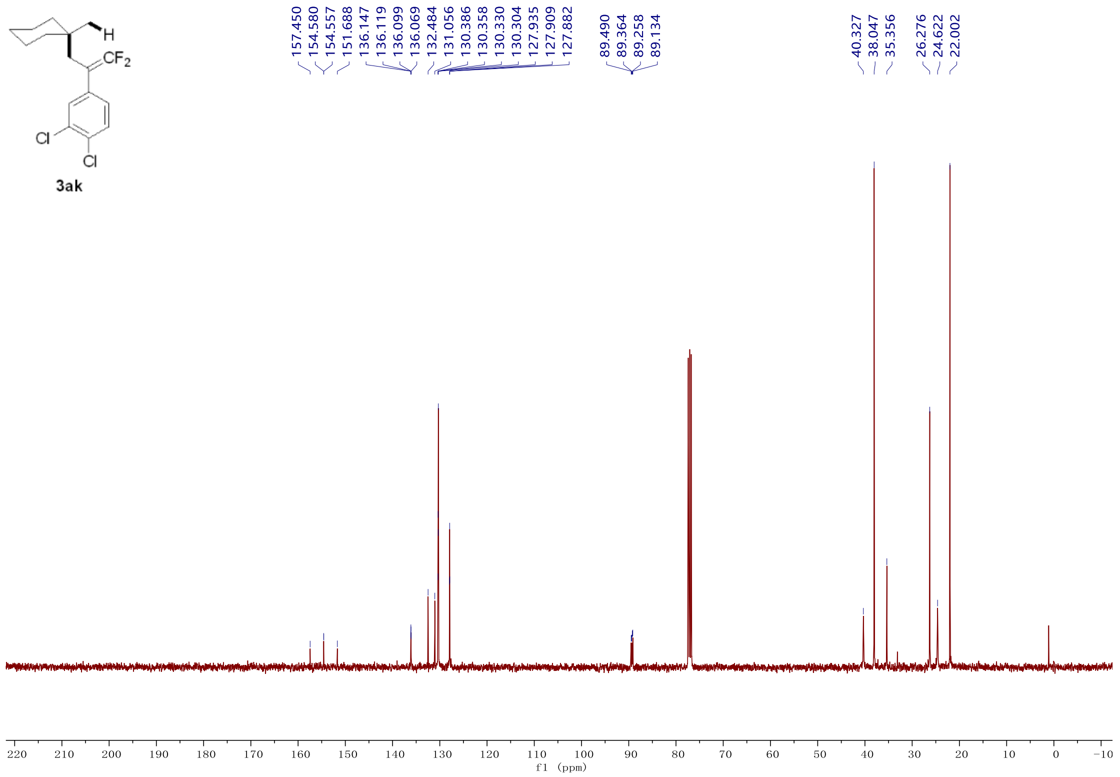

Supplementary Figure 238. ^{13}C NMR spectrum of compound **3ak**

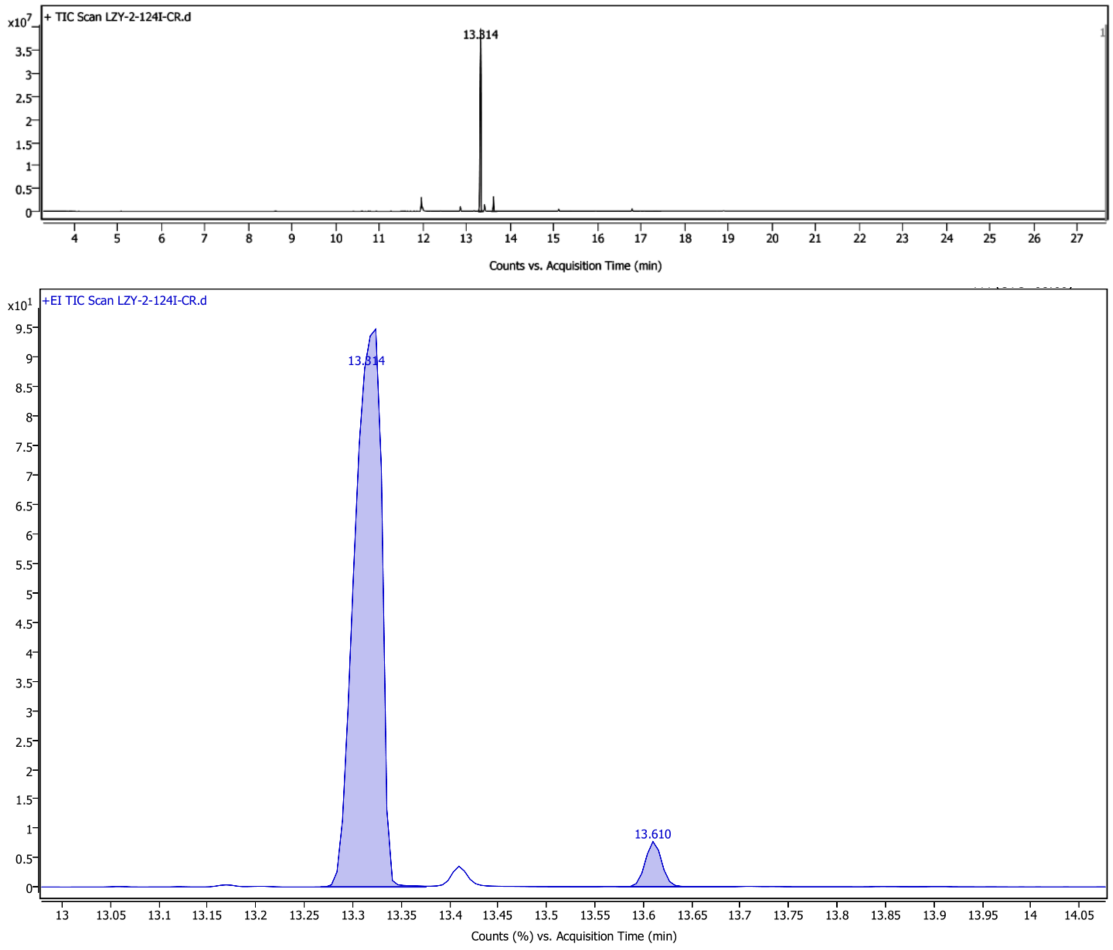

Chromatogram Peaks

Peak	Start	RT	End	Height	Area	Area %	SNR
1	13.267	13.314	13.375	36959270	77274715	100.00	
2	13.576	13.610	13.679	3224266	3865140	5.00	

Supplementary Figure 239. GC spectrum of compound **3ak** rr = 20 : 1

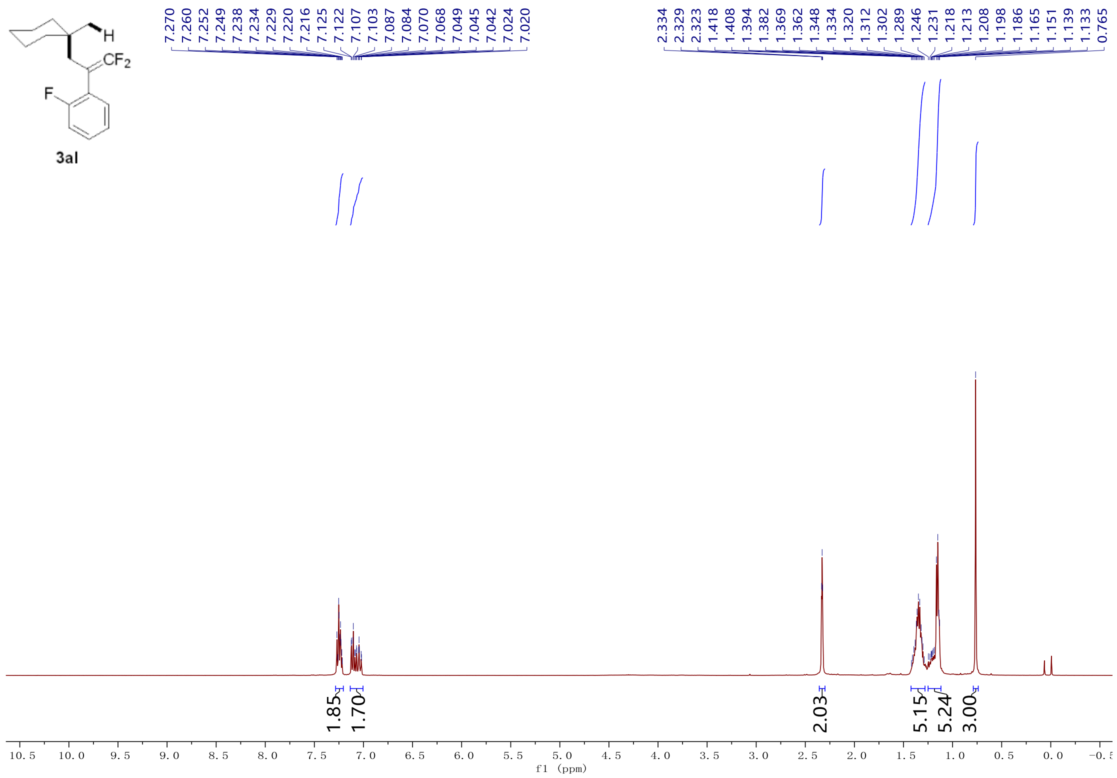

Supplementary Figure 240. ^{1}H NMR spectrum of compound **3al**

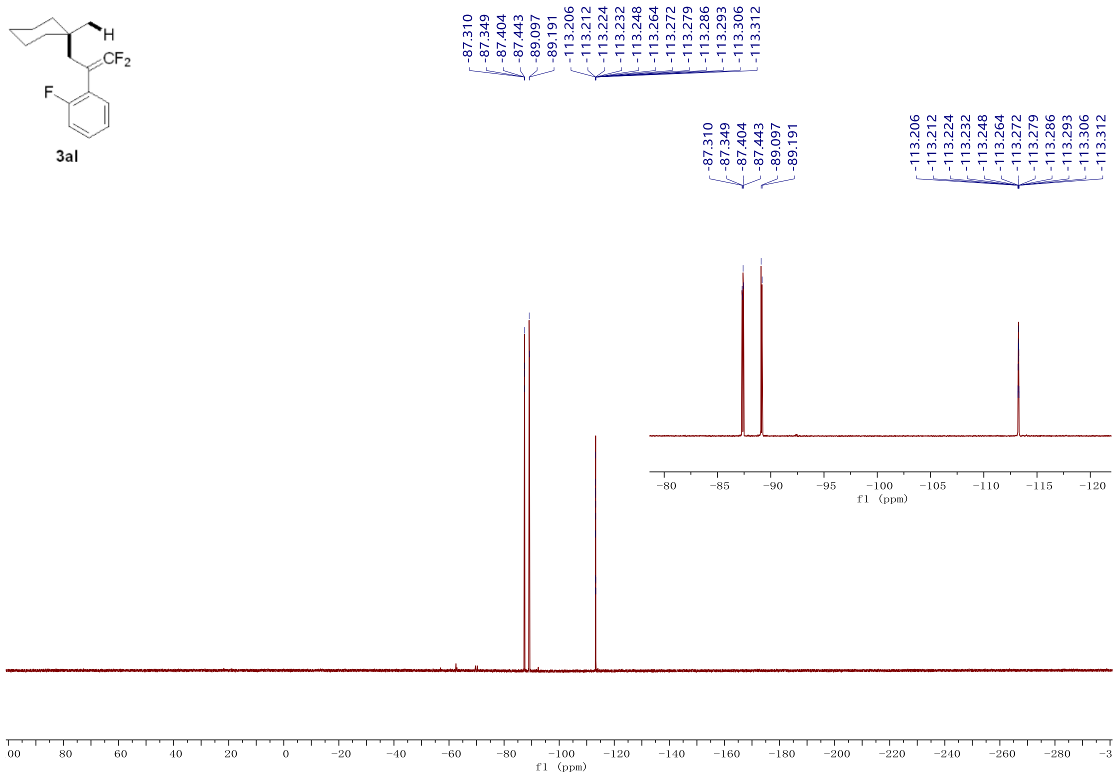

Supplementary Figure 241. ^{19}F NMR spectrum of compound **3al**

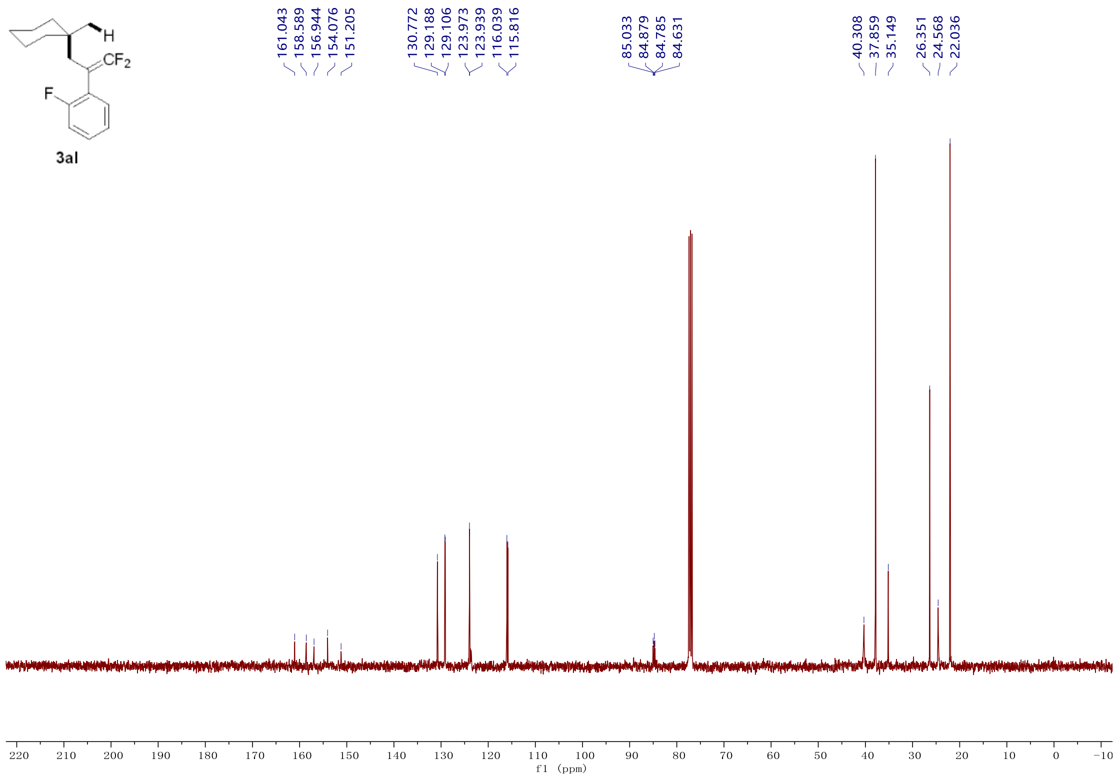

Supplementary Figure 242. ^{13}C NMR spectrum of compound **3al**

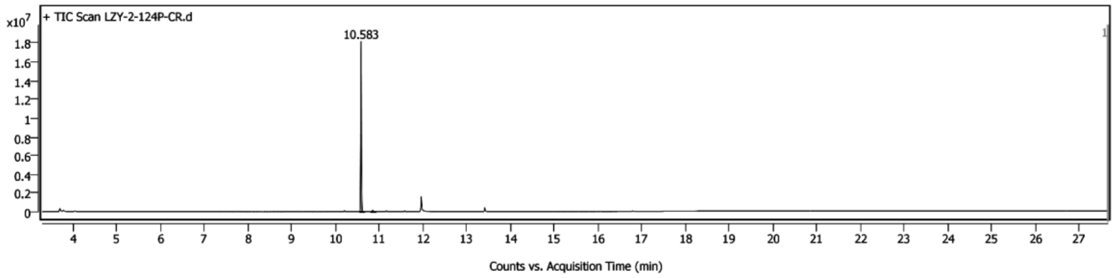

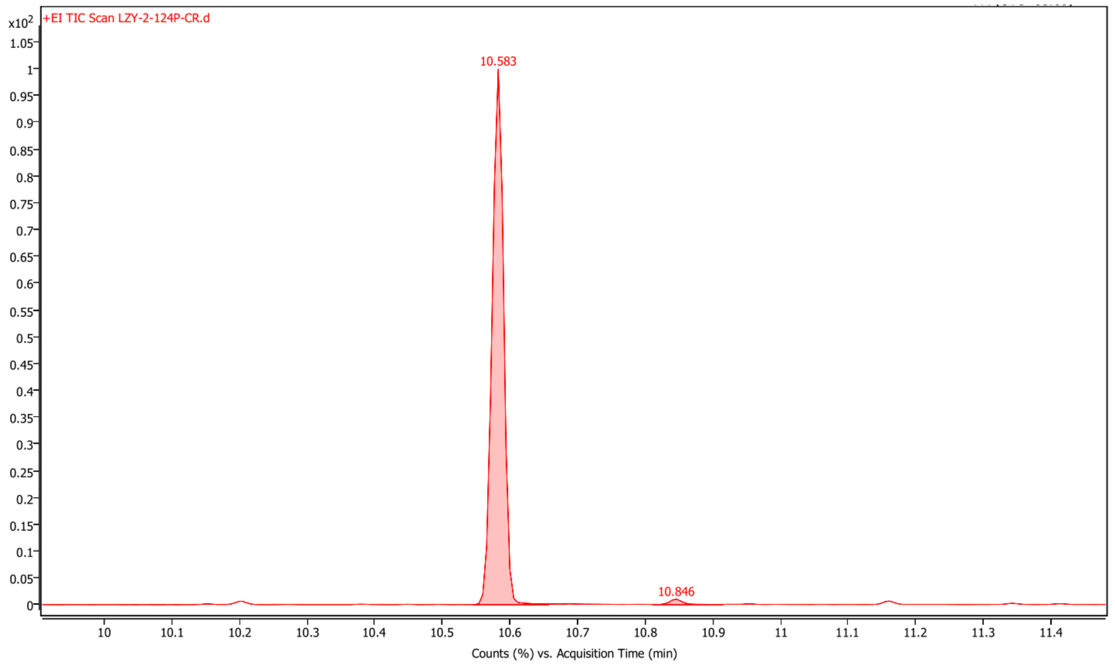

Chromatogram Peaks

Peak	Start	RT	End	Height	Area	Area %	SNR
1	10.545	10.583	10.657	18205730	21651001	100.00	
2	10.812	10.846	10.915	183549	250330	1.16	

Supplementary Figure 243. GC spectrum of compound **3al** rr > 20 : 1

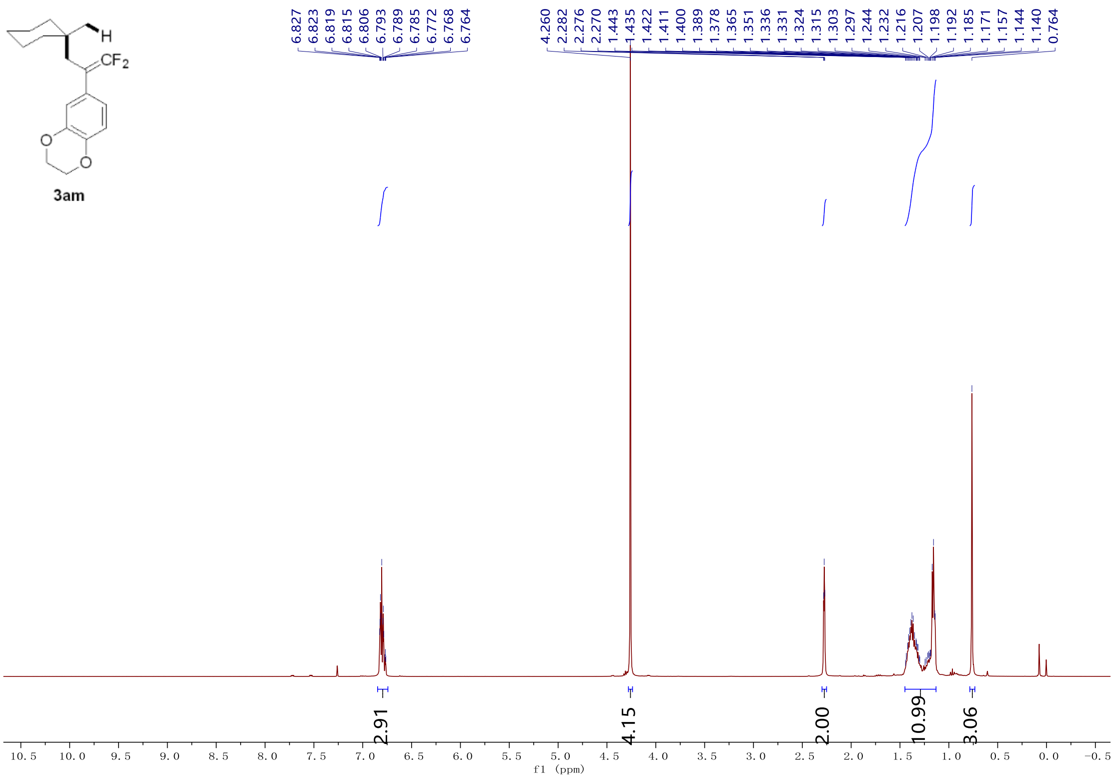

Supplementary Figure 244. ^{1}H NMR spectrum of compound **3am**

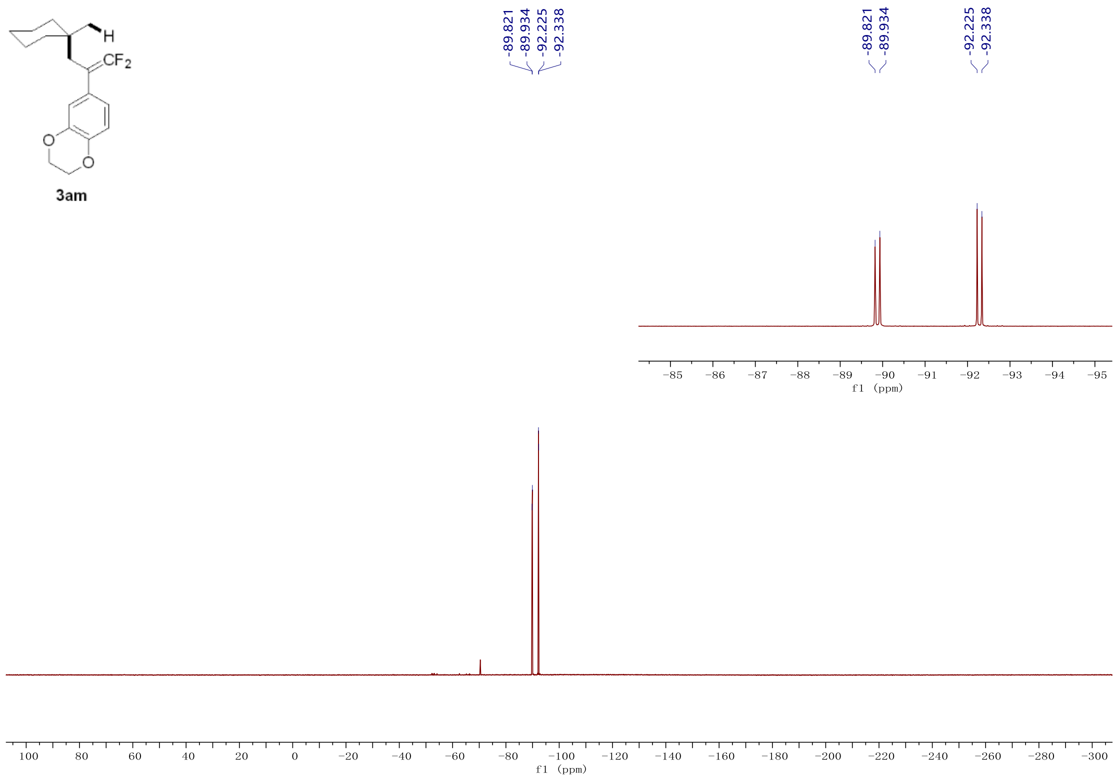

Supplementary Figure 245. ^{19}F NMR spectrum of compound **3am**

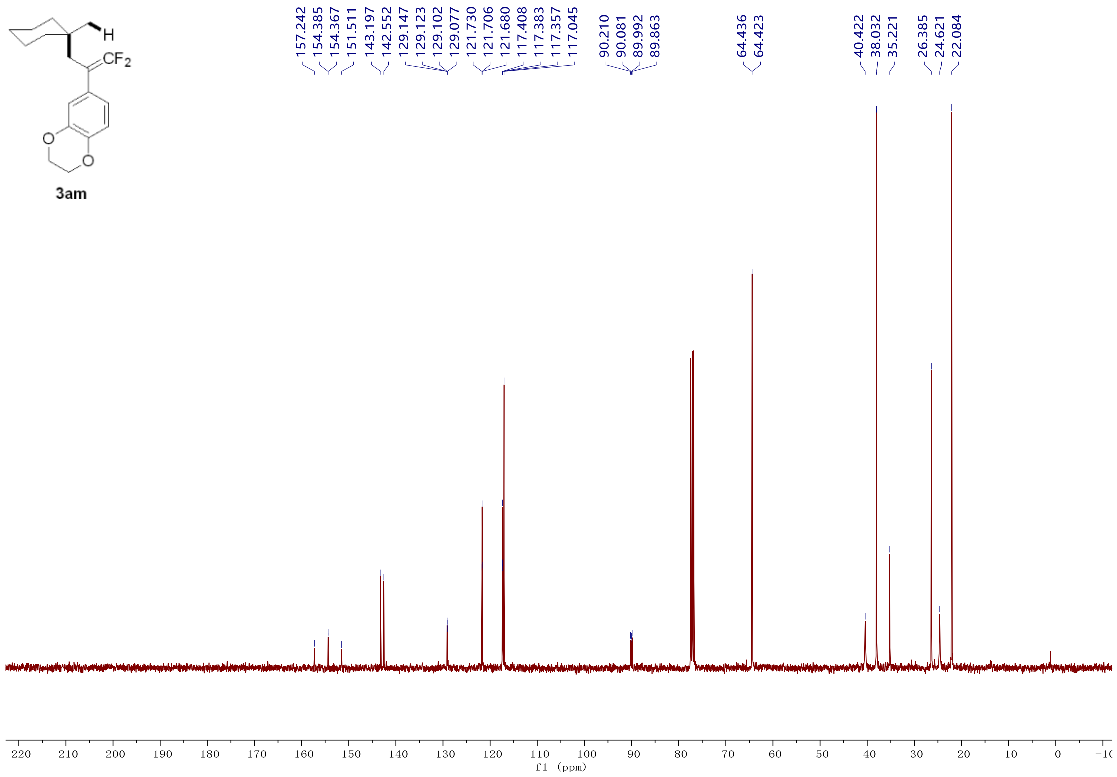

Supplementary Figure 246. ^{13}C NMR spectrum of compound **3am**

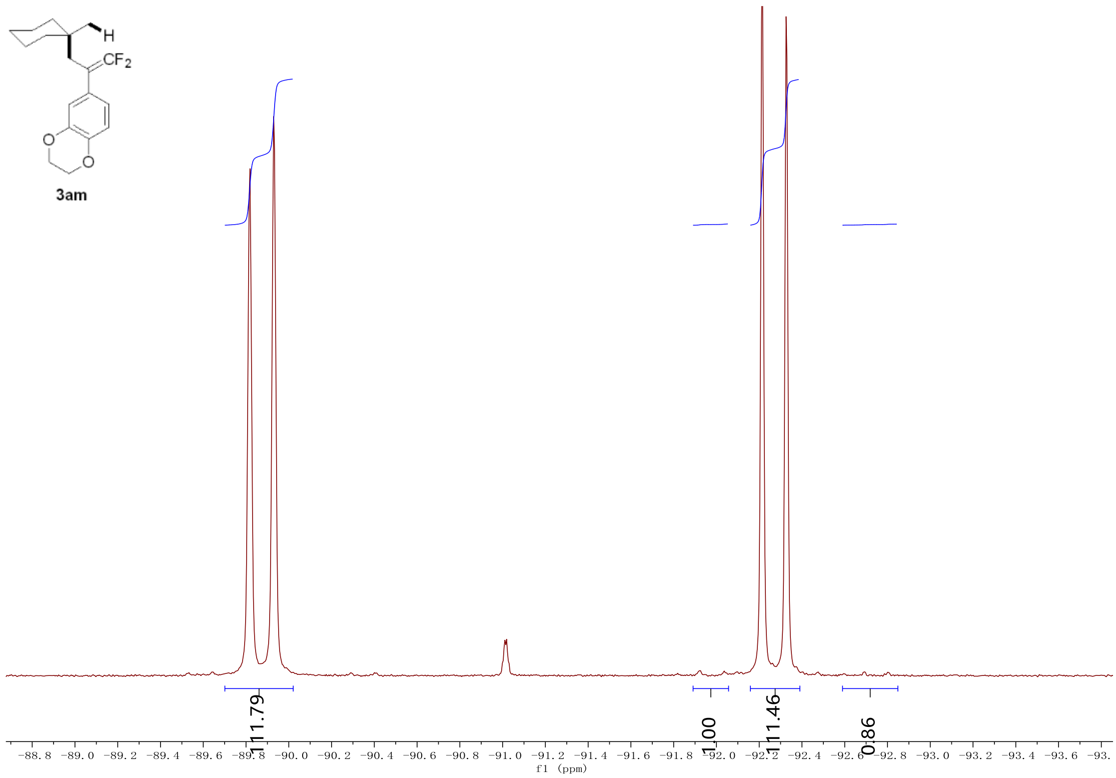

Supplementary Figure 247. Crude ^{13}C NMR spectrum of compound **3am** rr > 100 : 1

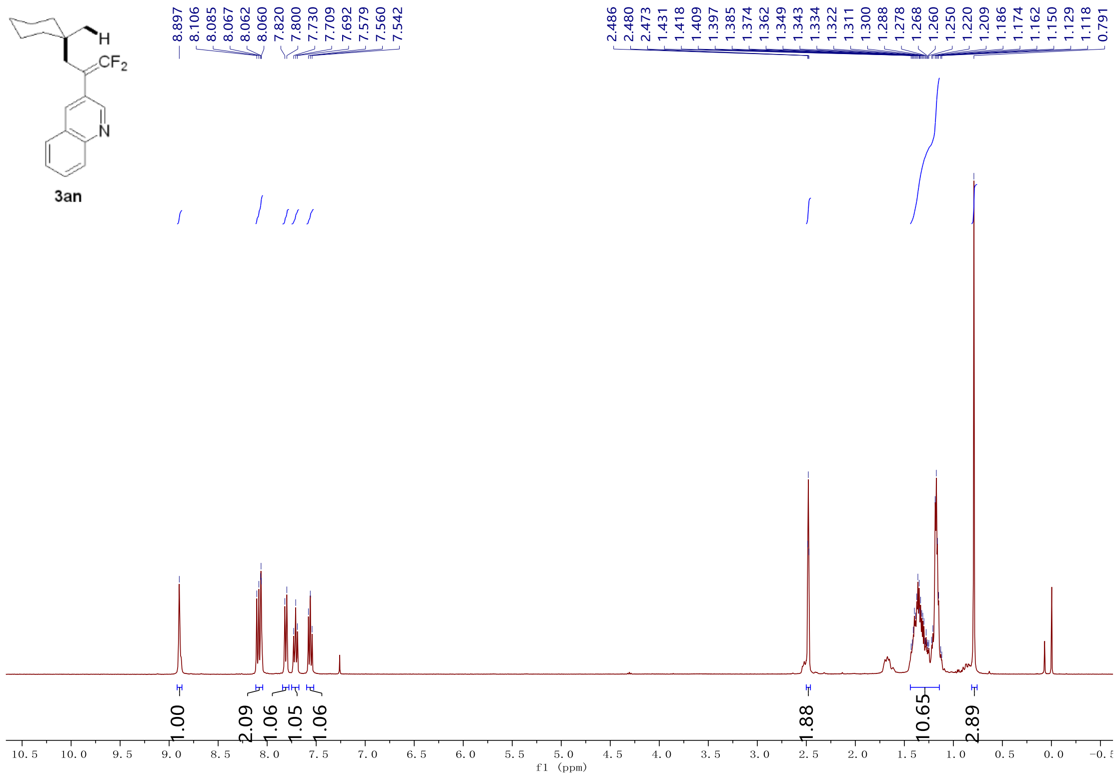

Supplementary Figure 248. ^{1}H NMR spectrum of compound **3an**

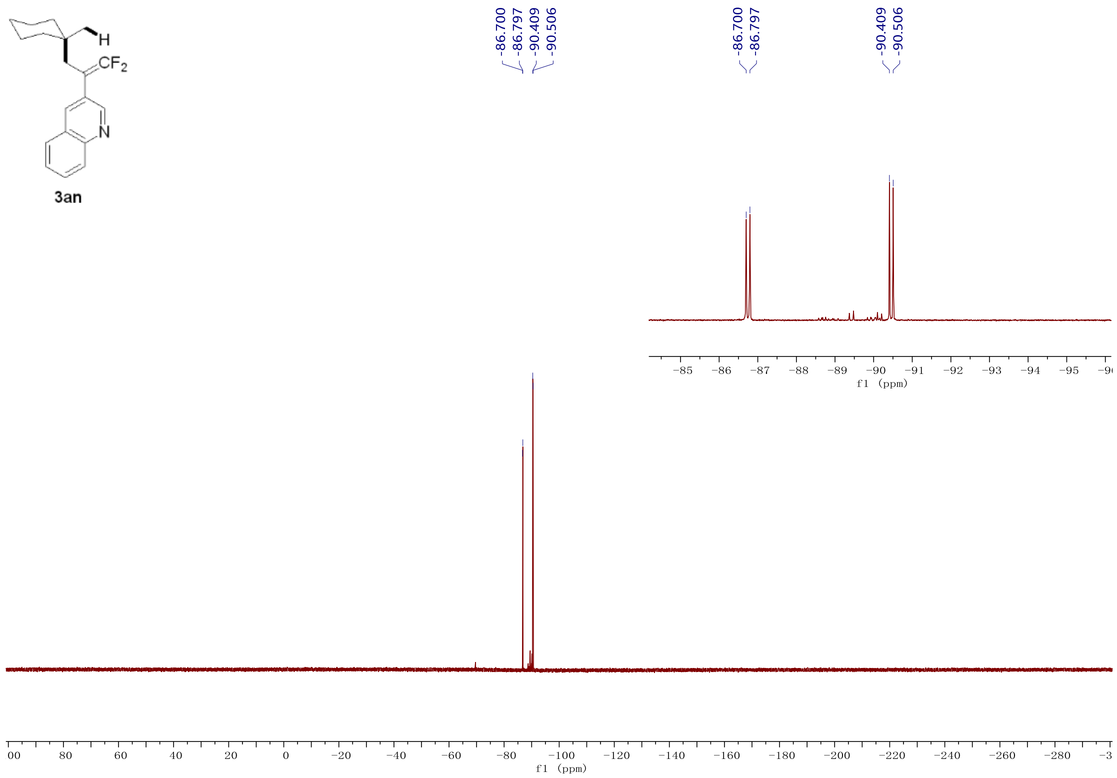

Supplementary Figure 249. ^{19}F NMR spectrum of compound **3an**

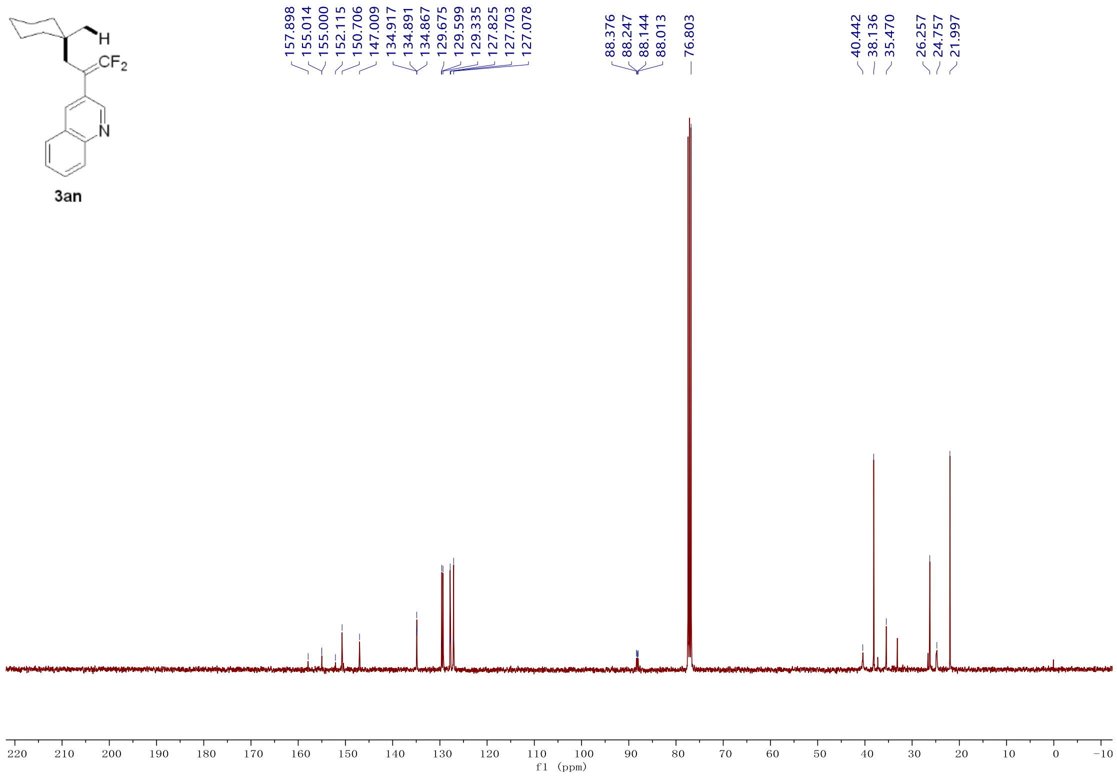

Supplementary Figure 250. ^{13}C NMR spectrum of compound **3an**

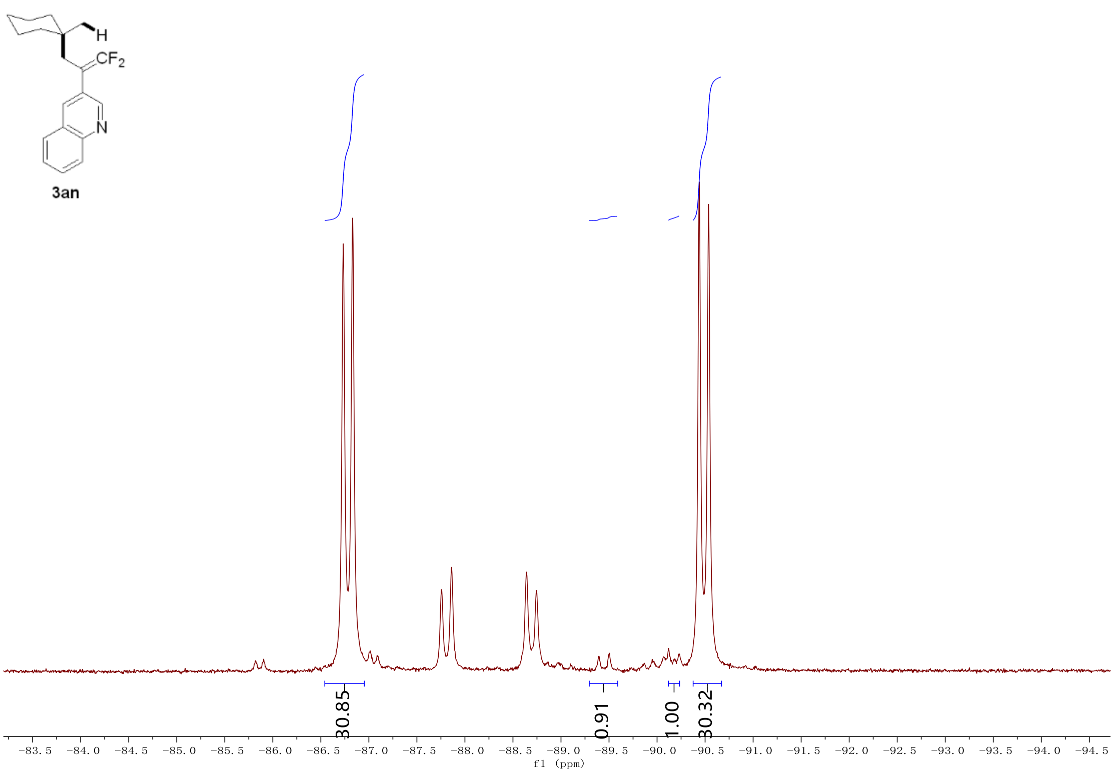

Supplementary Figure 251. Crude ^{19}F NMR spectrum of compound **3an** rr = 30 : 1

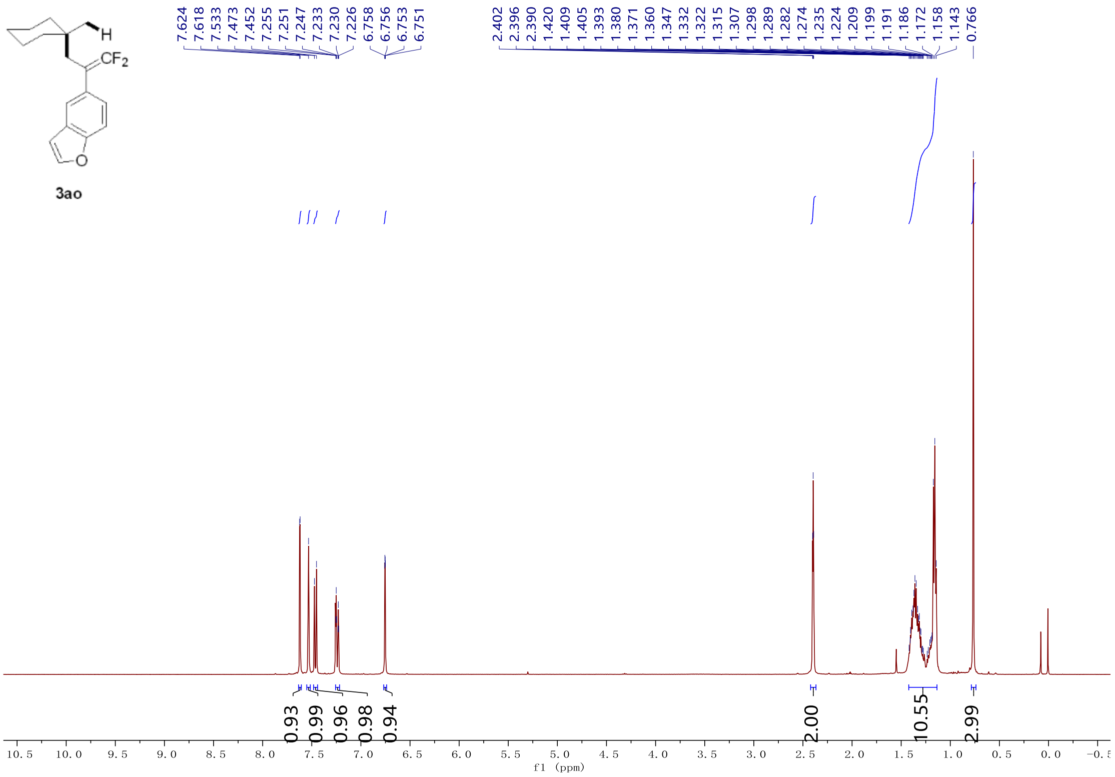

Supplementary Figure 252. ^{1}H NMR spectrum of compound **3ao**

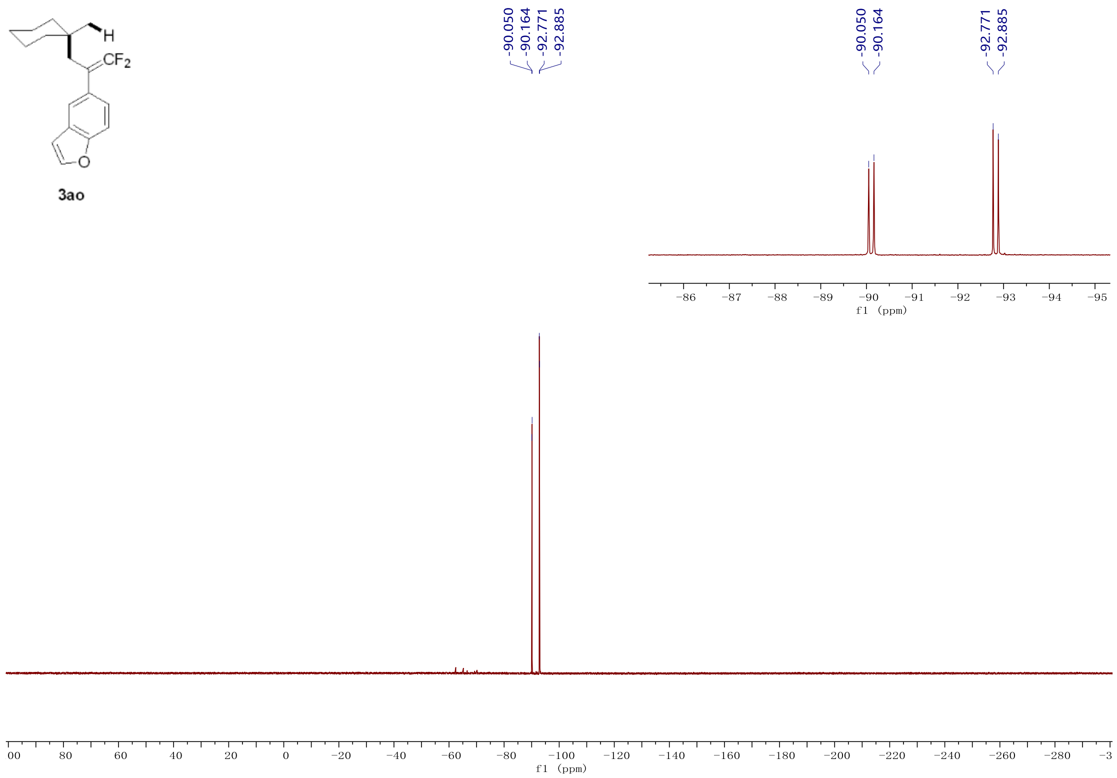

Supplementary Figure 253. ^{19}F NMR spectrum of compound **3ao**

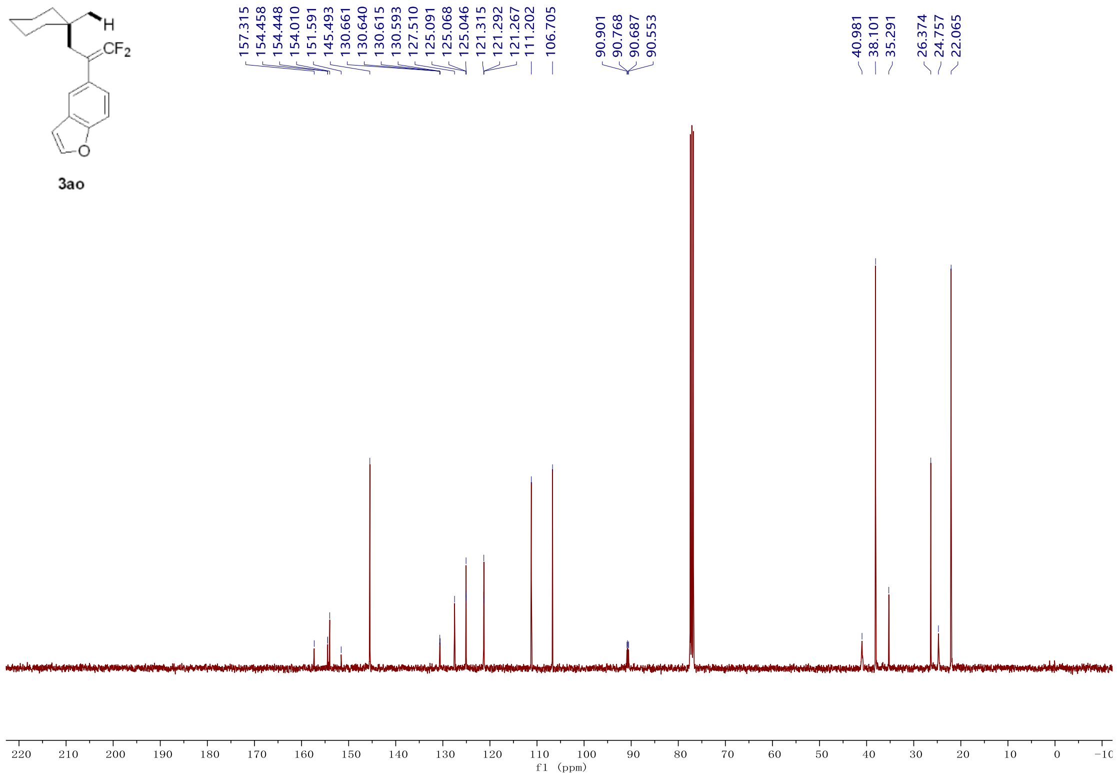

Supplementary Figure 254. ^{13}C NMR spectrum of compound **3ao**

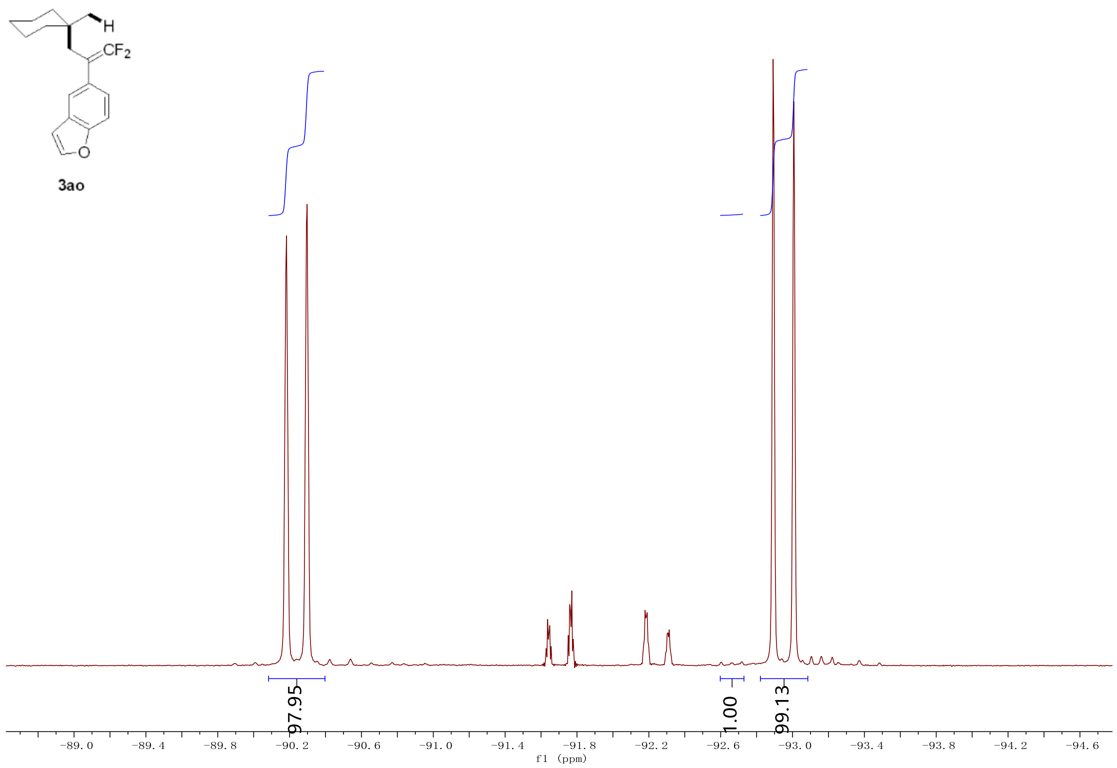

Supplementary Figure 255. Crude ^{19}F NMR spectrum of compound **3ao** rr > 100 : 1

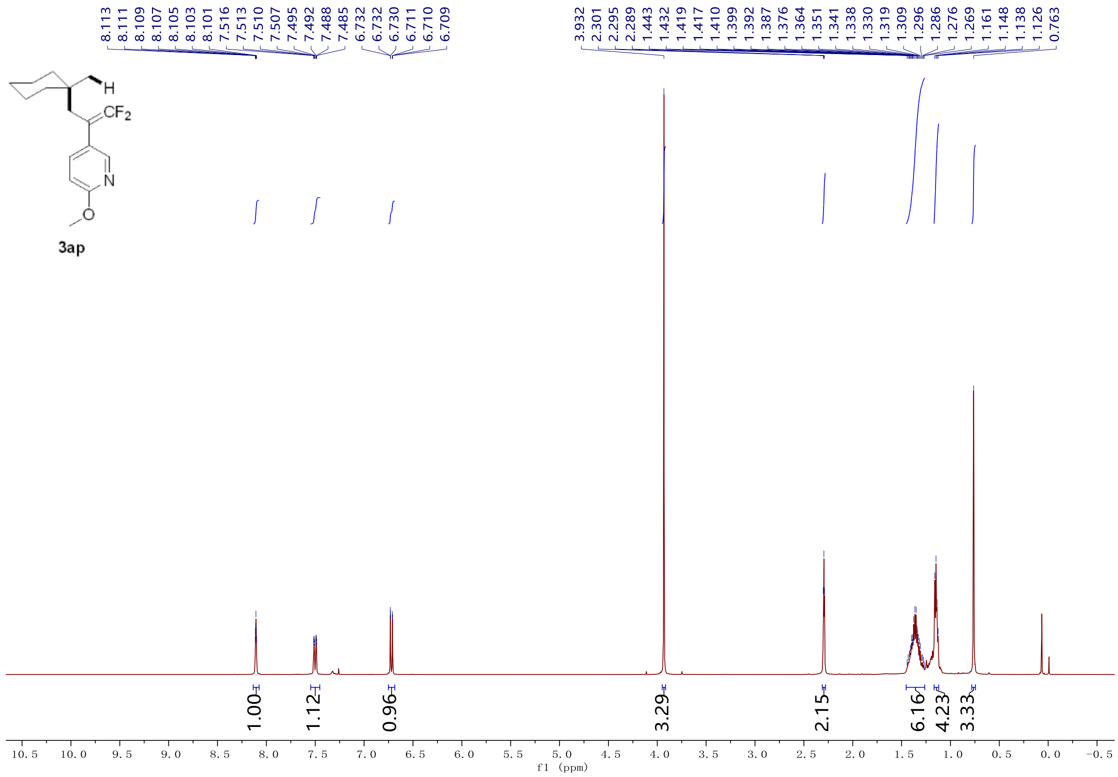

Supplementary Figure 256. ^{1}H NMR spectrum of compound **3ap**

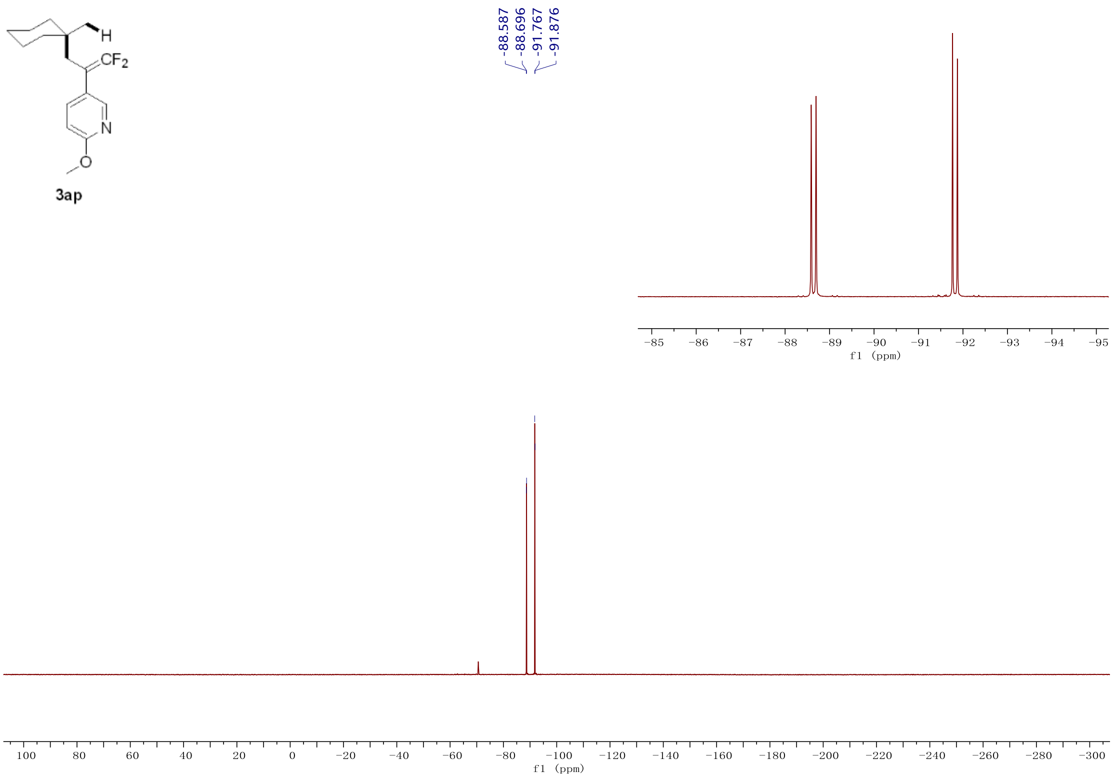

Supplementary Figure 257. ^{19}F NMR spectrum of compound **3ap**

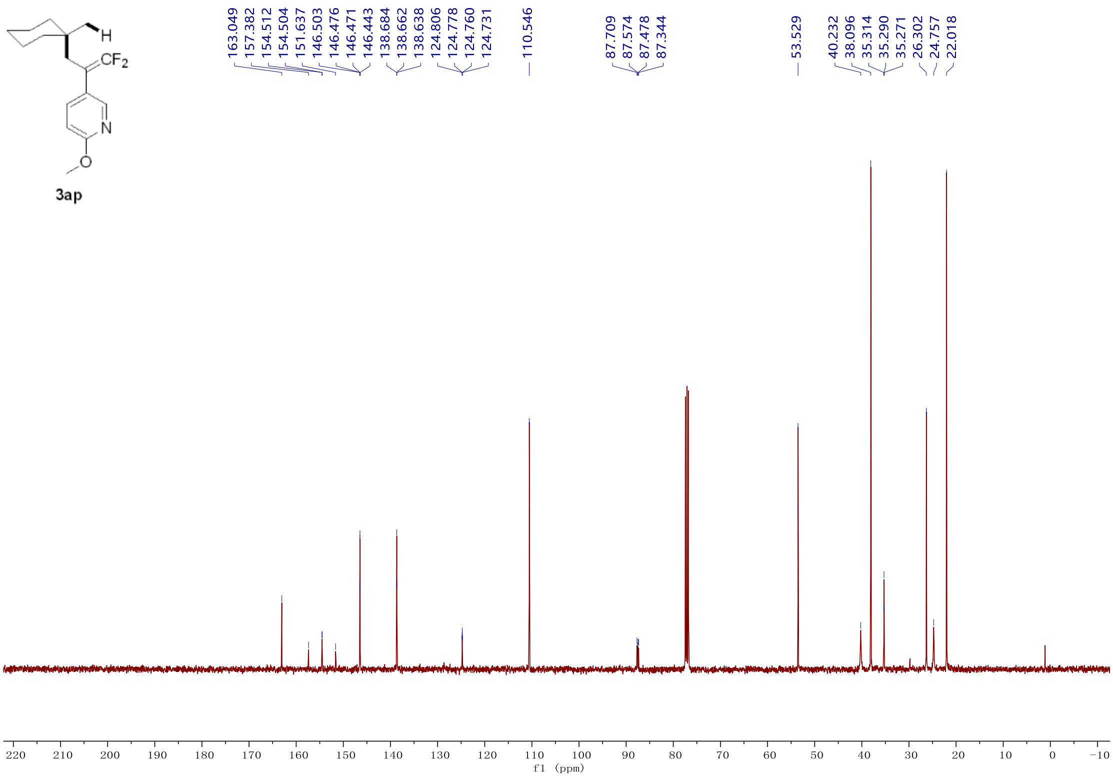

Supplementary Figure 258. ^{13}C NMR spectrum of compound **3ap**

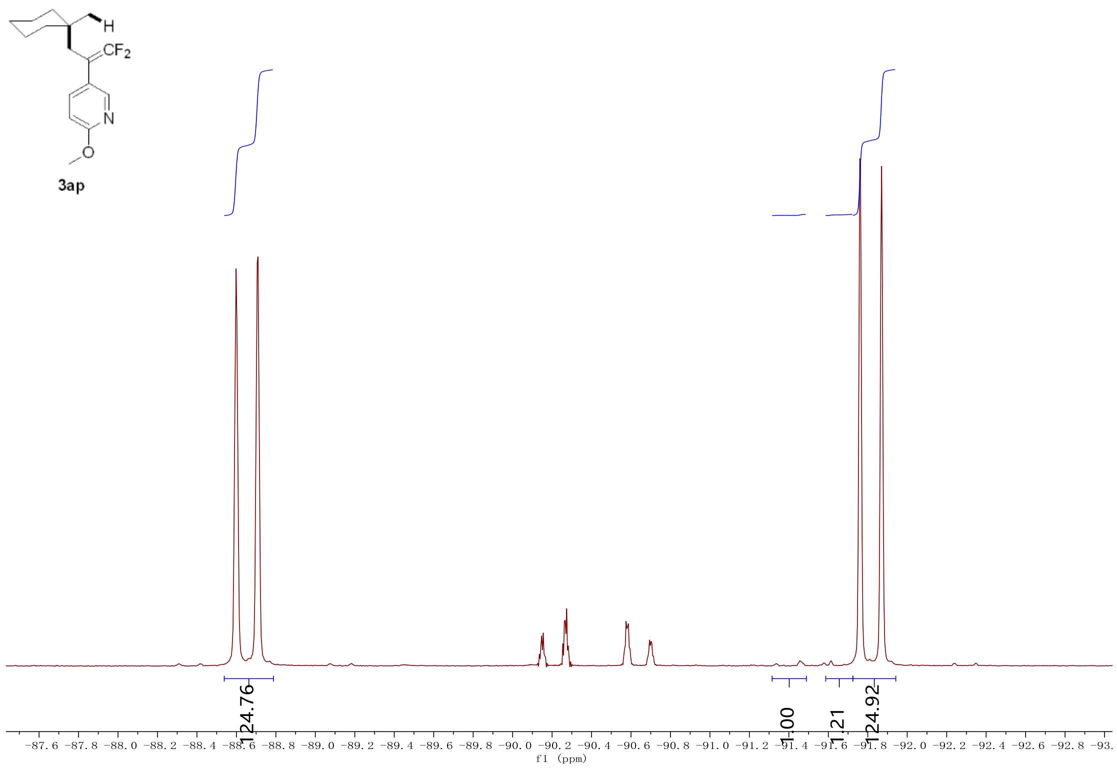

Supplementary Figure 259. Crude ^{19}F NMR spectrum of compound **3ap** rr > 100 : 1

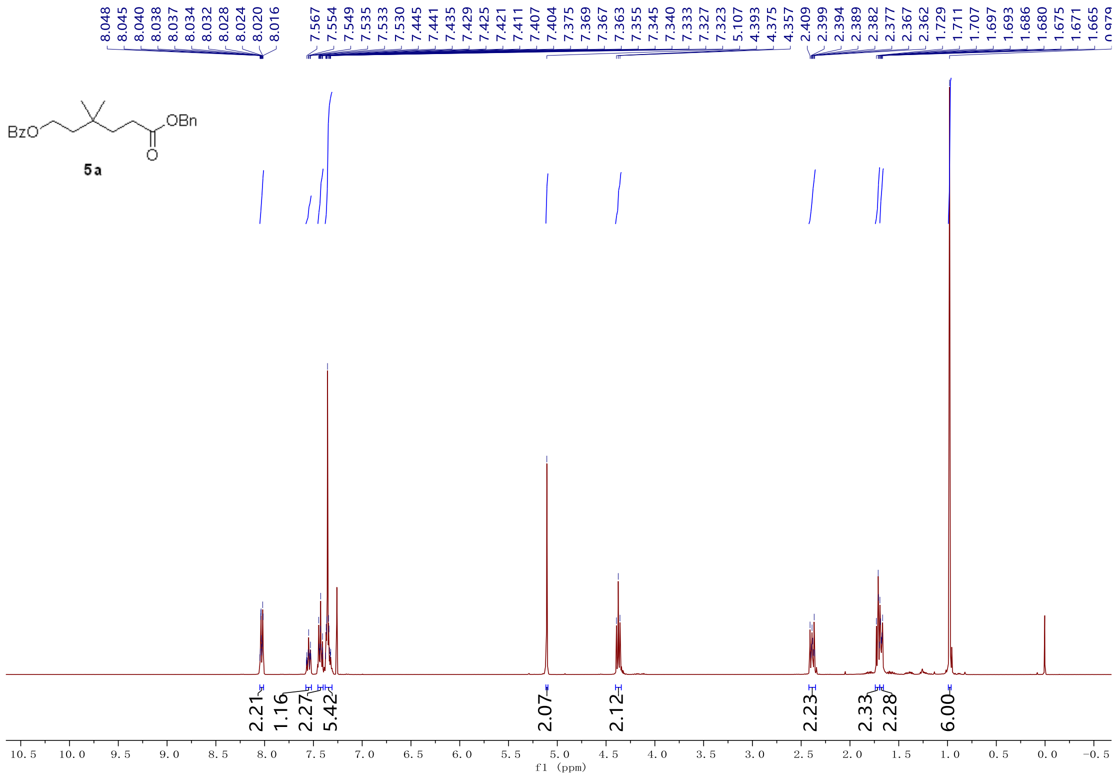

Supplementary Figure 260. ^{1}H NMR spectrum of compound **5a**

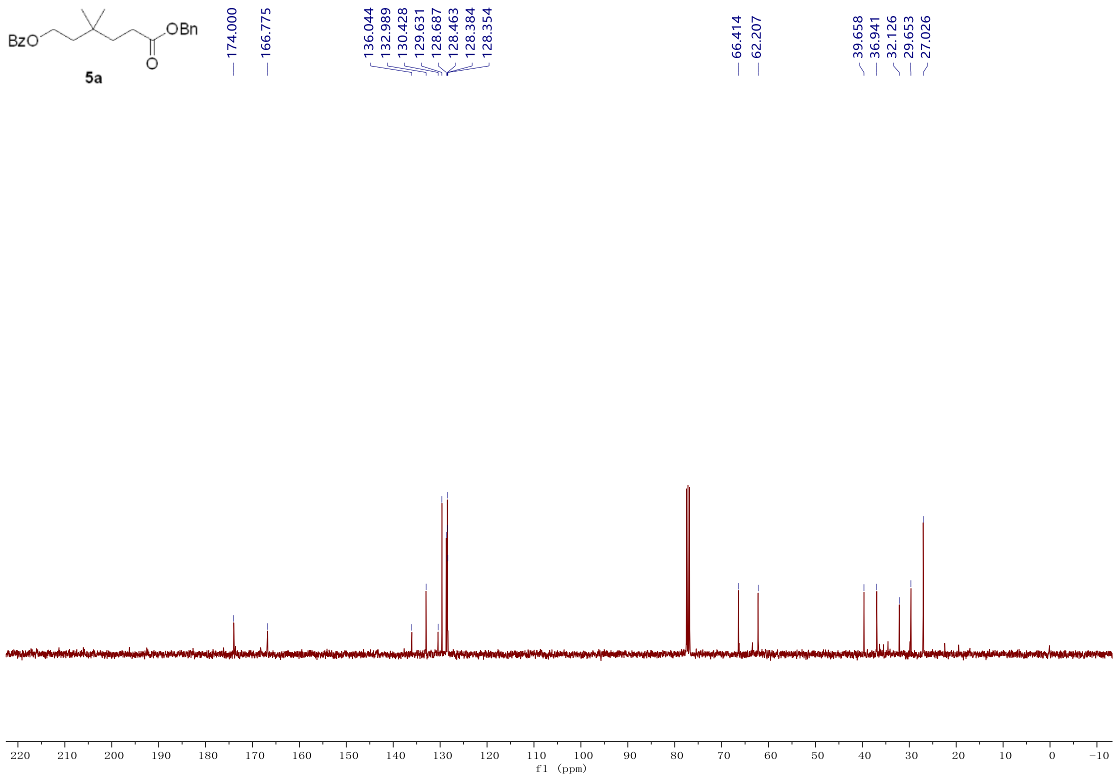

Supplementary Figure 261. ^{13}C NMR spectrum of compound **5a**

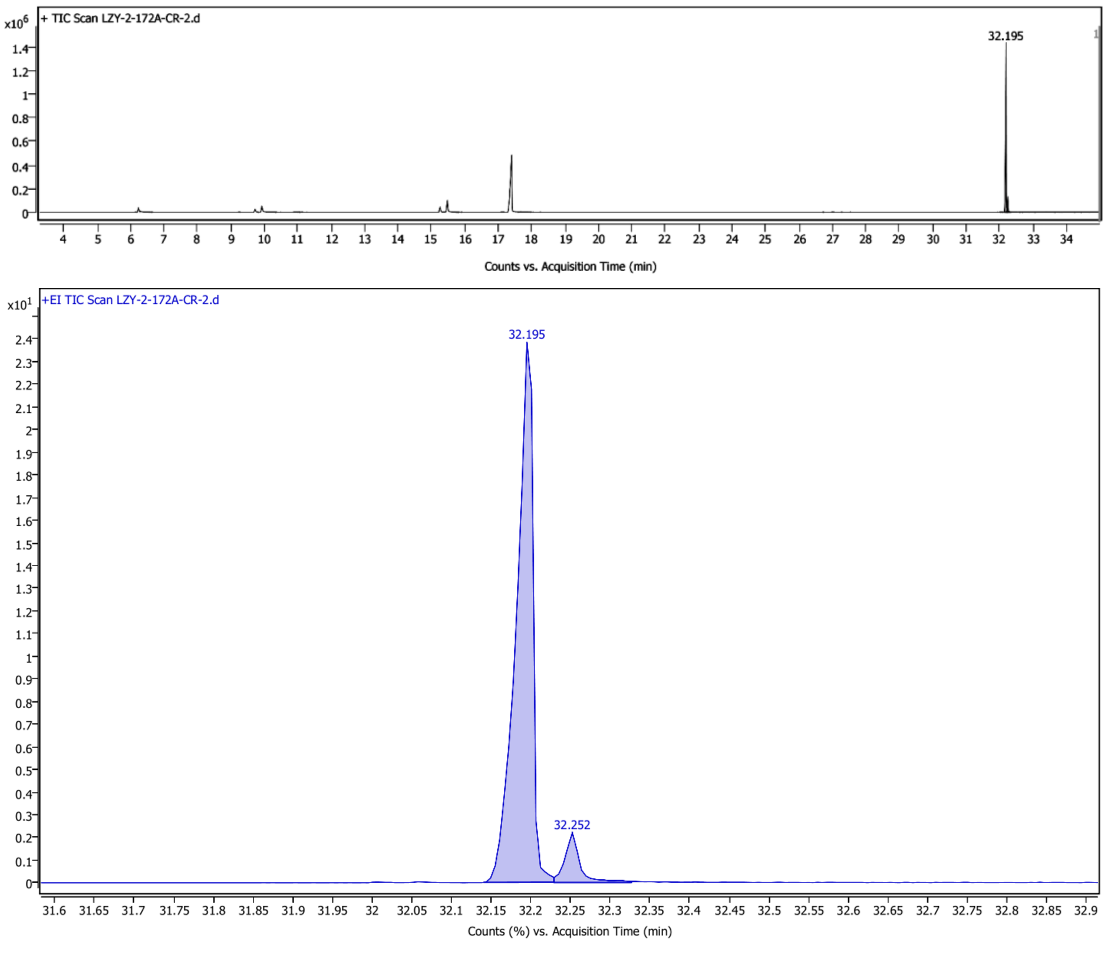

Chromatogram Peaks

Peak	Start	RT	End	Height	Area	Area %	SNR
1	32.140	32.195	32.229	1433372	2109749	100.00	
2	32.229	32.252	32.327	132644	171702	8.14	

Supplementary Figure 262. GC spectrum of compound **5a** rr = 12 : 1

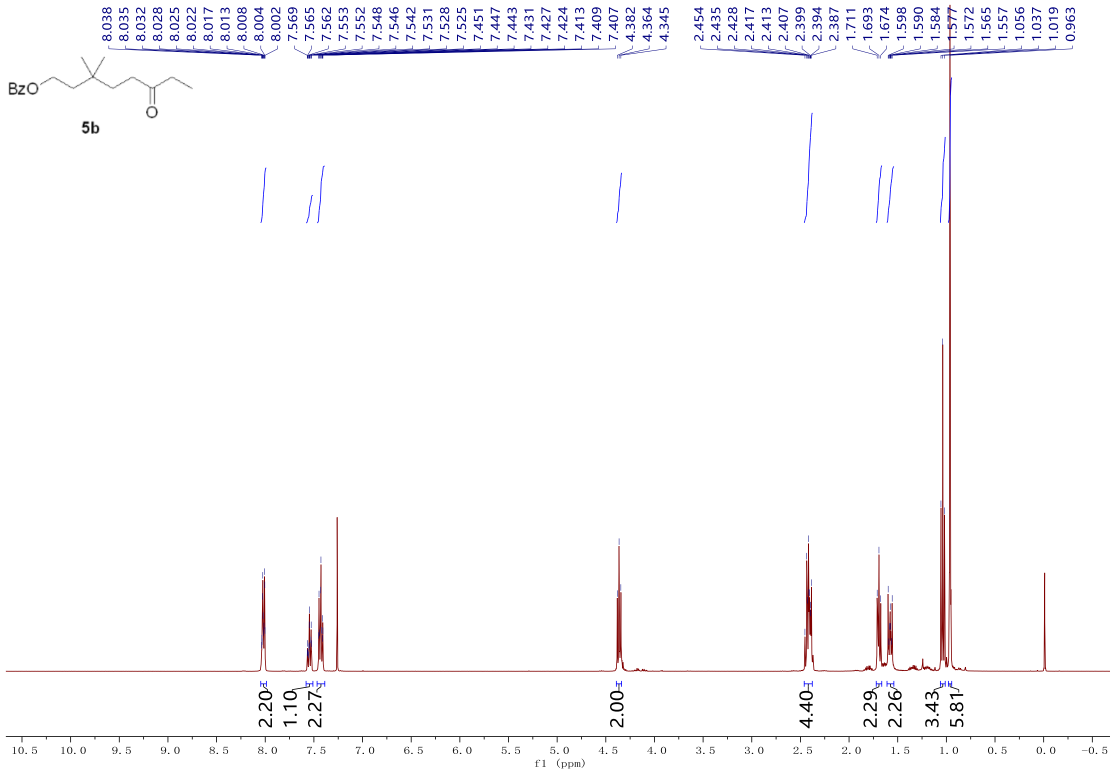

Supplementary Figure 263. ^{1}H NMR spectrum of compound **5b**

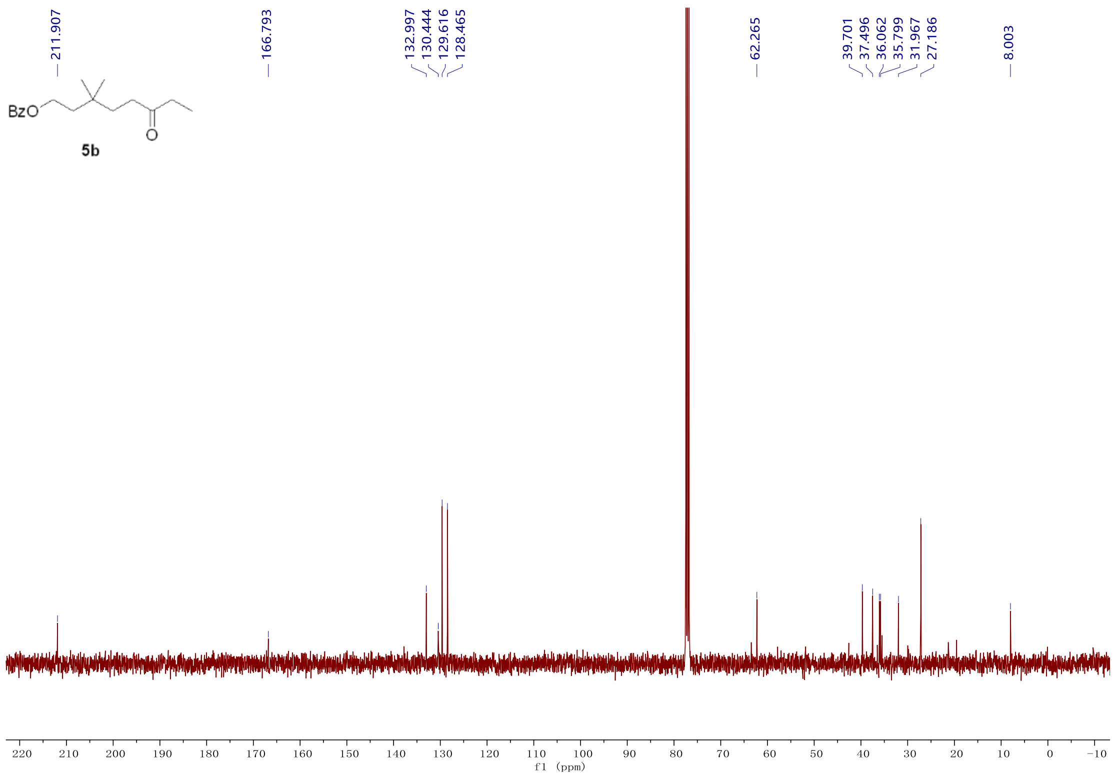

Supplementary Figure 264. ^{13}C NMR spectrum of compound **5b**

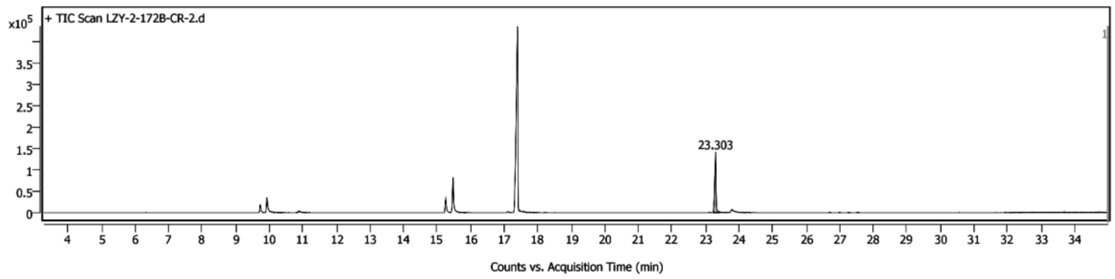

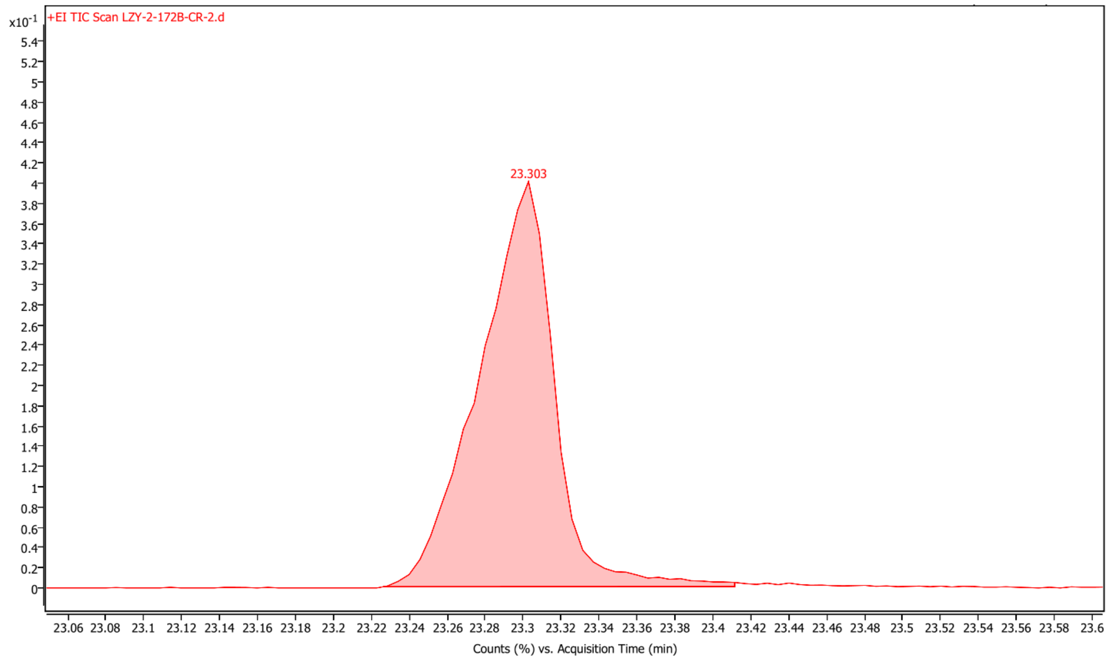

Chromatogram Peaks

Peak	Start	RT	End	Height	Area	Area %	SNR
1	23.226	23.303	23.412	140780	387666	100.00	

Supplementary Figure 265. GC spectrum of compound **5b** rr > 20 : 1

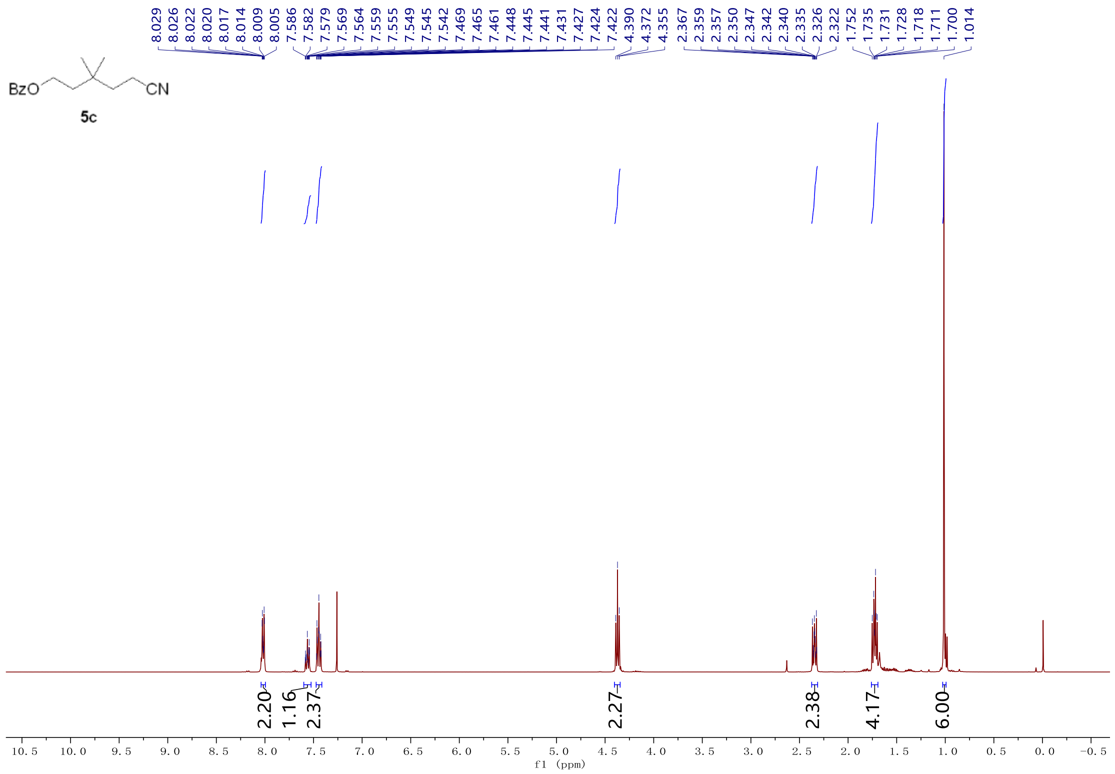

Supplementary Figure 266. ^{1}H NMR spectrum of compound **5c**

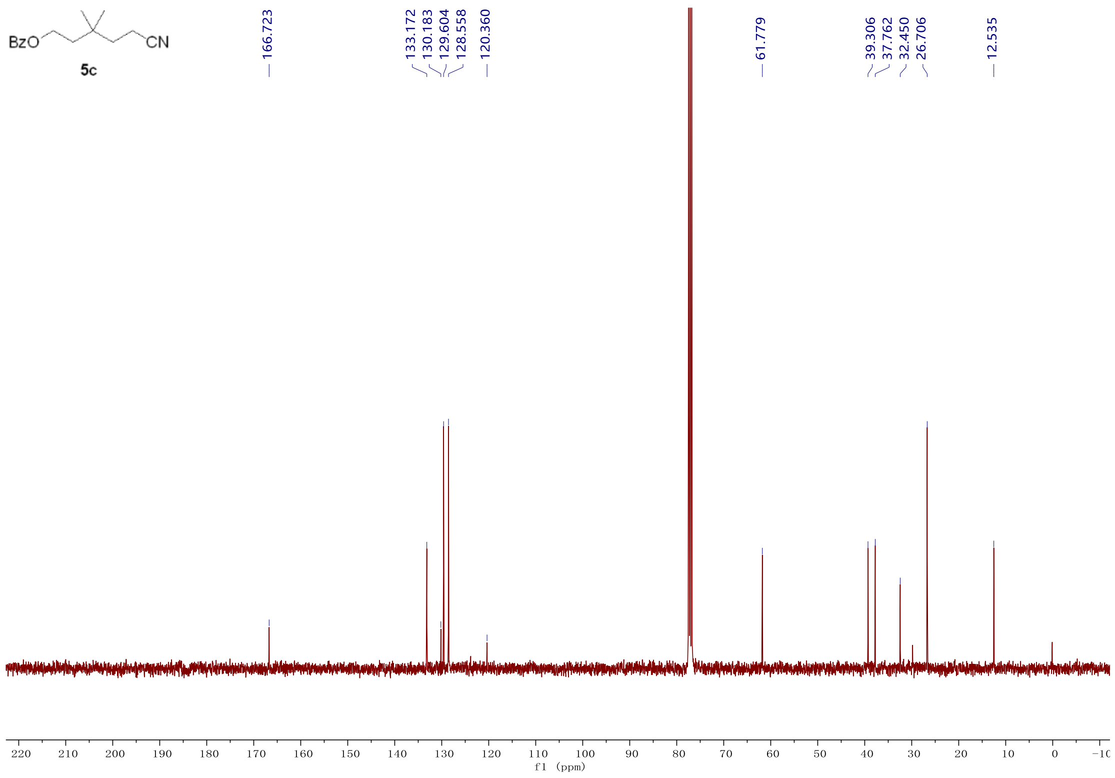

Supplementary Figure 267. ^{13}C NMR spectrum of compound **5c**

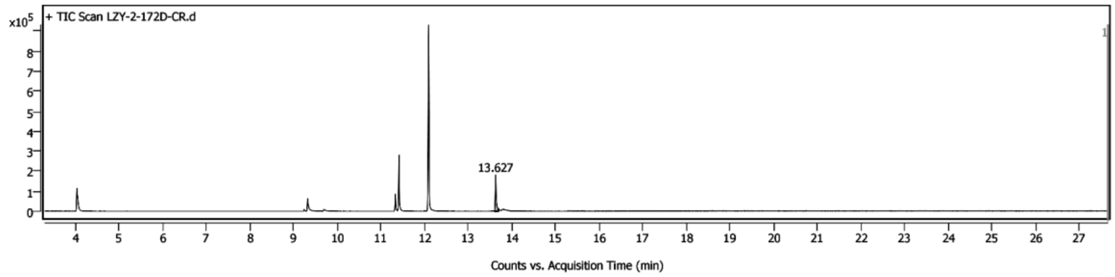

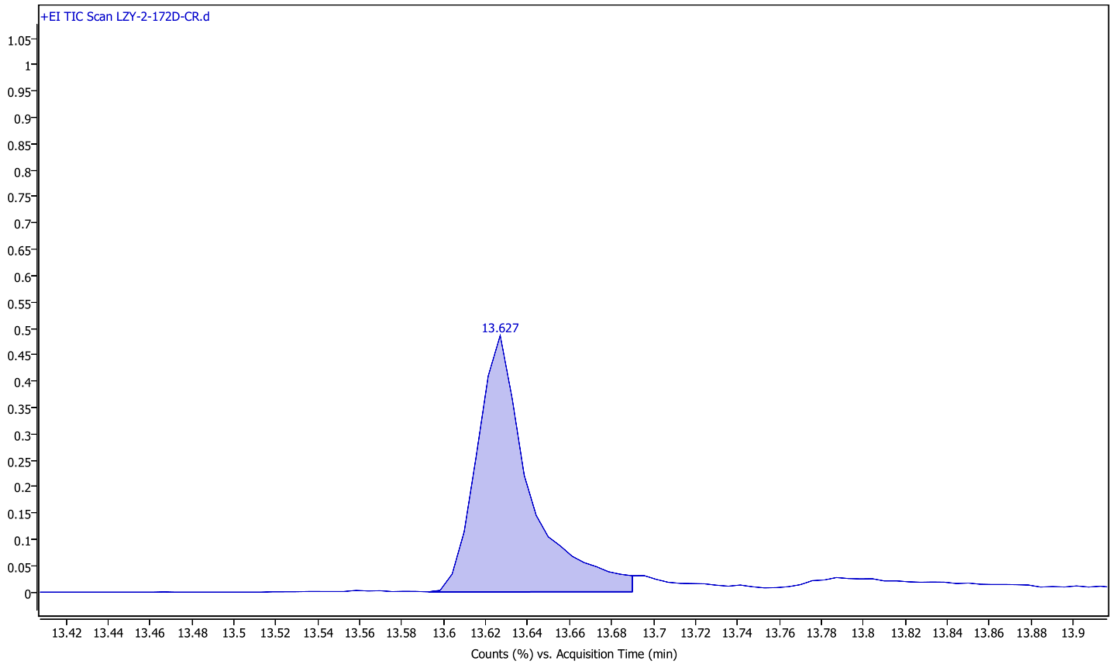

Chromatogram Peaks

Peak	Start	RT	End	Height	Area	Area %	SNR
1	13.593	13.627	13.690	182470	319563	100.00	

Supplementary Figure 268. GC spectrum of compound **5c** rr > 20 : 1

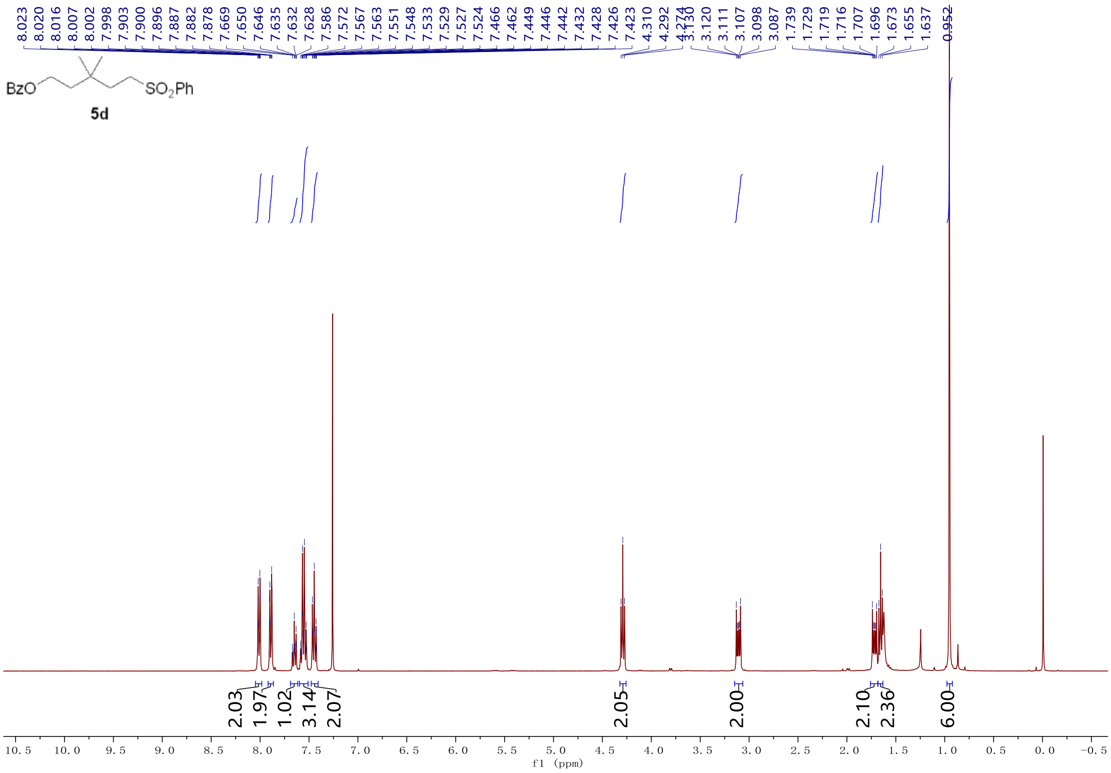

Supplementary Figure 269. ^{1}H NMR spectrum of compound **5d**

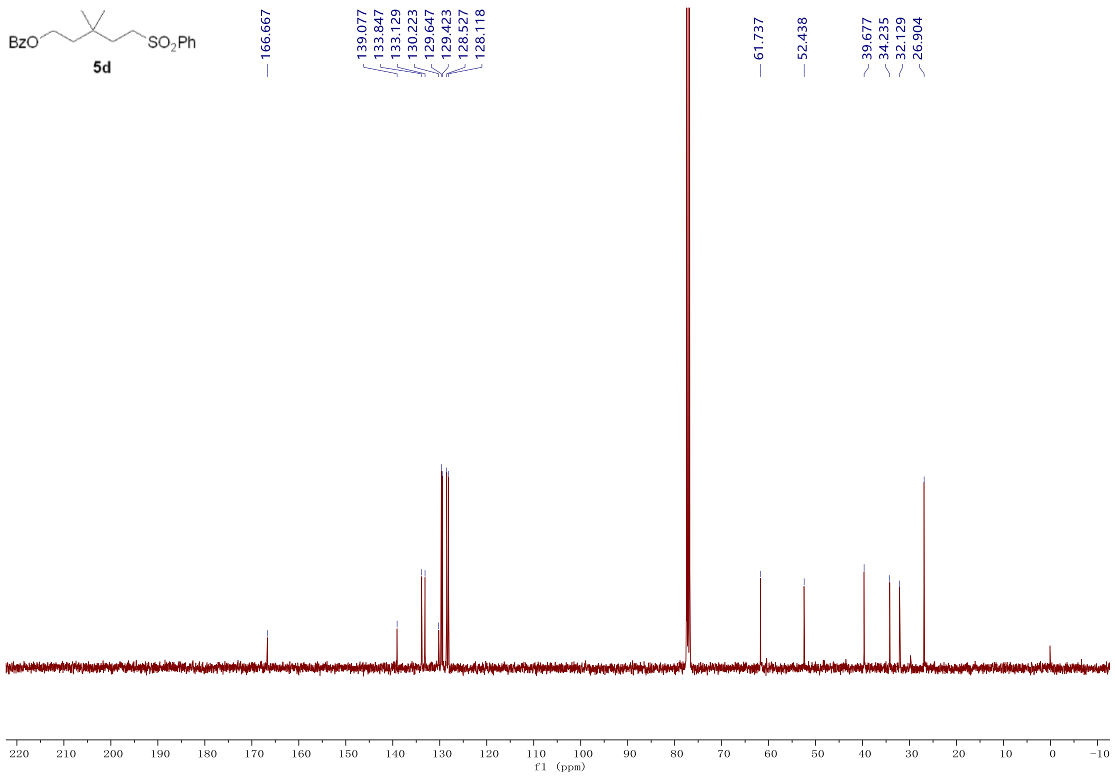

Supplementary Figure 270. ^{13}C NMR spectrum of compound **5d**

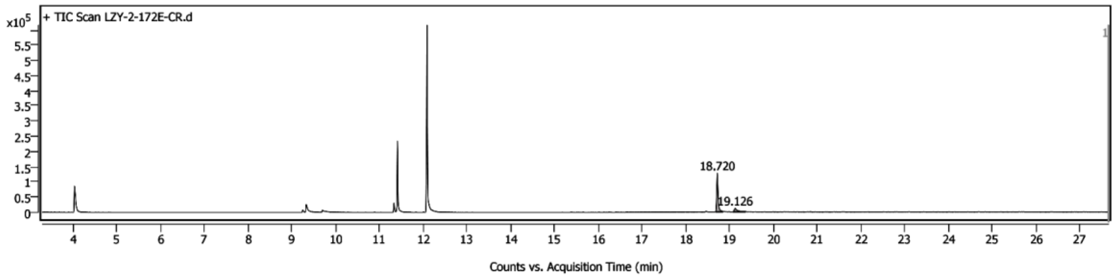

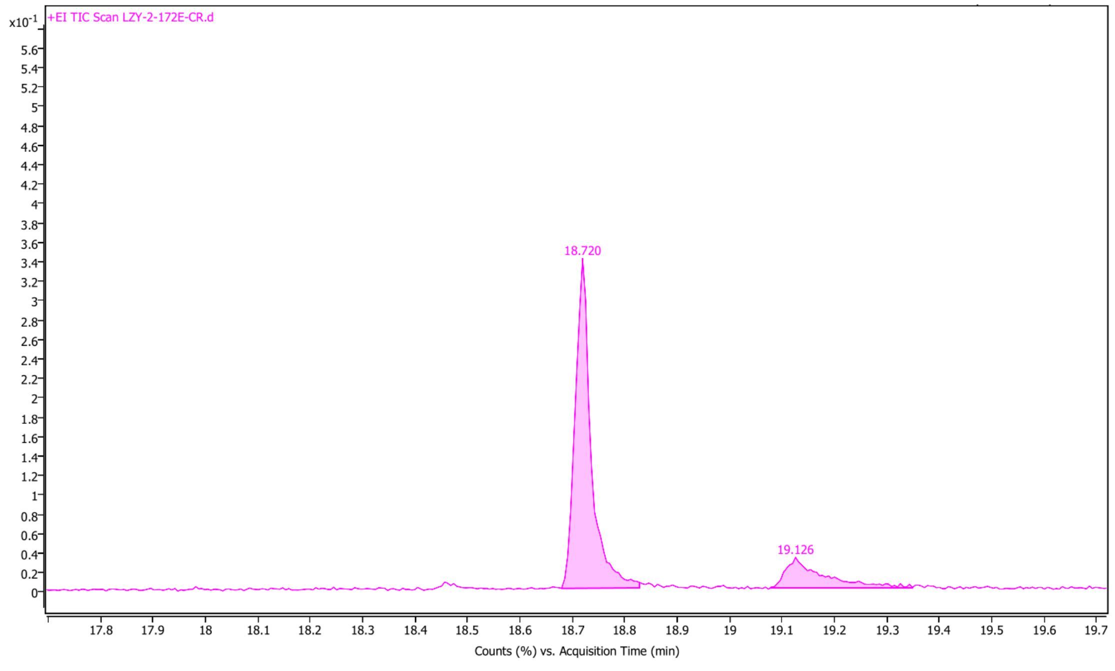

Chromatogram Peaks

Peak	Start	RT	End	Height	Area	Area %	SNR
1	18.680	18.720	18.828	127619	272711	100.00	
2	19.077	19.126	19.349	11816	57939	21.25	

Supplementary Figure 271. GC spectrum of compound **5d** rr = 5 : 1

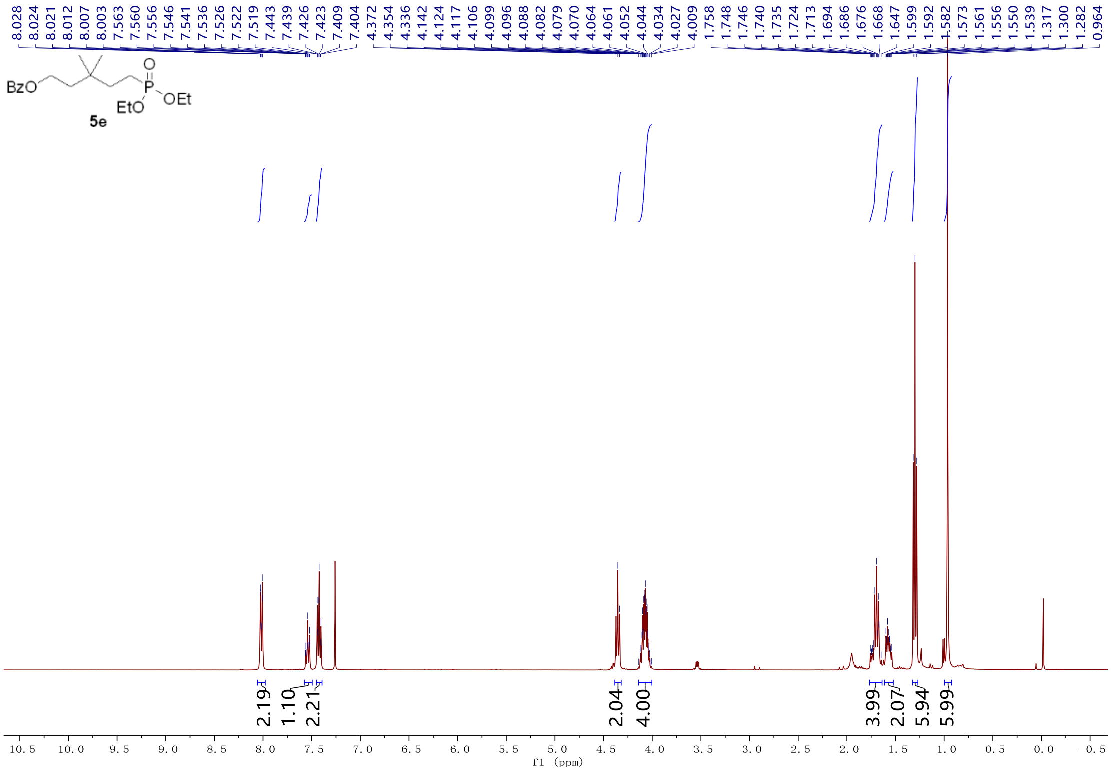

Supplementary Figure 272. ^{1}H NMR spectrum of compound **5e**

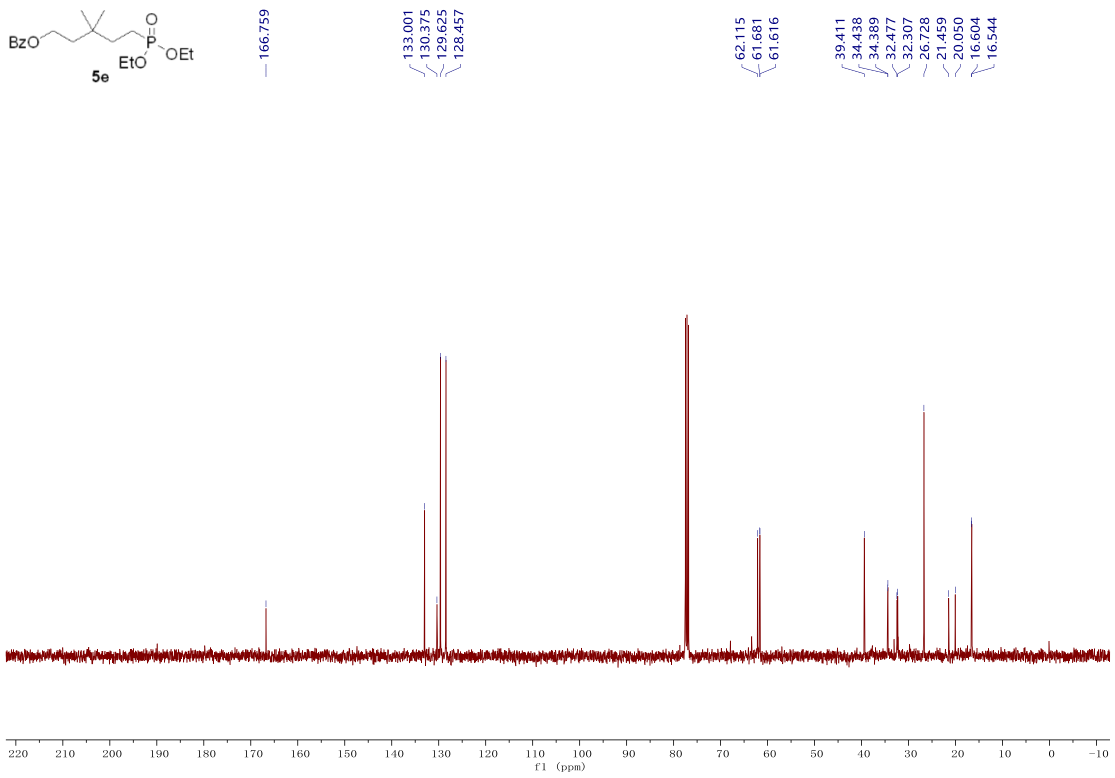

Supplementary Figure 273. ^{13}C NMR spectrum of compound **5e**

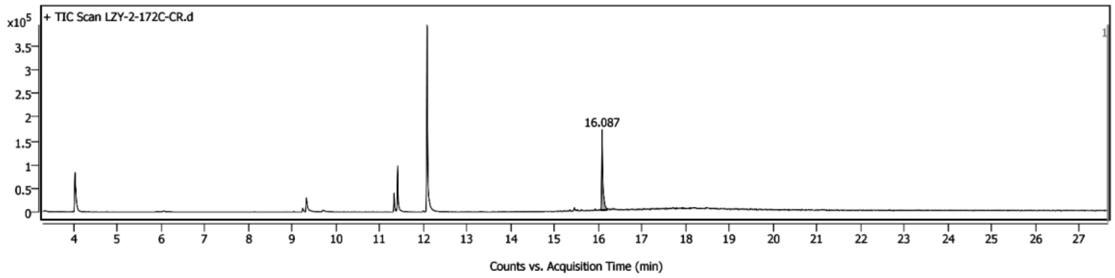

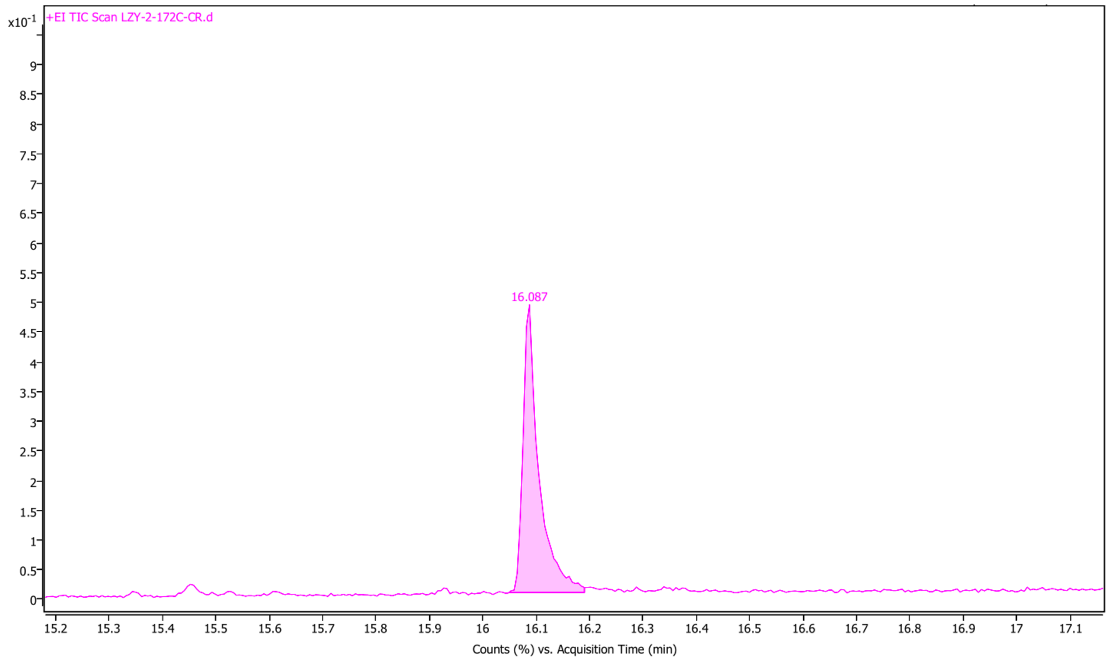

Chromatogram Peaks

Peak	Start	RT	End	Height	Area	Area %	SNR
1	16.048	16.087	16.190	170383	351700	100.00	

Supplementary Figure 274. GC spectrum of compound **5e** rr > 20 : 1

Supplementary References

[1] Ko, H. M., Lee, D. G., Kim, M. A., Kim, H. J., Park, J., Lah, M. S. & Lee, E. Total Synthesis of (−)-Blepharocalyxin D. *Org. Lett.* **9**, 141−144 (2007).

[2] Connolly, T., Wang, Z., Walker, M. A., McDonald, I. M. & Peese, K. M. Tandem Ring-Closing Metathesis/Transfer Hydrogenation: Practical Chemoselective Hydrogenation of Alkenes. *Org. Lett.* **16**, 4444−4447 (2014).

[3] Xie, Y., Sun, P. W., Li, Y., Wang, S., Ye, M. & Li, Z. Ligand-Promoted Iron(III)-Catalyzed Hydrofluorination of Alkenes. *Angew. Chem. Int. Ed.* **58**, 7097−7101 (2019).

[4] Passera, A. & Mezzetti, A., Mn(I) and Fe(II)/PN(H)P Catalysts for the Hydrogenation of Ketones: A Comparison by Experiment and Calculation. *Adv. Syn. Catal*. **361**, 4691−4706 (2019).

[5] Luo, N., Liao, J., Ouyang, L., Wen, H., Liu, J., Tang, W. & Luo, R. Highly pH-Dependent Chemoselective Transfer Hydrogenation of α,β-Unsaturated Aldehydes in Water *Organometallics* **38**, 3025−3031 (2019).

[6] Zhu, J.-B., Watson, E. M., Tang, J. & Chen, E. Y.-X. A synthetic polymer system with repeatable chemical recyclability. *Science* **360**, 398−403 (2018).

[7] Jiménez-Aquino, A., Vega, J. A., Trabanco, A. A. & Valdés C. A General Synthesis of α-Trifluoromethylstyrenes through Palladium-Catalyzed Cross-Couplings with 1,1,1-Trifluoroacetone Tosylhydrazone. *Adv. Syn. Catal.* **356**, 1079−1084 (2014).

[8] Lan, Y., Yang, F. & Wang, C. Synthesis of gem-Difluoroalkenes via Nickel-Catalyzed Allylic Defluorinative Reductive Cross-Coupling. *ACS Catal.* **8**, 9245–9251 (2018).

[9] Ichitsuka, T., Fujita, T., Arita, T. & Ichikawa, J. Double C–F Bond Activation through β-Fluorine Elimination: Nickel-Mediated [3+2] Cycloaddition of 2-Trifluoromethyl-1-alkenes with Alkynes. *Angew. Chem. Int. Ed.* **53**, 7564–7568 (2014).

[10] Xia, P.-J., Ye, Z.-P., Hu, Y.-Z., Song, D., Xiang, H.-Y., Chen, X.-Q. & Yang, H. Photocatalytic, Phosphoranyl Radical-Mediated N–O Cleavage of Strained Cycloketone Oximes. *Org. Lett.* **21**, 2658–2662 (2019).

[11] Yan, S.-S., Wu, D.-S., Ye, J.-H., Gong, L., Zeng, X., Ran, C.-K., Gui, Y.-Y., Li, J. & Yu, D.-G. Copper-Catalyzed Carboxylation of C–F Bonds with CO_2. *ACS Catal.* **9**, 6987–6992 (2019).

[12] Tang, L., Liu, Z.-Y., She, W. & C. Feng, Selective single C–F bond arylation of trifluoromethylalkene derivatives. *Chem. Sci.* **10**, 8701–8705 (2019).

[13] Sun, S.-Z., Börjesson, M., Martin-Montero, R. & Martin, R. Site-Selective Ni-Catalyzed Reductive Coupling of α-Haloboranes with Unactivated Olefins. *J. Am. Chem. Soc.* **140**, 12765–12769 (2018).

[14] Shi, Y., Si, H., Wang, P., Chen, S., Shang, S., Song, Z., Wang, Z. & Liao, S. Derivatization of Natural Compound β-Pinene Enhances Its In Vitro Antifungal Activity against Plant Pathogens *Molecules* **24**, 3144–3158 (2019).
